# Overcrowded Alkene Photo‐Redox‐Switches Based on Quinolinium/Carbene Building Blocks

**DOI:** 10.1002/anie.9975231

**Published:** 2026-05-21

**Authors:** Chris Burdenski, Patrick W. Antoni, Marcel E. Baumert, Leonie Ziemann, Samaresh C. Sau, Julian J. Holstein, Maria Castro, Nitin Kumar, Ofer Filiba, Igor Schapiro, Max M. Hansmann

**Affiliations:** ^1^ Fakultät für Chemie und Chemische Biologie Technische Universität Dortmund Dortmund Germany; ^2^ Fakultät für Physik Technische Universität Dortmund Dortmund Germany; ^3^ Institute of Chemistry The Hebrew University of Jerusalem Jerusalem Israel; ^4^ Research Center Chemical Sciences and Sustainability University Alliance Ruhr Bochum Germany

**Keywords:** carbenes, electron hole catalysis, photo‐switch, radicals, redox‐switch

## Abstract

The design and characterization of a novel class of overcrowded alkene‐based photo/redox switches are presented. Folded and twisted tetrasubstituted alkenes are prepared in a modular fashion by the combination of quinolinium salts and stable carbenes. The compounds show multi‐stimuli responsive properties: besides electrochemical redox‐switching, which allows C─C bond rotation via a two‐electron redox process, non‐symmetrical compounds enable photochemically driven *E*/*Z*‐switching. The different oxidation states can be isolated and fully characterized including structural changes verified by x‐ray diffraction. Excellent photostability for *E*/*Z*‐switching was observed in selected cases and is supported by UV–vis, NMR, and x‐ray data as well as computations. TD‐DFT calculations accurately predict the absorption properties of the *E*/*Z*‐photo‐switches and the substituent effects governing photo‐stationary states and half‐lifes. While the thermal *E*/*Z*‐back‐switching proceeds via a rather high energy barrier featuring a diradical transition state, electrochemical switching via the radical cation oxidation state allows instantaneous *E*/*Z*‐isomerization, which is catalytic in electrons (electron hole catalysis). The mechanism of the switching process is supported in detail by quantum chemical calculations including nonadiabatic molecular dynamics simulations.

## Graphical abstract

A modular synthesis of a new class of photo‐ and redox‐switches is described based on the combination of quinolinium salts and carbenes. Photochemical switching allows to selectively switch between folded *E/Z* isomers, while electrochemical switching allows to change between neutral, radical cation and dicationic oxidation states, which are all isolable oxidation states. Electron hole catalysis intercepts the photochemical switching, which is supported by theory.

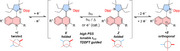

## 1 Introduction

**SCHEME 1:**
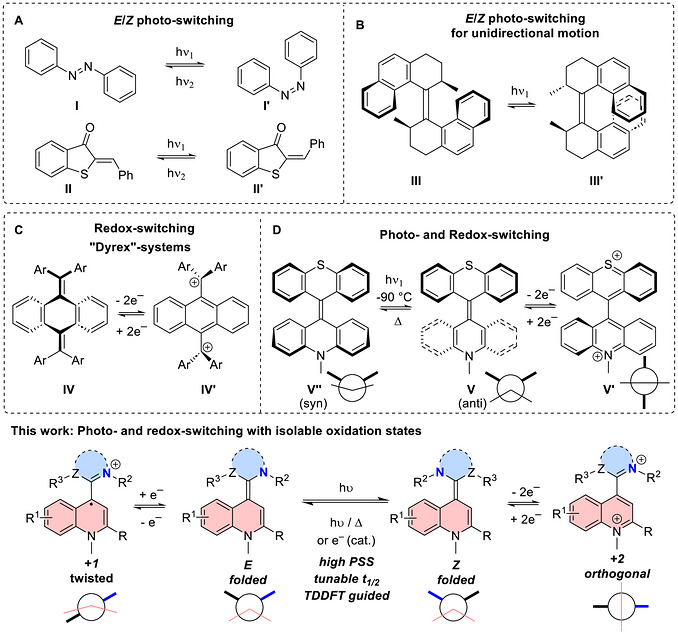
(A) Representative examples of well‐studied *E*/*Z* photo‐switches; (B) Overcrowded alkenes to drive unidirectional rotation; only the photo‐switching step is shown; (C) Dyrex systems and their conformational changes upon electron transfer; (D) Photo‐ and redox‐switching in overcrowded alkenes. This work: Combining photo‐ and redox‐switching in modular assembled overcrowded alkenes.

Stimuli‐driven reversible change of a molecular structure triggering motion on a molecular level is a highly vibrant area of research with plenty of applications. In particular light driven molecular switches, which can change between two or more states, have gained significant attention and achieved a multitude of different applications ranging from material science [1, 2, 3] over catalysis [4, 5, 6] and molecular machines [7, 8, 9, 10] to biological applications [11, 12]. Among the various types of photo‐switches, *E*/*Z*‐switches represent one of the most extensively investigated classes due to the pronounced geometrical and electronic changes accompanying their isomerization [13]. Archetypal examples are stilbenes [14] or azobenzenes (**I**), whose reversible *E*→*Z* photoisomerization (**I** → **I’**) has been exploited for decades as model systems (Scheme 1) [15, 16, 17]. Interestingly, Hecht and coworkers showed that electrons can catalyze the back‐switching process in azobenzenes via electron hole catalysis [18], which has been used in the context of redox‐switchable azobenzenes [19, 20, 21, 22, 23]. Beyond azobenzenes, structurally distinct systems such as indigo derivatives [24, 25], hemi(thio)indigo (**II** → **II’**) [26, 27], hydrazone‐based switches [28, 29, 30], spiropyranes [31], and dithienylethenes [32] have garnered significant attention, owing to their tunable absorption characteristics and improved fatigue resistance.

An important class of switches is based on tetrasubstituted, overcrowded alkenes. In combination with helical and point chirality, tetrasubstituted alkenes such as **III** have been utilized to drive light‐triggered monodirectional rotation first described by Feringa and coworkers in 1999 (Scheme 1B) [33]. In contrast to light excitation processes, redox driven switches remain relatively rare particularly to drive unimolecular rotation [34, 35], even though redox‐switchable supramolecular structures such as catenanes [36, 37] and rotaxanes [38, 39] were reported. Overcrowded alkenes have been utilized for electrochemical switching more frequently known as dynamic redox system (dyrex) exemplified with compound **IV** (Scheme 1C). In neutral **IV** the overcrowded substitution at the alkene unit affords a highly twisted conformation, which upon two electron oxidation results in a drastic change in geometry leading to the perpendicular orientation of the carbocation/anthracene planes (**IV′**) [40, 41, 42]. Often electrochemical bistability arises in dyrex systems originating from the pronounced difference between the oxidation potential of the folded neutral and twisted dicationic compounds, resulting in substantial alterations of the electronic structure and physical properties [43, 44, 45]. The combination of systems, that can be switched not only photochemically but also electrochemically, is rare [46, 47, 48]. Recently Hein, Feringa, and coworkers published a series of manuscripts about the photo‐redox switching of thioxanthene and acridine based overcrowded alkenes (**V**) (Scheme 1D) [49, 50, 51]. In these systems, photo‐switching has to be performed at low temperatures (−90°C) since the energy barrier for switching between the metastable *syn* (**V″**) to the thermodynamic *anti*‐conformer **V** is very low. Since the metastable photo‐generated state is not isolable and also the radical cation oxidation state remained elusive, further improvements are highly desirable to access more robust photo‐electro switches with well‐defined redox‐states ideally featuring negative photochromism properties [52, 53, 54]. Here, we present a design and synthetic approach to switches in which overcrowded alkenes are assembled by the combination of stable carbenes with quinolinium salts. Importantly, selected examples of the family of compounds qualify both as stable *E*/*Z*‐photo‐ as well as redox‐switches with three well defined, room‐temperature stable and isolable oxidation states. The isolation of the radical cation oxidation state allows to study in detail the effect of electron hole catalysis in photo‐switching processes.

## 2 Results and Discussion

**SCHEME 2:**
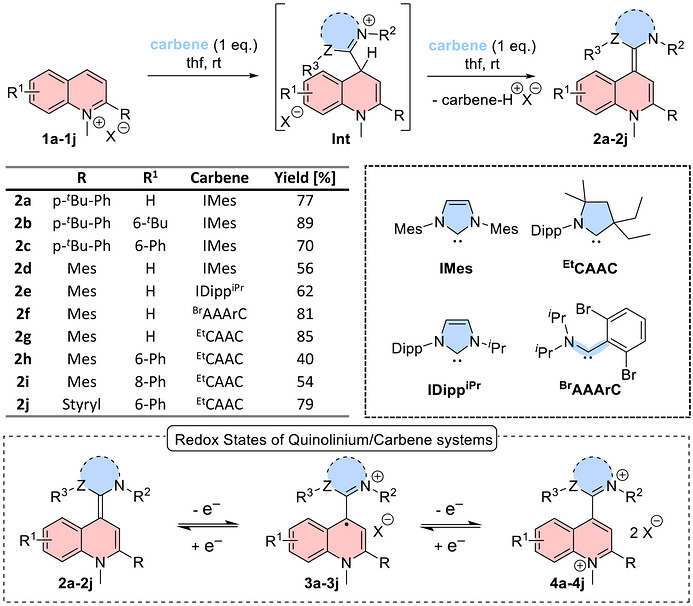
Synthesis of quinolinium/carbene switches and their corresponding neutral (**2**), radical cation (**3**) and dication (**4**) oxidation states. Mes = 2,4,6‐trimethylphenyl; Dipp: 2,6‐diisopropylphenyl.

The synthesis of photo‐ and electro‐switches was targeted by the addition of stable carbenes to quinolinium salts, wherein one equivalent of the carbene acts as a nucleophile to form intermediate **Int**, while a second equivalent acts as a base to afford the neutral compound **2** (Scheme 2). In contrast to our previously reported synthetic approach, which focused on symmetrical redox systems [55, 56, 57, 58]; here, the concept of combining quinolinium salts and unsymmetrical carbenes should afford non‐symmetric tetrasubstituted alkenes featuring *E*/*Z* isomers and hence allow for the first time both electrochemical as well as photochemical switching processes. We started with the symmetrical system employing IMes [59] and 2‐aryl‐substituted *N*‐methyl quinolinium salts to give access to compounds **2a–2d**. Despite the fact that C–C bond rotation of **2a–2d** formally produces identical and indistinguishable systems, compounds **2a–2d** were synthesized as reference compounds for electrochemical switching and spectroscopic investigations. Introduction of a substituent at the C2 position directed carbene addition selectively to the 4‐position. Otherwise, mixtures of the 2‐ and 4‐isomers were obtained. In general, the synthesis with differently substituted quinolinium salts and IMes afforded the desired quinoline/IMes hybrids **2a–2d** in moderate to excellent yields (56%–89%).

In order to develop photochemical switches, we selected an unsymmetrical imidazole based carbene (IDipp*^i^* ^Pr^) featuring *N*‐Dipp and *N*‐*i*Pr moieties to afford **2e**. Unfortunately, the ^1^H‐NMR signals of **2e** were significantly broadened at ambient temperature, and only sharpened upon cooling to −80°C (see Figure S58), which might indicate a fast rotation process or an inversion on a pyramidalized N‐fragment. This was also observed for **2a** but in a less pronounced fashion. We also investigated acyclic amino(haloaryl)carbenes (AAArC) recently described by Landais et al. [60] featuring strong π‐acceptor properties to reduce bond polarization and to increase C─C double bond character. Our standard conditions afforded the acyclic compound **2f** in a good yield (81%) as a mixture of *E*/*Z* (89:11) isomers in which ***E*‐2f** was structurally verified by x‐ray diffraction (see Figure S169). Finally, we selected the family of cyclic (alkyl)amino carbenes (CAACs) introduced by Bertrand et al. [61, 62, 63], which feature strong π‐acceptor properties. Addition of ^Et^CAAC to various quinolinium salts afforded the desired hybrid systems **2g–2j** in typically good to high yields as a mixture of *E*/*Z* isomers (Scheme 2).

**FIGURE 1:**
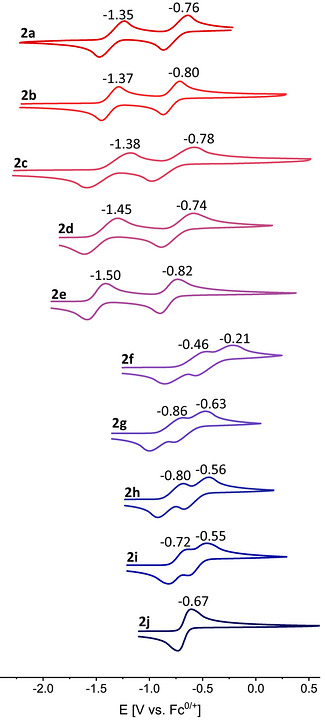
Comparison of normalized cyclic voltammograms in thf (0.1 M *n*‐Bu_4_NPF_6_) at room temperature; scan rate 200 mV s^−1^; referenced internally against ferrocene.

Cyclic voltammetry (CV) experiments were performed to investigate the redox properties of all ten compounds **2a–2j** (Figure 1). **2a–2i** show two distinct quasi‐reversible oxidation events (three‐stage redox system) with varying potential separation. The substituents in the 2‐ and 6‐positions of the quinoline have only a small influence on the electrochemical potentials, while the type of carbene strongly affects the redox potentials (Figure 1). The weak π‐acceptor properties of imidazole based carbenes (**2a–2e**) result in highly reducing redox potentials of the neutral hybrids (−1.50 V to −1.35 V vs. Fc/Fc^+^).

The stronger π‐accepting properties of AAArC and CAAC significantly shift the redox potentials for the first oxidation towards more positive redox potentials. The first oxidation of the AAArC hybrid **2f** (−0.46 V) was the most positively shifted by ca. 1 V in comparison to the IMes series. The CAAC series (**2g–2j**) features redox potentials for the first oxidation around −0.67 to −0.86 V (Figure 1A) slightly more negative than **2f**. This is attributed to the fact that in acyclic AAArC **2f** the R_2_N‐moiety can rotate out of the plane (see x‐ray structure, Figure S169), which is not possible when the nitrogen atom is embedded in a cyclic structure (CAAC). Interestingly, substitution at the C2 position from mesityl (**2g**) to styrenyl (**2j**) induces a strong potential compression close to a potential inversion [64], manifested by a single CV wave, indicative of a concerted two‐electron transfer.

### 2.1 Redox‐State Switching

**FIGURE 2:**
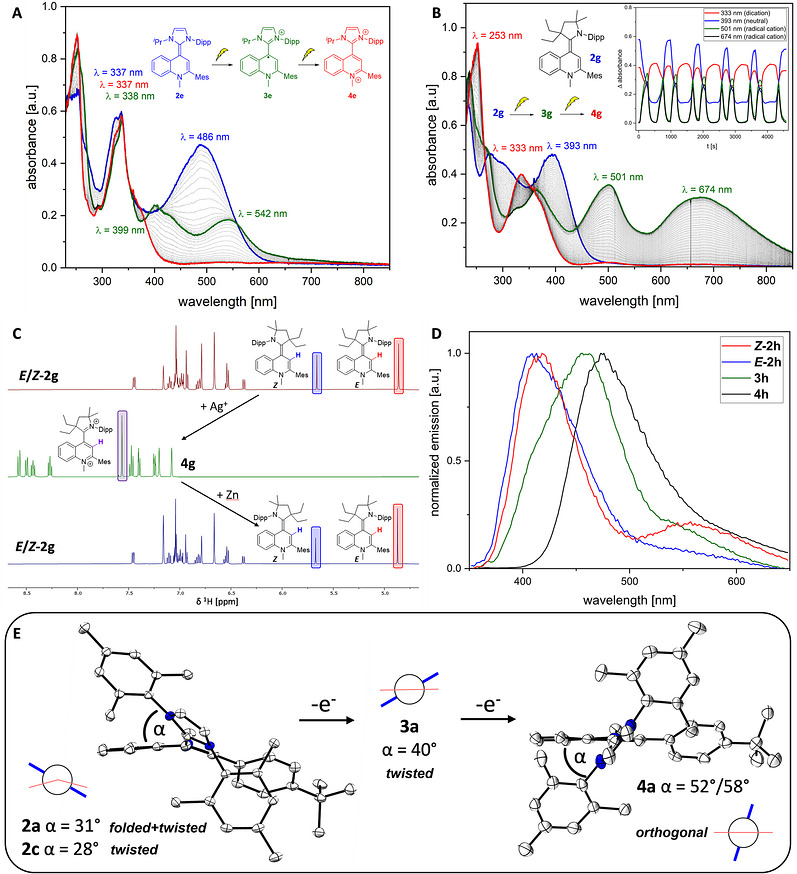
(A) UV–vis‐SEC of **2e** in thf and (B) UV–vis‐SEC of **2g** in thf. Color coding: neutral **2** (blue), radical cation **3** (green), dication **4** (red). Insert: Changes in absorption (difference spectrum) of **2g** in thf (0.1 M *n*‐Bu_4_NPF_6_) at room temperature. Controlled potential coulometry was used to switch between **2g**, **3g,** and **4g**. (C) ^1^H‐NMR spectra of top:**2g** in C_6_D_6_; middle: **4g** in CD_3_CN; bottom: **2g** in C_6_D_6_. (D) Normalized fluorescence spectra of ***E*‐2h**, ***Z*‐2h**, **3h,** and **4h** in thf. (E) x‐ray solid‐state structures of **2a** and **4a**. Thermal ellipsoids are shown at 50% probability. Hydrogen atoms are omitted for clarity. In case of dication, **4a** two counter‐anions (SbF_6_^−^) and two solvent molecules (thf) omitted for clarity. Torsion angle of **3a** calculated at the B3LYP/def2‐SVP(GD3‐BJ) level of theory.

In principle all new two‐stage redox systems **2a–2j** qualify as reversible redox‐switches. In fact, stoichiometric chemical oxidation of all compounds **2a–2** employing either one or two equivalents of AgSbF_6_ yielded the stable radical‐cations **3a–3i** and the dications **4a–4j** in moderate to excellent yields (35%–94%), evidenced by EPR or NMR spectroscopy, respectively (see the Supporting Information for synthetic details). Note, in contrast to other switches, the different oxidation states are cleanly isolable and stable compounds, including the open‐shell, radical cation oxidation state. In this manuscript, we focus on presenting the redox‐switching properties of two representative examples **2e** and **2g**, which share the same quinolinium fragment but feature two different carbene fragments (NHC and CAAC; for the remaining systems, see the Supporting Information). Spectro‐electrochemical measurements (SEC) of **2e** and **2g** showed a strong color change upon oxidation; the radical cations are typically bathochromic shifted compared to the neutral compounds [λ = 399/542 nm (**3e**); λ = 501/674 nm (**3g**)] resulting in a distinct dark brown or purple colour, while the dications are typically hypsochromic shifted [λ = 337 nm (**4e**); λ = 333 nm (**4g**)], resulting in yellow or colorless compounds (Figure 2A,B). The SEC measurements show excellent agreement with the UV–vis spectra of the isolated compounds (see Supporting Information). Given the limited use of electrical energy as a switching stimulus, in contrast to the widely used light, particularly for overcrowded alkenes, we explored the redox switchability of the systems in greater depth. SEC‐switching experiments were performed with **2g**, which showcased good stability over five back and forth cycles over all three redox states (Figure 2B; inset).

The switching can be visualized by NMR spectroscopy employing a chemical oxidant (AgSbF_6_) followed by a reductant (Zn) (Figure 2C). **2g** shows two distinct high‐field shifted ^1^H NMR signals for the dearomatized quinoline (C3)–H proton for the two *E*/*Z* isomers (*δ* = 4.87 ppm and 5.67 ppm; Figure 2C). Upon oxidation with two equivalents of Ag(I), the double bond is reduced to a single bond, permitting free rotation about this C–C axis and yielding a single downfield‐shifted ^1^H NMR resonance for the aromatic pyridinium proton (*δ* = 7.6 ppm). The respective dication **4g** can again be reduced back to the neutral hybrid **2g** by employing activated Zn‐powder. The isomeric ratio (*E*:*Z* = 65:35; derived by NOESY‐spectroscopy) after reduction is equivalent to the initial thermal equilibrium.

Next, we focused on the emissive properties of the switches. Interestingly, while the parent unsubstituted systems **2g**/**3g**/**4g** show no fluorescence, we noticed that phenyl substitution on the quinoline moiety has a marked influence on the emissive properties. The 8‐Ph compound (**2i**) exhibits moderate emission, while the 6‐Ph‐quinoline hybrid **2h** emits fluorescence from the neutral (**2h**), radical cationic (**3h**), and the dicationic (**4h**) oxidation states (Figure 2D). Despite the fluorescence signal for the neutral state **2h** being rather low, it is still different for the two *E*/*Z*‐isomers in both intensity and emission wavelength (for the clean preparation of the single isomers, see the photo switching paragraph below). The observation of luminescence from the open‐shell electronic state of radical cation **3h** highlights its potential as a candidate for organic functional materials, especially given the currently limited structural landscape of luminescent organic radicals [65]. Dication **4h** shows a strong light blue emission. Overall, the redox switch family can be utilized as electrochromic material, both in the absorption as well as emission properties.

Finally, we investigated the structural changes induced upon the redox switching process. Single crystals suitable for x‐ray diffraction could be obtained for the neutral (**2a**, **2c**, **2f–2j**), radical (**3g**), and dicationic (**4a**) compounds [66]. Interestingly, in contrast to regular switches in which the alkene substituents are in plane with the central C─C double bond, the neutral imidazole switches (**2a–2e**) feature a folded conformation with a twist of the carbene moiety in respective to the quinolinium plane (torsion‐angle ∢ ∼ 31 ° for **2a**; Figure 2E) while the N‐Mes groups are strongly pyramidalized out of plane avoiding donation of electron density into the already highly electron rich core (for the discussion on the structural distortions of the CAAC system, see below). DFT calculations showed that in the radical cations the IMes carbene moiety is additionally twisted out of plane (∢ 40° for **3a**), while x‐ray analysis of the dicationic oxidation state shows carbene and quinolinium planes to be tilted towards an orthogonal orientation (∢ 58° for **4a**) (Figure 2E). Overall, x‐ray analysis clearly confirms a large structural reorganization (defolding/rotation) from a twisted to orthogonal orientation upon switching through the three redox states, which goes in hand with an increase of the central C1─C2 bond length, from neutral (1.36–1.39 Å) over radical (1.44 Å) to dicationic (1.46–1.49 Å) compounds, which we attribute to a reduction of the formal C─C bond order from two to one.

### 2.2 Photo Switching and Electron Hole Catalysis

**FIGURE 3:**
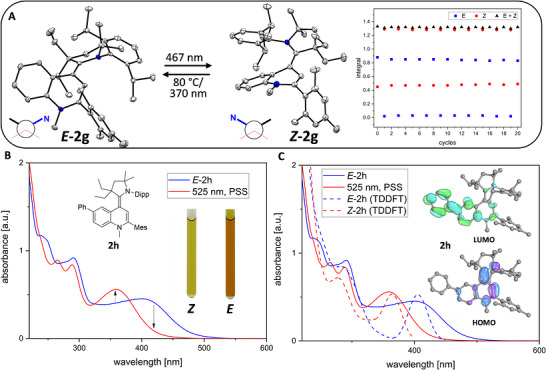
(A) X‐ray solid‐state structures of ***E*‐2g** and ***Z*‐2g**. Thermal ellipsoids are shown at 50% probability. Hydrogen atoms are omitted for clarity. Inset: Integrals obtained from ^1^H‐NMR measurements of **2g** during consecutive irradiation (*λ* = 467 nm, 15 min) and heating (80°C, 1h) cycles. (B) UV–vis spectra of **2h** in its thermodynamic equilibrium (blue; *E*:*Z* = 93:7) and after irradiation with 525 nm for 1 min (red; *E*:*Z* = 1:99, PSS) in thf. *Note*: A small amount of KHMDS (0.5–1 mg) was added prior to the experiment. Insert: Photos of ***E*‐2h** and ***Z*‐2h** in C_6_D_6_. (C) UV–vis spectra of **2h** in its thermodynamic equilibrium (blue; *E*:*Z* = 93:7) and after irradiation with 525 nm for 1 min (red; *E*:*Z* = 1:99, PSS) in thf compared with the calculated TD‐DFT spectra for the *E*‐ and *Z*‐isomer in thf. TD‐DFT spectra are corrected by 1.12 as a scalar factor according to Fehér et al. [68]. Insert: HOMO and LUMO of ***E*‐2h**. r2‐scan‐3c(SMD:THF) level of theory. ISO = 0.55. Hydrogen atoms were omitted for clarity.

Besides electrochemical switching we further investigated multi‐stimuli responsive properties including photo switching of selected unsymmetrical switches **2f–2j**. Unfortunately, in case of the acyclic ^Br^AAArC switch **2f** irradiation with 427 or 440 nm Kessil LED lamps, only led to decomposition of ***E*/*Z*‐2f** to an unknown mixture of substances with partially paramagnetic character, which might be due to the photosensitive nature of the Ar‐halide bond embedded in the carbene fragment. In order to avoid the photosensitive Ar‐halide substitution, we switched to the CAAC substituted systems **2g–2j**, which indeed proved to be photostable in contrast to **2f**. Importantly, in case of **2g** the initial *E*/*Z* isomer mixture (*E*/*Z* = 65:35) can be selectively and quantitatively isomerized with 467 nm LED irradiation to afford ***Z*‐2g** (*E*/*Z* < 1:99; Figure 3A). In the solid‐state, the thermodynamically more stable isomer ***E*‐2g** features a pyridine ring in a boat conformation, which causes the carbene moiety to fold backwards (Figure 3A). This folding/boat shape is significantly more pronounced compared to the NHC system, which was stronger tilted in its structure (see above). Furthermore, the benzannulated ring of the quinoline is bent out of the molecular plane, resulting in a butterfly‐type conformation. While the central C─C bond [1.374(1) Å] is in a range for typical double bonds (∼1.34 Å) [67], the overall folded geometry is far away from the traditional planarity of alkenes. Irradiation of the *E*/*Z* mixture of **2g** in solution followed by crystallization at −40°C in pentane allowed to obtain single crystals suitable for x‐ray diffraction for the metastable isomer ***Z*‐2g** (Figure 3). In ***Z*‐2g** the *N*‐Dipp fragment is positioned above the aryl moiety of the quinoline heterocycle, while the pyridine heterocycle is strongly distorted in a boat conformation leading to an overall folded structure.

The *E*/*Z* switching can be performed iteratively and occurs reversible and with excellent stability, with no decay of the system over 10 consecutive irradiating and heating cycles (Figure 3A inset). In case of **2g** no strong solvochromic effect of the neutral oxidation state was observed (see Figure S252). The irradiation with low energy light (467–525 nm; blue to green light) leads to a hypsochromic shift and thus the novel switches exhibit negative photochromism a sought‐after feature (Figure 3B) [52, 53, 54].

Back‐isomerization ***Z*‐2g**→ ***E*‐2g** to the thermal equilibrium was achieved partially by either irradiating with 370 nm (*E*:*Z* = 22:78) or by heating at 80°C for 1 h (*E*/*Z* = 65:35) showing a bathochromic shift. After establishing **2g** as the first *E*/*Z*‐photoswitchable hybrid system, we aimed to expand the scope and enhance the switching efficiency of this class through theory‐guided design. TD‐DFT (r2‐scan‐3c(SMD:THF)//CAM‐B3LYP/def2‐TZVPP‐def2/J(CPCM:THF)) calculations allowed to accurately predict the absorption and transitions of **2g** in both isomeric states (see Supporting Information). Encouraged by the excellent reliability of the in‐silico studies we predicted the UV–vis properties based on different substitution patterns. Interestingly, the introduction of phenyl‐substituents not only leads to a bathochromic shift for both isomers but also enlarges the difference between *λ*_max_ for the two *E*/*Z*‐isomers. To verify these findings, we isolated the most promising candidates (**2g–2j**) and analysed their switching behaviour (Table 1). In contrast to more elaborate photochemical switches, the introduction of various substituents can be achieved from commercially available starting materials [69] due to the highly modular approach. In accordance with the calculations, the enlargement of the π‐system leads to a bathochromic shift: while the unsubstituted system **2g** is yellow, the 6‐Ph‐ and 8‐Ph‐quinoline based systems **2h** and **2i** are orange and the introduction of an even more extended 2‐styrene group (**2j**) leads to a red coloured compound.

**TABLE 1:** Summary of photophysical properties of quinolinium/CAAC photo‐redox switches. PSS: Photo stationary state.

Compound	λ_max_ (*E*) (nm)	λ_max_ (*Z*) (nm)	Δλ_E‐Z_ (nm)	*E*:*Z* (thermal equilibrium)	PSS *E* → *Z* (λ_exc_ [nm])	*t*_1/2_ (25°C)	*t*_1/2_ (+KHMDS)
**2g**	392	378	14	65:35	1:99 (467)	2.3 h	7 d
**2h**	400	359	41	93:7	1:99 (525)	—	2.1 d
**2i**	407	386	21	75:25	2:98 (467)	26 min	—
**2j**	379	376	3	100:0	39:61 (427)	< 5 min	21.7 h

The best performing switch **2h** shows a Δ*λ*_max_(*E*/*Z*) of 41 nm. Thus, the photo‐switching leads to a visible color change from dark orange to light yellow (Figure 3B). Furthermore, the photo stationary state (PSS) for the switches is in most cases nearly quantitively the metastable *Z*‐isomer. The half‐lifes range from below 5 min to multiple hours at ambient temperature. To our surprise, the half‐lifes of the metastable *Z*‐isomers can be drastically extended up to the time scale of one week by the addition of potassium bis(trimethylsilyl)amide (KHMDS) which triggered our interest for looking into detail in the photochemical switching process.

**FIGURE 4:**
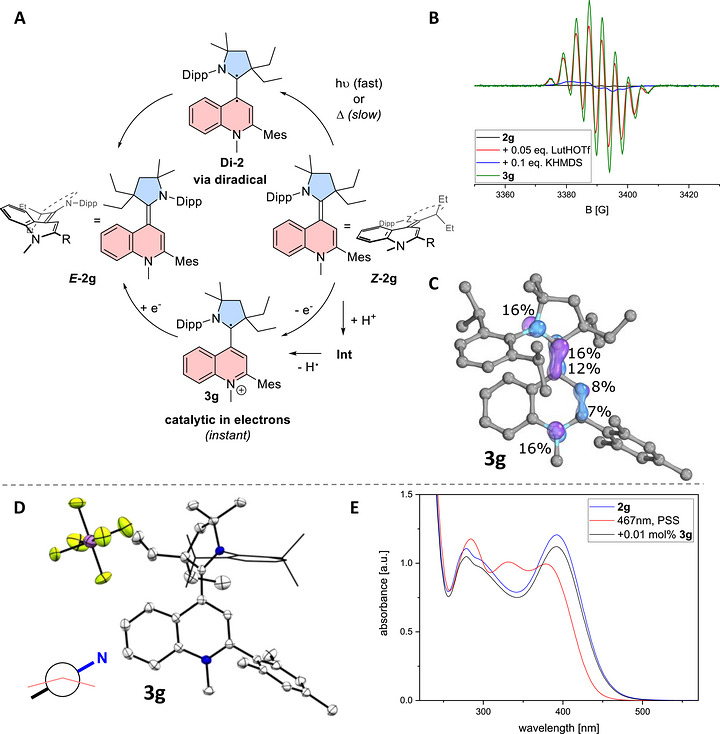
(A) Schematic overview of the (proton induced) electron hole catalysis mechanism. (B) EPR‐spectra of **2g**, after LutHOTf addition and after KHMDS addition in C_6_D_6_ and of **3g** in thf. (C) SOMO of **3g** with the significant Mulliken spin‐densities (in %). Isovalues: 0.30. Hydrogen atoms were omitted for clarity. (D) X‐ray solid‐state structure of **3g**. Thermal ellipsoids are shown at 50% probability. Hydrogen atoms are omitted for clarity; Dipp‐group is depicted as wireframe. (E) UV–vis spectra of hybrid **2g** in its thermodynamic equilibrium, after irradiation with 467 nm for 30 min (PSS) and then after the addition of 0.01 mol% **3g** to the metastable *Z‐*isomer in thf.

Initially, we expected that a proton catalyzes the back‐switching via addition onto the central double bond leading to a freely rotatable C─C single bond. Note, this intermediate is identical to the reaction intermediate **Int** formed by addition of the carbene to the cationic heterocycle (Scheme 2). To verify this hypothesis, we performed an NMR experiment with the metastable isomer ***Z*‐2g** and 0.05 equivalents of lutidinium triflate as a weak acid. Interestingly, immediate back‐switching to the thermodynamic equilibrium and broadened ^1^H‐NMR signals were observed (see Figure S198). The consecutive addition of 0.1 equivalents of KHMDS restored sharp signals, and isomerization by irradiation became possible again. This finding supports a proton‐catalyzed isomerization mechanism (Figure 4A). However, we also performed in parallel in situ EPR experiments: Upon addition of lutidinium triflate to **2g** the radical cation **3g** formed, which matched perfectly with the independently prepared radical **3g** by stoichiometric oxidation of **2g** (Figure 4B). In fact, we are able to obtain x‐ray data for the stable radical cation **3g** (Figure 4D). In **3g** the central C─C bond [1.440(3) Å] is significantly elongated compared to the neutral alkenes [1.374(1) Å (***E*‐2g**) and 1.371(2) Å (***Z*‐2g**)], indicating free C─C bond rotation in the radical oxidation state. The formal carbene entity is tilted by ca. 45° out of the quinolinium plane, while the quinolinium moiety remains slightly boat shape deformed. In the EPR spectrum, the distinct hyperfine‐couplings were simulated and match with the results obtained by computations (see Supporting Information). The SOMO is localized mainly on the quinoline moiety while the degree of spin delocalization onto the carbene moiety is dependent on the π‐acceptor properties of the carbene (14%–17% for IMes hybrids **3a–3d**; 39%–43% for CAAC hybrids **3g–3i** (Figure 4C and SI). Importantly, addition of KHMDS leads to a strong decrease in the EPR signal intensity indicating reduction of **3** to **2g** (Figures 4B and S200). These observations indicate a mechanism in which an electron can act as electron hole catalyst for the *E*/*Z* isomerization process. Once the radical cation **3g** is generated, rapid C─C bond rotation is feasible (for computational evidence, see below), which is followed by electron injection to form the thermodynamic *E*/*Z* ratio of **2g**, rendering the isomerization process catalytic in electrons (Figure 4A).

In order to verify this assumption, UV–vis absorption experiments were performed with the isolated electron hole **3g** as the initiator (Figure 4E). Indeed, ***Z*‐2g** is switched back to ***E*‐2g** instantaneously upon addition of as little as 0.01 mol% of **3g**. Upon further reduction of the concentration of **3g**, the back‐switching process becomes slower, requiring up to 8 min to re‐establish thermodynamic equilibrium after the addition of 0.001 mol% of **3g** (see Supporting Information). It should be emphasized that such electron hole mechanism was described for diazo switches by Hecht et al. [18]; however, in the here described system, we are able to cleanly isolate and structurally characterize the electron hole catalyst and to observe it spectroscopically. Previously, the electron hole was generated either by in situ oxidation using tailor‐made oxidants or by electrochemical methods requiring specialized instrumentation [18, 70, 71]. While the isomerization by light and the back‐switching either by light or temperature are time dependent, the back‐switching mechanism via an electron hole is instantaneous entailing various advantages. KHMDS has a twofold mechanism to elongate the half‐life of the metastable state: On the one hand, trace amount of radical cation can be reduced inhibiting the electron hole catalyzed back‐switching mechanism, and on the other hand, it can quench any free protons to generate radical cations inhibiting the back‐switching.

### 2.3 Computational Analysis of the Switching Mechanism

To gain detailed insight into the photochemical switching processes and the electron‐hole catalysis mechanism, quantum chemical calculations were conducted for **2g** as a model system. Excited states calculations of **2g** have reproduced the absorption spectra in the visible range (Figure 3C). Analysis of the excited states revealed that the lowest excited state is bright (highest oscillator strength), responsible for the main absorption band centered at 393 nm. The electron density difference and the molecular orbitals (Figure S280) indicate that S_0_→S_1_ excitation is changing the electron density on the central double bond, effectively weaking this bond. To study the subsequent processes following the excitation, we have performed nonadiabatic molecular dynamics (NAMD) simulations starting from S_1_. In total, 60 trajectories were initiated with different initial conditions sampled from an ab initio molecular dynamics simulation in the ground state. In 32 of the 60 trajectories, a successful *E* → *Z* isomerization is observed. In all 60 trajectories, the central C─C bond rotated in the counterclockwise direction, therefore achieving unidirectional rotation (Figure S281). Note, even though the motion is unidirectional, it is overall not a rotor but more like a “windshield wiper” movement. Structural analysis indicated that this is caused by the repulsion between the quinoline moiety and the geminal ethyl groups of the ^Et^CAAC unit.

**FIGURE 5:**
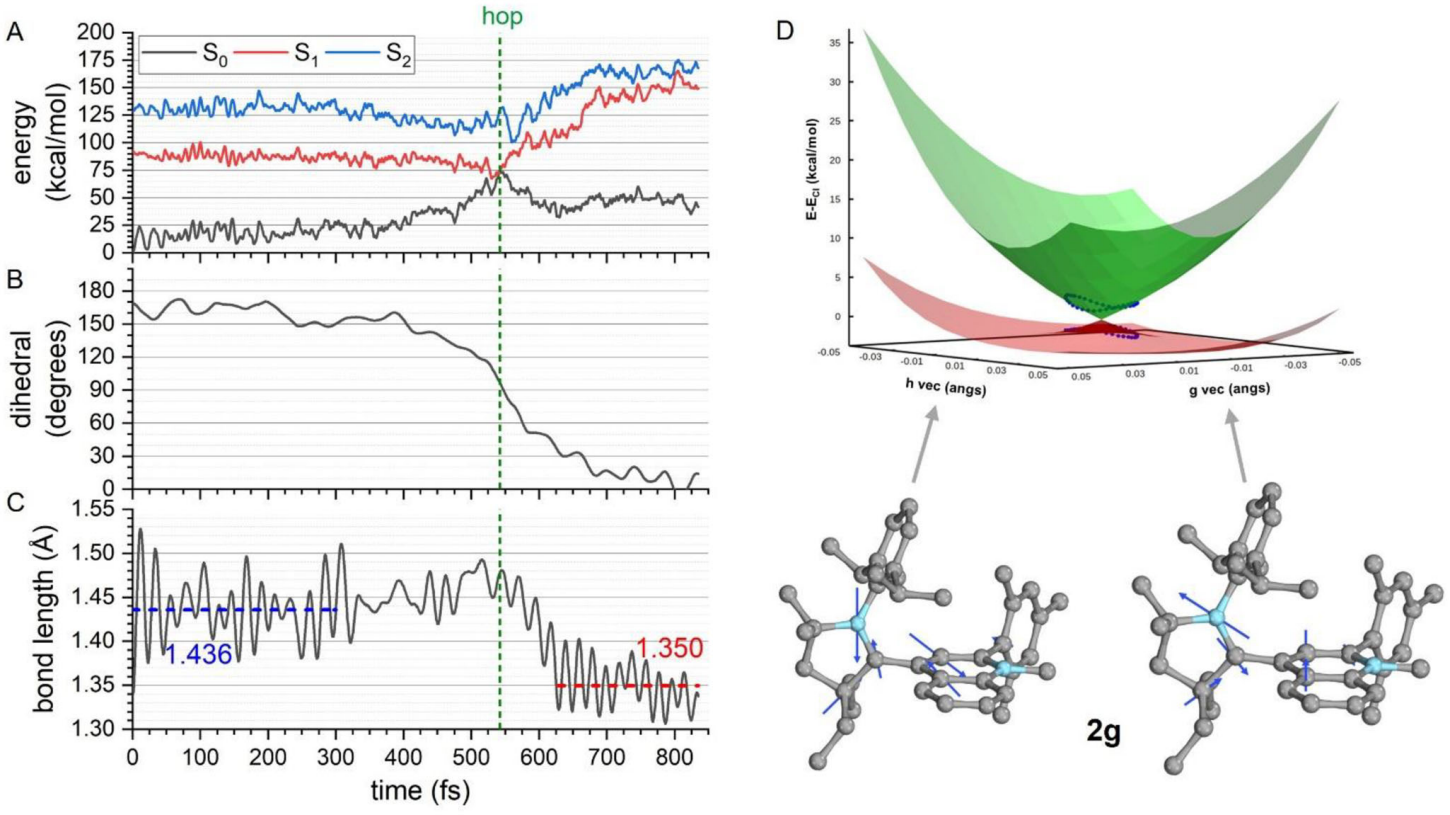
Computational study of the photoisomerization of **2g**. A prototypical nonadiabatic molecular dynamics trajectory is analyzed in detail. (A) Evolution of the S_0_, S_1_, and S_2_ potential energies as a function of time. The transition from S_1_ to S_0_ is indicated by the dotted green line at the time 544 fs into the simulation. (B) Evolution of the dihedral of the central double bond. (C) Evolution of the central double bond length. (D) Conical Intersection including the branching plane vectors g and h.

We have selected one trajectory as an example to describe the photoisomerization mechanism (Figure 5A–C). Upon vertical excitation, there is a stretch of the central bond in the excited state as indicated by the electron density difference map (see Supporting Information). The bond length oscillates around 1.43 Å in the excited state (Figure 5C). This elongation is associated with a rotation of the double bond, which starts from 170 degrees (Figure 5B). Following the relaxation in the excited state, the selected trajectory shows a hop from S_1_ to S_0_ at 544 fs. At this time, the dihedral angle has a value of 100 degrees and proceeds towards the *Z* conformation. After the relaxation to the ground state, the double bond length is oscillating around 1.35 Å.

Such an ultrafast photoisomerization is mediated by a conical intersection (CI), which establishes an effective funnel from the S_1_ to S_0_, enabling a nonadiabatic decay. Further characterization was done by optimizing the CI. Figure 5D shows the minimum energy conical intersection obtained using the hole–hole Tamm–Dancoff‐approximated density functional theory (hh‐TDA‐DFT) [72], the same level of theory as the nonadiabatic molecular dynamics trajectories. The two branching plane vectors that correspond to the nonadiabatic coupling and the gradient difference are depicted in Figure 5D. The former shows that rotation of the central double bond will lift the degeneracy between the ground and excited state, while the latter shows a coordinate involving the change of the bond stretching coordinates. These are two typical coordinates involved in the photoisomerization that has been previously found in other organic chromophores, for example, the retinal protonated Schiff base in rhodopsin [73].

**FIGURE 6:**
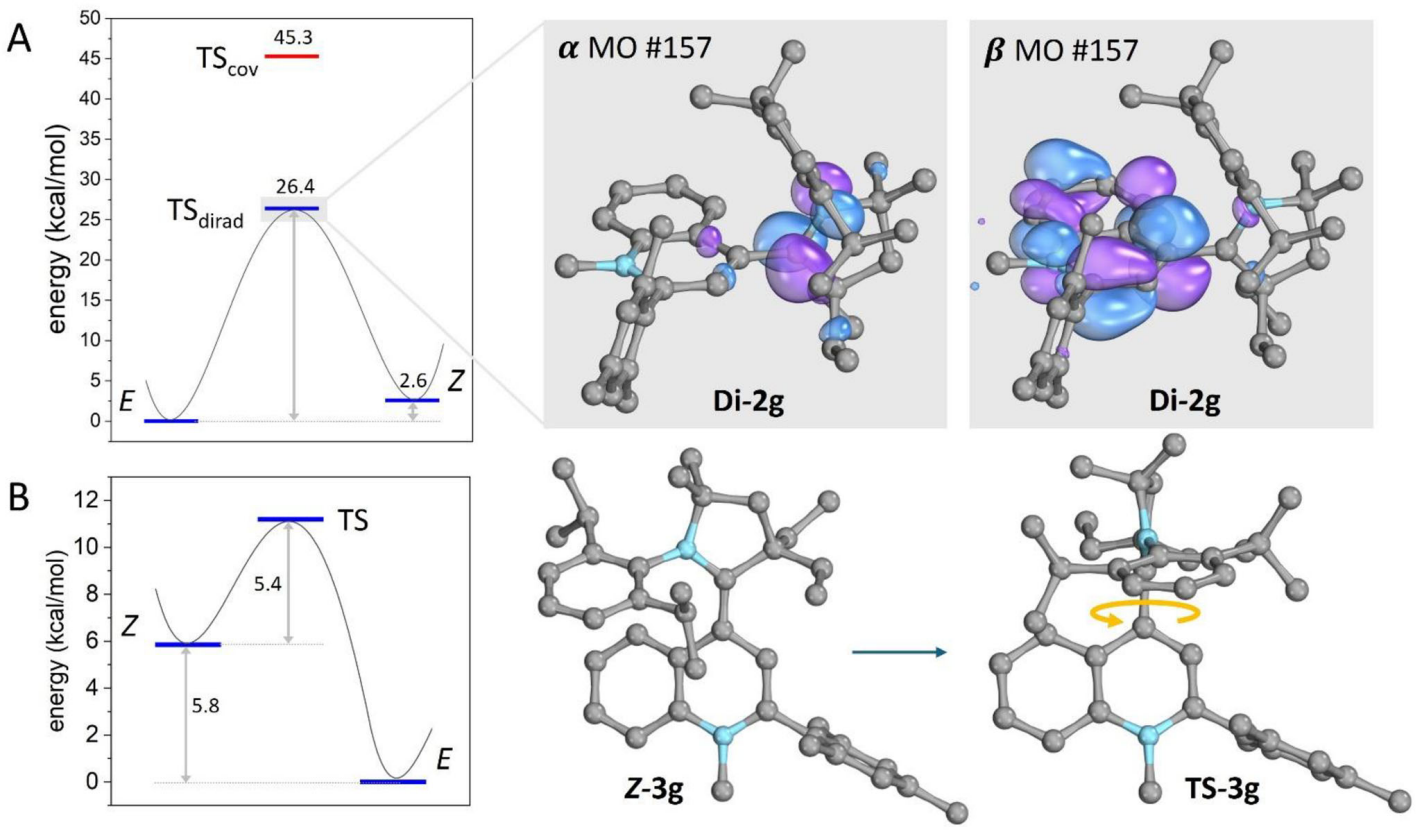
Computational study of the thermal isomerization. (A) comparison of the transition state energies obtained from a restricted closed shell and unrestricted open shell DFT calculation. The singly occupied frontier orbitals are shown as in inset. (B) Thermal isomerization of the radical cation.

Starting from the *Z* isomer we probed the thermal isomerization by characterizing the transition state and computing the activation barrier (Figure 6A). At the CAM‐B3LYP/def2‐SVP level of theory, the barrier is at 45.3 kcal mol^−1^ for the closed shell case. However, the use of broken symmetry (BS) leads to a lower energy at 26.4 kcal mol^−1^, that suggests the presence of effectively unpaired electrons indicative of a diradical mechanism. This is further corroborated by depicting the frontier orbitals of opposite spin from the BS‐DFT calculation (Figure 6). While one unpaired electron is located on the quinoline portion of the switch, the other electron is located on the fragment stemming from the carbene reflected in Lewis structure **Di‐2** (Figure 4A). It is interesting to note that carbene entities are well‐known to stabilize radicals and even diradicals [74, 75], which applied to this new area of photo‐switches, allows to fine tune the thermal isomerization barrier. The height of the barrier in the ground state is consistent with the very slow conversion measured experimentally. However, in the oxidized state, the barrier is significantly lowered to 5.4 kcal mol^−1^ starting from the *Z* isomer, consistent with the experimental finding from the electron hole catalysis (Figure 6B). Analysis of the electronic structure shows a single occupied molecular orbital localized on the central double bond. This explains the larger twist of the double bond discussed above as well as the lower barrier associated with the breaking of the double bond. The barrier for the reverse isomerization is 11.2 kcal mol^−1^ and therefore nearly doubled compared to the *Z* → *E* isomerization. The oxidized *E* isomer is twisted by 30 degrees out of plane. Further rotation toward the planar structure is associated with a barrier of 19.3 kcal mol^−1^ due to the repulsion between the quinoline moiety and the geminal ethyl groups of the ^Et^CAAC unit.

## 3 Conclusion

In conclusion, we report the highly modular design and the characterization of a class of multi‐stimuli‐responsive photo‐ and redox‐switches. Imidazole‐based systems do qualify as efficient redox‐switches, however, due to their strongly reducing redox‐potentials and high bond polarization do not allow photo switching. More π‐accepting carbenes, in particular CAACs, allow both redox‐ and photo‐switching processes with excellent stability in photo‐switching experiments and high stability during redox‐cycling. Additionally, all redox‐states of the switches are isolable and have been fully characterized including EPR, NMR, and in selected cases also x‐ray diffraction. Furthermore, selected compounds display multiple switching outputs in each oxidation state, including distinct color changes, geometric rearrangements, and fluorescence emission. By using a TD‐DFT guided design, we were able to synthesize a small family of novel switches entailing high PSS and tuneable t_1/2_. Additionally, an electron hole catalyzed back‐switching mechanism was described in which it was possible to isolate the electron hole and analyze it spectroscopically. This rapid hole catalysis switching mechanism can be efficiently turned off by addition of KHMDS and turned on by addition of the isolated stable radical cation. Importantly, we also give in depth computational insight into the photochemical switching mechanism. Nonadiabatic molecular dynamics simulations show a barrierless, ultrafast decay from the first excited state to the ground state via a conical intersection. This involves the bond stretching coordinate and the rotation of the central double bond. The computations for the thermal isomerization finds a much lower barrier for the radical cation compared to the neutral species. Since the two entities, carbene and quinolinium salts, are easily tuneable and assembled in a single step, several other improved switches are imaginable based on this design strategy. In particular, we hope that our work paves the way for (photo‐) redox controlled rotors [76, 77, 78], which are under investigation. By introducing chirality, at either the carbene or the heterocycle moiety [79], it should be feasible to introduce unidirectional motion.

## Author Contributions

**Chris Burdenski**: investigation, methodology, formal analysis, visualization, writing – original draft. **Patrick W. Antoni**: investigation, data curation, methodology. **Marcel E. Baumert**: investigation, methodology, data curation. **Leonie Ziemann**: investigation, formal analysis. **Samaresh C. Sau**: investigation, formal analysis. **Julian J. Holstein**: data curation, formal analysis. **Maria Castro**: investigation. **Nitin Kumar**: investigation. **Ofer Filiba**: investigation. **Igor Schapiro**: writing – review and editing, writing – original draft, funding acquisition, supervision, resources. **Max M. Hansmann**: writing – review and editing, writing – original draft, conceptualization, funding acquisition, project administration, supervision, resources.

## Conflicts of Interest

The authors declare no conflicts of interest

## Supporting information




**Supporting File 1**: anie72720‐sup‐0001‐SuppMat.pdf.

**Supporting File 2**: anie72720‐sup‐0002‐DataFiles.zip.

## Data Availability

The data that supports the findings of this study are available in the Supporting Information of this article
