# Supplementary material for: Overcrowded Alkene Photo‐Redox‐Switches Based on Quinolinium/Carbene Building Blocks
Source: Angew Chem Int Ed Engl. 2026 May 21;65(29):e9975231. doi: 10.1002/anie.9975231 (PMC13360384; doi:10.1002/anie.9975231)
Supplement: Supplementary file 1 — Supporting File 1: anie72720‐sup‐0001‐SuppMat.pdf. [file ANIE-65-e9975231-s002.pdf]

Supporting Information

Overcrowded Alkene Photo-Redox-Switches based on Quinolinium/Carbene Building Blocks

Chris Burdenski, Patrick W. Antoni, Marcel E. Baumert, Leonie Ziemann, Samaresh C. Sau, Julian J. Holstein, Maria Castro, Nitin Kumar, Ofer Filiba, Igor Schapiro*, Max M. Hansmann*

[a] C. Burdenski, Dr. P. W. Antoni, M. E. Baumert, L. Ziemann, Dr. S. C. Sau, Dr. J. J. Holstein, Prof. Dr. M. M. Hansmann
Fakultät für Chemie und Chemische Biologie
Technische Universität Dortmund
Otto-Hahn-Str. 6, 44227 Dortmund, Germany
E-mail: max.hansmann@tu-dortmund.de

[b] Dr. M. Castro, Dr. N. Kumar, O. Filiba, Prof. Dr. I. Schapiro
Fakultät für Physik
Technische Universität Dortmund
Otto-Hahn-Str. 4, 44227 Dortmund, Germany
E-mail: igor.shapiro@tu-dortmund.de

[c] Prof. Dr. I. Schapiro
Institute of Chemistry, The Hebrew University of, Jerusalem, 9190401, Jerusalem, Israel

[d] Prof. Dr. I. Schapiro
Research Center Chemical Sciences and Sustainability, University Alliance Ruhr, 44801, Bochum, Germany

Table of Contents

Materials and Methods 3

Synthetic Procedures 4

NMR Spectra 36

EPR Spectroscopy 110

X-ray Characterization 120

Isomerization and Switching Experiments 134

- Setup 134
- Wavelength dependency 134
- Control experiments 137
- Half-life of the metastable *Z*-Isomer 137
- Light driven forth- and back-switching 141
- UV-vis switching studies 142
- Investigation of electron hole catalysis 148
- Stability measurements 155

Electrochemical Measurements 157

UV-vis Spectroscopy 171

UV-vis Spectroelectrochemistry 187

Fluorescence spectroscopy 193

Computational Data 198

- Geometry optimization 198
  - For EPR calculation of Radical Cations 198
  - For TD-DFT calculations of Photoswitches 210
  - Transition state and E/Z isomer optimization 226
- TDDFT Calculations 227
  - Electron density difference 244
  - Nonadiabatic molecular dynamics simulations 244

References 246

Materials and Methods

All solvents were purified with a MBraun SPS – 800 and were stored over molecular sieves and degassed with argon. C_6D_6 and d_8-thf were distilled over Na and degassed. Reactions were carried out either under N_2 or Ar atmosphere. Solids were handled and NMR samples were prepared in a nitrogen filled glovebox. High resolution MS (ESI): Finnigan MAT 95, accurate mass determinations: Bruker APEX III FT-MS (7 T magnet) and LTQ-Orbitrap-XL (Thermo Scientific) equipped with a heated electrospray ionization source (HESI). NMR spectra were measured on the spectrometers Bruker AV 500 Avance NEO and AV 600 Avance III HD and chemical shifts (δ) are referenced to their solvent signals [C_6D_6, 7.16 (^{1}H NMR) 128.06 (^{13}C NMR); CD_3CN, 1.94 (^{1}H NMR) 118.26 (^{13}C NMR); $CDCl_3$, 7.26 (^{1}H NMR) 77.16 (^{13}C NMR), coupling constants (*J*) in Hz. All spectra were recorded in 5 mm NMR tubes at the temperatures indicated. The solvent signals were used as references and the chemical shifts converted to the TMS scale. Flash chromatography was performed with Merck 60 silica gel (40-63 µm). Thin-layer chromatography (TLC) analysis was performed using Merck silica gel 60 F254 TLC plates and visualized by UV irradiation and/or ceric ammonium molybdate, $KMnO_4$ or *p*-anisaldehyde. All commercially available compounds (Acros, abcr, Alfa Aesar, BLD pharm, Carbolution, ChemPur, Fluorochem, Sigma Aldrich, TCI) were used as received. IR-ATR measurements (diamond) were performed in reflection mode on a Bruker Alpha II inside a glovebox, wavenumbers in cm^{-1}. Melting points were measured with a Büchi M-560 apparatus.

Free ^{Et}**CAAC** was synthesized by deprotonation of the imidazolium-BF_4 salt as described in the literature.[1]

For NMR-characterization of the switches only the main isomer is described if not otherwise stated.

Synthetic Procedures

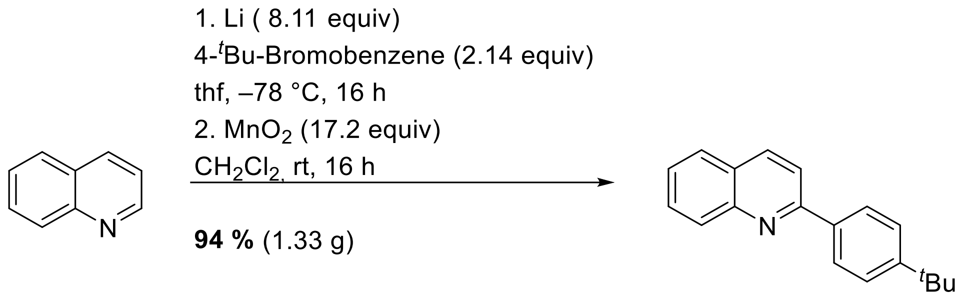

2-*p*-^{t}Bu-Ph-quinoline **S1:** Elemental lithium stored in paraffin oil (Li, 6.94 g/mol, 305.03 mg, 43.95 mmol, 8.11 equiv) was washed with pentane (10 mL), activated by addition of methanol (5 mL) for 10 seconds, before it was washed with pentane (2 x 10 mL) again and dried in vacuo. The activated lithium was suspended in Et_2O (10 mL). Under cooling to 0 °C and vigorous stirring, 4-^{t}Bu-bromobenzene ($C_{10}H_{13}Br$, 213.12 g/mol, 2.47 g, 2.14 equiv) was added to the suspension. The mixture was stirred for 30 min at 0 °C and for further 4 h at ambient temperature. The mixture was added directly transferred into a cooled (-78 °C) solution of quinoline (C_9H_7N, 129.16 g/mol, 700 mg, 1.09 g/mL, 642.2 µL, 5.42 mmol, 1.0 equiv) in Et_2O (10 mL) with a filter cannula. The reaction mixture was left to slowly warm up to ambient temperature and was stirred for 16 h. The mixture was poured slowly over ice and was extracted with Et_2O (3 x 100 mL) three times. The combined organic layers were dried over Na_2SO_4 and all volatiles were removed under reduced pressure. The crude product was dissolved in CH_2Cl_2 (250 ml), activated MnO_2 (MnO_2, 86.94 g/mol, 8.1 g, 93.22 mmol, 17.2 equiv) was added and the mixture was left to stir for 16 h. Filtration over Celite and removal of all volatiles under reduced pressure yielded 2-*p*-^{t}Bu-Ph-quinoline **S1** ($C_{19}H_{19}N$, 261.37 g/mol, 1.33 g, 5.09 mmol, 94 %) as an orange solid. The spectroscopic data are in good agreement with previous reports.[2]

^{1}H NMR (400 MHz, $CDCl_3$, 298K): δ [ppm] = 8.20 (t, *J* = 8.7 Hz, 2H), 8.10 (d, *J* = 8.5 Hz, 2H), 7.87 (d, *J* = 8.7 Hz, 1H), 7.83 (d, *J* = 8.1 Hz, 1H), 7.72 (t, *J* = 7.0 Hz, 1H), 7.60 – 7.48 (m, 3H), 1.39 (s, 9H); **^{13}C NMR** (101 MHz, $CDCl_3$, 298K): δ [ppm] = 157.5, 152.8, 147.0, 136.9, 129.8, 127.6, 127.5, 127.2, 126.3, 125.8, 125.6, 125.3, 119.6, 34.9, 31.4.

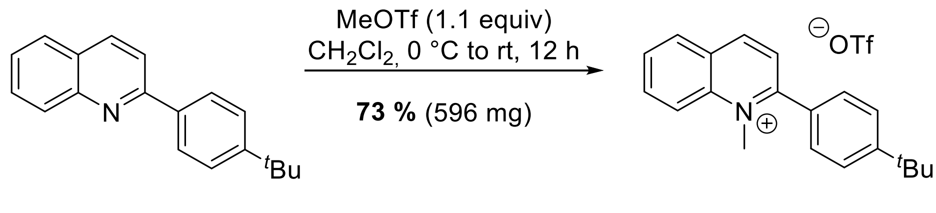

Quinolinium salt **1a:** 2-*p*-^{t}Bu-quinoline **S1** ($C_{19}H_{19}N$, 261.37 g/mol, 500 mg, 1.91 mmol, 1.0 equiv.) was dissolved in CH_2Cl_2 (10mL) and cooled to 0°C. Then MeOTf ($C_2H_3F_3O_3S$, 164.1 g/mol, 345.31 mg, 1.5 g/mL, 230.21 µL, 2.1 mmol, 1.1 equiv) was added dropwise. The solution was stirred for 5 min at 0 °C, warmed up to room temperature and stirred for 12 h. Then the solvent was evaporated until 1-2 mL of the solvent were left over. Then Et_2O (30 mL) was added resulting in the formation of a white solid. The solvent was removed using a filter cannula and the residue solid was removed with Et_2O (3 x 10 mL). Drying of the solid

under reduced pressure yielded quinolinium salt **1a** ($C_{21}H_{22}F_3NO_3S$, 425.47 g/mol, 596.0 mg, 1.40 mmol, 73 %) as a colourless solid.

m.p. 173°C; **^{1}H-NMR** (300 MHz, CD_3CN, 298K): δ [ppm] = 9.03 (d, *J* = 8.5 Hz, 1H), 8.42 (d, *J* = 9.1 Hz, 1H), 8.38 (d, *J* = 8.2 Hz, 1H), 8.27 (t, *J* = 8.7 Hz, 1H), 8.03 (t, *J* = 7.6 Hz, 1H), 7.97 (d, *J* = 8.7 Hz, 1H), 7.76 (d, *J* = 8.2 Hz, 2H), 7.62 (d, *J* = 8.7 Hz, 2H), 4.36 (s, 3H), 1.41 (s, 9H); **^{13}C-NMR** (101 MHz, CD_3CN, 298K): δ [ppm] = 161.3, 156.6, 147.0, 140.9, 137.0, 131.5, 131.3, 130.9, 130.4, 129.7, 127.4, 126.3, 120.3, 43.5, 35.9, 31.3; **IR** [cm^{-1}]: $\tilde{\nu}$ = 3064, 3033, 2961, 2915, 2870, 1605, 1584, 1509, 1464, 1441, 1368, 1263, 1224, 1146, 1074, 1031, 1011, 876, 860, 830, 778, 754, 633, 597, 566, 516, 484, 463, 452, 439, 428, 409; **HR-MS-ESI(+)** calc. for $C_{20}H_{22}N^+$: 276.1747 $[M]^+$; found 276.1749.

Synthesis of neutral **2a**

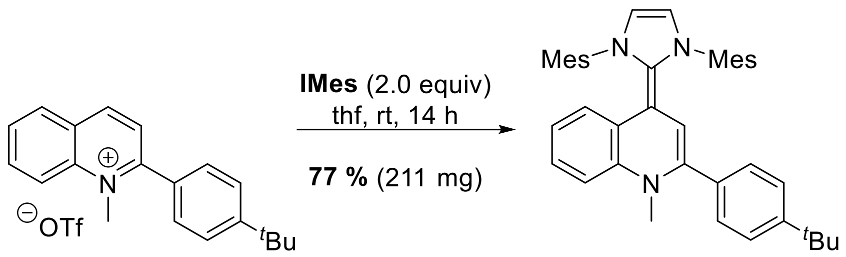

A suspension of *N*-methyl-2-*p*-^{t}Bu-Ph-quinolinium salt **1a** ($C_{21}H_{22}F_3NO_3S$, 425.47 g/mol, 200 mg, 0.471 mmol, 1.0 equiv) in thf (20mL) was slowly added to a colorless solution of **IMes** ($C_{21}H_{24}N_2$, 304.44 g/mol, 286.21 mg, 0.940 mmol, 2.0 equiv) in thf (20 mL) and stirred for 14 h. The solution turned intense dark red. The solvent was removed under reduced pressure. Then Et_2O (2x 20 mL) was added and the suspension was filtrated using a filter cannula and the residue solid was extracted with Et_2O. The combined organic extracts were dried under reduced pressure, which gave hybrid system **2a** ($C_{41}H_{45}N_3$, 579.83 g/mol, 211 mg, 363.9 µmol, 77 %) as a red solid.

Single crystals suitable for X-ray diffraction could be obtained from a saturated Et_2O solution at –40 °C.

m.p. 131 °C; **^{1}H NMR** (400 MHz, C_6D_6, 298K): δ [ppm] = 7.22 (d, *J* = 8.5 Hz, 2H), 7.13 (d, *J* = 8.5 Hz, 2H), 6.74 (s, 2H), 6.67 – 6.58 (m, 3H), 6.52 (dd, *J* = 7.6, 1.5 Hz, 1H), 6.33 (t, *J* = 7.6 Hz, 2H), 5.77 (s, 1H), 5.71 (s, 1H), 5.25 (s, 1H), 2.78 (s, 3H), 2.39 (s, 6H), 2.34 (s, 6H), 2.02 (s, 6H), 1.21 (s, 9H); **^{13}C NMR** (126 MHz, C_6D_6, 298K): δ [ppm] = 148.3, 146.5, 140.3, 137.6, 137.4, 136.6, 136.3, 135.6, 134.5, 130.7, 129.9, 129.6, 128.4, 126.7, 125.1, 122.9, 122.3, 119.7, 117.2, 113.9, 111.1, 83.4, 65.9, 39.4, 34.4, 31.5, 20.9, 19.1, 18.5, 7.2, 2.7; **IR** [cm^{-1}]: $\tilde{\nu}$ = 2949, 2915, 2859, 1588, 1538, 1504, 1475, 1403, 1359, 1328, 1280, 1264, 1201, 1161, 1126, 1078, 1030, 1009, 916, 842, 816, 736, 695, 681, 637, 613, 572, 544, 517, 486, 471, 460, 436, 420, 406; **HR-MS-ESI(+)** calc. for $C_{41}H_{46}N_3$: 580.3687 $[M+H]^+$; found 580.3678; **UV-vis:** λ_{max}: 474nm (ε = 24961 cm^{-1} M^{-1}), 384 nm (ε = 22924 cm^{-1} M^{-1}).

Synthesis of radical cation **3a**

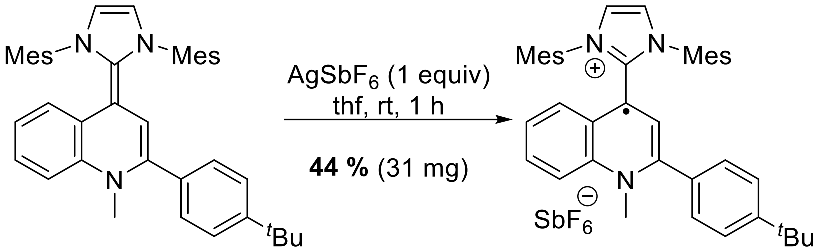

$AgSbF_6$ ($AgSbF_6$, 343.62 g/mol, 29.63 mg, 96.23 µmol, 1.0 equiv) was dissolved in thf (5 mL) and added dropwise to a solution of switch **2a** ($C_{41}H_{45}N_3$, 579.83 g/mol, 50 mg, 86.23 µmol, 1.0 equiv) in thf (5 mL). The now dark solution was stirred for 1 h at room temperature. After that the solution was filtrated over a celite plug (1.5 cm). Then the solvent was evaporated to give radical cation **3a** ($C_{41}H_{45}F_6N_3Sb\cdot$, 815.58 g/mol, 31 mg, 38.01 µmol, 44 %) as a red solid.

m.p. 121 °C; **EPR:** g = 2.0031 (1xN 13.29; 2xN 6.0485; 1xH 4.246; 1xH 4.1803; 3xH 12.0721); **IR** [cm^{-1}]: $\tilde{v}$ = 3150, 2961, 2868, 1738, 1605, 1557, 1478, 1380, 1364, 1261, 1230, 1171, 1110, 1084, 1062, 1031, 929, 888, 852, 802, 752, 651, 573; **HR-MS-ESI(+)** calc. for $C_{41}H_{46}N_3$: 580.3687 $[M+H]^+$; found 580.3661; **UV-vis:** 370 nm (ε = 9734 cm^{-1} M^{-1}), 557 nm (ε = 2766 cm^{-1} M^{-1}).

Synthesis of dication **4a**

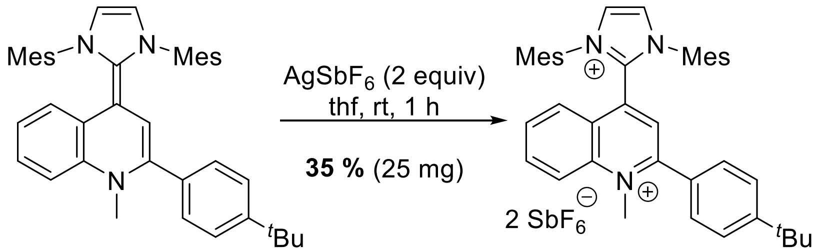

$AgSbF_6$ ($AgSbF_6$, 343.62 g/mol, 47.41 mg, 137.97 µmol, 2.0 equiv) was dissolved in thf (5 mL) and added dropwise to a solution of hybrid system **2a** ($C_{41}H_{45}N_3$, 579.83 g/mol, 40.0 mg, 68.99 µmol, 1.0 equiv) in thf (5 mL). The solution turned dark and was stirred for 1 h. During that time the solution changed to a colourless solution. Afterwards the solution was filtrated over a celite plug (1.5 cm). Then the solvent was evaporated under reduced pressure to give dication **4a** ($C_{41}H_{45}F_{12}N_3Sb_2$, 1051.34 g/mol, 25.0 mg, 34.5 µmol, 35 %) as a colourless powder.

Single crystals suitable for X-ray diffraction could be obtained from a saturated Et_2O solution at –40 °C.

m.p. 181°C (decomp.); **^{1}H NMR** (400 MHz, CD_3CN, 298K): δ [ppm] = 8.41 (d, *J* = 9.0 Hz, 1H, Ar-CH), 8.27 – 8.21 (m, 3H, Ar-CH), 7.98 (ddd, *J* = 8.2, 7.1, 0.9 Hz, 1H, Ar-CH), 7.80 (dd, *J* = 8.3, 1.3 Hz, 1H, Ar-CH), 7.77 – 7.69 (m, 2H, Ar-CH), 7.53 (s, 1H, Pyridine-CH), 7.22 – 7.15 (m, 2H, Ar-CH), 7.03 (s, 4H, Mes-CH), 4.31 (s, 3H, N-CH_3), 2.25 (s, 6H, Mes-CH_3), 2.20 (s, 6H, Mes-CH_3), 2.02 (s, 6H, Mes-CH_3), 1.41 (s, 9H, $C(CH_3)_3$); **^{13}C NMR** (101 MHz, CD_3CN, 298K): δ [ppm] = 172.1 (C_q), 160.6 (C_q), 157.7 (C_q), 143.1 (C_q), 141.5 (C_q), 139.8 (C_q), 138.1 (Ar-CH), 137.5 (C_q), 135.6 (C_q), 135.3 (C_q), 131.6 (Ar-CH), 131.1 (Mes-CH), 130.6 (Ar-CH), 129.8 (Ar-CH), 129.0 (Ar-CH), 127.6 (Ar-CH), 126.6 (Ar-CH), 125.8 (C_q), 121.9 (Ar-CH), 44.5 (N-CH_3), 36.0 ($\mathit{C}(CH_3)_3$), 31.2 ($C(\mathit{C}H_3)_3$), 21.0 (Mes-CH_3), 18.8 (Mes-CH_3); **IR** [cm^{-1}]: $\tilde{v}$ = 3144, 2955, 2872, 1741, 1604, 1578, 1522, 1488,

1464, 1368, 1351, 1294, 1253, 1230, 1178, 1114, 1012, 908, 889, 849, 770, 734, 656, 582, 571; **HR-MS-ESI(+)** calc. for $C_{41}H_{45}N_3^{2+}$: 289.6801 $[M]^{2+}$; found 289.6806; **UV-vis:** λ_{max}: 370 nm (ε = 12874cm^{-1} M^{-1}).

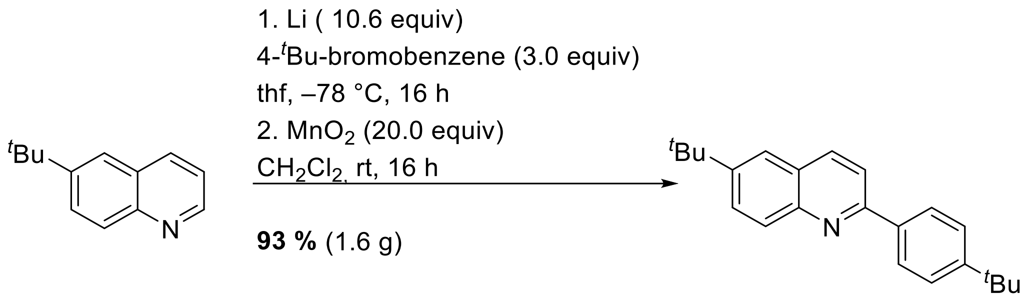

6-^{t}Bu-2-*p*-^{t}Bu-Ph-quinoline **S2:** Elemental lithium stored in paraffin oil (Li, 6.94 g/mol, 397.06 mg, 57.21 mmol, 10.6 equiv) was washed with pentane (10 mL), activated by addition of methanol (5 mL) for 10 seconds, before it was washed with pentane (2 x 10 mL) again and dried in vacuo. The activated lithium was suspended in Et_2O (10 mL). Under cooling to 0 °C and vigorous stirring, 4-^{t}Bu-bromobenzene ($C_{10}H_{13}Br$, 213.12 g/mol, 3.45 g, 3.0 equiv) was added to the suspension. The mixture was stirred for 30 min at (0 °C) and for further 4 h at ambient temperature. The mixture was directly transferred into a cooled (–78 °C) solution of 6-^{t}Bu-quinoline ($C_{13}H_{15}N$, 185.27 g/mol, 1.0 g, 5.4 mmol, 1.0 equiv) in Et_2O (10 mL) with a filter cannular. The reaction mixture was left to slowly warm to ambient temperature for 16 h. The mixture was poured slowly over ice and was extracted with Et_2O three times. The combined organic layers were dried over Na_2SO_4 and all volatiles were removed under reduced pressure. The crude product was dissolved in CH_2Cl_2 (250 mL), activated MnO_2 (MnO_2, 86.94 g/mol, 9.38 g, 107.95 mmol, 20.0 equiv) was added and the mixture was left to stir for 16 h. Filtration over Celite and removal of all volatiles under reduced pressure and subsequent crystallization from *n*-pentane yielded 6-^{t}Bu-2-*p*-^{t}Bu-Ph-quinoline **S2** ($C_{23}H_{27}N$, 317.48 g/mol, 1.6 g, 5.04 mmol, 93 %) as white needles. The spectroscopic data are in good agreement with previous reports.[3]

^{1}H NMR (400 MHz, $CDCl_3$, 298K): δ [ppm] = 8.16 (d, *J* = 8.7 Hz, 1H), 8.09 (t, *J* = 8.7 Hz, 3H), 7.87 – 7.78 (m, 2H), 7.73 (d, *J* = 2.1 Hz, 1H), 7.55 (d, *J* = 8.7 Hz, 2H), 1.44 (s, 9H), 1.38 (s, 9H); **^{13}C NMR** (101 MHz, $CDCl_3$, 298K): δ [ppm] = 157.0, 152.4, 149.1, 147.0, 137.3, 136.7, 129.4, 128.6, 127.4, 126.9, 125.9, 122.6, 119.0, 35.1, 34.9, 31.5, 31.4.

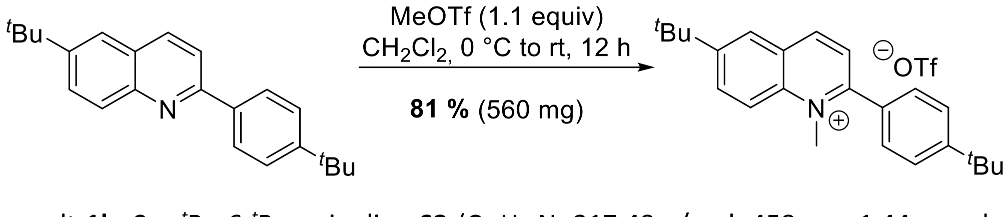

Quinolinium salt **1b:** 2-*p*-^{t}Bu-6-^{t}Bu-quinoline **S2** ($C_{23}H_{27}N$, 317.48 g/mol, 458 mg, 1.44 mmol, 1.0 equiv.) was dissolved in CH_2Cl_2 (10 mL) and cooled to 0°C. Then MeOTf ($C_2H_3F_3O_3S$, 164.1 g/mol, 260.41 mg, 1.5 g/mL, 173.6 µL, 1.59 mmol, 1.1 equiv) was added dropwise. The solution was stirred for 5 min at 0 °C, warmed to room temperature and stirred overnight (12 h). Then the solvent was evaporated until 1-2 mL of the solvent were left over. Then Et_2O (30 mL) was added resulting in the formation of a white solid. The solvent was removed using a filter cannula and the residue solid was washed with Et_2O (3 x 10 mL). Drying

under reduced pressure yielded quinolinium salt **1b** ($C_{25}H_{30}F_3NO_3S$, 481.57 g/mol, 560.0 mg, 1.16 mmol, 81 %) as a colourless solid.

m.p. 145°C; **^{1}H-NMR** (400 MHz, CD_3CN, 298K): δ [ppm] = 8.98 (d, *J* = 8.5 Hz, 1H), 8.42 – 8.29 (m, 3H), 7.93 (dd, *J* = 8.6, 2.4 Hz, 1H), 7.76 (dd, *J* = 8.6, 2.3 Hz, 2H), 7.66 – 7.57 (m, 2H), 4.34 (s, 3H), 1.49 (s, 9H), 1.41 (s, 9H); **^{13}C-NMR** (101 MHz, CD_3CN, 298K): δ [ppm] = 160.3, 156.5, 154.3, 146.7, 139.4, 135.9, 131.4, 130.3, 129.8, 127.4, 126.8, 126.2, 120.0, 43.3, 36.1, 35.8, 31.3, 31.0; **IR** [cm^{-1}]: ṽ = 3070, 2964, 2910, 2870, 1602, 1590, 1506, 1471, 1428, 1395, 1366, 1350, 1257, 1224, 1157, 1115, 1069, 1030, 1012, 925, 838, 820, 777, 756, 702, 675, 636, 615, 574, 517, 468, 444; **HR-MS-ESI(+)** calc. for $C_{24}H_{30}N^+$ 332.2373 [M]$^+$; found 332.2375.

Synthesis of neutral **2b**

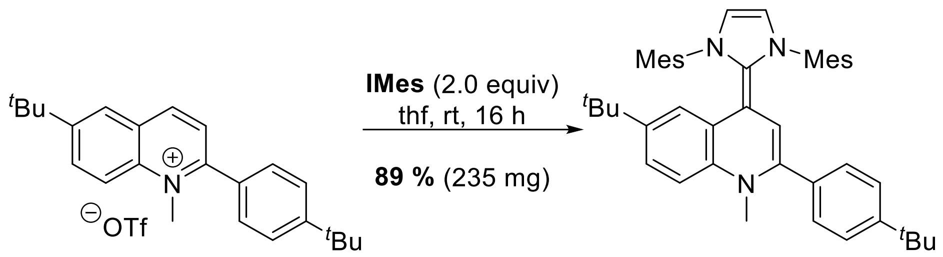

A solution of quinolinium salt **1b** ($C_{25}H_{31}F_3NO_3S$, 481.57 g/mol, 200 mg, 0.15 mmol, 1 equiv) in thf (20 mL) was slowly added to a colorless solution of **IMes** ($C_{21}H_{24}N_2$, 304.44 g/mol, 252.87 mg, 0.831 mmol, 2.0 equiv) in thf (20 mL) and stirred for 16 h. The now red solution was then dried under reduced pressure. The residual solid was extracted with Et_2O (3 x 15 mL) utilising a filter cannula. Removal of the solvent under reduced pressure, afforded switch **2b** ($C_{45}H_{53}N_3$, 635.94 g/mol, 235.0 mg, 369.53 µmol, 89 %) as a red solid.

m.p. 168 °C; **^{1}H NMR** (400 MHz, toluene-*d*$_8$, 298K): δ [ppm] = 7.22 (d, *J* = 8.3 Hz, 2H), 7.17 (d, *J* = 8.9 Hz, 2H), 6.73 (dd, *J* = 8.3, 2.1 Hz, 1H), 6.64 (s, 2H), 6.50 (s, 2H), 6.44 (d, *J* = 8.4 Hz, 1H), 6.28 (d, *J* = 2.1 Hz, 1H), 5.60 (d, *J* = 2.4 Hz, 1H), 5.51 (d, *J* = 2.4 Hz, 1H), 5.31 (s, 1H), 2.89 (s, 3H), 2.44 (s, 6H), 2.41 (s, 6H), 2.03 (s, 3H), 1.97 (s, 3H), 1.29 (s, 9H), 1.21 (s, 9H); **^{13}C NMR** (101 MHz, C_6D_6, 298K): δ [ppm] = 158.2, 154.2, 152.4, 148.8, 147.1, 146.9, 146.8, 146.32, 145.5, 145.3, 143.6, 140.5, 139.8, 138.9, 136.8, 134.9, 131.1, 130.3, 129.2, 127.2, 126.7, 124.4, 120.8, 92.5, 48.6, 44.2, 43.8, 41.4, 41.3, 30.7, 29.8, 28.4, 12.5; **IR** [cm^{-1}]: ṽ = 2952, 2864, 1739, 1606, 1538, 1505, 1482, 1376, 1258, 1227, 1155, 1076, 1031, 1007, 913, 853, 839, 804, 673, 637, 576, 543, 409; **HR-MS-ESI(+)** calc. for $C_{45}H_{54}N_3^+$: 636.4312 [M+H]$^+$; found 636.4311; **UV-vis:** λ_{max}: 382 nm (ε = 10679 cm^{-1} M^{-1}), 466 nm (ε = 12386 cm^{-1} M^{-1}).

Synthesis of radical cation **3b**

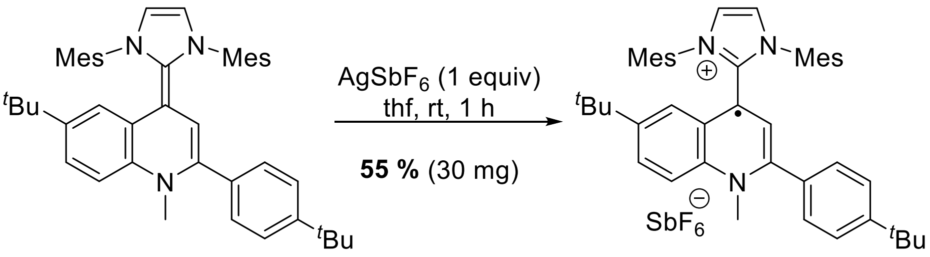

$AgSbF_6$ ($AgSbF_6$, 343.62 g/mol, 21.61 mg, 62.9 µmol, 1.0 equiv) was dissolved in thf (5 mL) and added dropwise to a solution of hybrid system **2b** ($C_{45}H_{53}N_3$, 635.94 g/mol, 40 mg, 62.9 µmol, 1.0 equiv) in thf

(5 mL). The now dark solution was stirred for 1 h at room temperature. After that the solution was filtrated over a celite plug (1.5 cm). Then the solvent was evaporated to give radical cation **3b** ($C_{45}H_{53}F_6N_3Sb\cdot$, 871.69 g/mol, 30 mg, 34.42 µmol, 55 %) as a brown solid.

m.p. 135 °C (decomp.); **EPR:** g = 2.0031 (1xN 13.4045; 2xN 6.7040; 1xH 3.9405; 1xH 4.6503; 3xH 12.0597); **IR** [cm^{-1}]: ṽ = 2959, 2867, 1608, 1558, 1482, 1461, 1381, 1364, 1258, 1231, 1151, 1111, 1063, 1031, 930, 853, 753, 734, 654, 574, 521, 451, 410; **HR-MS-ESI(+)** calc. for $C_{45}H_{54}N_3^+$: 636.4313 [M+H]$^+$; found 636.4297; **UV-vis:** 377nm (ε = 22847 cm^{-1} M^{-1}), 566 nm (ε = 6371 cm^{-1} M^{-1}).

Synthesis of dication **4b**

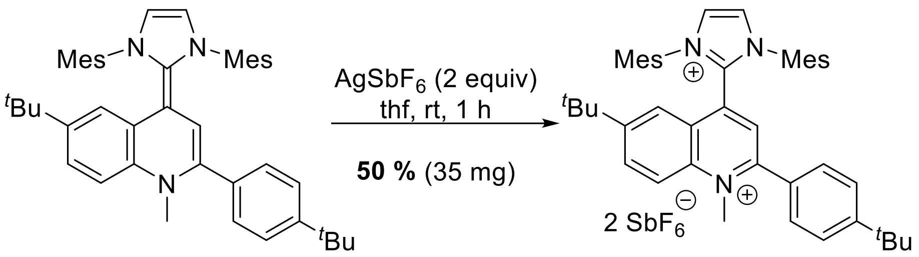

$AgSbF_6$ ($AgSbF_6$, 343.62 g/mol, 43.23 mg, 125.8 µmol, 2.0 equiv) was dissolved in thf (5 mL) and added dropwise to a solution of hybrid system **2b** ($C_{45}H_{53}N_3$, 635.94 g/mol, 40.0 mg, 62.9 µmol, 1.0 equiv) in thf (5 mL). The solution turned dark and was stirred for 1 h. During that time the solution changed to a colourless solution. After that the solution was filtrated over a celite plug (1.5 cm). Then the solvent was evaporated under reduced pressure to give dication **4b** ($C_{45}H_{53}F_{12}N_3Sb_2$, 1107.45 g/mol, 35.0 mg, 31.6 µmol, 50%) as a white powder.

m.p. 168 °C; **^{1}H NMR** (500 MHz, CD_3CN, 298K): δ [ppm] = 8.42 – 8.34 (m, 2H), 8.22 (s, 2H), 7.78 – 7.71 (m, 2H), 7.56 (s, 1H), 7.46 (dd, *J* = 1.9, 0.8 Hz, 1H), 7.24 – 7.18 (m, 2H), 7.06 – 7.02 (m, 4H), 4.30 (s, 3H), 2.24 (s, 6H), 2.23 (s, 6H), 2.04 (s, 6H), 1.41 (s, 9H), 1.37 (s, 9H); **^{13}C NMR** (126 MHz, CD_3CN, 298K): δ [ppm] = 159.4, 157.5, 156.6, 143.1, 140.3, 140.0, 137.2, 135.6, 135.4, 135.0, 131.6, 131.2, 130.6, 130.5, 130.4, 129.7, 128.9, 127.6, 126.3, 122.0, 121.0, 44.5, 36.5, 35.9, 31.2, 30.8, 20.9, 19.5, 19.0; **IR** [cm^{-1}]: ṽ = 3171, 3138, 2960, 2872, 1605, 1578, 1515, 1483, 1386, 1369, 1301, 1253, 1229, 1193, 1139, 1120, 1033, 1012, 930, 884, 850, 827, 769, 733, 654, 607, 580, 548; **HR-MS-ESI(+)** calc. for $C_{45}H_{53}N_3^{2+}$: 317.7114 [M]$^{2+}$; found 317.7124; **UV-vis:** λ_{max}: 374 nm (ε = 14335 cm^{-1} M^{-1}).

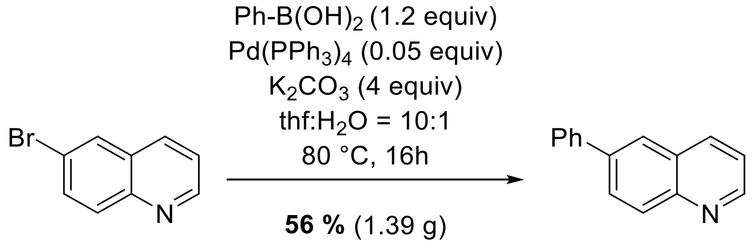

6-Phenylquinoline **S3**: Phenylboronic acid ($C_6H_7BO_2$, 121.93 g/mol, 1.76 g, 14.42 mmol, 1.2 equiv), 6-bromoquinoline (C_9H_6BrN, 208.06 g/mol, 2.5 g, 12.02 mmol, 1 equiv), $Pd(PPh_3)_4$ ($C_{72}H_{60}P_4Pd$, 1155.59 g/mol, 697.27 mg, 600.79 µmol, 0.05 equiv) and potassium carbonate (K_2CO_3, 138.2 g/mol, 6.64 g, 48.06 mmol, 4 equiv) were suspended in a mixture of thf and water (v(thf):v(water) = 10:1). The mixture was stirred for 16 h at 80 °C in a closed pressure *Schlenk* flask. After cooling down to ambient temperature

the mixture was extracted with CH_2Cl_2 (3 x 50 mL) and the combined organic layers were washed with brine and dried over Na_2SO_4. The crude product was adsorbed on silica by addition of silica and removal of all volatiles *in vacuo* on the rotary evaporator. The crude mixture was purified by column chromatography on silica (*n*-hexane/EtOAc: 20/1 to 1/1) to yield 6-phenylquinoline **S3** ($C_{15}H_{11}N$, 205.26 g/mol, 1.39 g, 6.77 mmol, 56.4 %) as a yellow solid. The spectroscopic data are in good agreement with previous reports.[4]

^{1}H NMR (400 MHz, $CDCl_3$, 298K): δ [ppm] = 8.93 (d, *J* = 4.3 Hz, 1H), 8.21 (t, *J* = 9.2 Hz, 2H), 8.00 (dd, *J* = 8.3, 1.4 Hz, 2H), 7.72 (d, *J* = 8.4 Hz, 2H), 7.51 (t, *J* = 7.5 Hz, 2H), 7.46 – 7.38 (m, 2H). **^{13}C NMR** (101 MHz, $CDCl_3$, 298K): δ [ppm] = 150.4, 147.7, 140.4, 139.5, 136.5, 130.0, 129.5, 129.1, 128.6, 127.9, 127.6, 125.6, 121.6.

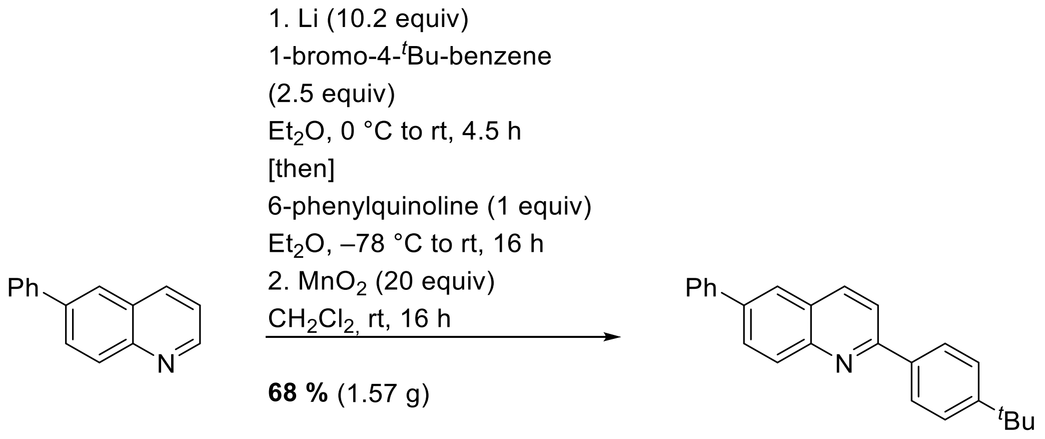

2-*p*-^{t}Bu-6-phenylquinoline **S4:** Elemental lithium stored in paraffin oil (Li, 6.94 g/mol, 479.37 mg, 69.07 mmol, 10.2 equiv), was washed with pentane (10 mL), activated by addition of methanol (5 mL) for 10 seconds, before it was washed with pentane (2 x 10 mL) again and dried in vacuo. The activated lithium was suspended in Et_2O 10 mL). Under cooling to 0 °C and vigorous stirring, 4-^{t}Bu-bromobenzene ($C_{10}H_{13}Br$, 212.12 g/mol, 3.61 g, 16.93 mmol, 2.50 equiv) was added to the suspension. The mixture was stirred for 30 min (0 °C) and for further 4 h at ambient temperature. The mixture was directly transferred into a cooled (-78 °C) solution of 6-phenylquinoline **S3** ($C_{15}H_{11}N$, 205.26 g/mol, 1.39 g, 6.77 mmol, 1.0 equiv) in Et_2O (10 mL) with a filter cannula. The reaction mixture was left to slowly warm to ambient temperature for 16 h. The mixture was poured slowly over ice and was extracted with Et_2O three times. The combined organic layers were dried over Na_2SO_4 and all volatiles were removed under reduced pressure. The crude product was dissolved in CH_2Cl_2 (250 mL), activated MnO_2 (MnO_2, 86.94 g/mol, 11.77 g, 135.44 mmol, 20.0 equiv) was added and the mixture was left to stir for 16 h. Filtration over Celite and removal of all volatiles under reduced pressure yielded the crude product which was recrystallized from *n*-pentane to yield 2-*p*-^{t}Bu-6-phenylquinoline **S4** ($C_{25}H_{23}N$, 337.47 g/mol, 1.57 g, 4.65 mmol, 68%) as white needles.

m.p.: 192 °C; **^{1}H NMR** (300 MHz, $CDCl_3$, 298K): δ [ppm] = 8.24 (t, *J* = 8.0 Hz, 2H), 8.12 (d, *J* = 8.5 Hz, 2H), 8.04 – 7.96 (m, 2H), 7.90 (d, *J* = 8.7 Hz, 1H), 7.75 (dd, *J* = 6.9, 1.5 Hz, 2H), 7.62 – 7.48 (m, 4H), 7.40 (t, *J* = 7.4 Hz, 1H), 1.39 (s, 9H); **^{13}C NMR** (101 MHz, $CDCl_3$, 298K): δ [ppm] = 157.5, 152.8, 147.6, 140.6, 139.0, 137.0, 130.2, 129.5, 129.1, 127.8, 127.6, 127.4, 127.4, 126.0, 125.8, 125.3, 119.5, 34.9, 31.5; **IR** [cm^{-1}]: ṽ = 3063, 3046, 2949, 2901, 2864, 1594, 1552, 1506, 1489, 1442, 1409,1361, 1338, 1295, 1267, 1200, 1156, 1140, 1110, 1074, 1012, 979, 891, 845, 834, 816, 789, 758, 742, 688, 644, 610, 599, 555, 496, 478, 439; **HR-MS-ESI(+)** calc. for $C_{25}H_{24}N^+$: 338.1904 $[M+H]^+$; found 338.1905.

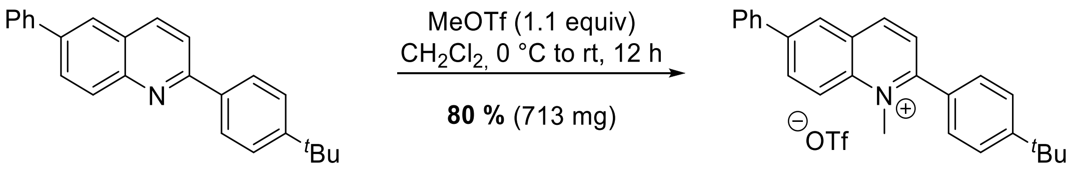

Quinolinium salt **1c:** 2- *p*-^{t}Bu-6-phenylquinoline **S4** ($C_{25}H_{23}N$, 337.47 g/mol, 600 mg 1.78 mmol, 1.0 equiv.) was dissolved in CH_2Cl_2 (20 mL) and cooled to 0°C. Then MeOTf ($C_2H_3F_3O_3S$, 164.1 g/mol, 320.94 mg, 1.5 g/mL, 213.96 µL, 1.96 mmol, 1.1 equiv) was added dropwise. The solution was stirred for 5 min at 0 °C, warmed up to room temperature and stirred overnight (12 h). Then the solvent was evaporated until 1-2 mL of the solvent were left over. Then Et_2O (30 mL) was added resulting in the formation of a white solid. The solvent was removed using a filter cannula and the residue solid was washed with Et_2O (3 x 10 mL). Drying of the solid yielded quinolinium salt **1c** ($C_{27}H_{26}F_3NO_3S$, 501.56 g/mol, 713.0 mg, 1.42 mmol, 80 %) as a white solid powder.

m.p. 200 °C; **^{1}H-NMR** (500 MHz, CD_3CN, 298K): δ [ppm] = 9.06 (d, *J* = 8.5 Hz, 1H, ^{p}*t*-Bu-Ph-C*H*), 8.61 (d, *J* = 2.2 Hz, 1H, Ar-CH), 8.56 (dd, *J* = 9.2, 2.2 Hz, 1H, Ar-CH), 8.50 (d, *J* = 9.2 Hz, 1H, Ar-CH), 7.98 (d, *J* = 8.5 Hz, 1H, ^{p}*t*-Bu-Ph-C*H*), 7.93 – 7.86 (m, 2H, Ar-CH), 7.81 – 7.74 (m, 2H, Ar-CH), 7.70 – 7.64 (m, 2H, Ar-CH), 7.60 (t, *J* = 7.5 Hz, 2H, Ar-CH), 7.57 – 7.51 (m, 1H, Ar-CH), 4.40 (s, 3H-N-C*H*$_3$), 1.42 (s, 9H, C(C*H*$_3$)$_3$); **^{13}C-NMR** (126 MHz, CD_3CN, 298K): δ [ppm] = 160.9 (C_q), 156.6 (C_q), 146.9 (^{p}*t*-Bu-Ph-*C*H), 142.8 (C_q), 140.2 (C_q), 138.6 (C_q), 136.0 (Ar-CH), 131.3 (C_q), 130.4 (Ar-CH), 130.2 (Ar-CH), 130.2 (Ar-CH), 128.6 (Ar-CH), 128.4 (Ar-CH), 127.4 (Ar-CH), 126.7 (^{p}*t*-Bu-Ph-*C*H), 121.0 (Ar-CH), 43.5 (N-*C*H$_3$), 35.9 (*C*(CH$_3$)$_3$), 31.3 (C(*C*H$_3$)$_3$); **IR** [cm^{-1}]: ṽ = 2967, 2867, 1603, 1587, 1496, 1456, 1426, 1396, 1371, 1347, 1224, 1203, 1176, 1144, 1114, 1079, 1030, 914, 890, 855, 830, 779, 767, 698, 635, 603, 570, 515, 488, 436, 410; **HR-MS-ESI(+)** calc. for $C_{26}H_{26}N^+$: 352.2060 [M]$^+$; found 352.2060.

Synthesis of neutral **2c**

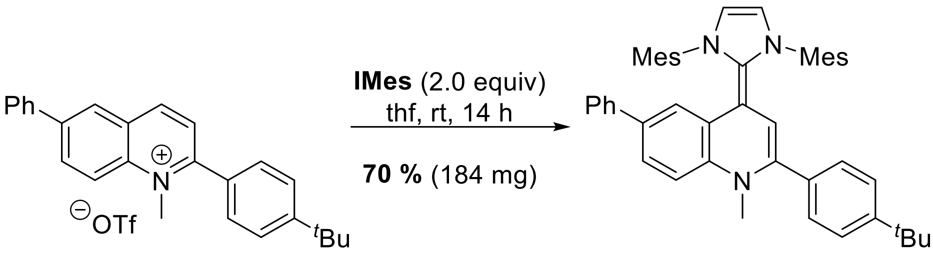

A suspension of quinolinium salt **1c** ($C_{27}H_{26}F_3NO_3S$, 501.56 g/mol, 200 mg, 0.399 mmol, 1.0 equiv) in thf (10 mL) was slowly added to a colorless solution of **IMes** ($C_{21}H_{24}N_2$, 304.44 g/mol, 242.79 mg, 0.798 mmol, 2.0 equiv) in thf (10 mL) and stirred for 14 h. The now red solution was then dried under reduced pressure. Then Et_2O was added and the suspension was then filtrated using a filter cannula and the residue solid was extracted with Et_2O. The combined organic phases were dried under reduced pressure, which gave the switch **2c** ($C_{47}H_{49}N_3$, 655.93 g/mol, 184.0 mg, 280.52 µmol, 70 %) as a red solid.

Single crystals suitable for X-ray diffraction could be obtained from a saturated Et_2O solution at –40 °C.

m.p. 140 °C; **^{1}H NMR** (500 MHz, C_6D_6, 298K): δ [ppm] = 7.37 – 7.34 (m, 2H, Ar-CH), 7.28 – 7.24 (m, 2H, Ar-CH), 7.24 – 7.20 (m, 2H, Ar-CH), 7.20 – 7.17 (m, 1H, Ar-CH), 7.14 – 7.10 (m, 2H, Ar-CH), 6.84 (dd, *J* = 8.1, 2.1 Hz, 1H), 6.76 (s, 2H, Ar-CH), 6.49 (d, *J* = 2.2 Hz, 1H, Ar-CH), 6.34 – 6.30 (m, 3H, Ar-CH), 5.73 (s, 1H, Ar-CH), 5.70 (s, 1H, Ar-CH), 5.24 (s, 1H, Pyridine-CH), 2.81 (s, 3H, N-CH$_3$), 2.39 (s, 6H, Mes-CH$_3$), 2.25 (s, 6H, Mes-CH$_3$), 2.04 (s, 3H, Mes-CH$_3$), 2.01 (s, 3H, Mes-CH$_3$), 1.22 (s, 9H, C(C*H*$_3$)$_3$); **^{13}C NMR** (126 MHz, C_6D_6, 298K): δ

[ppm] = 148.5 (C_q), 146.7 (C_q), 142.7 (C_q), 139.4 (C_q), 137.8 (C_q), 137.1 (C_q), 136.8 (C_q), 136.5 (C_q), 135.8 (C_q), 135.2 (C_q), 133.6 (C_q), 133.2 (C_q), 130.8 (C_q), 129.9 (Ar-CH), 129.8 (Ar-CH), 127.0 (Ar-CH), 126.7 (Ar-CH, 125.8 (Ar-CH), 125.2 (Ar-CH), 122.1 (Ar-CH), 121.7 (Ar-CH), 116.9 (Ar-CH), 116.7 (Ar-CH), 112.9 (Ar-CH), 111.0 (Pyridine-CH), 81.7 (C_q), 38.7 (N-CH_3), 34.4 ($C(CH_3)_3$, 31.5 ($C(CH_3)_3$, 21.0 (Mes-CH_3), 20.9 (Mes-CH_3), 19.2 (Mes-CH_3), 18.6 (Mes-CH_3); **IR** [cm^{-1}]: $\tilde{v}$ = 3027, 2952, 2863, 1742, 1599, 1531, 1478, 1376, 1362, 1320, 1279, 1259, 1227, 1200, 1158, 1128, 1109, 1075, 1049, 1031, 1008, 983, 934, 913, 885, 840, 822, 760, 697, 666, 637, 614, 577, 563, 549,499; **HR-MS-ESI(+)** calc. for $C_{47}H_{50}N_3^+$: 656.4000 $[M+H]^+$; found 656.4000; **UV-vis:** λ_{max}: 286 nm (ε = 23552 cm^{-1} M^{-1}), 432 nm (ε = 16104 cm^{-1} M^{-1}).

Synthesis of radical cation **3c**

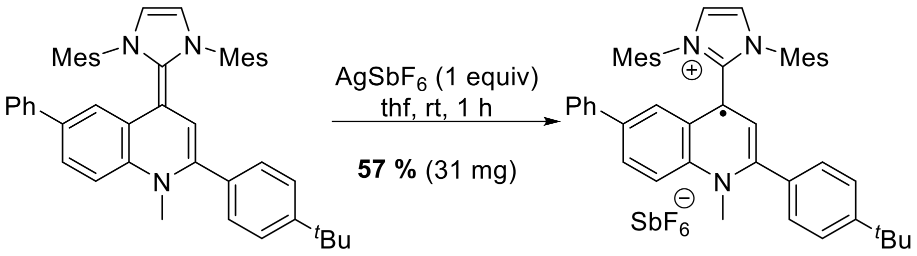

$AgSbF_6$ ($AgSbF_6$, 343.62 g/mol, 20.95 mg, 60.98 µmol, 1.0 equiv) was dissolved in thf (5 mL) and added dropwise to a solution of hybrid system **2c** ($C_{47}H_{49}N_3$, 655.93 g/mol, 40 mg, 60.98 µmol, 1.0 equiv) in thf (5 mL). The now dark solution was stirred for 1 h at room temperature. The solution was filtrated over a celite plug (1.5 cm). Then the solvent was evaporated to give radical cation **3c** ($C_{47}H_{49}F_6N_3Sb\cdot$, 891.68 g/mol, 31 mg, 34.77 µmol, 57 %) as a dark purple solid.

m.p. 133 °C; **EPR:** g = 2.0030 (1xN 13.1514; 1xN 6.0936; 1xN 6.8074; 1xH 4.0244; 1xH 4.5544; 1xH 1.5309; 3xH 11.4908.); **IR** [cm^{-1}]: $\tilde{v}$ = 2969, 2867, 1737, 1606, 1556, 1450, 1365, 1263, 1229, 1216, 1108, 1029, 853, 763, 725, 700, 655, 571; **HR-MS-ESI(+)** calc. for $C_{48}H_{52}N_3O^+$: 686.4105 $[M+OCH_3]^+$; found 686.4086; **UV-vis:** 280 nm (ε = 35514 cm^{-1} M^{-1}), 390 nm (ε = 16995 cm^{-1} M^{-1}), 480 nm (ε = 8267 cm^{-1} M^{-1}).

Synthesis of dication **4c**

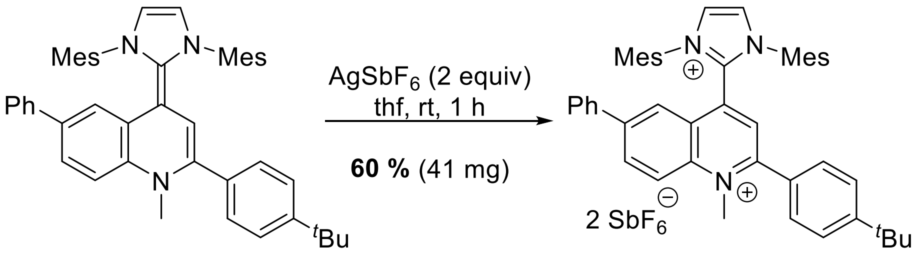

$AgSbF_6$ ($AgSbF_6$, 343.62 g/mol, 41.91 mg, 121.96 µmol, 2.0 equiv) was dissolved in thf (5 mL) and added dropwise to a solution of hybrid system **2c** ($C_{47}H_{49}N_3$, 655.93 g/mol, 40.0 mg, 60.98 µmol, 1.0 equiv) in thf (5 mL). The solution turned dark and was stirred for 1 h. During that time the solution changed to a yellow solution. After that the solution was filtrated over a celite plug (1.5 cm). Then the solvent was evaporated to give dication **4c** ($C_{47}H_{49}F_{12}N_3Sb_2$, 1127.44 g/mol, 41.0 mg, 36.37 µmol, 60 %) as a yellow powder.

m.p. 187 °C (decomp.); **^{1}H NMR** (500 MHz, CD_3CN, 298K): δ [ppm] = 8.48 (d, J = 9.2 Hz, 1H, Ar-CH), 8.44 (dd, J = 9.2, 2.0 Hz, 1H, Ar-CH), 8.21 (s, 2H, Ar-CH), 7.76 – 7.70 (m, 2H, Ar-CH), 7.67 – 7.61 (m, 3H, Ar-CH), 7.63 – 7.58 (m, 2H, Ar-CH), 7.51 (s, 1H, Ar-CH), 7.22 – 7.14 (m, 2H, Ar-CH), 7.02 (s, 2H, Mes-CH), 6.84 (s, 2H, Mes-CH), 4.34 (s, 3H, N-CH_3), 2.23 (s, 6H, Mes-CH_3), 2.05 (s, 6H, Mes-CH_3), 2.01 (s, 6H, Mes-CH_3), 1.41

(s, 9H, C(CH_3)$_3$); **^{13}C NMR** (126 MHz, CD_3CN, 298K): δ [ppm] = 160.1 (C_q), 157.6 (C_q), 145.1 (C_q), 143.0 (C_q), 140.9 (C_q), 139.7 (C_q), 137.7 (C_q), 137.5 (Ar-CH), 135.5 (C_q), 135.2 (C_q), 131.4 (Mes-CH), 131.1 (Mes-CH), 130.8 (Ar-CH), 130.7 (C_q), 130.5 (Ar-CH), 130.4 (Ar-CH), 130.0 (Ar-CH), 129.7 (C_q), 129.0 (Ar-CH), 128.9 (Ar-CH), 127.5 (Ar-CH), 126.0 (C_q), 123.2 (Ar-CH), 122.5 (Ar-CH), 44.6 (N-CH_3), 35.9 (*C*(CH_3)$_3$), 31.2 (C(*C*H_3)$_3$), 20.9 (Mes-CH_3), 18.8 (Mes-CH_3); **IR** [cm^{-1}]: ṽ = 3179, 3153, 2964, 2870, 1738, 1606, 1579, 1557, 1482, 1387, 1354, 1257, 1227, 1140, 1113, 1088, 1033, 930, 862, 840, 764, 739, 723, 700, 609, 589, 568, 514; **HR-MS-ESI(+)** calc. for $C_{47}H_{49}N_3^{2+}$: 327.6958 [M]$^{2+}$; found 327.6962; **UV-vis:** λ_{max}: 270 nm (ε = 33018 cm^{-1} M^{-1}), 289 nm (ε = 28283 cm^{-1} M^{-1}), 387 nm (ε = 13434 cm^{-1} M^{-1}).

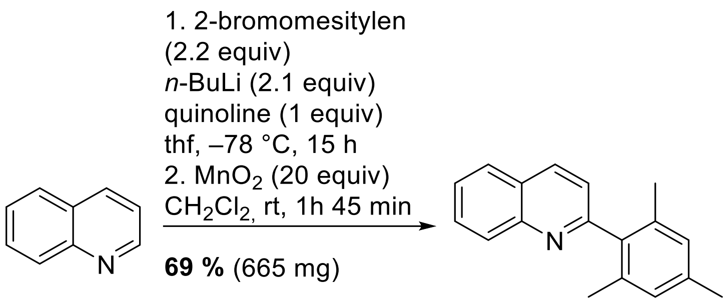

2-Mesitylene-quinoline **S5:** 2-Bromomesitylen ($C_9H_{11}Br$, 199.09 g/mol, 1.7 g, 1.3 g/mL, 1.3 mL, 8.52 mmol, 2.2 equiv) was dissolved in thf (15 mL) and cooled down to –78 °C. Then *n*-BuLi (C_4H_9Li, 64.06 g/mol, 520.73 mg, 1.6 M, 5.08 mL, 8.13 mmol, 2.1 equiv) was slowly added and the resulting white suspension was stirred for 10 minutes at –78 °C. This suspension was then added to a solution of quinoline (C_9H_7N, 129.16 g/mol, 500 mg, 1.09 g/mL, 458.72 mL, 3.87 mmol, 1 equiv) in thf (15 mL), which was also cooled to –78 °C. The quinoline solution turned orange instantly and was allowed to warm up to room temperature over night (15 h). The now red to orange solution was slowly poured over ice and then extracted using ethylacetate (3 x 50 mL). The combined organic phases were dried using Na_2SO_4 and the residue solvent was removed under reduced pressure. The yellow oil was then dissolved in CH_2Cl_2 (200 mL) and activated MnO_2 (MnO_2, 86.94 g/mol, 6.73 g, 77.42 mmol, 20 equiv) was added resulting in a brown suspension. After 1 hour and 45 minutes the suspension was filtrated over a silica plug (2 cm) using EtOAc as solvent until the solution was colourless. The crude product was then purified by column chromatography on silica (Cyclohexane/EtOAc: 100/1 to 50/1 to 25/1) to yield 2-mesitylene-quinoline **S5** ($C_{18}H_{17}N$, 247.34 g/mol, 665 mg, 2.69 mmol, 69 %) as a yellow oil. The spectroscopic data are in good agreement with previous reports.[5]

^{1}H NMR (500 MHz, $CDCl_3$, 298K): δ [ppm] = 8.21 (d, *J* = 9.2 Hz, 1H), 8.16 (d, *J* = 8.4 Hz, 1H), 7.88 (dd, *J* = 8.2, 1.8 Hz, 1H), 7.74 (ddd, *J* = 8.4, 6.9, 1.4 Hz, 1H), 7.57 (ddd, *J* = 8.1, 6.9, 1.3 Hz, 1H), 7.36 (d, *J* = 8.4 Hz, 1H), 6.97 (s, 2H), 2.35 (s, 3H), 2.05 (s, 6H); **^{13}C NMR** (126 MHz, $CDCl_3$, 298K): δ [ppm] = 160.77, 148.28, 138.00, 137.79, 136.35, 135.78, 129.67, 129.63, 128.57, 127.70, 126.86, 126.50, 123.06, 21.25, 20.28.

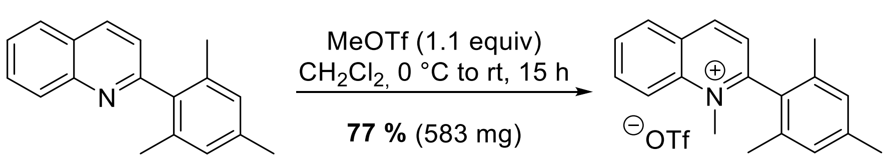

N-Methyl-2-mesitylene-quinolinium salt **1d:** 2-Mesitylene-quinoline **S5** ($C_{18}H_{17}N$, 247.34 g/mol, 452.5 mg, 1.83 mmol, 1 equiv) was dissolved in CH_2Cl_2 (20 mL) and cooled to 0°C. Then MeOTf ($C_2H_3F_3O_3S$, 164.1 g/mol, 330.23 mg, 1.5 g/mL, 220.15 µL, 2.01 mmol, 1.1 equiv) was added dropwise. The yellow solution turned red immediately and was allowed to warm up to room temperature over night (15 h). Then the solvent was evaporated until 1-2 mL of the solvent were left over. Et_2O (30 mL) was added resulting in the formation of a pale orange solid. The solvent was removed using a filter cannula and the residue solid was washed with Et_2O (3 x 10 mL). Drying of the solid yielded *N*-methyl-2-mesitylene-quinolinium salt **1d** ($C_{20}H_{20}F_3NO_3S$, 411.44 g/mol, 582.8 mg, 1.42 mmol, 77 %) as a pale orange powder.

m.p. 171 °C; **^{1}H NMR** (500 MHz, CD_3CN, 298K): δ [ppm] = 9.12 (d, *J* = 8.4 Hz, 1H, Ar-CH), 8.48 (d, *J* = 9.2 Hz, 1H, Ar-CH), 8.43 (d, *J* = 9.9 Hz, 1H, Ar-CH), 8.29 (ddd, *J* = 9.0, 7.1, 1.6 Hz, 1H, Ar-CH), 8.07 (t, *J* = 8.2 Hz, 1H, Ar-CH), 7.91 (d, *J* = 8.4 Hz, 1H, Ar-CH), 7.18 (s, 2H-Mes-CH), 4.27 (s, 3H, N-CH_3), 2.40 (s, 3H, Mes-$^{para}CH_3$), 1.98 (s, 6H, , Mes-$^{ortho}CH_3$); **^{13}C NMR** (126 MHz, CD_3CN, 298K): δ [ppm] = 161.2 (C_q), 148.1 (Ar-CH), 142.6 (C_q), 141.1 (C_q), 136.9 (Ar-CH), 136.6 (C_q), 131.6 (Ar-CH), 131.2 (Ar-CH), 130.7 (C_q), 130.2 (C_q), 130.0 (Mes-CH), 125.9 (Ar-CH), 120.6 (Ar-CH), 41.3 (N-CH_3), 21.3 (Mes-$^{para}CH_3$), 19.9 (Mes-$^{ortho}CH_3$); **IR** [cm^{-1}]: $\tilde{\nu}$ = 1602, 1581, 1519, 1440, 1382, 1349, 1263, 1224, 1148, 1029, 875, 861, 840, 783, 754, 636, 598, 573, 516; **HR-MS-ESI(+)** calc. for $C_{19}H_{20}N^+$: 262.1590 $[M]^+$; found 262.1597.

Synthesis of neutral **2d**

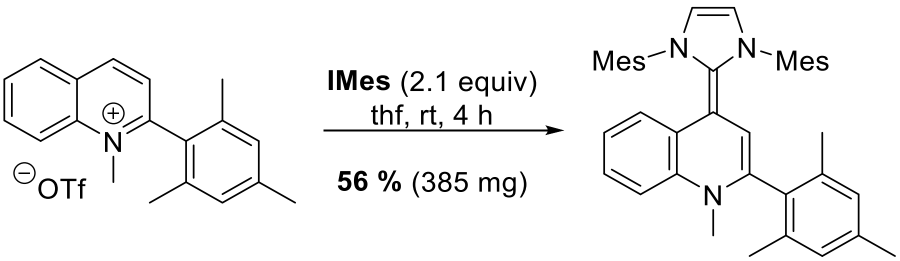

A suspension of *N*-methyl-2-mesitylene-quinolinium salt **1d** ($C_{20}H_{20}F_3NO_3S$, 411.44 g/mol, 500 mg, 1.22 mmol, 1 equiv) in thf (15 mL) was slowly added to a colorless solution of **IMes** ($C_{21}H_{24}N_2$, 304.44 g/mol, 776.93 mg, 2.55 mmol, 2.1 equiv) in thf (20 mL) and stirred for 4 h. The now red solution was then dried under reduced pressure. Then Et_2O (20 mL) was added and the suspension was sonicated in a super-sonic bath for 1 minute. The suspension was then filtrated using a filter cannula and the residue solid was extracted with Et_2O (3x 20 mL and 2 x 10 mL). The united organic phase was dried under reduced pressure. The solid was then washed with pentane (1 x 8 mL and 2 x 5 mL) again using a super-sonic bath for the first time washing. The residue solvent was evaporated under reduced pressure, which gave hybrid system **2d** ($C_{40}H_{43}N_3$, 565.81 g/mol, 385.3 mg, 680.98 µmol, 56 %) as a red solid.

Note: To obtain NMR spectra it proved necessary to add a small amount of KHMDS (0.5 mg -1 mg) to the freshly prepared NMR sample in C_6D_6, otherwise only broad spectra were observed.

m.p. 217 °C; **^{1}H NMR** (500 MHz, C_6D_6, 298K): δ [ppm] = 6.65 (m, 8H, overlapping signals including: Ar-CH, Mes-CH), 6.38 (t, *J* = 7.2 Hz, 1H, Ar-CH), 6.06 (d, *J* = 7.8 Hz, 1H, Ar-CH), 5.77 (d, *J* = 2.6 Hz, 1H, NC*H*CHN), 5.67 (d, *J* = 2.6 Hz, 1H, NCHC*H*N), 4.56 (s, 1H, Qu-CH), 2.49 (s, 6H, Mes-CH_3), 2.32 (s, 6H, Mes-CH_3), 2.31 (s, 3H, N-CH_3), 2.21 (s, 6H, Mes-CH_3), 2.02 (s, 3H, Mes-CH_3), 2.02 (s, 3H, Mes-CH_3), 1.91 (s, 3H, Mes-CH_3); **^{13}C NMR** (126 MHz, C_6D_6, 298K): δ [ppm] = 145.5 (C_q), 139.3 (C_q), 137.7 (C_q), 137.0 (C_q), 136.8 (C_q), 136.3 (C_q), 136.2 (C_q), 135.7 (C_q), 135.6 (C_q), 134.5 (C_q), 133.2 (C_q), 130.0 (Mes-CH), 129.8 (Mes-CH), 129.7 (C_q), 128.4 (C_q), 128.4 (C_q), 123.1 (Ar-CH), 122.5 (Ar-CH), 119.5 (Ar-CH), 117.3 (N*C*HCHN), 117.1 (NCH*C*HN), 109.7 (Ar-

CH), 105.2 (Qu-CH), 82.9 (C_q), 33.3 (N-CH_3), 21.1 (Mes-CH_3), 20.9 (Mes-CH_3), 20.9 (Mes-CH_3), 20.4 (Mes-CH_3), 19.1 (Mes-CH_3), 18.5 (Mes-CH_3); **IR** [cm^{-1}]: $\tilde{\nu}$ = 2946, 2913, 1772, 1632, 1611, 1555, 1482, 1444, 1393, 1373, 1267, 1246, 1198, 1187, 1157, 1082, 1066, 1032, 1004, 978, 910, 851, 832, 794, 711, 695, 676, 662, 649, 638, 611, 571, 541, 517, 494, 430; **HR-MS-ESI(+)** calc. for $C_{40}H_{44}N_3^+$: 566.3530 $[M+H]^+$; found 566.3517; **UV-vis:** λ_{max}: 319 nm (ε = 12304 cm^{-1} M^{-1}), 395 nm (ε = 11170 cm^{-1} M^{-1}).

Synthesis of radical cation **3d**

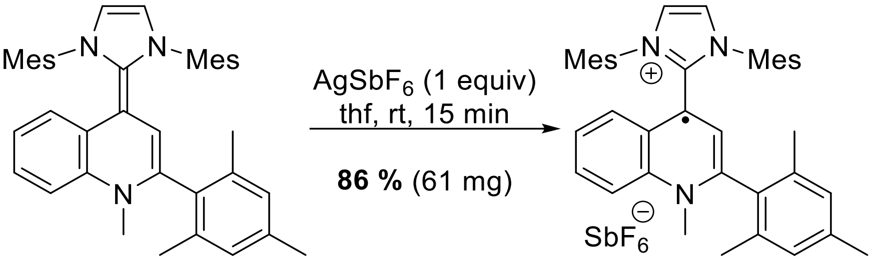

$AgSbF_6$ ($AgSbF_6$, 343.62 g/mol, 30.37 mg, 88.37 µmol, 1 equiv) was dissolved in thf (2 mL) and added dropwise to a solution of hybrid system **2d** ($C_{40}H_{43}N_3$, 565.81 g/mol, 50 mg, 88.37 µmol, 1 equiv) in thf (4 mL). The now dark purple solution was stirred for 15 minutes at room temperature. After that the solution was filtrated over a celite plug (1.5 cm). Then the solvent was evaporated and the resulting dark solid was washed with pentane (2 x 4 mL). The dark solid was dried to give radical cation **3d** ($C_{40}H_{43}F_6N_3Sb\cdot$, 801.56 g/mol, 61 mg, 76.1 µmol, 86 %) as a black solid.

m.p. 114 °C; **EPR:** g = 2.0036 (1xN 12.4644; 1xN 6.3077; 1xN 6.3061; 1xH 8.2999; 1xH 4.7328; 1xH 6.4536; 3xH 13.3210); **IR** [cm^{-1}]: $\tilde{\nu}$ = 2972, 2920, 2860, 1609, 1557, 1544, 1445, 1379, 1230, 1212, 1030, 929, 854, 751, 737, 722, 652, 573; **HR-MS-ESI(+)** calc. for $C_{40}H_{43}N_3^{2+}$: 282.6723 $[M]^{2+}$; found 282.6715; **UV-vis:** 344 nm (ε = 7022 cm^{-1} M^{-1}), 401 nm (ε = 3725 cm^{-1} M^{-1}), 560 nm (ε = 2550 cm^{-1} M^{-1}).

Synthesis of dication **4d**

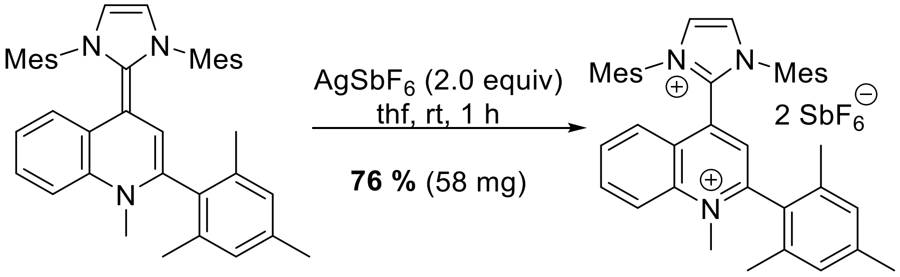

$AgSbF_6$ ($AgSbF_6$, 343.62 g/mol, 50.53 mg, 147.05 µmol, 2.0 equiv) was dissolved in thf (2 mL) and added dropwise to a solution of switch **2d** ($C_{40}H_{43}N_3$, 565.81 g/mol, 41.6 mg, 73.52 µmol, 1 equiv) in thf (4 mL). The solution turned dark purple and was stirred for 1 h. During that time the solution changed to a light green, clear solution. After that the solution was filtrated over a celite plug (1.5 cm). Then the solvent was evaporated until 1 mL of the solution was left. Then Et_2O (20 mL) was added to form a green oil. The Et_2O was decanted off and the green oil was redissolved in thf (1 mL) and then treated with pentane (20 mL) to form a pale green solid. After three minutes the pentane was decanted off and solid was dried to give dication **4d** ($C_{40}H_{43}F_{12}N_3Sb_2$, 1037.31 g/mol, 57.8 mg, 55.72 µmol, 76 %) as a beige powder.

m.p. 164 °C (decomp.); **^{1}H NMR** (500 MHz, CD_3CN, 298K): δ [ppm] = 8.47 (d, *J* = 9.2 Hz, 1H, Ar-CH), 8.27 (ddd, *J* = 9.0, 7.0, 1.4 Hz, 1H, Ar-CH), 8.22 (s, 2H, NC*H*C*H*N), 8.05 (ddd, *J* = 8.2, 7.1, 1.1 Hz, 1H, Ar-CH), 7.86

(dd, J = 8.5, 1.5 Hz, 1H, Ar-CH), 7.61 (s, 1H, Qu-CH), 7.11 (s, 2H, Mes-CH), 7.01 (s, 2H, Mes-CH), 6.98 (s, 2H, Mes-CH), 4.19 (s, 3H, N-CH_3), 2.38 (s, 3H, Mes-CH_3), 2.23 (s, 6H, Mes-CH_3), 2.21 (s, 6H, Mes-CH_3), 2.03 (s, 6H, Mes-CH_3), 1.59 (s, 6H, Mes-CH_3); **^{13}C NMR** (126 MHz, CD_3CN, 298K): δ [ppm] = 161.1 (C_q), 143.4 (C_q), 143.2 (C_q), 142.4 (C_q), 139.6 (C_q), 138.1 (Ar-CH), 136.9 (C_q), 136.8 (C_q), 135.5 (C_q), 135.4 (C_q), 132.7 (Ar-CH), 131.5 (Mes-CH), 131.3 (Mes-CH), 130.6 (C_q), 130.4 (C_q), 130.2 (C_q), 129.1 (Mes-CH), 129.0 (C_q), 129.0 (N*C*H*C*HN), 127.0 (Ar-CH), 126.9 (C_q), 122.7 (Ar-CH), 42.6 (N-CH_3), 21.3 (Mes-CH_3), 21.0 (Mes-CH_3), 19.7 (Mes-CH_3), 18.9 (Mes-CH_3), 18.8 (Mes-CH_3); **IR** [cm^{-1}]: $\tilde{\nu}$ = 3153, 1609, 1572, 1560, 1521, 1486, 1342, 1233, 1165, 1121, 1054, 905, 853, 803, 764, 735, 652, 602, 574; **HR-MS-ESI(+)** calc. for $C_{40}H_{43}N_3^{2+}$: 282.6723 $[M]^{2+}$; found 282.6712; **UV-vis:** λ_{max}: 330 nm (ε = 8807 cm^{-1} M^{-1}).

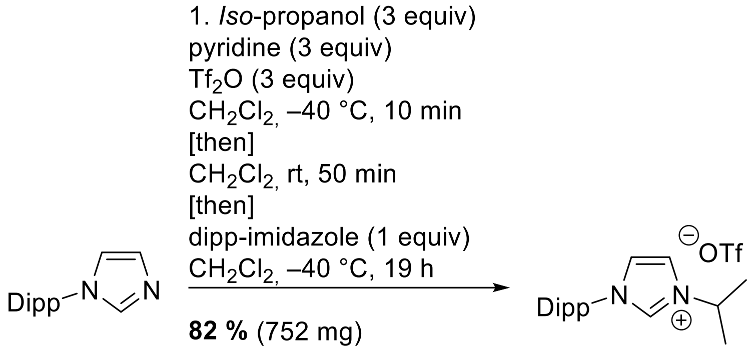

Imidazolium salt **S6**: *Iso*-propanol (C_3H_8O, 60.10 g/mol, 394.78 mg, 0.786 g/mL, 502.27 µL, 6.57 mmol, 3 equiv) was dissolved in CH_2Cl_2 (20 mL). Then pyridine (C_5H_5N, 79.1 g/mol, 519.64 mg, 0.982 g/mL, 529.16 µL, 6.57 mmol, 3 equiv) was added and the solution was cooled to –40 °C. After that trifilic anhydride ($C_2F_6O_5S_2$, 282.13 g/mol, 1.85 g, 1.68 g/mL, 1.1 mL, 6.57 mmol, 3 equiv) was added and the resulting suspension was stirred at –40 °C for 10 minutes and additional 50 minutes at room temperature. The suspension was again cooled to –40 °C and treated with pentane (20 mL) resulting in the formation of a colourless solid. The solution was now transferred dropwise in a second Schlenk containing a –40 °C cold solution of dipp-imidazol ($C_{15}H_{20}N_2$, 228.34 g/mol, 500 mg, 2.19 mmol, 1 equiv) in CH_2Cl_2 (20 mL) employing a filtration cannula. After that the light yellow solution was allowed to warm up to ambient temperature and stirred for 19 hours. A saturated K_2CO_3 solution (60 mL) was added and the biphasic solution was extracted with CH_2Cl_2 (3x 50 mL). The combined organic layers were dried over Na_2SO_4 and all volatiles were removed under reduced pressure. The crude product was then purified by column chromatography on silica gel (CH_2Cl_2/MeOH: 50/1 to 25/1) to yield imidazolium salt **S6** ($C_{19}H_{27}F_3N_2O_3S$, 420.49 g/mol, 751.8 mg, 1.79 mmol, 81.7 %) as a white solid. The spectroscopic data are in good agreement with previous reports.[6]

^{1}H NMR (500 MHz, CD_3CN, 298K): 8.72 (t, J = 1.7 Hz, 1H), 7.75 (t, J = 1.8 Hz, 1H), 7.60 (t, J = 7.8 Hz, 1H), 7.55 (t, J = 1.8 Hz, 1H), 7.42 (d, J = 7.8 Hz, 2H), 4.74 (hept, J = 6.7 Hz, 1H), 2.37 – 2.25 (m, 2H), 1.61 (d, J = 6.7 Hz, 6H), 1.22 – 1.12 (m, 13H); **^{13}C NMR** (126 MHz, CD_3CN, 298K): 146.67, 136.29, 132.79, 131.40, 126.24, 125.55, 122.47, 54.86, 29.38, 24.29, 24.14, 22.85.

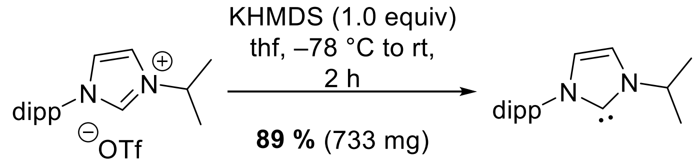

Imidazole-carbene **Idipp^{iPr}**: Imidazolium salt **S6** ($C_{19}H_{27}F_3N_2O_3S$, 420.49 g/mol, 1.28 g, 3.04 mmol, 1 equiv) was dissolved in thf (25 mL) and cooled to –78 °C. KHMDS ($C_6H_{18}KNSi_2$, 199.49 g/mol, 607.25 mg, 3.04 mmol, 1 equiv) was added and the reaction was stirred for 25 minutes at –78 °C and additional 1 hour and 35 minutes at ambient temperature. After that the solvent was evaporated and the crude product was extracted with Et_2O (4 x 20 mL) employing a filtration cannula. The yellow solution was dried to yield imidazole-carbene **Idipp^{iPr}** ($C_{18}H_{26}N_2$, 270.42 g/mol, 733.4 mg, 2.71 mmol, 89.1 %) as an off-white solid.

m.p. 123 °C; **^{1}H-NMR** (600 MHz, C_6D_6, 298K): δ [ppm] = 7.27 (d, *J* = 7.7 Hz, 1H, dipp-^{para}C*H*), 7.16 (d, *J* = 7.7 Hz, 2H, ^{meta}dipp-C*H*), 6.60 – 6.58 (m, 2H, NC*H*C*H*N), 4.46 (hept, *J* = 6.7 Hz, 1H, N-C*H*(CH_3)$_2$), 2.87 (hept, *J* = 6.9 Hz, 2H, dipp-C*H*(CH_3)$_2$), 1.30 (d, *J* = 6.7 Hz, 6H, N-CH(C*H*$_3$)$_2$), 1.24 (d, *J* = 6.9 Hz, 6H, dipp-CH(C*H*$_3$)$_2$), 1.12 (d, *J* = 7.0 Hz, 6H, dipp-CH(C*H*$_3$)$_2$); **^{13}C-NMR** (151 MHz, C_6D_6, 298K): δ [ppm] = 216.4 (carbene-*C*), 146.4 (C_q), 139.5 (C_q), 128.8 (dipp-^{para}CH), 123.6 (dipp-^{meta}CH), 121.5 (N*C*HCHN), 115.7 (NCH*C*HN), 52.2 (N-C*H*(CH_3)$_2$), 28.6 (dipp-C*H*(CH_3)$_2$), 24.6 (dipp-CH(C*H*$_3$)$_2$), 24.3(N-CH(C*H*$_3$)$_2$), 24.1 (dipp-CH(C*H*$_3$)$_2$); **IR** [cm^{-1}]: ṽ = 3152, 3048, 2961, 2867, 1589, 1472, 1457, 1399, 1362, 1310, 1260, 1246, 1230, 1213, 1119, 1106, 1070, 1058, 984, 942, 880, 808, 764, 749, 653, 575, 512, 455; **HR-MS-ESI(+)** calc. for $C_{18}H_{27}N_2^+$: $[M+H]^+$ 271.2169; found 271.2172.

Synthesis of neutral **2e**

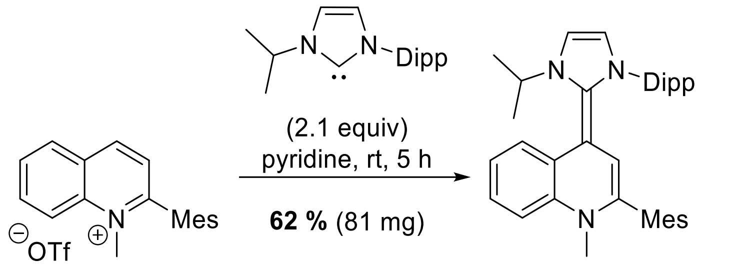

N-methyl-2-mesitylene-quinolinium salt **1d** ($C_{20}H_{20}F_3NO_3S$, 411.44 g/mol, 100 mg, 243.05 µmol, 1 equiv) and imdazole-carbene **Idipp^{iPr}** ($C_{18}H_{26}N_2$, 270.42 g/mol, 138.02 mg, 510.4 µmol, 2.1 equiv) were dissolved in pyridine (8 mL). The mixture turned deep red immediately and was stirred at ambient temperature for 5 hours. Then the solvents were evaporated and the resulting red solid was extracted with pentane. The red solution was dried and washed with HMDSO (2 x 3 mL). The residual solid was dried to yield hybrid-system **2e** ($C_{37}H_{45}N_3$, 531.79 g/mol, 80.6 mg, 151.56 µmol, 62.4 %) as a red solid.

Note: It was necessary to cool the sample to –80 °C to obtain sharp signals in the NMR-spectra. Otherwise only broad signals were observed.

m.p. 162 °C (decomp.); **^{1}H-NMR** (500 MHz, thf-d_8, 193K): δ [ppm] = 7.23 – 7.16 (m, 2H, quin-CH), 7.04 – 7.00 (m, 1H, quin-CH), 6.89 (d, *J* = 7.5 Hz, 1H- dipp-CH), 6.73 (s, 1H- Mes-CH), 6.65 (s, 1H, Mes-CH), 6.49 (s, 1H, NCHC*H*N), 6.47 (s, 1H, NC*H*CHN), 6.45 (d, *J* = 7.5 Hz, 1H, dipp-CH), 6.40 (t, *J* = 7.5 Hz, 1H, dipp-CH), 6.02 (d, *J* = 7.8 Hz, 1H, quin-CH), 4.09 (hept, *J* = 6.6 Hz, 1H, N-C*H*(CH_3)$_2$), 4.02 (s, 1H, pyr-CH), 3.16 (hept, *J* = 7.0 Hz, 1H, dipp-C*H*(CH_3)$_2$), 2.72 (hept, *J* = 6.9 Hz, 1H, dipp-C*H*(CH_3)$_2$), 2.21 (s, 3H-N-C*H*$_3$), 2.17 (s, 6H, Mes-^{ortho}C*H*$_3$), 1.74 (s, 3H, Mes-^{para}C*H*$_3$ (under thf-d_8), 1.33 (d, *J* = 6.8 Hz, 3H, N-CH(C*H*$_3$)$_2$), 1.30 (d, *J* = 6.8 Hz, 3H, dipp-CH(C*H*$_3$)$_2$), 1.16 (d, *J* = 6.2 Hz, 3H, N-C*H*(CH_3)$_2$), 1.12 (d, *J* = 6.9 Hz, 3H, dipp-CH(C*H*$_3$)$_2$), 1.08 (d, *J* = 6.9

Hz, 3H, dipp-CH(CH_3)$_2$), 0.82 (d, J = 6.8 Hz, 3H, dipp-CH(CH_3)$_2$); **^{13}C-NMR** (126 MHz, thf-d$_8$, 193K): δ [ppm] = 148.0 (dipp-C$_q$), 146.4 (dipp-C$_q$), 143.3 (C$_q$), 138.4 (Mes-C$_q$), 137.8 (Mes-C$_q$), 136.9 (C$_q$), 136.6 (Mes-C$_q$), 135.9 (Mes-C$_q$), 132.5 (C$_q$), 129.9 (C$_q$), 129.1 (quin-CH), 128.5 (Mes-CH), 128.3 (Mes-CH), 125.3 (quin-CH), 124.6 (quin-CH), 123.4 (dipp-CH), 122.6 (N*C*HCHN), 120.4 (dipp-CH), 119.9 (dipp-CH), 113.1 (N*C*HCHN), 110.5 (quin-CH), 106.0 (pyr-CH), 82.1 (C$_q$), 49.4 (N-*C*H(CH$_3$)$_2$), 33.3 (N-*C*H$_3$), 29.4 (dipp-*C*H(CH$_3$)$_2$), 28.8 (dipp-*C*H(CH$_3$)$_2$), 25.4 (dipp-CH(*C*H$_3$)$_2$, under thf-d$_8$), 25.4 (dipp-CH(*C*H$_3$)$_2$, under thf-d$_8$) 24.3 (dipp-CH(*C*H$_3$)$_2$), 22.6 (dipp-CH(*C*H$_3$)$_2$), 22.6 (N-CH(*C*H$_3$)$_2$), 21.4 (Mes-CH$_3$), 20.7 (Mes-CH$_3$), 20.3 (Mes-CH$_3$), 19.1 (N-CH(*C*H$_3$)$_2$); **IR** [cm^{-1}]: ṽ = 3112, 2962, 2864, 1598, 1545, 1484, 1465, 1402, 1387, 1374, 1333, 1295, 1271, 1255, 1230, 1198, 1179, 1123, 1087, 1043, 1016, 948, 848, 832, 809, 771, 748, 703, 692, 674, 661, 603, 524, 482, 423; **HR-MS-ESI(+)** calc. for $C_{37}H_{45}N_3^{2+}$: 265.6801 [M]$^{2+}$; found 265.6796; **UV-vis:** λ_{max}: 328 nm (ε = 11880 cm^{-1} M^{-1}), 484 nm (ε = 44914 cm^{-1} M^{-1}).

Synthesis of radical cation **3e**

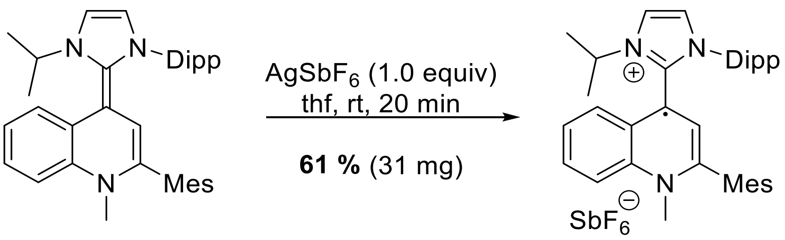

$AgSbF_6$ ($AgSbF_6$, 343.62 g/mol, 23.0 mg, 66.94 µmol, 1.0 equiv) was dissolved in thf (3 mL) and added dropwise to a solution of hybrid system **2e** ($C_{37}H_{45}N_3$, 531.79 g/mol, 35.6 mg, 66.94 µmol, 1.0 equiv) in thf (3 mL). The now dark solution was stirred for 20 minutes at room temperature. After that the solution was filtrated over a celite plug (1.5 cm). Then the solvent was evaporated until 1 mL of solvent was left. Pentane (17 mL) was added to precipitate a dark red solid. The solvent was decanted off and the remaining solid was washed with pentane (4 mL). Drying yielded radical cation **3e** ($C_{37}H_{45}F_6N_3Sb$, 767.54 g/mol, 31.2 mg, 40.65 µmol, 60.7 %) as a dark red solid.

m.p. 85 °C; **EPR:** g = 2.0028 (1xN 13.1510; 1xN 7.6787; 1xN 5.6376; 1xH 5.5131; 1xH 1.8653; 1xH 5.6380; 1xH 2.0472; 1xH 1.5928; 3xH 12.5349); **IR** [cm^{-1}]: ṽ = 2966, 2928, 2871, 1610, 1559, 1544, 1468, 1379, 1333, 1288, 1205, 1134, 1125, 1086, 1057, 854, 808, 754, 723, 652, 544, 489, 460; **HR-MS-ESI(+)** calc. for $C_{37}H_{45}N_3^{2+}$: 265.6801 [M]$^{2+}$; found 265.6795; **UV-vis:** λ_{max}: 339 nm (ε = 8860 cm^{-1} M^{-1}), 398 nm (ε = 4106 cm^{-1} M^{-1}), λ_{max}: 542 nm (ε = 2366 cm^{-1} M^{-1}).

Synthesis of dication **4e**

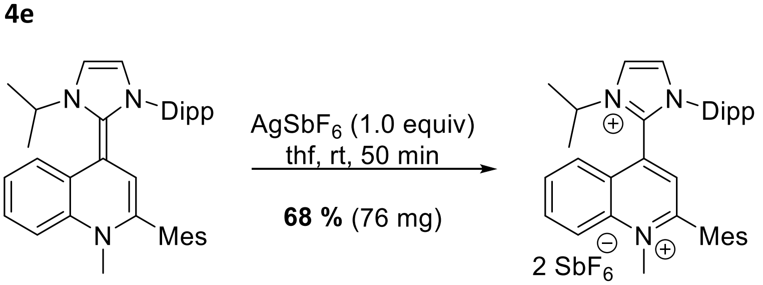

$AgSbF_6$ ($AgSbF_6$, 343.62 g/mol, 75.99 mg, 221.14 µmol, 2.0 equiv) was dissolved in thf (4 mL) and added dropwise to a solution of hybrid system **2e** ($C_{37}H_{45}N_3$, 531.79 g/mol, 58.8 mg, 110.57 µmol, 1.0 equiv) in thf (4 mL). The dark suspension was stirred for 50 minutes and then filtrated over a celite plug (1.5 cm). Then the solvent was evaporated until ca. 1 mL of solvent was left. Et_2O (20 mL) was added to form a

yellow precipitate. The solvent was decanted off and the remaining solid was washed with Et_2O (4 x 5 mL). Drying under reduced pressure yielded dication **4e** ($C_{37}H_{45}F_{12}N_3Sb_2$, 1003.29 g/mol, 75.5 mg, 75.25 µmol, 68 %) as a pale green-yellow solid.

m.p. 221 °C (decomp.); **^{1}H-NMR** (600 MHz, CD_3CN, 298K): δ [ppm] = 8.61 (d, *J* = 9.0 Hz, 1H, quin-CH), 8.47 – 8.40 (m, 1H, quin-CH), 8.25 (d, *J* = 1.8 Hz, 1H, NC*H*CHN), 8.24 – 8.16 (m, 2H, quin-CH), 8.07 (d, *J* = 2.1 Hz, 1H, NC*H*CHN), 7.61 (s, 1H, pyr-CH), 7.48 (t, *J* = 7.8 Hz, 1H, dipp-CH), 7.37 (dd, *J* = 7.9, 1.4 Hz, 1H, dipp-CH), 7.17 (s, 1H, Mes-CH), 7.15 (dd, *J* = 7.8, 1.4 Hz, 1H, dipp-CH), 7.08 (s, 1H, Mes-CH), 4.40 (hept, *J* = 6.7 Hz, 1H, N-C*H*(CH_3)$_2$), 4.32 (s, 3H, N-C*H*$_3$), 2.52 (hept, *J* = 6.7 Hz, 1H, dipp-C*H*(CH_3)$_2$), 2.38 (s, 3H, Mes-CH_3), 2.31 (hept, *J* = 6.7 Hz, 1H, dipp-C*H*(CH_3)$_2$), 2.02 (s, 3H, Mes-CH_3), 1.77 (d, *J* = 6.7 Hz, 3H, N-CH(C*H*$_3$)$_2$), 1.47 (d, *J* = 6.5 Hz, 3H, N-CH(C*H*$_3$)$_2$), 1.27 (s, 3H, Mes-CH_3), 1.17 (d, *J* = 6.7 Hz, 3H, dipp-CH(C*H*$_3$)$_2$), 1.04 (d, *J* = 6.7 Hz, 3H, dipp-CH(C*H*$_3$)$_2$), 0.94 (d, *J* = 6.7 Hz, 3H, dipp-CH(C*H*$_3$)$_2$), 0.33 (d, *J* = 6.8 Hz, 3H, dipp-CH(C*H*$_3$)$_2$); **^{13}C NMR** (151 MHz, CD_3CN, 298K): δ [ppm] = 161.2 (C_q), 146.7 (C_q), 146.6 (C_q), 143.5 (C_q), 142.1 (C_q), 138.4 (quin-CH), 138.1 (C_q), 136.7 (C_q), 136.5 (C_q), 136.1 (C_q), 133.9 (quin-CH), 133.5 (dipp-CH), 130.2 (Mes-CH), 130.1 (Mes-CH), 129.7 (C_q), 129.2 (pyr-CH), 129.2 (C_q), 129.0 (N*C*HCHN), 128.1 (C_q), 127.3 (quin-CH), 126.4 (dipp-CH), 126.2 (dipp-CH), 123.2 (N*C*HCHN), 122.6 (quin-CH), 55.8 (N-*C*H(CH_3)$_2$), 42.7 (N-CH_3), 29.7 (dipp-*C*H(CH_3)$_2$), 29.1 (dipp-*C*H(CH_3)$_2$), 26.5 (dipp-CH(*C*H$_3$)$_2$), 25.9 (dipp-CH(*C*H$_3$)$_2$), 24.2 (N-CH(*C*H$_3$)$_2$), 22.2 (dipp-CH(*C*H$_3$)$_2$), 21.9 (dipp-CH(*C*H$_3$)$_2$), 21.4 (N-CH(*C*H$_3$)$_2$), 21.3 (Mes-CH_3), 20.1 (Mes-CH_3), 19.4 (Mes-CH_3); **IR** [cm^{-1}]: ṽ = 2970, 1608, 1565, 1522, 1479, 1367, 1350, 1300, 1250, 1207, 1122, 1057, 891, 855, 808, 760, 730, 652, 546; **HR-MS-ESI(+)** calc. for $C_{37}H_{45}N_3^{2+}$: 265.6801 $[M]^{2+}$; found 265.6796; **UV-vis:** λ_{max}: 334 nm (ε = 10567 cm^{-1} M^{-1}).

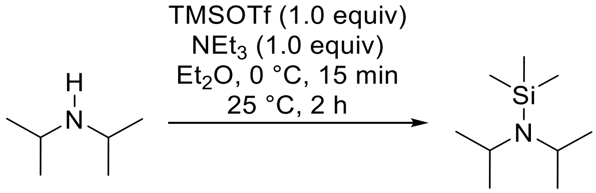

Silylamine **S7**: Diisopropylamine ($C_6H_{15}N$, 101.19 g/mol, 7.08 g, 0.72 g/mL, 9.88 mL, 70.00 mmol, 1.0 equiv) and triethylamine ($C_6H_{15}N$, 101.19 g/mol, 7.08 g, 0.73 g/mL, 9.76 mL, 70.00 mmol, 1.0 equiv) were added to the Schlenk flask containing diethylether (20 mL). The clear solution was cooled to 0 °C. Trimethylsilyl trifluoromethanesulfonate ($C_4H_9F_3O_3SSi$, 222.25 g/mol, 15.56 g, 1.23 g/mL, 12.67 mL, 70.00 mmol, 1.0 equiv) was added dropwise. A white precipitation formed, which was dissolved again after a view minutes. After ca. 15 min the reaction mixture formed two phases. The reaction was warmed to room temperature (20 °C) and stirred for 2 h. The upper organic phase was separated using a filter cannula. Drying of the solvent under reduced pressure gave a clear solution. Bulk to bulk distillation under static vacuum (approx. 10^{-2} to 10^{-3} mbar) gave N-(trimethylsilyl)diisopropylamine **S7** ($C_9H_{23}NSi$, 173.38 g/mol) as a colourless liquid. The yield has not been determined and the compound directly used. The spectroscopic data are in good agreement with previous reports.[7]

^{1}H-NMR (501 MHz, C_6D_6, 298 K) δ [ppm] = 3.12 (hept, *J* = 6.77 Hz, 2H), 1.04 (d, *J* = 6.79 Hz, 12H), 0.17 (s, 9H); **^{13}C-NMR** (151 MHz, C_6D_6, 298 K): δ [ppm] = 45.55, 24.62, 2.74.

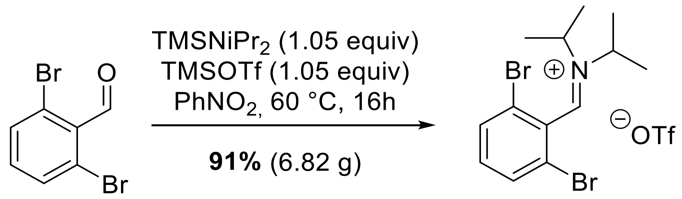

Iminium salt **S8**: 2,6-dibromobenzaldehyde ($C_7H_4Br_2O$, 263.92 g/mol, 3.96 g, 15.00 mmol, 1.0 equiv) was dissolved in anhydrous nitrobenzene (10 mL). Then *N*-(trimethylsilyl)diisopropylamine **S7** ($C_9H_{23}NSi$, 173.38 g/mol, 2.73 g, 0.79 g/mL, 3.47 mL, 15.75 mmol, 1.05 equiv) was added to the yellow solution. Trimethylsilyl trifluoromethanesulfonate ($C_4H_9F_3O_3SSi$, 222.25 g/mol, 3.50 g, 1.23 g/mL, 2.85 mL, 15.75 mmol, 1.05 equiv) was added dropwise. After this addition gas development was observed. The reaction was heated at 60 °C and stirred for 16 h. The reaction was cooled to room temperature. Et_2O (20 mL) was added and the resulting precipitate was washed with Et_2O (2 x 20 mL). The colourless solid was separated using a filter cannula. The solid was washed again with thf (3 x 10 mL) and Et_2O (1 x 20 mL, 2 x 10 mL) using a filter cannula. Drying under reduced pressure gave the iminium salt **S8** ($C_{14}H_{18}Br_2F_3NO_3S$, 497.16 g/mol, 6.82 g, 13.65 mmol, 91%) as a white solid. The spectroscopic data are in good agreement with previous reports.[7]

^{1}H-NMR (501 MHz, CD_3CN) δ [ppm] = 9.25 (s, 1H), 7.84 (d, *J* = 8.18 Hz, 2H), 7.51 (t, *J* = 8.19 Hz, 1H), 4.65 (hept, *J* = 6.69 Hz, 1H), 4.43 (hept, *J* = 6.5 Hz, 1H), 1.69 (d, *J* = 6.69 Hz, 6H), 1.48 (d, *J* = 6.59 Hz, 6H); **^{13}C-NMR** (126 MHz, CD_3CN) δ [ppm] = 172.61, 135.99, 133.55, 130.05, 120.61, 62.27, 57.73, 24.02, 19.31.

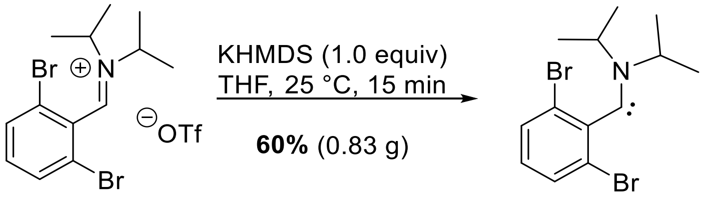

Free carbene ^{Br}**AAArC**: A solution of KHMDS ($C_6H_{18}KNSi_2$, 199.49 g/mol, 0.80 g, 4.00 mmol, 1.00 equiv) in thf (1.5 mL) was added to a solution of the iminium salt **S8** ($C_{14}H_{18}Br_2F_3NO_3S$, 497.16 g/mol, 1.99 g, 4.0 mmol, 1.0 equiv) in thf (1.5 mL). The colour of the reaction mixture changed to brown. After 15 min of stirring the solvent was removed under reduced pressure. The brown solid was extracted with *n*-pentane (60 mL). The solvent was reduced to 40 mL under reduced pressure. The brown solution was cooled to –78 °C for crystallization. The supernatant was separated and repetition of the crystallization and drying under reduced pressure gave the free carbene ^{Br}**AAArC** ($C_{13}H_{17}Br_2N$, 347.09 g/mol, 0.83 g, 2.39 mmol, 60%) as a yellow solid. The spectroscopic data are in good agreement with previous reports.[7]

^{1}H-NMR (501 MHz, C_6D_6, 298 K) δ [ppm] = 7.20 (d, *J* = 7.92 Hz, 2H), 6.16 (t, *J*= 7.92 Hz, 1H), 3.64 (m, 2H), 1.43 (d, *J* = 6.40 Hz, 6H), 0.88 (d, *J* = 6.69 Hz, 6H); **^{13}C-NMR** (126 MHz, C_6D_6, 298 K): δ [ppm] = 299.91 (carbene-C), 148.25, 131.41, 123.67, 109.89, 60.20, 51.80, 24.92, 19.62.

Synthesis of neutral **2f**

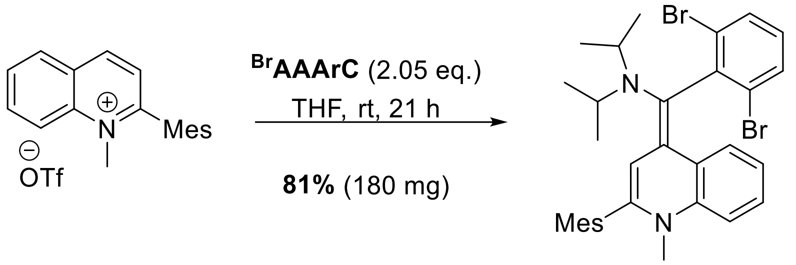

A solution of the quinolinium salt **1d** ($C_{20}H_{20}F_3NO_3S$, 411.44 g/mol, 150 mg, 0.37 mmol, 1.00 equiv) was dissolved in thf (8.0 mL) and added to a solution of the carbene ^{Br}**AAArC** ($C_{13}H_{17}Br_2N$, 347.09 g/mol, 259 mg, 0.75 mmol, 2.05 equiv) in thf (8.0 mL). The reaction mixture changed its color to deep red. After 21 h of stirring, the solvent was removed under reduced pressure. The red solid was extracted with Et_2O (4 x 10 mL). Drying of the red solution under reduced pressure gave the hybrid system **2f** ($C_{32}H_{36}Br_2N_2$, 608.46 g/mol, 180 mg, 0.30 mmol, 81%) as a red solid.

Single crystals suitable for X-ray diffraction could be obtained from a saturated Et_2O solution at –40 °C.

m.p. 165°C; ^{1}**H-NMR** (501 MHz, C_6D_6, 298 K) δ [ppm] = 7.29 (d, *J* = 7.93 Hz, 2H, Ar-C*H*), 7.24 (dd, *J* = 1.44 Hz; 8.25 Hz, 1H, Ar-C*H*), 6.91 (m, 1H, Ar-C*H*), 6.79 (d, *J* = 1.17 Hz, 2H, Ar-C*H*), 6.56 (m, 2H, Ar-C*H*), 6.26 (t, *J* = 7.91 Hz, 1H, Ar-C*H*), 6.09 (s, 1H, quinolinium-C*H*), 3.52 (hept, *J* = 6.64 Hz, 2H, C*H*$_2$), 2.56 (s, 3H, N-C*H*$_3$), 2.31 (s, 6H, mesityl-C*H*$_3$), 2.13 (s, 3H, mesityl-C*H*$_3$), 1.30 (d, *J* = 6.65 Hz, 12H, $CH_2(CH_3)_2$); ^{13}**C-NMR** (126 MHz, C_6D_6, 298 K): δ [ppm] = 143.87 (C_q), 142.71 (C_q), 138.46 (C_q), 137.70 (C_q), 135.04 (C_q), 133.58 (Ar-CH), 130.13 (C_q), 129.15 (C_q), 128.70 (C_q), 125.88 (C_q), 121.50 (Ar-*C*H), 112.92 (Ar-*C*H), 109.27 (quinolinium-*C*H), 51.58 ($CH_2(CH_3)_2$), 33.19 (N-*C*H$_3$), 23.54 (*C*H$_2$), 21.15 (Mesityl-*C*H$_3$), 20.22 (Mesityl-*C*H$_3$). notice: two aromatic ^{13}C-signals are overlayed by the solvent (according to 2D-NMR) ; **IR**: $\tilde{\nu}$ [cm^{-1}] = 2962, 2933, 2918, 2852, 1627, 1594, 1527, 1538, 1529, 1468, 1450, 1430, 1411, 1374, 1360, 1314, 1297, 1256, 1190, 1154, 1133, 1112, 1094, 1044, 1007, 994, 929, 856, 793, 768, 756, 748, 728, 715, 672, 655, 637, 606, 588, 533, 485, 448, 437, 410; **HR-MS-ESI(+)** calc. for $C_{32}H_{36}{}^{79}Br_2N_2^+$: 606.1245 [M]$^+$; found 606.1235, 283.0372; **UV-vis** λ_{max}: 462 nm (ε = 15492 cm^{-1}M^{-1}).

Synthesis of radical cation **3f**

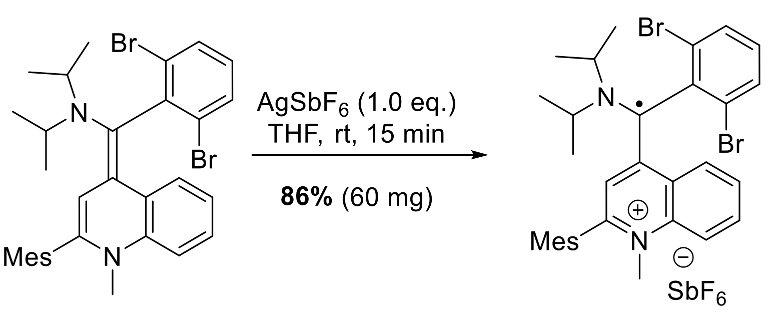

Silver hexafluoroantimonate ($AgSbF_6$, 343.62 g/mol, 28 mg, 0.08 mmol, 1.00 equiv) was dissolved in thf (7 mL) and added dropwise to a solution of switch **2f** ($C_{32}H_{36}Br_2N_2$, 608.46 g/mol, 50 mg, 0.08 mmol, 1.00 equiv) in thf (5 mL). The reaction mixture changed its color to deep black. After 15 min of stirring, the reaction mixture was filtered over Celite. The solvent was evaporated until 1 mL of the dark solution was left. After addition of Et_2O (10 mL) a black solid precipitated. The solvent was decanted off. Drying of the

black solid under reduced pressure gave radical cation **3f** ($C_{32}H_{36}Br_2F_6N_2Sb$, 844.21 g/mol, 60 mg, 0.07 mmol, 86%) as a black solid.

m.p. 190°C; **EPR**: g = 1.9847 (1xN: 7.7337 MHz, 1xN: 14.9389 MHz; 1xH: 0.6539 MHz; 1xH: 0.6208 MHz; 3xH: 10.0005 MHz; 1xH: 13.0936 MHz; 1xH: 10.3416 MHz); **IR**: $\tilde{\nu}$ [cm^{-1}] = 1595, 1544, 1484, 1448, 1397, 1371, 1313, 1293, 1253, 1166, 1128, 1093, 1081, 905, 851, 785, 767, 725, 653, 608, 516, 491, 452; **HR-MS-ESI(+)** calc. for $C_{32}H_{36}{}^{79}Br_2N_2^{+}$: 606.1245 $[M]^{+}$; found 606.1250; **UV-vis** λ_{max}: 508 nm (ε = 7216 $cm^{-1}M^{-1}$), 702 nm (ε = 47108 $cm^{-1}M^{-1}$).

Synthesis of dication **4f**

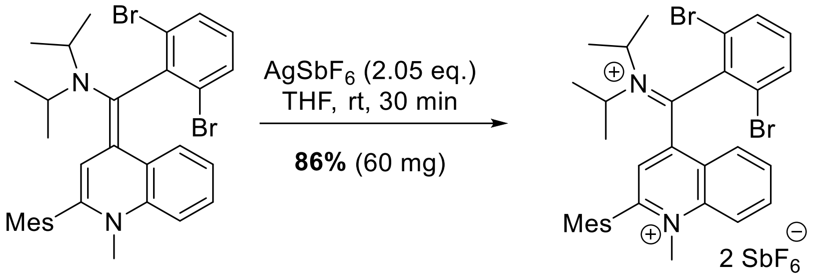

Silver hexafluoroantimonate ($AgSbF_6$, 343.62 g/mol, 58 mg, 0.17 mmol, 2.05 euiv.) was dissolved in thf (2.0 mL) and dropwise added to a solution of hybrid system **2f** ($C_{32}H_{36}Br_2N_2$, 608.46 g/mol, 50 mg, 0.08 mmol, 1.00 equiv) in thf (5.0 mL). The reaction mixture changed its color from deep black to a light green color. After 30 min of stirring, the reaction mixture was filtered over Celite. The solvent was evaporated until 2 mL of the yellow solution was left. After addition of Et_2O (18 mL) a yellow precipitate was formed. The solvent was decanted off. The yellow solid was dried to give dication **4f** ($C_{32}H_{36}Br_2F_6N_2Sb$, 844.21 g/mol, 60 mg, 0.07 mmol, 86%) as a yellow solid.

m.p. 215°C; **^{1}H-NMR** (501 MHz, CD_3CN, 298 K) δ [ppm] = 8.65 (d, *J* = 9.00 Hz, 1H, Ar-C*H*), 8.47 (m, 2H, Ar-C*H*), 8.35 (m, 1H, Ar-C*H*), 8.09 (s, 1H, quinolinium-C*H*), 7.96 (dd, *J* = 8.22 Hz; 0.94, 1H, Ar-C*H*), 7.81 (dd, *J* = 8.21; 0.96 Hz, 1H, Ar-C*H*), 7.50 (t, *J* = 8.19 Hz, 1H, Ar-C*H*), 7.20 (s, 1H, Ar-C*H*), 7.13 (s, 1H, Ar-C*H*), 5.16 (hept, *J* = 6.90 Hz,1H. CH_2), 5.00 (hept, *J* = 6.88 Hz, 1H, CH_2), 4.38 (s, 3H, N-CH_3), 2.39 (s, 3H, Mesityl-CH_3), 2.06 (s, 3H, Mesityl-CH_3), 1.91 (m, 6H, CH_2(CH_3)$_2$), 1.80 (d, *J* = 5.49 Hz ,3H, Mesityl-CH_3), 1.77 (s, 3H, CH_2(CH_3)$_2$), 1.61 (d, *J* = 6.88 Hz, 3H, CH_2(CH_3)$_2$). **^{13}C-NMR** (126 MHz, CD_3CN, 298 K): δ [ppm] = 176.67 (C_q), 161.45 (C_q), 144.57 (C_q), 143.71 (C_q), 142.62 (Ar-*C*H), 138.21 (Ar-*C*H), 137.39 (C_q), 137.02 (C_q), 136.62 (C_q), 136.05 (Ar-*C*H), 135.62 (Ar-*C*H), 133.49 (Ar-*C*H), 130.44 (Ar-*C*H), 130.42 (C_q), 129.39 (Ar-*C*H), 128.49 (Ar-*C*H), 126.53 (C_q), 125.70 (Ar-*C*H), 122.41 (Ar-*C*H), 122.14 (C_q), 121.08 (Ar-*C*H), 69.20 (*C*H_2), 68.24(*C*H_2), 66.22, 43.10 (N-CH_3), 26.21 (C_q), 24.81 (*C*H_2), 23.32 (*C*H_2), 21.36 (CH_2(CH_3)$_2$), 21.27 (CH_2(CH_3)$_2$), 20.29 (CH_2(CH_3)$_2$), 20.05 (Mesityl-*C*H_3), 19.72 (Mesityl-CH_3), 15.58 (CH_2(CH_3)$_2$); **IR**: $\tilde{\nu}$ [cm^{-1}] = 1611, 1569, 1516, 1468, 1428, 1380, 1356, 1298, 1266, 1244, 1198. 1166, 1141, 1128, 1109, 1096, 1063, 1016, 891, 854, 843, 770, 750, 721, 653, 569, 509, 491, 454, 416; **HR-MS-ESI(+)** $C_{32}H_{36}{}^{79}Br_2N_2^{2+}$: 303.0617 $[M]^{+}$; found 303.0617; **UV-vis** λ_{max}: 345 nm (ε = 5178 $cm^{-1}M^{-1}$).

Synthesis of neutral **2g**

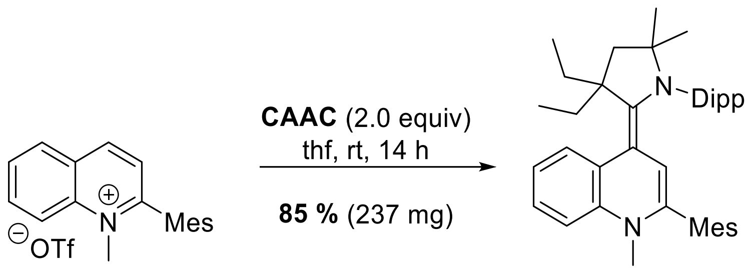

N-methyl-2-mesitylene-quinolinium salt **1d** ($C_{20}H_{20}F_3NO_3S$, 411.44 g/mol, 200 mg, 486.1 µmol, 1 equiv) and **CAAC** ($C_{22}H_{35}N$, 313.53 g/mol, 304.81 mg, 972.2 µmol, 2.0 equiv) were dissolved in thf (15 mL) and stirred over night (14 h). The solvent was then evaporated under reduced pressure and the residual solid was extracted with pentane (4 x 20 mL). Crystallisation from a saturated pentane solution at –40 °C yielded switch **2g** ($C_{41}H_{54}N_2$, 574.9 g/mol, 237 mg, 412.25 µmol, 85 %) as a yellow solid. The *E*:*Z* ratio after the reaction was determined by ^{1}H NMR as 65:35.

Single crystals suitable for X-ray diffraction could be obtained by cooling a saturated pentane solution to –40 °C.

m.p. 88 °C; **^{1}H-NMR** (500 MHz, C_6D_6, 298K): δ [ppm] = 7.46 (dd, *J* = 7.6, 1.6 Hz, 1H, Ar-CH), 7.11 (td, *J* = 7.7, 1.6 Hz, 1H, Ar-CH), 7.06 – 7.02 (m, 3H, Ar-CH), 7.02 – 6.96 (m, 1H, Ar-CH), 6.66 (s, 2H, Mes-CH), 6.55 – 6.51 (m, 1H, Ar-CH), 4.87 (s, 1H, pyr-CH), 3.64 (hept, *J* = 6.7 Hz, 2H, CH_3CHCH_3), 2.49 (s, 3H, N-C*H*$_3$), 2.09 (s, 3H, ^{para}Mes-C*H*$_3$), 2.05 (s, 6H, ^{ortho}Mes-C*H*$_3$), 2.03 – 1.91 (m, 4H, C*H*$_2$CH$_3$), 1.85 (s, 2H, C*H*$_2$), 1.30 (d, *J* = 6.7 Hz, 6H, CH$_3$CHC*H*$_3$), 1.11 (s, 6H, C*H*$_3$), 1.06 (d, *J* = 6.6 Hz, 6H, CH$_3$CHC*H*$_3$), 0.99 (t, *J* = 7.3 Hz, 6H, CH$_2$C*H*$_3$); Due to the complex isomeric mixture it was not possible to clearly assign the signals of only one isomer, instead both are reported. **^{13}C NMR** (126 MHz, C_6D_6, 298K): δ [ppm] = 148.17, 148.05, 147.42, 147.01, 145.36, 144.88, 140.96, 139.30, 137.85, 137.53, 137.27, 137.08, 136.83, 135.27, 135.09, 134.56, 129.48, 128.46, 128.35, 128.16, 127.97, 126.83, 126.49, 126.31, 125.62, 125.22, 124.46, 123.33, 119.91, 119.31, 110.22, 109.15, 106.56, 105.97, 105.45, 102.89, 64.13, 63.16, 51.03, 49.65, 49.32, 45.10, 35.04, 33.60, 33.17, 33.05, 30.85, 29.58, 29.11, 28.50, 27.16, 25.81, 25.06, 23.86, 23.38, 21.43, 21.14, 20.54, 20.45, 10.82, 10.45; **IR** [cm^{-1}]: ṽ = 2964, 2868, 1552, 1467, 1433, 1362, 1284, 1246, 1192, 1166, 1097, 1045, 1009, 994, 930, 850, 812, 798, 740, 714, 680, 578, 493, 431; **HR-MS-ESI(+)** calc. for $C_{41}H_{54}N_2^{2+}$: $[M]^{2+}$ 287.2138; found 287.2134; **UV-vis:** λ_{max}: 279 nm (ε = 9275 cm^{-1} M^{-1}), 392 nm (ε = 9420 cm^{-1} M^{-1}).

Synthesis of radical **3g**

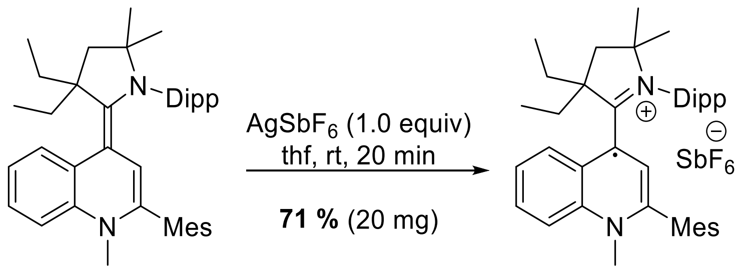

$AgSbF_6$ ($AgSbF_6$, 343.62 g/mol, 11.95 mg, 34.79 µmol, 1.0 equiv) was dissolved in thf (2 mL) and added dropwise to a solution of switch **2g** ($C_{41}H_{54}N_2$, 574.90 g/mol, 20 mg, 34.79 µmol, 1.0 equiv) in thf (4 mL). The now dark solution was stirred for 20 minutes at room temperature. After that the solution was filtrated over a celite plug (1.5 cm). Then the solvent was evaporated until ca. 1 mL of solvent was left. Pentane

(17 mL) was added to precipitate a black solid. The solvent was decanted off and the remaining solid was washed with pentane (3 mL). Evaporation of the solvent yielded radical cation **3g** ($C_{41}H_{54}F_6N_2Sb$, 810.65 g/mol, 20.1 mg, 24.79 µmol, 71 %) as a black solid.

Single crystals suitable for X-ray diffraction could be obtained by slow diffusion of Et_2O into a saturated THF solution.

m.p. 256 °C; **EPR:** g = 2.0036 (1xN 11.4398; 1xN 10.7994; 1xH 4.4854; 1xH 1.8865; 1xH 2.0414; 1xH 0.5494; 3xH 12.1624); **IR** [cm^{-1}]: ṽ = 2981, 1609, 1594, 1575, 1544, 1442, 1393, 1355, 1332, 1253, 1190, 1163, 1142, 1050, 853, 811, 765, 715, 655, 606, 568, 492; **HR-MS-ESI(+)** calc. for $C_{41}H_{54}N_2^{2+}$: $[M]^{2+}$ 287.2138; found 287.2134; **UV-vis:** λ_{max}: 337 nm (ε = 5779 cm^{-1} M^{-1}), 358 nm (ε = 6000 cm^{-1} M^{-1}), λ_{max}: 500 nm (ε = 3241 cm^{-1} M^{-1}), 671 nm (ε = 2707 cm^{-1} M^{-1}).

Synthesis of dication **4g**

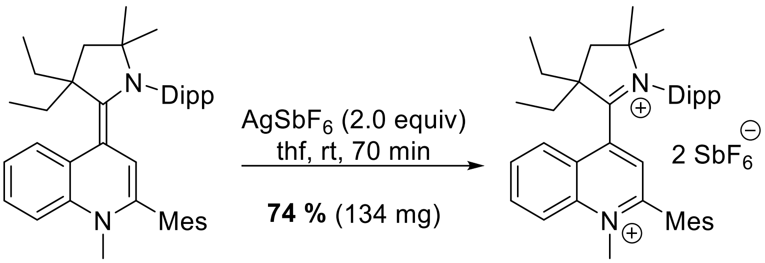

$AgSbF_6$ ($AgSbF_6$, 343.62 g/mol, 119.6 mg, 347.9 µmol, 2.0 equiv) was dissolved in thf (8 mL) and added dropwise to a solution of hybrid system **2g** ($C_{41}H_{54}N_2$, 574.90 g/mol, 100 mg, 173.94 µmol, 1.0 equiv) in thf (8 mL). The dark yellow solution was stirred for 70 minutes and then filtrated with a filtration cannula. The solvent was evaporated until ca. 1 mL of solvent was remaining. Et_2O (40 mL) was added to form a yellow precipitate. The solvent was filtered off and the remaining solid was washed with Et_2O (2 x 10 mL). Drying yielded dication **4g** ($C_{41}H_{54}F_{12}N_2Sb_2$, 1046.40 g/mol, 134 mg, 128.06 µmol, 74 %) as a pale-yellow solid.

m.p. 128 °C (decomp.); **^{1}H-NMR** (500 MHz, CD_3CN, 298K): δ [ppm] = 8.57 (dd, *J* = 9.2, 1.0 Hz, 1H, quinoline-CH), 8.50 (dd, *J* = 8.5, 1.3 Hz, 1H, quinoline-CH), 8.43 (ddd, *J* = 8.9, 7.2, 1.3 Hz, 1H, quinoline-CH), 8.27 (ddd, *J* = 8.2, 7.1, 1.0 Hz, 1H, quinoline-CH), 7.56 (s, 1H, pyridine-CH), 7.47 (t, *J* = 7.8 Hz, 1H, dipp-CH), 7.40 (dd, *J* = 7.9, 1.6 Hz, 1H, dipp-CH), 7.25 (dd, *J* = 7.8, 1.5 Hz, 1H, dipp-CH), 7.20 (s, 1H, Mes-CH), 7.08 (s, 1H, Mes-CH), 4.28 (s, 3H, N-C*H*$_3$), 2.84 – 2.74 (m, 3H, CH_3C*H*CH_3, CH_2), 2.70 (h, *J* = 6.2 Hz, 1H, CH_3C*H*CH_3), 2.38 (s, 3H, Mes-C*H*$_3$), 2.30 (dq, *J* = 15.1, 7.6 Hz, 1H, CH_3C*H*$_2$), 2.21 – 2.06 (m, 3H, CH_3C*H*$_2$), 2.05 (s, 3H, Mes-C*H*$_3$), 1.87 (s, 3H, C*H*$_3$), 1.66 (s, 3H, C*H*$_3$), 1.39 (s, 3H, Mes-C*H*$_3$), 1.34 (d, *J* = 6.2 Hz, 3H, CH_3CHC*H*$_3$), 1.28 (d, *J* = 6.3 Hz, 3H, CH_3CHC*H*$_3$), 0.95 (t, *J* = 7.3 Hz, 3H, CH_2C*H*$_3$), 0.89 (d, *J* = 6.4 Hz, 3H, CH_3CHC*H*$_3$), 0.68 (t, *J* = 7.4 Hz, 3H, CH_2C*H*$_3$), 0.08 (d, *J* = 6.4 Hz, 3H, CH_3CHC*H*$_3$); **^{13}C NMR** (126 MHz, CD_3CN, 298K): δ [ppm] = 197.7 (C_q), 160.8 (C_q), 145.9 (C_q), 145.6 (C_q), 143.7 (C_q), 143.3 (C_q), 141.4 (C_q), 138.6 (quinoline-CH), 136.8 (C_q), 136.5 (C_q), 133.4 (quinoline-CH), 133.2 (Dipp-CH), 130.5 (Mes-CH), 130.2 (Mes-CH), 129.3 (quinoline-CH), 128.6 (C_q), 128.4 (dipp-CH), 128.4 (dipp-CH), 125.7 (C_q), 124.0 (pyridine-CH), 122.8 (quinoline-CH), 88.4 (C_q), 62.8 (C_q), 45.6 (CH_2), 42.8 (N-CH_3), 32.5 (CH_3*C*H_2), 31.5 (CH_3), 30.0 (CH_3CHCH_3), 30.0 (CH_3*C*HCH_3), 29.9 (*C*H_2CH_3), 28.3 (CH_3), 27.1 (CH_3CH*C*H_3), 26.5 (CH_3CH*C*H_3), 26.1 (CH_3CH*C*H_3), 25.3 (CH_3CH*C*H_3), 21.3 (Mes-CH), 20.6 (Mes-CH), 19.5 (Mes-CH), 9.3 (*C*H_3CH_2), 9.2 (*C*H_3CH_2); **IR** [cm^{-1}]: ṽ = 2982, 2870, 1609, 1574, 1517, 1459, 1361, 1256, 1165, 1139, 1091, 929, 811, 766, 734, 654, 605, 594, 565, 491; **HR-MS-ESI(+)** calc. for $C_{41}H_{54}N_2^{2+}$: $[M]^{2+}$ 287.2138; found 287.2139; **UV-vis:** λ_{max}: 332 nm (ε = 7883 cm^{-1} M^{-1}).

Synthesis of neutral **2g**

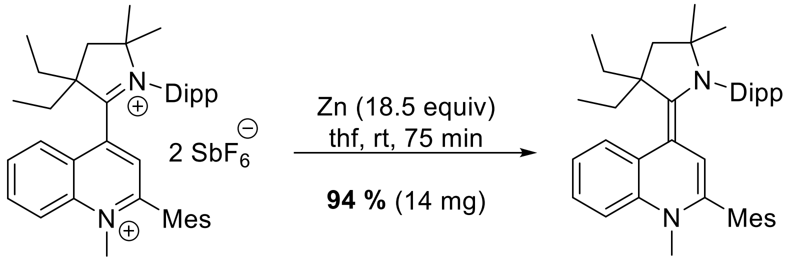

Dication **4g** ($C_{41}H_{54}F_{12}N_2Sb_2$, 1046.40 g/mol, 27 mg, 25.8 µmol, 1 equiv) was dissolved in thf (5 mL). Then a suspension of activated zinc dust (Zn, 65.38 g/mol, 31.2 mg, 477.35 µmol, 28.5 equiv) in thf (5 mL) was added. The suspension turned dark over 5 minutes. After 75 minutes the now yellow suspension was dried under reduced pressure. The residual solid was extracted with pentane over a short celite plug (1 cm) and then dried under reduced pressure to yield switch **2g** ($C_{41}H_{54}N_2$, 574.90 g/mol, 14 mg, 24.35 µmol, 94 %) as a yellow solid.

The analytical data matches those from the synthesis starting from quinolinium salt **1d** and free **CAAC**.

For the synthesis of activated zinc see [8].

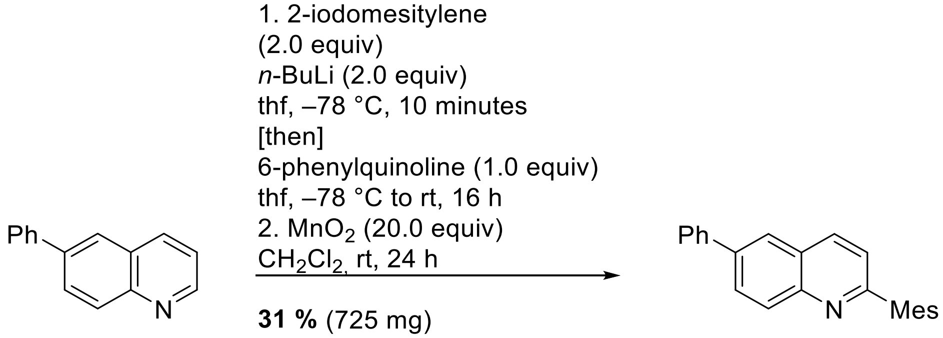

2-Mesityl-6-phenylquinolin **S9:** 2-Iodomesitylen ($C_9H_{11}I$, 246.09 g/mol, 3.60 g, 14.62 mmol, 2.0 equiv.) was dissolved in thf (20 mL) and cooled down to –78 °C. Then *n*-BuLi (C_4H_9Li, 64.06, 936.22 mg, 2.5 M, 5.85 mL, 14.62 mmol, 2.0 equiv.) was slowly added and the resulting suspension was stirred for 10 minutes at –78 °C. This suspension was then added to a solution of 6-phenylquinoline **S3** ($C_{15}H_{11}N$, 205.26 g/mol, 1.5 g, 7.31 mmol, 1.0 equiv.) in thf (20 mL) which was also cooled to –78 °C. The resulting solution was allowed to warm up to room temperature and stirred for 16 h. Then the solution was slowly poured over ice and then extracted using ethyl acetate (3 x 50 mL). The combined organic phases were dried using Na_2SO_4 and the residue solvent was removed under reduced pressure. The yellow oil was then dissolved in CH_2Cl_2 and activated MnO_2 (MnO_2, 86.94 g/mol, 12.71 g, 146.16 mmol, 20.0 equiv.) was added resulting in a brown suspension. After 24 h the suspension was filtrated over a silica plug (2.0 cm) using CH_2Cl_2 as a solvent. The crude product was then purified by column chromatography on silica (Cyclohexane/EtOAc: 50/1 to 10/1) to yield 2-mesityl-6-phenylquinoline **S9** ($C_{24}H_{21}N$, 323.44 g/mol, 725.0 mg, 2.24 mmol, 31 %) as a white solid.

m.p. 141 °C; **^{1}H-NMR** (500 MHz, $CDCl_3$, 298K): δ [ppm] = 8.28 (d, *J* = 8.2 Hz, 2H, Ar-CH), 8.07 (d, *J* = 2.1 Hz, 1H, Ar-CH), 8.02 (dd, *J* = 8.7, 2.1 Hz, 1H, Ar-CH), 7.80 – 7.72 (m, 2H, Ar-CH), 7.53 (t, *J* = 7.7 Hz, 2H, Ar-CH),

7.47 – 7.36 (m, 2H, Ar-CH), 7.00 (s, 2H, Mes-CH), 2.37 (s, 3H, Mes-^{para}CH$_3$), 2.09 (s, 6H, Mes-^{ortho}CH$_3$); **^{13}C-NMR** (126 MHz, CDCl$_3$, 298K): δ [ppm] = 160.7 (C$_q$), 147.5 (C$_q$), 140.6 (C$_q$), 139.4 (C$_q$), 137.9 (C$_q$), 136.7 (Ar-CH), 135.8 (C$_q$), 130.0 (Ar-CH), 129.5 (Ar-CH), 129.1 (Ar-CH), 128.6 (Mes-CH), 127.8 (Ar-CH), 127.6 (Ar-CH), 127.0 (C$_q$), 125.4 (Ar-CH), 123.5 (Ar-CH), 21.3 (Mes-^{para}CH$_3$), 20.3 (Mes-^{ortho}CH$_3$); **IR** [cm^{-1}]: ṽ = 3053, 2995, 2919, 2857, 2041, 1610, 1482, 1377, 1178, 1074, 1017, 885, 825, 760, 721, 647, 575, 515, 460; **HR-MS-ESI(+)** calc. for $C_{24}H_{22}N^+$: 324.1747; found 324.1744.

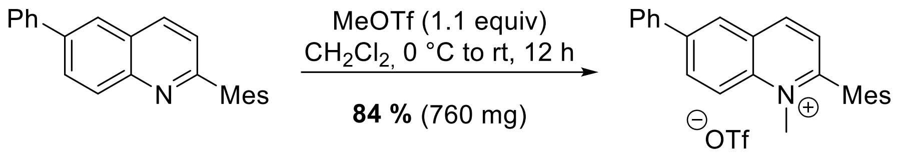

Quinoline salt **1h:** 2-mesityl-6-phenylquinoline **S9** ($C_{24}H_{21}N$, 323.44 g/mol, 600.0 mg, 1.86 mmol, 1.0 equiv) was dissolved in CH_2Cl_2 (20 mL) and cooled to 0°C. Then MeOTf ($C_2H_3F_3O_3S$, 164.1 g/mol, 334.85 mg, 1.5 g/mL, 223.24 µL, 2.04 mmol, 1.1 equiv) was added dropwise. The solution was stirred for 5 min at 0 °C, warmed up to room temperature and stirred overnight. Then the solvent was evaporated until 1-2 mL of the solvent were left over. Then Et_2O (30 mL) was added resulting in the formation of a white solid. The solvent was removed using a filter cannula and the residue solid was washed with Et_2O (3 x 10 mL). Drying of the solid yielded quinolinium salt **1h** ($C_{26}H_{24}F_3NO_3$, 487.54 g/mol, 760.0 mg, 1.56 mmol, 84 %) as a white solid powder.

m.p. 102 °C; **^{1}H-NMR** (500 MHz, CD$_3$CN, 298K): δ [ppm] = 9.15 (d, *J* = 8.4 Hz, 1H, Ar-CH), 8.66 (d, *J* = 2.1 Hz, 1H, Ar-CH), 8.61 – 8.53 (m, 2H, Ar-CH), 8.00 – 7.86 (m, 3H, Ar-CH), 7.61 (dd, *J* = 8.3, 6.8 Hz, 2H, Ar-CH), 7.56 – 7.52 (m, 1H, Ar-CH), 7.18 (s, 2H, Mes-CH), 4.29 (s, 3H, N-CH$_3$), 2.41 (s, 3H, Mes-^{para}CH$_3$), 2.01 (s, 6H, Mes-^{ortho}CH$_3$); **^{13}C-NMR** (126 MHz, CD$_3$CN, 298K): δ [ppm] = 160.8 (C$_q$), 148.1 (Ar-CH), 143.2 (C$_q$), 142.7 (C$_q$), 140.5 (C$_q$), 138.5 (C$_q$), 136.8 (C$_q$), 135.9 (Ar-CH), 130.8 (C$_q$), 130.7 (C$_q$), 130.5 (Ar-CH), 130.3 (Ar-CH), 130.1 (Mes-CH), 128.6 (Ar-CH), 128.5 (Ar-CH), 126.4 (Ar-CH), 121.4 (Ar-CH), 41.4 (N-CH$_3$), 21.3 (Mes-^{para}CH$_3$), 20.0 (Mes-^{ortho}CH$_3$); **IR** [cm^{-1}]: ṽ = 2917, 2462, 2077, 1999, 1584, 1506, 1372, 1347, 1256, 1221, 1146, 1028, 896, 851, 762, 697, 635, 571, 516; **HR-MS-ESI(+)** calc. for $C_{26}H_{24}N^+$: 338.1904 [M]$^+$; found 338.1898.

Synthesis of neutral **2h**

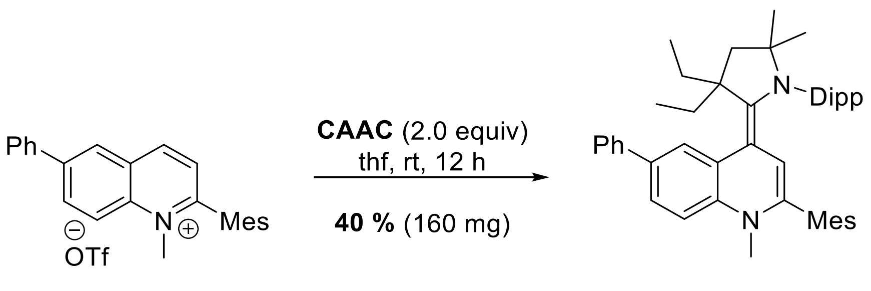

To a mixture **CAAC** ($C_{22}H_{35}N$, 313.53 g/mol, 385.53 mg, 1.23 mmol, 2.0 equiv) and quinolinium salt **1h** ($C_{26}H_{24}F_3NO_3S$, 487.54 g/mol, 300.0 mg, 615.34 µmol, 1.0 equiv) thf (20 mL) was added and the solution stirred for 12 h at room temperature to give a slightly red suspension. The solvent was removed under reduced pressure, Et_2O (15 mL) added and extracted under argon. The solvent was evaporated and the solid was then washed with pentane (3 x 5 mL) to furnish the switch **2h** ($C_{47}H_{58}N_2$, 651.0 g/mol, 160.0 mg, 246.14 µmol, 40 %) as orange solid. *E*:*Z* after the reaction = 93:7.

Single crystals suitable for X-ray diffraction could be obtained by slow diffusion of pentane into a saturated Et_2O solution.

Note: To obtain NMR spectra it proved necessary to add a small amount of KHMDS (0.5 mg - 1 mg) to the freshly prepared NMR sample in C_6D_6, otherwise only broad spectra were observed.

m.p. 194 °C; **^{1}H-NMR** (*E*-Isomer) (500 MHz, C_6D_6, 298K): δ [ppm] = 7.87 – 7.85 (m, 2H, Ar-CH), 7.83 (d, *J* = 2.3 Hz, 1H, Ar-CH), 7.45 (dd, *J* = 8.3, 2.2 Hz, 1H, Ar-CH), 7.35 (t, *J* = 7.7 Hz, 2H, Ar-CH), 7.17 (m, 1H, Ar-CH), 7.03 (m, 3H, Ar-CH), 6.68 (s, 2H, Mes-CH), 6.58 (d, *J* = 8.4 Hz, 1H, Ar-CH), 4.93 (s, 1H, pyridine-CH), 3.67 (p, *J* = 6.7 Hz, 2H, CH_3CHCH_3), 2.52 (s, 3H, N-CH_3), 2.19 – 2.12 (m, 2H, CH_2CH_3), 2.11 (s, 3H, Mes-$^{para}CH_3$), 2.07 (s, 6H, Mes-$^{orrtho}CH_3$), 2.00 – 1.96 (m, 2H, CH_2CH_3), 1.84 (s, 2H, CH_2), 1.28 (d, *J* = 6.7 Hz, 6H, CH_3CHCH_3), 1.11 (s, 6H, CH_3), 1.09 (d, *J* = 6.7 Hz, 6H, CH_3CHCH_3), 0.98 (t, *J* = 7.3 Hz, 6H, CH_2CH_3); **^{13}C-NMR** (126 MHz, C_6D_6, 298K): δ [ppm] = 148.1 (C_q), 147.7 (C_q), 145.1 (C_q), 142.0 (C_q), 139.5 (C_q), 137.6 (C_q), 137.0 (C_q), 134.8 (C_q), 134.4 (C_q), 132.4 (C_q), 129.2 (Ar-CH), 128.4 (Mes-CH), 128.3 (C_q), 127.9 (Ar-CH), 126.9 (C_q), 126.5 (Ar-CH), 126.2 (Ar-CH), 125.4 (Ar-CH), 125.0 (Ar-CH), 110.7 (Ar-CH), 105.9 (Pyridine-CH), 64.1 (C_q), 50.1 (C_q), 45.6 (CH_2), 35.8 (CH_2CH_3), 33.3 (N-CH_3), 29.6 (CH_3), 29.2 (CH_3CHCH_3), 25.8 (CH_3CHCH_3), 25.1 (CH_3CHCH_3), 21.2 (Mes-$^{para}CH_3$), 20.5 (Mes-$^{ortho}CH_3$), 10.5 (CH_2CH_3); **IR** [cm^{-1}]: ṽ = 2964, 2870, 2183, 2179, 2124, 1995, 1944, 1672, 1552, 1431, 1357, 1281, 1191, 1103, 991, 851, 785, 749, 691, 637, 572, 546, 453; **HR-MS-ESI(+)** calc. for $C_{47}H_{59}N_2^+$: 651.4673 $[M+H]^+$; found 651.4638; **UV-vis:** λ_{max}: 409 nm (ε = 10384 cm^{-1} M^{-1}).

Synthesis of radical cation **3h**

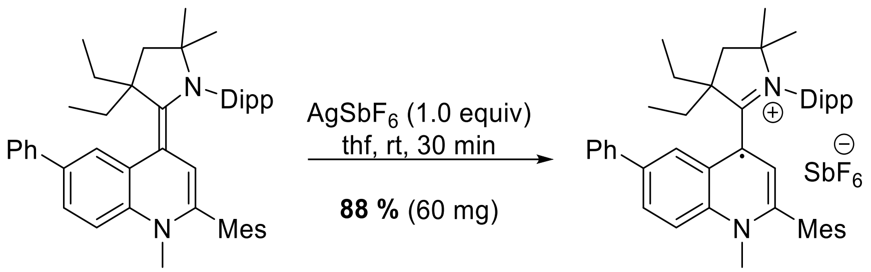

$AgSbF_6$ ($AgSbF_6$, 343.62 g/mol, 26.39 mg, 76.81 µmol, 1.0 equiv) was dissolved in thf (2 mL) and added dropwise to a solution of switch **2h** ($C_{47}H_{58}N_2$, 651.0 g/mol, 50.0 mg, 76.81 µmol, 1.0 equiv) in thf (4 mL). The now dark purple solution was stirred for 30 minutes at room temperature. After that the solution was filtrated over a celite plug (1.5 cm). Then the solvent was evaporated and the resulting dark solid was washed with pentane (3 x 5 mL). The dark solid was dried to give radical cation **3h** ($C_{47}H_{58}F_6N_2Sb$, 886.75, 60.0 mg, 67.66 µmol, 88 %) as a black solid.

m.p. 247 °C; **EPR:** ; g = 2.0035 (1xN 13.0355; 1xN 10.4437; 3xH 11.0355); **IR** [cm^{-1}]: ṽ = 2976, 2224, 2092, 2012, 1596, 1448, 1328, 1190, 1157, 832, 811, 771, 703, 684, 656, 547, 453; **HR-MS-ESI(+)** calc. for $C_{47}H_{58}N_2^+$: 650.4595 $[M]^+$; found 650.4562; **UV-vis:** λ_{max}: 389 nm (ε = 6576 cm^{-1} M^{-1}), 524 nm (ε = 5882 cm^{-1} M^{-1}), 684 nm (ε = 4311 cm^{-1} M^{-1}).

Synthesis of dication **4h**

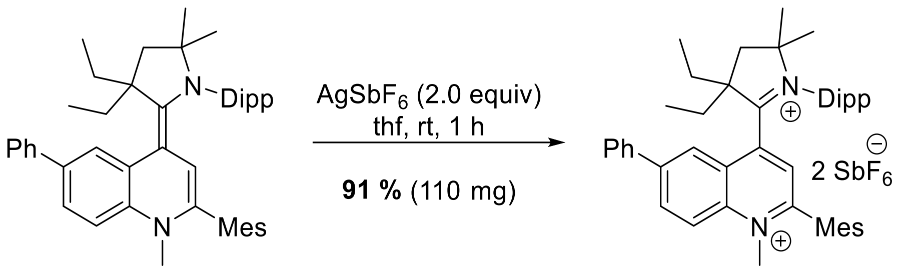

$AgSbF_6$ ($AgSbF_6$, 343.62 g/mol, 73.9 mg, 215.06 µmol, 2.0 equiv) was dissolved in thf (2 mL) and added dropwise to a solution of switch **2h** ($C_{47}H_{58}N_2$, 651.0 g/mol, 70.0 mg, 107.53 µmol, 1.0 equiv) in thf (4 mL). The solution turned dark purple and was stirred for 1 h. After that the solution was filtrated over a celite plug (1.5 cm) and the celite plug was washed with acetonitrile. Then the solvent was evaporated until 1 mL of the solution was left. Then Et_2O (20 mL) was added to precipitate a yellow solid. The Et_2O was decanted off and the yellow solid was washed with Et_2O three times and dried to give dication **4h** ($C_{47}H_{58}F_{12}N_2Sb_2$, 1122.5 g/mol, 110.0 mg, 98.0 µmol, 91 %) as a yellow powder.

m.p. 160 °C; **^{1}H-NMR** (500 MHz, CD_3CN, 298K): δ [ppm] = 8.71 (dd, *J* = 9.3, 1.9 Hz, 1H, Ar-CH), 8.66 (d, *J* = 9.4 Hz, 1H, Ar-CH), 8.32 (d, *J* = 2.0 Hz, 1H, Ar-CH), 7.90 – 7.84 (m, 2H, Ar-CH), 7.71 (t, *J* = 7.4 Hz, 2H, Ar-CH), 7.68 – 7.64 (m, 1H, Ar-CH), 7.57 (s, 1H, pyridine-CH), 7.48 (t, *J* = 7.8 Hz, 1H, Ar-CH), 7.41 (dd, *J* = 7.9, 1.6 Hz, 1H, Ar-CH), 7.28 – 7.18 (m, 2H, Ar-CH, Mes-CH), 7.09 (s, 1H-Mes-CH), 4.31 (s, 3H, N-CH_3), 2.91 (d, *J* = 14.1 Hz, 1H, CH_2), 2.79 (d, *J* = 14.1 Hz, 1H, CH_2), 2.72 – 2.66 (m, 2H, $CH_3C\mathit{H}CH_3$), 2.42 – 2.39 (m, 4H, $C\mathit{H}_2CH_3$, Mes-CH_3), 2.35 – 2.26 (m, 1H, $C\mathit{H}_2CH_3$), 2.17 – 2.10 (m, 2H, $C\mathit{H}_2CH_3$), 2.08 (s, 3H, Mes-CH_3), 1.84 (s, 3H, CH_3), 1.68 (s, 3H. CH_3), 1.44 (s, 3H, Mes-CH_3), 1.34 (d, *J* = 6.2 Hz, 3H, $CH_3CHC\mathit{H}_3$), 1.19 (d, *J* = 6.3 Hz, 3H, $CH_3CHC\mathit{H}_3$), 0.96 (t, *J* = 7.3 Hz, 3H, $CH_2C\mathit{H}_3$), 0.86 (d, *J* = 6.4 Hz, 3H, $CH_3CHC\mathit{H}_3$), 0.82 (t, *J* = 7.3 Hz, 3H, $CH_2C\mathit{H}_3$), 0.05 (d, *J* = 6.5 Hz, 3H, $CH_3CHC\mathit{H}_3$); **^{13}C-NMR** (126 MHz, CD_3CN, 298K): δ [ppm] = 197.9 (C_q), 160.1 (C_q), 146.0 (C_q), 145.5 (C_q), 145.4 (C_q), 143.9 (C_q), 142.9 (C_q), 141.1 (C_q), 138.0 (Ar-CH), 137.4 (C_q), 137.0 (C_q), 136.8 (C_q), 133.3 (Ar-CH), 131.3 (Ar-CH), 130.9 (Ar-CH), 130.6 (Mes-CH), 130.3 (Mes-CH), 129.4 (C_q), 129.1 (Ar-CH), 128.6 (Ar.CH), 128.57 (C_q), 128.4 (Ar-CH), 126.5 (C_q), 124.9 (Ar-CH), 124.86 (Pyridine-CH), 123.8 (Ar-CH), 88.6 (C_q), 68.3 (C_q), 63.0 (C_q), 45.4 (CH_2), 43.1 (N-CH_3), 32.7 ($\mathit{C}H_2CH_3$), 31.6 (CH_3), 30.8 ($\mathit{C}H_2CH_3$), 30.3 ($CH_3\mathit{C}HCH_3$), 30.2 ($CH_3\mathit{C}HCH_3$), 28.6 (CH_3), 26.6 ($\mathit{C}H_3CHCH_3$), 26.3 (C_q), 26.2 ($\mathit{C}H_3CHCH_3$), 26.15 ($\mathit{C}H_3CHCH_3$), 25.5 ($\mathit{C}H_3CHCH_3$), 21.3 (Mes-CH_3), 20.7 (Mes-CH_3), 19.6 (Mes-CH_3), 9.5 (CH_3CH_2), 9.45 (CH_3CH_2); **IR** [cm^{-1}]: ṽ = 2978, 2142, 2015, 1986, 1571, 1457, 1388, 1274, 1133, 1095, 1019, 930, 855, 807, 763, 699, 653, 568; **HR-MS-ESI(+)** calc. for $C_{47}H_{58}N_2^{2+}$: 325.2295 $[M]^{2+}$; found 325.2297; **UV-vis:** λ_{max}: 355 nm (ε = 3377 cm^{-1} M^{-1}), 390 nm (ε = 3764 cm^{-1} M^{-1}).

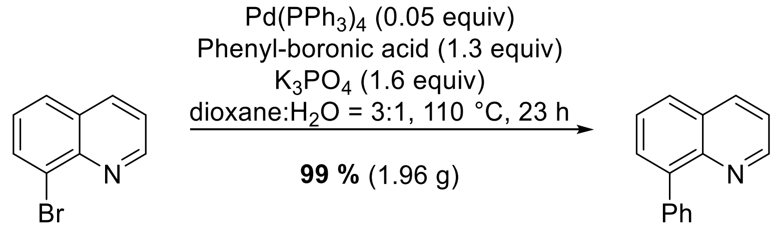

8-Ph-quinoline **S10:** Subsequently $Pd(PPh_3)_4$ ($C_{72}H_{60}P_4Pd$, 1155.59 g/mol, 555.42 mg, 480.64 µmol, 0.05 equiv), 8-bromo-quinoline (C_9H_6BrN, 208.06 g/mol, 2.0 g, 1.59 g/mL, 1.25 mL, 9.61 mmol, 1 equiv),

phenylboronic acid ($C_6H_7BO_2$, 121.93 g/mol, 1.52 g, 12.5 mmol, 1.3 equiv) and potassium phosphate (K_3PO_4, 212.26 g/mol, 3.26 g, 15.38 mmol, 1.6 equiv) were dissolved in a 3:1 mixture of degassed dioxane and H_2O (30:10 mL). The yellow suspension was then stirred for 23 hours at 110 °C. After cooling Na_2SO_4 was added and the resulting suspension was filtered over a Na_2SO_4-plug (4 cm) and rinsed with EtOAc until the solution turned colourless. The residual solvent was evaporated under reduced pressure and the crude product was then purified by column chromatography on silica (Cyclohexane/EtOAc: 100/0 to 90/10) to yield 8-Ph-quinoline **S10** ($C_{15}H_{11}N$, 205.26 g/mol, 1.96 g, 9.55 mmol, 99 %) as a yellow oil. The spectroscopic data are in good agreement with previous reports.[9]

^{1}H NMR (500 MHz, $CDCl_3$, 298K): δ [ppm] = 8.97 (dd, *J* = 4.1, 1.8 Hz, 1H), 8.21 (dd, *J* = 8.3, 1.8 Hz, 1H), 7.78 – 7.68 (m, 3H), 7.61 (dd, *J* = 8.1, 7.1 Hz, 1H), 7.55 – 7.47 (m, 2H), 7.46 – 7.39 (m, 2H); **^{13}C NMR** (126 MHz, $CDCl_3$, 298K): δ [ppm] = 150.41, 146.20, 141.08, 139.70, 136.36, 130.75, 130.45, 128.88, 128.14, 127.67, 127.51, 126.40, 121.12.

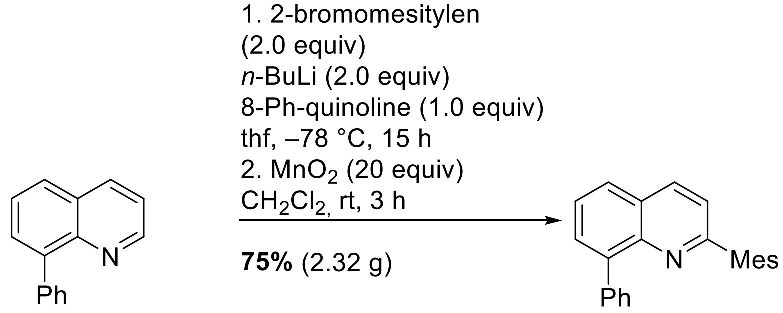

2-Mesitylene-8-Ph-quinoline **S11:** 2-Bromomesitylen ($C_9H_{11}Br$, 199.09 g/mol, 3.8 g, 1.3 g/mL, 2.92 mL, 19.1 mmol, 2.0 equiv) was dissolved in thf (40 mL) and cooled down to –78 °C. Then *n*-BuLi (C_4H_9Li, 64.06 g/mol, 1.22 g, 1.6 M, 11.94 mL, 19.1 mmol, 2.0 equiv) was slowly added and the resulting white suspension was stirred for 10 minutes at –78 °C. This suspension was then added to a solution of 8-Ph-quinoline **S10** ($C_{15}H_{11}N$, 205.26 g/mol, 1.96 g, 9.55 mmol, 1.0 equiv) in thf (40 mL), which was also cooled to –78 °C. The quinoline solution turned orange instantly and was allowed to warm up to room temperature over night (15 h). The orange solution was slowly poured over ice and then extracted using ethylacetate (3 x 50 mL). The combined organic phases were dried using Na_2SO_4 and the residue solvent was removed under reduced pressure. The yellow oil was then dissolved in CH_2Cl_2 (200 mL) and cooled to 0 °C. Then activated MnO_2 (MnO_2, 86.94 g/mol, 16.6 g, 190.98 mmol, 20 equiv) was added resulting in a brown suspension. After 3 hours the suspension was filtrated over a silica plug (4 cm) using ethyl acetate as solvent. The crude product was then purified by column chromatography on silica (Cyclohexane/EtOAc: 100/0 to 95/5) to yield 2-mesitylene-8-Ph-quinoline **S11** ($C_{24}H_{21}N$, 323.44 g/mol, 2.32 g, 7.17 mmol, 75 %) as a yellow oil.

^{1}H NMR (500 MHz, CD_3CN, 298K): δ [ppm] = 8.23 (d, *J* = 8.4 Hz, 1H, Ar-CH), 7.86 (dd, *J* = 8.1, 1.5 Hz, 1H, Ar-CH), 7.76 (dd, *J* = 7.2, 1.5 Hz, 1H, Ar-CH), 7.74 – 7.68 (m, 2H, Ar-CH), 7.61 (dd, *J* = 8.1, 7.1 Hz, 1H, Ar-CH), 7.44 – 7.30 (m, 4H, Ar-CH), 6.93 (s, 2H, Mes-CH), 2.33 (s, 3H, Mes-$^{para}CH_3$), 2.09 (s, 6H, Mes-$^{ortho}CH_3$); **^{13}C NMR** (126 MHz, CD_3CN, 298K): δ [ppm] = 160.0 (C_q), 145.9 (C_q), 141.1 (C_q), 140.0 (C_q), 138.4 (C_q), 137.4 (C_q), 136.1 (C_q), 136.0 (Ar-CH), 131.0 (Ar-CH), 130.6 (Ar-CH), 128.5 (Mes-CH), 127.8 (Ar-CH), 127.4 (Ar-CH), 127.3 (C_q), 127.1 (Ar-CH), 126.1 (Ar-CH), 123.5 (Ar-CH), 21.21 (Mes-$^{para}CH_3$), 20.66 (Mes-$^{ortho}CH_3$); **IR** [cm^{-1}]: $\tilde{\nu}$ = 3058, 2920, 2857, 1738, 1611, 1599, 1562, 1482, 1455, 1436, 1377, 1325, 1270, 1241, 1169, 1137, 1118,

1069, 1041, 968, 908, 844, 819, 758, 694, 648, 613, 589, 565, 523, 495, 459, 430; **HR-MS-ESI(+)** calc. for C24H22N^{+}: 324.1747 $[M+H]^{+}$; found 324.1746.

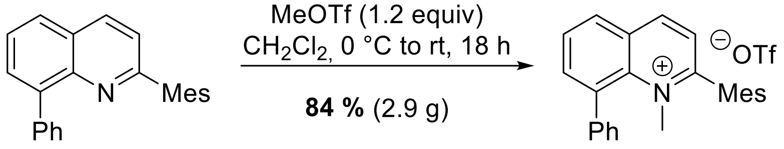

N-Methyl-2-mesitylene-8-Ph-quinolinium salt **1i:** 2-mesitylene-8-Ph-quinoline **S11** ($C_{24}H_{21}N$, 323.44 g/mol, 2.3 g, 7.11 mmol, 1 equiv) was dissolved in CH_2Cl_2 (40 mL) and cooled to 0°C. Then MeOTf ($C_2H_3F_3O_3S$, 164.1 g/mol, 1.4 g, 1.5 g/mL, 933.53 µL, 8.53 mmol, 1.2 equiv) was added dropwise. The yellow solution was allowed to warm up to room temperature over night (18 h). The solvent was evaporated until 2-3 mL of the solvent were left over. Then Et_2O (60 mL) was added resulting in the formation of a pale off-white solid. The solvent was removed using a filtration cannula and the residue solid was washed with Et_2O (3 x 20 mL). Drying of the solid yielded *N*-methyl-2-mesitylene-8-Ph-quinolinium salt **1i** ($C_{26}H_{24}F_3NO_3S$, 487.54 g/mol, 2.9 g, 5.95 mmol, 84 %) as an off-white powder.

m.p. 182 °C; **^{1}H NMR** (500 MHz, CD_3CN, 298K): δ [ppm] = 9.27 (d, *J* = 8.4 Hz, 1H, Ar-CH), 8.48 (dd, *J* = 7.8, 1.9 Hz, 1H, Ar-CH), 8.12 – 8.01 (m, 2H, Ar-CH), 7.95 (d, *J* = 8.4 Hz, 1H, Ar-CH), 7.61 – 7.46 (m, 5H, Ar-CHz), 7.13 (s, 2H, Mes-CH), 3.49 (s, 3H, N-CH_3), 2.35 (s, 3H, Mes-$^{para}CH_3$), 2.04 (s, 6H, Mes-$^{ortho}CH_3$); **^{13}C NMR** (126 MHz, CD_3CN, 298K): δ [ppm] = 163.2 (C_q), 149.2 (Ar-CH), 142.7 (C_q), 141.4 (C_q), 141.1 (Ar-CH), 140.9 (C_q), 136.4 (C_q), 136.0 (C_q), 132.1 (C_q), 131.6 (Ar-CH), 131.1 (C_q), 130.4 (Ar-CH), 130.3 (Mes-CH), 130.2 (Ar-CH), 129.9 (Ar-CH), 129.8 (Ar-CH), 126.3 (Ar-CH), 48.3 (N-CH_3), 21.4 (Mes-$^{para}CH_3$), 20.0 (Mes-$^{ortho}CH_3$); **IR** [cm^{-1}]: ṽ = 1605, 1576, 1517, 1447, 1354, 1278, 1258, 1223, 1144, 1030, 860, 763, 707, 636, 603, 589, 572, 550, 516; **HR-MS-ESI(+)** calc. for $C_{25}H_{24}N^{+}$: 338.1903 $[M]^{+}$; found 338.1911.

Synthesis of neutral **2i**

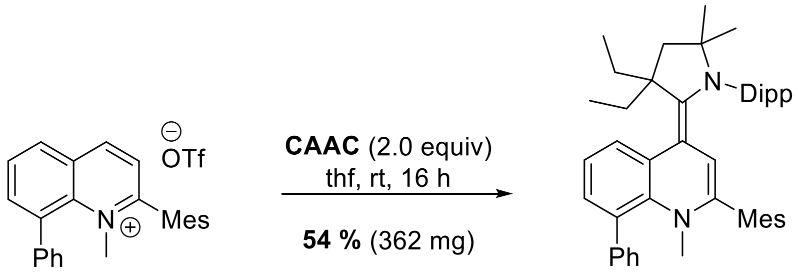

To a solution of CAAC (C22H35N, 313.53 g/mol, 643.09 mg, 2.05 mmol, 2.0 equiv) in thf (30 mL) a solution of quinolinium salt **1i** ($C_{26}H_{24}F_3NO_3S$, 487.54 g/mol, 500.0 mg, 1.03 mmol, 1.0 equiv) in thf (40 mL) was added. The orange solution was then stirred for 12 h at room temperature. The solvent was removed under reduced pressure and the residual solid was extracted with pentane (40+3x20 mL) using a filtration cannula. Recrystallisation of the crude product from a saturated pentane solution at –40 °C yielded the switch **2i** ($C_{47}H_{58}N_2$, 651.0 g/mol, 362 mg, 556.07 µmol, 54 %) as orange crystals with an *E*:*Z* ratio of 1:0.32.

Single crystals suitable for X-ray diffraction could be obtained from a saturated pentane solution at –40 °C.

m.p. 182 °C; **^{1}H-NMR** (*E*-Isomer) (500 MHz, C_6D_6, 298K): δ [ppm] = 7.50 – 7.45 (m, 1H, Ar-CH), 7.35 (dd, *J* = 7.5, 1.7 Hz, 1H, Ar-CH), 7.15 – 7.10 (m, 3H, Ar-CH), 7.07 (s, 3H), 7.01 – 6.94 (m, 2H, Ar-CH), 6.66 (s, 2H, Mes-CH), 5.17 (s, 1H, pyridine-CH), 3.62 (hept, *J* = 6.6 Hz, 2H, $CH_3C\mathit{H}CH_3$), 2.30 (s, 3H, N-CH_3), 2.16 (s, 6H, Mes-$^{orrtho}CH_3$), 2.08 (s, 3H, Mes-$^{para}CH_3$), 1.97 (hept, *J* = 7.0 Hz, 4H, $CH_3C\mathit{H}_2$), 1.86 (s, 2H, $C\mathit{H}_2$), 1.29 (d, *J* =

6.7 Hz, 6H, CH_3CHCH$_3$), 1.12 (s, 6H, CH_3), 1.07 (d, J = 6.6 Hz, 6H, CH_3CHCH$_3$), 1.02 (t, J = 7.3 Hz, 6H, CH_3CH$_2$); Due to the complex isomeric mixture it was not possible to clearly assign the signals of only one isomer, instead both are described. **^{13}C NMR** (126 MHz, C_6D_6, 298K): δ [ppm] = 148.47, 148.36, 148.14, 147.36, 144.95, 144.43, 144.35, 142.97, 140.71, 139.26, 138.10, 137.98, 137.30, 137.04, 136.47, 135.78, 135.15, 134.95, 130.39, 130.09, 129.52, 128.93, 128.67, 128.58, 128.48, 128.46, 128.30, 126.98, 126.67, 126.35, 126.33, 125.50, 125.22, 120.17, 119.73, 112.81, 109.83, 106.00, 103.67, 64.18, 63.47, 51.13, 49.77, 49.42, 44.98, 40.78, 38.53, 34.74, 33.66, 30.88, 29.50, 29.16, 28.74, 27.16, 25.69, 25.10, 23.65, 21.12, 21.10, 20.81, 20.63, 10.87, 10.41; **IR** [cm^{-1}]: ṽ = 2971, 2944, 2916, 2863, 1628, 1535, 1467, 1446, 1433, 1398, 1381, 1366, 1321, 1288, 1251, 1207, 1191, 1173, 1154, 1095, 1066, 1034, 1013, 996, 899, 850, 825, 801, 790, 759, 740, 696, 669, 649, 628, 588, 564, 546, 532, 501, 474, 433; **HR-MS-ESI(+)** calc. for $C_{47}H_{58}N_2^{2+}$: 325.2295 [M]$^{2+}$; found 325.2290; **UV-vis:** λ_{max}: 347 nm (ε = 11526 cm^{-1} M^{-1}), 405 nm (ε = 13226 cm^{-1} M^{-1}).

Synthesis of radical cation **3i**

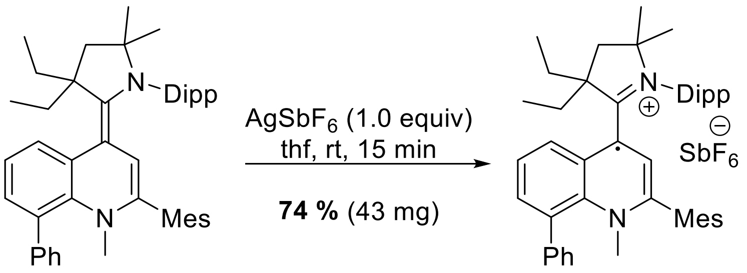

AgSbF$_6$ (AgSbF$_6$, 343.62 g/mol, 22.33 mg, 64.98 µmol, 1.0 equiv) was dissolved in thf (4 mL) and added dropwise to a solution of hybrid system **2i** ($C_{47}H_{58}N_2$, 651.0 g/mol, 42.3 mg, 64.98 µmol, 1.0 equiv) in thf (4 mL). The now dark solution was stirred for 15 minutes at room temperature. After that the solution was filtrated over a celite plug (1.5 cm). Then the solvent was evaporated until 1 mL of solvent was left. Pentane (17 mL) was added to precipitate a dark solid. The solvent was decanted off and the leftover solid was washed with pentane (4 x 5 mL). Drying under reduced pressure yielded the radical cation **3i** ($C_{47}H_{58}F_6N_2Sb$, 886.75 g/mol, 42.6 mg, 48.04 µmol, 74 %) as a purple powder.

m.p. 148 °C (decomp.); **EPR:** g = 2.0034 (1xN 10.0799; 1xN 11.977; 1xH 4.36435; 3xH 10.9798); **IR** [cm^{-1}]: ṽ = 2975, 2930, 1611, 1597, 1569, 1441, 1388, 1356, 1326, 1299, 1248, 1187, 1162, 1114, 1060, 1028, 928, 853, 808, 767, 706, 655, 562, 537; **HR-MS-ESI(+)** calc. for $C_{47}H_{58}N_2^{+}$: 650.4595 [M]$^+$; found 650.4596; **UV-vis:** λ_{max}: 381 nm (ε = 5469 cm^{-1} M^{-1}), 530 nm (ε = 3705 cm^{-1} M^{-1}), λ_{max}: 670 nm (ε = 3142 cm^{-1} M^{-1}).

Synthesis of dication **4i**

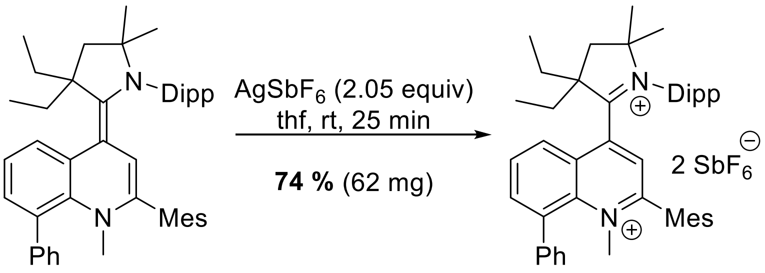

AgSbF$_6$ (AgSbF$_6$, 343.62 g/mol, 53.0 mg, 154.3 µmol, 2.0 equiv) was dissolved in thf (4 mL) and added dropwise to a solution of switch **2i** ($C_{47}H_{58}N_2$, 651.0 g/mol, 49.0 mg, 75.27 µmol, 1.0 equiv) in thf (4 mL). The dark yellow solution was stirred for 25 minutes and then filtrated over a celite plug (1.5 cm). Then the

solvent was evaporated until 1 mL of solvent was left. Et_2O (18 mL) was added to form a yellow precipitate. The solvent was decanted off and the leftover solid was washed with Et_2O (2 x 8 mL). Drying under reduced pressure yielded dication **4i** ($C_{47}H_{58}F_{12}N_2Sb_2$, 1122.5 g/mol, 62.4 mg, 55.59 µmol, 74 %) as a yellow solid.

m.p. 192 °C (decomp.); **^{1}H-NMR** (500 MHz, CD_3CN, 298K): δ [ppm] = 8.45 (dd, *J* = 8.2, 1.5 Hz, 1H, Ar-CH), 8.29 (dd, *J* = 7.3, 1.5 Hz, 1H, Ar-CH), 8.23 (dd, *J* = 8.2, 7.3 Hz, 1H, Ar-CH), 7.72 (s, 1H, pyridine-CH), 7.68 (dt, *J* = 7.8, 1.7 Hz, 1H, Ar-CH), 7.63 (td, *J* = 7.5, 1.5 Hz, 1H, Ar-CH), 7.55 (tt, *J* = 7.5, 1.4 Hz, 1H, Ar-CH), 7.53 – 7.47 (m, 2H, Ar-CH), 7.41 (d, *J* = 1.5 Hz, 1H, Ar-CH), 7.31 (dd, *J* = 7.8, 1.5 Hz, 1H, Ar-CH), 7.23 (dt, *J* = 7.7, 1.7 Hz, 1H, Ar-CH), 7.17 (s, 1H, Mes-CH), 7.08 (s, 1H, Mes-CH), 3.52 (s, 3H, N-CH_3), 2.91 (h, *J* = 6.4 Hz, 1H, $CH_3C\mathit{H}CH_3$), 2.88 (d, *J* = 14.0 Hz, 1H, CH_2), 2.74 (d, *J* = 14.0 Hz, 1H, CH_2), 2.69 (h, *J* = 6.3 Hz, 1H, $CH_3C\mathit{H}CH_3$), 2.35 (s, 3H, Mes-CH_3), 2.31 – 2.07 (m, 4H, $C\mathit{H}_2CH_3$), 2.05 (s, 3H, Mes-CH_3), 1.79 (s, 3H, CH_3), 1.72 (s, 3H, Mes-CH_3), 1.63 (s, 3H, CH_3), 1.39 – 1.33 (m, 6H, $CH_3CHC\mathit{H}_3$), 1.11 (t, *J* = 7.4 Hz, 3H, $CH_2C\mathit{H}_3$), 0.88 (d, *J* = 6.4 Hz, 3H, $CH_3CHC\mathit{H}_3$), 0.68 (t, *J* = 7.4 Hz, 3H, $CH_2C\mathit{H}_3$), 0.29 (d, *J* = 6.4 Hz, 3H, $CH_3CHC\mathit{H}_3$); **^{13}C NMR** (126 MHz, CD_3CN, 298K): δ [ppm] = 197.5 (C_q), 162.7 (C_q), 146.1 (C_q), 145.7 (C_q), 143.8 (C_q), 143.6 (C_q), 142.7 (Ar-CH), 141.4 (C_q), 140.5 (C_q), 137.6 (C_q), 136.7 (C_q), 136.3 (C_q), 133.2 (Ar-CH), 132.3 (Ar-CH), 130.9 (Ar-CH), 130.7 (Mes-CH), 130.5 (Mes-CH), 130.3 (Ar-CH), 130.1 (Ar-CH), 130.1 (Ar-CH), 129.3 (C_q), 129.3 (Ar-CH), 129.1 (C_q), 128.9 (Ar-CH), 128.3 (Ar-CH), 128.2 (Ar-CH), 127.6 (C_q), 124.8 (pyridine-CH), 88.5 (C_q), 62.9 (C_q), 50.3 (N-CH_3), 44.6 (CH_2), 31.7 ($\mathit{C}H_2CH_3$), 30.9 (CH_3), 30.9 ($\mathit{C}H_2CH_3$), 30.4 ($CH_3\mathit{C}HCH_3$), 30.1 ($CH_3\mathit{C}HCH_3$), 28.5 ($CH_3CH\mathit{C}H_3$), 28.2 (CH_3), 26.8 ($CH_3CH\mathit{C}H_3$), 25.5 ($CH_3CH\mathit{C}H_3$), 25.0 ($CH_3CH\mathit{C}H_3$), 21.2 (Mes-CH_3), 20.9 (Mes-CH_3), 19.5 (Mes-CH_3), 9.5 ($CH_2\mathit{C}H_3$), 9.5 ($CH_2\mathit{C}H_3$); **IR** [cm^{-1}]: ṽ = 2980, 1610, 1594, 1566, 1507, 1454, 1419, 1393, 1378, 1340, 1167, 1138, 855, 824, 810, 769, 734, 708, 654, 568, 539; **HR-MS-ESI(+)** calc. for $C_{47}H_{58}N_2^{2+}$: 325.2295 $[M]^{2+}$; found 325.2298; **UV-vis:** λ_{max}: 342 nm (ε = 6554 cm^{-1} M^{-1}), 380 nm (ε = 6665 cm^{-1} M^{-1}).

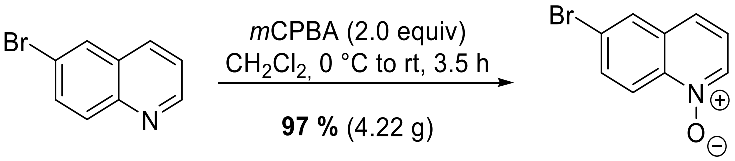

N-Oxide **S12**: 6-Bromo-quinoline (C_9H_6BrN, 208.06 g/mol, 4.0 g, 19.23 mmol, 1 equiv) was dissolved in CH_2Cl_2 (120 mL) and cooled to 0 °C. Then *m*CPBA ($C_7H_5ClO_3$, 172.56 g/mol, 8.85 g, 38.45 mmol, 2 equiv) was added. After 5 minutes the now brown solution was warmed up to ambient temperature and stirred for 3.5 hours. The solution was then transferred into a separation funnel and washed 3 times with aqueous KOH-solution (6 M) The organic phase was dried over Na_2SO_4 and the residual solvent was evaporated under reduced pressure to yield *N*-Oxide **S12** (C_9H_6BrNO, 224.06 g/mol, 4.22 g, 19.23 mmol, 97 %) as a white solid. The spectroscopic data are in good agreement with previous reports.[10]

^{1}H NMR (500 MHz, $CDCl_3$, 298K): δ [ppm] = 8.60 (d, *J* = 9.3 Hz, 1H), 8.49 (dd, *J* = 6.1, 1.0 Hz, 1H), 8.02 (d, *J* = 2.1 Hz, 1H), 7.80 (dd, *J* = 9.3, 2.1 Hz, 1H), 7.64 – 7.59 (m, 1H), 7.30 (dd, *J* = 8.5, 6.0 Hz, 1H); **^{13}C NMR** (126 MHz, $CDCl_3$, 298K): δ [ppm] = 140.6, 135.8, 133.8, 131.7, 130.2, 124.6, 123.4, 122.3, 121.9.

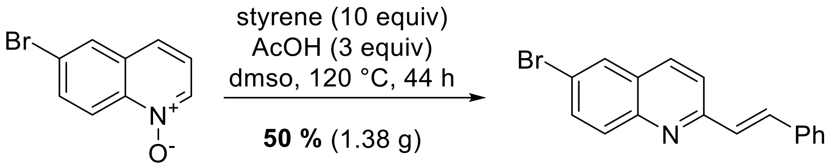

2-Styrene-6-bromo-quinoline **S13**: *N*-Oxide **S12** (C_9H_6BrNO, 224.06 g/mol, 2.0 g, 8.93 mmol, 1 equiv) was dissolved in dmso (120 mL). Subsequently styrene (C_8H_8, 104.15 g/mol, 9.3 g, 0.91 g/mL, 10.22 mL, 89.26 mmol, 10 equiv) and acetic acid ($C_2H_4O_2$, 60.05 g/mol, 1.61 g, 1.05 g/mL, 1.53 mL, 26.78 mmol, 3 equiv) were added. The yellow solution was stirred under reflux for 44 h at 120 °C. The now orange solution was cooled down, diluted with H_2O (50 mL) and then extracted with Et_2O (5 x 60 mL). The combined organic phase was then washed with brine (2x) and then dried over Na_2SO_4. The residual solvent was evaporated under reduced pressure. Recrystallisation from hot Et_2O yielded 2-styrene-6-bromo-quinoline **S13** ($C_{17}H_{12}BrN$, 310.19 g/mol, 1.38 g, 4.45 mmol, 50 %) as an off white solid. The spectroscopic data are in good agreement with previous reports.[11]

^{1}H NMR (500 MHz, $CDCl_3$, 298K): δ [ppm] = 8.03 (d, *J* = 8.6 Hz, 1H), 7.97 – 7.91 (m, 2H), 7.77 (dd, *J* = 9.0, 2.2 Hz, 1H), 7.74 – 7.61 (m, 4H), 7.49 – 7.31 (m, 4H); **^{13}C NMR** (126 MHz, $CDCl_3$, 298K): δ [ppm] = 156.5, 147.0, 136.5, 135.5, 135.2, 133.3, 131.1, 129.7, 129.0, 129.0, 128.7, 128.5, 127.5, 120.4, 120.1.

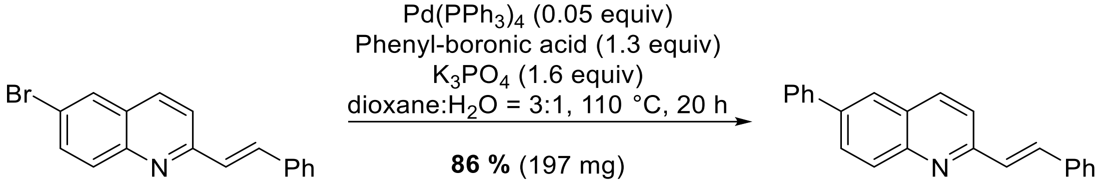

6-Ph-2-styrene-quinoline **S14:** Subsequently $Pd(PPh_3)_4$ ($C_{72}H_{60}P_4Pd$, 1155.59 g/mol, 42.84 mg, 37.07 µmol, 0.05 equiv), 2-styrene-6-bromo-quinoline **S13** ($C_{17}H_{12}BrN$, 310.19 g/mol, 230 mg, 741.47 µmol, 1 equiv), phenylboronic acid ($C_6H_7BO_2$, 121.93 g/mol, 117.53 mg, 963.91 µmol, 1.3 equiv) and potassium phosphate (K_3PO_4, 212.26 g/mol, 251.82 mg, 1.19 mmol, 1.6 equiv) were dissolved in a 3:1 mixture of degassed dioxane and H_2O (9:3 mL). The yellow suspension was then stirred for 20 hours at 110 °C. After cooling Na_2SO_4 was added and the resulting suspension was filtered over a Na_2SO_4-plug (4 cm) and rinsed with EtOAc. The residual solvent was evaporated under reduced pressure and the crude product was then purified by column chromatography on silica (Cyclohexane/EtOAc: 100/0 to 90/10) to yield 6-Ph-2-styrene-quinoline **S14** ($C_{23}H_{17}N$, 307.4 g/mol, 197 mg, 640.87 µmol, 86 %) as a yellow solid.

m.p. 198°C; **^{1}H NMR** (500 MHz, CD_3CN, 298K): δ [ppm] = 8.20 – 8.12 (m, 2H, Ar-CH), 8.04 – 7.95 (m, 2H, Ar-CH), 7.81 – 7.61 (m, 6H, Ar-CH), 7.57 – 7.47 (m, 2H, Ar-CH), 7.47 – 7.36 (m, 4H, Ar-CH), 7.36 – 7.30 (m, 1H, Ar-CH); **^{13}C NMR** (126 MHz, CD_3CN, 298K): δ [ppm] = 156.1 (C_q), 147.8 (C_q), 140.5 (C_q), 139.0 (C_q), 136.7 (C_q), 136.6 (Ar-CH), 134.6 (Ar-CH), 129.8 (Ar-CH), 129.6 (Ar-CH), 129.1 (Ar-CH), 129.1 (Ar-CH), 129.0 (Ar-CH), 128.8 (Ar-CH), 127.8 (Ar-CH), 127.7 (C_q), 127.5 (Ar-CH), 127.4 (Ar-CH), 125.4 (Ar-CH), 119.8 (Ar-CH); **IR** [cm^{-1}]: ṽ = 1635, 1588, 1489, 1445, 1393, 1321, 1204, 1126, 1074, 1031, 948, 892, 843, 819, 786, 765, 750, 693, 629, 610, 540, 520, 487, 434; **HR-MS-ESI(+)** calc. for $C_{23}H_{18}N^+$: 308.1434 $[M+H]^+$; found 308.1433.

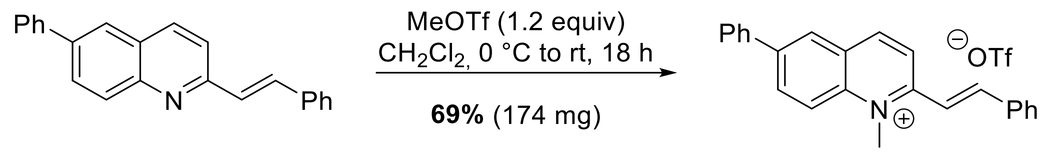

N-Methyl-2-styrene-6-Ph-quinolinium salt **1j:** 2-styrene-6-Ph-quinoline **S14** ($C_{23}H_{17}N$, 307.4 g/mol, 165 mg, 536.77 µmol, 1 equiv) was dissolved in CH_2Cl_2 (10 mL) and cooled to 0°C. Then MeOTf ($C_2H_3F_3O_3S$, 164.1 g/mol, 105.7 mg, 1.5 g/mL, 70.47 µL, 644.12 µmol, 1.2 equiv) was added dropwise. The yellow solution was allowed to warm up to room temperature overnight (18 h). Then the solvent was evaporated until 2-3 mL of the solvent were left over. Then Et_2O (60 mL) was added resulting in the formation of a yellow solid. The solvent was separated using a filtration cannula and the residue solid was washed with Et_2O (3 x 20 mL). Drying off the solid under reduced pressure yielded *N*-methyl-2-styrene-6-Ph-quinolinium salt **1j** ($C_{25}H_{20}F_3NO_3S$, 471.49 g/mol, 174 mg, 369.04 µmol, 64 %) as a yellow powder.

m.p. 280 °C (decomp.); **^{1}H NMR** (500 MHz, CD_3CN, 298K): δ [ppm] = 8.88 (d, *J* = 8.8 Hz, 1H, Ar-CH), 8.52 – 8.35 (m, 3H, Ar-CH), 8.28 (d, *J* = 8.8 Hz, 1H, Ar-CH), 7.92 (d, *J* = 16.0 Hz, 1H, styrene-C*H*CH), 7.88 – 7.81 (m, 4H, Ar-CH), 7.68 (d, *J* = 16.0 Hz, 1H, styrene-C*H*=CH), 7.60 – 7.51 (m, 5H, Ar-CH), 7.51 – 7.43 (m, 1H, Ar-CH), 4.48 (s, 3H, N-C*H*$_3$); **^{13}C NMR** (126 MHz, CD_3CN, 298K): δ [ppm] = 157.3 (C_q), 148.0 (styrene *C*H=CH), 145.8 (Ar-CH), 142.2 (C_q), 139.9 (C_q), 138.5 (C_q), 135.7 (C_q), 135.2 (Ar-CH), 132.6 (Ar-CH), 130.4 (Ar-CH), 130.3 (Ar-CH), 130.1 (Ar-CH), 129.9 (Ar-CH), 129.8 (C_q), 128.4 (Ar-CH), 128.2 (Ar-CH), 122.8 (Ar-CH), 120.5 (Ar-CH), 119.9 (styrene *C*H=CH), 41.0 (N-*C*H$_3$); **IR** [cm^{-1}]: ṽ = 1603, 1581, 1509, 1453, 1383, 1354, 1258, 1225, 1153, 1073, 1029, 921, 824, 773, 755, 693, 636, 573, 517; **HR-MS-ESI(+)** calc. for $C_{24}H_{20}N^+$: 322.1591 [M]$^+$; found 322.1589.

Synthesis of neutral **2j**

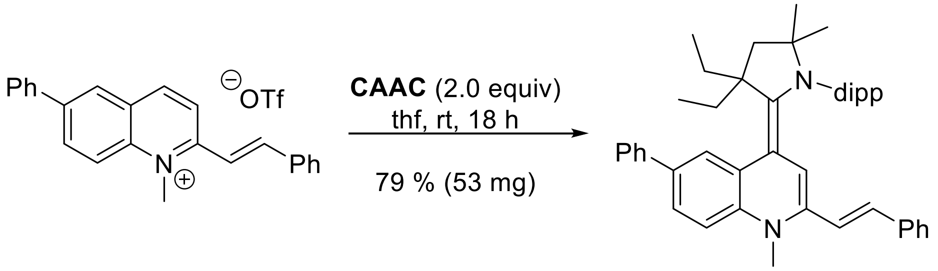

To a solution of CAAC (C22H35N, 313.53 g/mol, 68.16 mg, 217.39 µmol, 2.0 equiv) in thf (4 mL) a solution of quinolinium salt **1j** ($C_{25}H_{30}F_3NO_3S$, 471.49 g/mol, 50 mg, 106.05 µmol, 1.0 equiv) in thf (4 mL) was added. The orange solution was then stirred for 18 h at room temperature. The solvent was removed under reduced pressure and the residual solid was extracted with pentane using a filtration cannula. Recrystallisation of the crude product from a saturated pentane solution at –40 °C yielded the neutral **2j** ($C_{46}H_{54}N_2$, 634.95 g/mol, 53 mg, 83.47 µmol, 79 %) as red crystals exclusively as *E*-isomer.

Single crystals suitable for X-ray diffraction could be obtained from a saturated pentane solution at –40 °C.

m.p. 181 °C (decomp.); **^{1}H NMR** (*E*-Isomer) (500 MHz, CD_3CN, 298K): δ [ppm] = 7.82 (d, *J* = 2.1 Hz, 1H, Ar-CH), 7.80 – 7.74 (m, 2H, Ar-CH), 7.46 (dd, *J* = 8.3, 2.1 Hz, 1H, Ar-CH), 7.39 – 7.35 (m, 2H, Ar-CH), 7.33 (t, *J* = 7.7 Hz, 2H, Ar-CH), 7.29 (dd, *J* = 8.4, 7.0 Hz, 1H, Ar-CH), 7.23 – 7.18 (m, 4H, Ar-CH), 7.18 – 7.14 (m, 2H, Ar-CH), 7.11 – 7.03 (m, 1H, Ar-CH), 6.69 (d, *J* = 8.3 Hz, 1H, Ar-CH), 6.35 (d, *J* = 16.0 Hz, 1H, styrene CH=C*H*), 6.17 (d, *J* = 16.0 Hz, 1H, styrene CH=C*H*), 4.92 (s, 1H, pyridine-CH), 3.56 – 3.49 (m, 2H, CH_3CHCH_3), 2.87 (s,

3H, N-CH_3), 2.06 – 1.84 (m, 4H, CH_3CH_2), 1.82 (s, 2H, CH_2), 1.35 (d, *J* = 6.7 Hz, 6H, CH_3CHCH_3), 1.28 (d, *J* = 6.8 Hz, 6H, CH_3CHCH_3), 1.16 (s, 6H, CH_3), 0.91 (t, *J* = 7.3 Hz, 6H, CH_3CH_2); **^{13}C NMR** (126 MHz, CD_3CN, 298K): δ [ppm] = 149.8 (C_q), 147.8 (C_q), 147.3 (C_q), 142.2 (C_q), 138.9 (C_q), 138.6 (C_q), 136.7 (C_q), 132.7 (C_q), 129.2 (Ar-CH), 128.9 (Ar-CH), 128.7 (styrene *C*H=CH), 128.7 (C_q), 127.5 (Ar-CH), 127.3 (Ar-CH), 126.6 (Ar-CH), 126.5 (Ar-CH), 126.4 (Ar-CH), 125.2 (Ar-CH), 124.3 (Ar-CH), 123.3 (styrene CH=*C*H), 111.2 (Ar-CH), 110.6 (pyridine-CH), 101.4 (C_q), 64.0 (C_q), 50.4 (C_q), 45.6 (CH_2), 34.5 (CH_3CH_2), 34.1 (N-CH_3), 29.4 (CH_3), 29.1 (CH_3CHCH_3), 25.0 (CH_3CHCH_3), 24.4 (CH_3CHCH_3), 9.9 (CH_3CH_2). (One Ar-CH signal overlaps with C_6D_6 and is thus not picked); **IR** [cm^{-1}]: ṽ = 3018, 2884, 2954, 2945, 2920, 2859, 2802, 1570, 1540, 1463, 1361, 1320, 1280, 1225, 1193, 1168, 1138, 1103, 1035, 957, 894, 801, 759, 741, 686, 608, 553, 499; **HR-MS-APCI(+)** calc. for $C_{46}H_{55}N_2^+$: 635.4360 $[M+H]^+$; found 635.4352; **UV-vis:** λ_{max}: 381 nm (ε = 9365 cm^{-1} M^{-1}).

Synthesis of dication **4j**

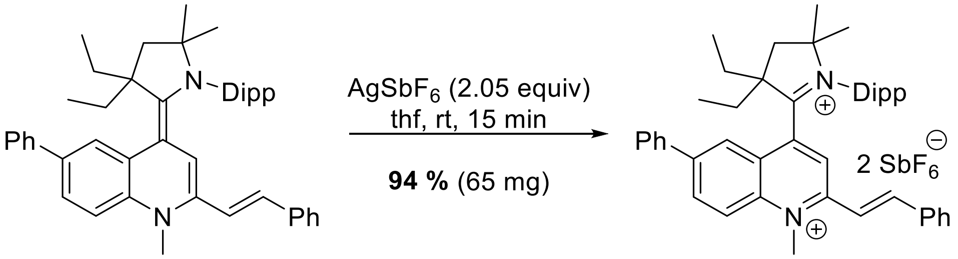

$AgSbF_6$ ($AgSbF_6$, 343.62 g/mol, 44.38 mg, 129.14 µmol, 2.05 equiv) was dissolved in thf (4 mL) and added dropwise to a solution of switch **2j** ($C_{46}H_{54}N_2$, 634.95 g/mol, 40.0 mg, 63.0 µmol, 1.0 equiv) in thf (4 mL). The dark yellow solution was stirred for 15 minutes and then filtrated over a celite plug (1.5 cm). Then the solvent was evaporated until ca. 1 mL of solvent was left. Pentane (18 mL) was added to form a yellow precipitate. The solvent was decanted off and the remaining solid was washed with pentane (2 x 8 mL). Drying under reduced pressure yielded dication **4j** ($C_{46}H_{54}F_{12}N_2Sb_2$, 1106.45 g/mol, 65.2 mg, 58.93 µmol, 94 %) as a yellow solid.

m.p. 189 °C (decomp.); **^{1}H-NMR** (500 MHz, CD_3CN, 298K): δ [ppm] = 8.58 (dd, *J* = 9.3, 1.9 Hz, 1H, Ar-CH), 8.52 (d, *J* = 9.4 Hz, 1H Ar-CH), 8.21 (d, *J* = 2.0 Hz, 1H Ar-CH), 7.98 – 7.91 (m, 3H Ar-CH), 7.89 – 7.81 (m, 3H Ar-CH), 7.72 – 7.67 (m, 2H Ar-CH), 7.65 – 7.57 (m, 5H Ar-CH), 7.45 (t, *J* = 7.8 Hz, 1H Ar-CH), 7.38 (dd, *J* = 7.9, 1.6 Hz, 1H Ar-CH), 7.23 (dd, *J* = 7.8, 1.6 Hz, 1H Ar-CH), 4.47 (s, 3H, N-CH_3), 2.97 – 2.82 (m, 3H, CH_2+CH_3CHCH_3), 2.72 (hept, *J* = 6.6 Hz, 1H, CH_3CHCH_3), 2.51 – 2.17 (m, 4H, CH_2CH_3), 1.71 (s, 3H, CH_3), 1.68 (s, 3H, CH_3), 1.44 (d, *J* = 6.2 Hz, 3H, CH_3CHCH_3), 1.20 (d, *J* = 6.4 Hz, 3H, CH_3CHCH_3), 1.17 – 1.05 (m, 6H, CH_3CHCH_3+CH_2CH_3), 0.73 (t, *J* = 7.4 Hz, 3H, CH_2CH_3), 0.09 (d, *J* = 6.5 Hz, 3H, CH_3CHCH_3); **^{13}C NMR** (126 MHz, CD_3CN, 298K): δ [ppm] = 198.0 (Iminium-C_q), 156.4 (C_q), 150.5 (Ar-CH), 145.7 (C_q), 145.5 (C_q), 143.9 (C_q), 141.0 (C_q), 140.3 (C_q), 137.5 (C_q), 137.0 (Ar-CH), 135.2 (C_q), 133.5 (Ar-CH), 133.0 (Ar-CH), 130.9 (Ar-CH), 130.7 (Ar-CH), 130.4 (Ar-CH), 130.3 (Ar-CH), 128.8 (Ar-CH), 128.1 (Ar-CH), 128.0 (Ar-CH), 125.2 (C_q), 124.8 (Ar-CH), 122.1 (Ar-CH), 122.0 (Ar-CH), 119.2 (Ar-CH), 88.0 (C_q), 62.2 (C_q), 45.2 (CH_2), 42.2 (N-CH_3), 31.5 (CH_2CH_3), 30.9 (CH_2CH_3), 30.7 (CH_3CHCH_3), 30.5 (CH_3), 30.5 (CH_3CHCH_3), 29.1 (CH_3), 27.8 (CH_3CHCH_3), 27.1 (CH_3CHCH_3), 25.3 (CH_3CHCH_3), 24.7 (CH_3CHCH_3), 9.3 (CH_2CH_3), 9.1 (CH_2CH_3); **IR** [cm^{-1}]: ṽ = 2969, 2940, 1615, 1589, 1565, 1491, 1459, 1447, 1388, 1366, 1333, 1210, 1177, 1132, 1114, 971, 768, 754, 692, 652; **HR-MS-ESI(+)** calc. for $C_{46}H_{54}N_2^{2+}$: 317.2138 $[M]^{2+}$; found 317.2135; **UV-vis:** λ_{max}: 424 nm (ε = 38994 cm^{-1} M^{-1}).

NMR Spectra

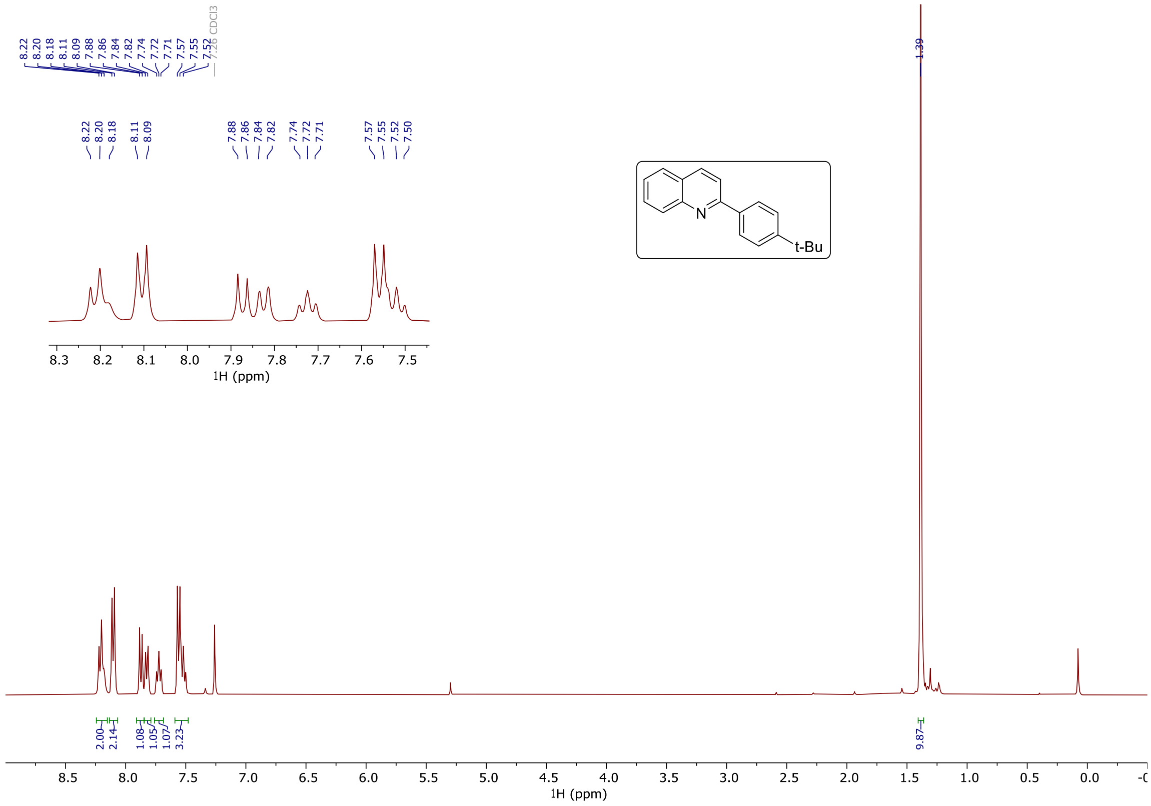

Figure S1: ^{1}H NMR (400 MHz, $CDCl_3$, 298 K) of **S1**.

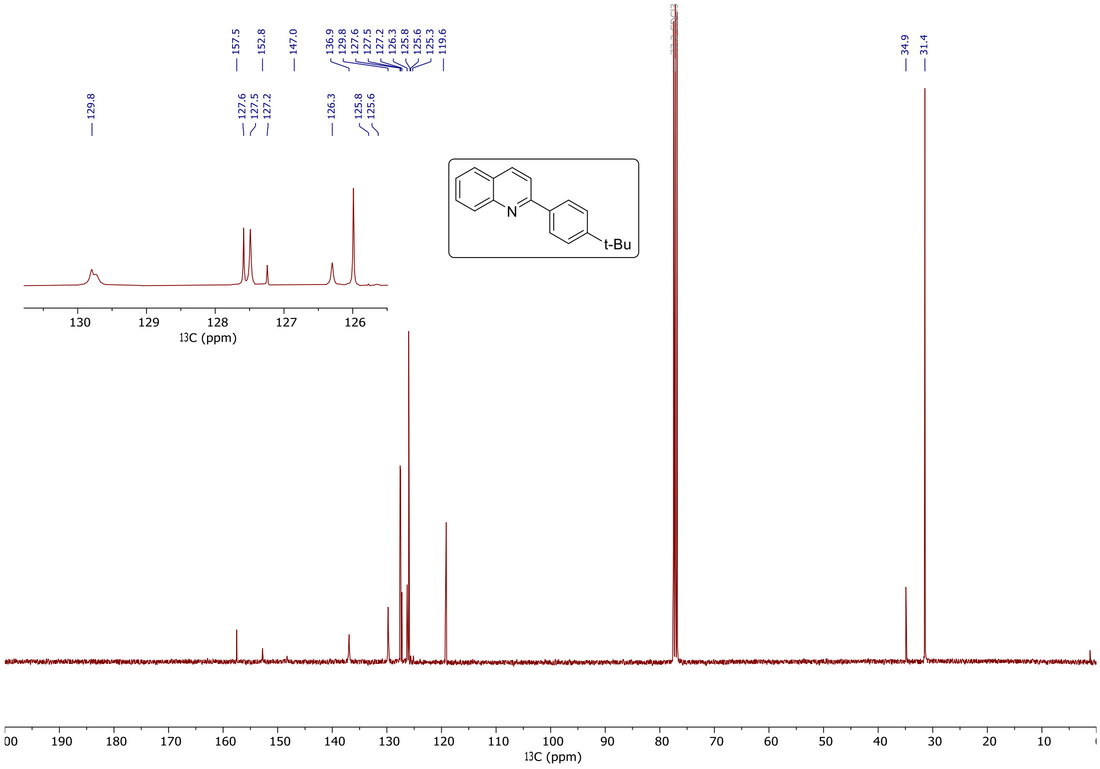

Figure S2: ^{13}C {^{1}H} NMR (101 MHz, $CDCl_3$, 298K) of **S1**.

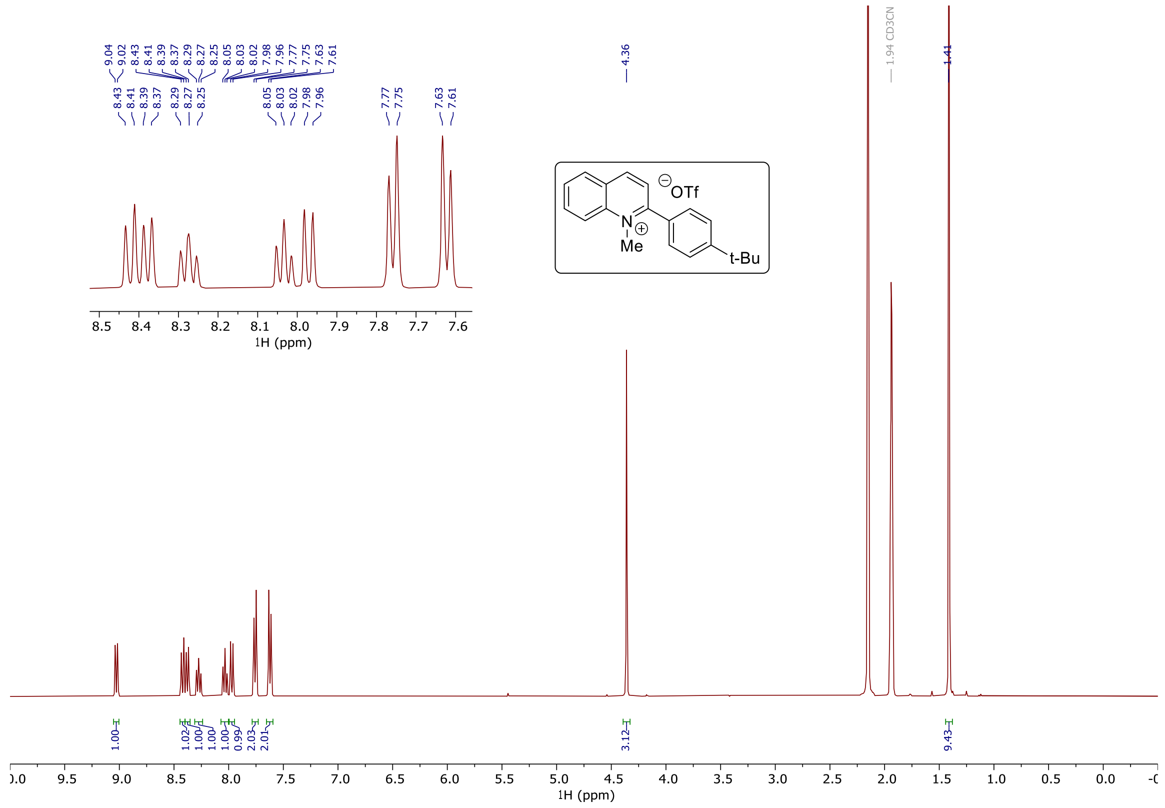

Figure S3: ^{1}H NMR (300 MHz, CD_3CN, 298 K) of **1a.**

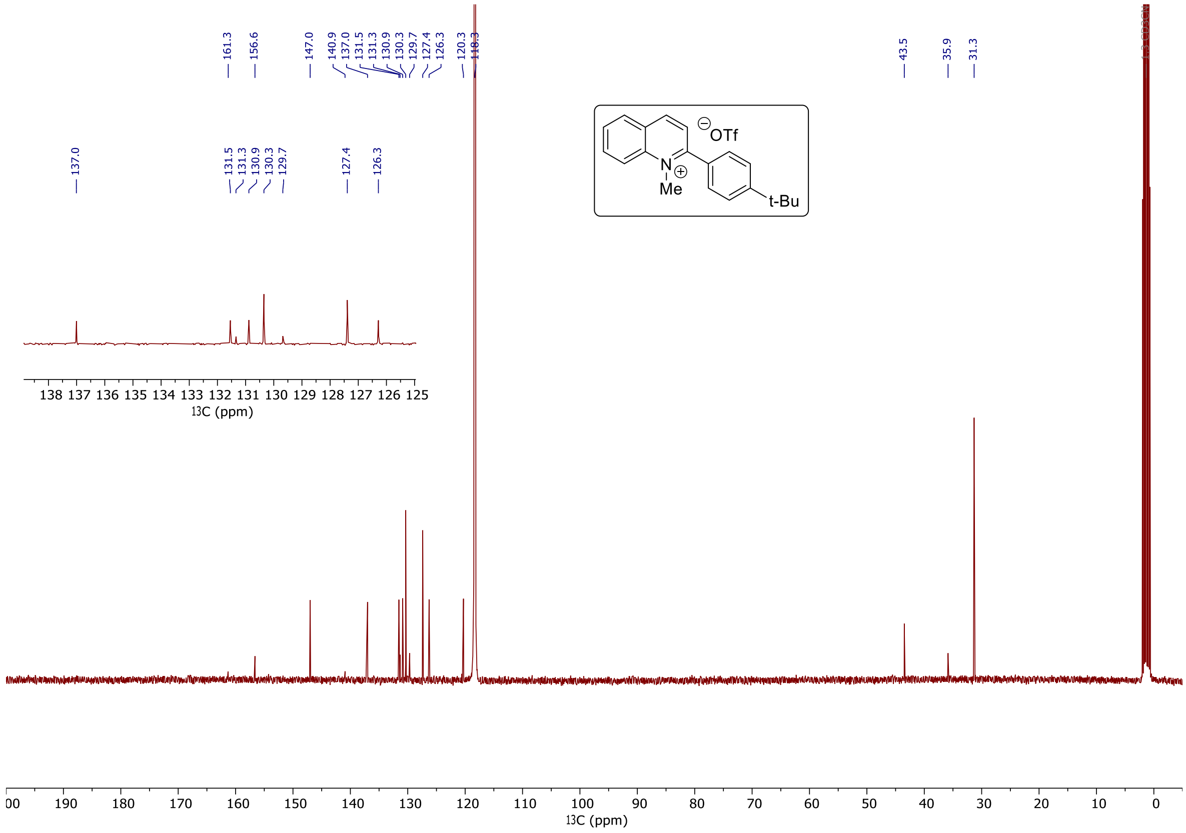

Figure S4: ^{13}C {^{1}H} NMR (101 MHz, CD_3CN, 298K) of **1a**.

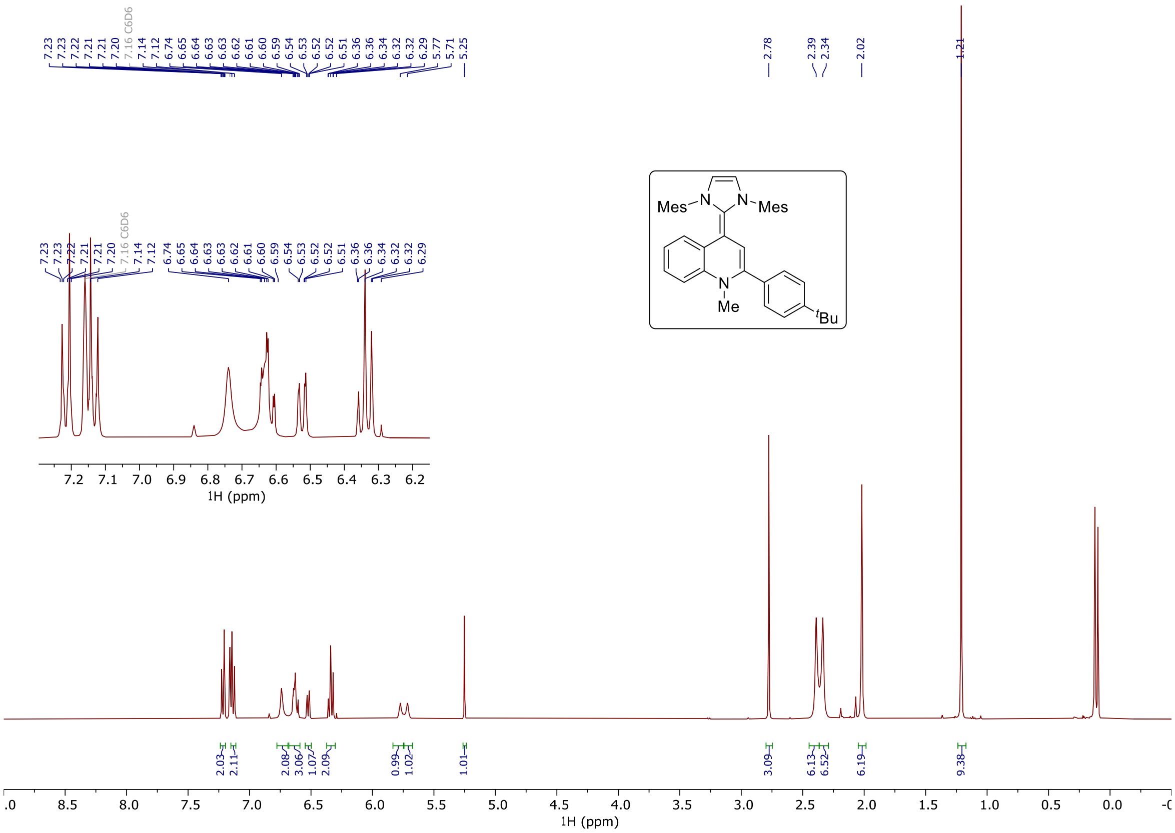

Figure S5: ^{1}H NMR (400 MHz, C_6D_6, 298K) of **2a**.

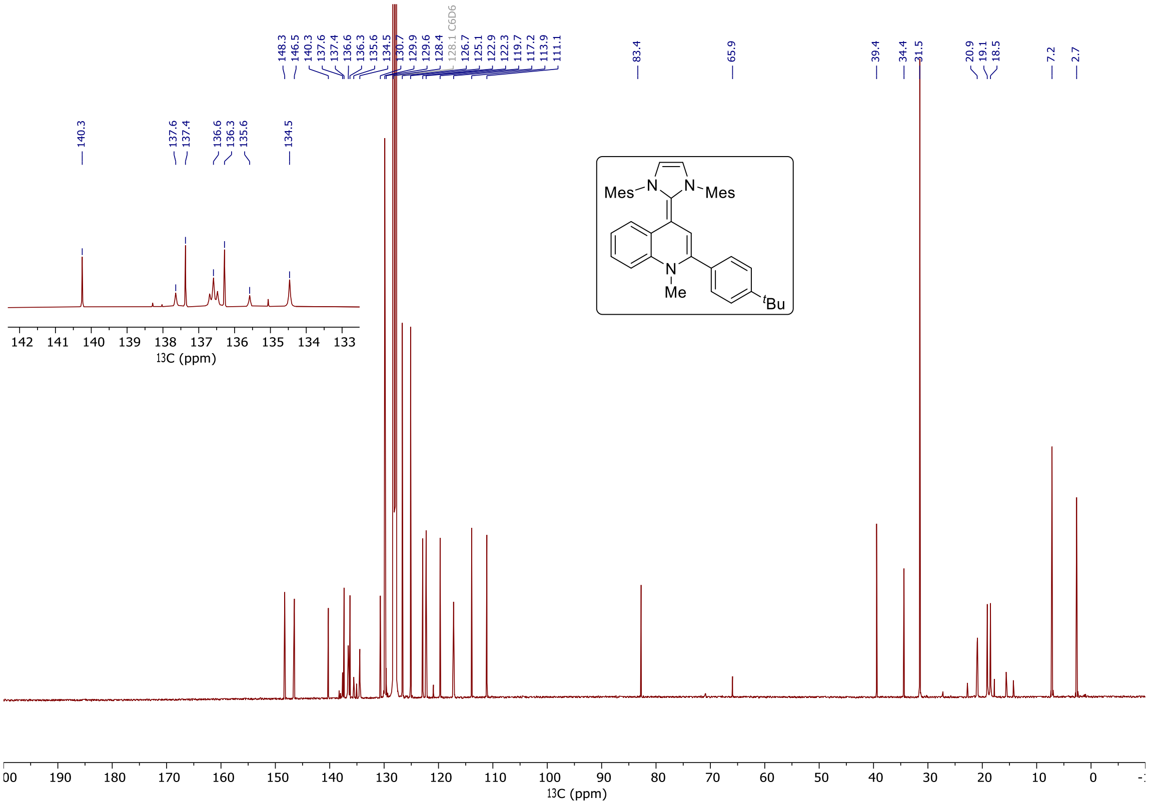

Figure S6: ^{13}C {^{1}H} NMR (126 MHz, C_6D_6, 298K) of **2a**.

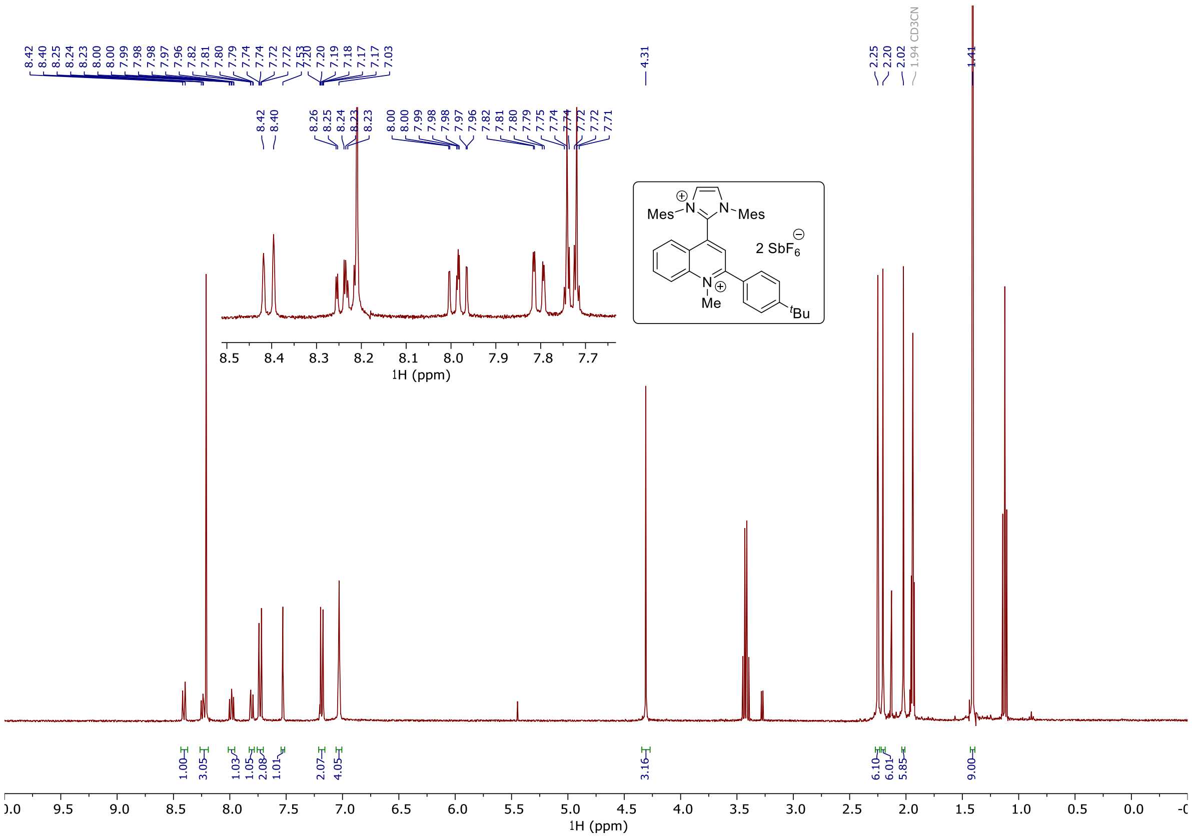

Figure S7: ^{1}H NMR (400 MHz, CD_3CN, 298K) of **4a**.

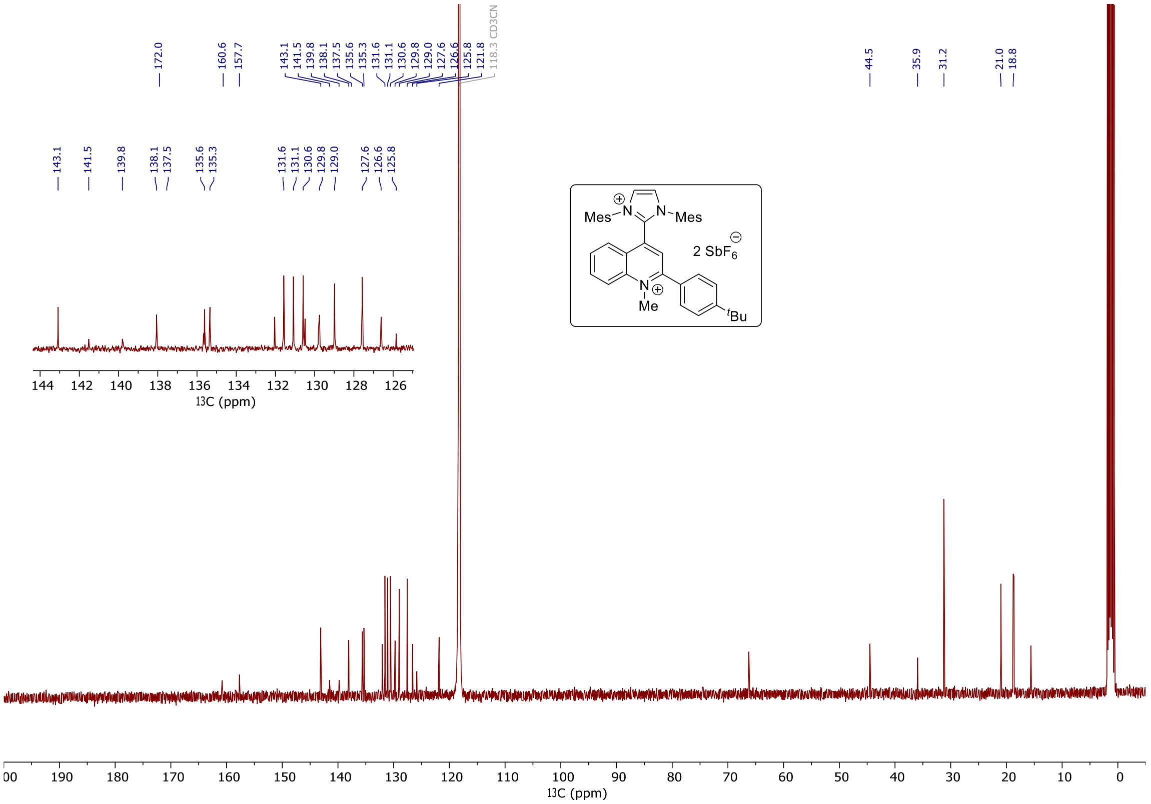

Figure S8: ^{13}C {^{1}H} NMR (101 MHz, CD_3CN, 298K) of **4a**.

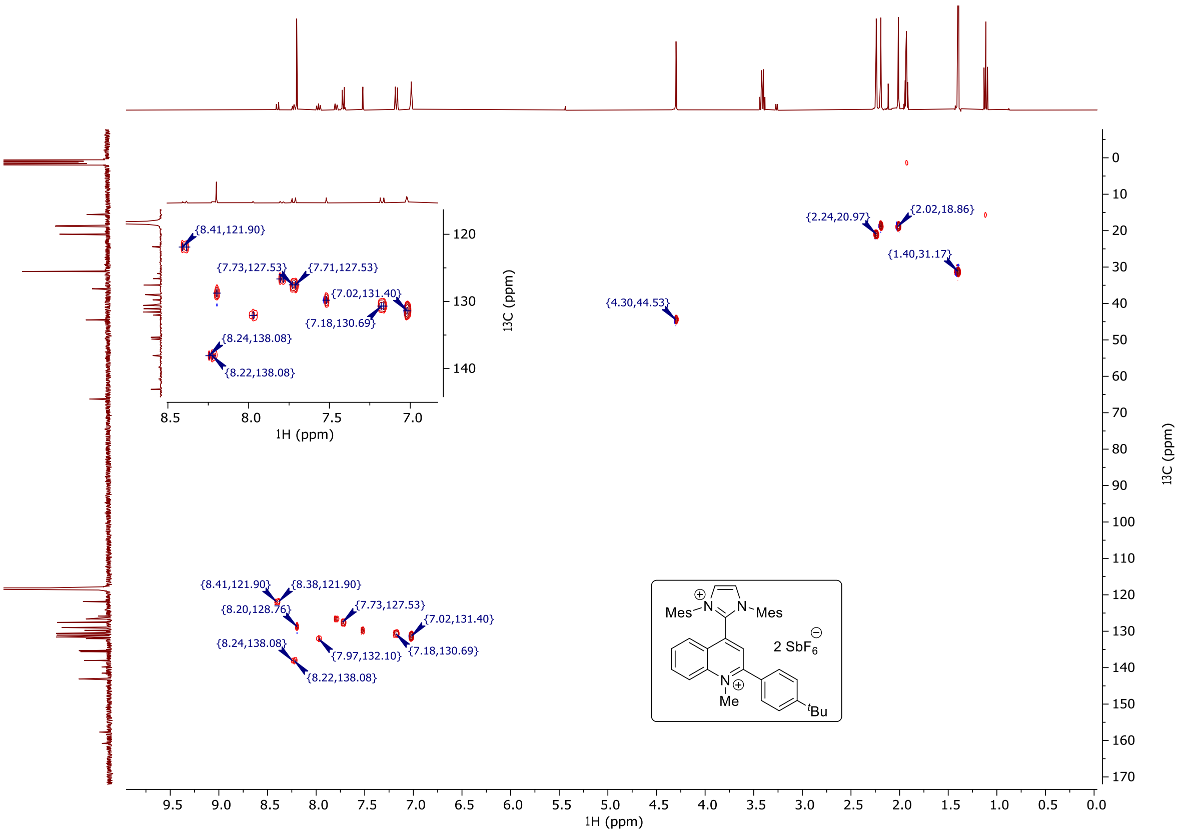

Figure S9: ^{1}H/^{13}C HSQC (400/101 MHz, CD_3CN, 298K) of **4a**.

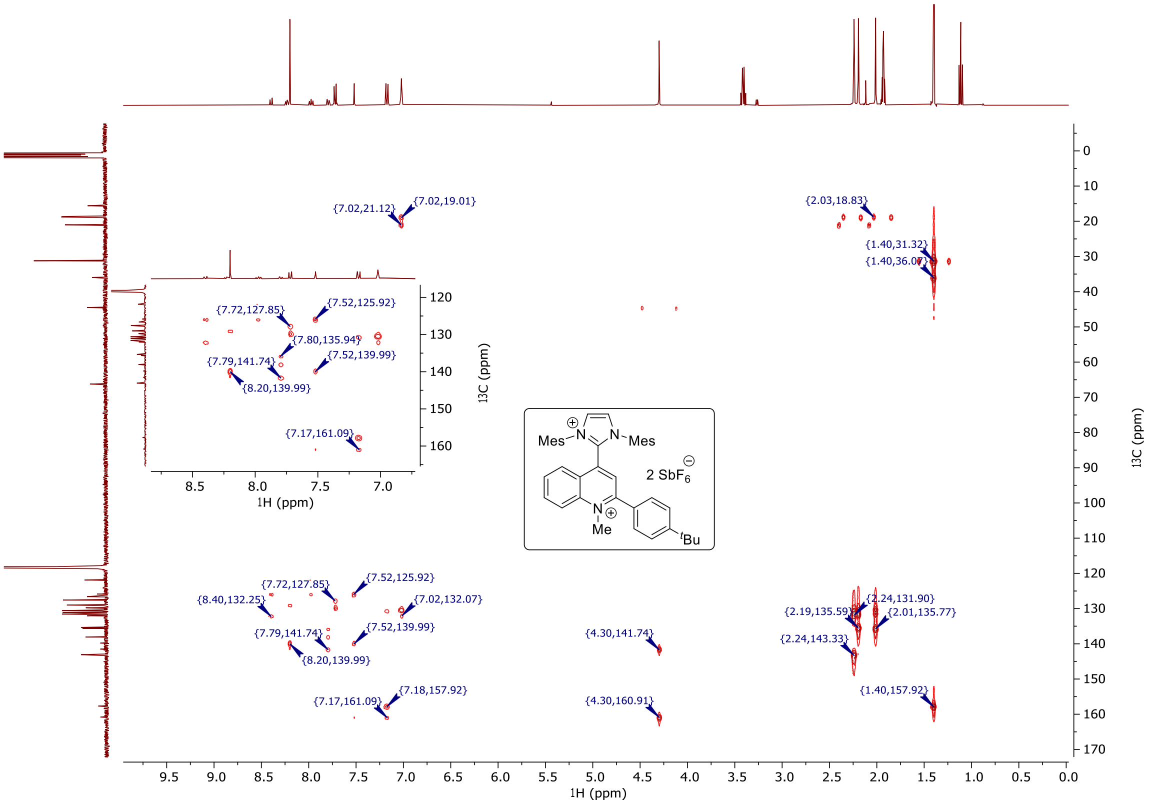

Figure S10: ^{1}H/^{13}C HMBC (400/101 MHz, CD_3CN, 298K) of **4a**.

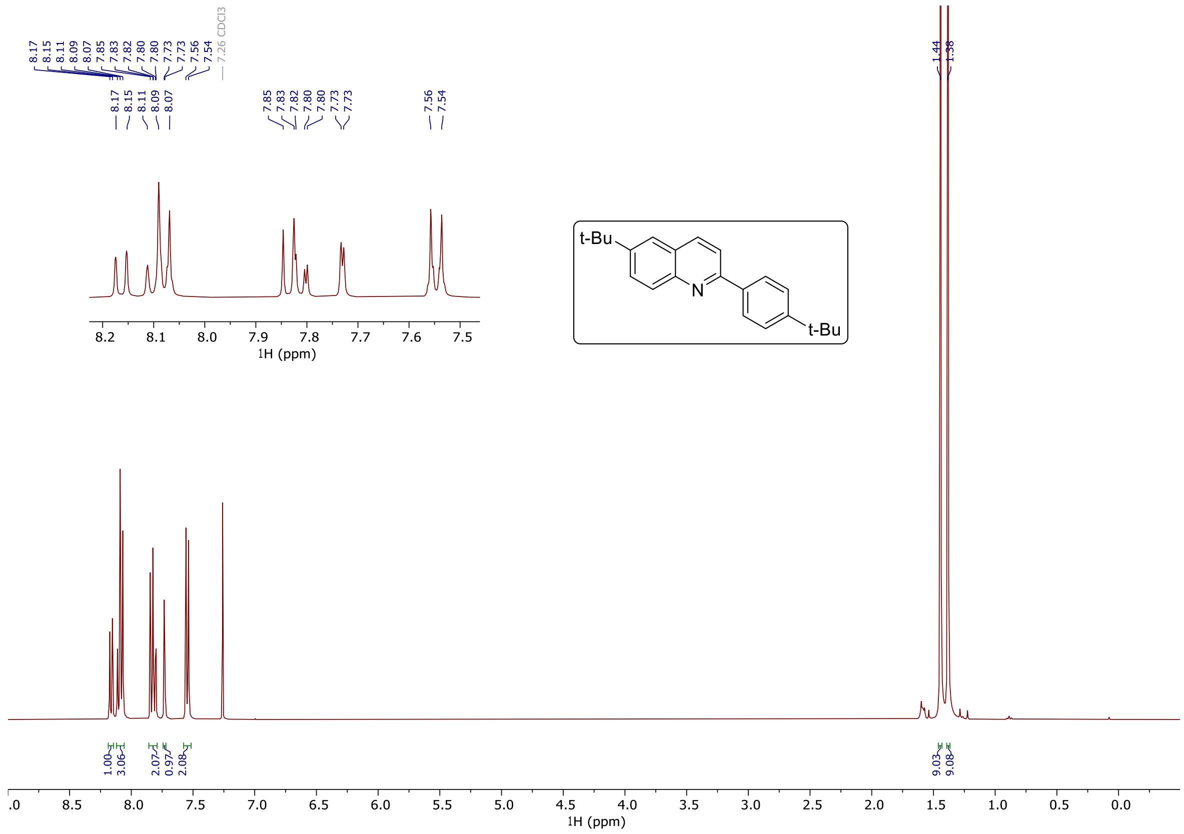

Figure S11: ^{1}H NMR (400 MHz, $CDCl_3$, 298 K) of **S2.**

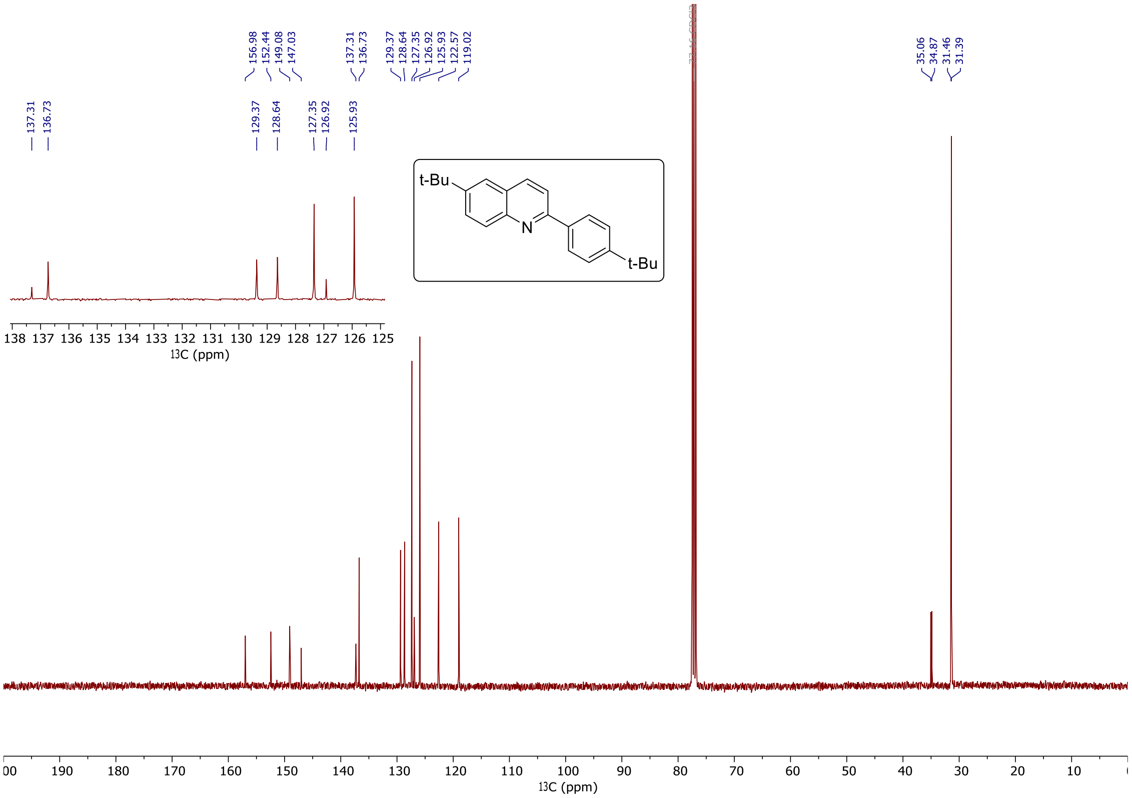

Figure S12: ^{13}C {^{1}H} NMR (101 MHz, $CDCl_3$, 298K) of **S2**.

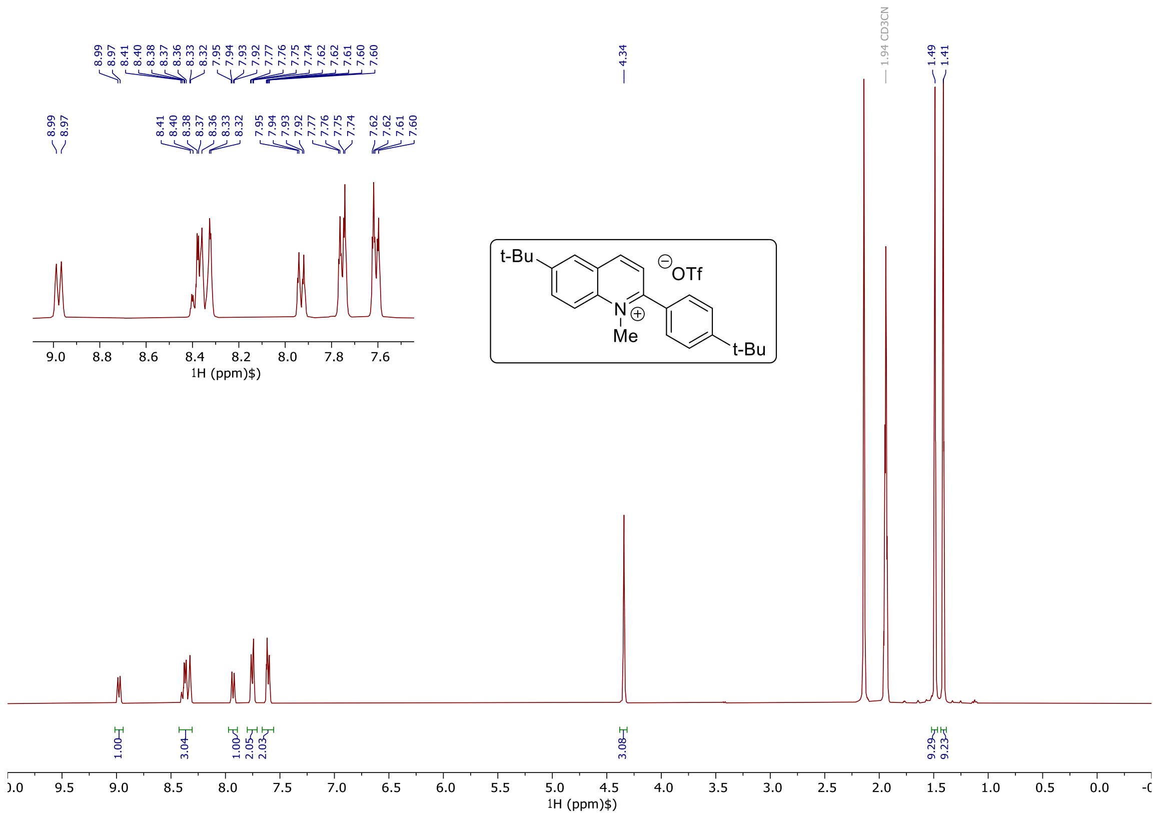

Figure S13: ^{1}**H NMR** (400 MHz, CD_3CN, 298 K) of **1b**.

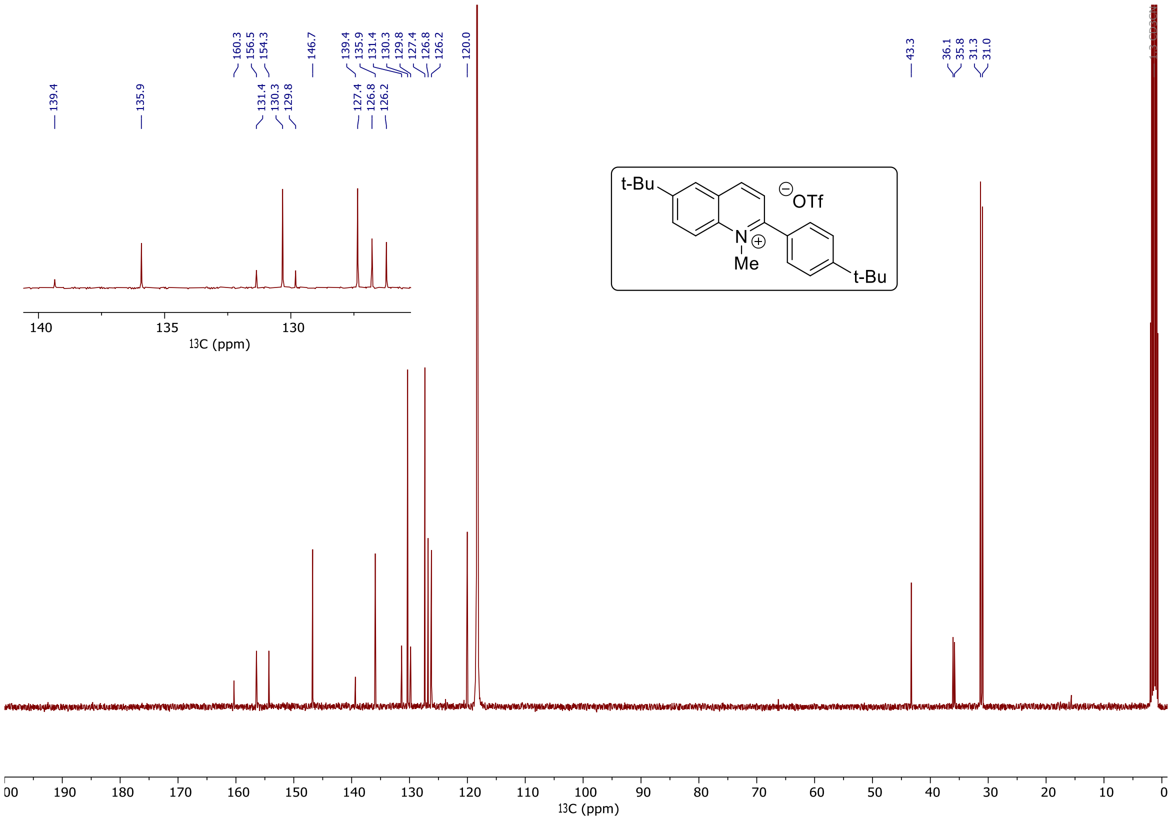

Figure S14: ^{13}**C {**^{1}**H} NMR** (101 MHz, CD_3CN, 298K) of **1b**.

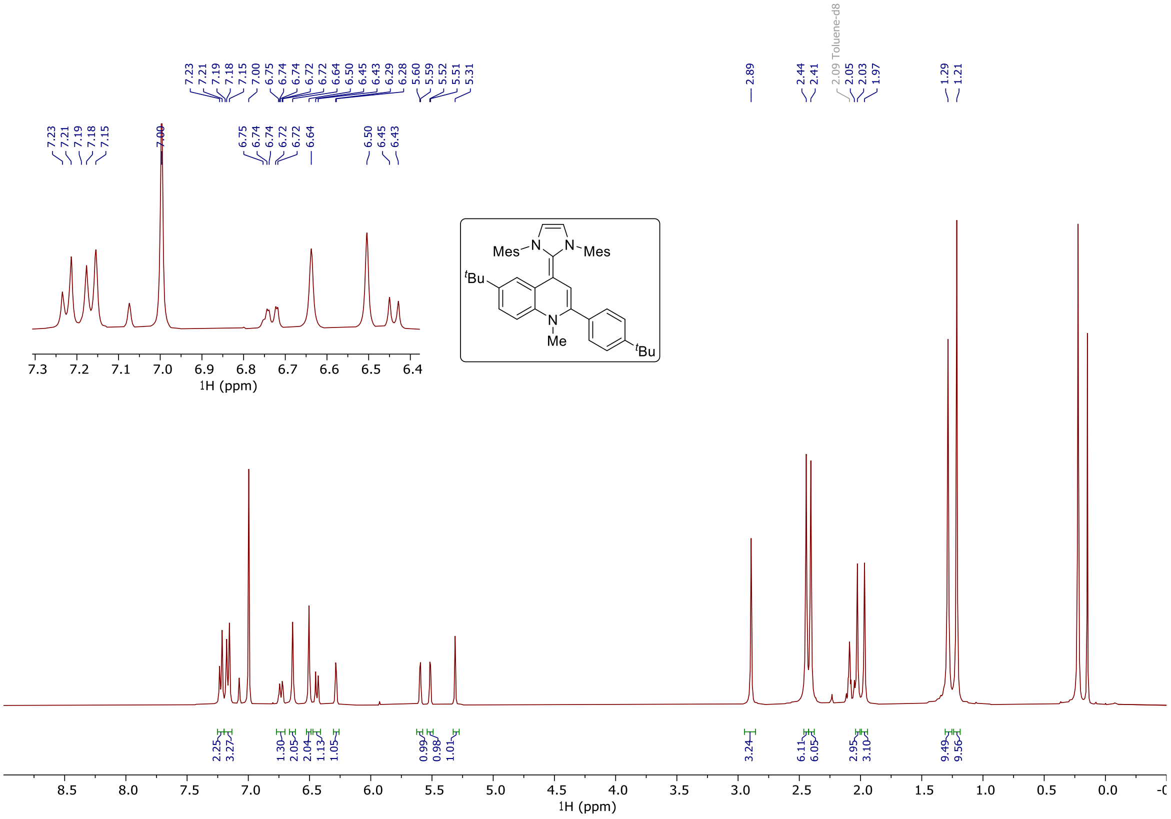

Figure S15: ¹H NMR (400 MHz, toluene-d_8, 298K) of **2b**.

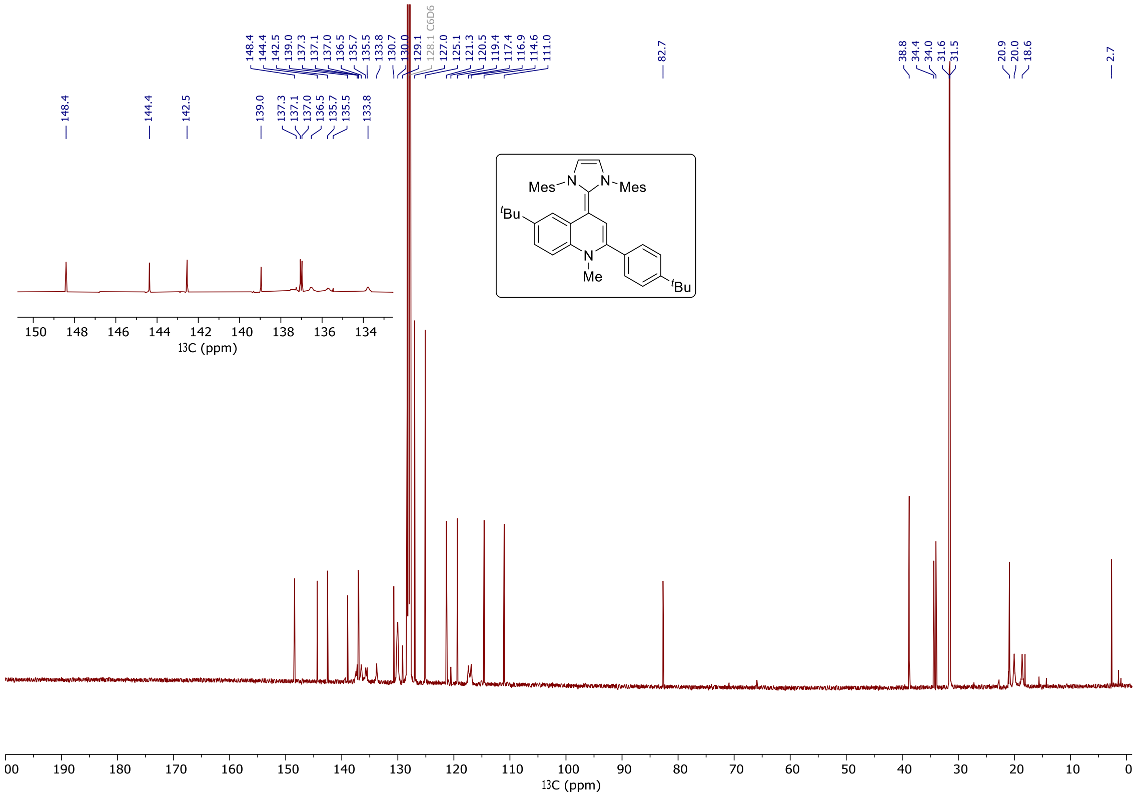

Figure S16: ¹³C {¹H} NMR (101 MHz, C_6D_6, 298K) of **2b**.

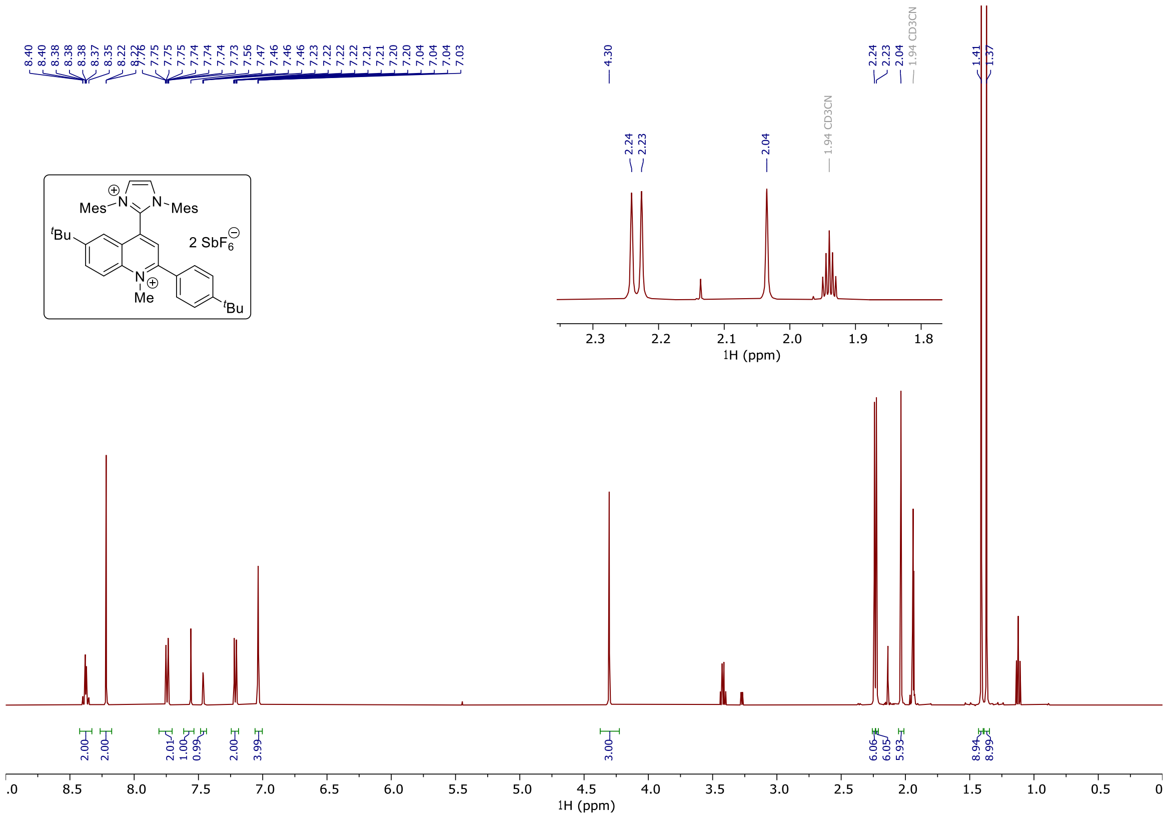

Figure S17: ^{1}H NMR (500 MHz, CD_3CN, 298K) of **4b**.

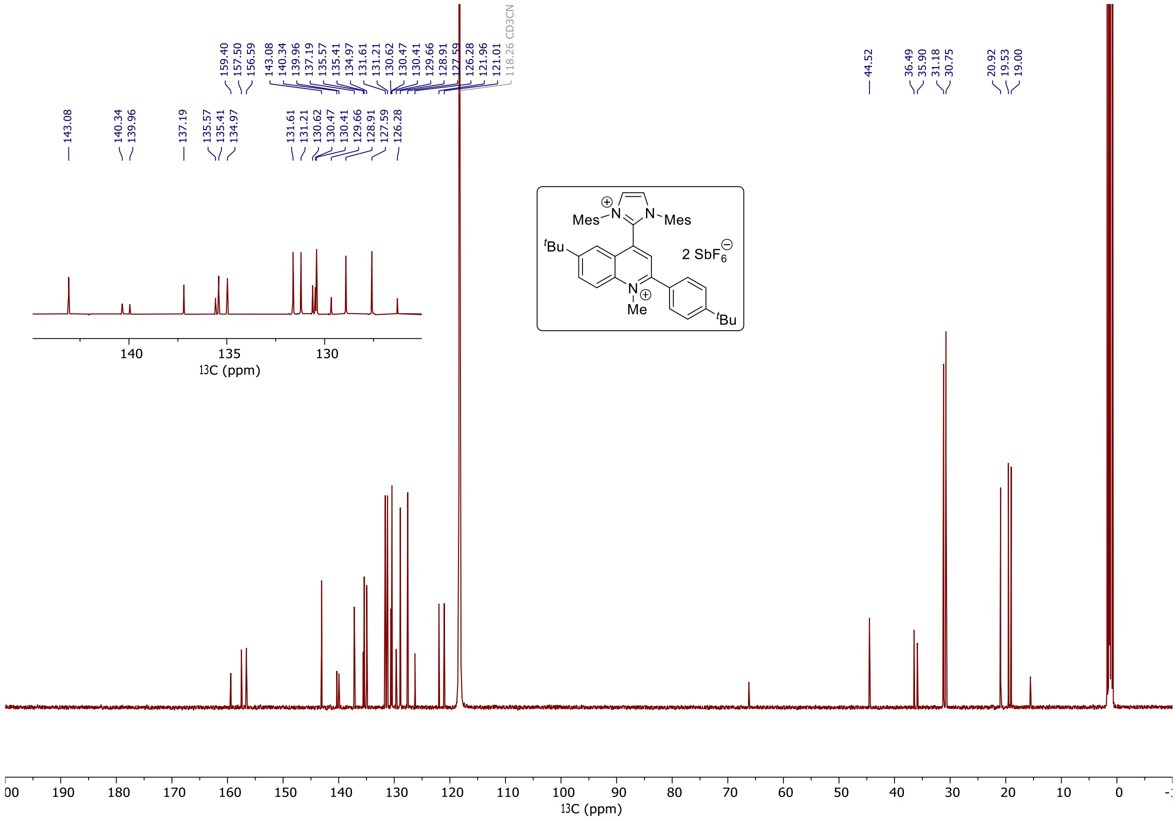

Figure S18: ^{13}C {^{1}H} NMR (126 MHz, CD_3CN, 298K) of **4b**.

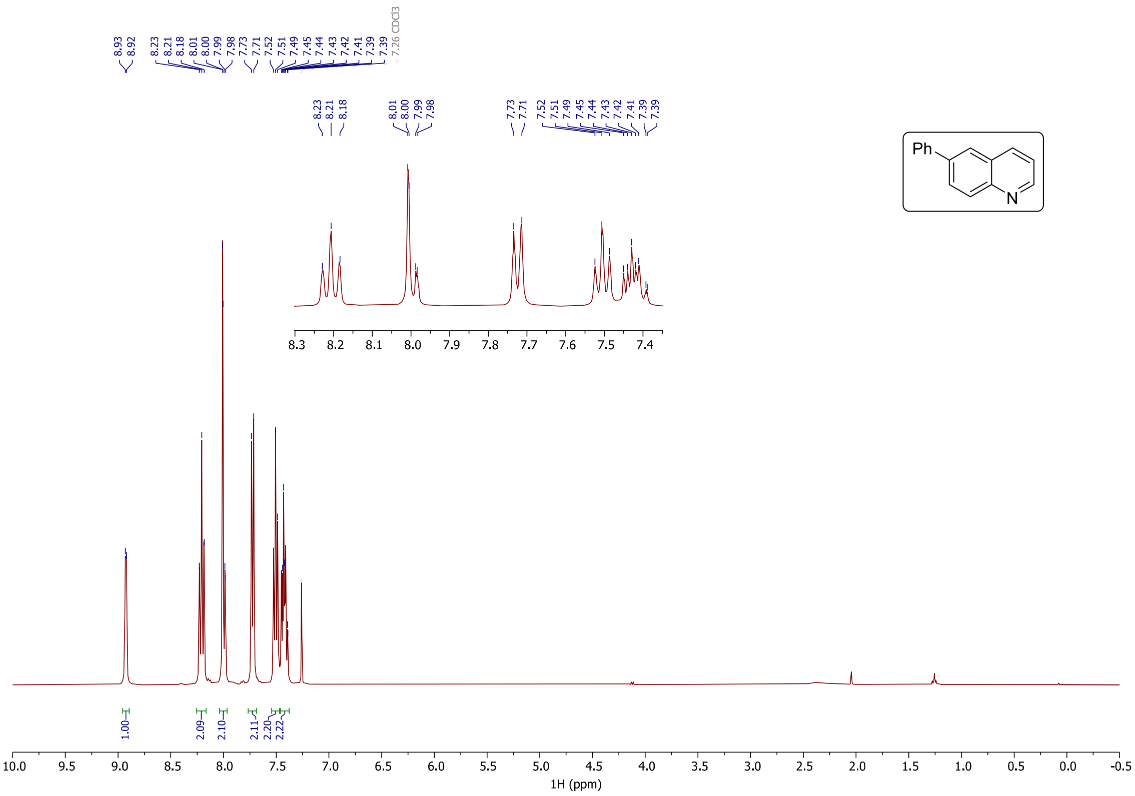

Figure S19: ^{1}H NMR (400 MHz, $CDCl_3$, 298K) of **S3**.

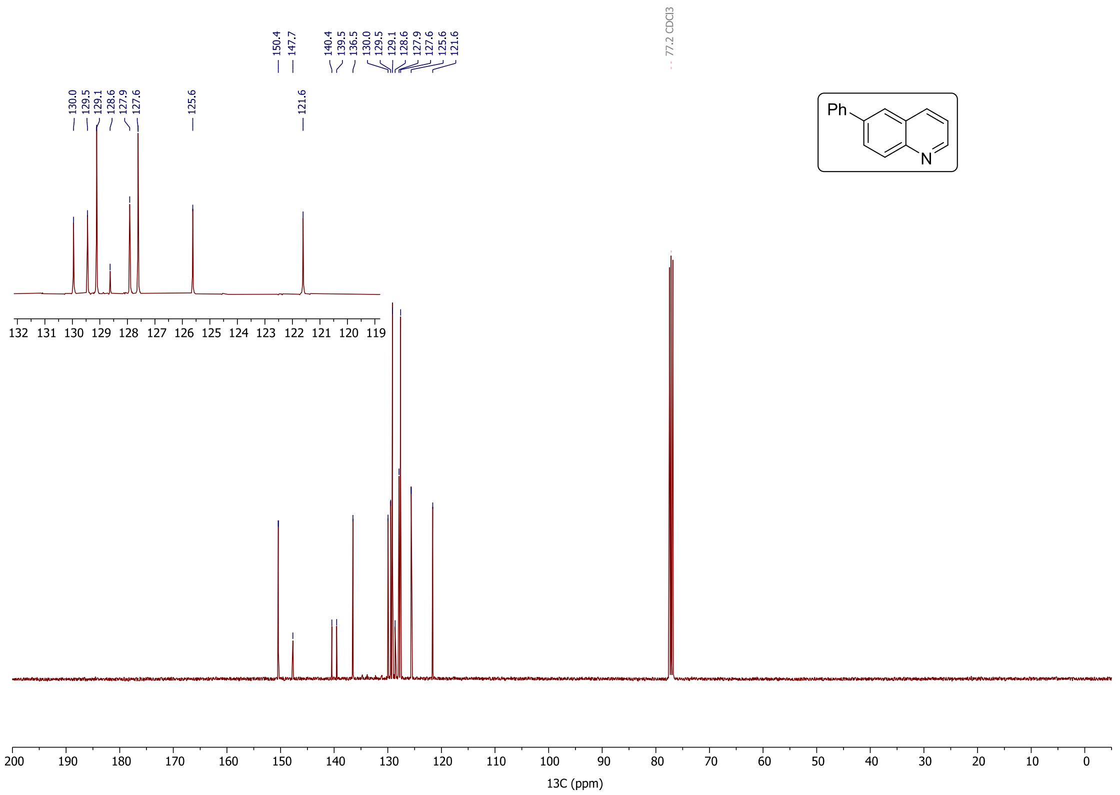

Figure S20: ^{13}C {^{1}H} NMR (101 MHz, $CDCl_3$, 298K) of **S3**.

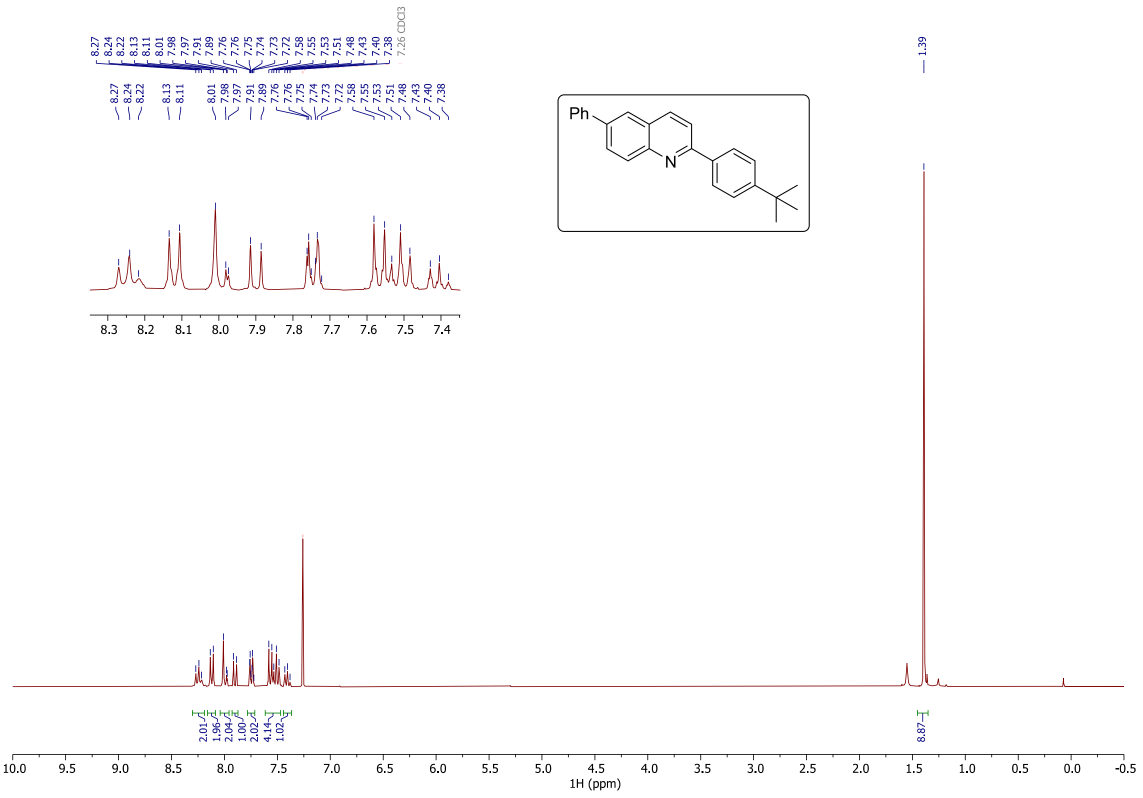

Figure S21: ^{1}H NMR (300 MHz, $CDCl_3$, 298K) of **S4**.

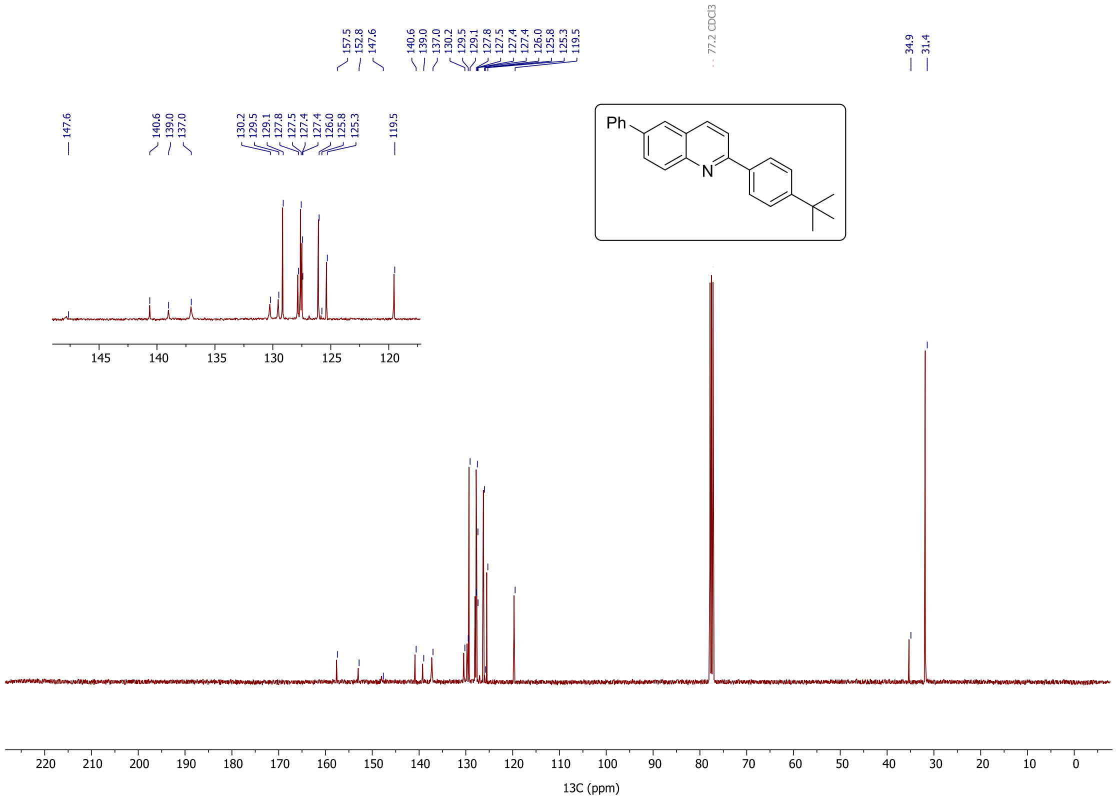

Figure S22: **^{13}C {^{1}H} NMR** (101 MHz, $CDCl_3$, 298K) of **S4**.

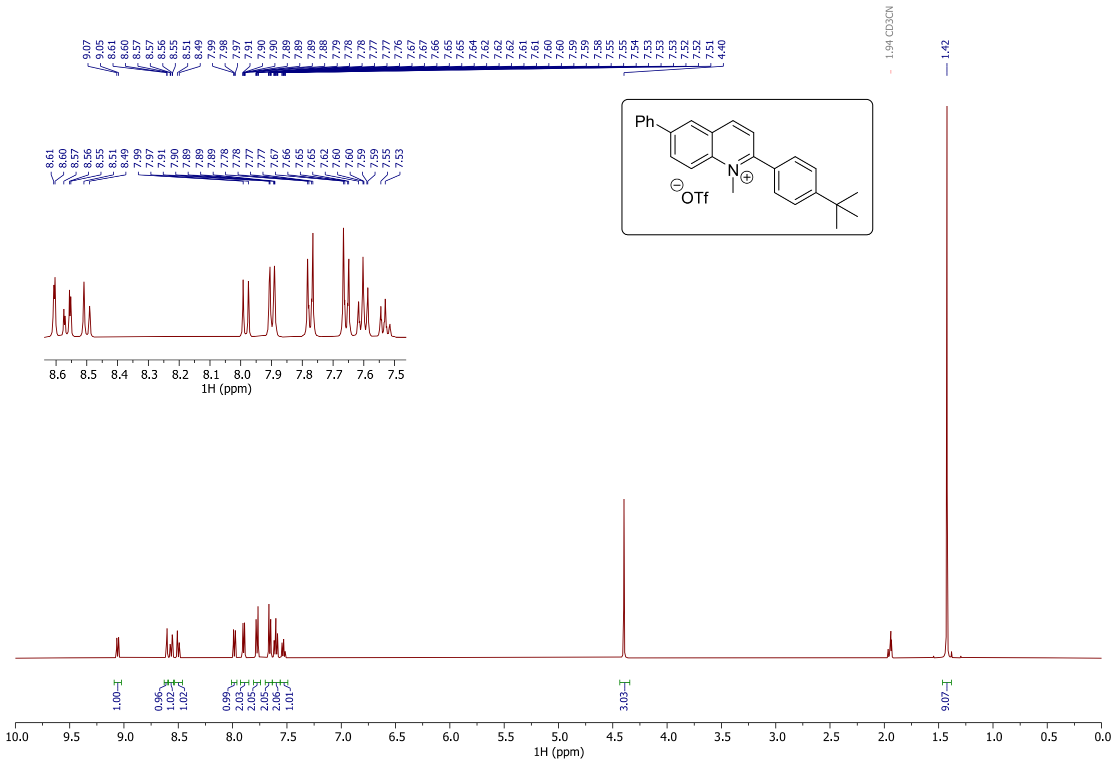

Figure S23: ^{1}**H NMR** (500 MHz, CD_3CN, 298K) of **1c**.

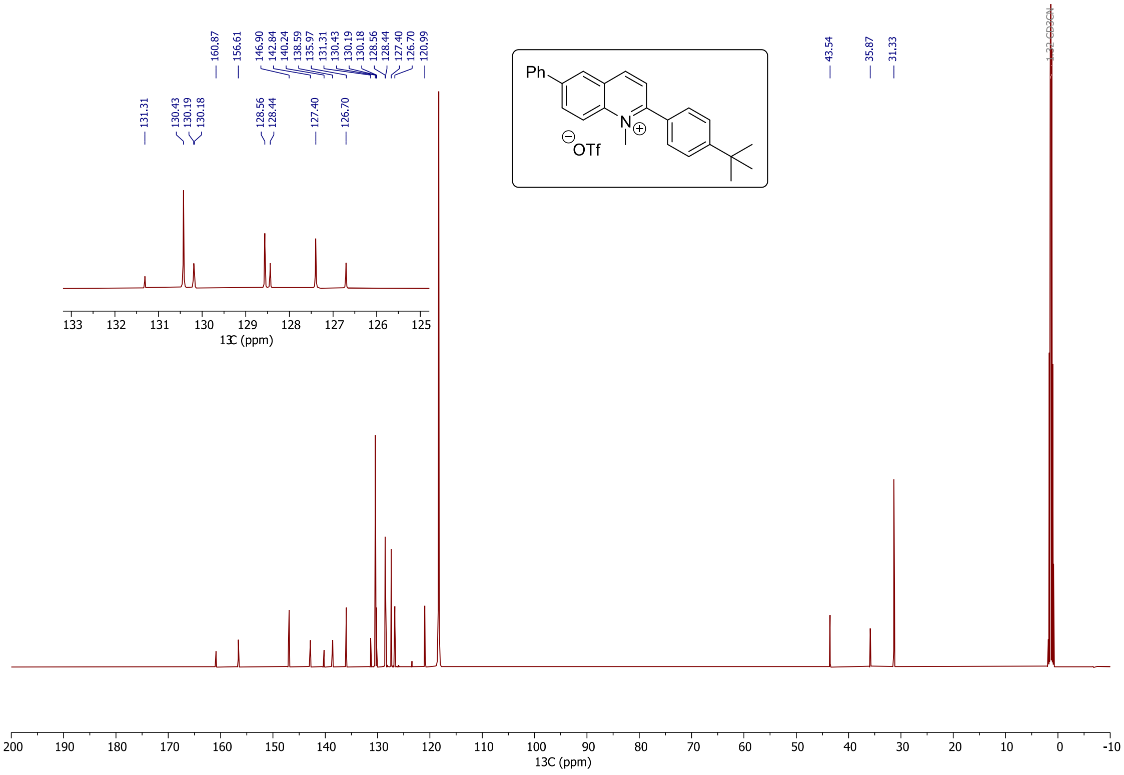

Figure S24: ^{13}**C {^{1}H} NMR** (126 MHz, CD_3CN, 298K) of **1c**.

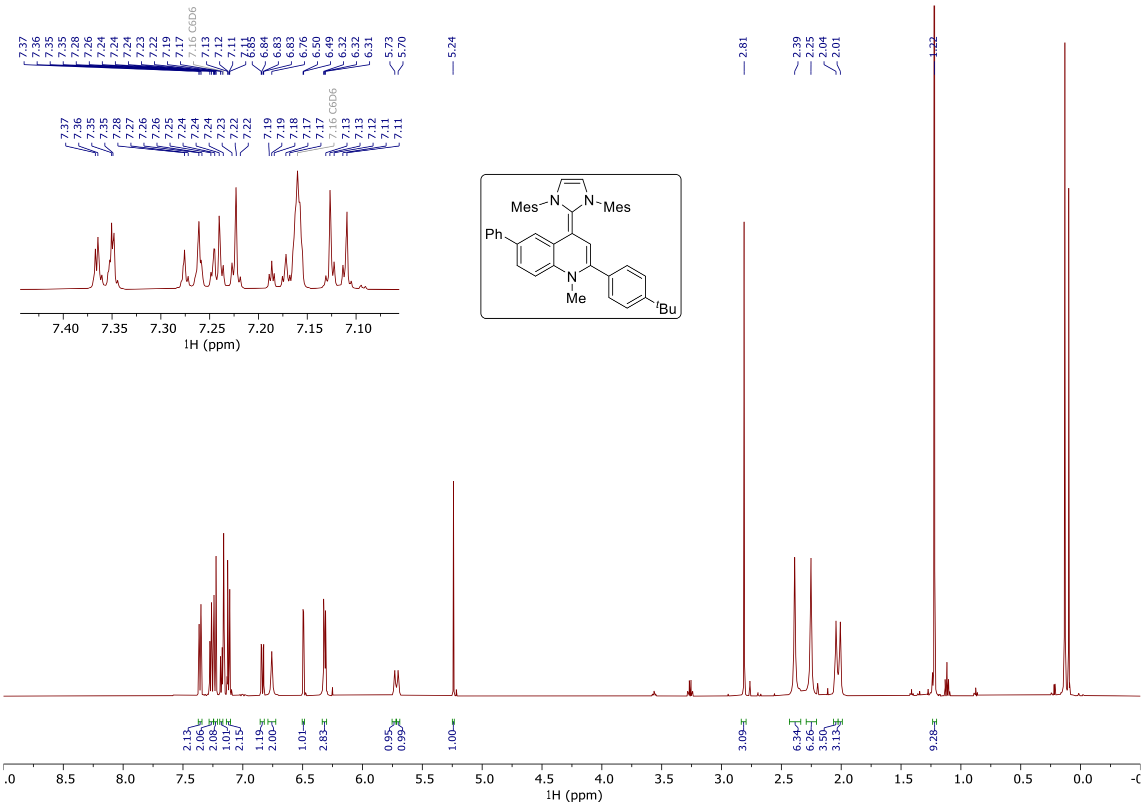

Figure S25: ^{1}H NMR (500 MHz, C_6D_6, 298K) of **2c**.

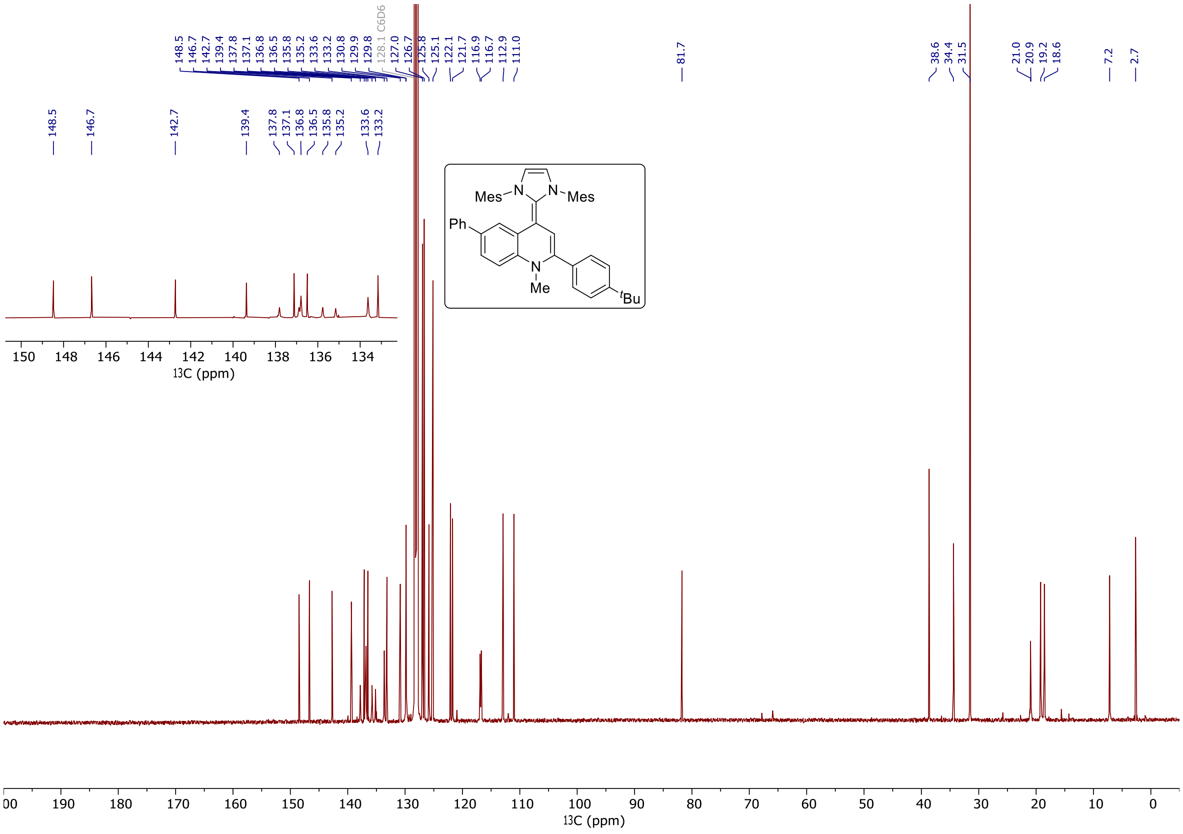

Figure S26: ^{13}C {^{1}H} NMR (126 MHz, C_6D_6, 298K) of **2c**.

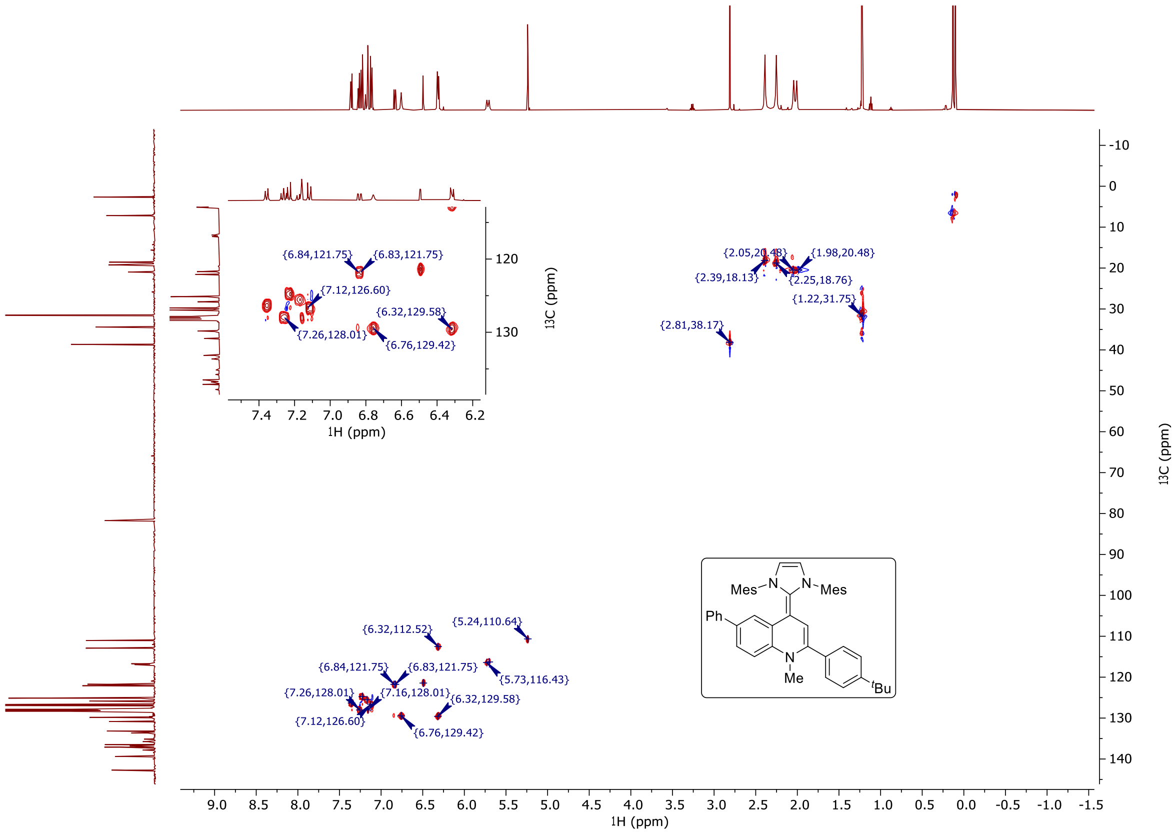

Figure S27: ^{1}H/^{13}C HSQC (500/126 MHz, C_6D_6, 298K) of **2c**.

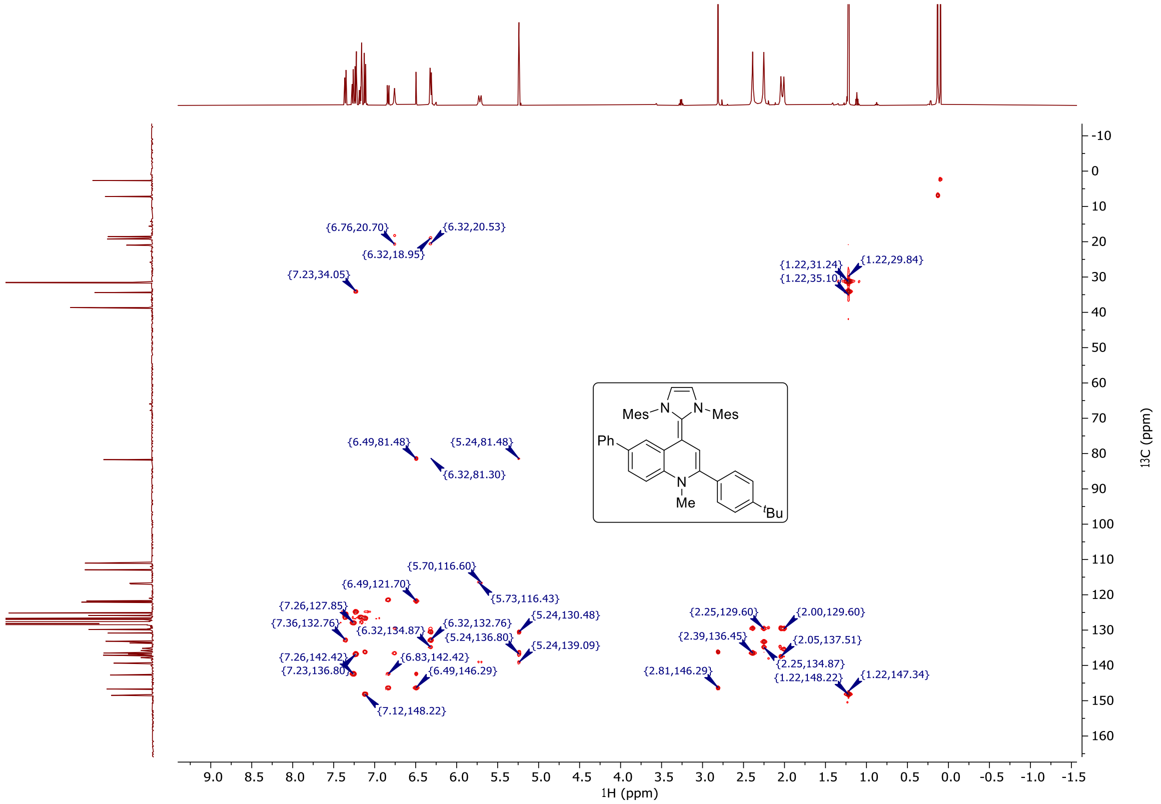

Figure S28: ^{1}H/^{13}C HMBC (500/126 MHz, C_6D_6, 298K) of **2c**.

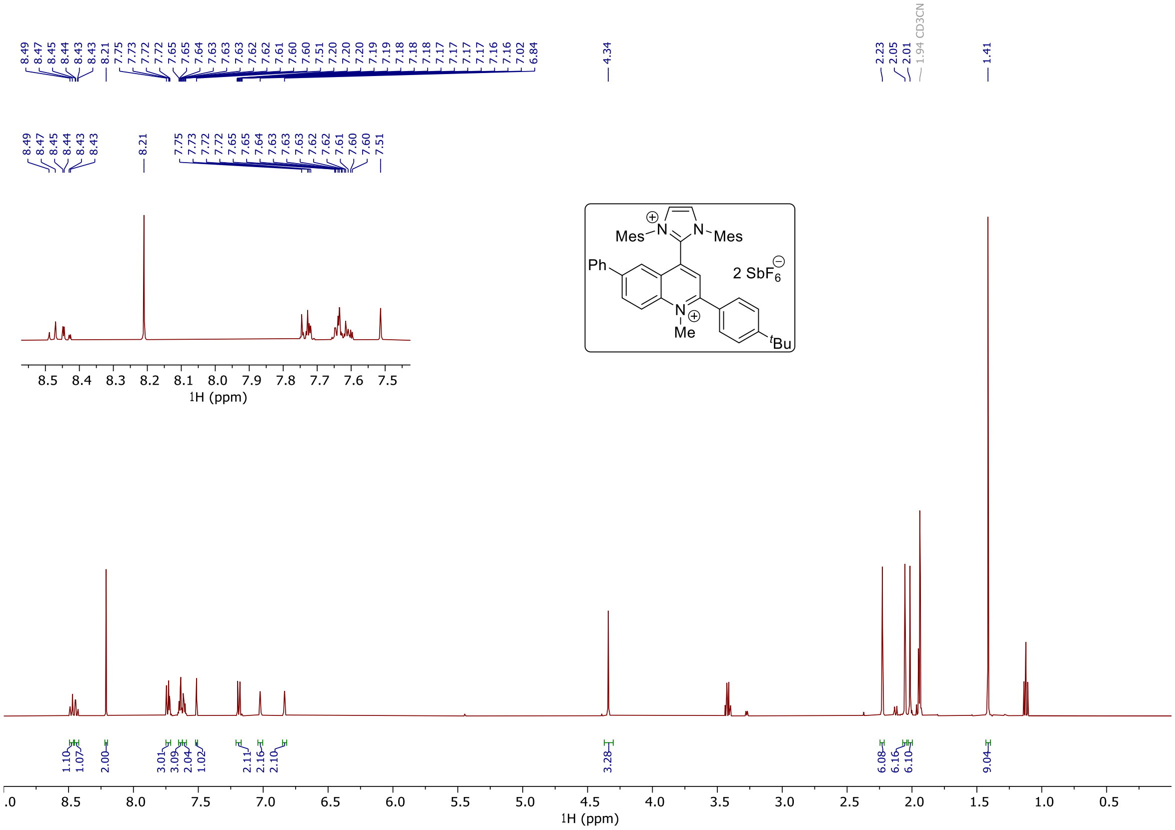

Figure S29: ^{1}H NMR (500 MHz, CD_3CN, 298K) of **4c**.

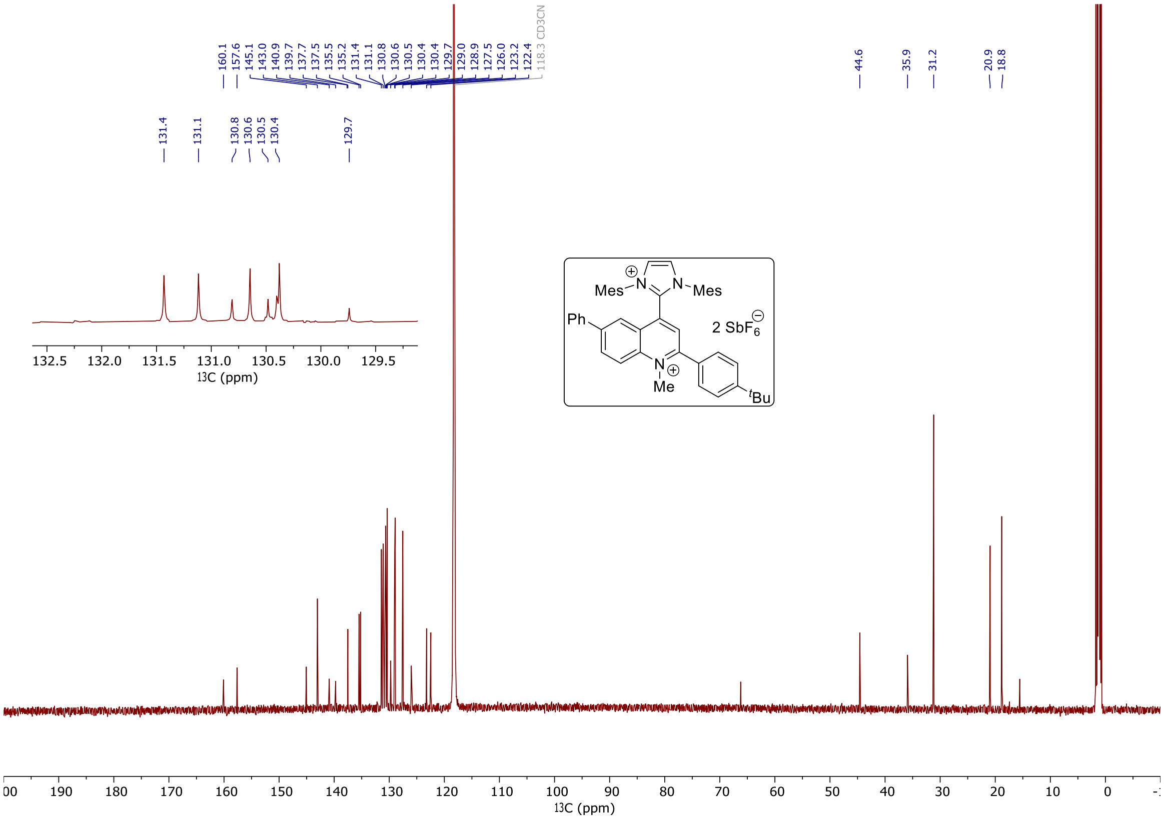

Figure S30: ^{13}C {^{1}H} NMR (126 MHz, CD_3CN, 298K) of **4c**.

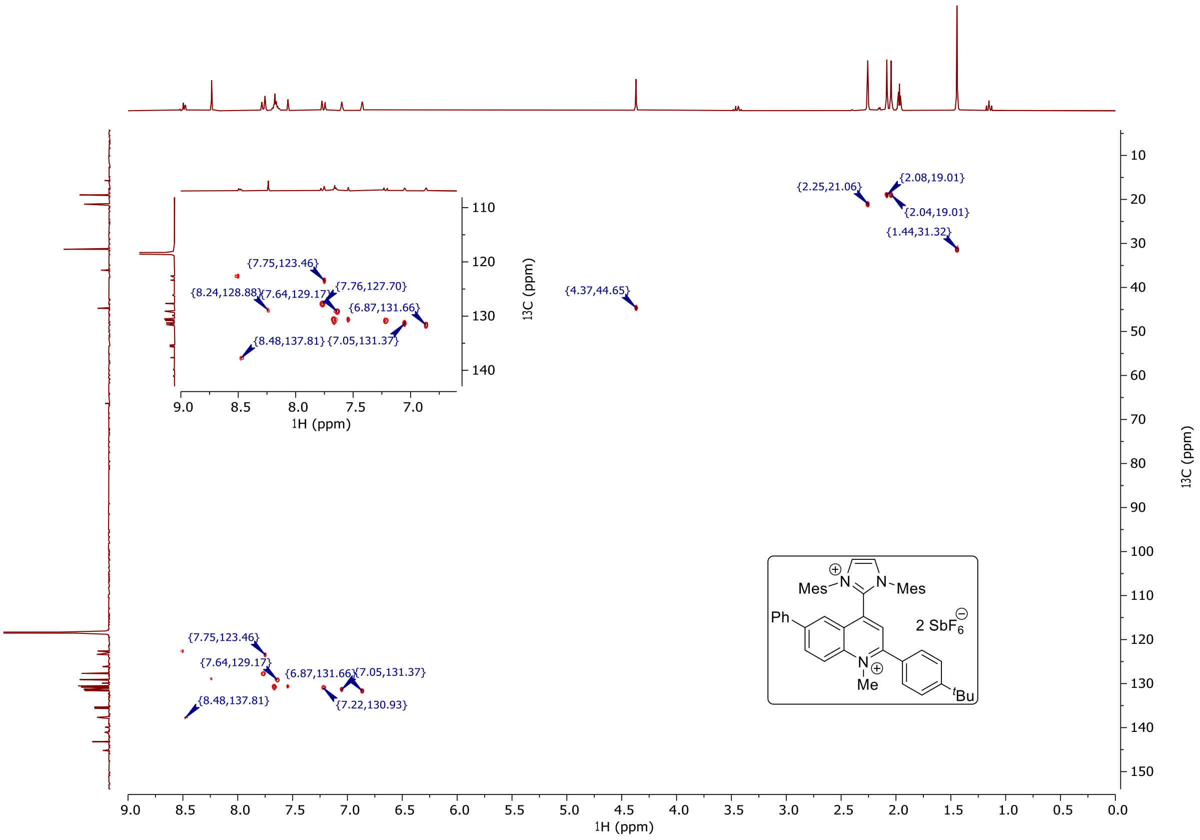

Figure S31: ^{1}H/^{13}C HSQC (500/126 MHz, CD_3CN, 298K) of **4c**.

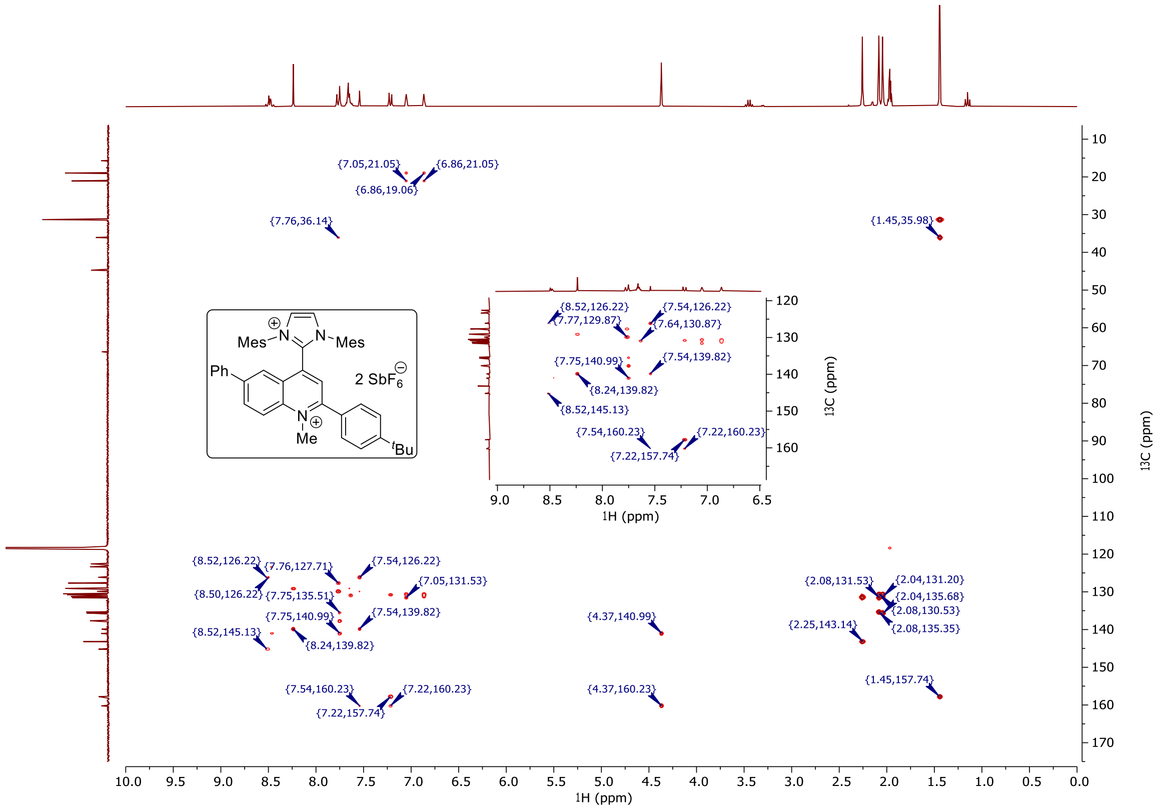

Figure S32: ^{1}H/^{13}C HMBC (500/126 MHz, CD_3CN, 298K) of **4c**.

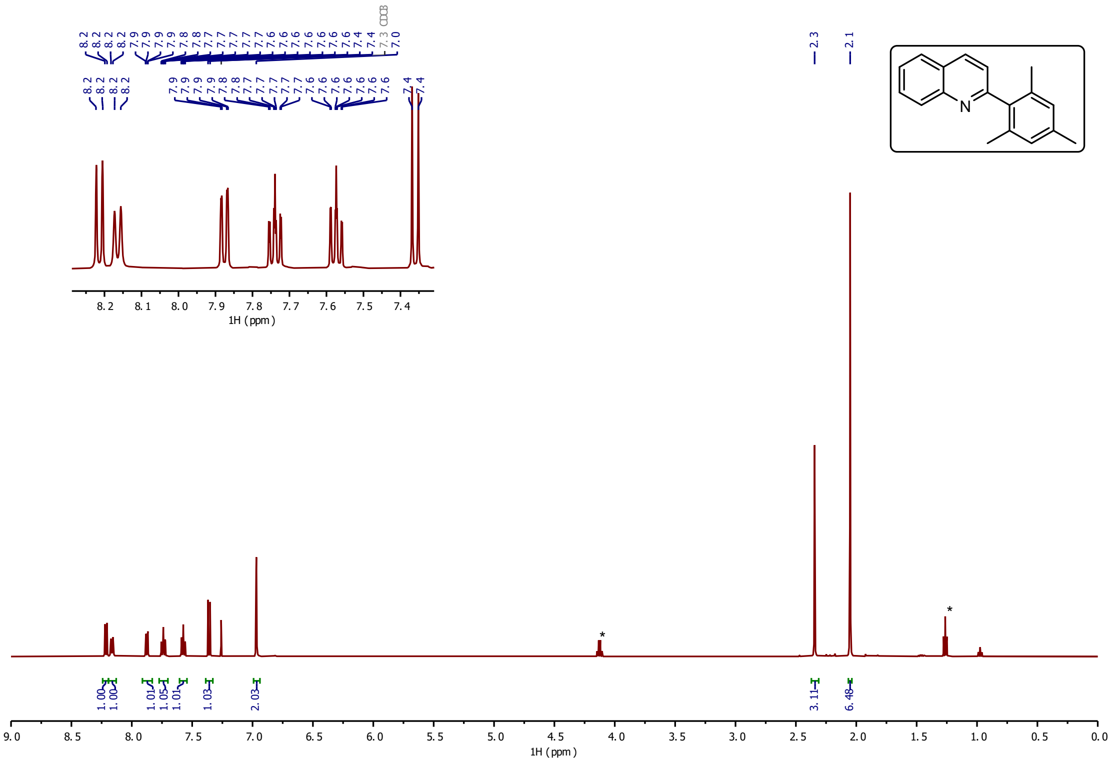

Figure S33: **^{1}H NMR** (500 MHz, $CDCl_3$, 298K) of **S5**, residual Et_2O is marked with *.

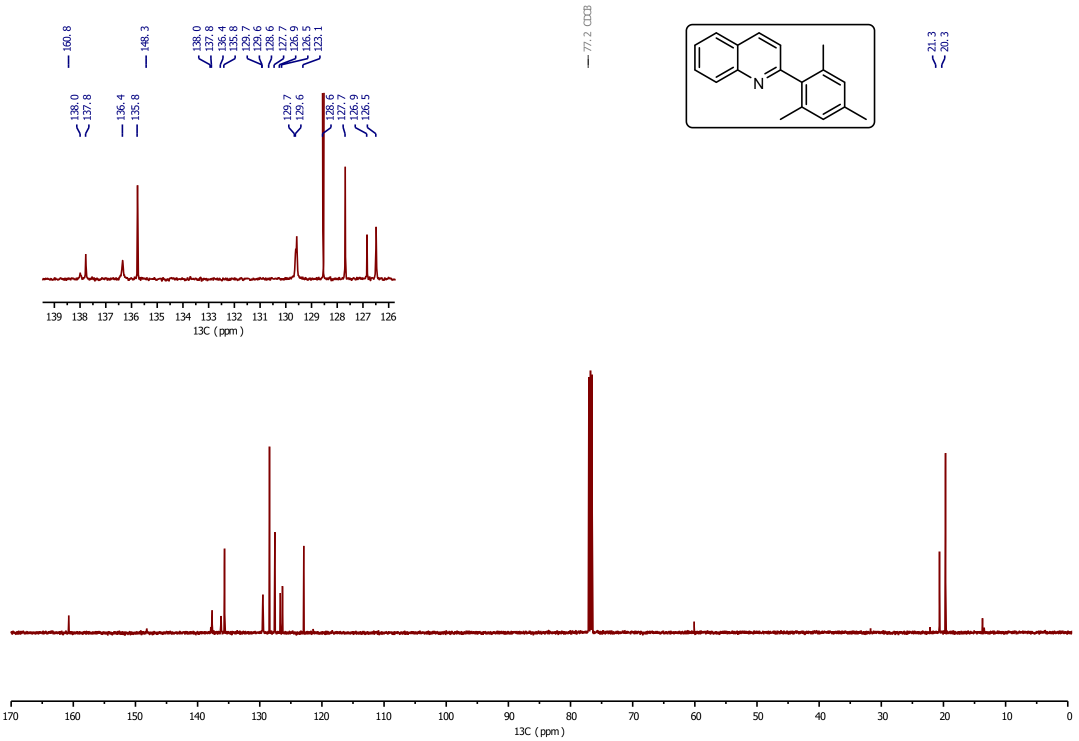

Figure S34: **^{13}C {^{1}H} NMR** (126 MHz, $CDCl_3$, 298K) of **S5**.

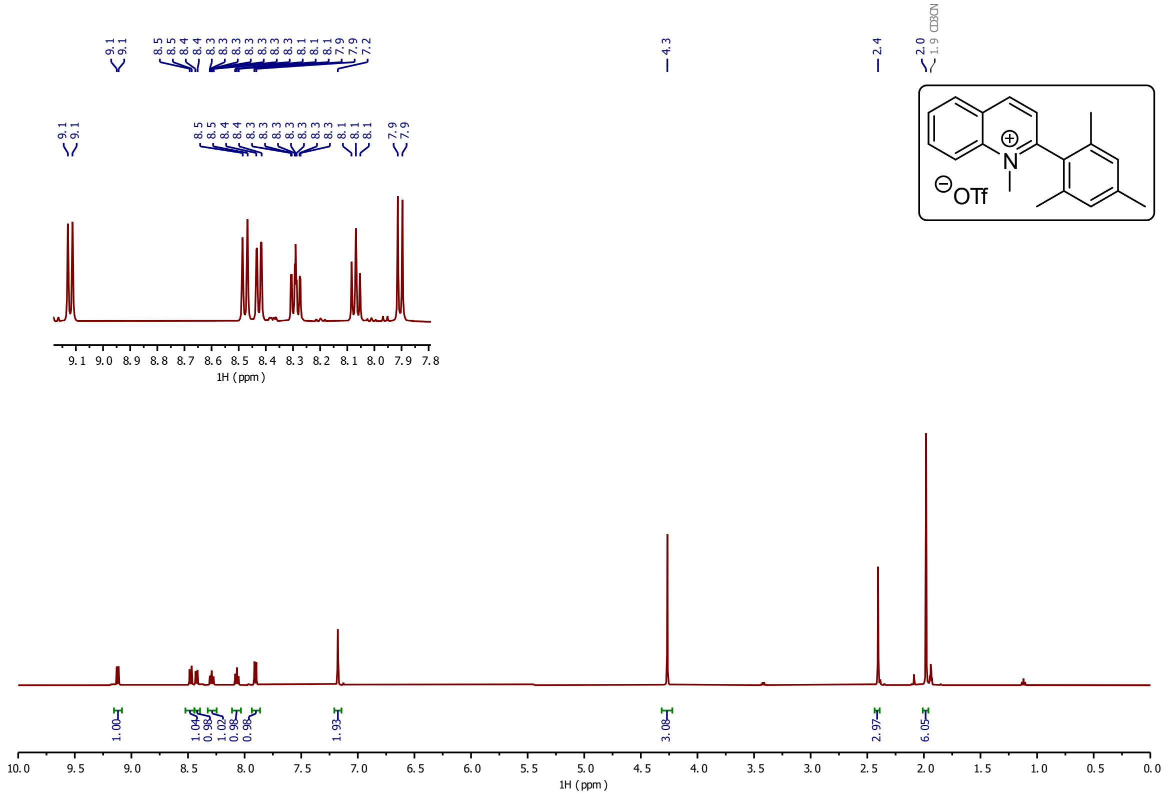

Figure S35: **^{1}H NMR** (500 MHz, CD_3CN, 298K) of **1d**.

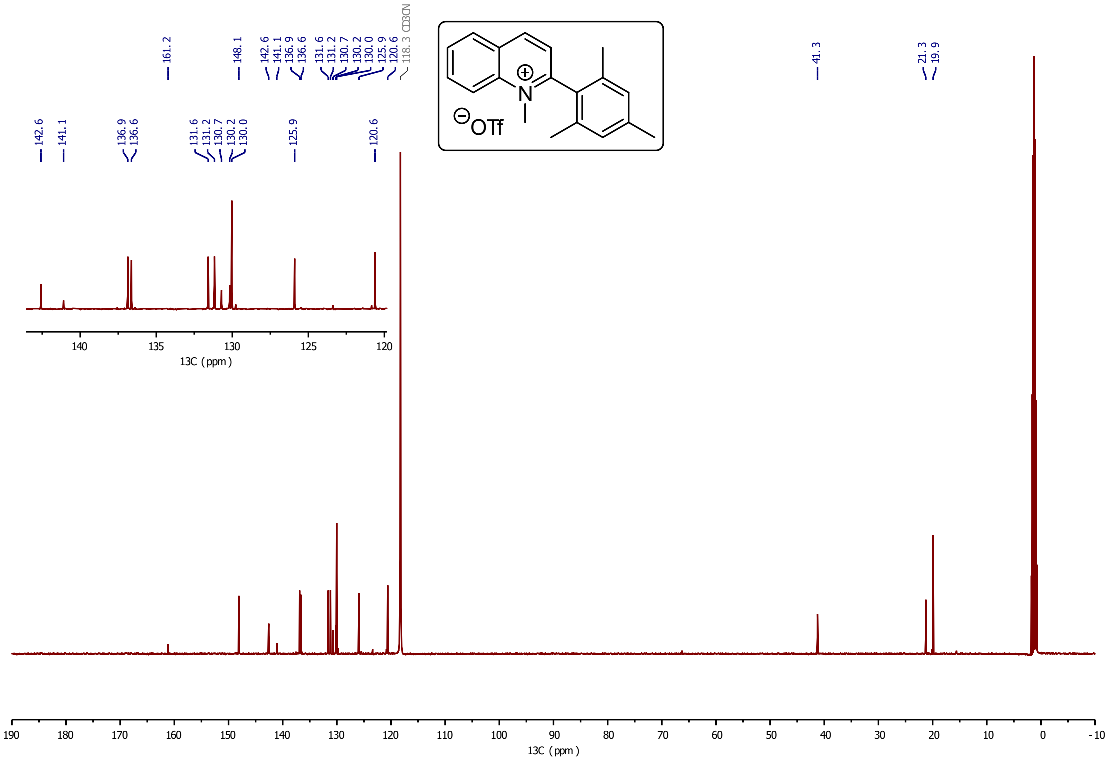

Figure S36: **^{13}C {^{1}H} NMR** (126 MHz, CD_3CN, 298K) of **1d**.

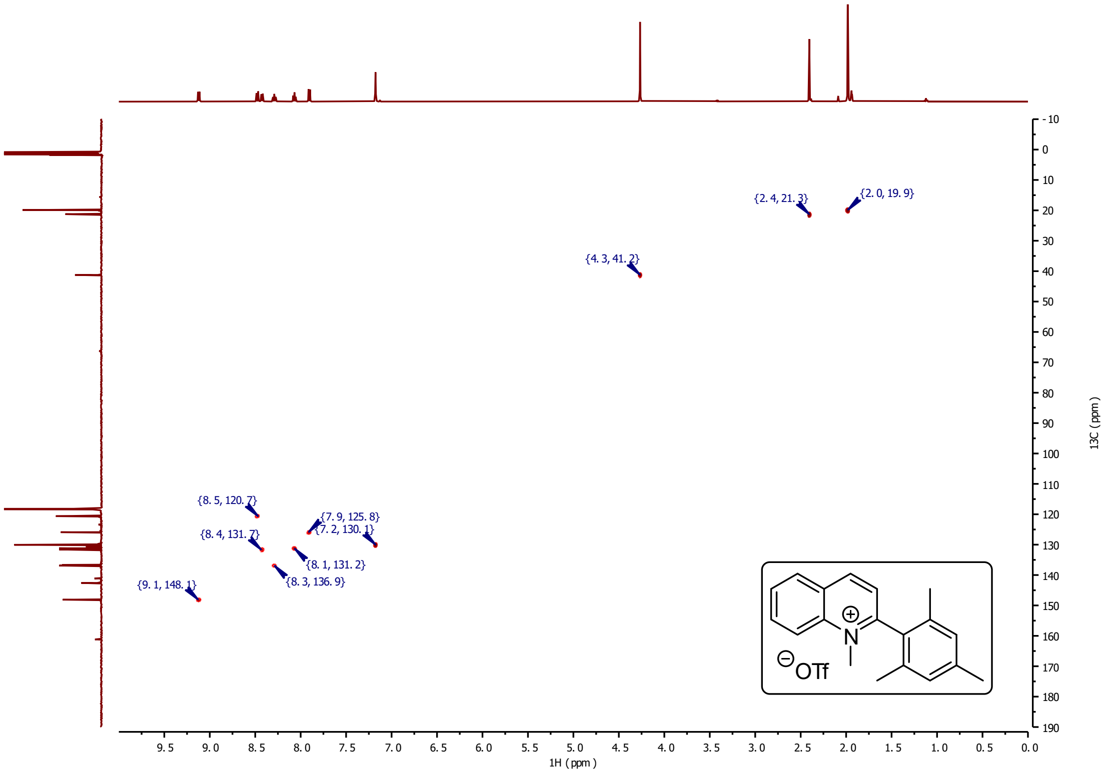

Figure S37: **$^{1}H/^{13}C$ HSQC** (500/126 MHz, CD_3CN, 298K) of **1d**.

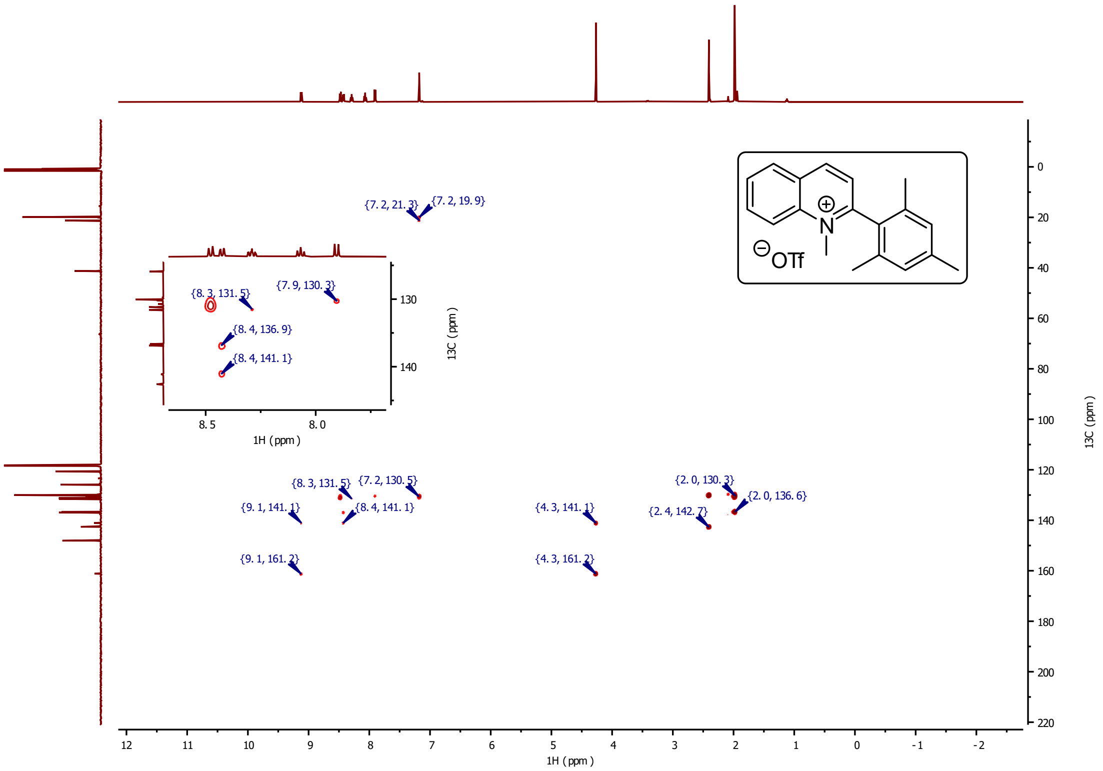

Figure S38: **$^{1}H/^{13}C$ HMBC** (500/126 MHz, CD_3CN, 298K) of **1d**.

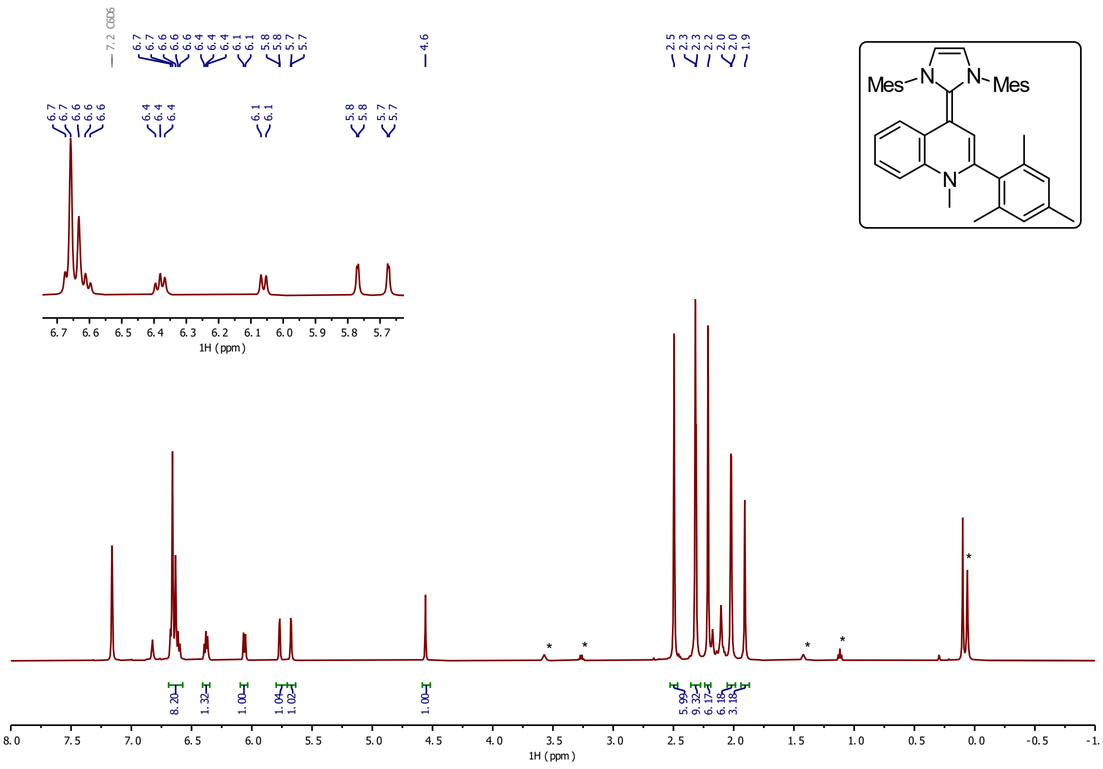

Figure S39: ^{1}H NMR (500 MHz, C_6D_6, 298K) of **2d**, residual thf, Et_2O, KHMDS marked with *.

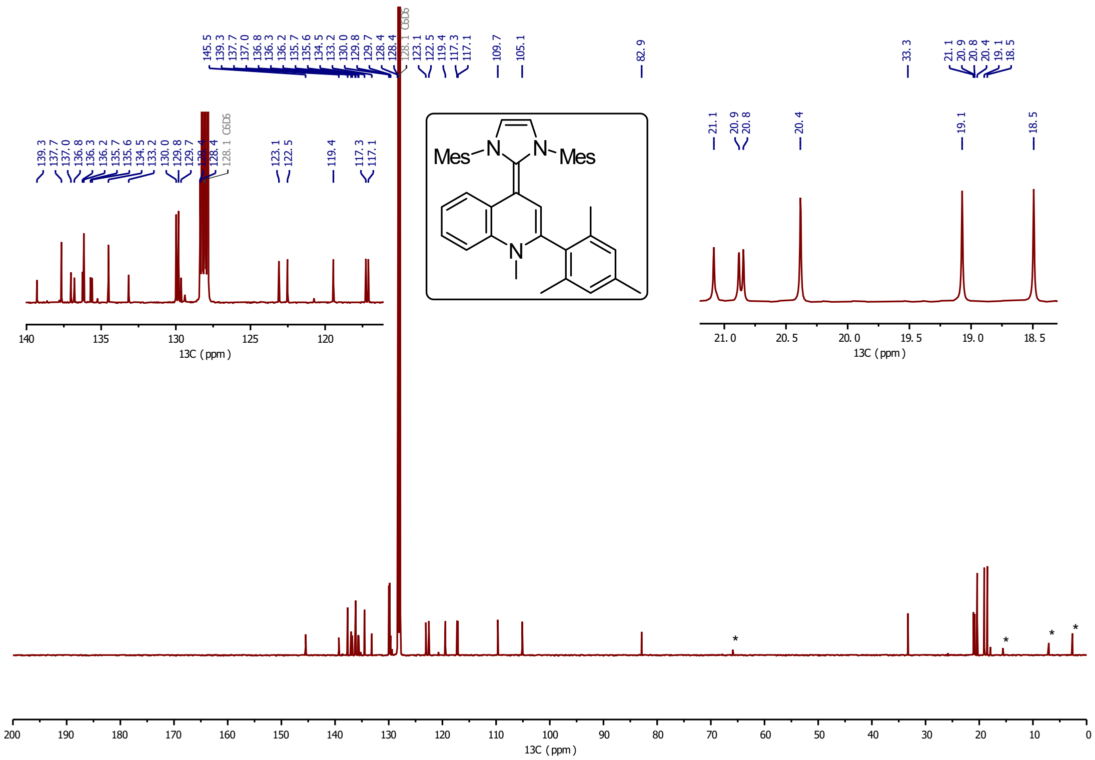

Figure S40: ^{13}C {^{1}H} NMR (126 MHz, C_6D_6, 298K) of **2d**, residual Et_2O, KHMDS marked with *.

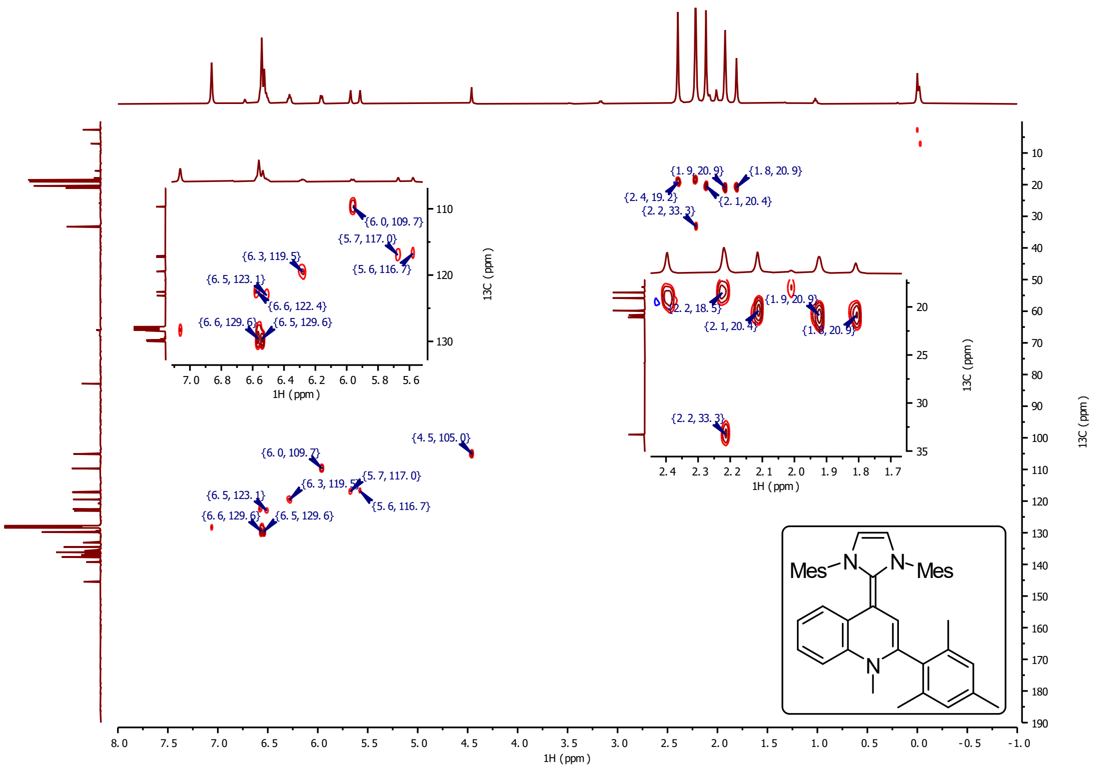

Figure S41: $^{1}H/^{13}C$ **HSQC** (500/126 MHz, C_6D_6, 298K) of **2d**.

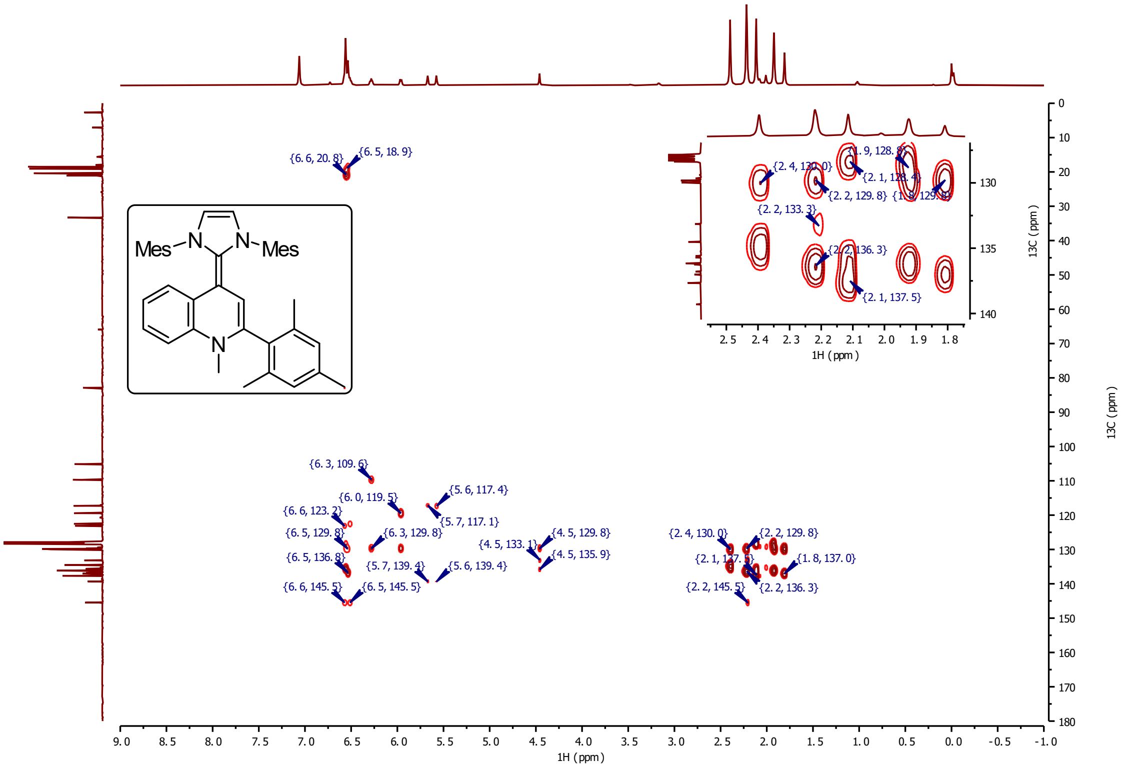

Figure S42: $^{1}H/^{13}C$ **HMBC** (500/126 MHz, C_6D_6, 298K) of **2d**.

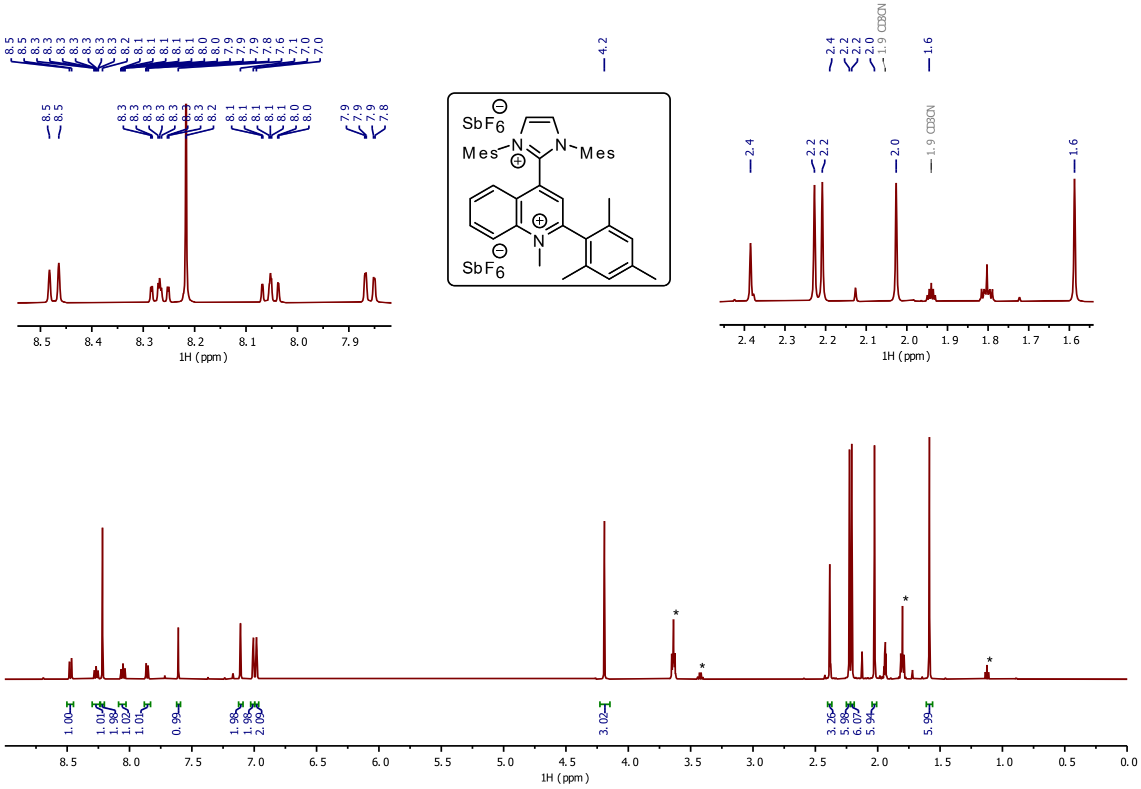

Figure S43: **^{1}H NMR** (500 MHz, CD_3CN, 298K) of **4d**, residual thf, Et_2O marked with *.

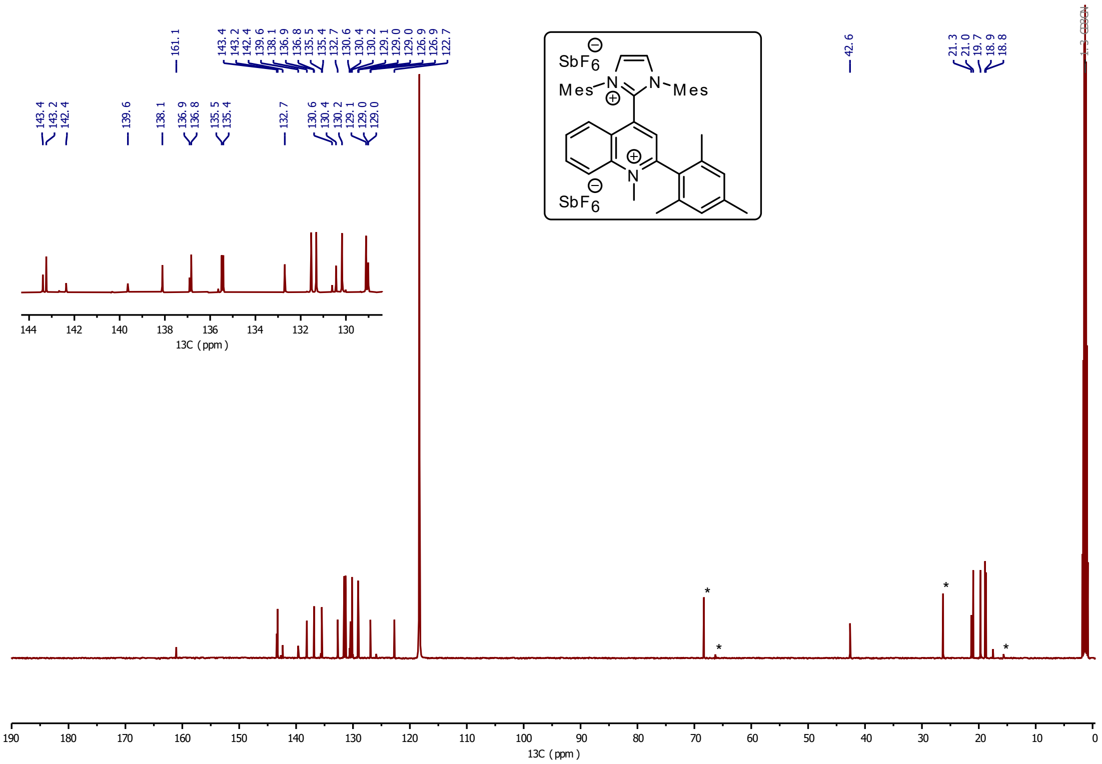

Figure S44: **^{13}C {^{1}H} NMR** (126 MHz, CD_3CN, 298K) of **4d**, residual thf, Et_2O marked with *.

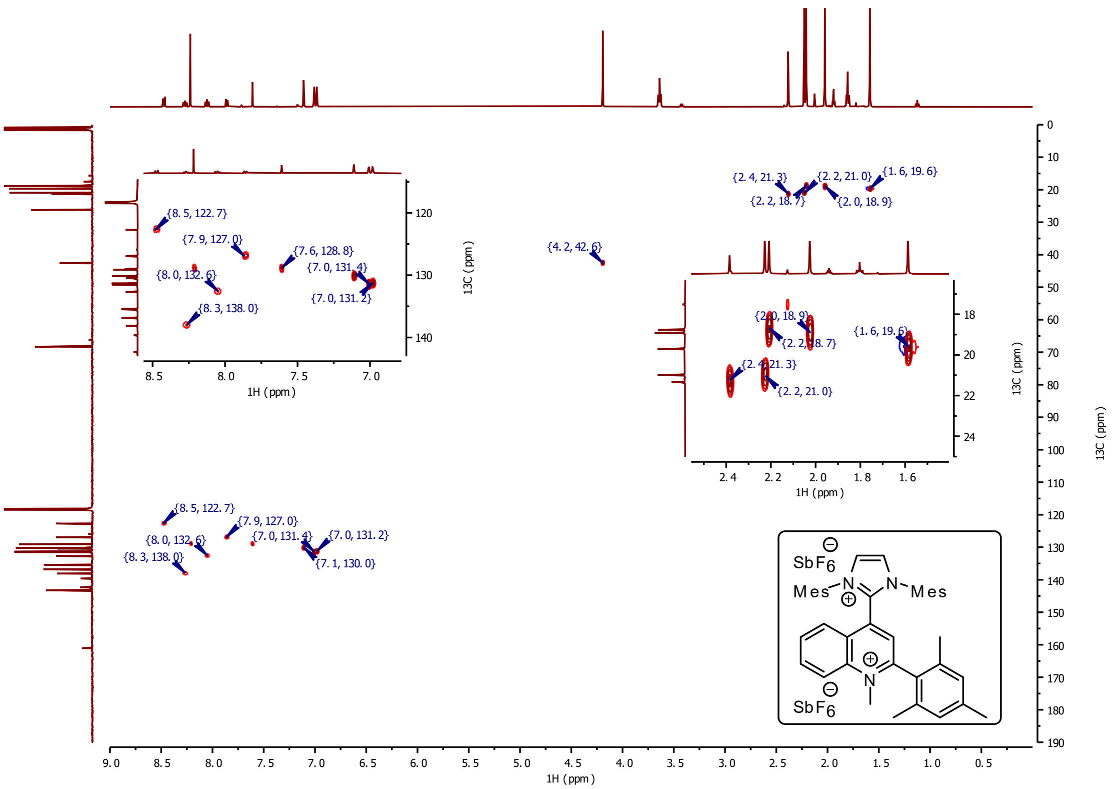

Figure S45: ^{1}H/^{13}C HSQC (500/126 MHz, CD_3CN, 298K) of **4d**.

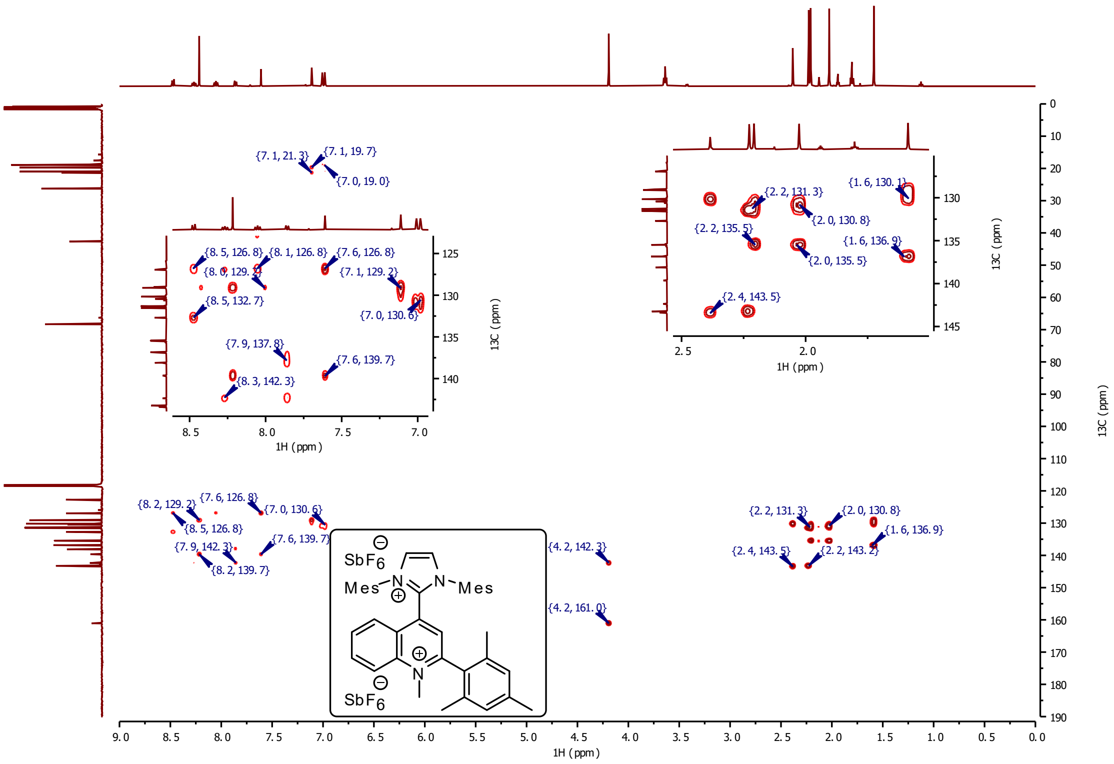

Figure S46: ^{1}H/^{13}C HMBC (500/126 MHz, CD_3CN, 298K) of **4d**.

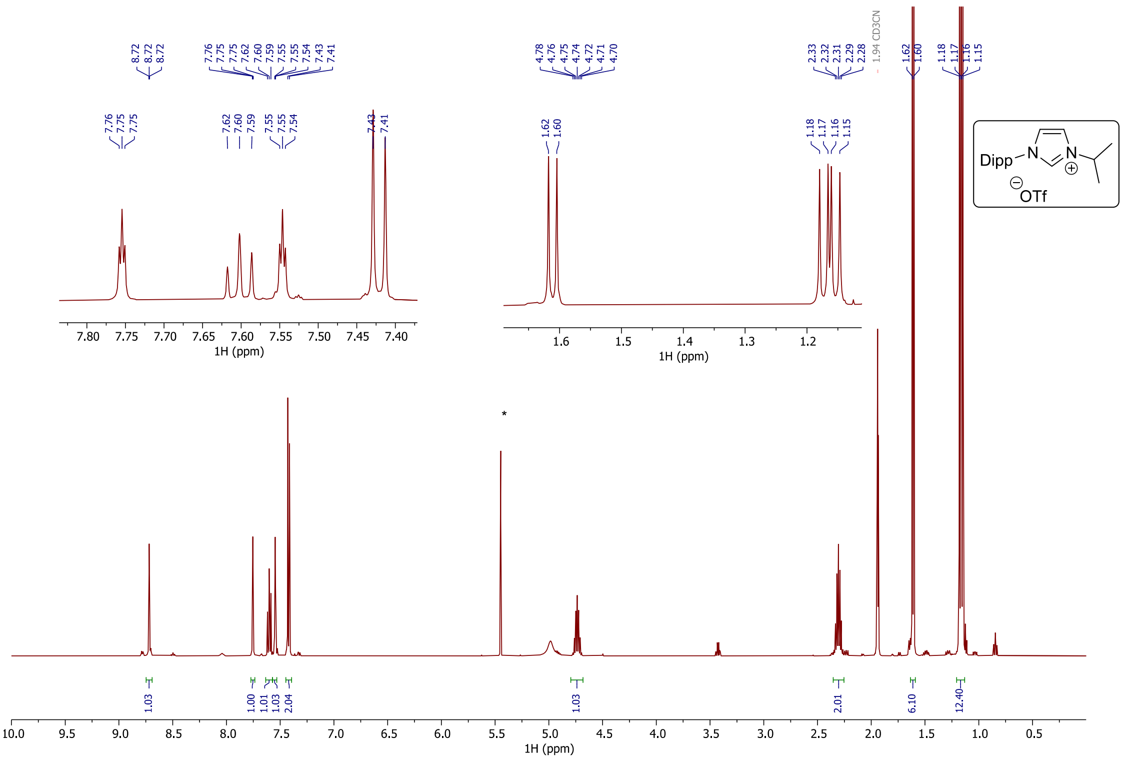

Figure S47: ^{1}H NMR (500 MHz, CD_3CN, 298K) of **S6**, residual CH_2Cl_2 is marked with a *.

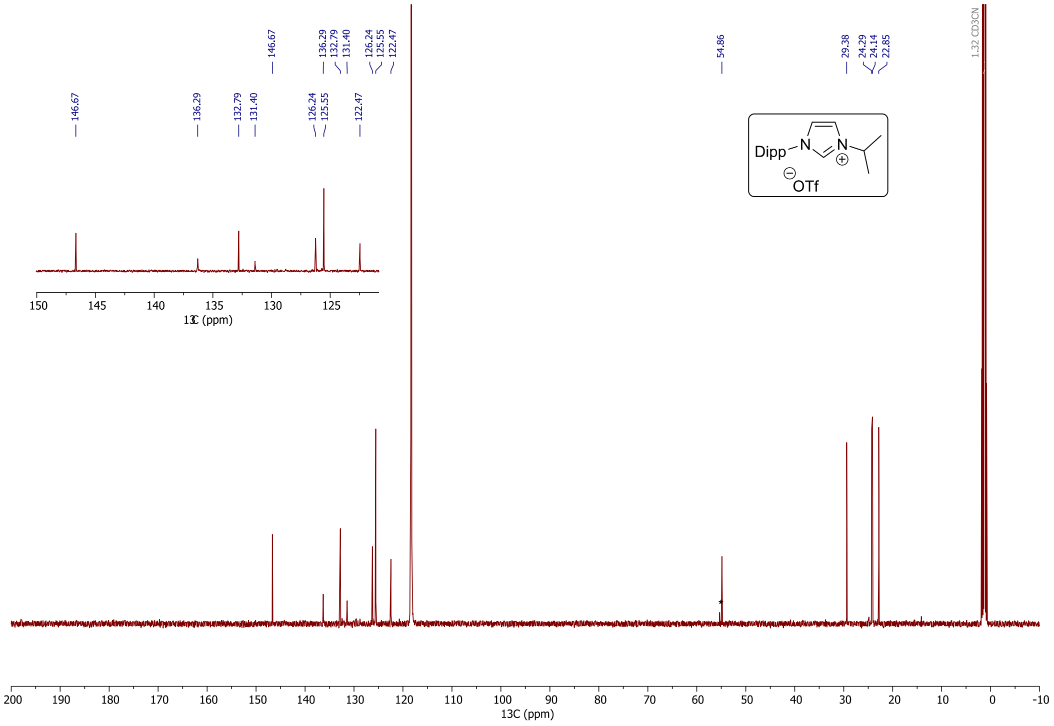

Figure S48: ^{13}C {^{1}H} NMR (126 MHz, CD_3CN, 298K) of **S6**, residual CH_2Cl_2 is marked with a *.

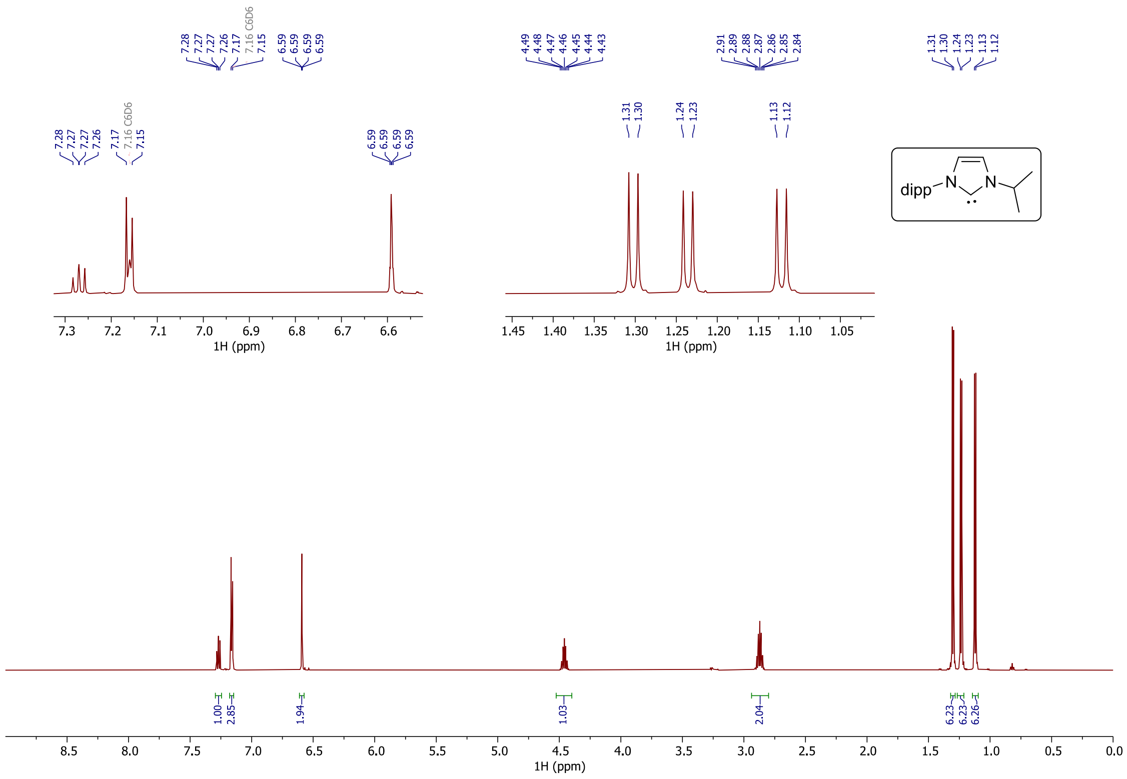

Figure S49: ^{1}H NMR (600 MHz, C_6D_6, 298K) of **Idipp^{iPr}**.

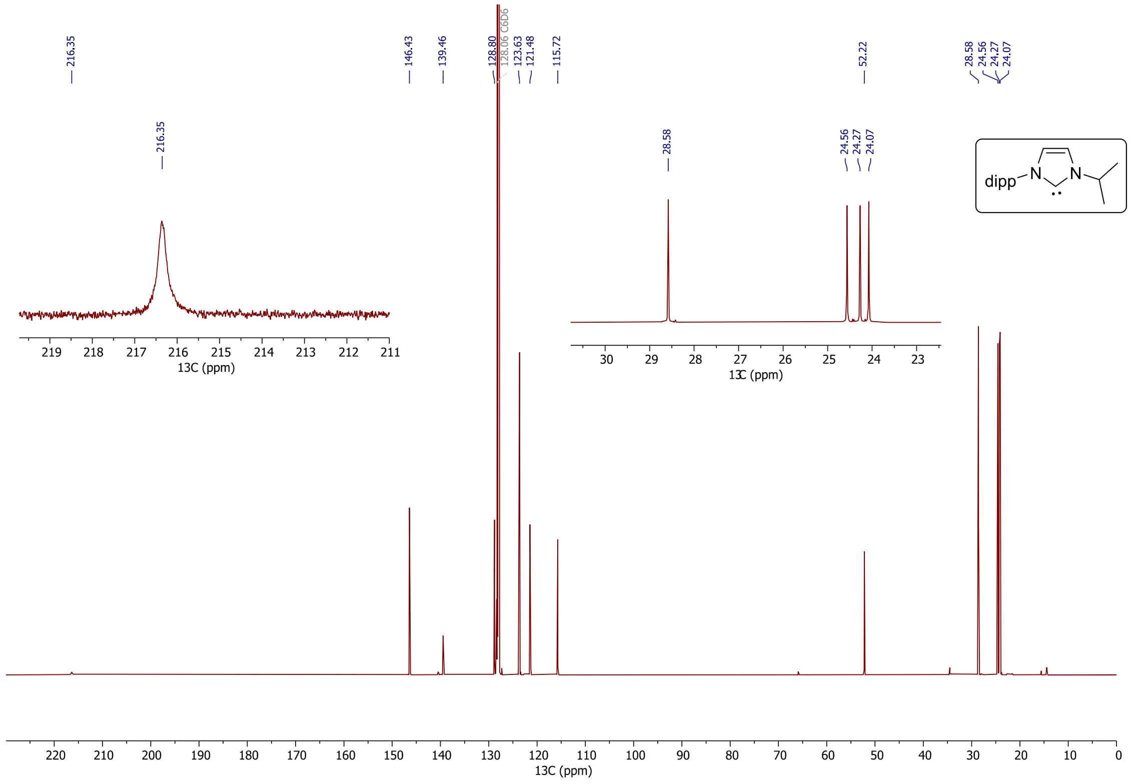

Figure S50: ^{13}C {^{1}H} NMR (151 MHz, C_6D_6, 298K) of **Idipp^{iPr}**.

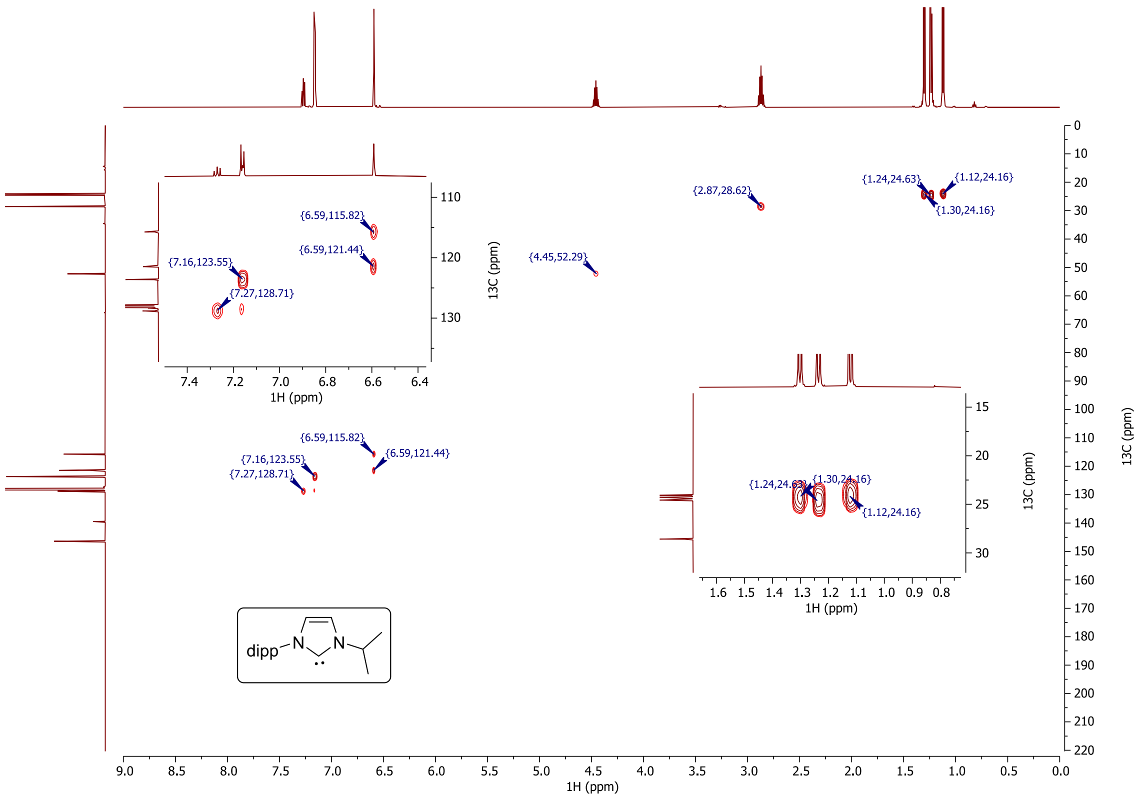

Figure S51: $^{1}H/^{13}C$ **HSQC** (600/151 MHz, C_6D_6, 298K) of **Idipp^{iPr}**.

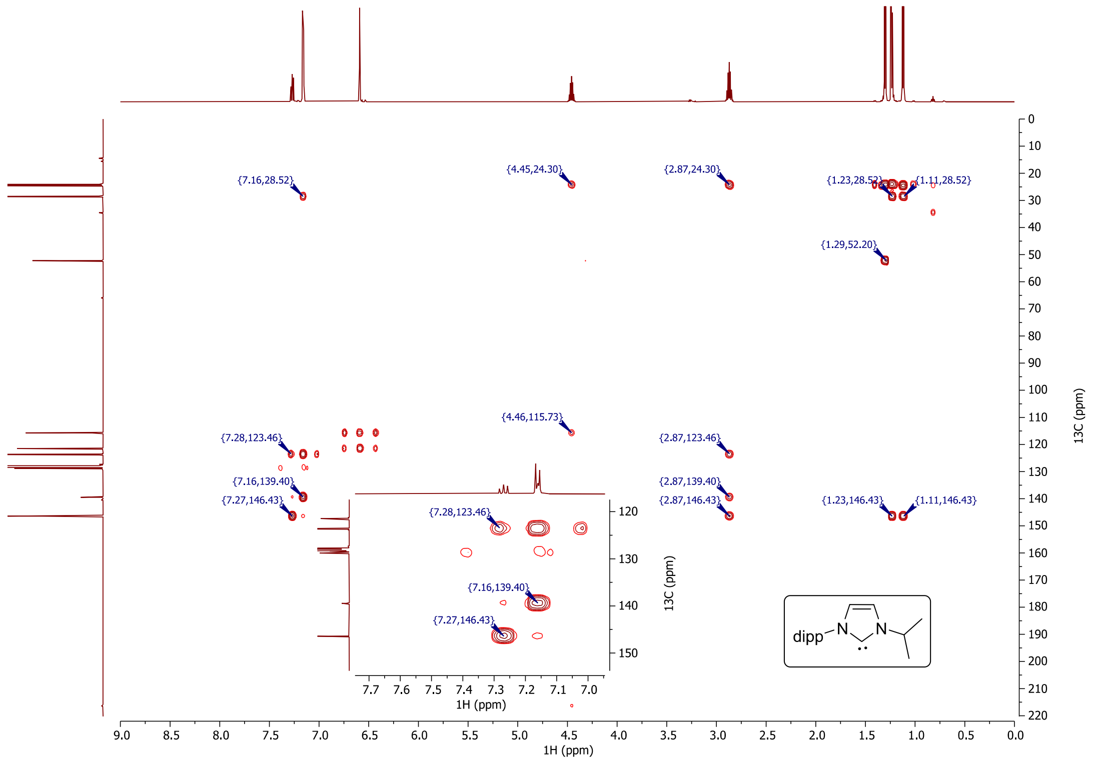

Figure S52: $^{1}H/^{13}C$ **HMBC** (600/151 MHz, C_6D_6, 298K) of **Idipp^{iPr}**.

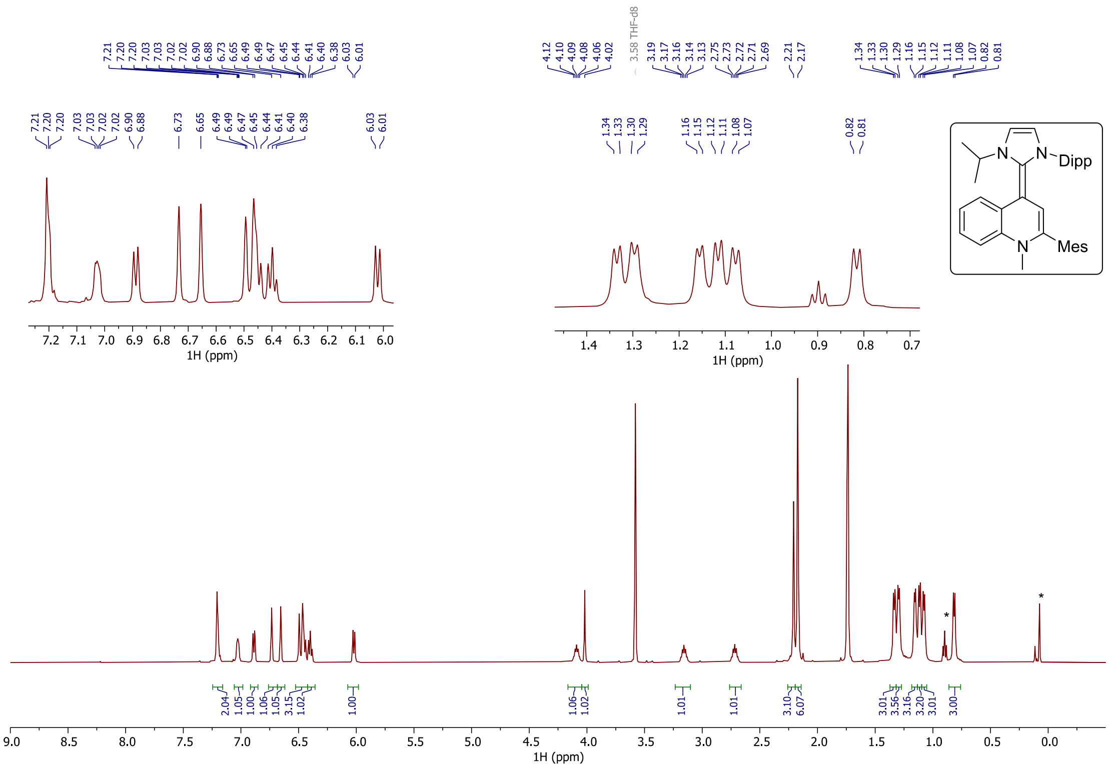

Figure S53: ^{1}**H NMR** (500 MHz, thf-d_8, 193K) of **2e**, residual pentane, HMDSO is marked with a *.

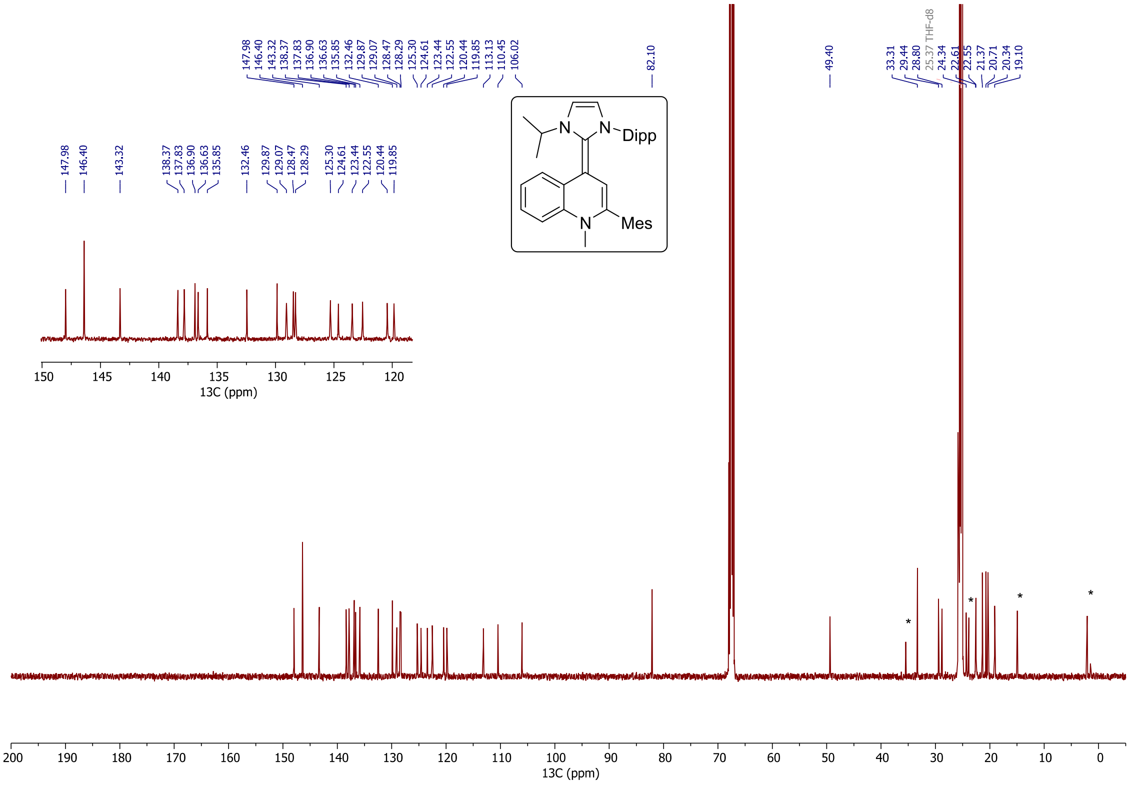

Figure S54: ^{13}**C {**^{1}**H} NMR** (126 MHz, thf-d_8, 193K) of **2e**, residual HMDSO is marked with a *.

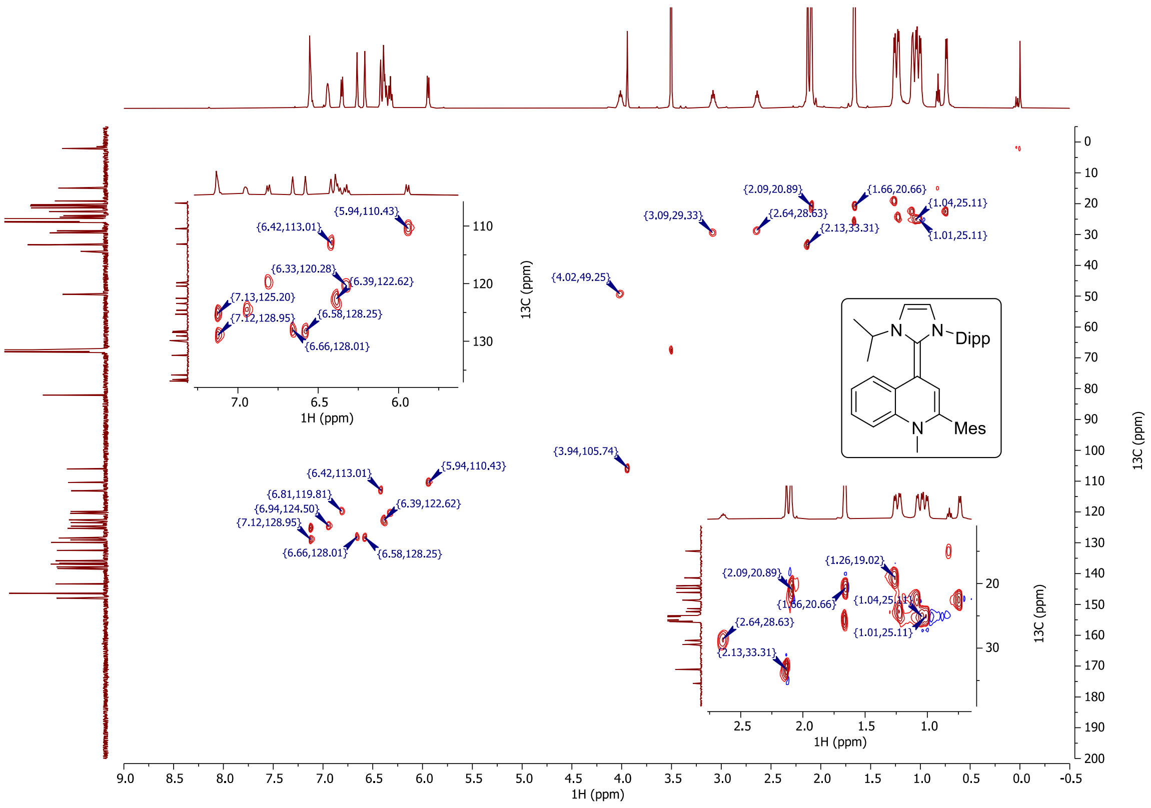

Figure S55: ^{1}H/^{13}C HSQC (500/126 MHz, thf-d$_8$, 193K) of **2e**.

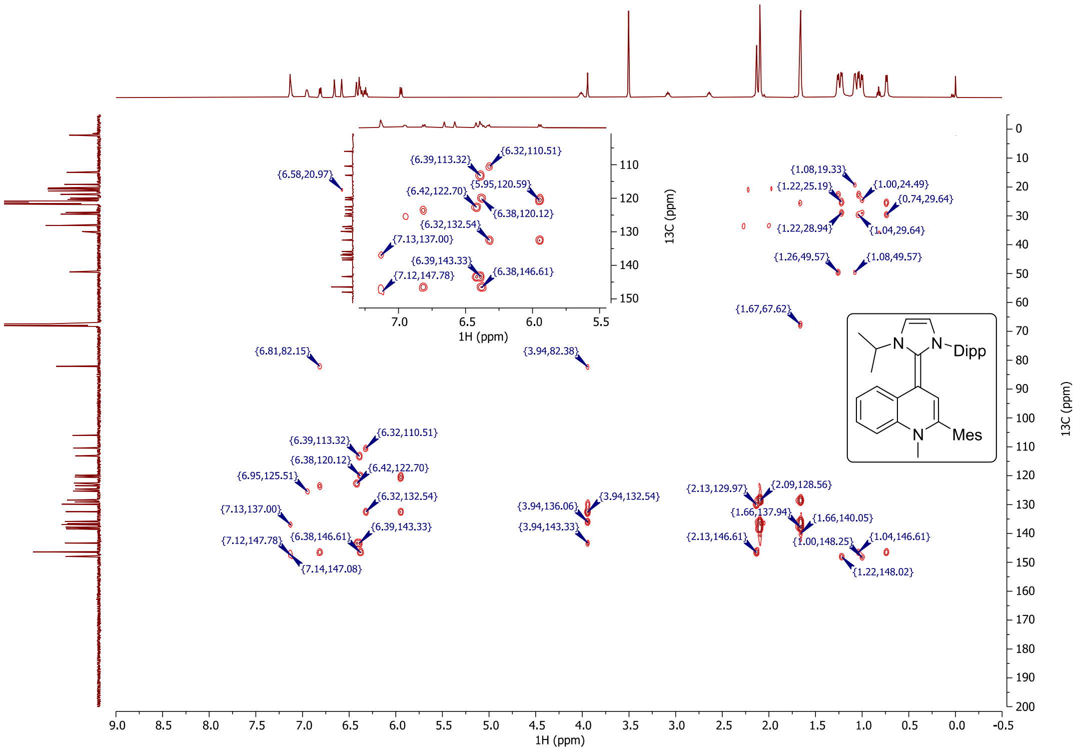

Figure S56: ^{1}H/^{13}C HMBC (500/126 MHz, thf-d$_8$, 193K) of **2e**.

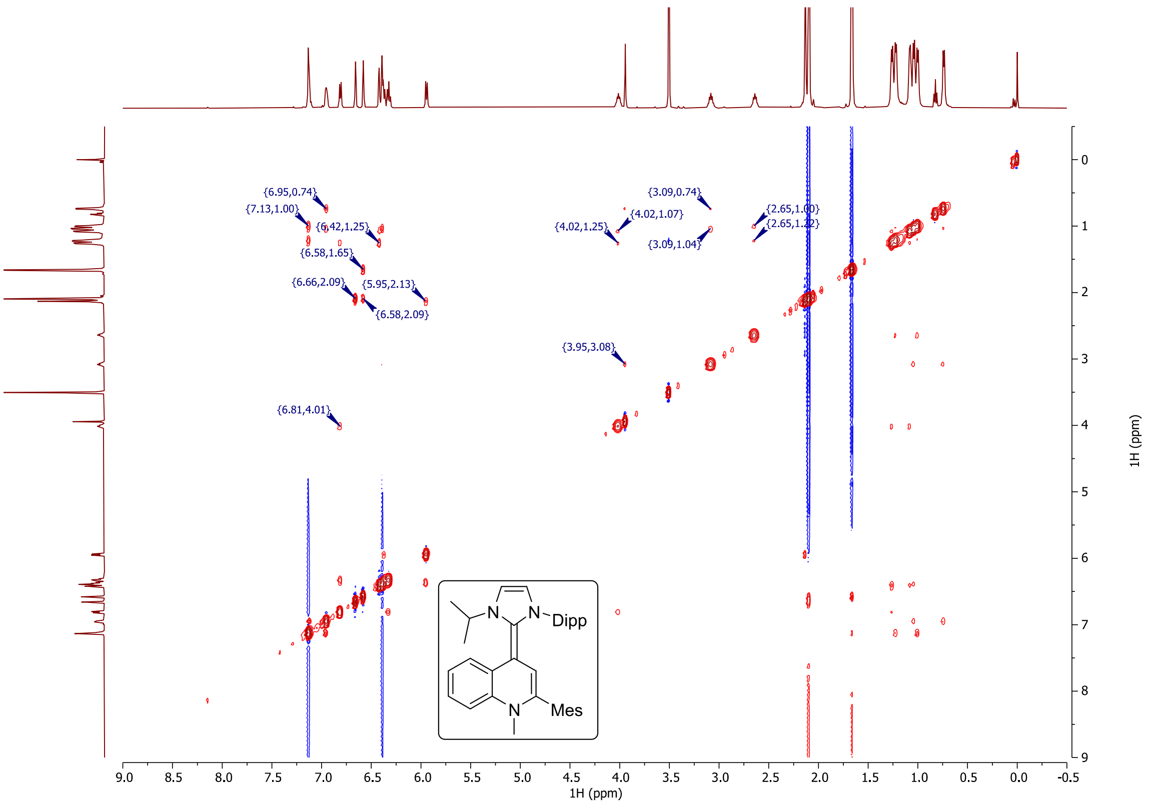

Figure S57: NOESY-NMR (500 MHz, thf-d$_8$, 193K) of **2e**.

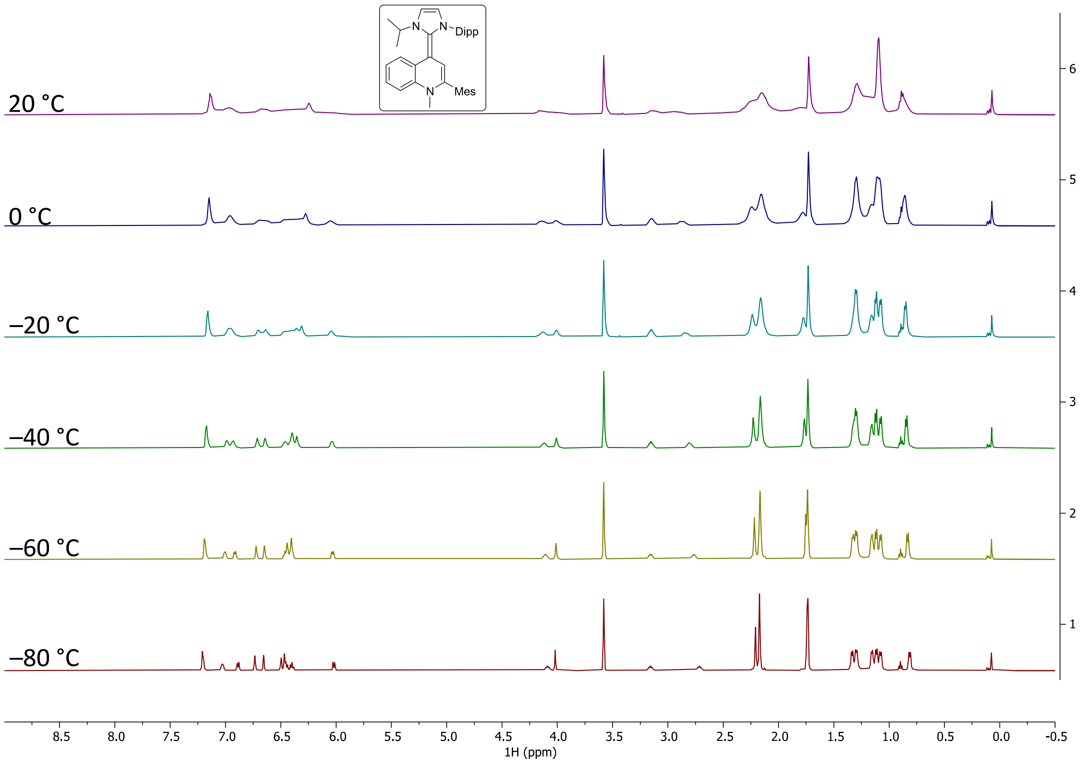

Figure S58: ^{1}H NMR (500 MHz, thf-d$_8$, temperatures as indicated) of **2e** while warming up from –80 °C to 20 °C.

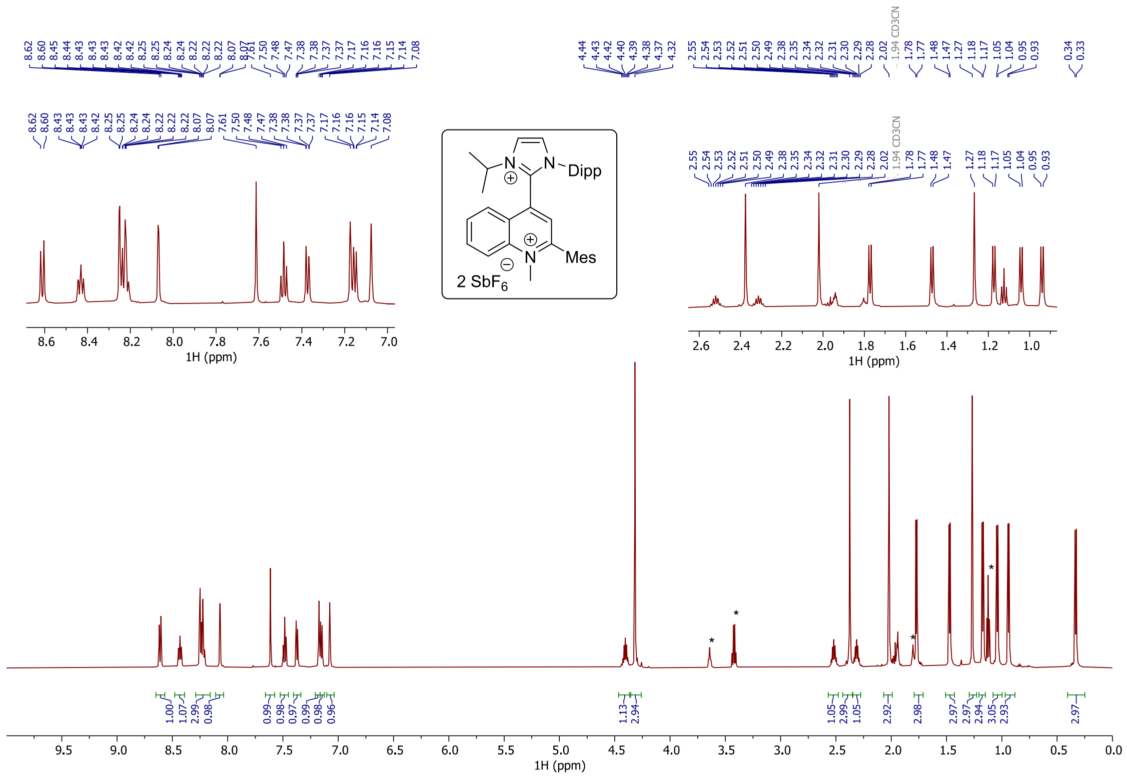

Figure S59: ^{1}H NMR (600 MHz, CD_3CN, 298K) of **4e**, residual thf, Et_2O is marked with a *.

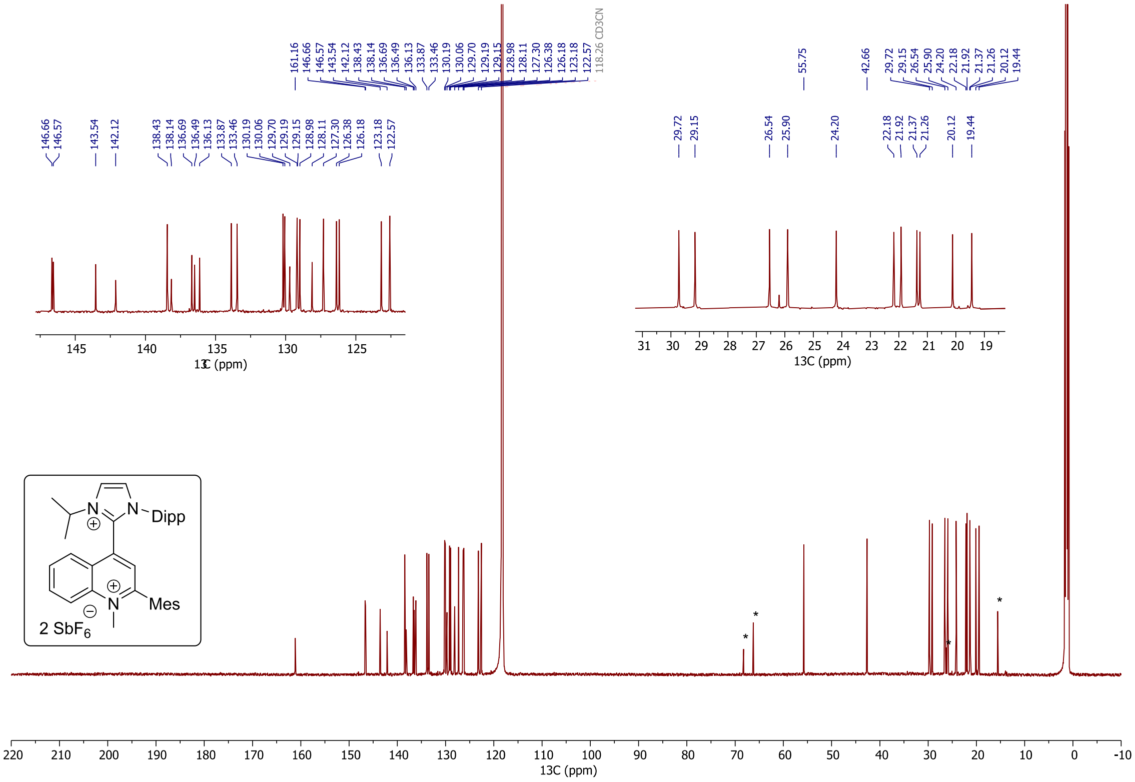

Figure S60: ^{13}C {^{1}H} NMR (151 MHz, CD_3CN, 298K) of **4e**, residual thf, Et_2O is marked with a *.

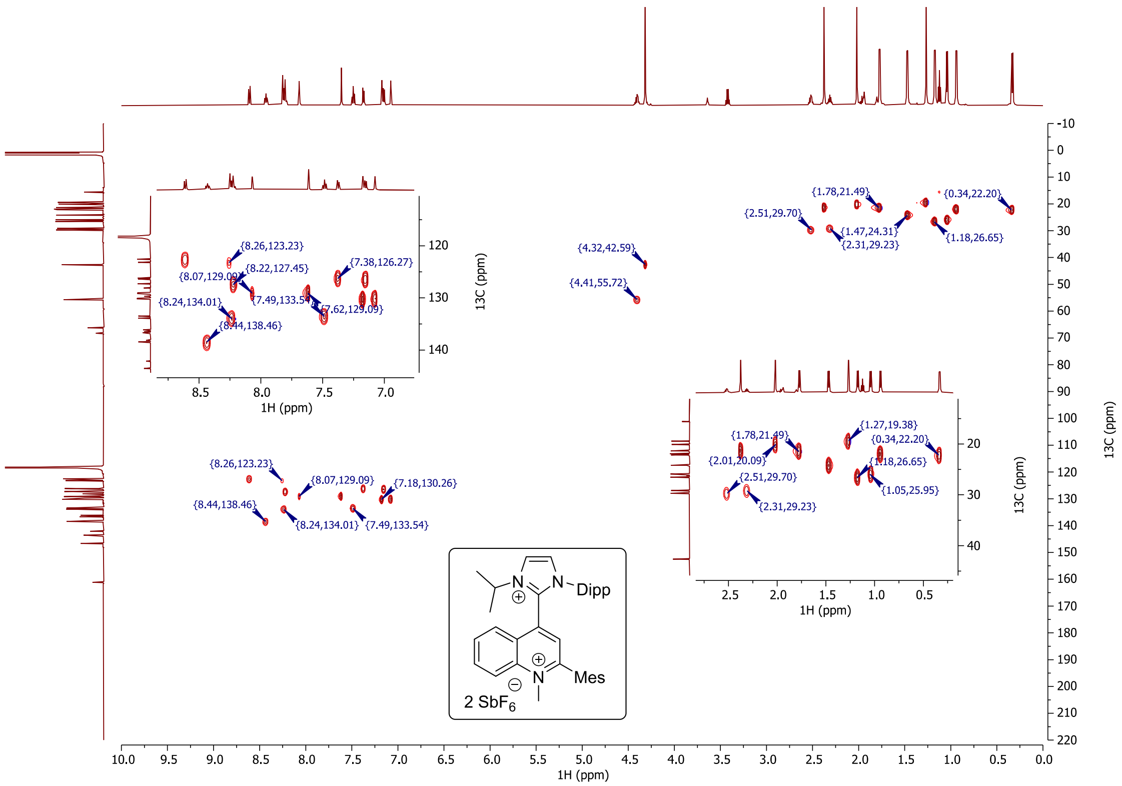

Figure S61: $^{1}H/^{13}C$ HSQC (600/151 MHz, CD_3CN, 298K) of **4e**.

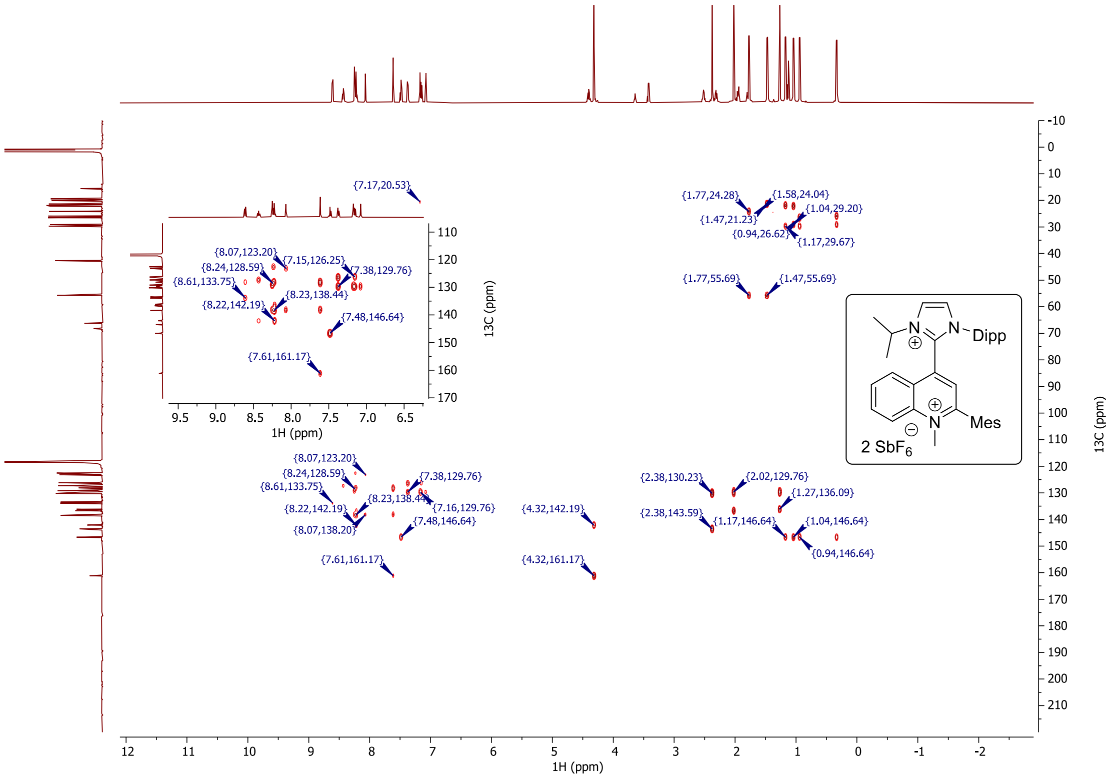

Figure S62: $^{1}H/^{13}C$ HMBC (600/151 MHz, CD_3CN, 298K) of **4e**.

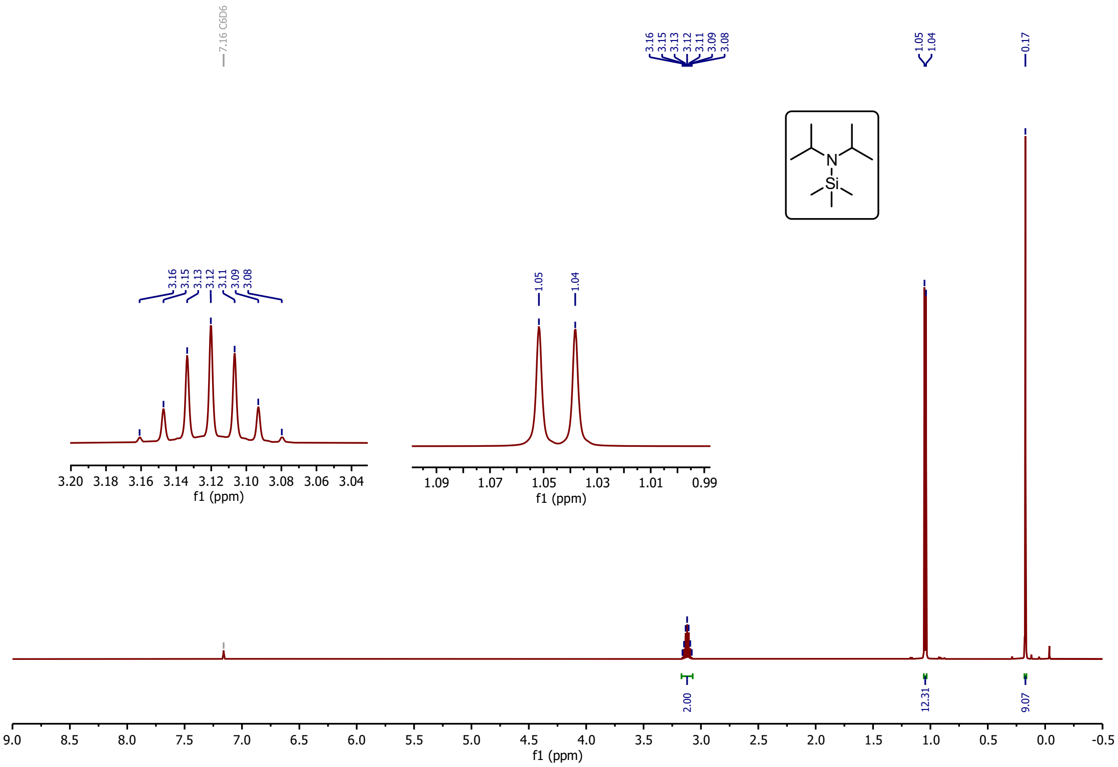

Figure S63: **^{1}H NMR** (501 MHz, C_6D_6, 298 K) of **S7**.

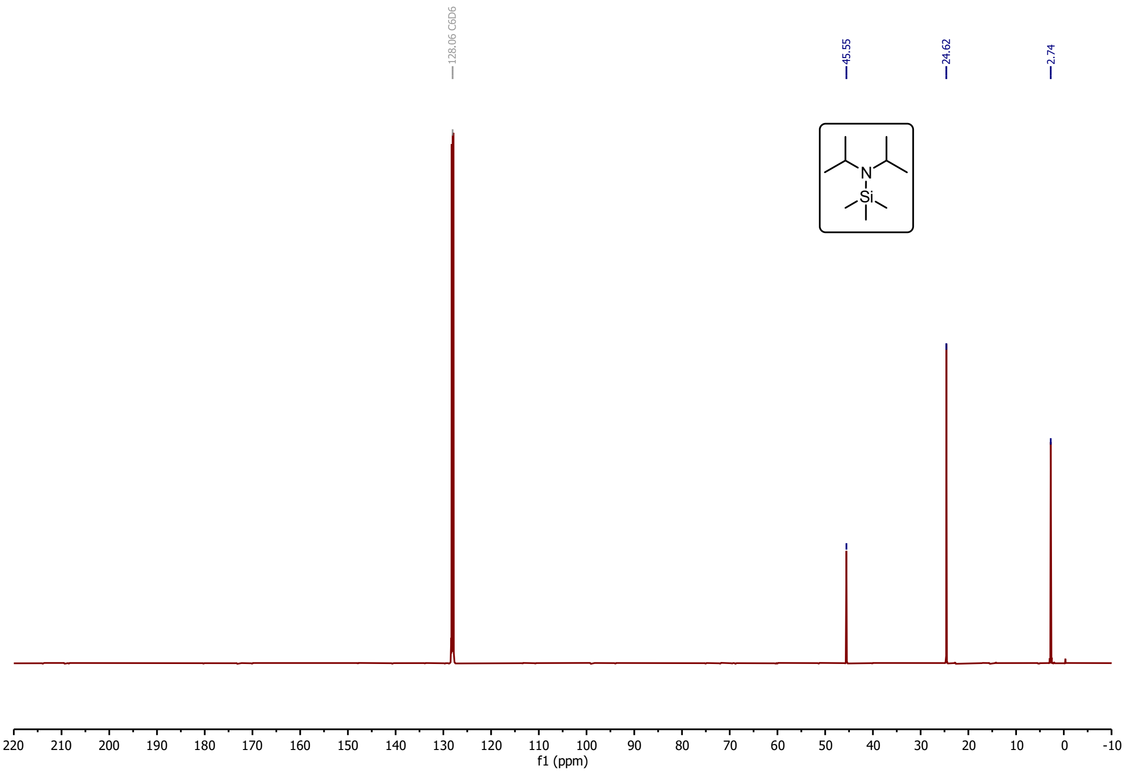

Figure S64: **^{13}C {1H} NMR** (126 MHz, C_6D_6, 298 K) of **S7.**

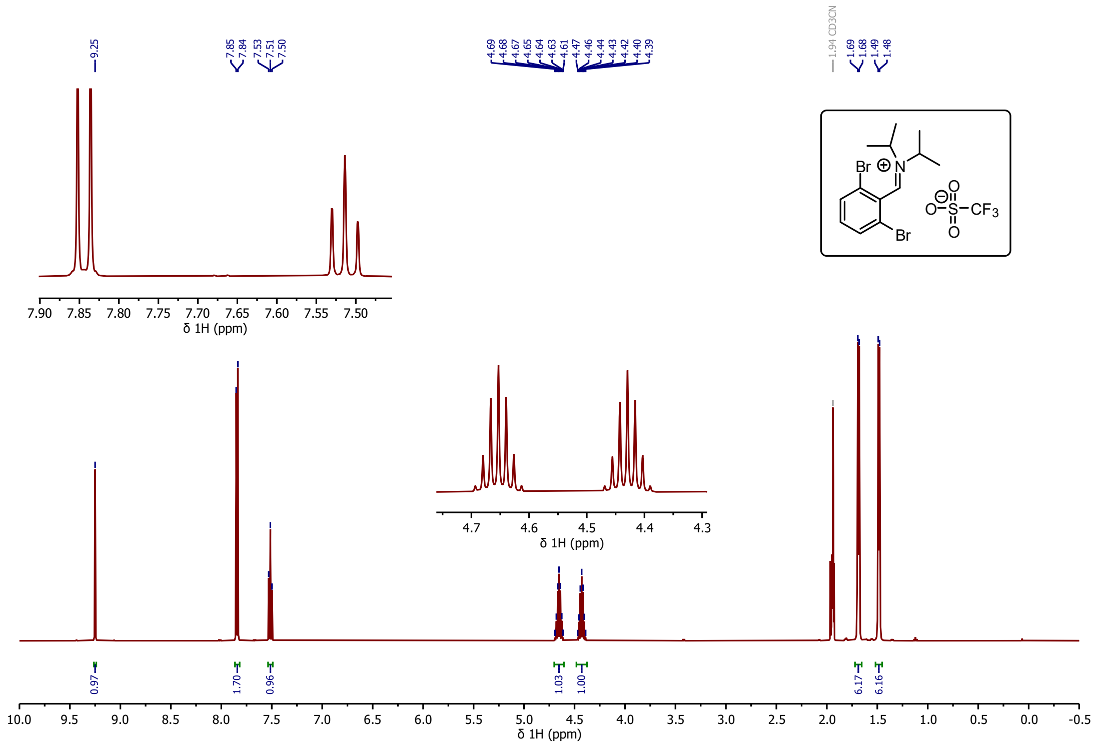

Figure S65: ¹H NMR (501 MHz, CD_3CN, 298 K) of **S8.**

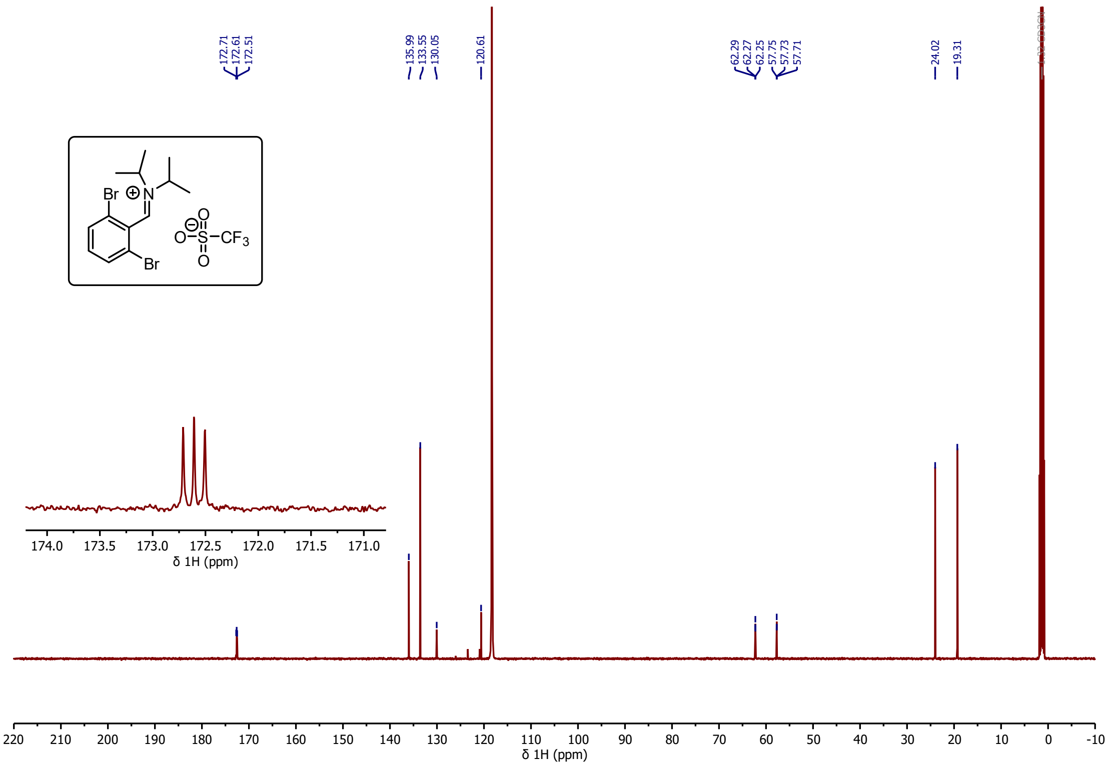

Figure S66: **^{13}C {1H} NMR** (126 MHz, CD_3CN, 298 K) of **S8**.

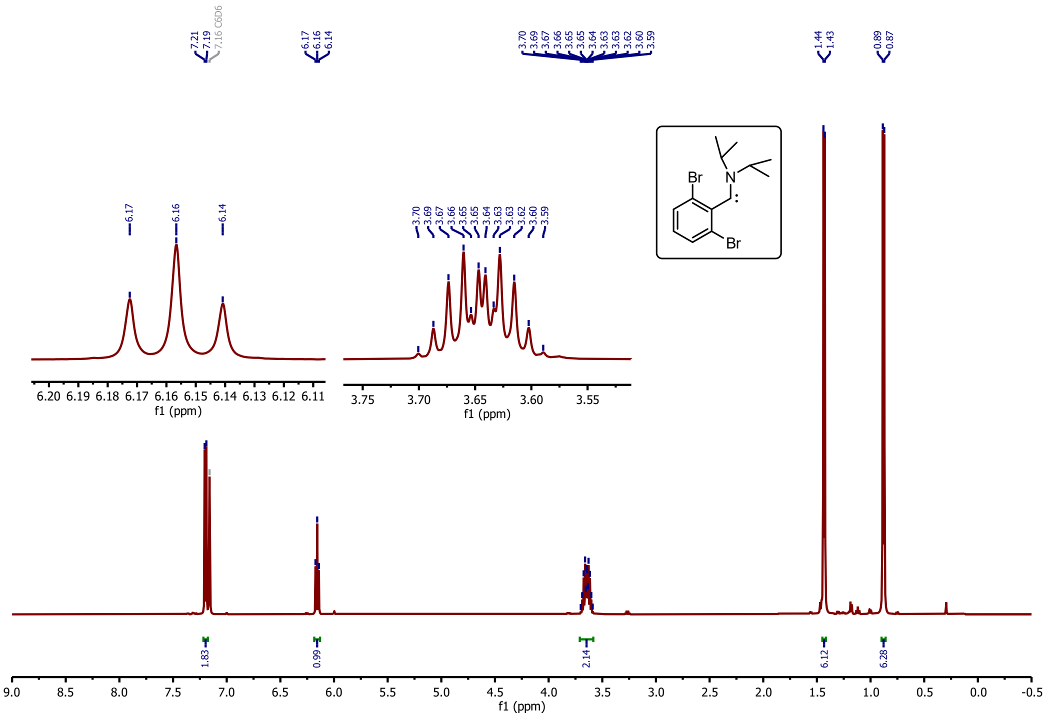

Figure S67: **^{1}H NMR** (501 MHz, C_6D_6, 298 K) of **^{Br}AAArc.**

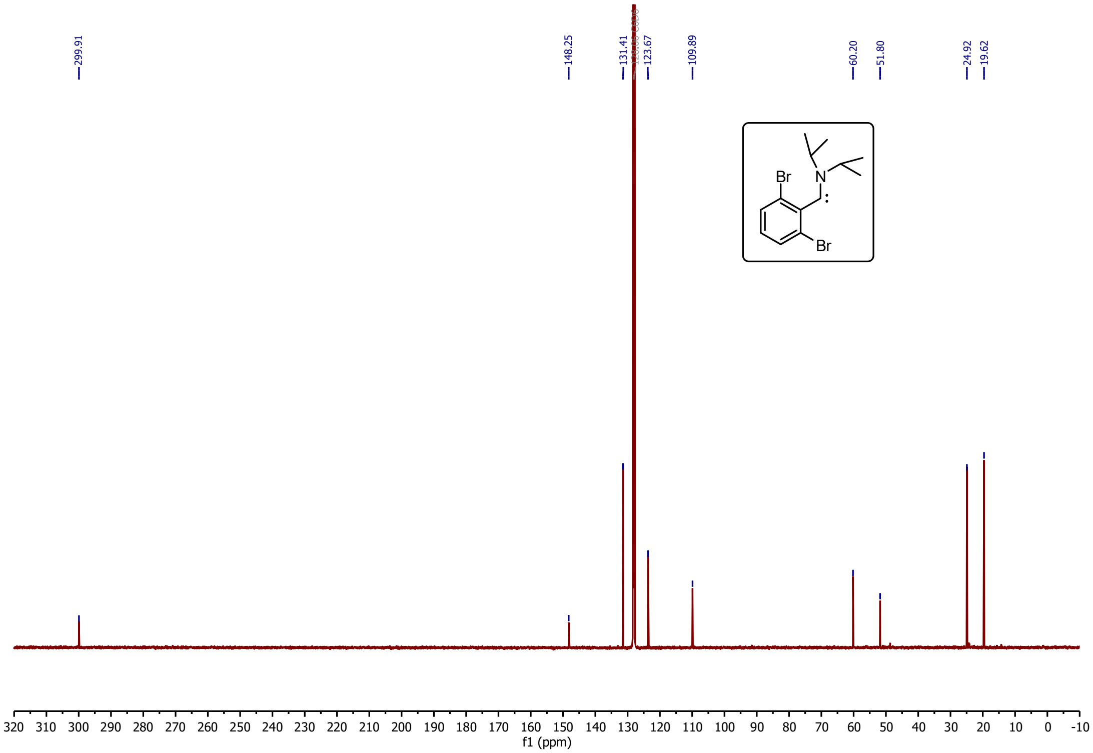

Figure S68: **^{13}C {1H} NMR** (126 MHz, C_6D_6, 298 K) of **^{Br}AAArc.**

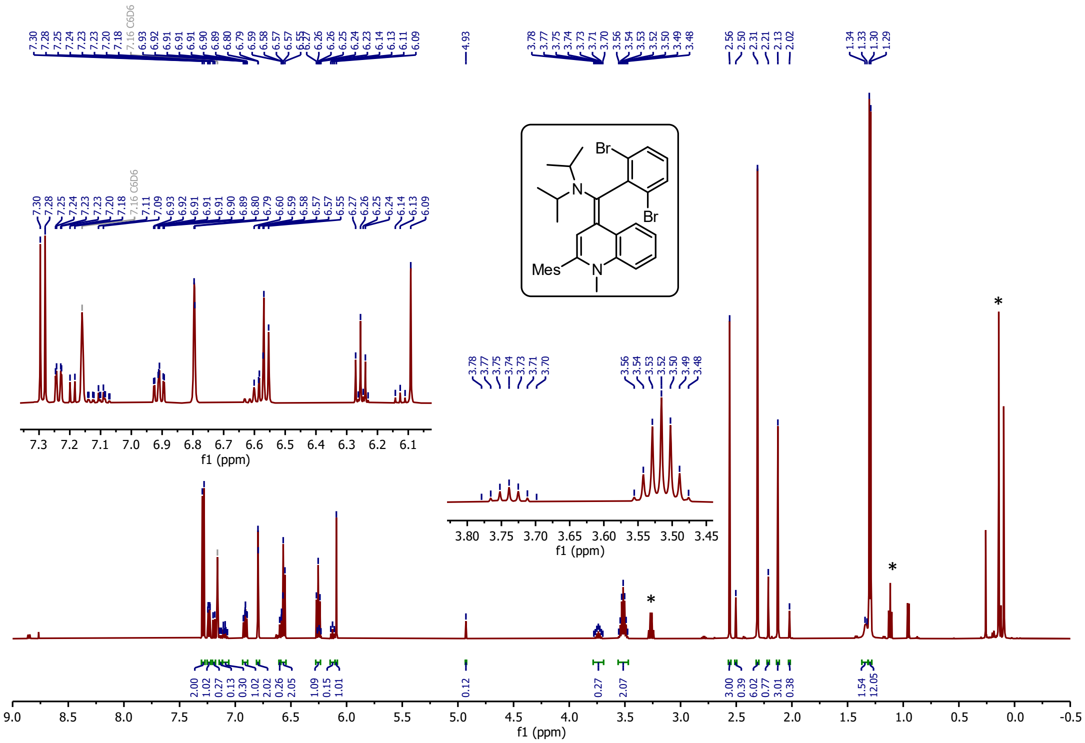

Figure S69: **^{1}H NMR** (501 MHz, C_6D_6, 298 K) of **2f**. Residual HMDSO and Et_2O is marked with *.

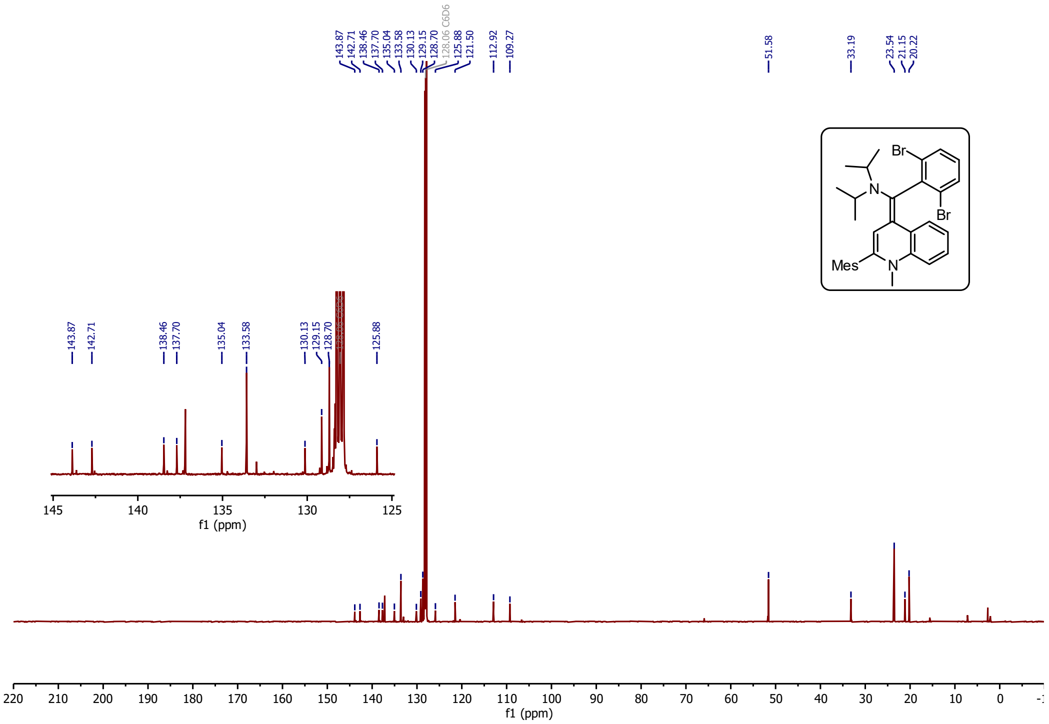

Figure S70: **^{13}C {1H} NMR** (126 MHz, C_6D_6, 298 K) of **2f**. (note: two aromatic ^{13}C-signals are according to 2D-NMR overlayed by the signal of the solvent).

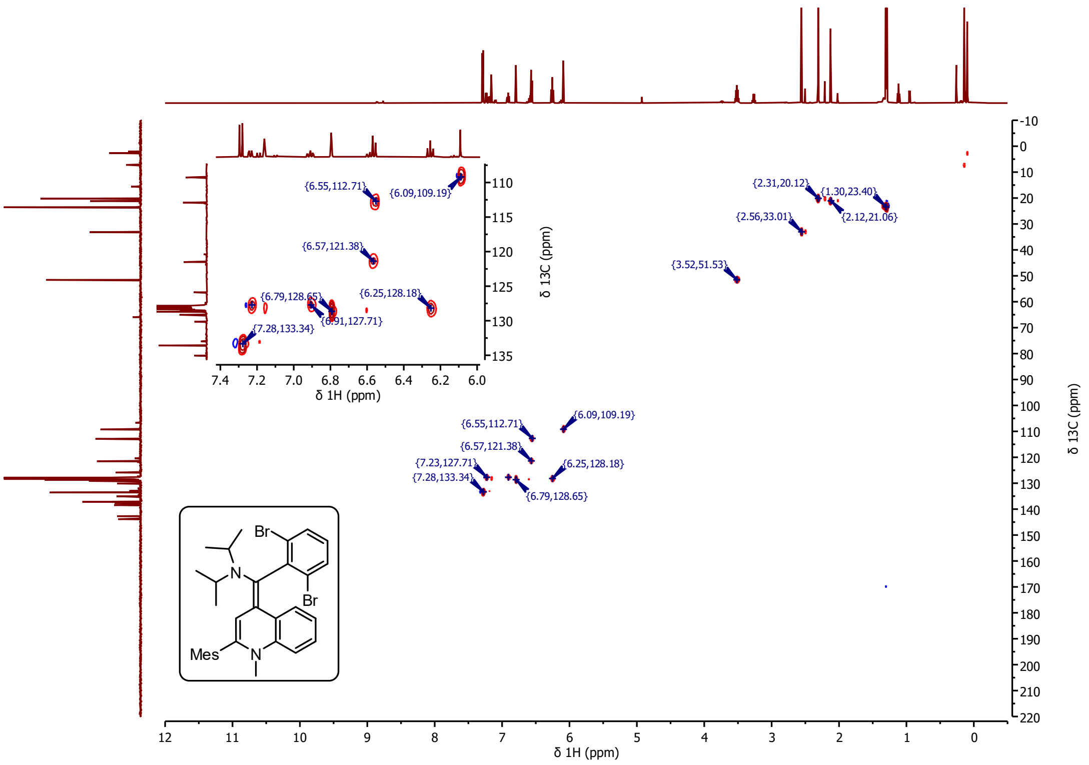

Figure S71: **$^{1}H/^{13}C$ HSQC** (501 MHz/126 MHz, C_6D_6, 298 K) of **2f**.

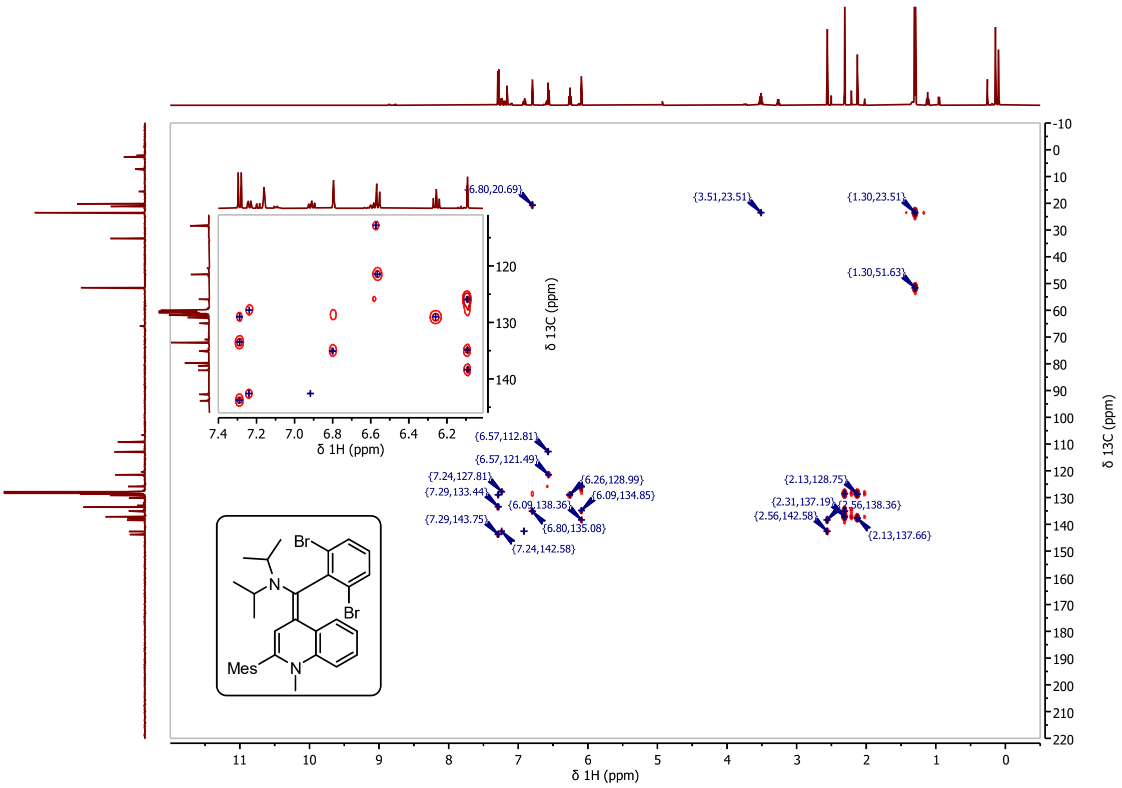

Figure S72: **$^{1}H/^{13}C$ HMBC** (501 MHz/126 MHz, C_6D_6, 298 K) of **2f**.

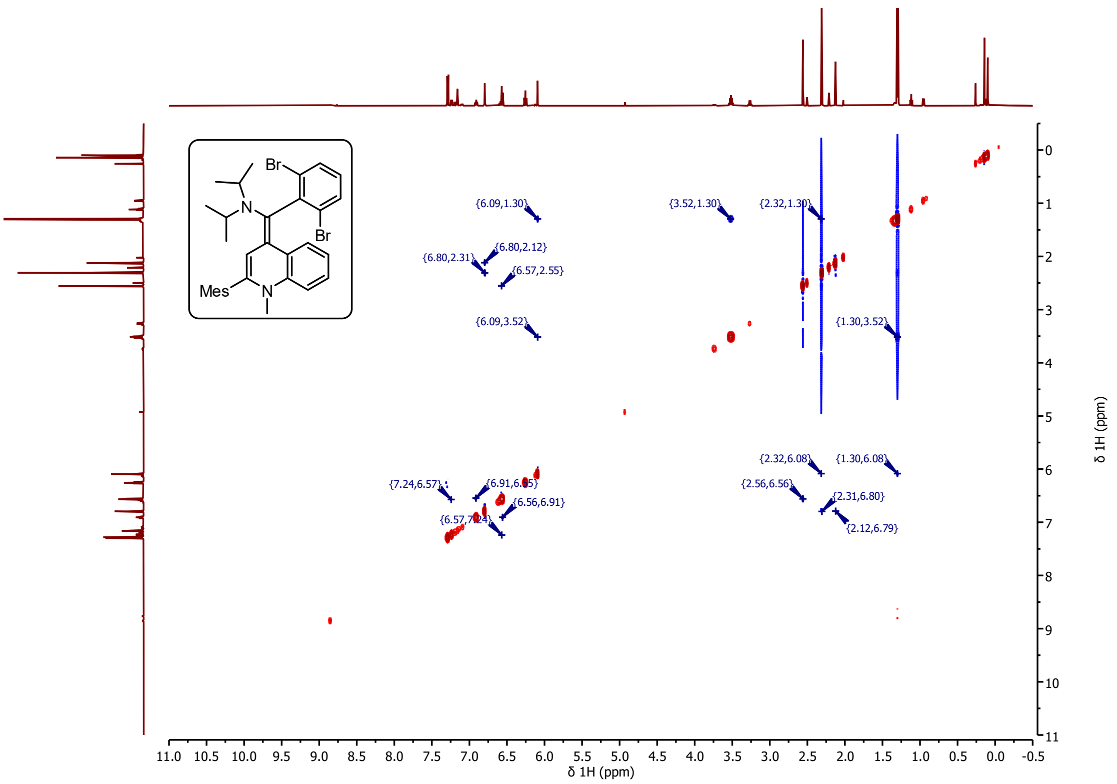

Figure S73: **NOESY-NMR** (501 MHz, 126 MHz, C_6D_6, 298 K) of **2f**.

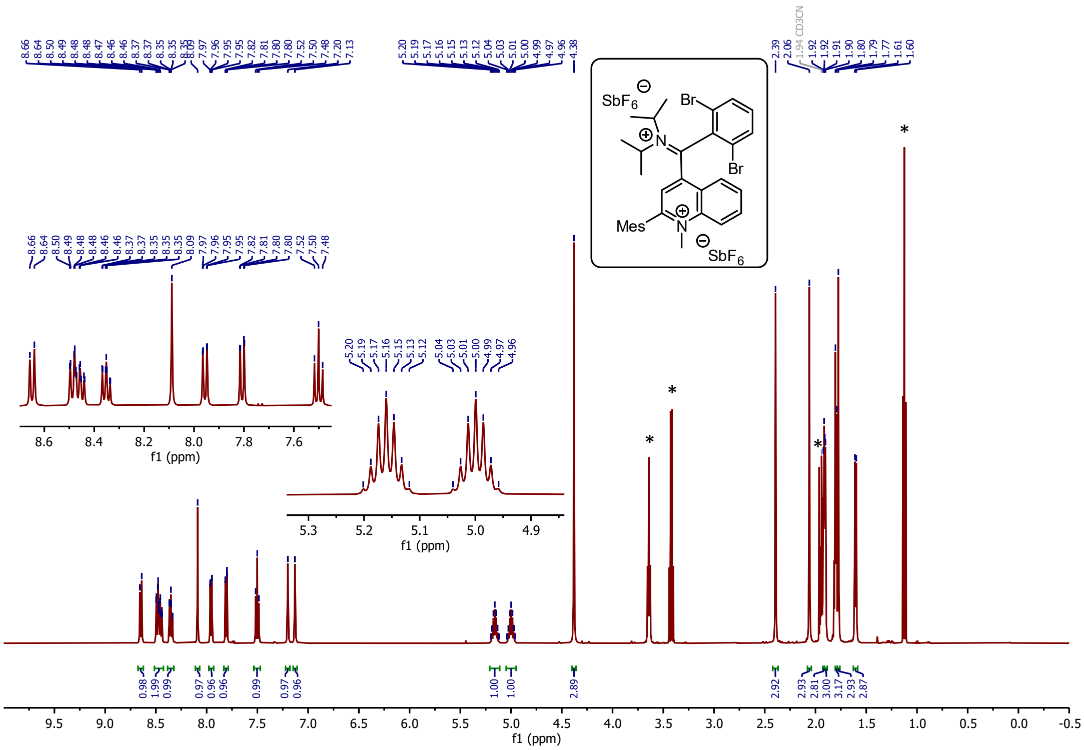

Figure S74: **^{1}H NMR** (501 MHz, CD_3CN, 298 K) of **4f**. (Impurities marked by star: hexamethyldisilazane, diethylether and thf).

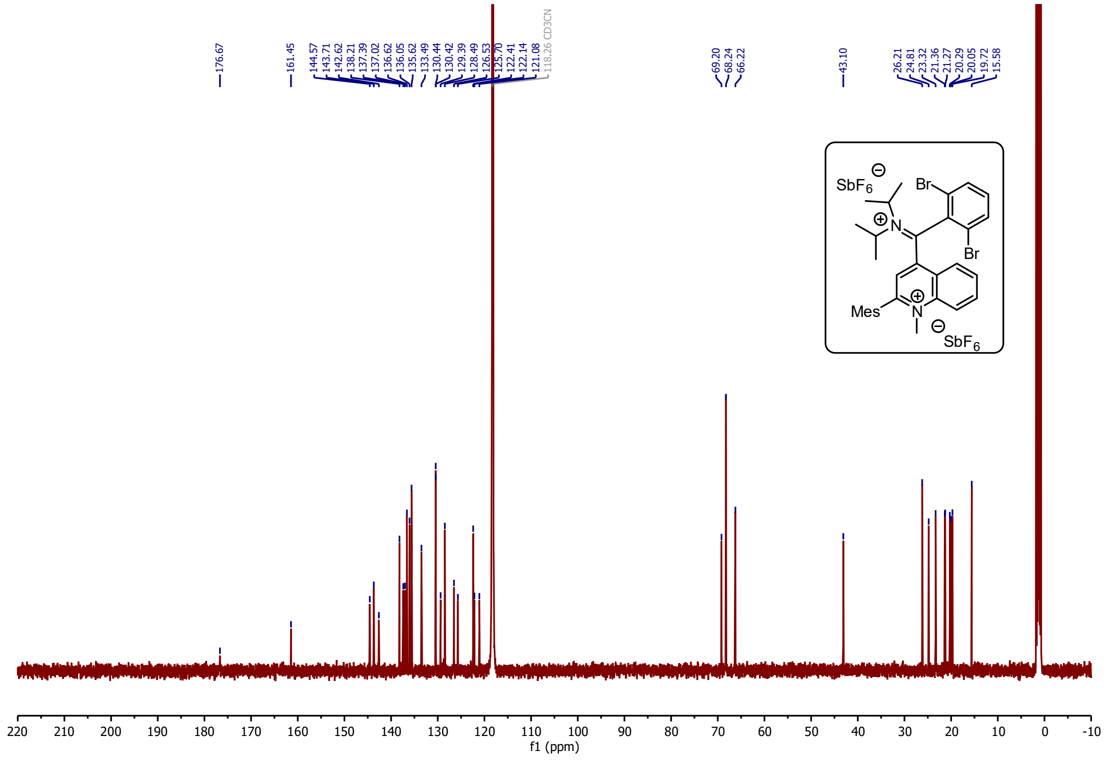

Figure S75: **^{13}C {1H} NMR** (126 MHz, CD_3CN, 298 K) of **4f**.

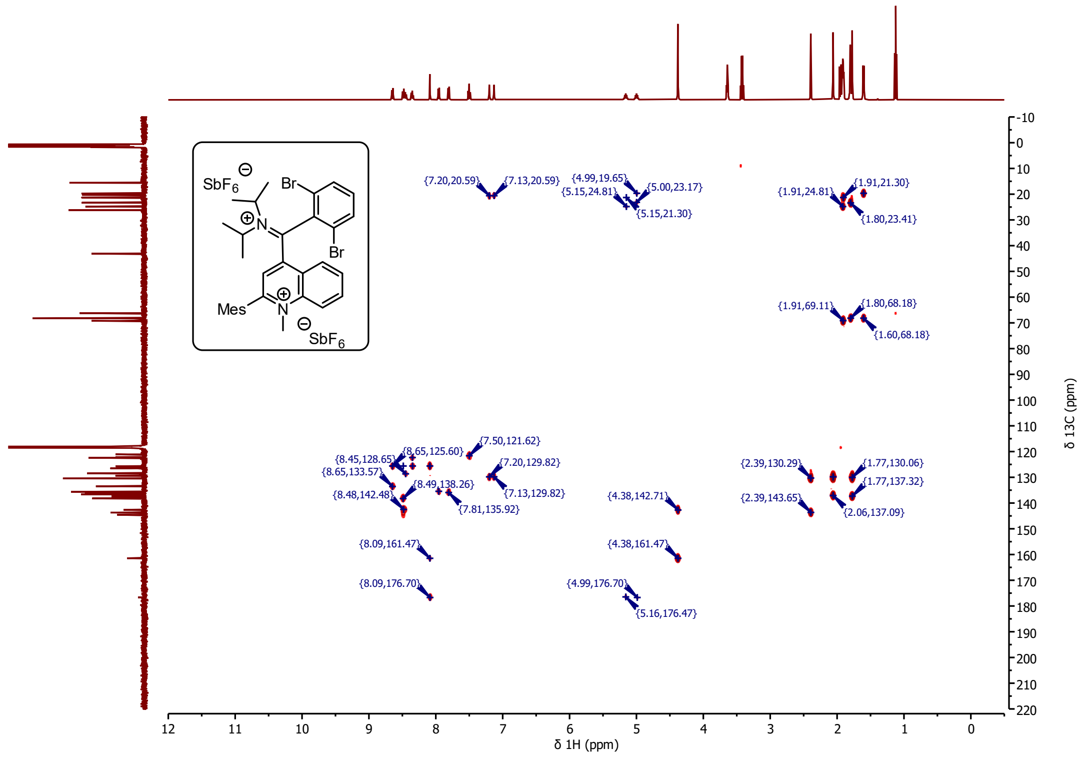

Figure S76: **^{1}H/^{13}C HSQC** (501 MHz/126 MHz, CD_3CN, 298 K) of **4f**.

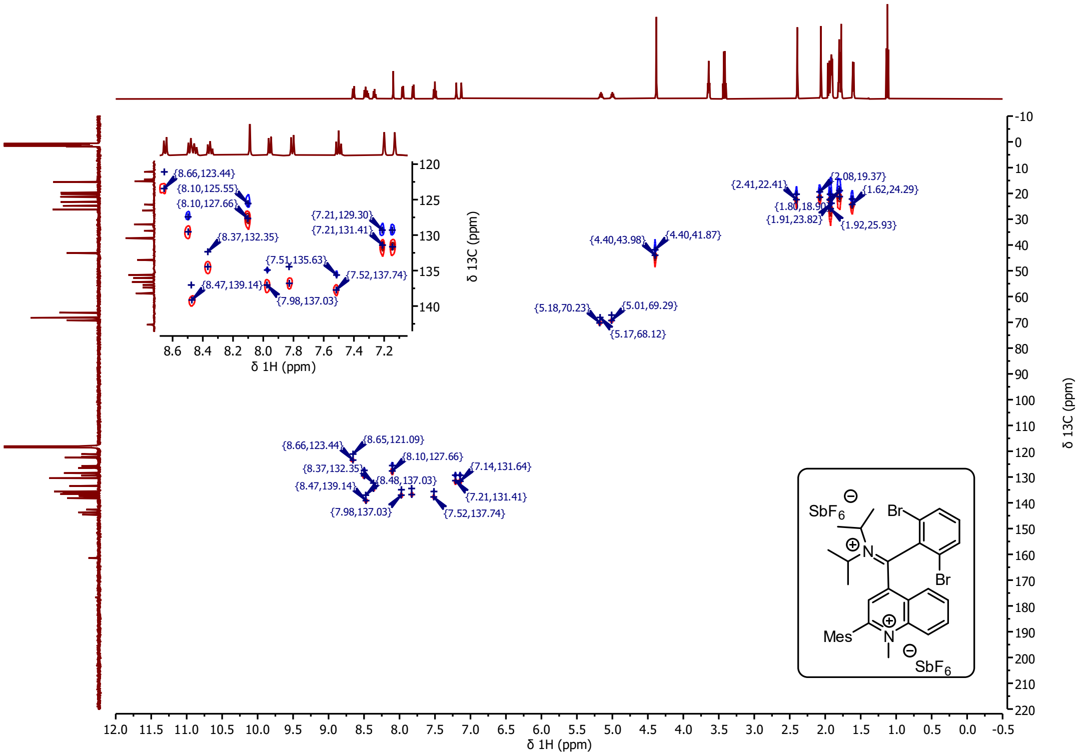

Figure S77: **$^{1}H/^{13}C$ HMBC** (501 MHz/126 MHz, CD_3CN, 298 K) of **4f**.

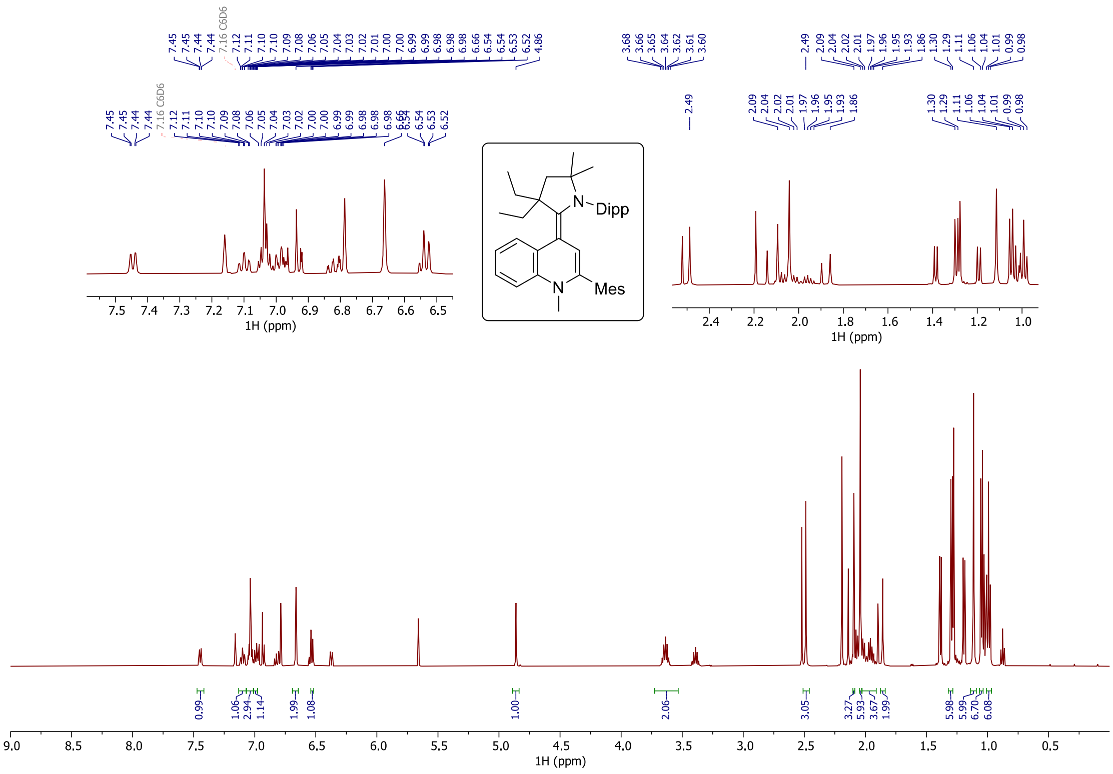

Figure S78: **^{1}H NMR** (500 MHz, C_6D_6, 298K) of **2g**. *E*:*Z* after the reaction = 65:35.

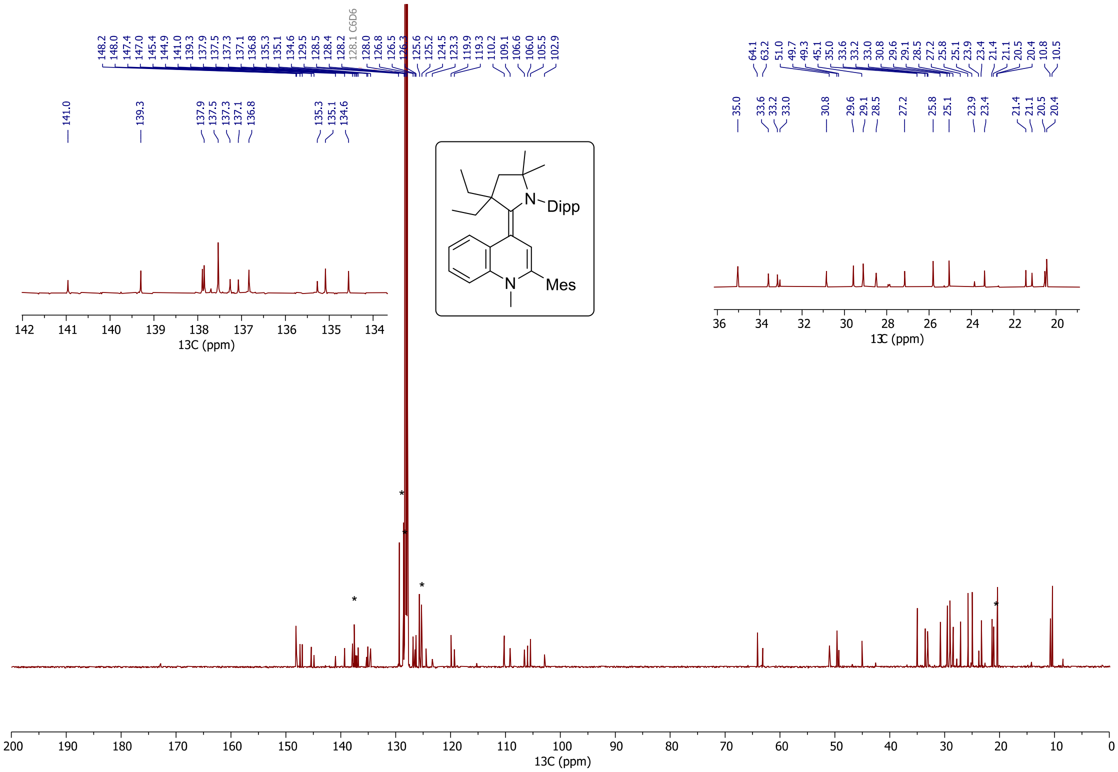

Figure S79: ^{13}C {^{1}H} NMR (126 MHz, C_6D_6, 298K) of **2g**, residual toluene is marked with a *. Due to the complex isomeric mixture it was not possible to clearly assign the signals of only one isomer, instead both are picked.

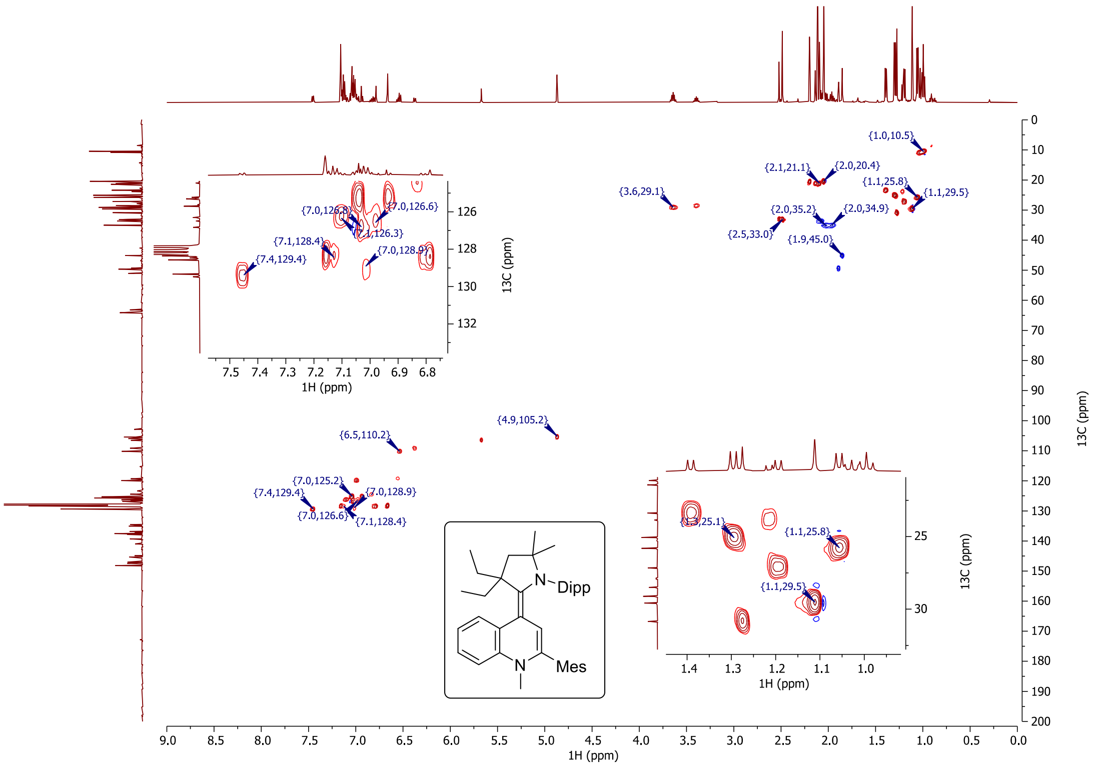

Figure S80: ^{1}H/^{13}C HSQC (500/126 MHz, C_6D_6, 298K) of **2g**.

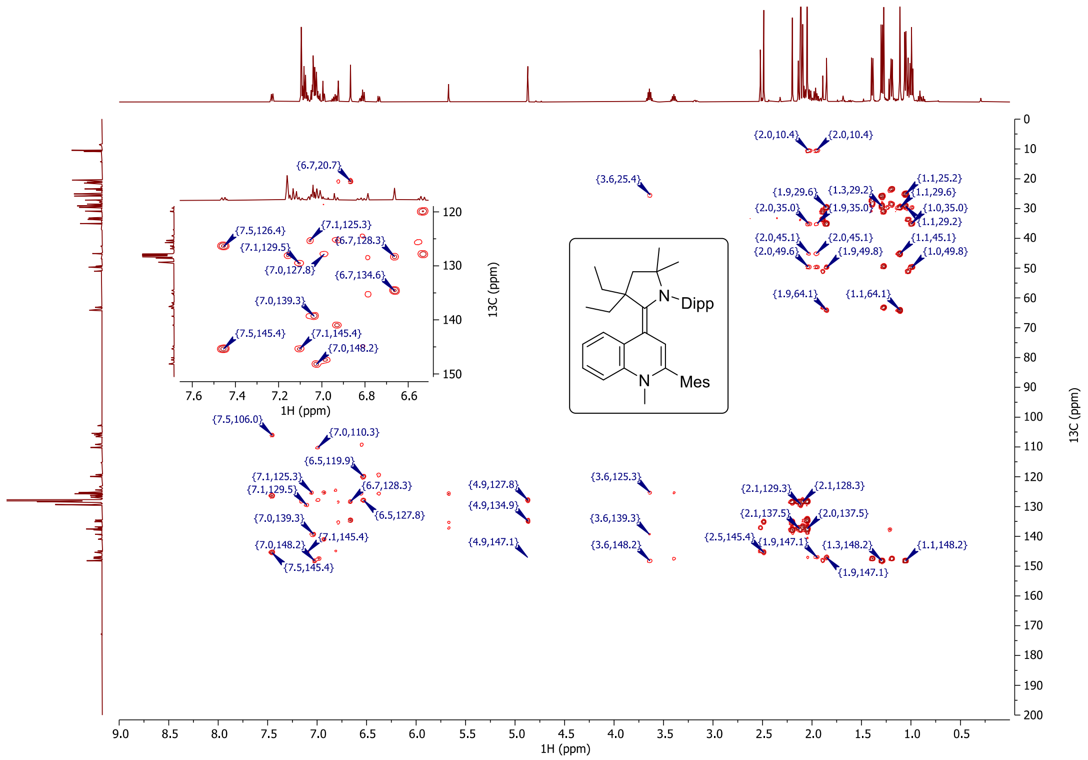

Figure S81: $^{1}H/^{13}C$ HMBC (500/126 MHz, C_6D_6, 298K) of **2g**.

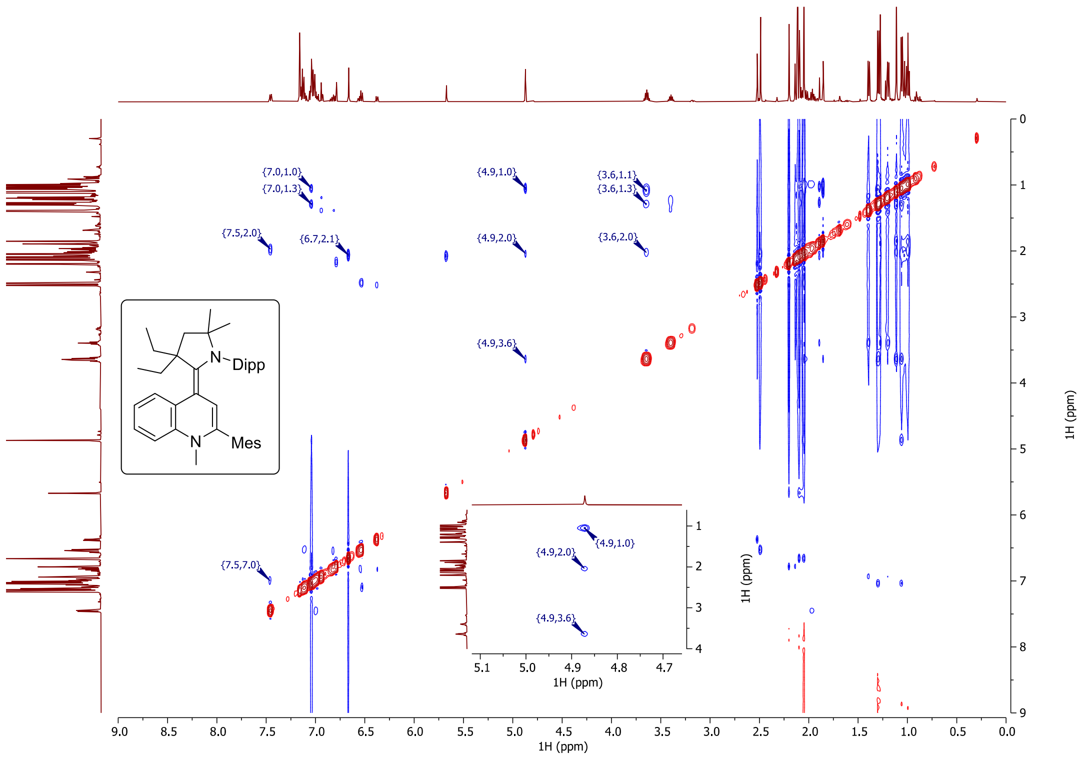

Figure S82: NOESY-NMR (500 MHz, C_6D_6, 298K) of **2g**.

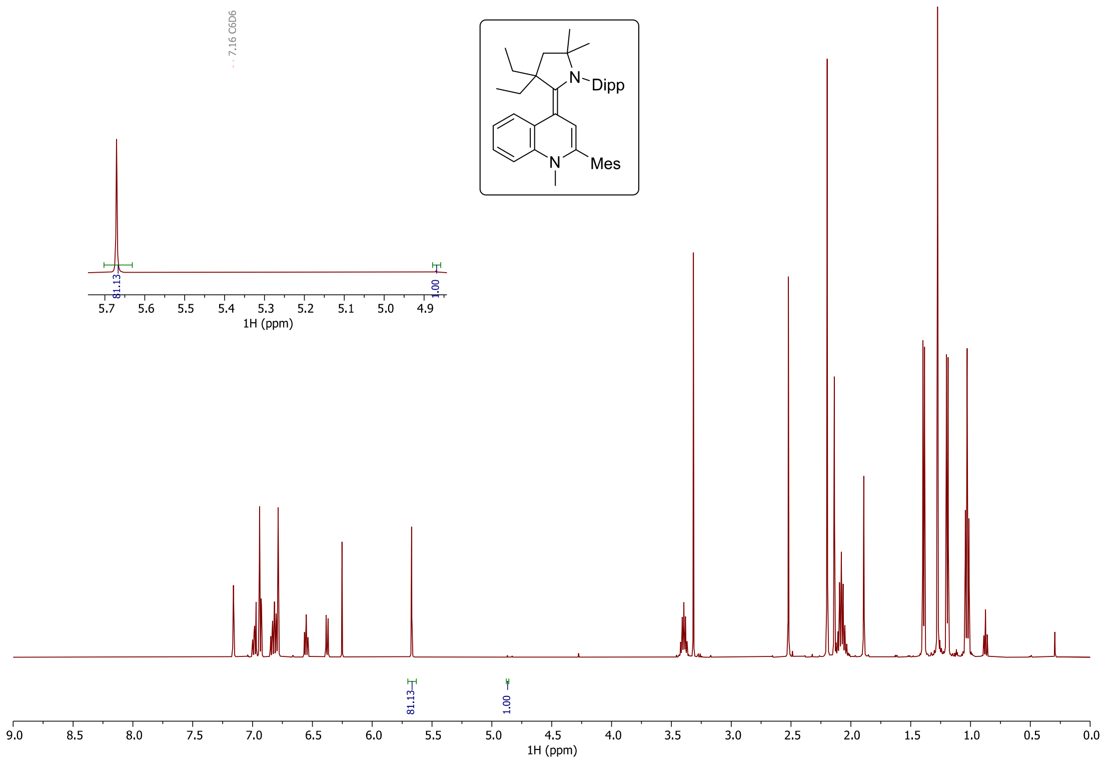

Figure S83: ^{1}**H NMR** (500 MHz, C_6D_6, 298 K) of **2g** after irradiation λ = 467 nm, 1h) *E*:*Z* = 1:99. Contains trimethoxy-benzene as internal standard. Indicative signals are integrated to represent the isomers.

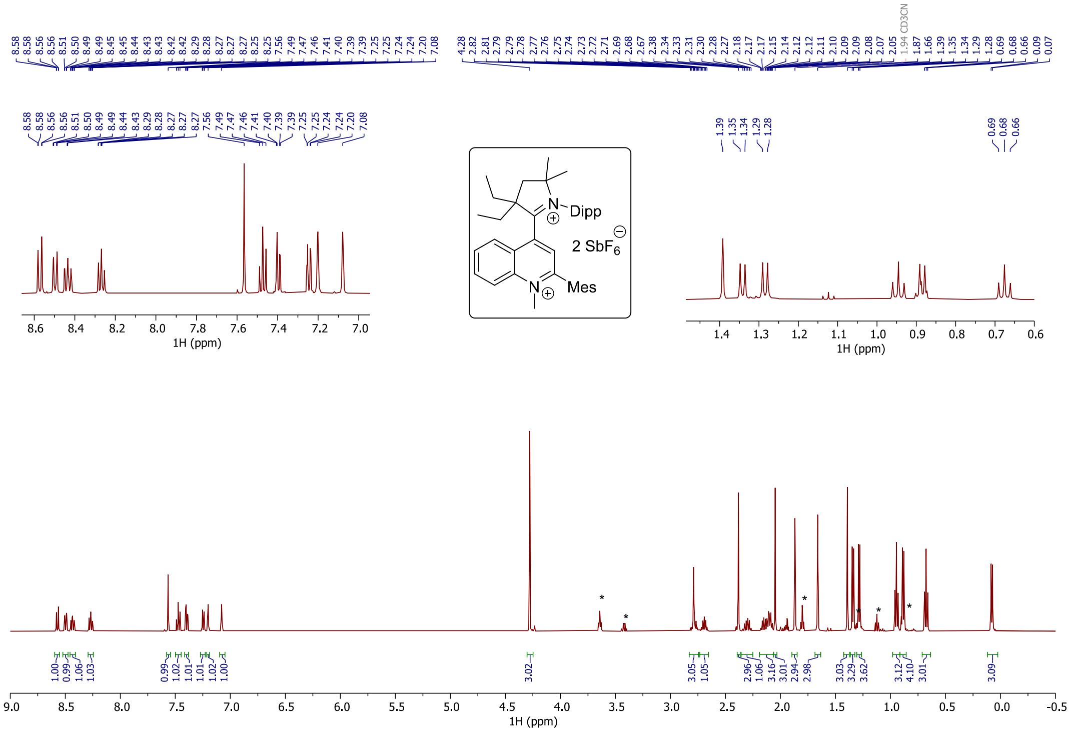

Figure S84: ^{1}**H NMR** (500 MHz, CD_3CN, 298K) of **4g**, residual thf, Et_2O, pentane is marked with a *.

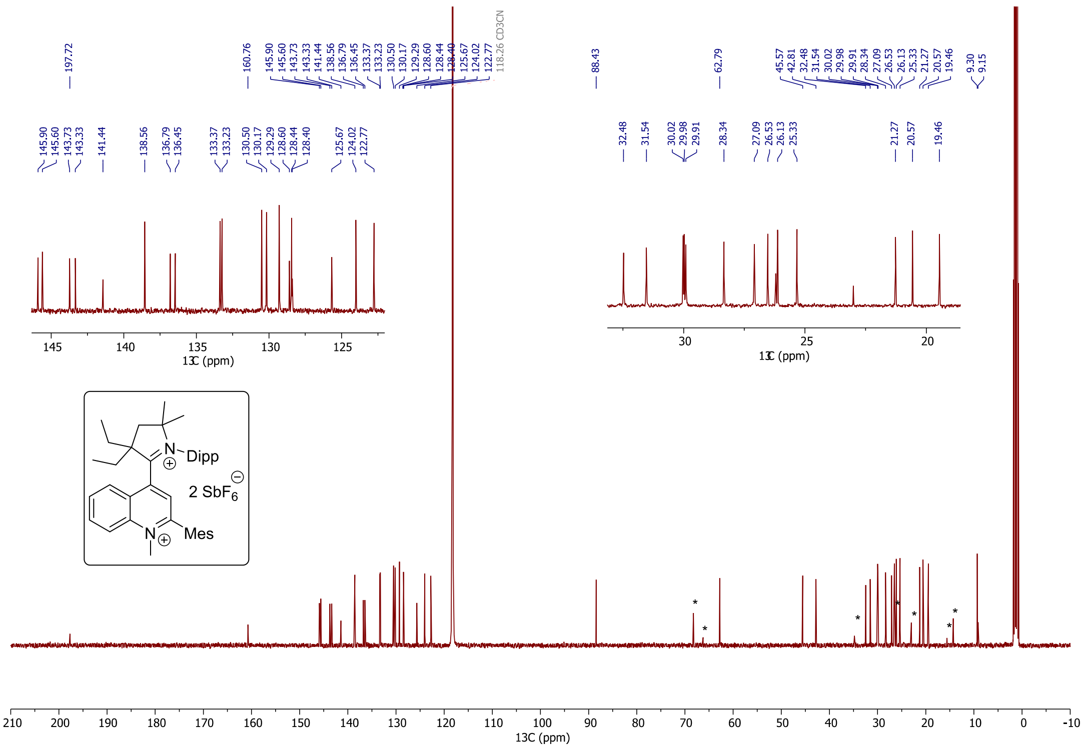

Figure S85: ^{13}C {^{1}H} NMR (126 MHz, CD_3CN, 298K) of **4g**, residual thf, Et_2O, pentane is marked with a *.

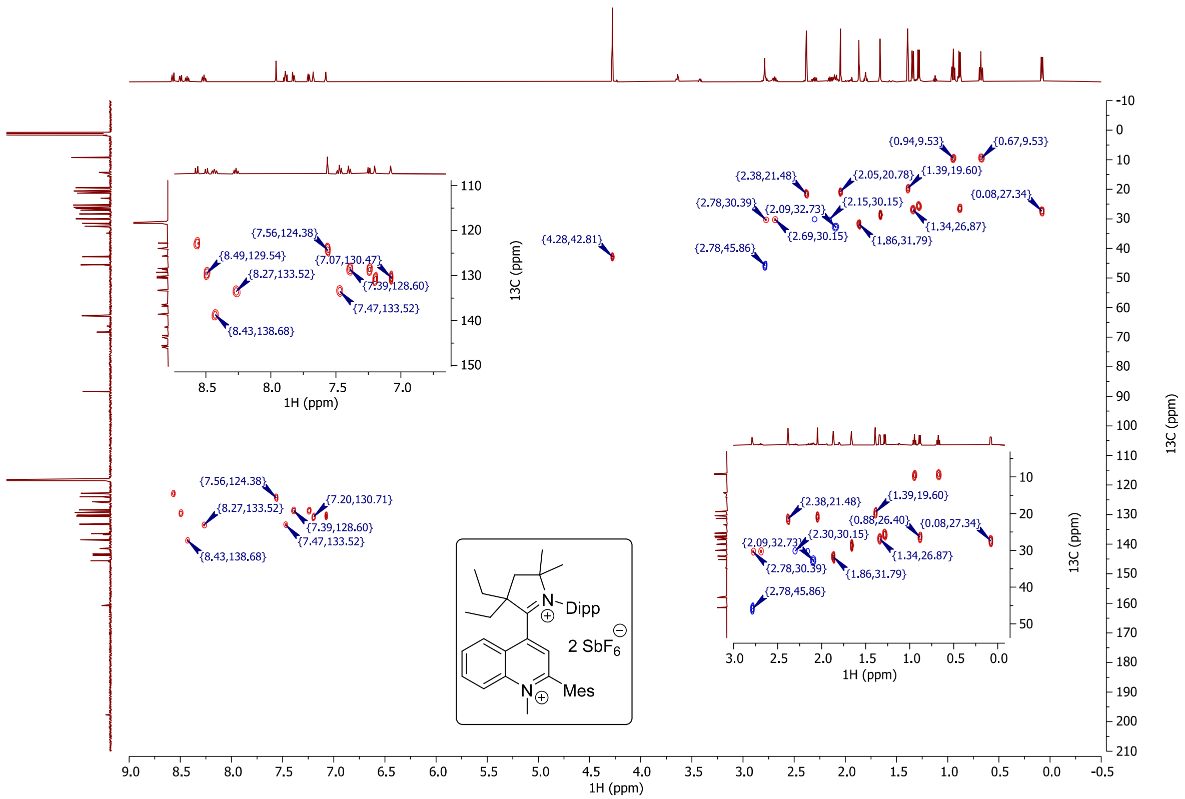

Figure S86: ^{1}H/^{13}C HSQC (500/126 MHz, CD_3CN, 298K) of **4g**.

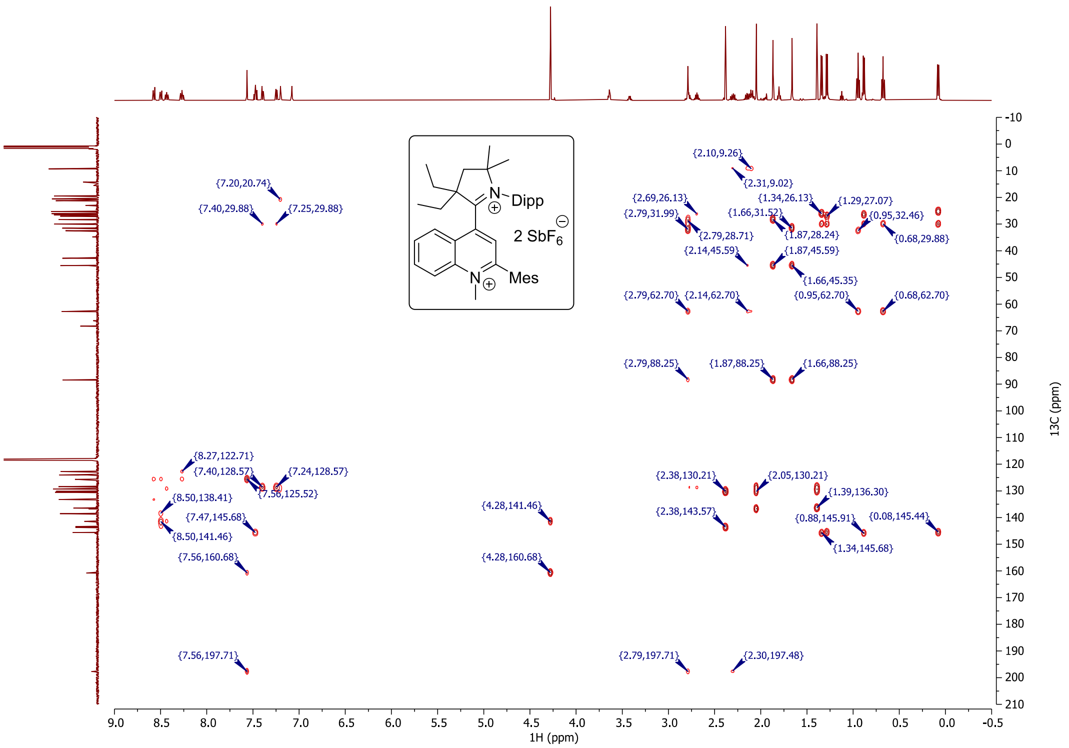

Figure S87: $^{1}H/^{13}C$ HMBC (500/126 MHz, CD_3CN, 298K) of **4g**.

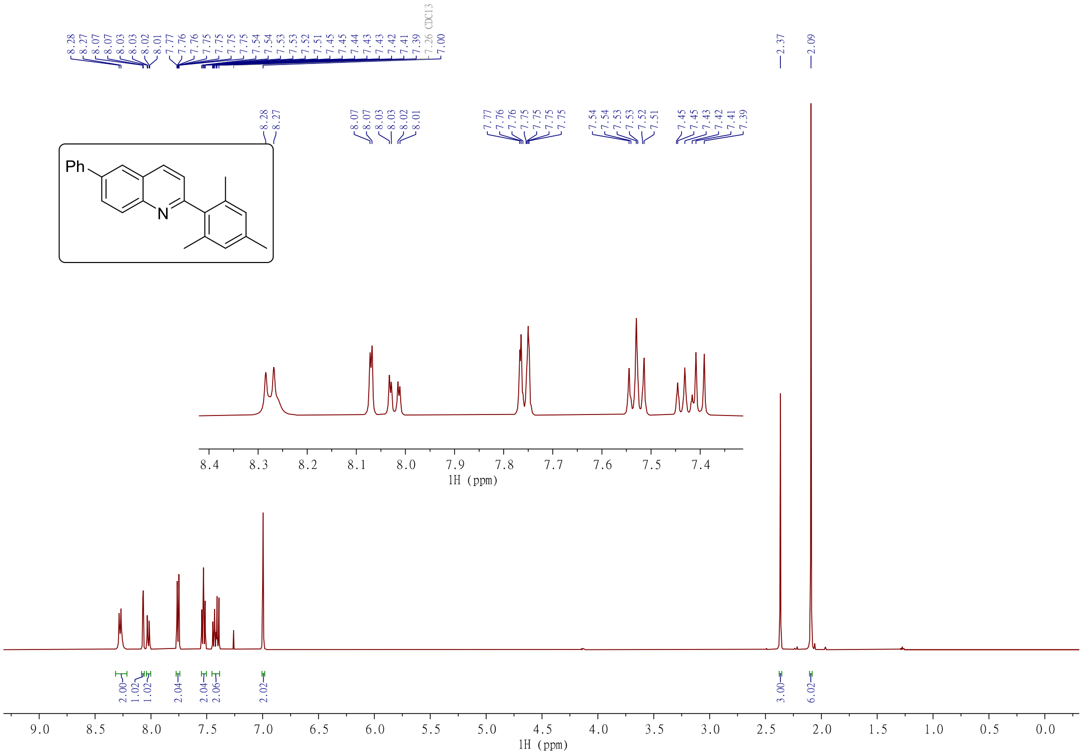

Figure S88: ^{1}H NMR (500 MHz, $CDCl_3$, 298K) of **S9**.

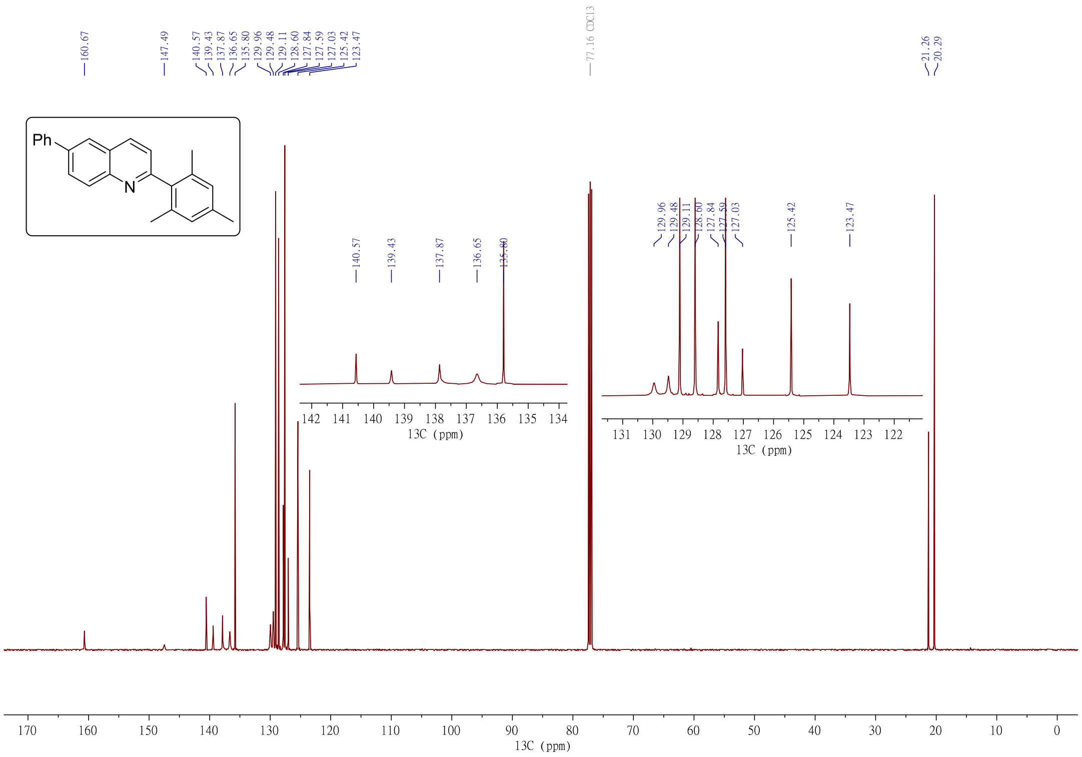

Figure S89: ^{13}C {^{1}H} NMR (126 MHz, $CDCl_3$, 298K) of **S9**.

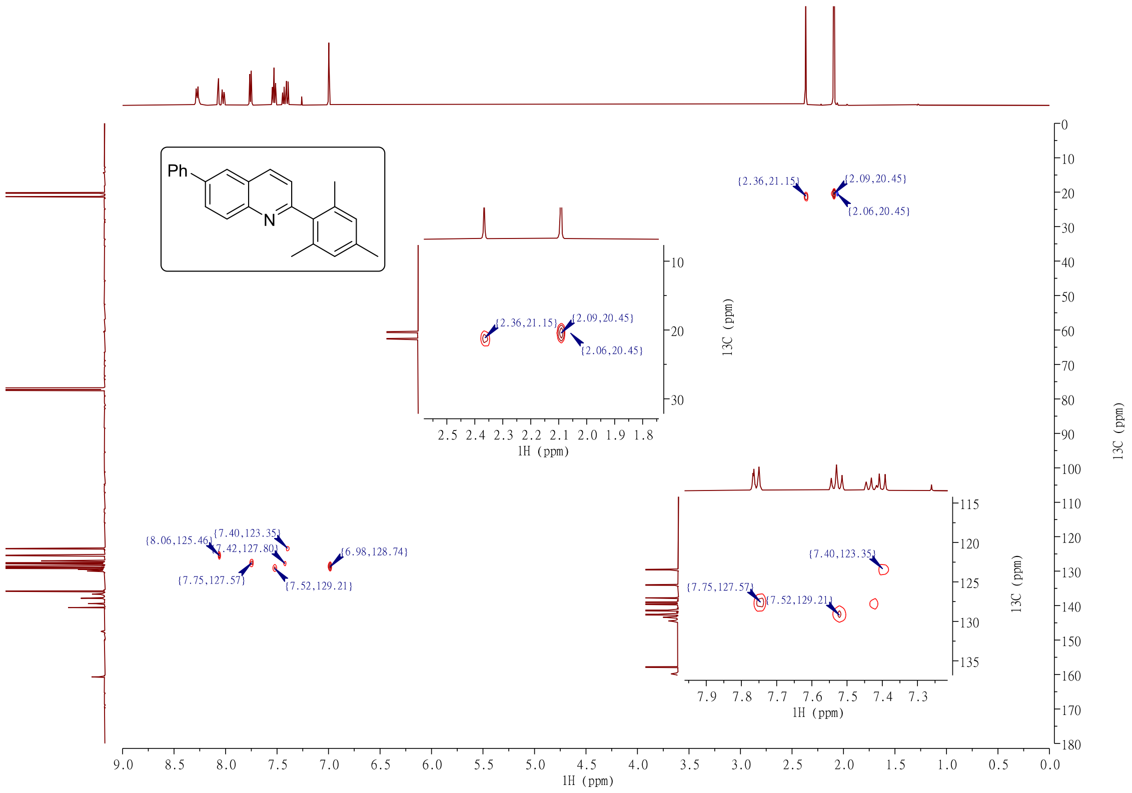

Figure S90: ^{1}H/^{13}C HSQC (500/126 MHz, $CDCl_3$, 298K) of **S9**.

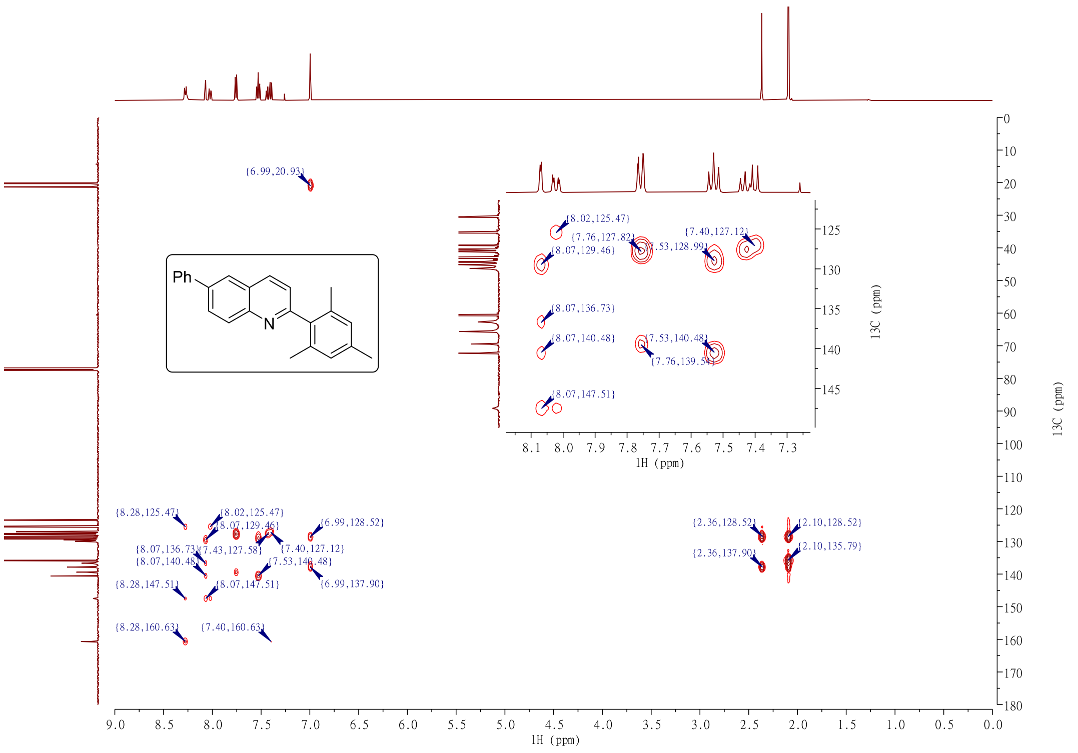

Figure S91: **^{1}H/^{13}C HMBC** (500/126 MHz, $CDCl_3$, 298K) of **S9**.

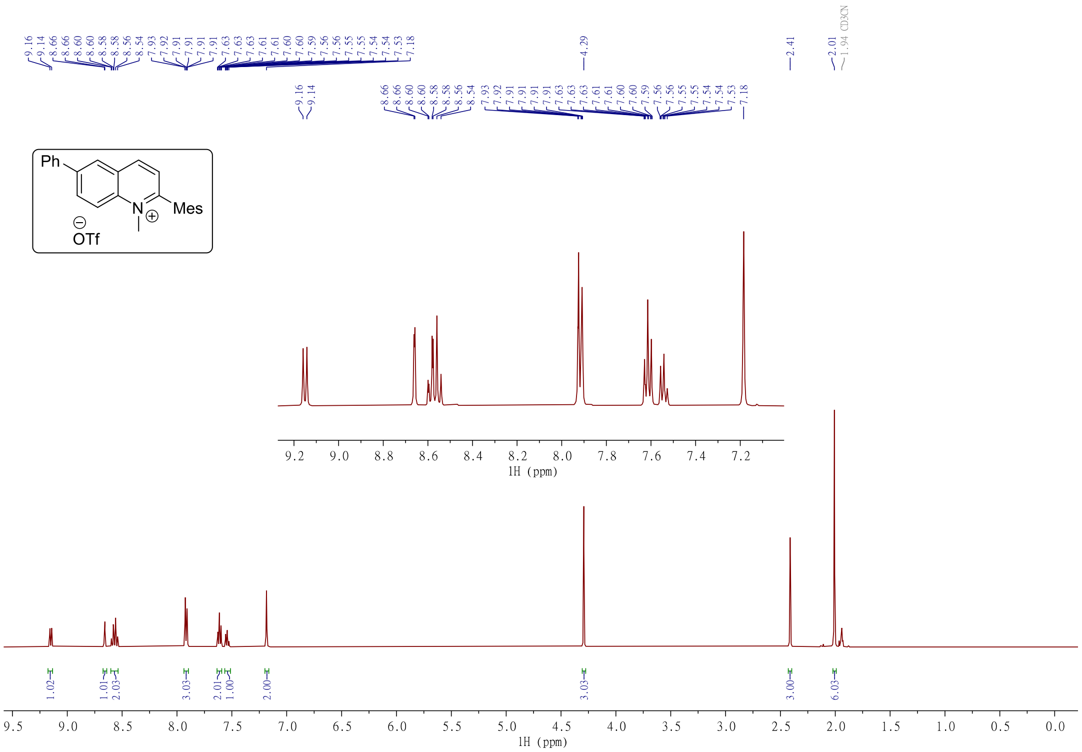

Figure S92: **^{1}H NMR** (500 MHz, CD_3CN, 298K) of **1h**.

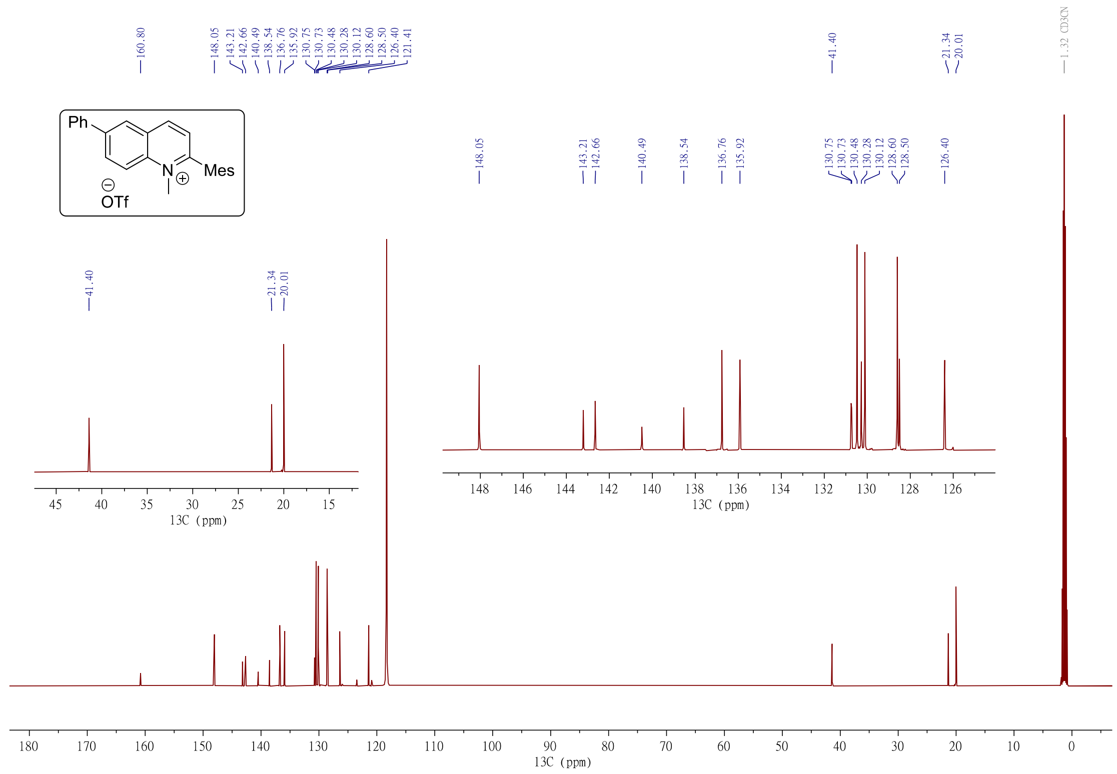

Figure S93: ^{13}**C {**^{1}**H} NMR** (126 MHz, CD_3CN, 298K) of **1h**.

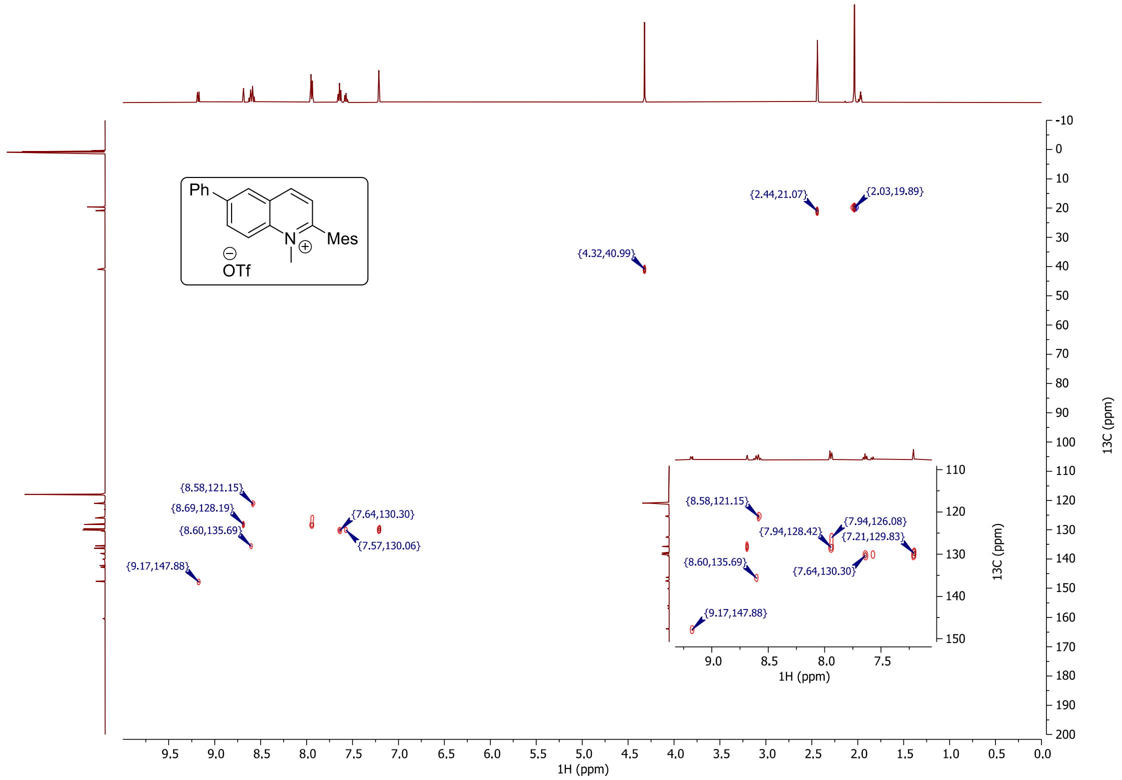

Figure S94: ^{1}**H/**^{13}**C HSQC** (500/126 MHz, CD_3CN, 298K) of **1h**.

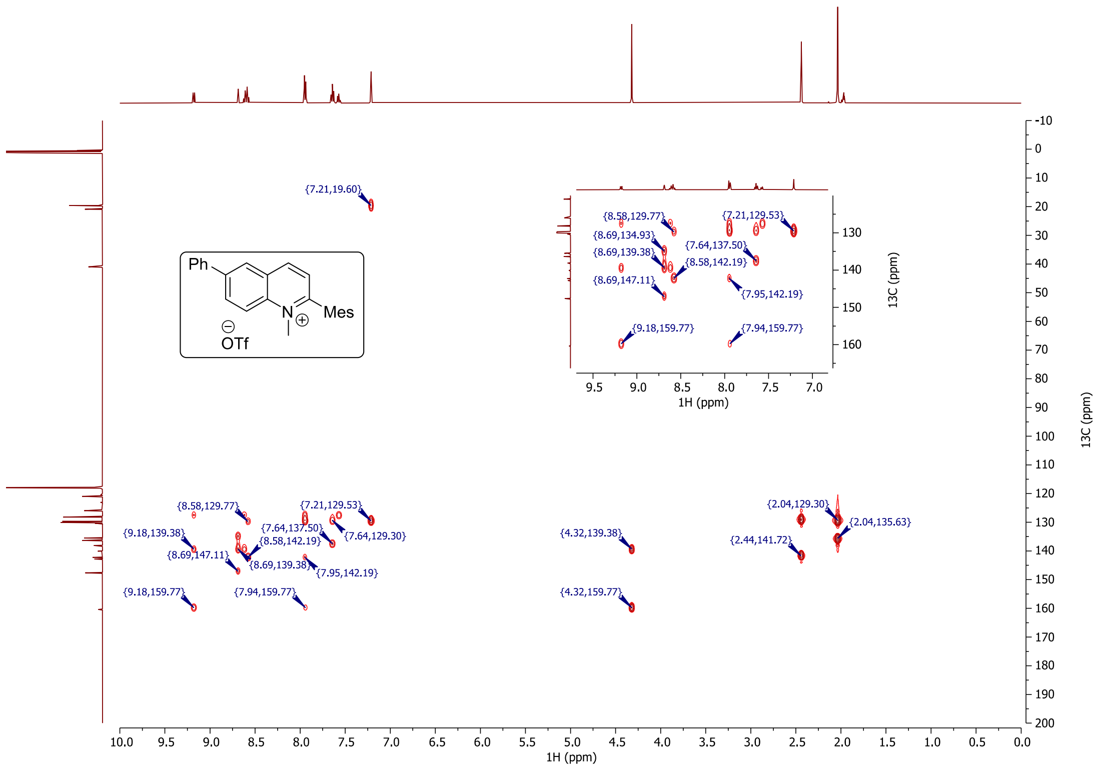

Figure S95: $^1H/^{13}C$ HMBC (500/126 MHz, CD_3CN, 298K) of **1h**.

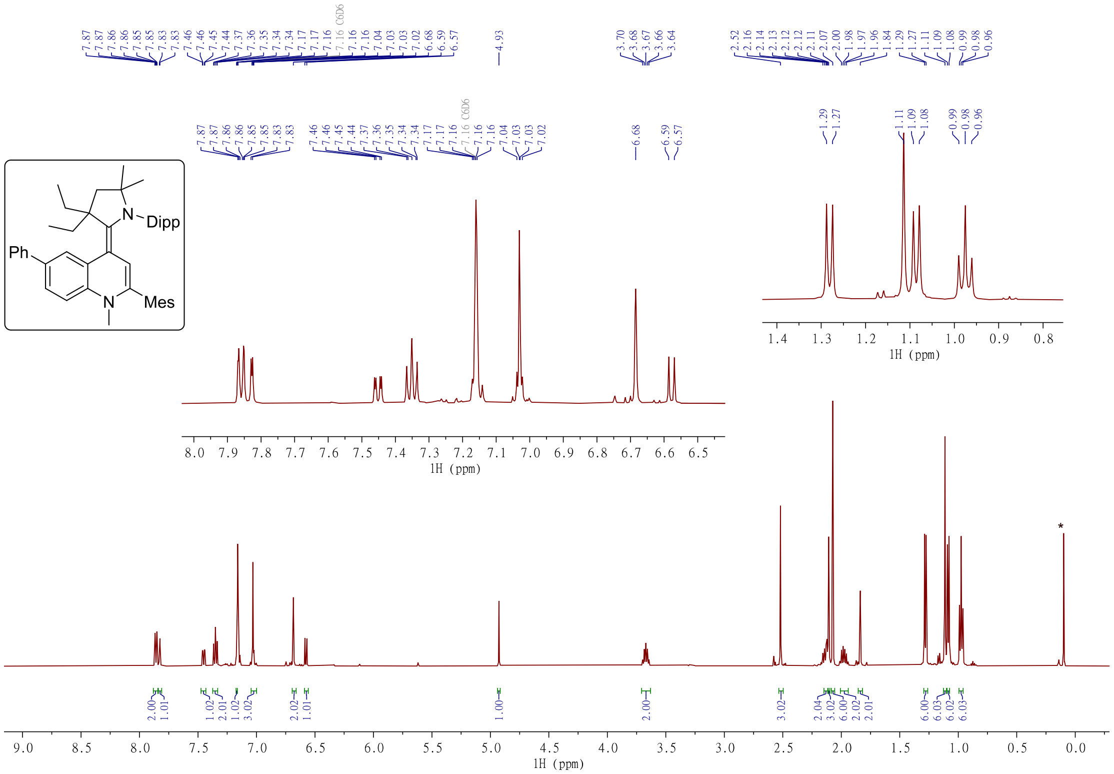

Figure S96: ^1H NMR (500 MHz, C_6D_6, 298K) of **2h**, residual KHMDS is marked with a *.

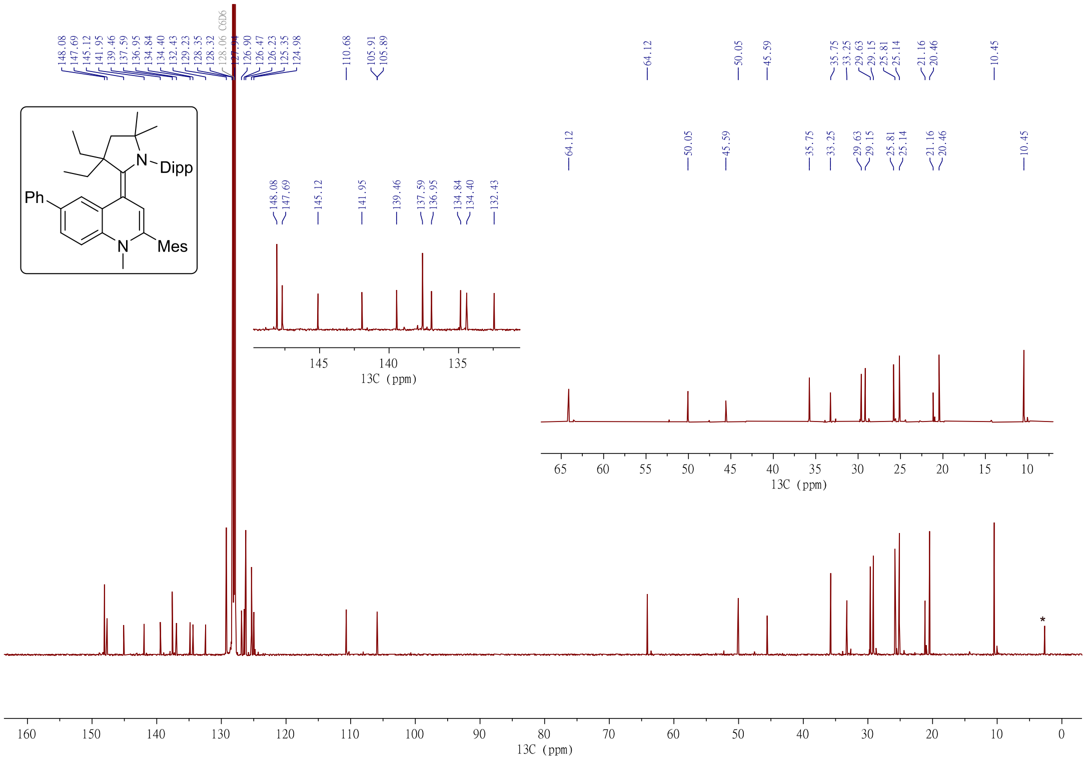

Figure S97: ^{13}**C {**^{1}**H} NMR** (126 MHz, C_6D_6, 298K) of **2h**, residual KHMDS is marked with a *.

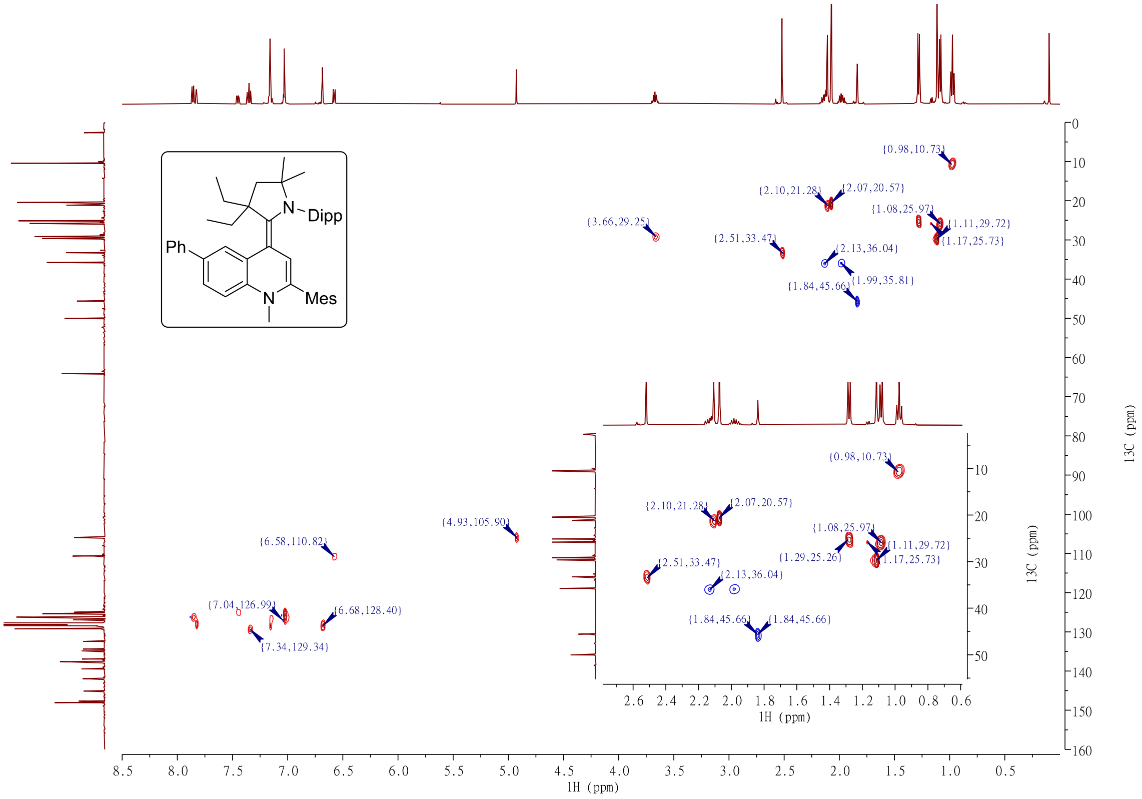

Figure S98: ^{1}**H/**^{13}**C HSQC** (500/126 MHz, C_6D_6, 298K) of **2h**.

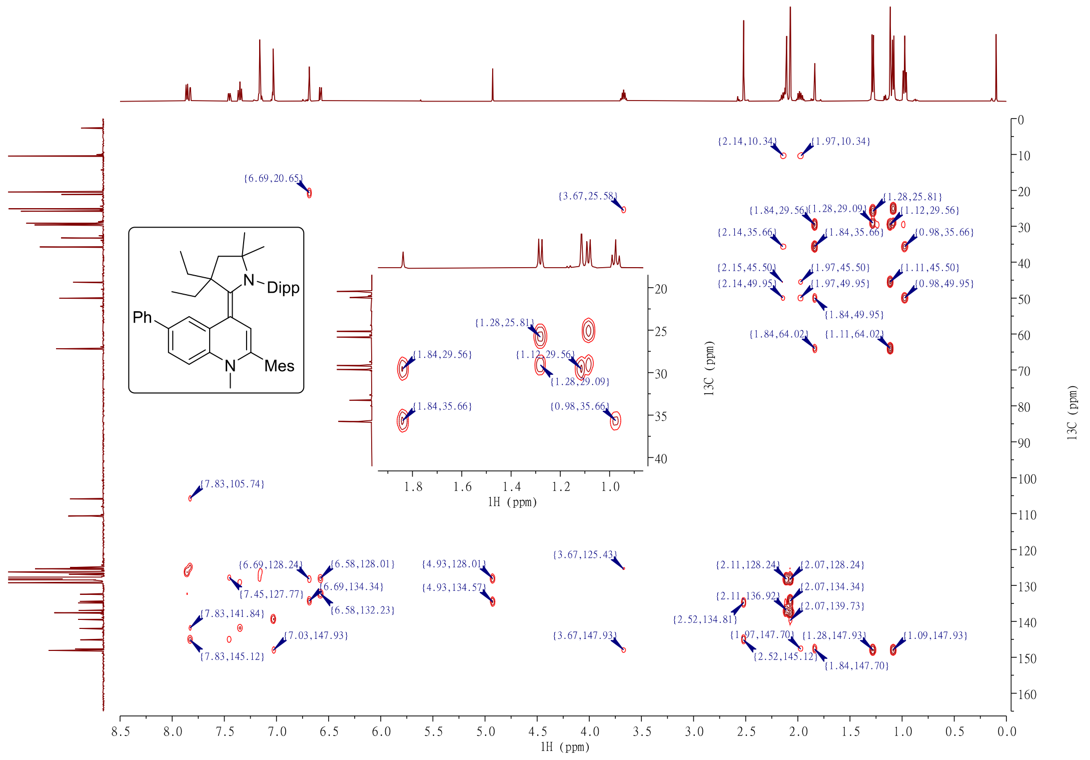

Figure S99: $^1H/^{13}C$ HMBC (500/126 MHz, C_6D_6, 298K) of **2h**.

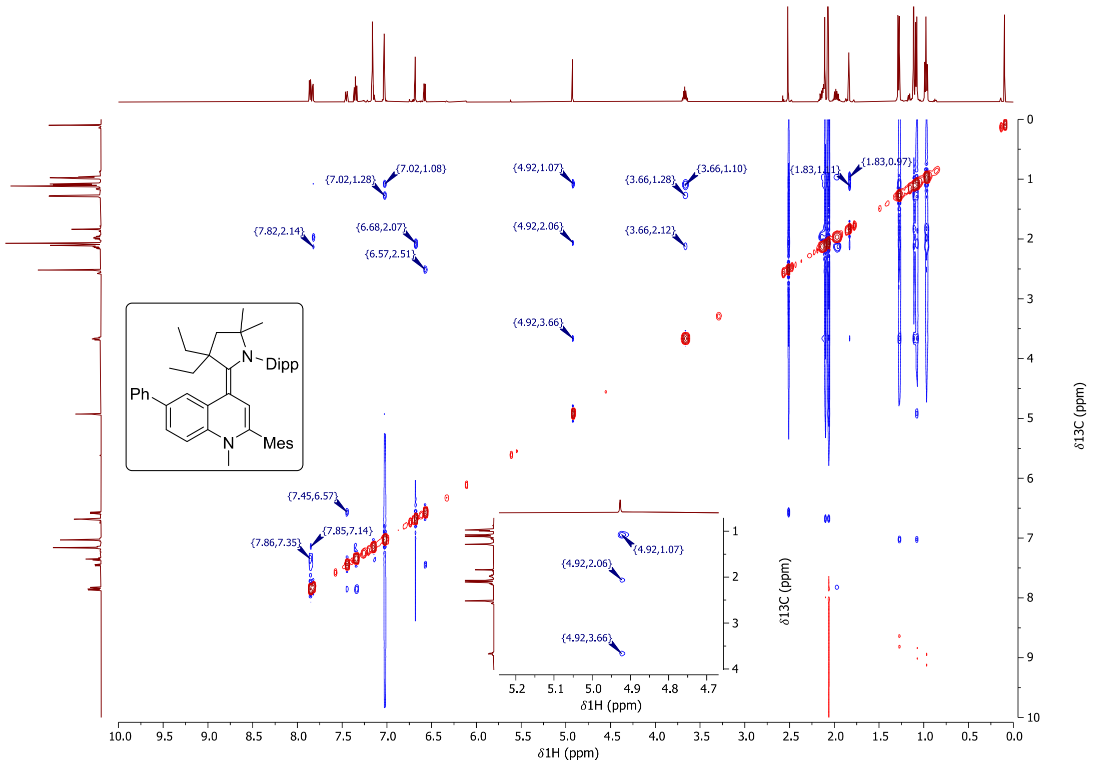

Figure S100: NOESY-NMR (500 MHz, C_6D_6, 298K) of **2h**.

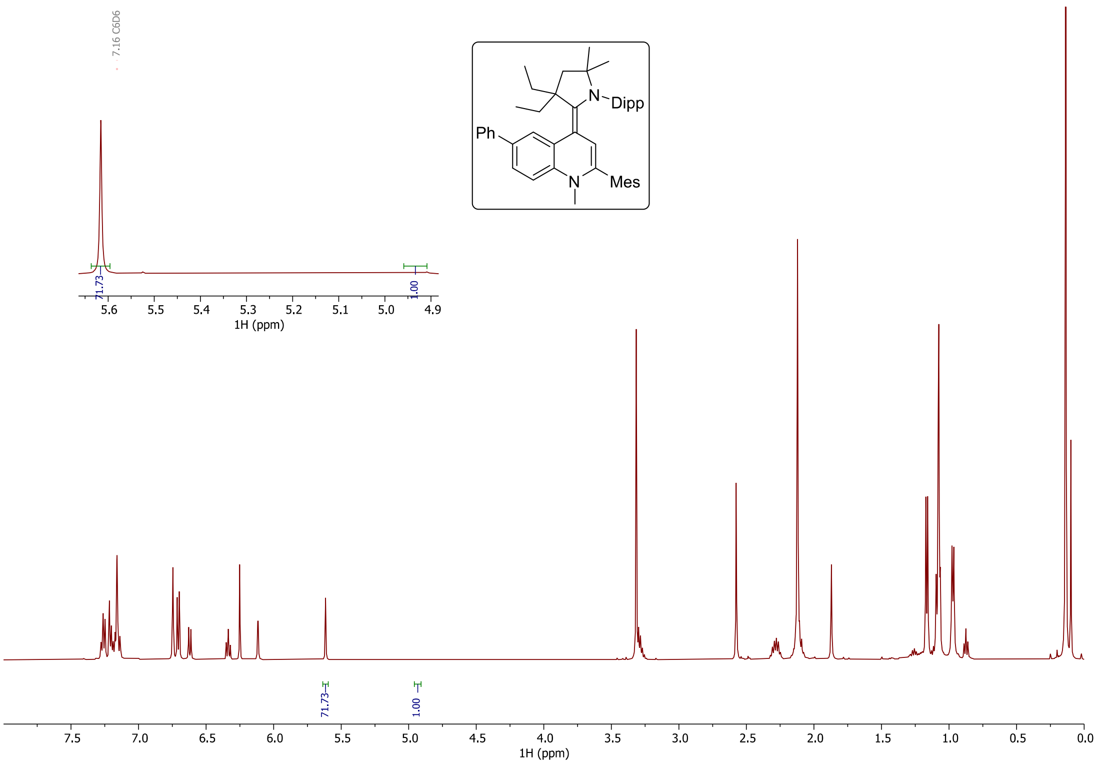

Figure S101: ^{1}**H NMR** (500 MHz, C_6D_6, 298 K) of **2h** after irradiation λ = 525 nm, 1h; *E*:*Z* = 1:99. Contains trimethoxy-benzene as internal standard and KHMDS. Indicative signals are integrated to represent the isomers.

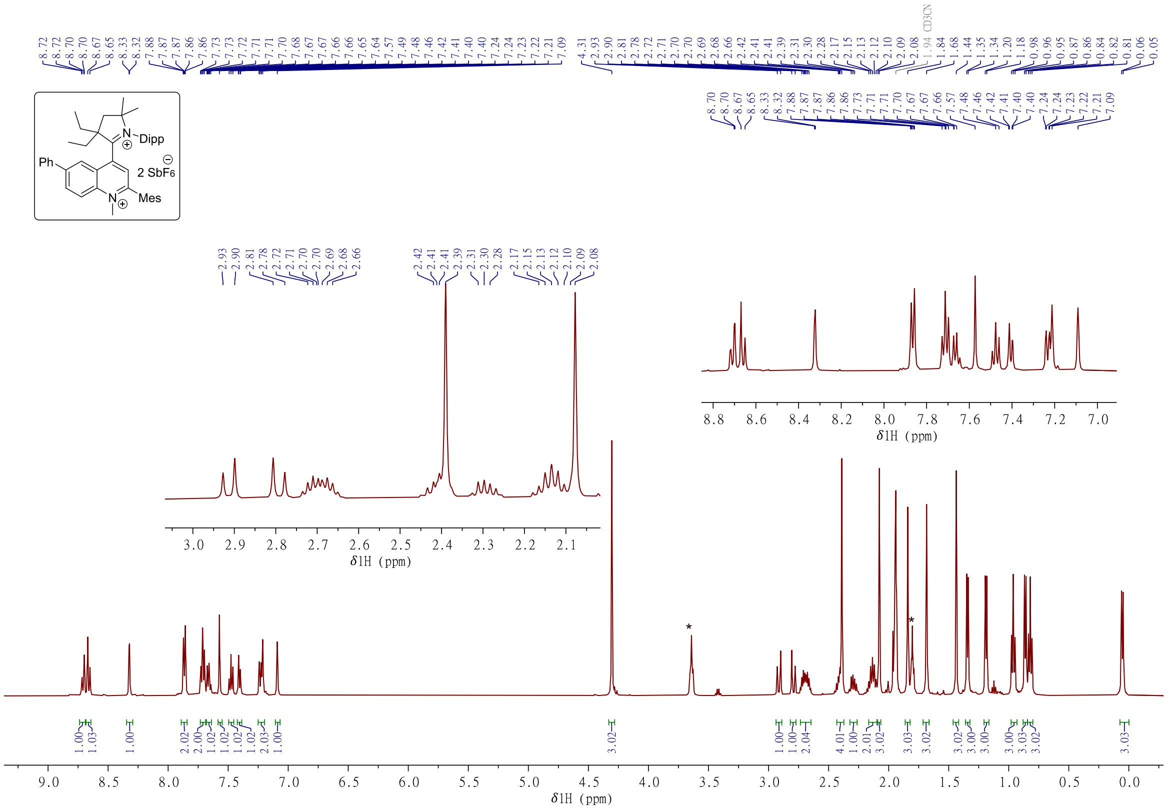

Figure S102: ^{1}**H NMR** (500 MHz, CD_3CN, 298K) of **4h**, residual thf is marked with a *.

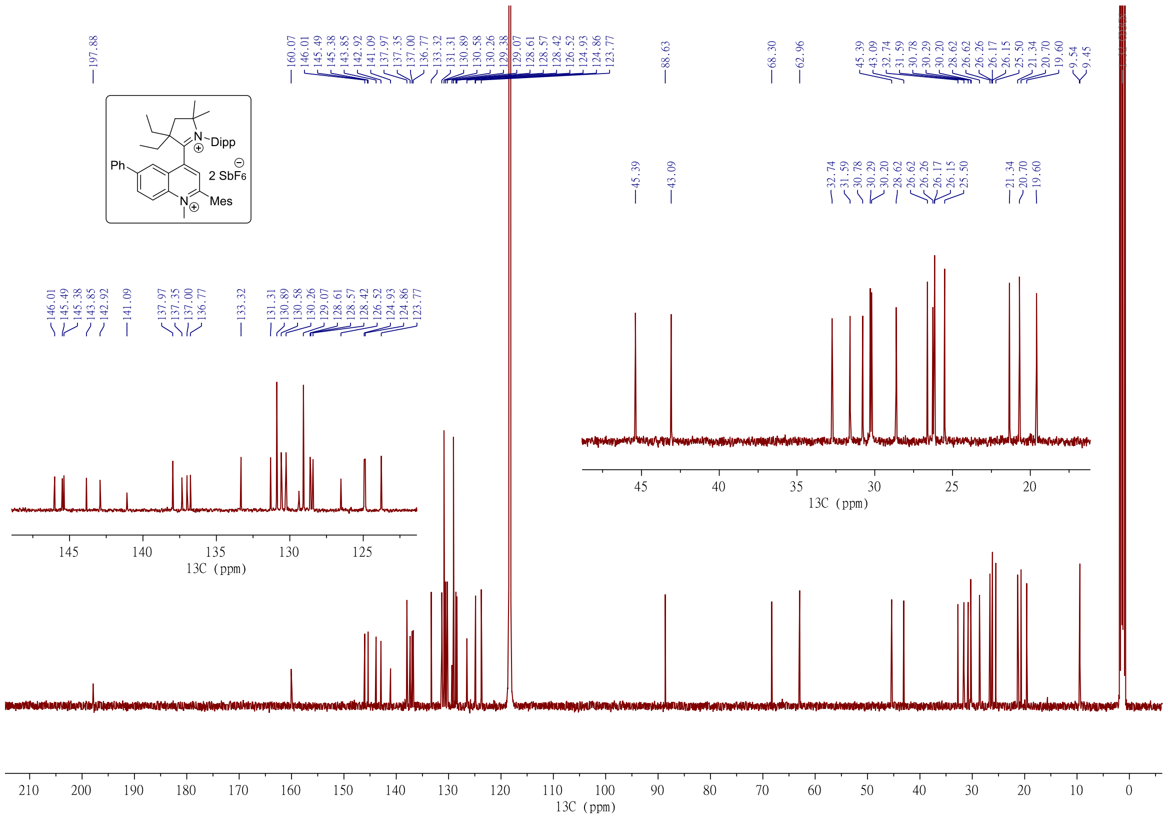

Figure S103: ^{13}C {^{1}H} NMR (126 MHz, CD_3CN, 298K) of **4h**, residual thf is marked with a *.

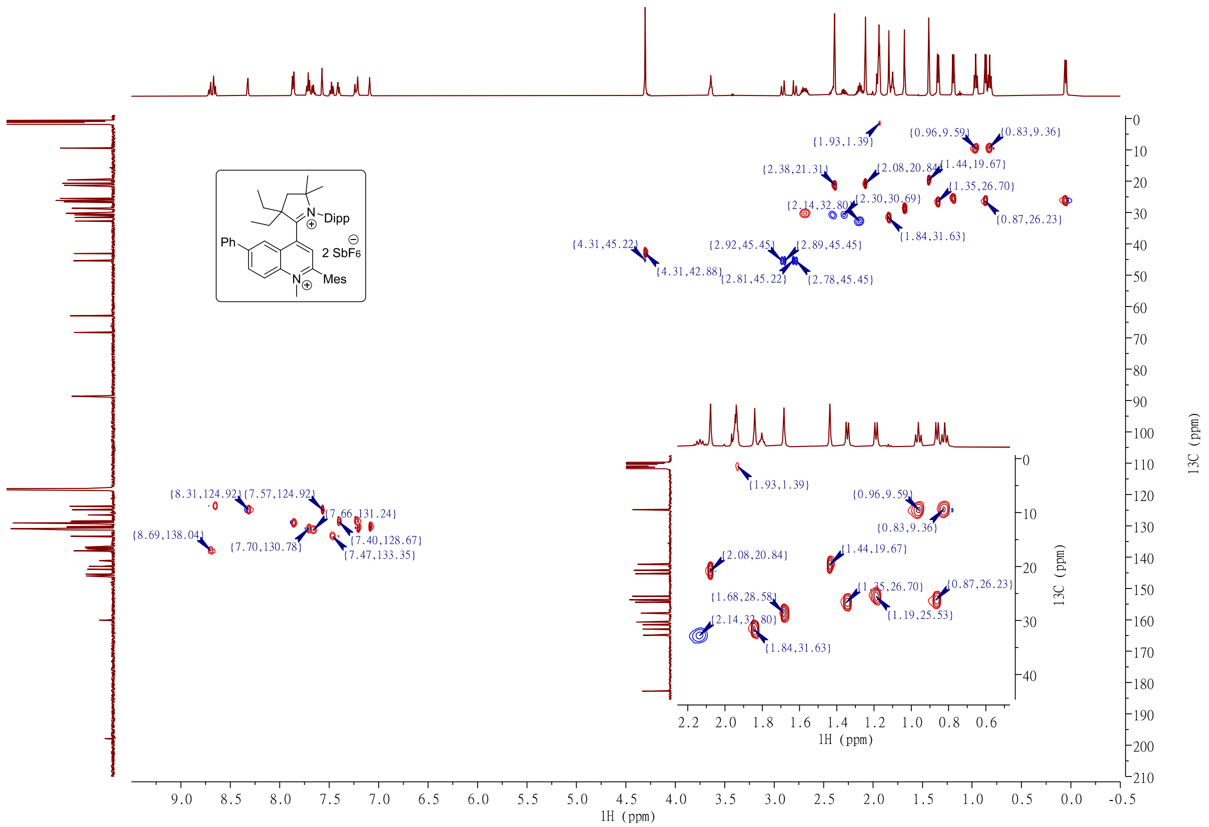

Figure S104: ^{1}H/^{13}C HSQC (500/126 MHz, CD_3CN, 298K) of **4h**.

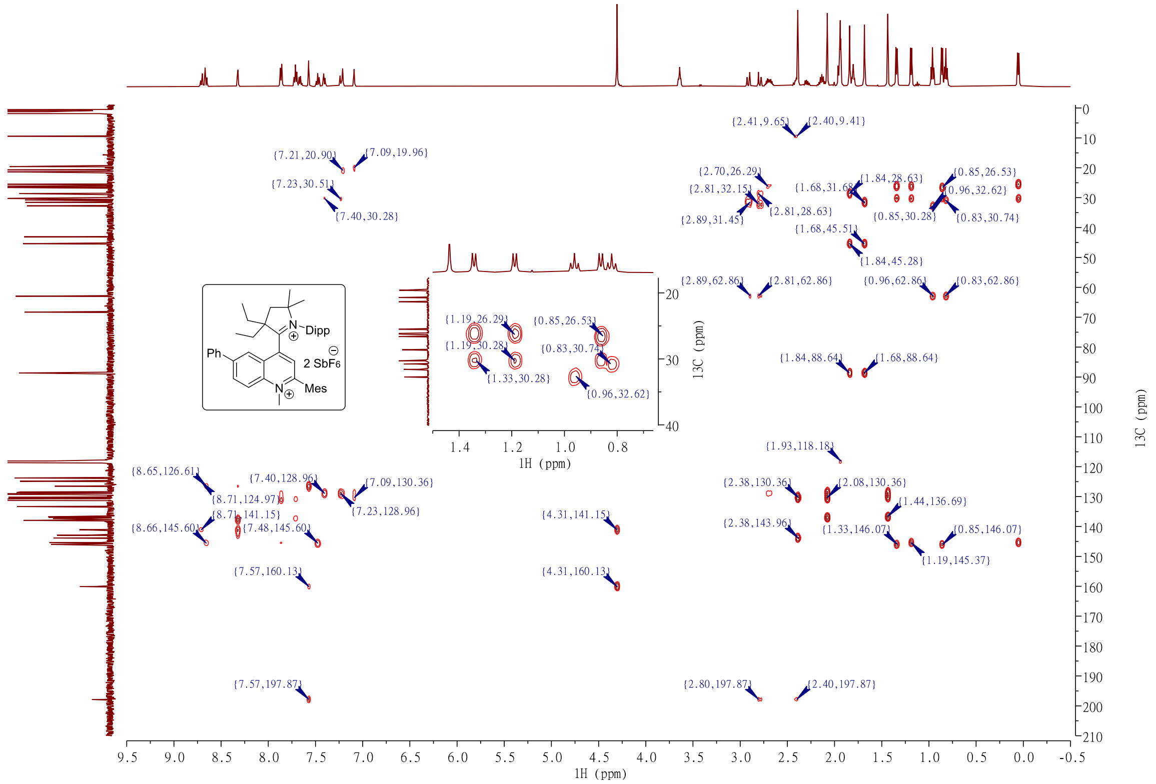

Figure S105: ^{1}H/^{13}C HMBC (500/126 MHz, CD_3CN, 298K) of **4h**.

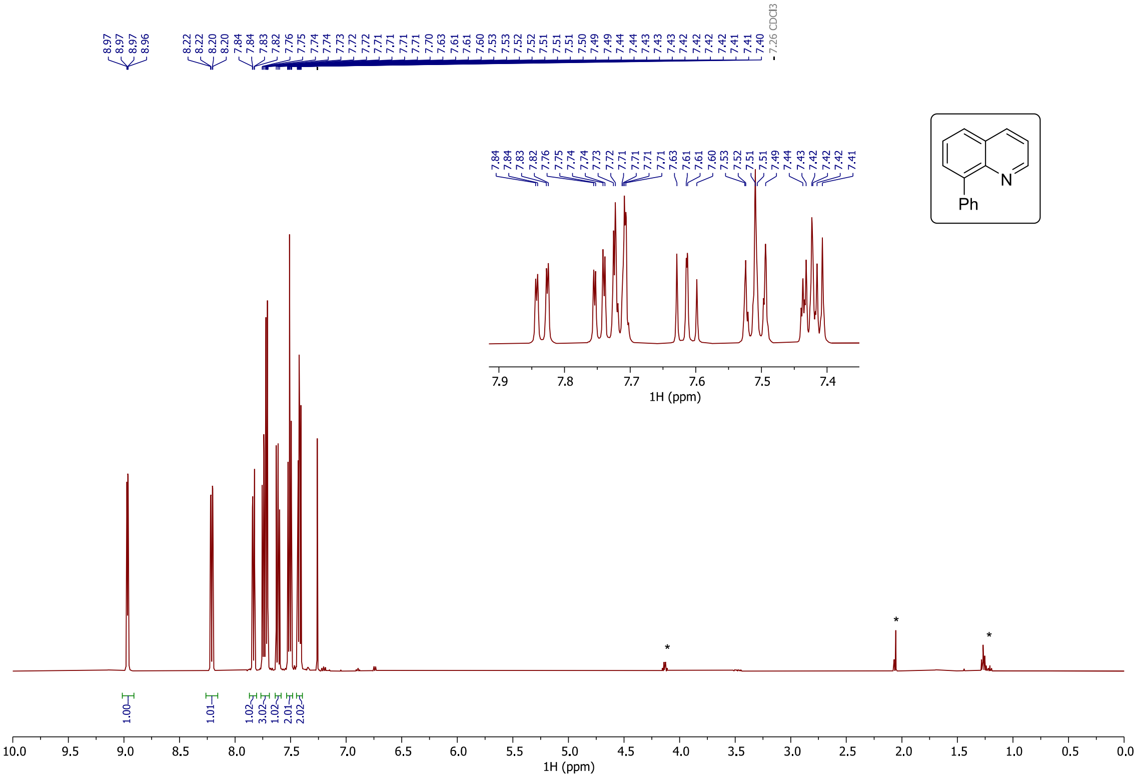

Figure S106: ^{1}H NMR (500 MHz, $CDCl_3$, 298K) of **S10**, residual EtOAc is marked with *.

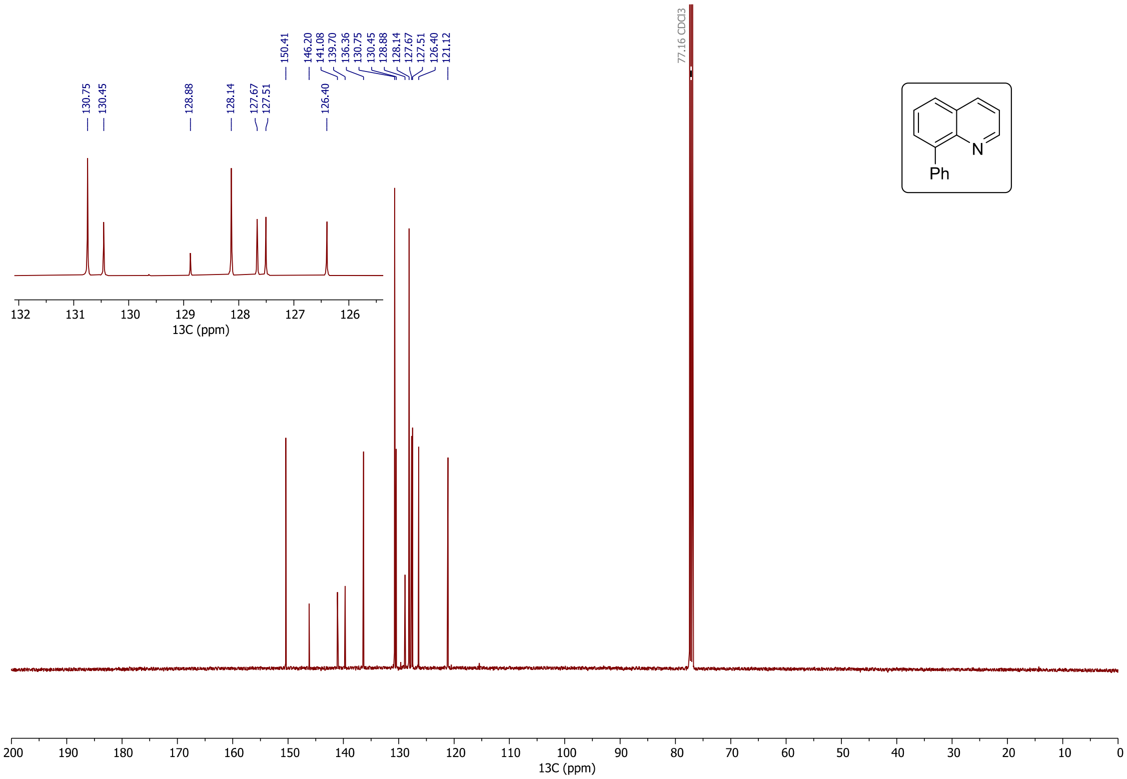

Figure S107: **^{13}C {^{1}H} NMR** (126 MHz, $CDCl_3$, 298K) of **S10**..

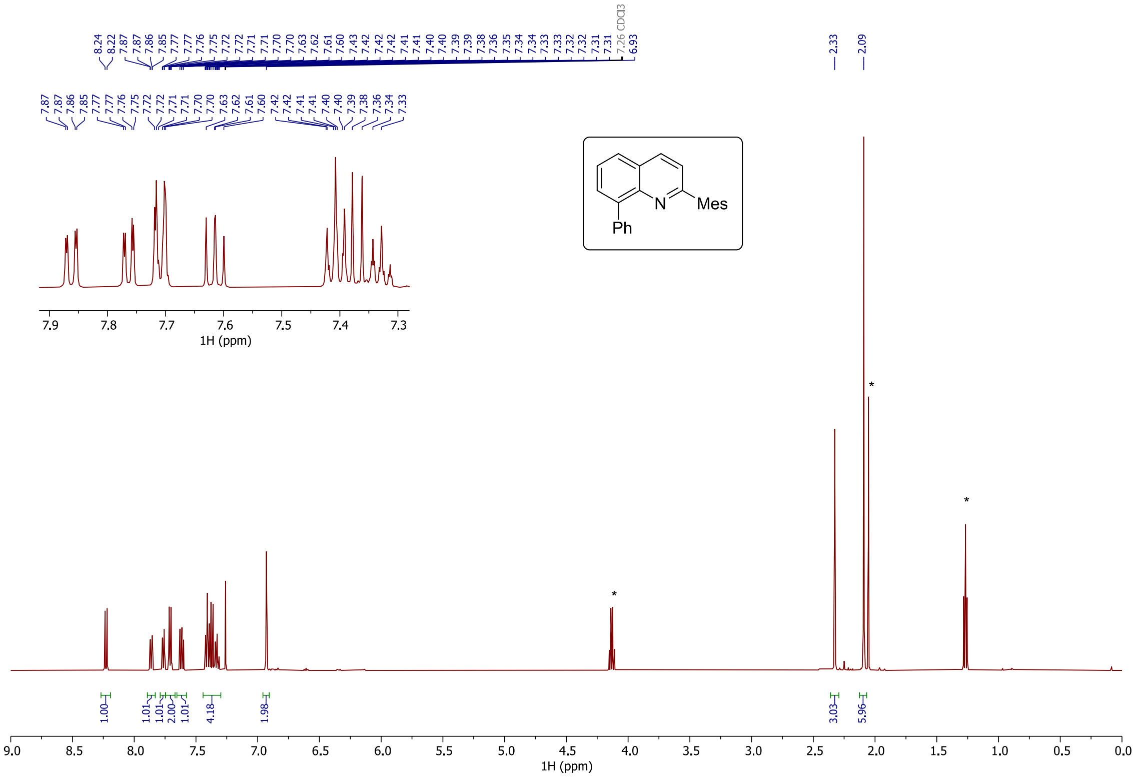

Figure S108: **^{1}H NMR** (500 MHz, $CDCl_3$, 298K) of **S11**, residual EtOAc is marked with *.

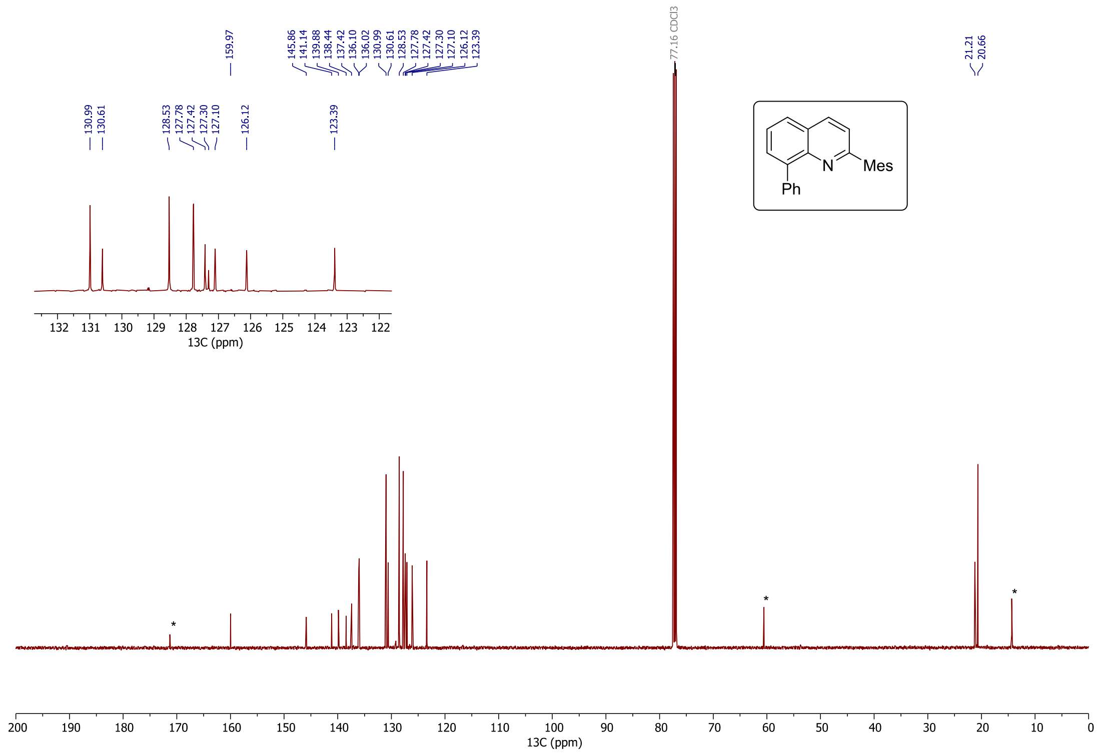

Figure S109: $^{13}C\ \{^{1}H\}$ NMR (126 MHz, $CDCl_3$, 298K) of **S11**, residual EtOAc is marked with *.

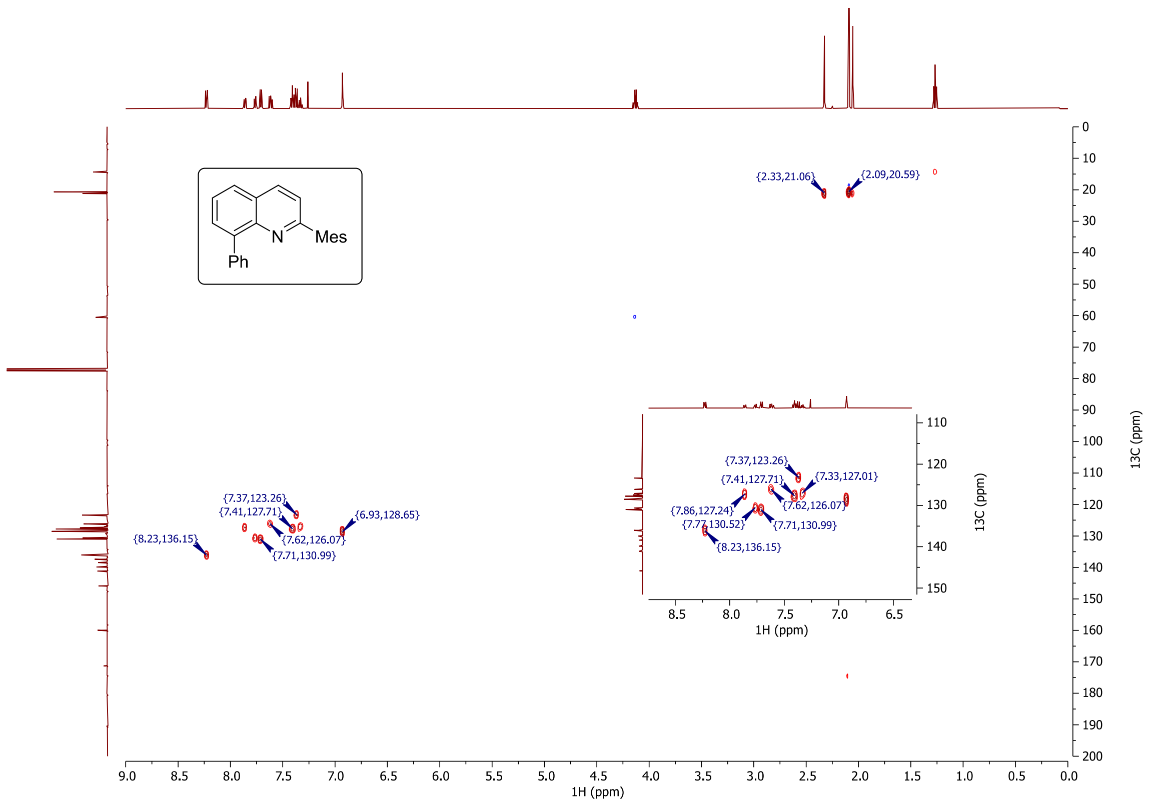

Figure S110: $^{1}H/^{13}C$ HSQC (500/126 MHz, $CDCl_3$, 298K) of **S11**.

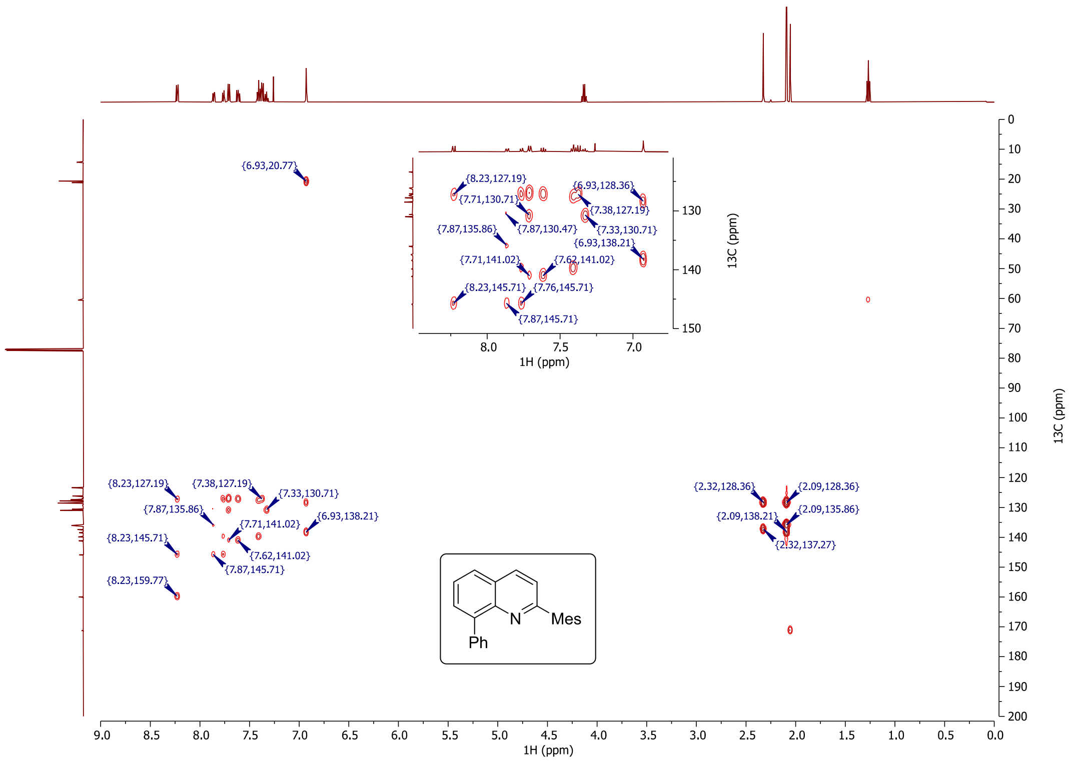

Figure S111: ^{1}H/^{13}C HMBC (500/126 MHz, $CDCl_3$, 298K) of **S11**.

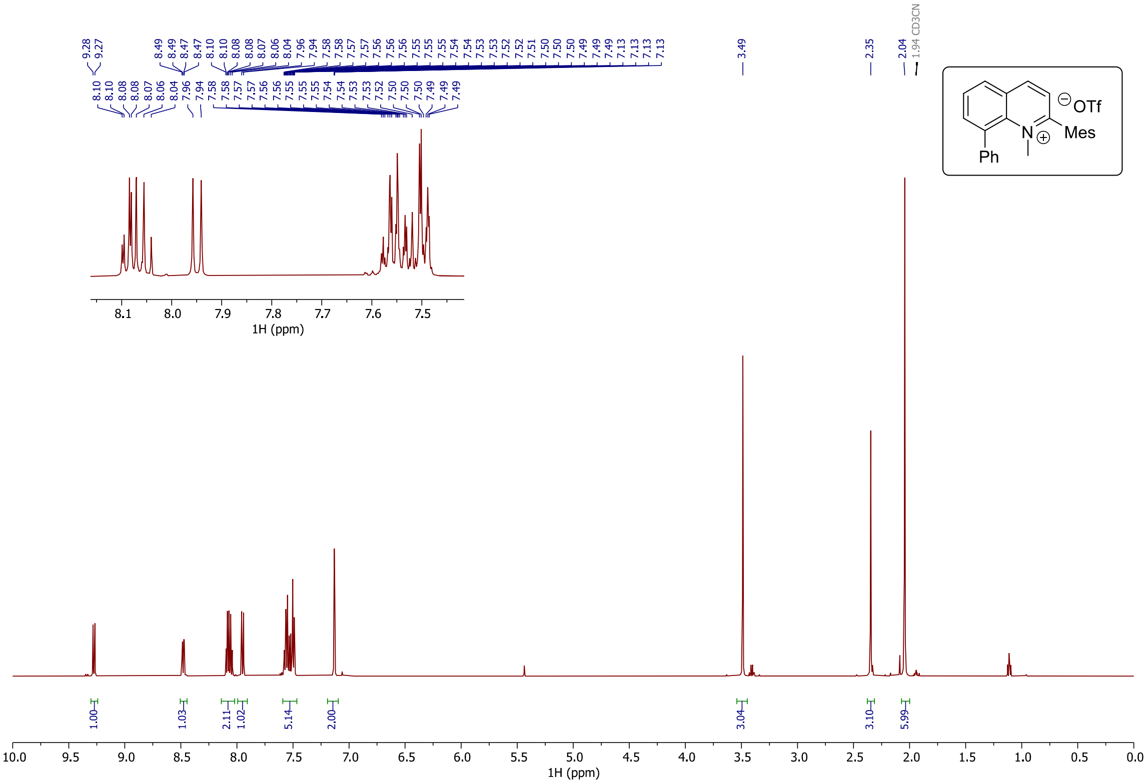

Figure S112: ^{1}H NMR (500 MHz, CD_3CN, 298K) of **1i**.

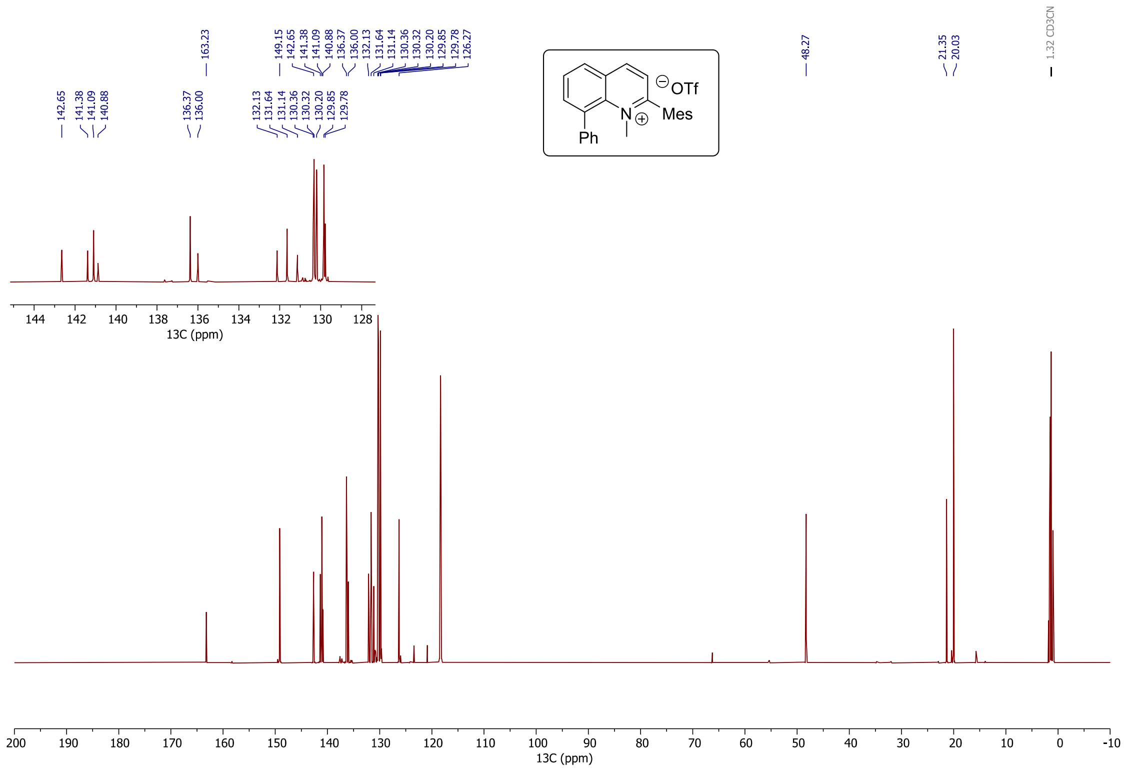

Figure S113: ^{13}C {^{1}H} NMR (126 MHz, CD_3CN, 298K) of **1i**.

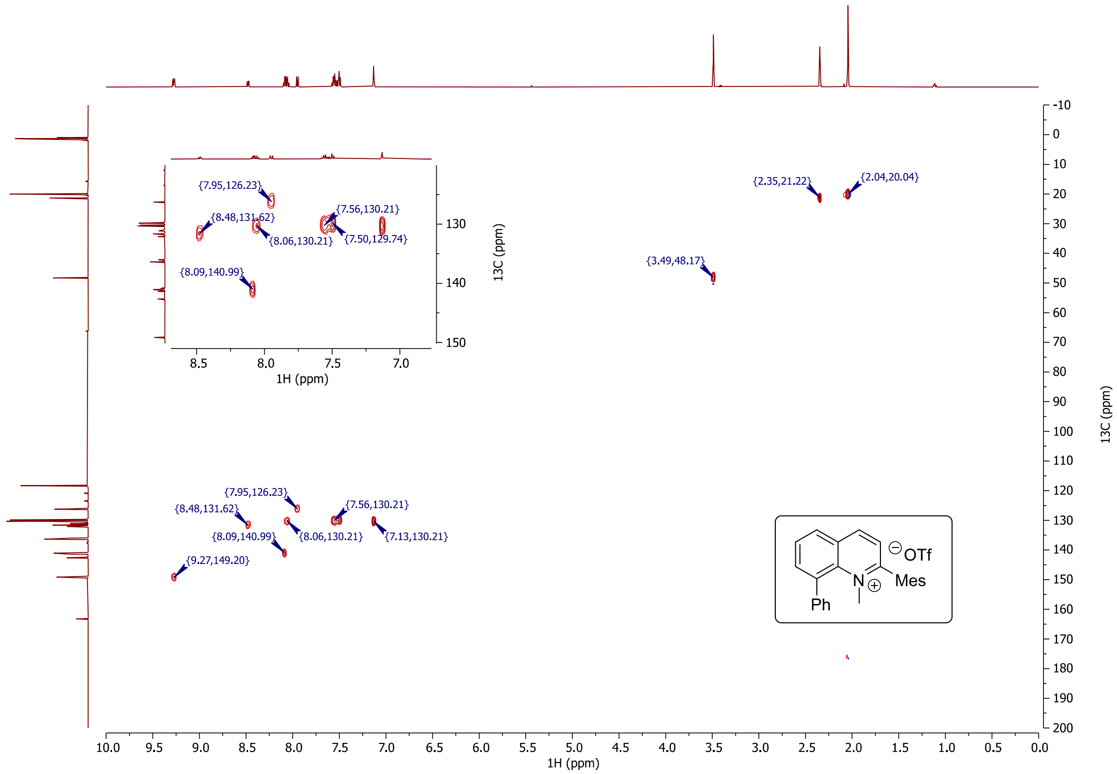

Figure S114: $^{1}H/^{13}C$ HSQC (500/126 MHz, CD_3CN, 298K) of **1i**.

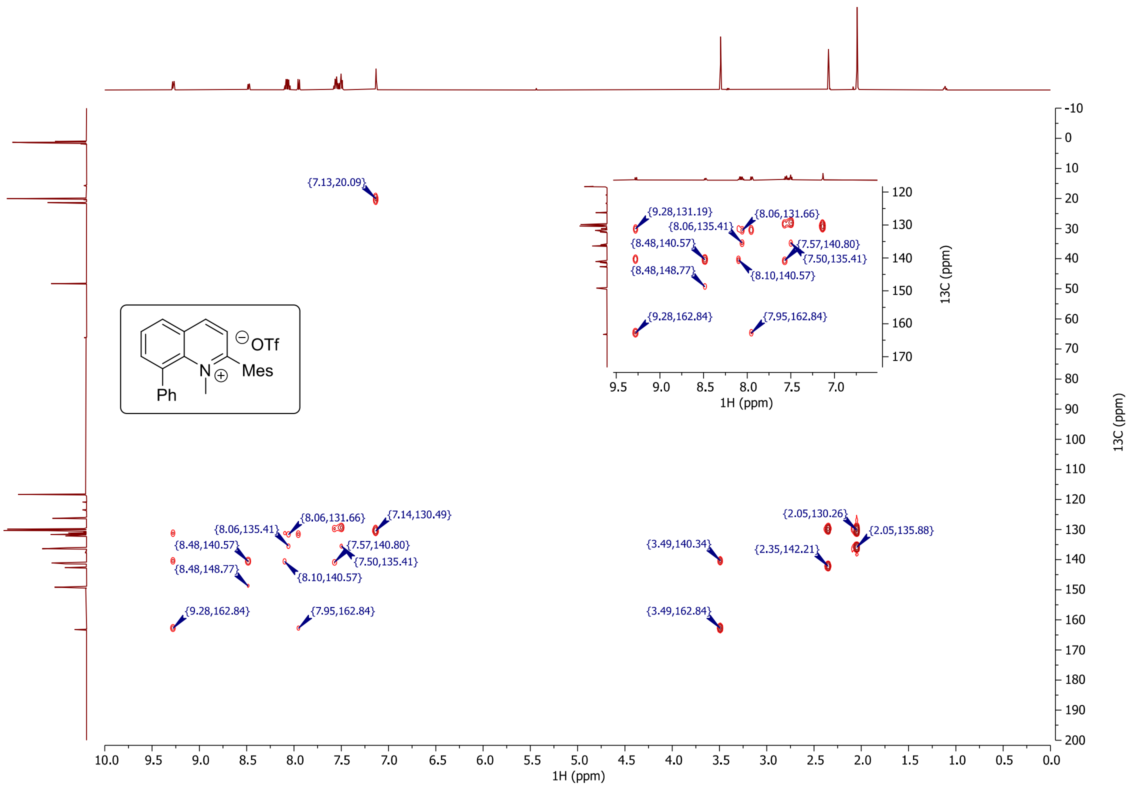

Figure S115: ^{1}H/^{13}C HMBC (500/126 MHz, CD_3CN, 298K) of **1i**.

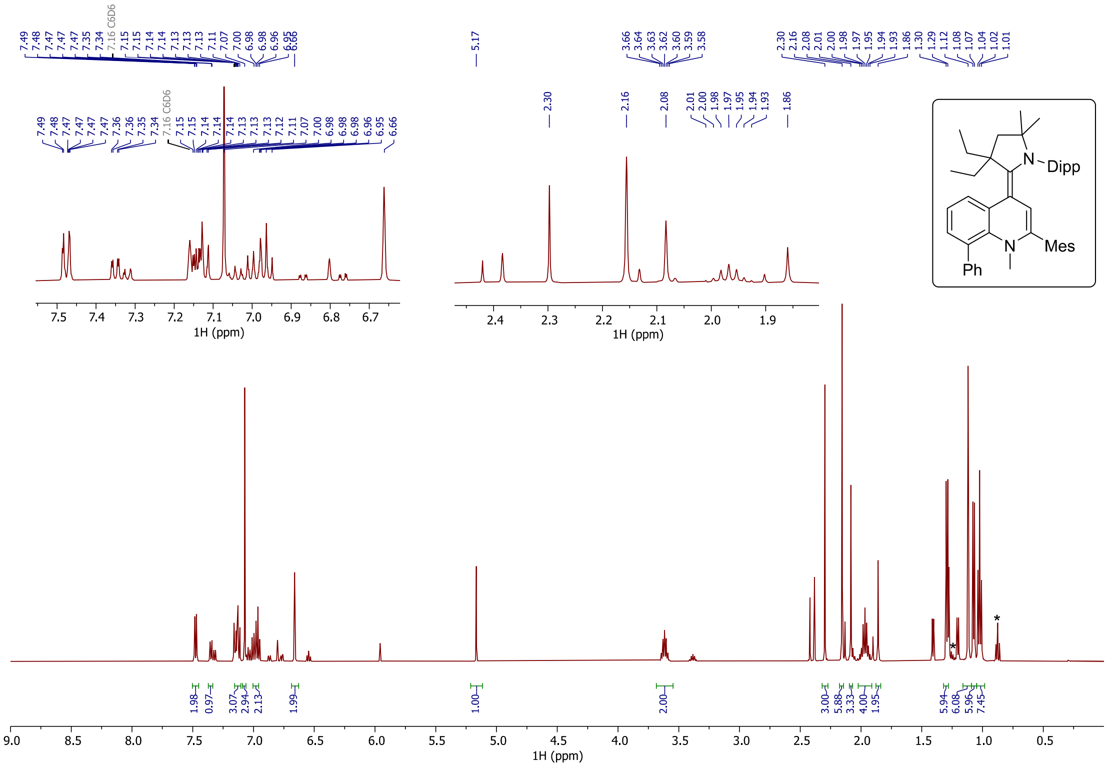

Figure S116: ^{1}H NMR (500 MHz, C_6D_6, 298K) of **2i**. *E*:*Z* after the reaction = 76:24

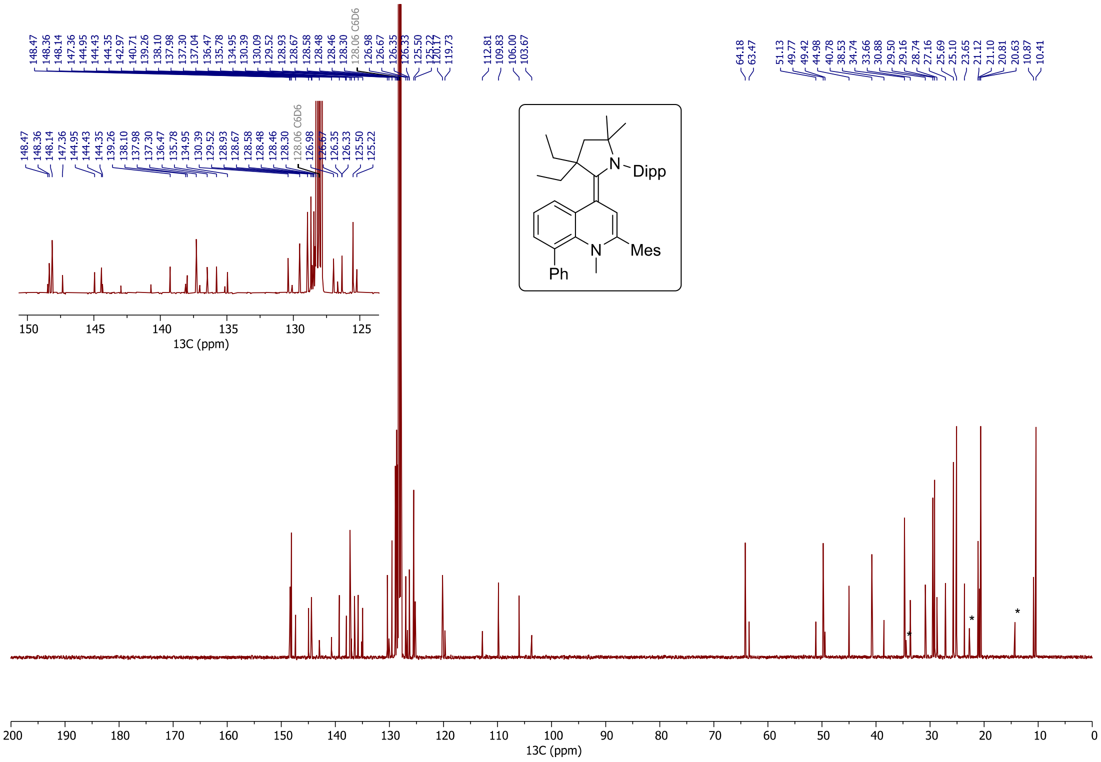

Figure S117: **^{13}C {^{1}H} NMR** (126 MHz, C_6D_6, 298K) of **2i**.

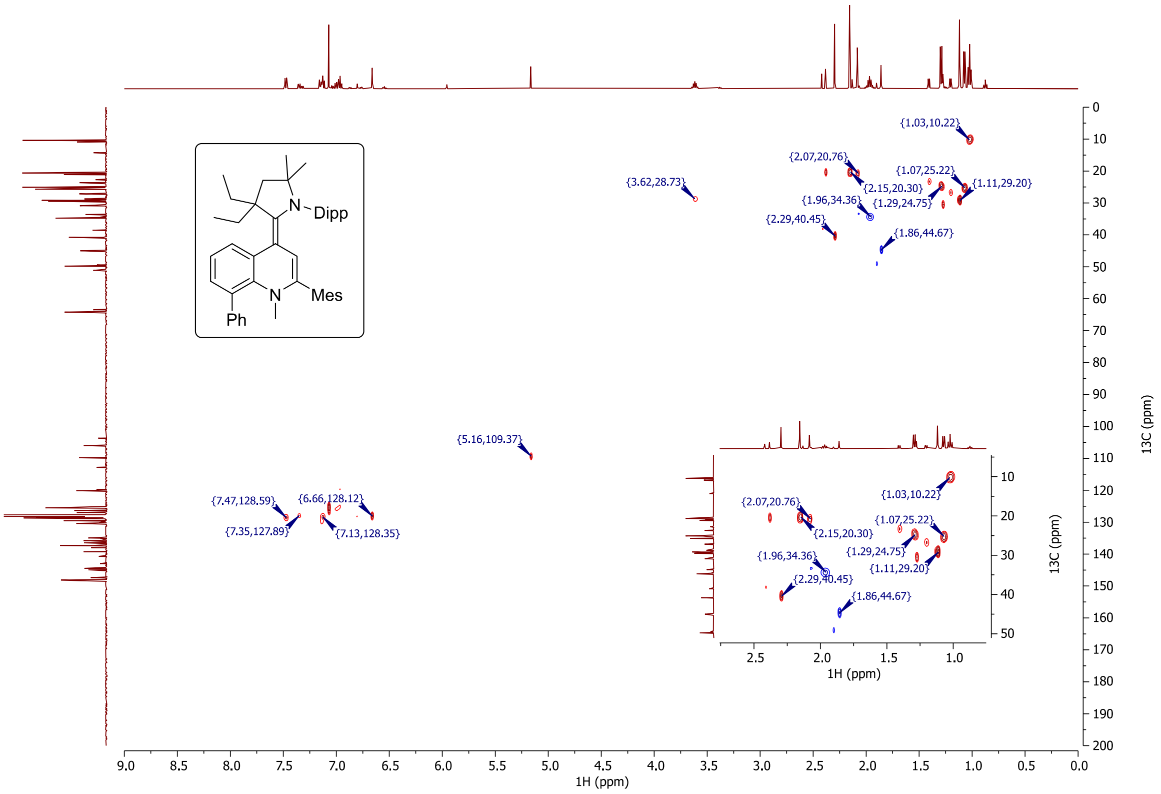

Figure S118: **^{1}H/^{13}C HSQC** (500/126 MHz, C_6D_6, 298K) of **2i**.

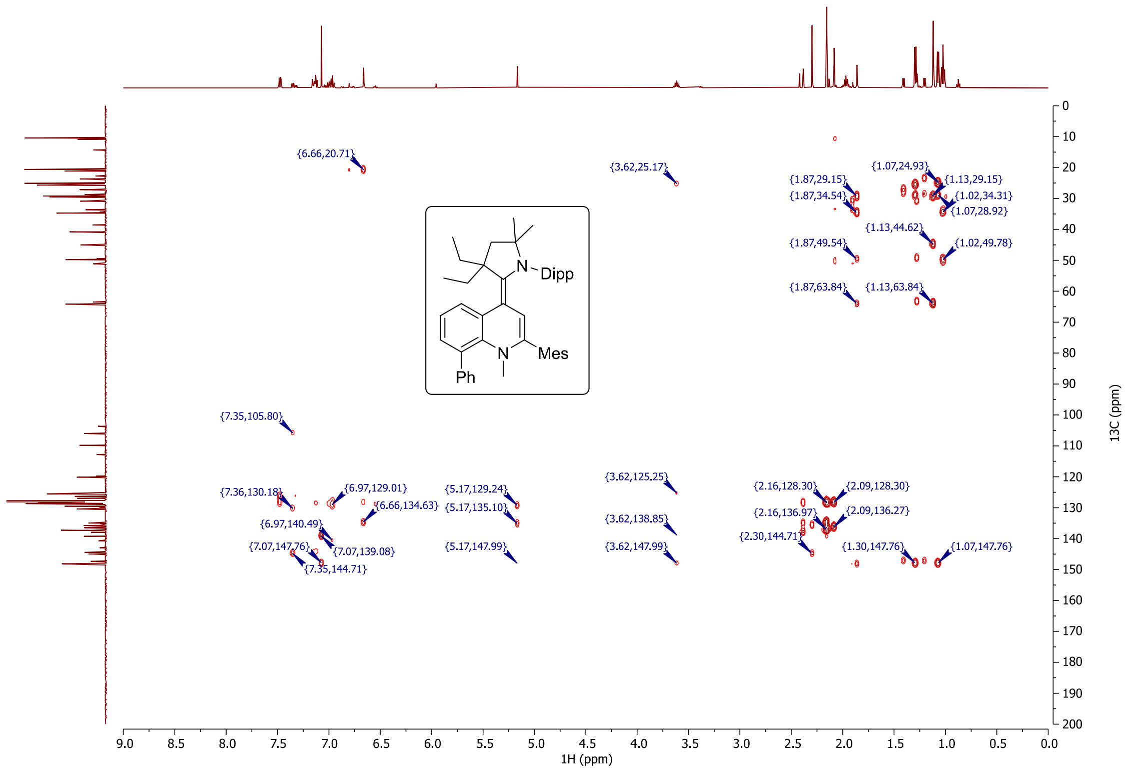

Figure S119: $^{1}H/^{13}C$ HMBC (500/126 MHz, C_6D_6, 298K) of **2i**.

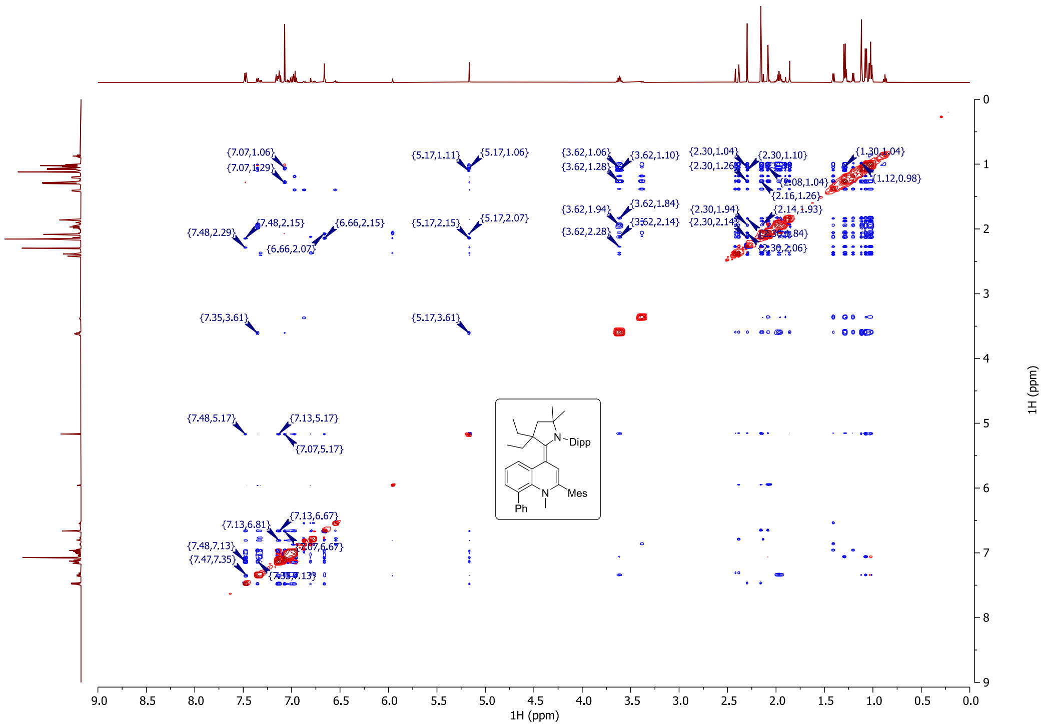

Figure S120: NOESY-NMR (500 MHz, C_6D_6, 298K) of **2i**.

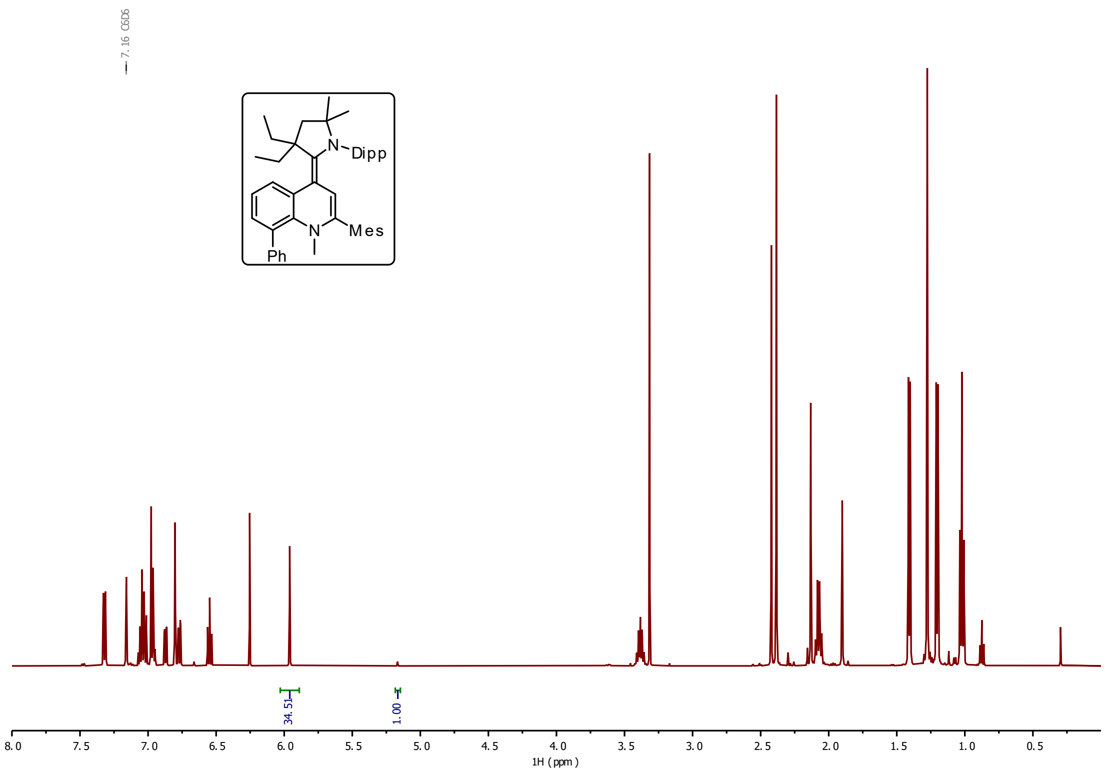

Figure S121: ^{1}**H NMR** (500 MHz, C_6D_6, 298 K) of **2i** after irradiation λ = 467 nm, 1h; *E*:*Z* = 1:98. Contains Trimethoxy-benzene as internal standard. Indicative signals are integrated to represent the isomers.

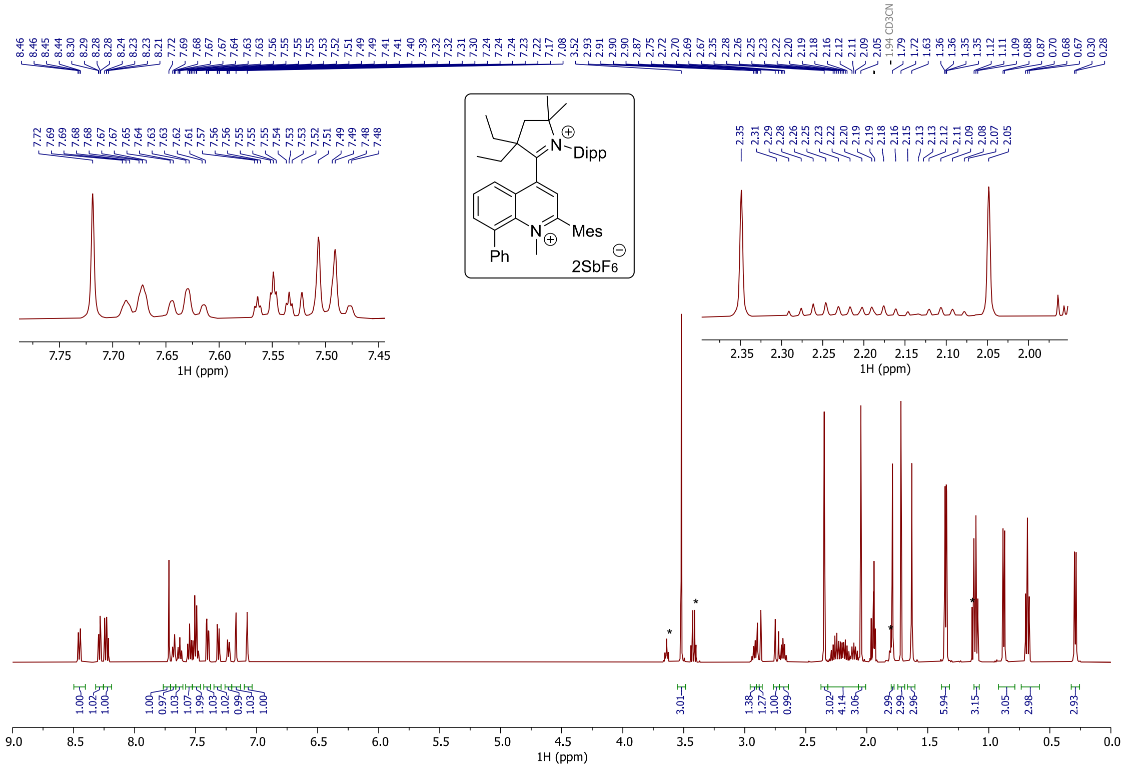

Figure S122: ^{1}**H NMR** (500 MHz, CD_3CN, 298K) of **4i**, residual thf, Et_2O is marked with a *.

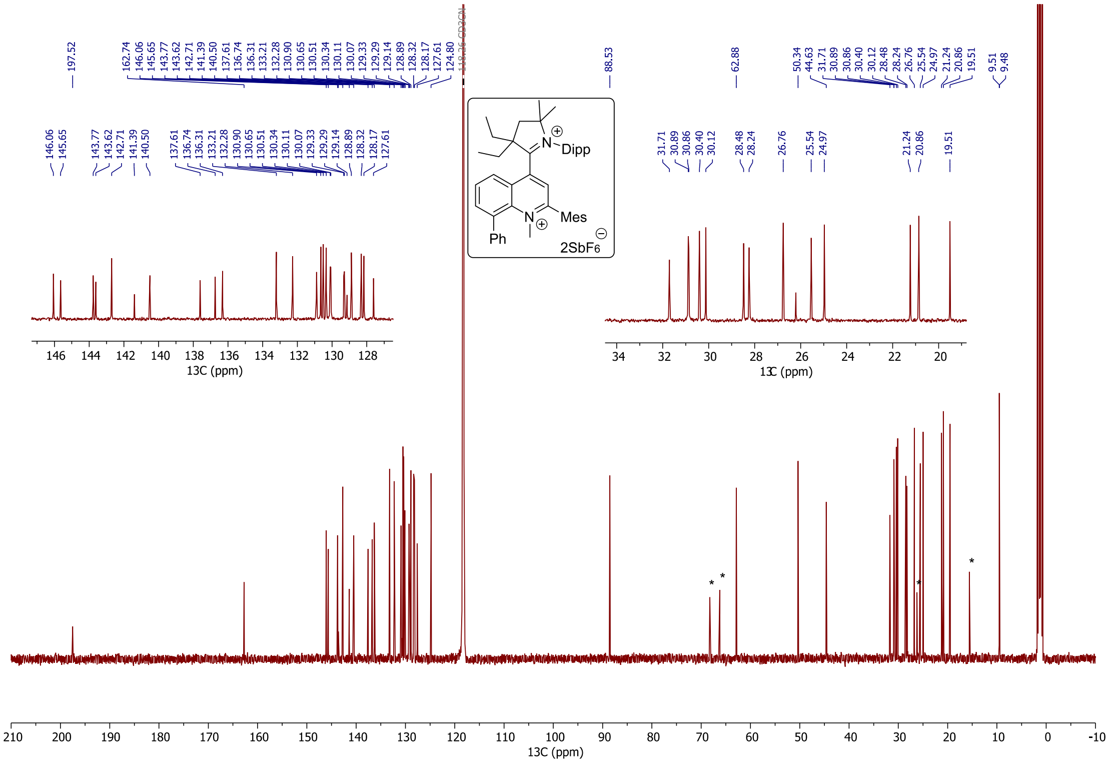

Figure S123: ^{13}C {^{1}H} NMR (126 MHz, CD_3CN, 298K) of **4i**, residual thf, Et_2O is marked with a *.

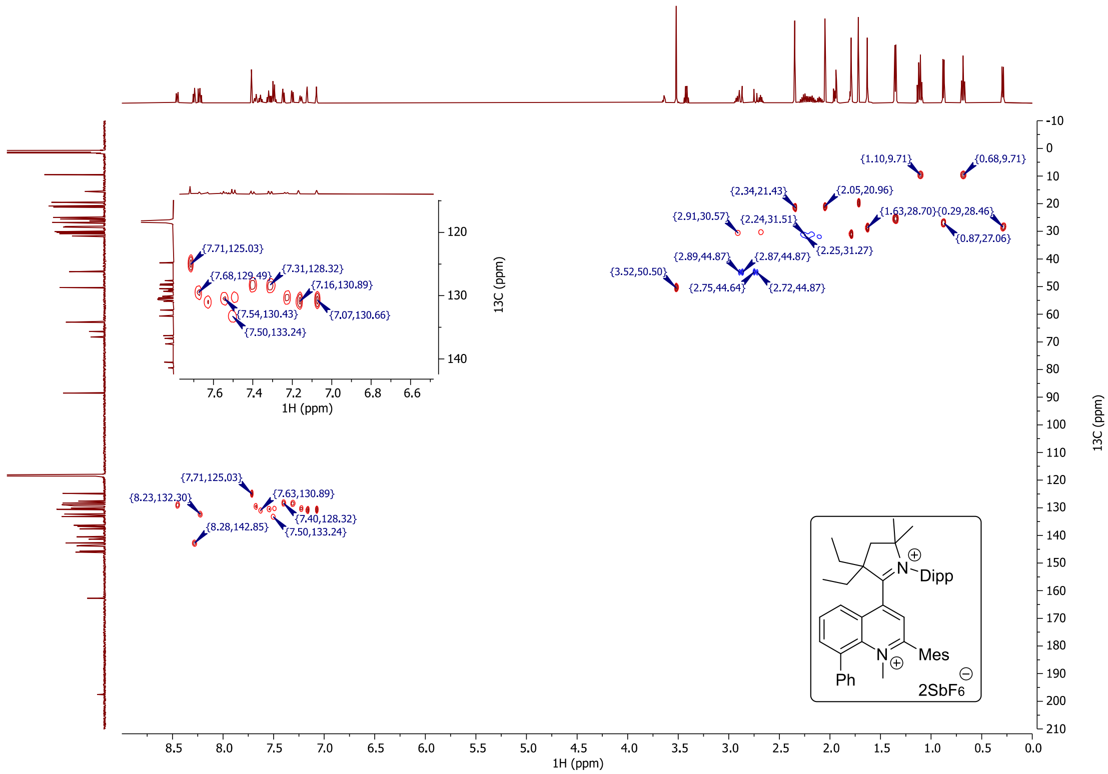

Figure S124: ^{1}H/^{13}C HSQC (500/126 MHz, CD_3CN, 298K) of **4i**.

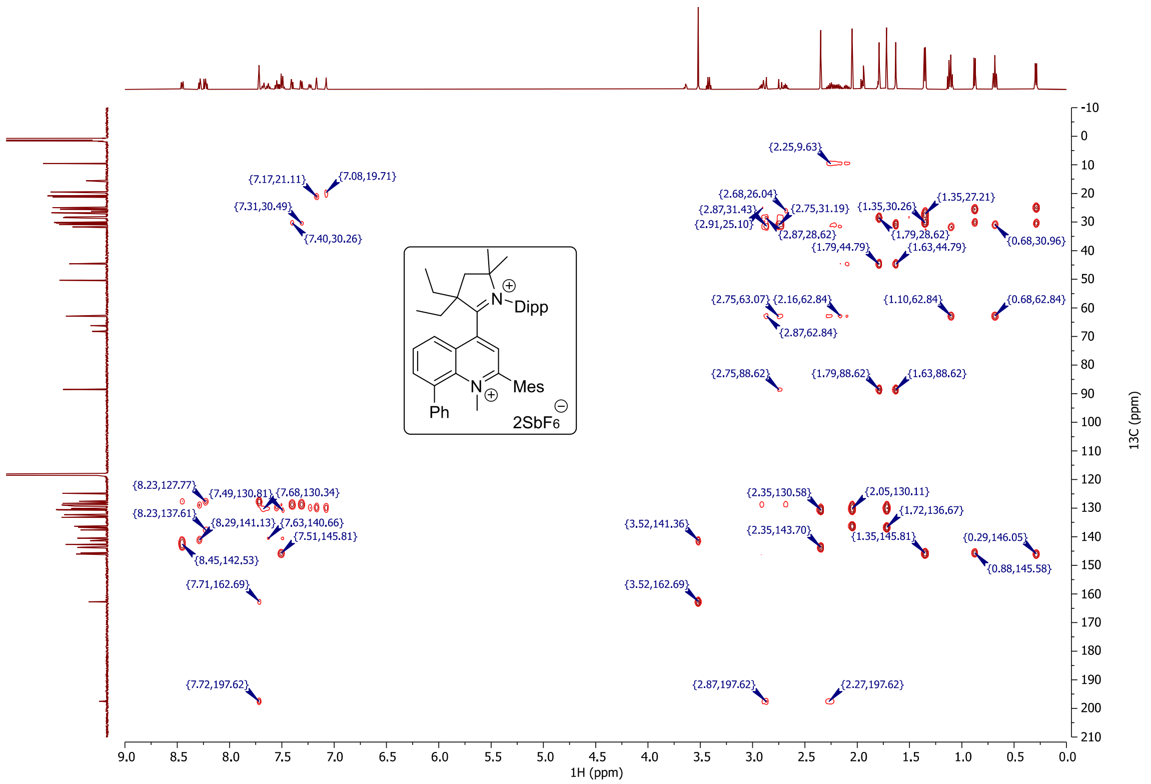

Figure S125: ^{1}H/^{13}C HMBC (500/126 MHz, CD_3CN, 298K) of **4i**.

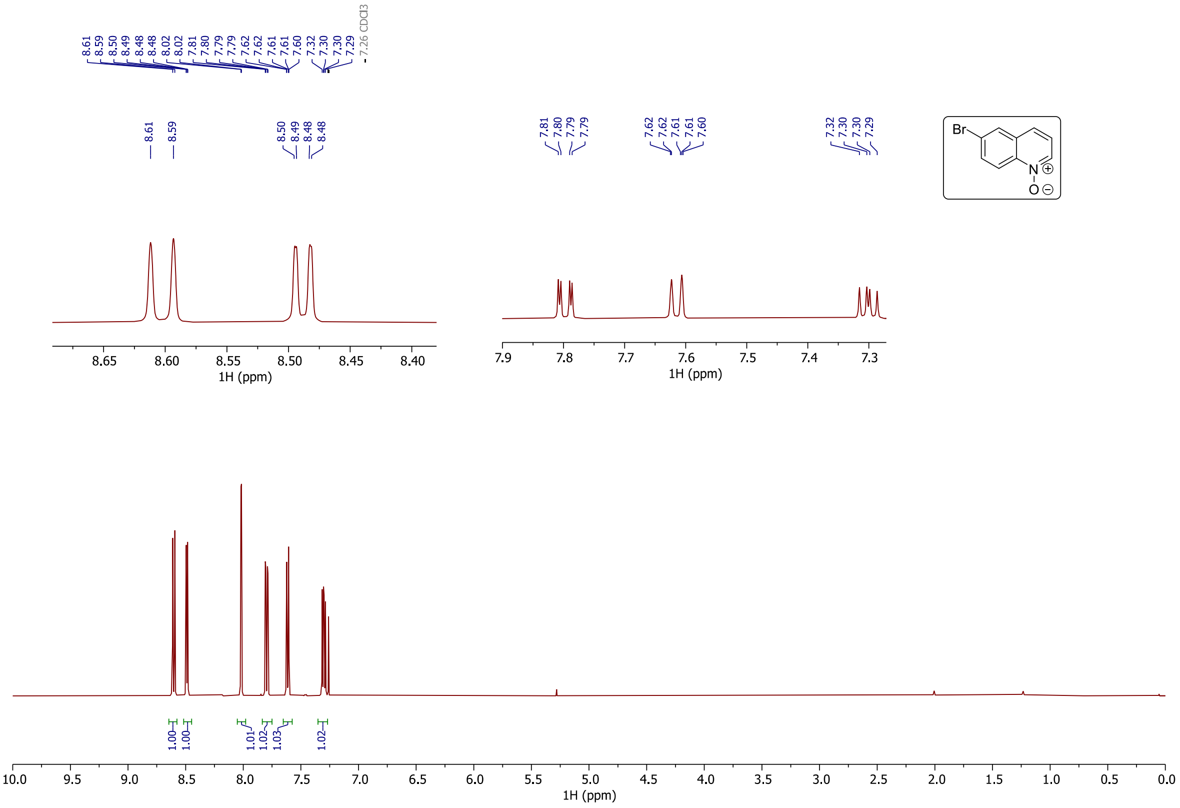

Figure S126: ^{1}H NMR (500 MHz, $CDCl_3$, 298K) of **S12**.

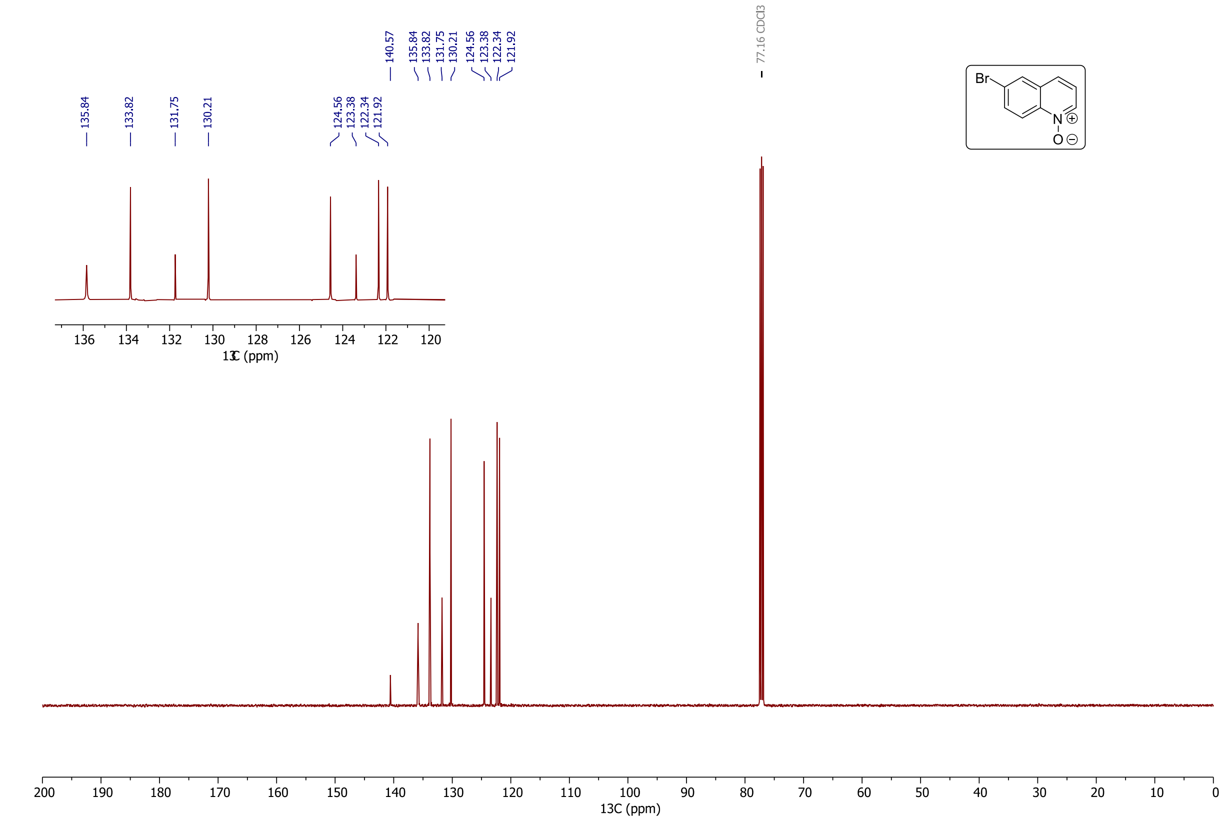

Figure S127: ^{13}**C {**^{1}**H} NMR** (126 MHz, $CDCl_3$, 298K) of **S12**.

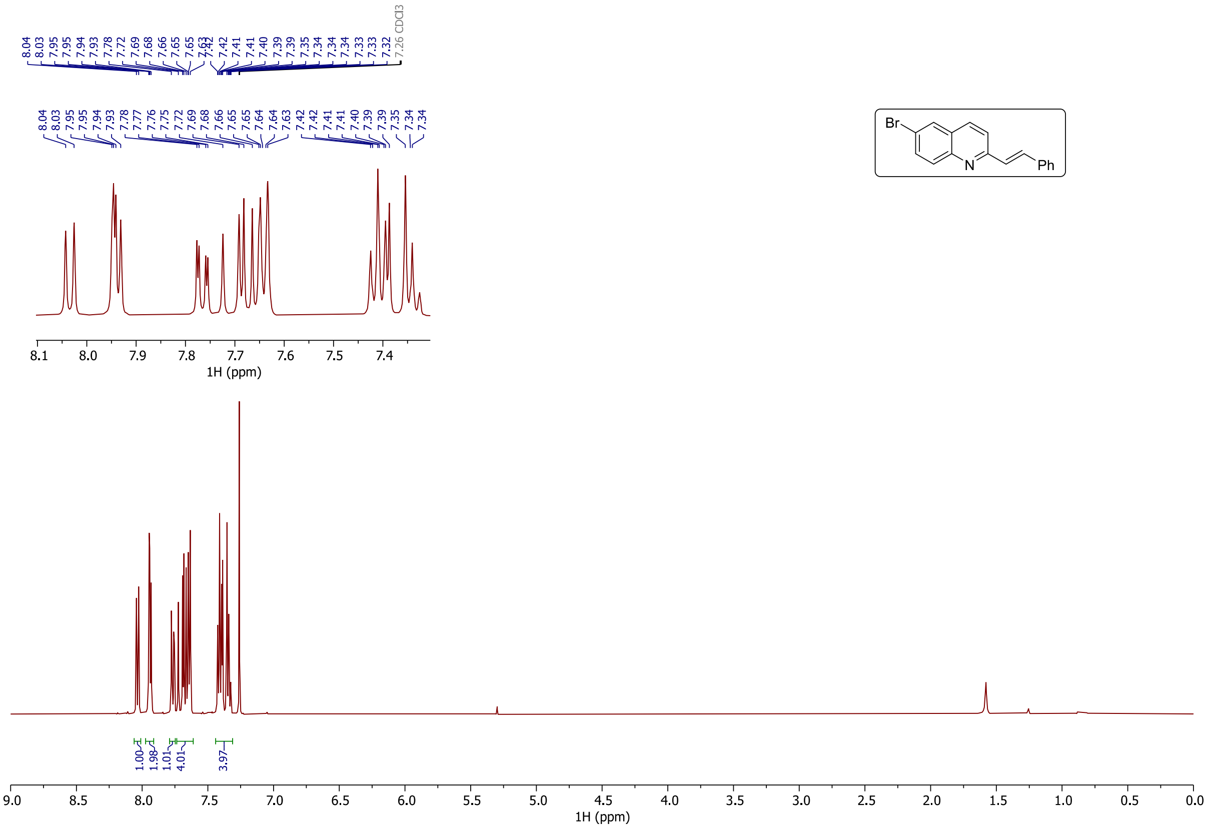

Figure S128: ^{1}**H NMR** (500 MHz, $CDCl_3$, 298K) of **S13**.

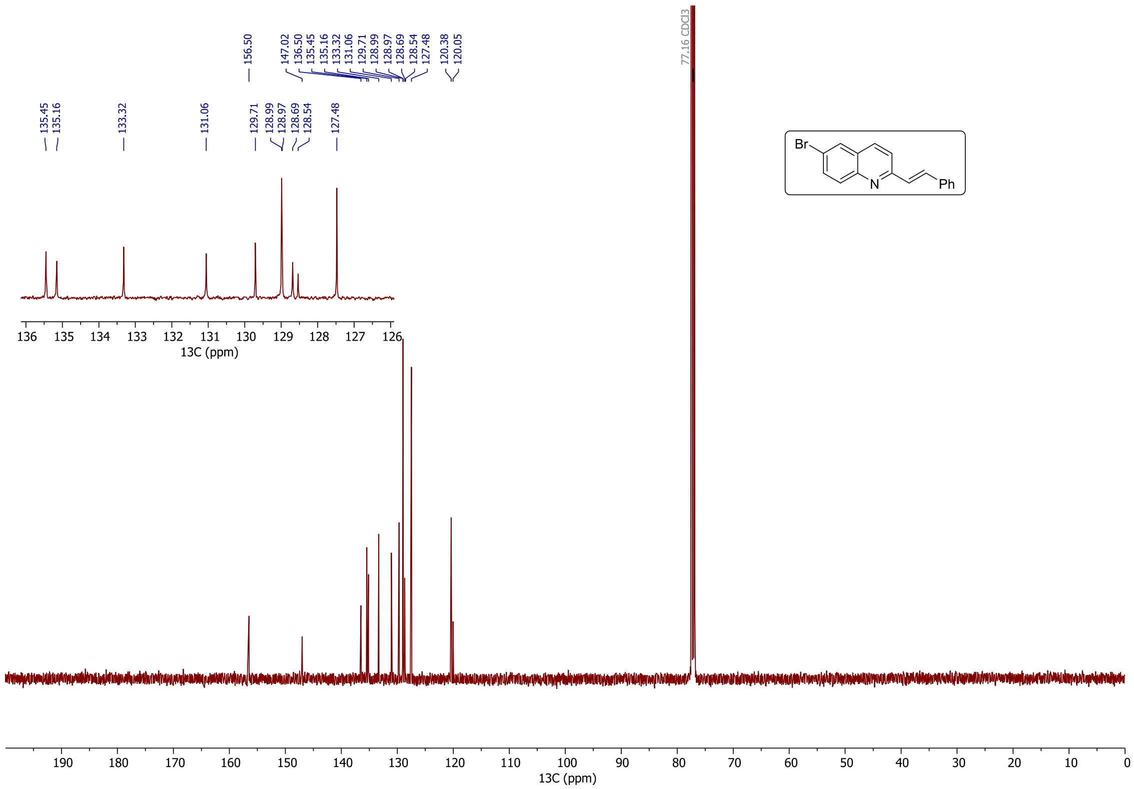

Figure S129: **^{13}C {^{1}H} NMR** (126 MHz, $CDCl_3$, 298K) of **S13**.

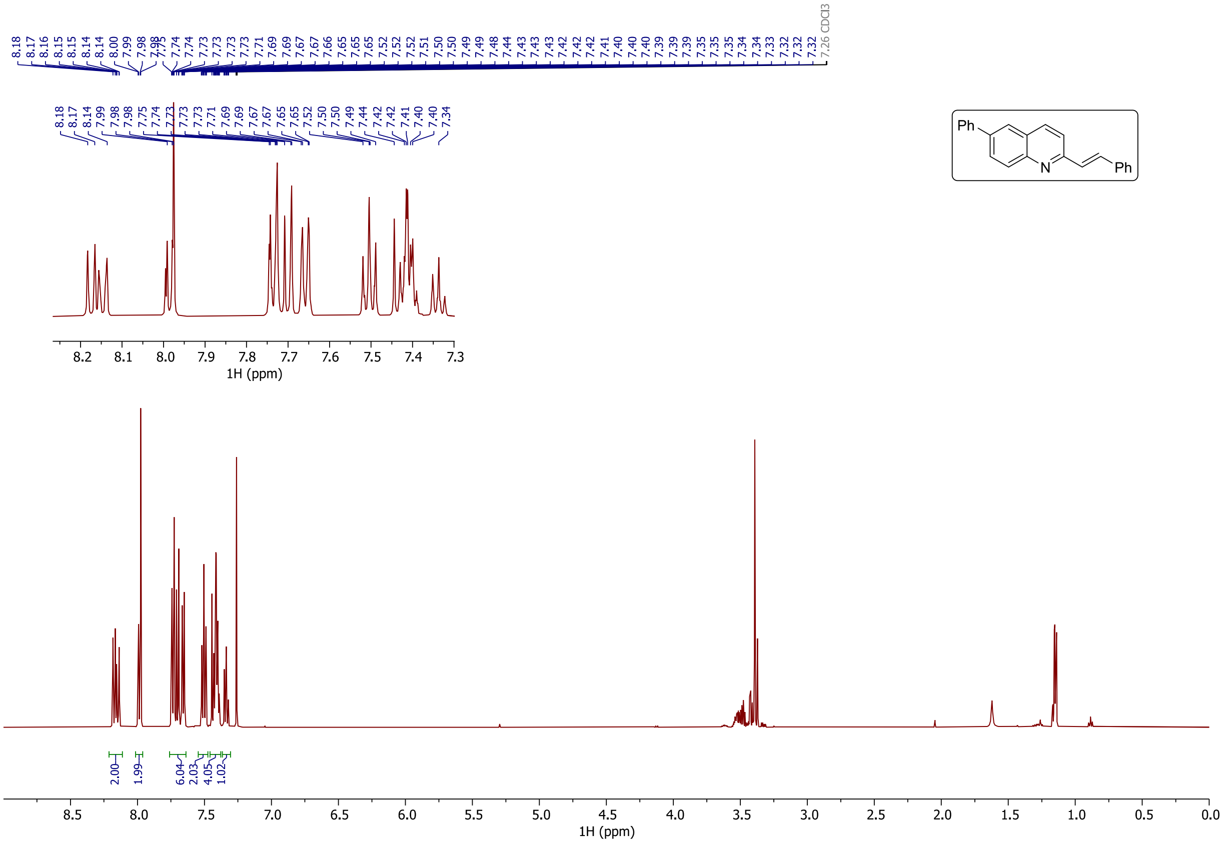

Figure S130: ^{1}H NMR (500 MHz, $CDCl_3$, 298K) of **S14**.

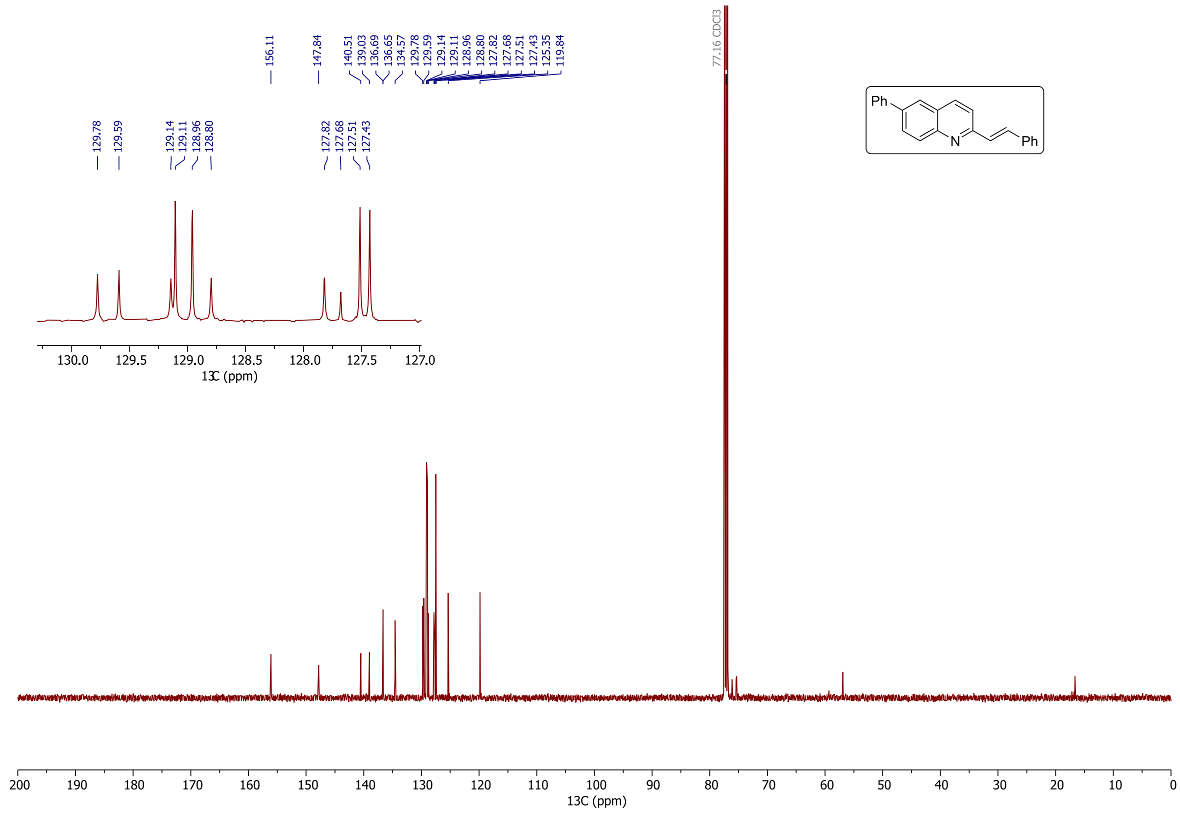

Figure S131: ^{13}**C {**^{1}**H} NMR** (126 MHz, $CDCl_3$, 298K) of **S14**.

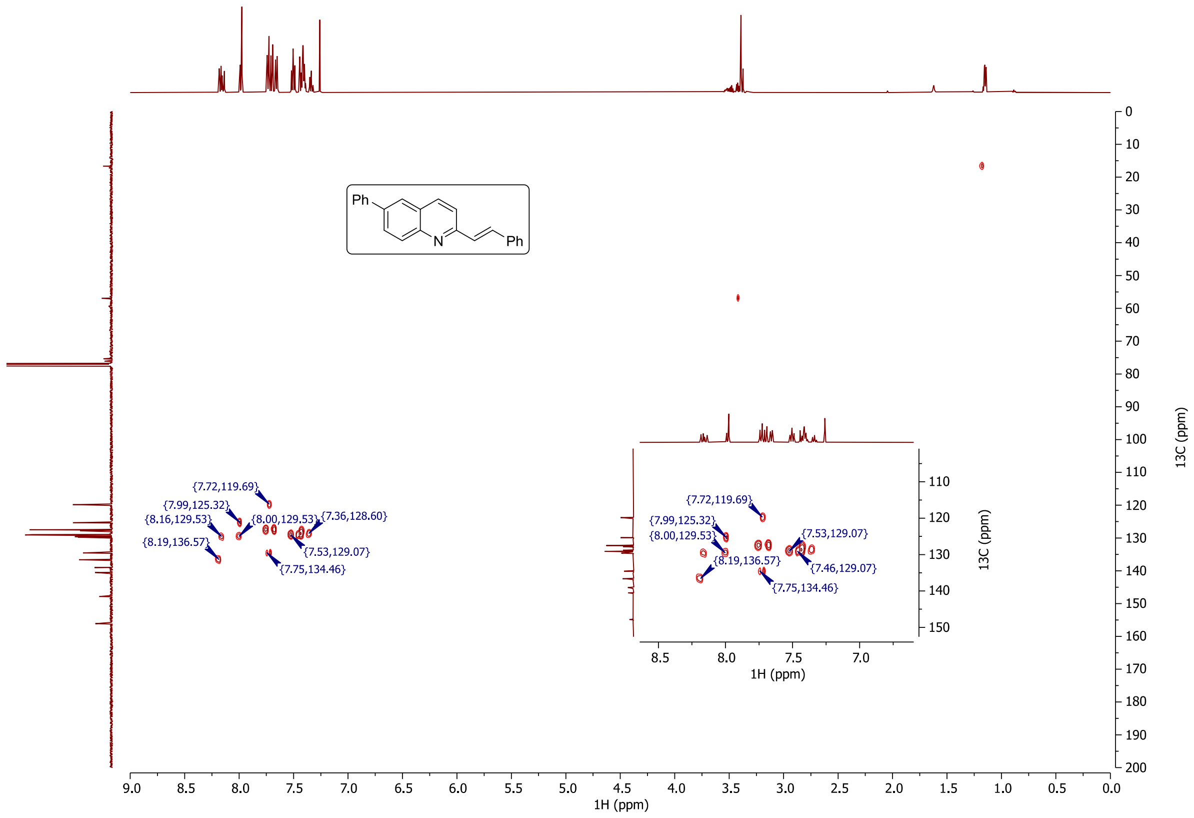

Figure S132: ^{1}**H/**^{13}**C HSQC** (500/126 MHz, $CDCl_3$, 298K) of **S14**.

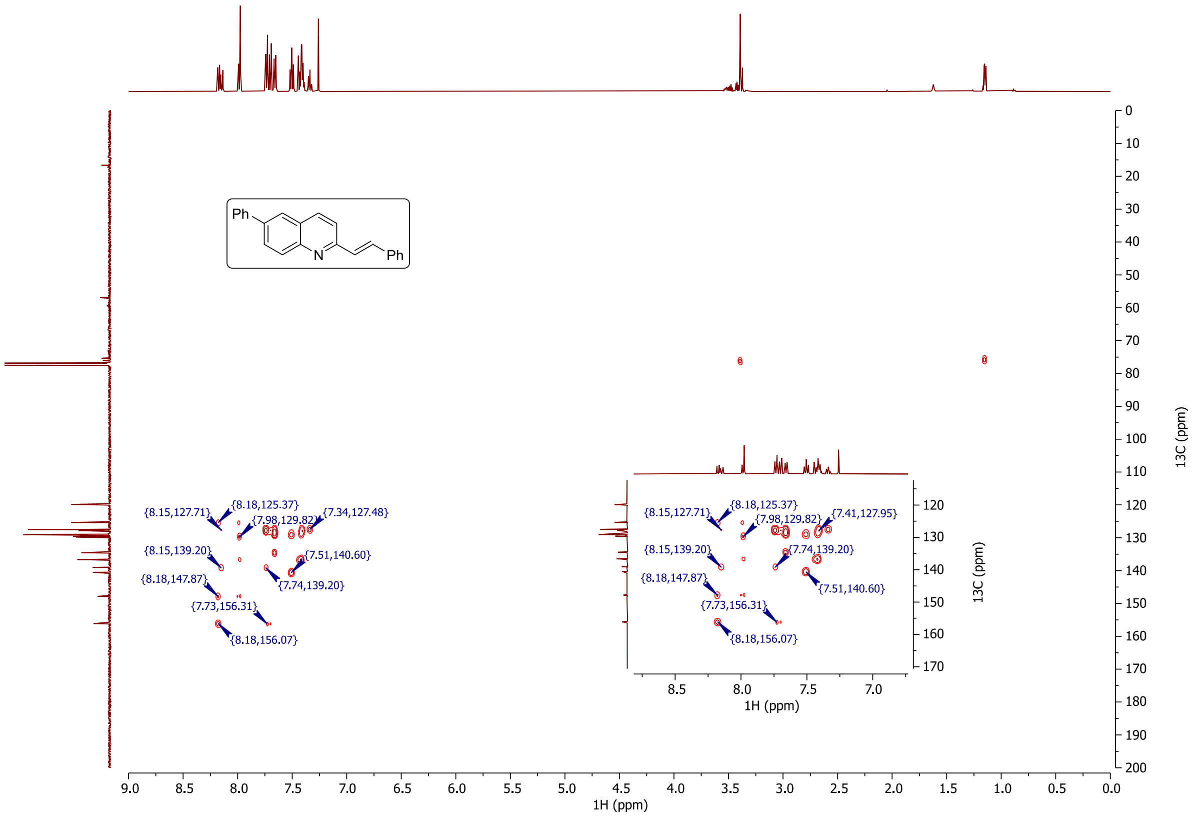

Figure S133: ^{1}H/^{13}C HMBC (500/126 MHz, $CDCl_3$, 298K) of **S14**.

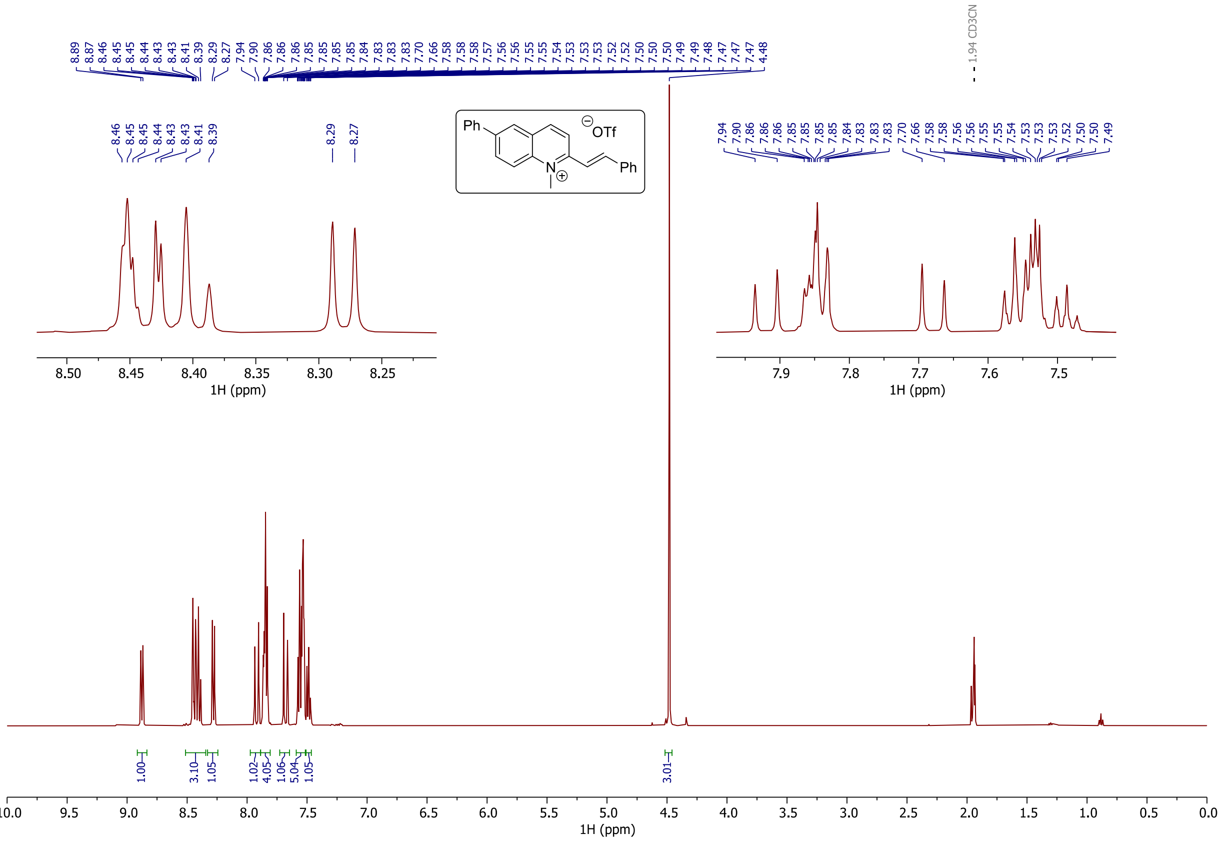

Figure S134: ^{1}H NMR (500 MHz, CD_3CN, 298K) of **1j**.

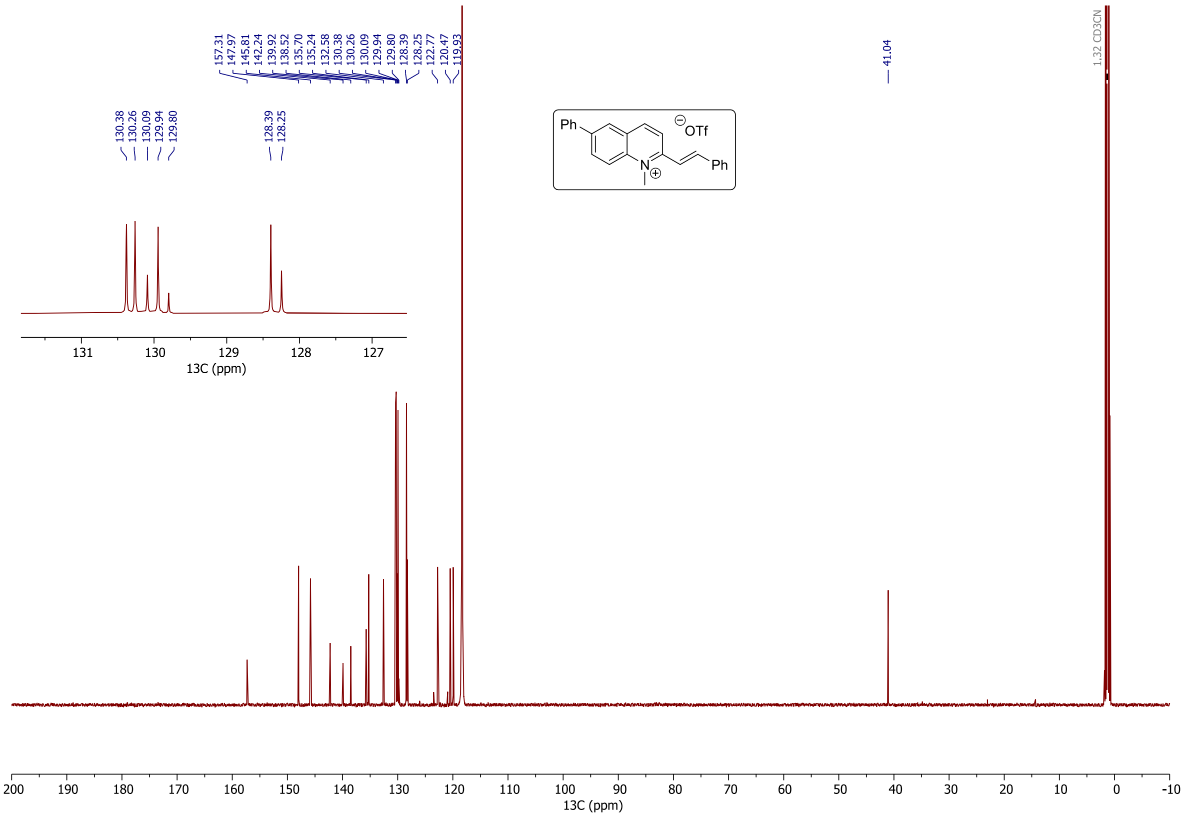

Figure S135: $^{13}C\ \{^{1}H\}$ NMR (126 MHz, CD_3CN, 298K) of **1j**.

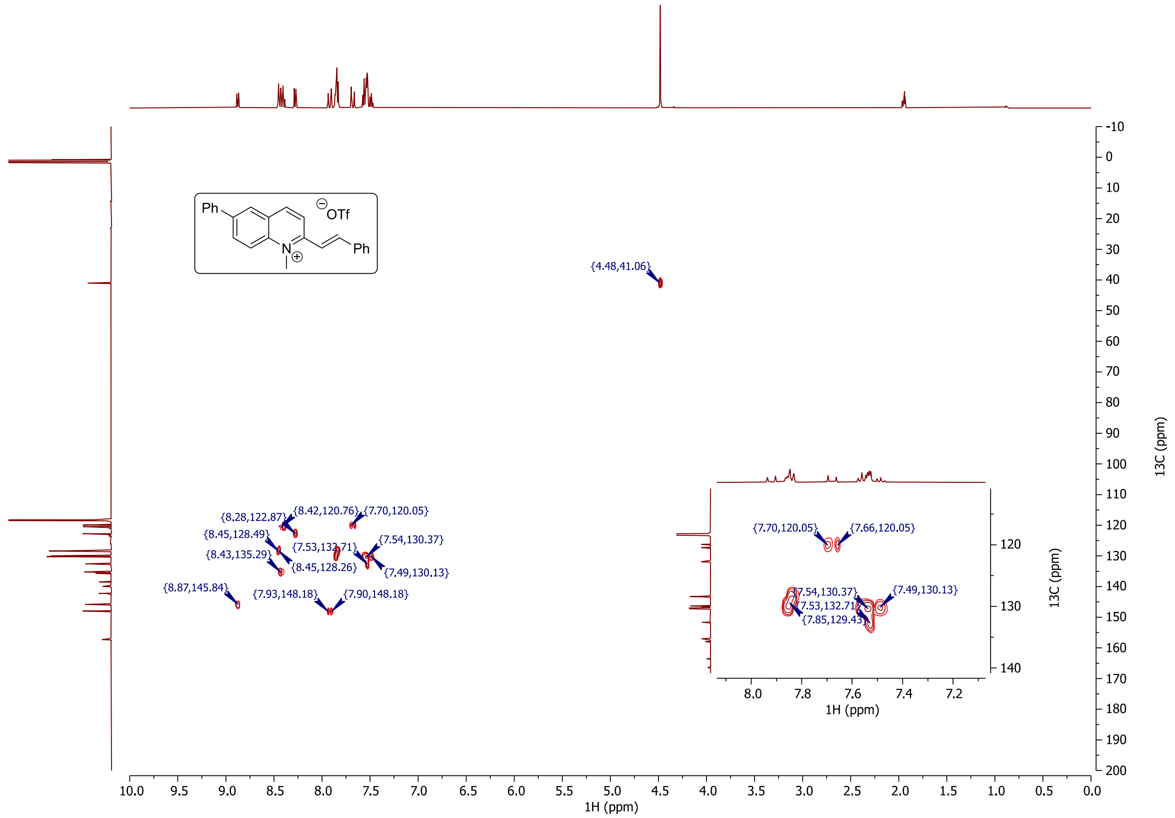

Figure S136: $^{1}H/^{13}C$ HSQC (500/126 MHz, CD_3CN, 298K) of **1j**.

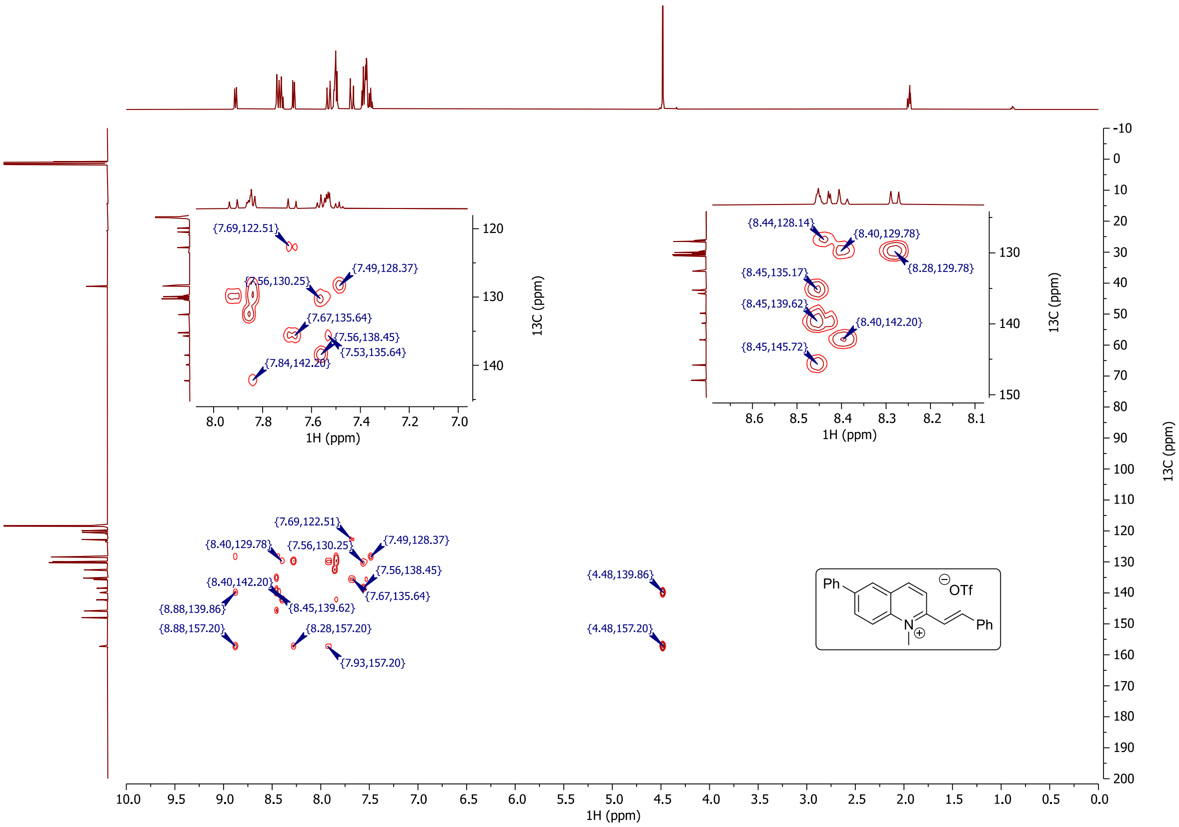

Figure S137: $^{1}H/^{13}C$ **HMBC** (500/126 MHz, CD_3CN, 298K) of **1j**.

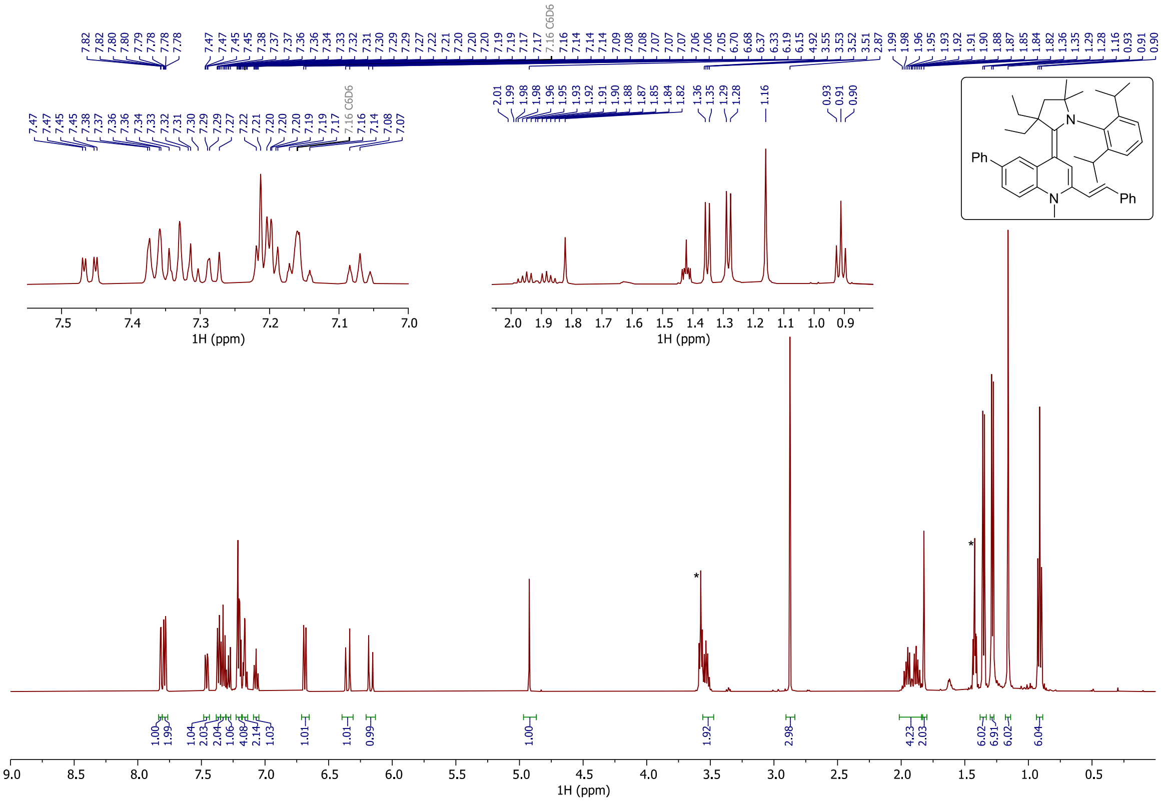

Figure S138: ^{1}**H NMR** (500 MHz, C_6D_6, 298K) of **2j**, residual thf is marked with *.

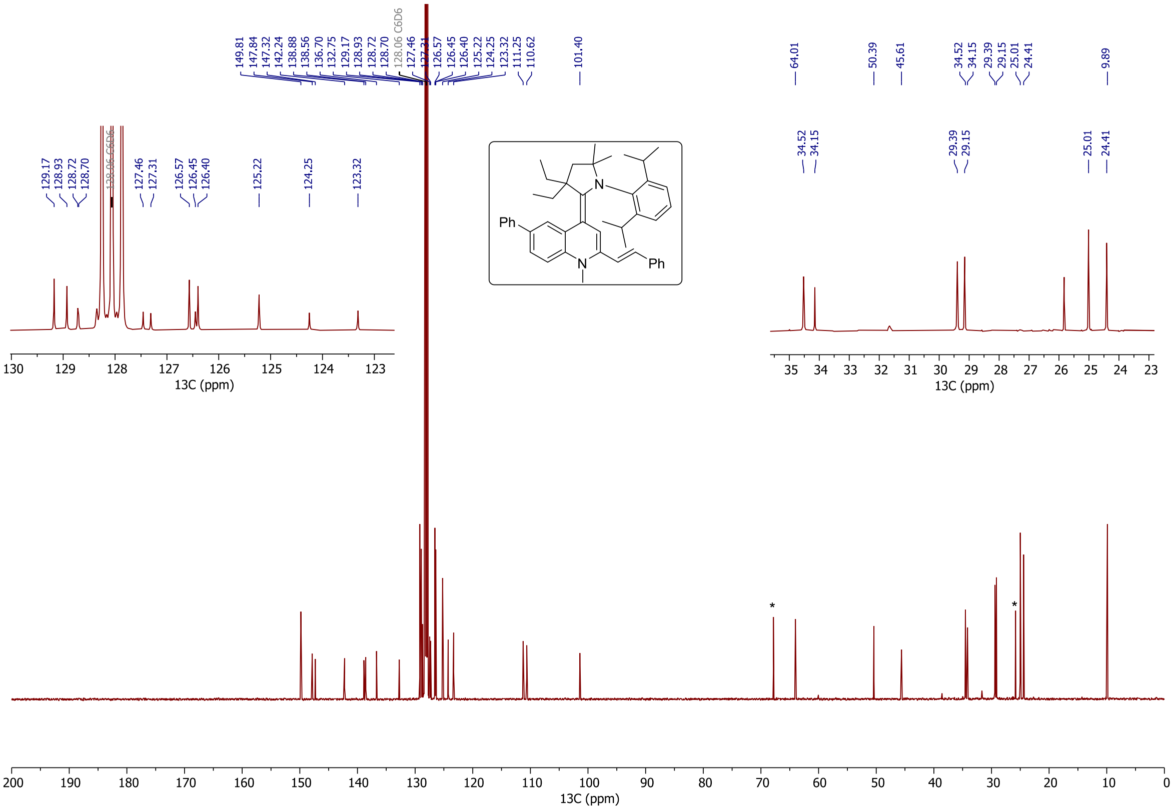

Figure S139: ^{13}**C {**^{1}**H} NMR** (126 MHz, C_6D_6, 298K) of **2j**, residual thf is marked with *.

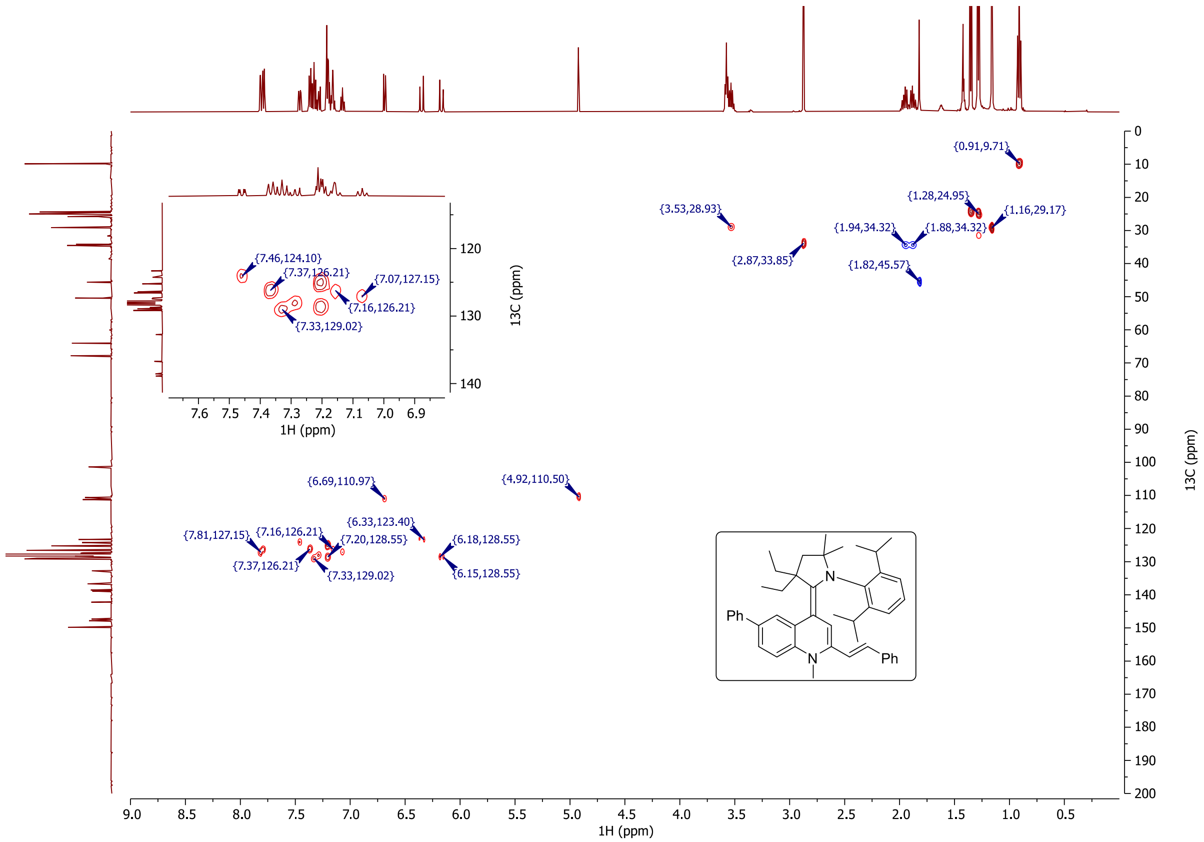

Figure S140: ^{1}**H/**^{13}**C HSQC** (500/126 MHz, C_6D_6, 298K) of **2j**.

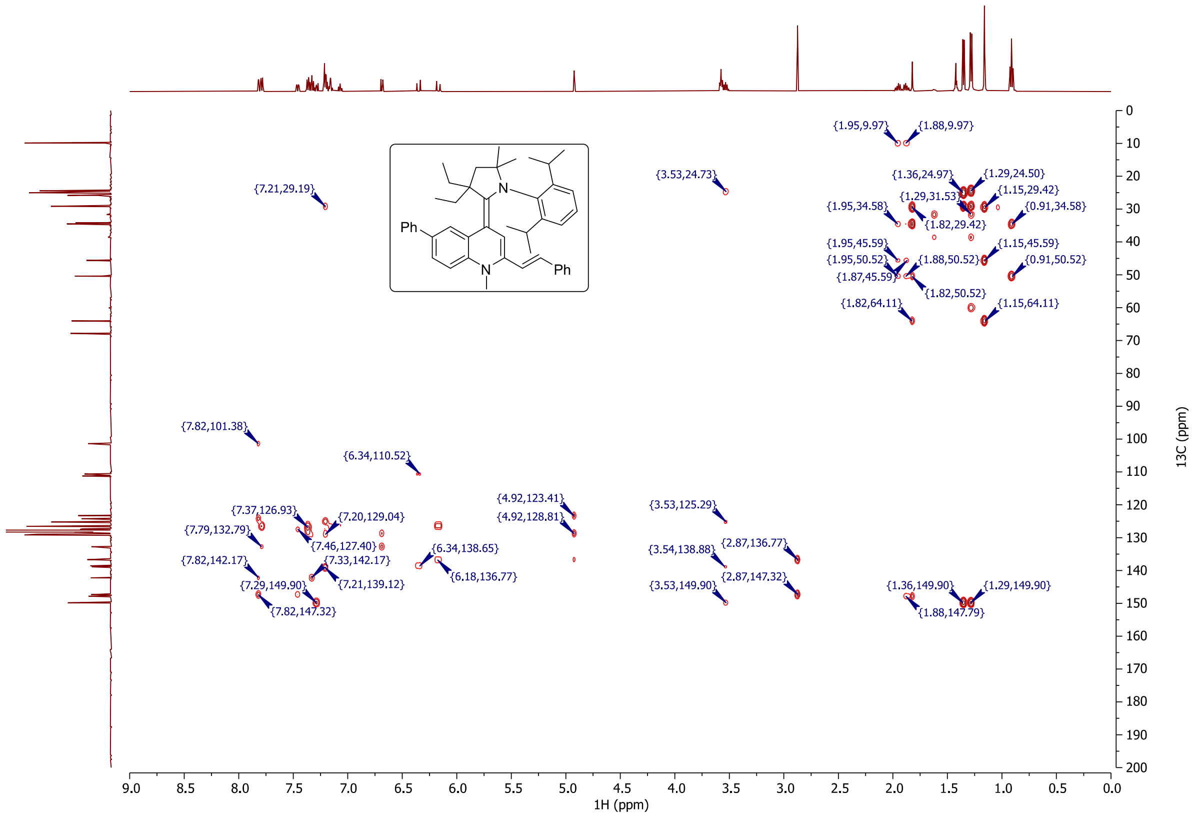

Figure S141: ^{1}H/^{13}C HMBC (500/126 MHz, C_6D_6, 298K) of **2j**.

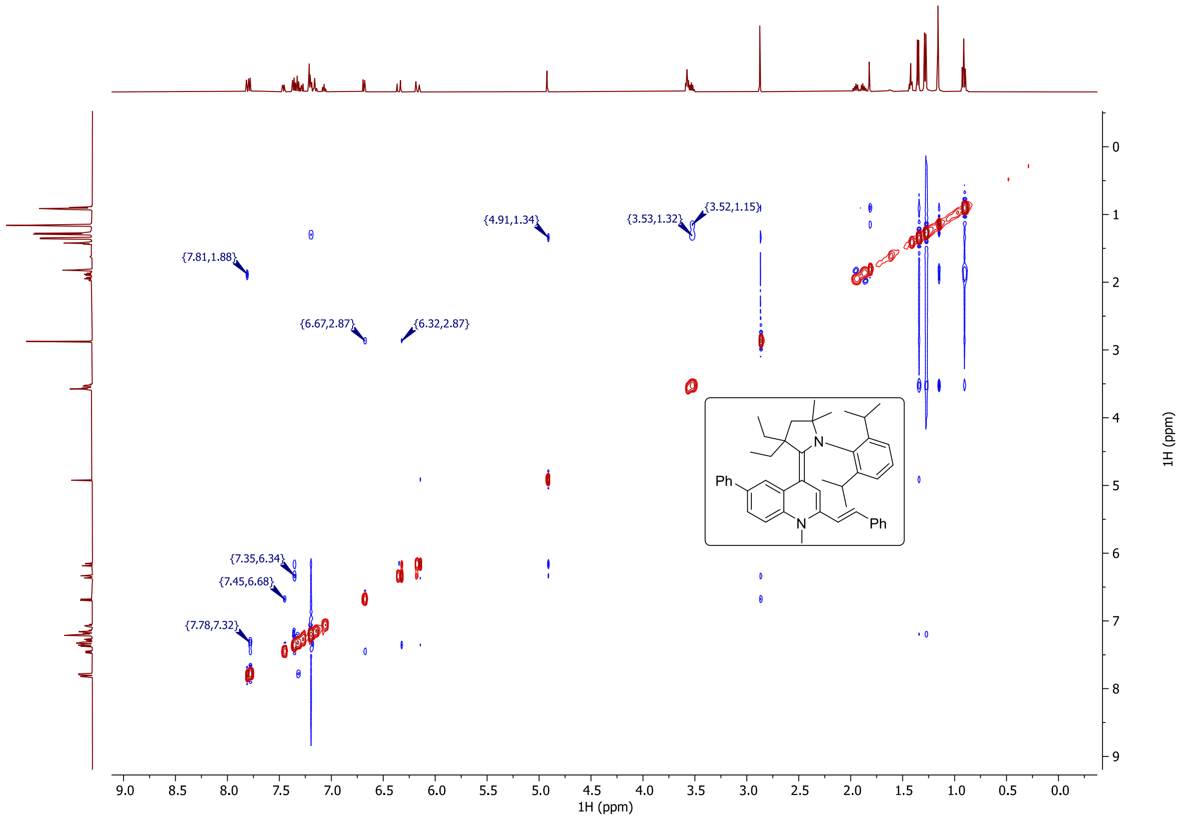

Figure S142: NOESY-NMR (500 MHz, C_6D_6, 298K) of **2j**.

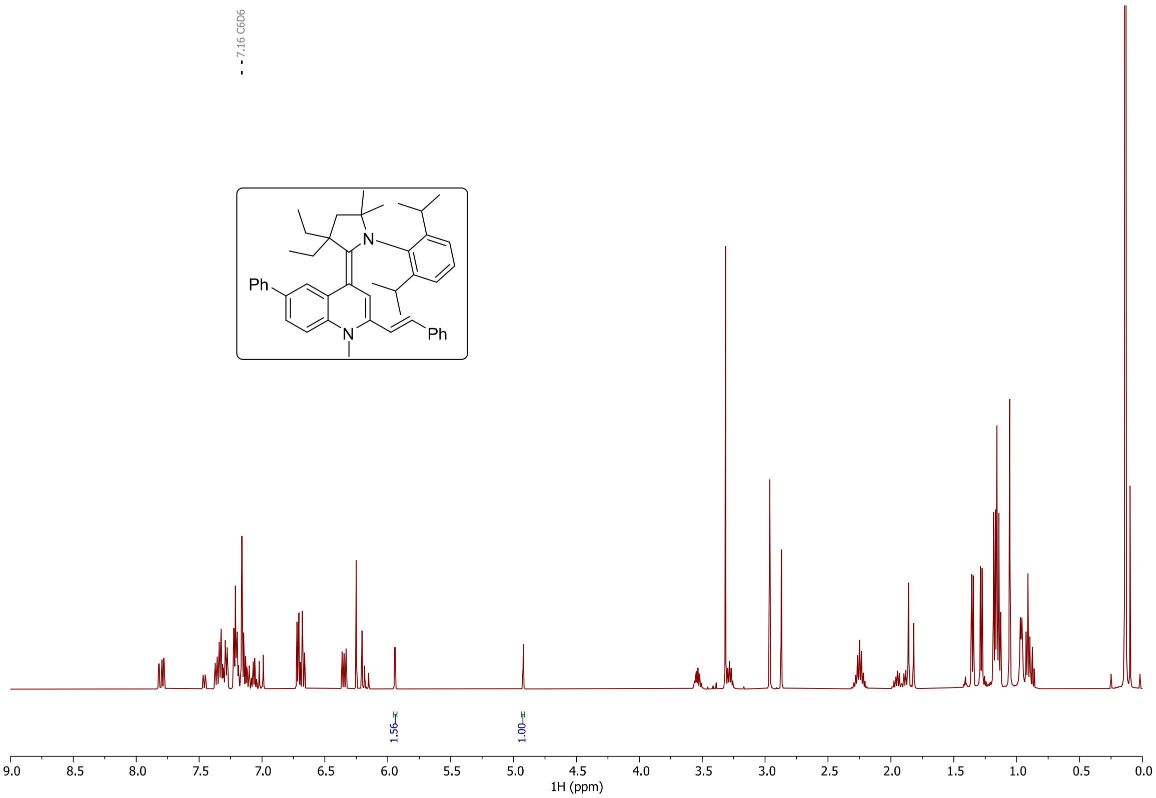

Figure S143: ^{1}H NMR (500 MHz, C_6D_6, 298 K) of **2j** after irradiation λ = 427 nm, 1h; *E*:*Z* = 1:1.56. Contains Trimethoxy-benzene as internal standard and KHMDS. Indicative signals are integrated to represent the isomers.

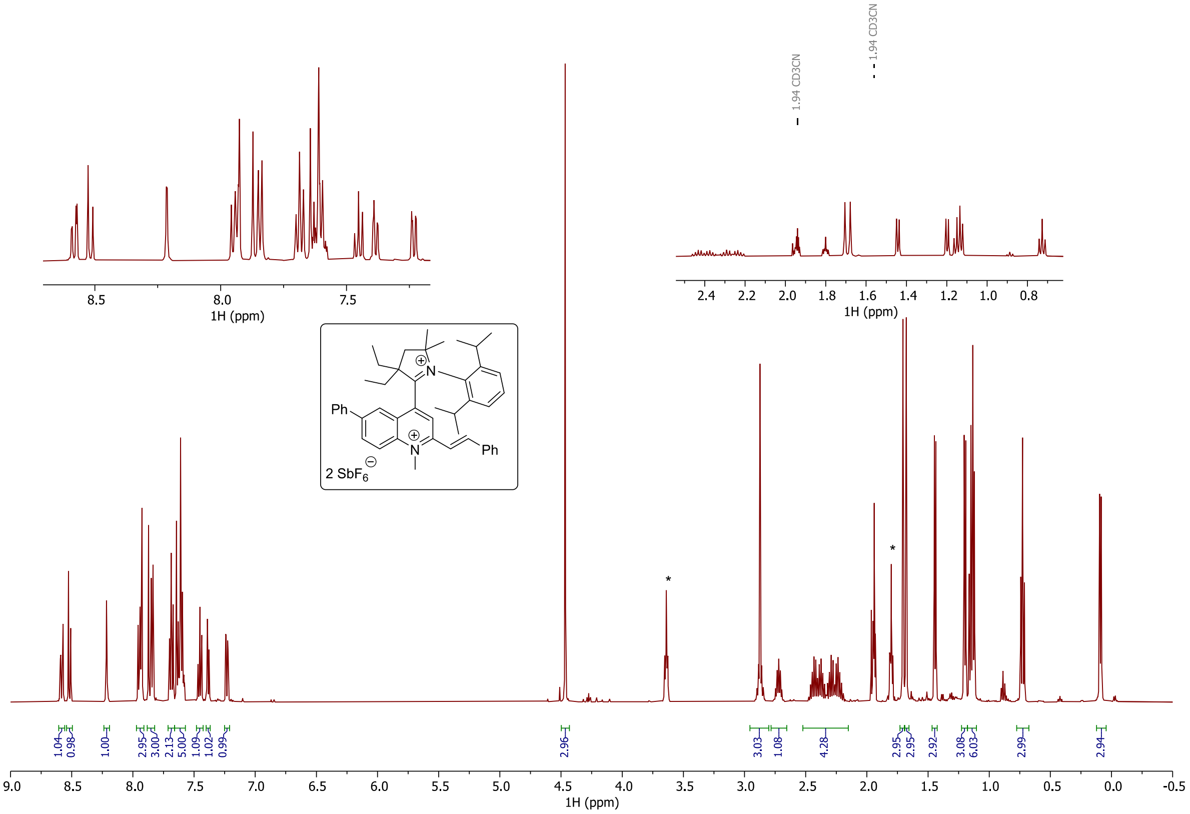

Figure S144: ^{1}H NMR (500 MHz, CD_3CN, 298K) of **4j**, residual thf is marked with a *.

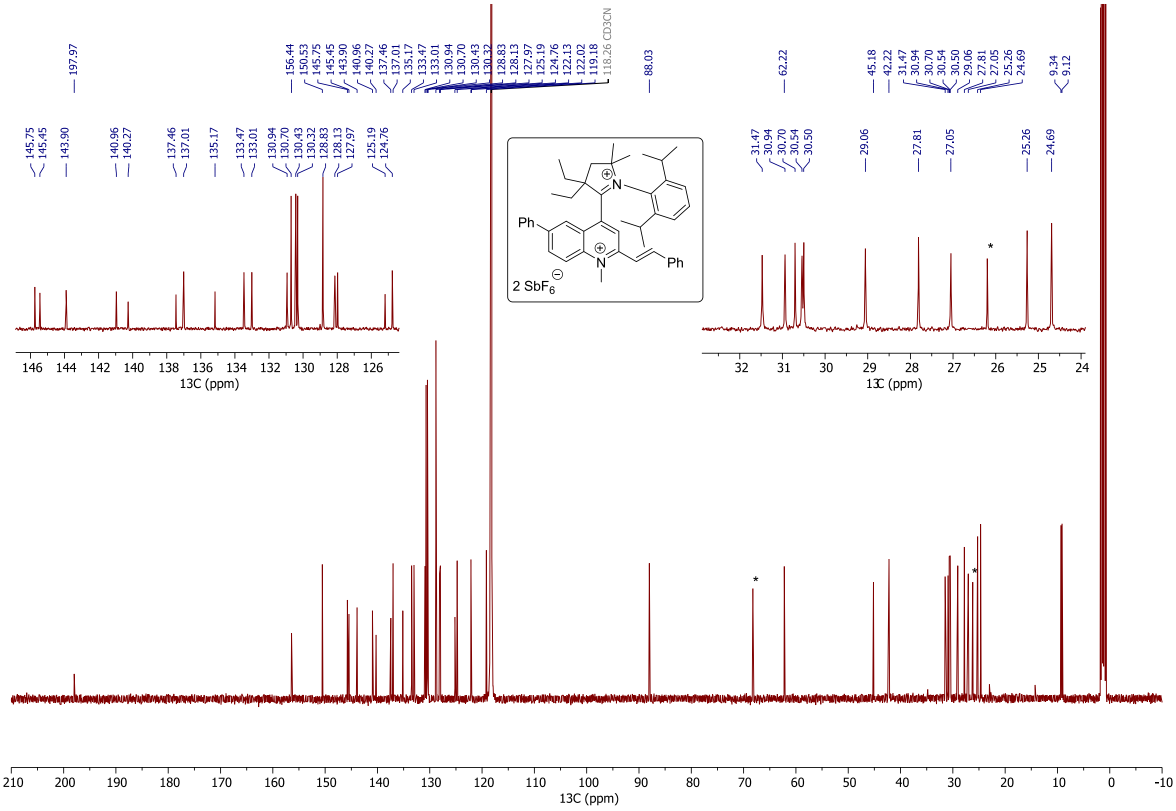

Figure S145: ^{13}C {^{1}H} NMR (126 MHz, CD_3CN, 298K) of **4j**, residual thf is marked with a *.

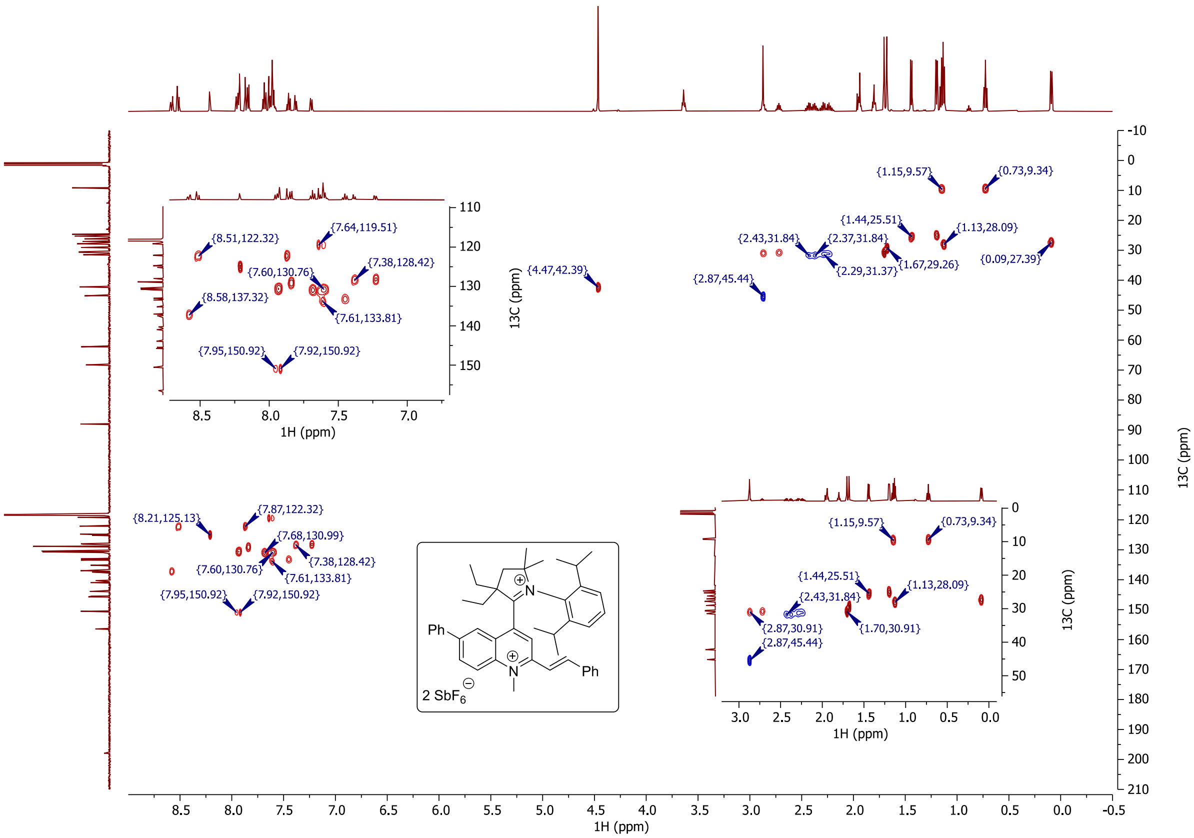

Figure S146: ^{1}H/^{13}C HSQC (500/126 MHz, CD_3CN, 298K) of **4j**.

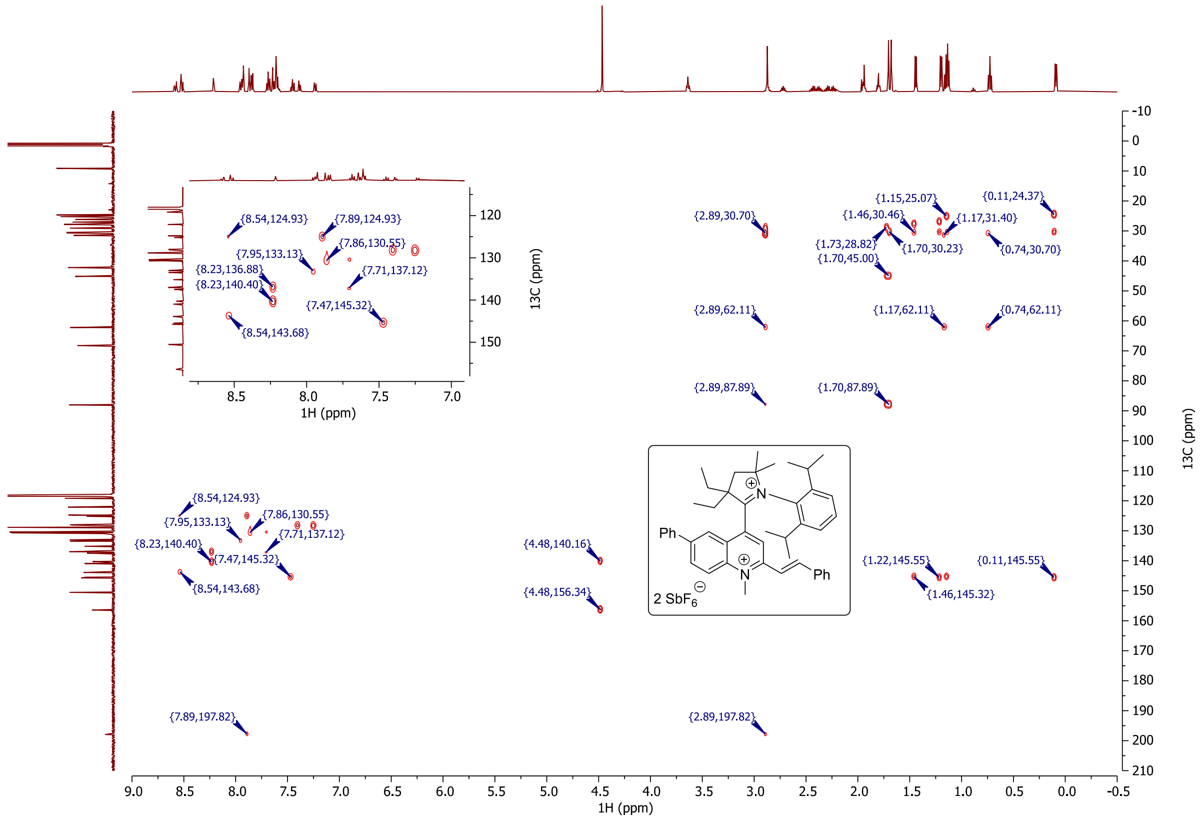

Figure S147: $^{1}H/^{13}C$ HMBC (500/126 MHz, CD_3CN, 298K) of **4j**.

EPR Spectroscopy

X-Band EPR experiments were performed using a Bruker EMX-Nano Benchtop spectrometer and a magnettech MiniScope MS400 Benchtop spectrometer. To account for magnetic field drift using the magnettech MiniScope MS400 the measurement was referenced to TEMPO in CH_3CN (0.2 mM; g = 2.0055).[12] TEMPO was measured after the investigated sample and its spectrum was then simulated. The determined difference to the literature value (for *B* and *g*) was then added/subtracted from the determined value of the investigated sample. Simulations were performed with the EasySpin program.[13]

All DFT[14,15] calculations for the prediction of EPR parameters were performed with Gaussian16 program package[16] (version g16, rev.C01). All calculations were performed with the B3LYP functionals employing Ahlrich's def2-SVP basis sets.[17,18] Ground states were fully optimized without constraints at the corresponding level of theory and their identity as a minimum were confirmed by a frequency calculation (no imaginary mode). Grimme's D3 dispersion correction with Becke-Johnson damping was used in order to take dispersion effects into account.[19,20] Isotopic hyperfine constants and Mulliken spin densities were calculated at the M06-2X/cc-pVDZ level of theory. For the visualization of frontier molecular orbitals IboView[21], GaussView 6.1[22] and Chemcraft[23] were used. Mulliken spin-densities are displayed as normalized values utilizing the Kusanagi programme.[24]

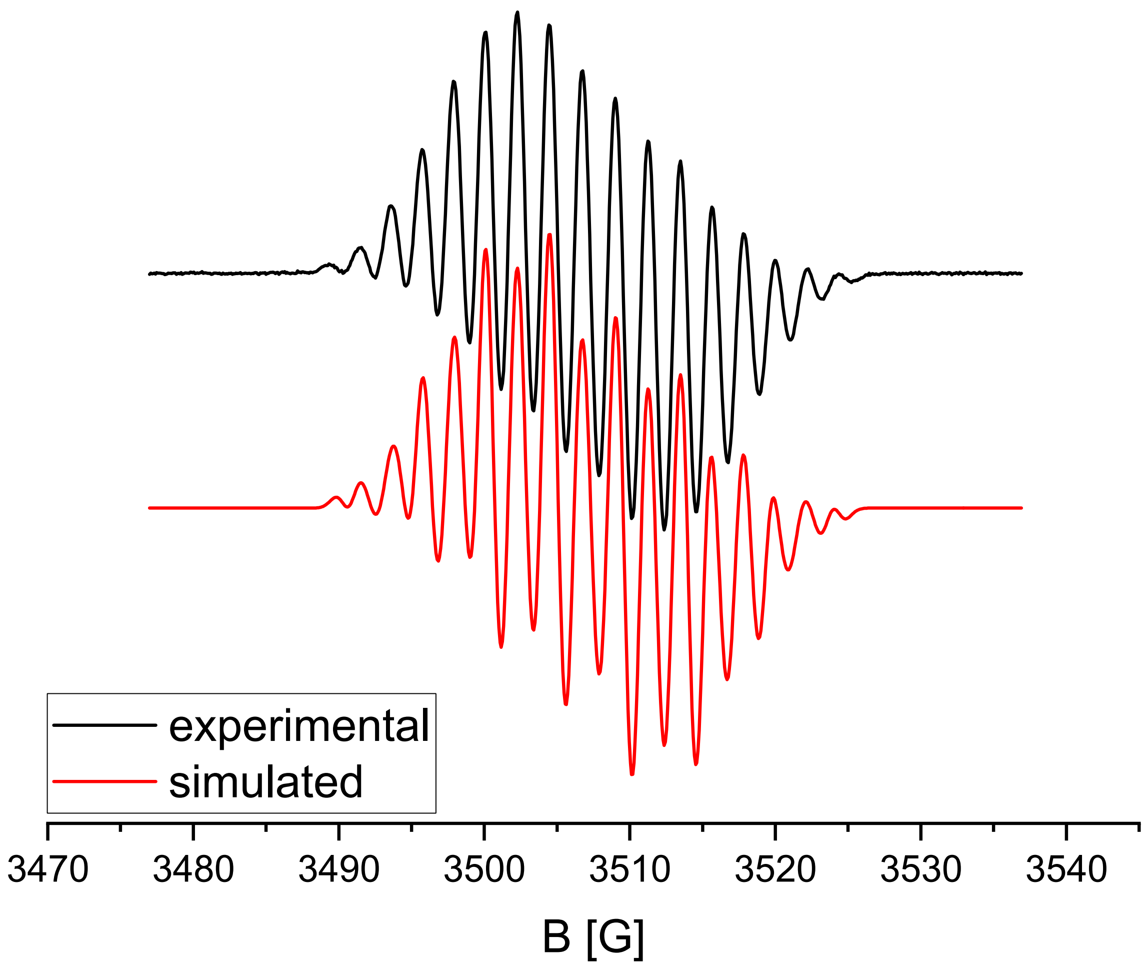

Figure S148: X-Band EPR spectrum of **3a** in thf (1 mM).
Fitting parameter: g= 2.0031; LW 0.132
Hyperfine coupling: 1xN 13.29; 2xN 6.0485; 1xH 4.246; 1xH 4.1803; 3xH 12.0721.

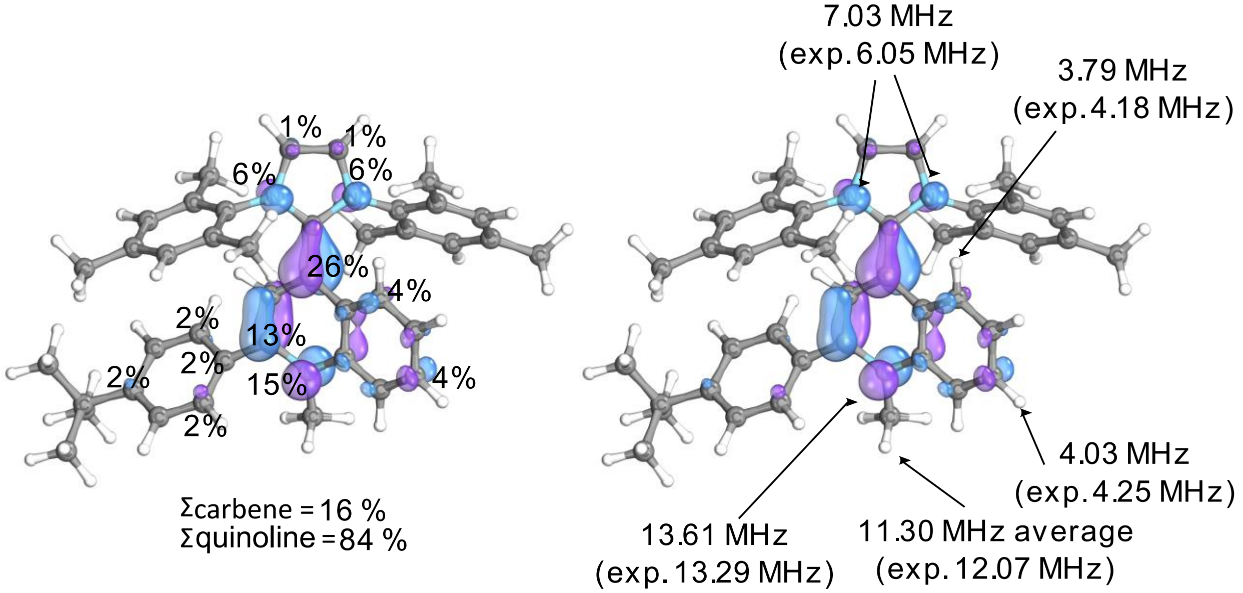

Figure S149: Left: SOMO of **3a** with the significant Mulliken spin-densities (in %). Isovalue: 0.45; Right: SOMO of **3a** with the calculated hfcs. Isovalue: 0.45.

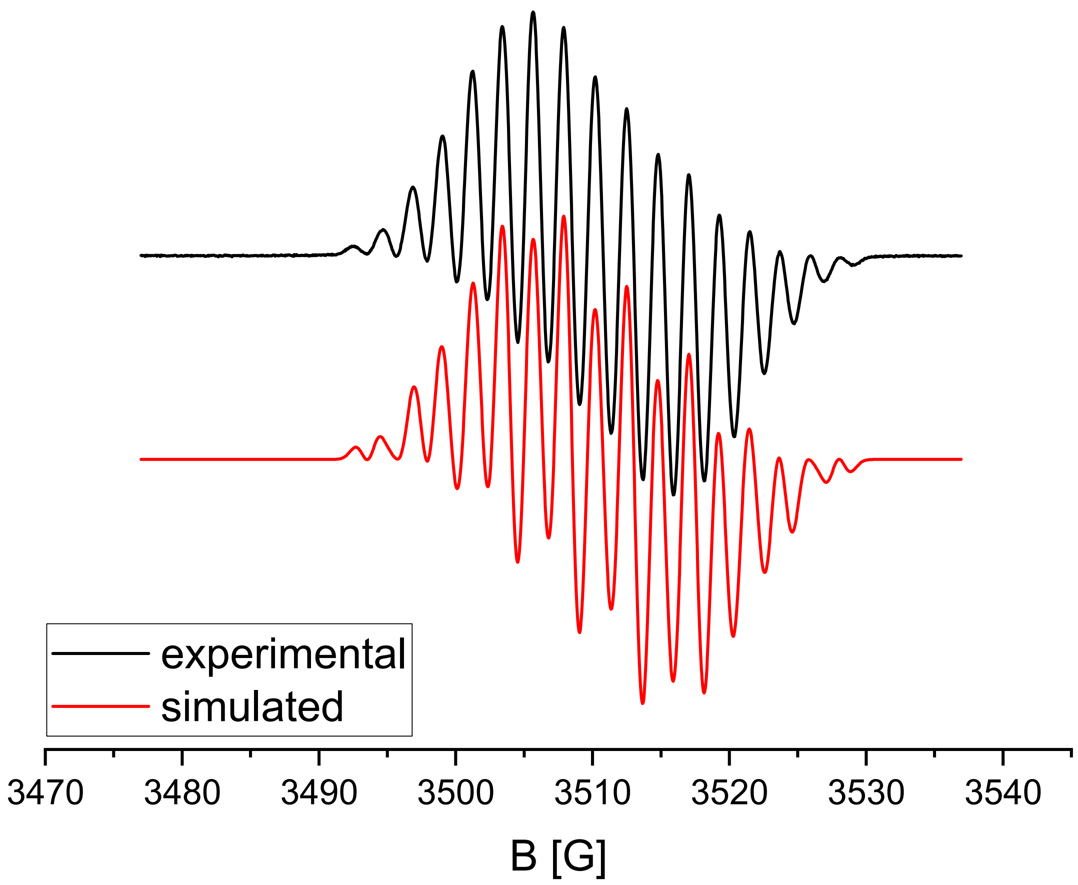

Figure S150: X-Band EPR spectrum of **3b** in thf (1 mM).
Fitting parameter: g= 2.0031; LW 0.1343
Hyperfine coupling: 1xN 13.4045; 2xN 6.7040; 1xH 3.9405; 1xH 4.6503; 3xH 12.0597.

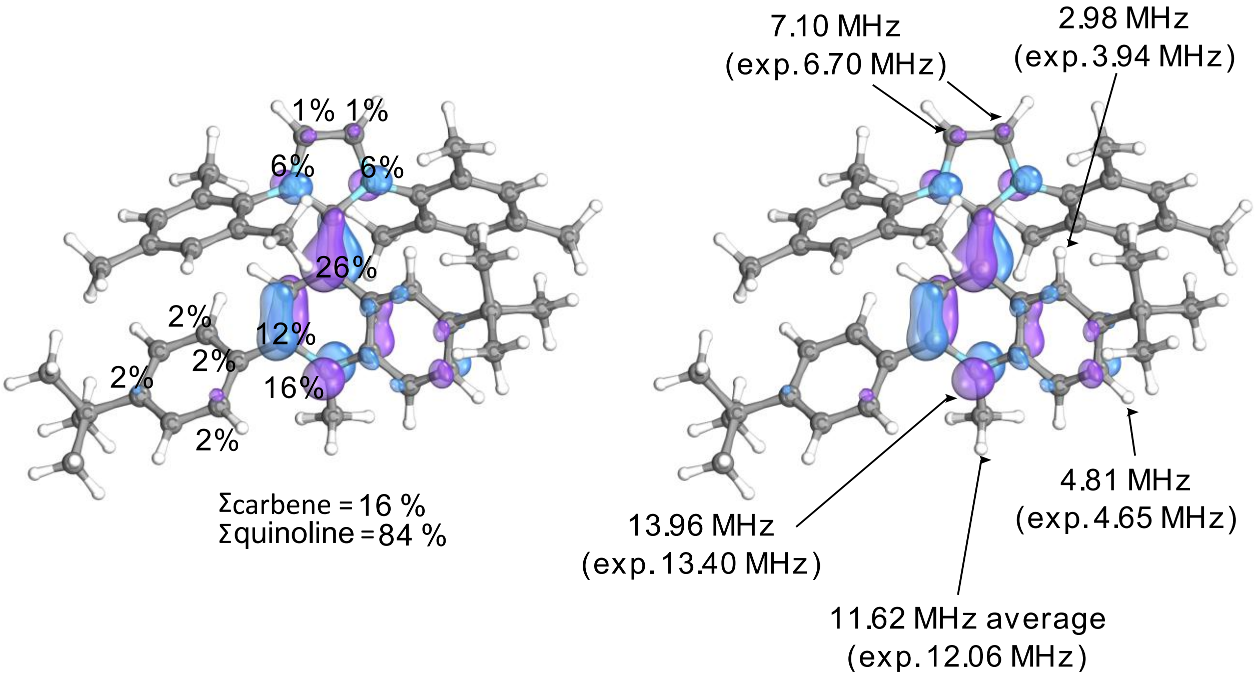

Figure S151: Left: SOMO of **3b** with the significant Mulliken spin-densities (in %). Isovalue: 0.45; Right: SOMO of **3b** with the calculated hfcs. Isovalue: 0.45.

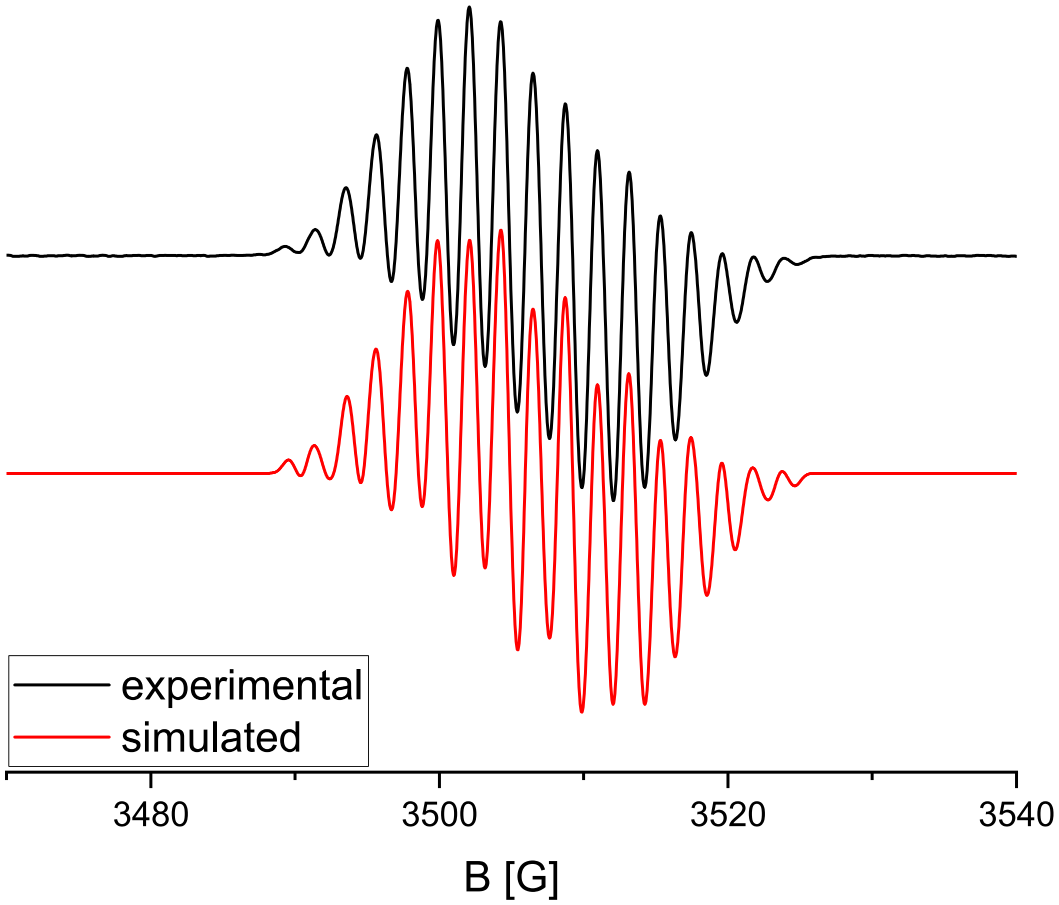

Figure S152: X-Band EPR spectrum of **3c** in thf (1 mM).
Fitting parameter: g= 2.0030; LW 0.1062
Hyperfine coupling: 1xN 13.1514; 1xN 6.0936; 1xN 6.8074; 1xH 4.0244; 1xH 4.5544; 1xH 1.5309; 3xH 11.4908.

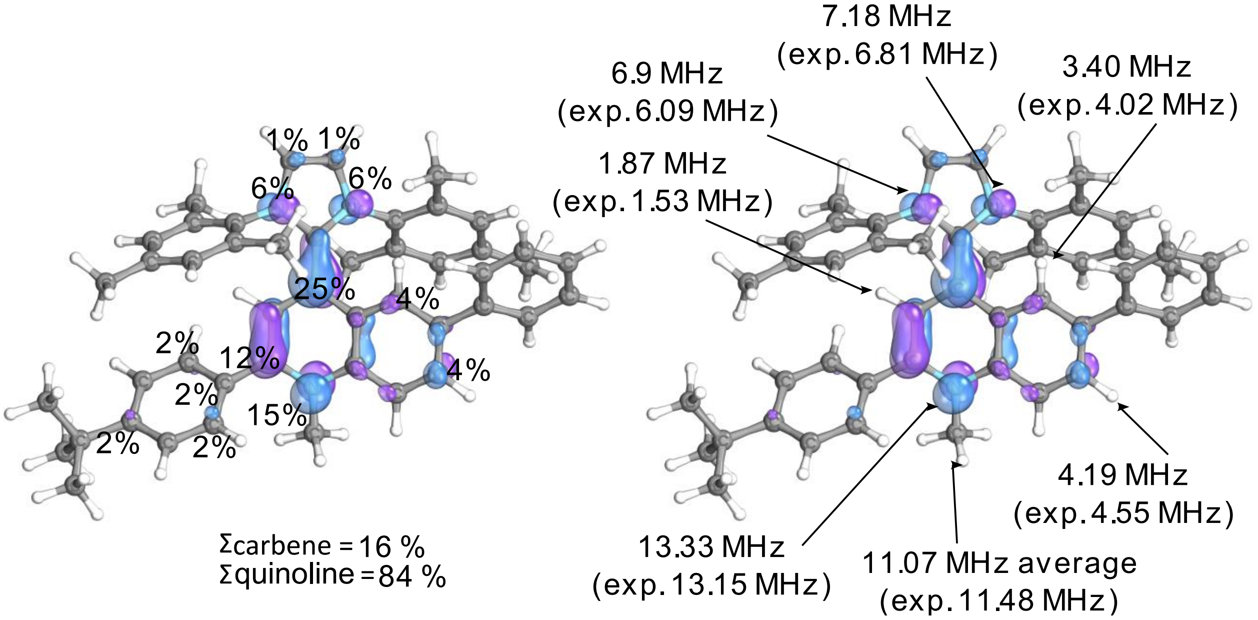

Figure S153: Left: SOMO of **3c** with the significant Mulliken spin-densities (in %). Isovalue: 0.45; Right: SOMO of **3c** with the calculated hfcs. Isovalue: 0.45.

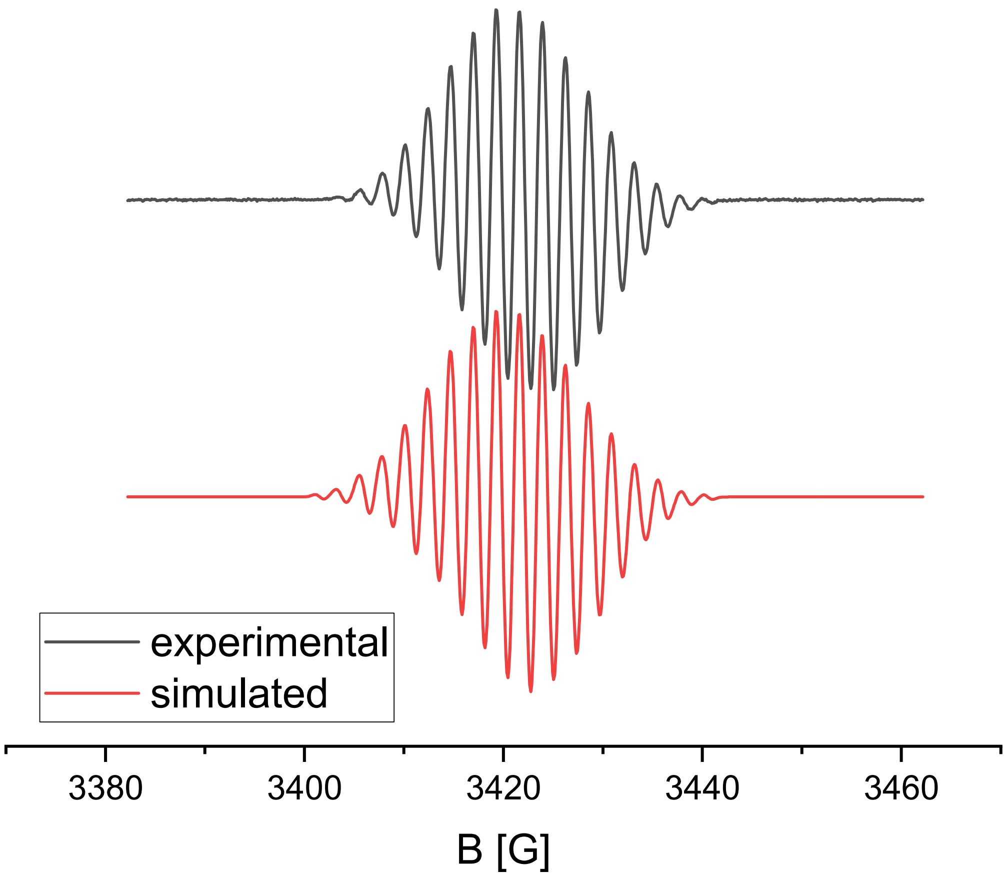

Figure S154: X-Band EPR spectrum of **3d** in thf (1 mM).
Fitting parameter: g= 2.0036; LW 0.1127
Hyperfine coupling: 1xN 12.4644; 1xN 6.3077; 1xN 6.3061; 1xH 8.2999; 1xH 4.7328; 1xH 6.4536; 3xH 13.3210.

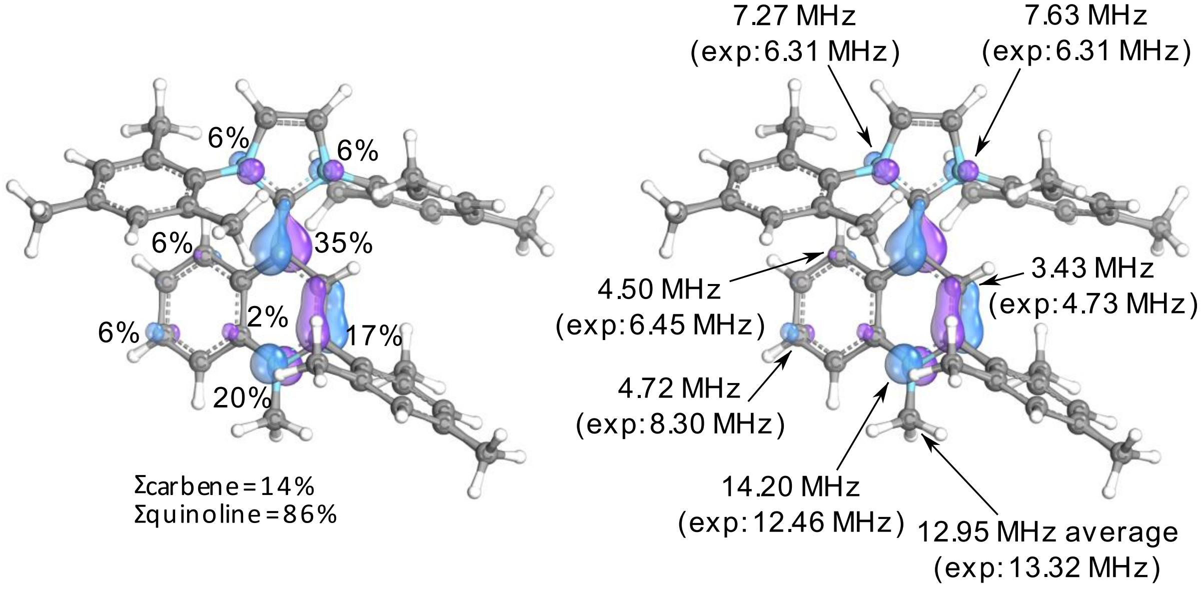

Figure S155: Left: SOMO of **3d** with the significant Mulliken spin-densities (in %). Isovalue: 0.35; Right: SOMO of **3d** with the calculated hfcs. Isovalue: 0.35.

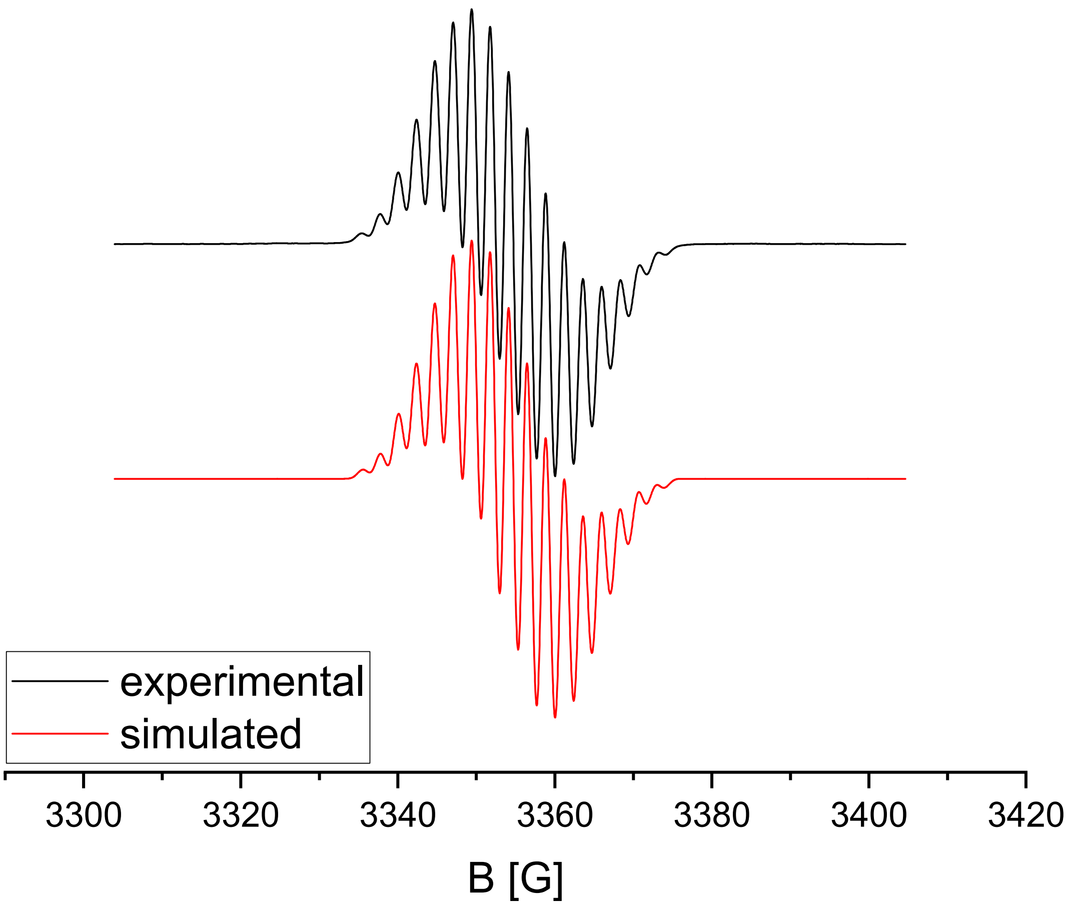

Figure S156: X-Band EPR spectrum of **3e** in thf (1 mM).
Fitting parameter: g= 2.0028; LW 0.1397
Hyperfine coupling: 1xN 13.1510; 1xN 7.6787; 1xN 5.6376; 1xH 5.5131; 1xH 1.8653; 1xH 5.6380; 1xH 2.0472; 1xH 1.5928; 3xH 12.5349.

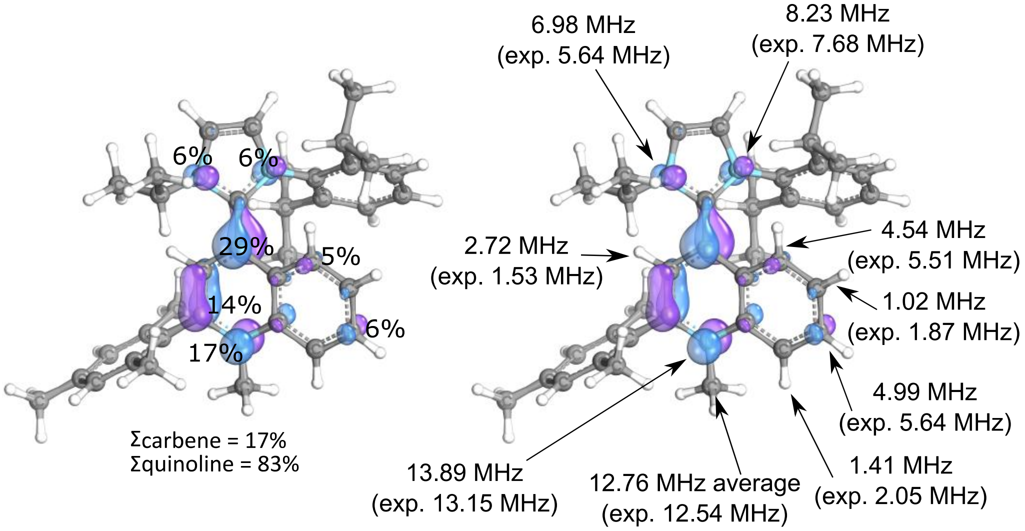

Figure S157: Left: SOMO of **3e** with the significant Mulliken spin-densities (in %). Isovalue: 0.40; Right: SOMO of **3e** with the calculated hfcs. Isovalue: 0.40.

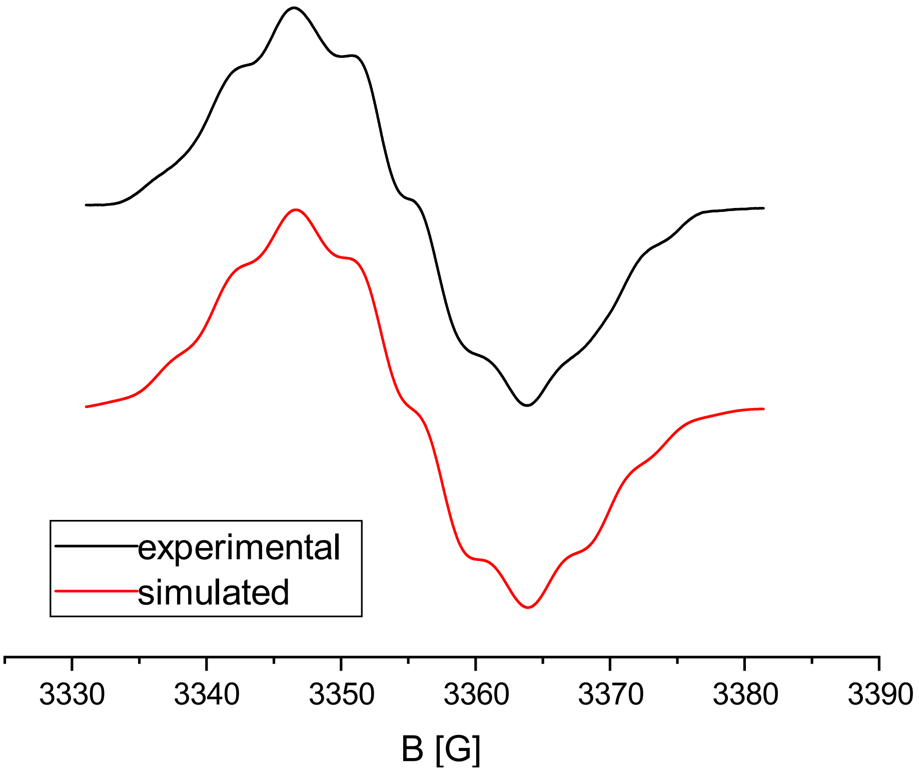

Figure S158: X-Band EPR spectrum of **3f** in thf (1 mM).
Fitting parameter: g = 1.9847; LW 0.4257
Hyperfine coupling: 1xN: 7.7337 MHz, 1xN: 14.9389 MHz; 1xH: 0.6539 MHz; 1xH: 0.6208 MHz; 3xH: 10.0005 MHz; 1xH: 13.0936 MHz; 1xH: 10.3416 MHz. The EPR spectrum is referenced to TEMPO (2 mM in CH_3CN; literature value: g = 2.0055[12]).

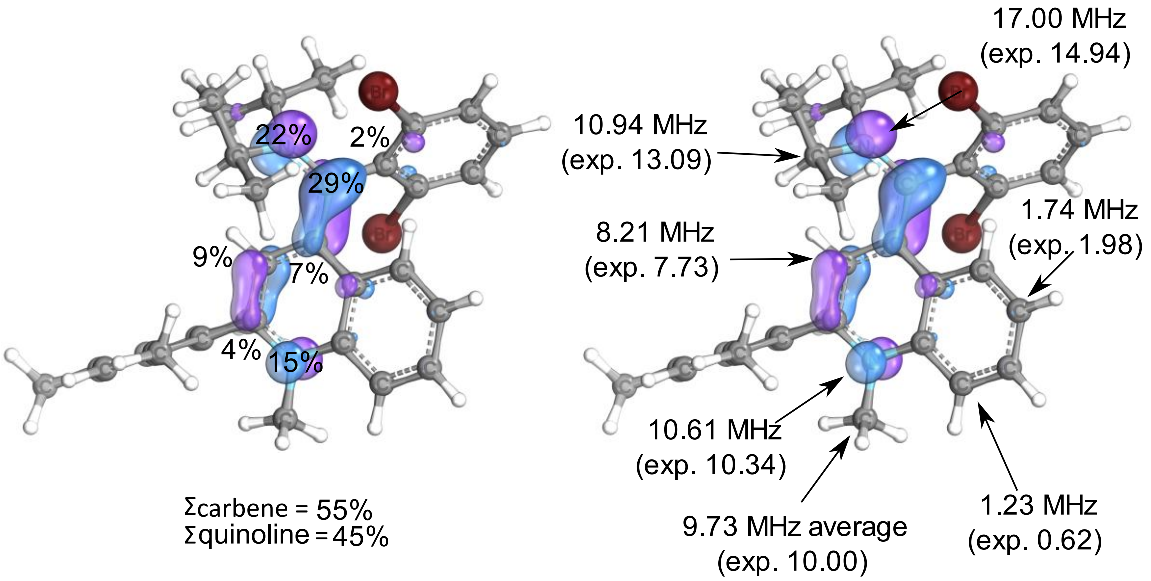

Figure S159: Left: SOMO of **3f** with the significant MULLIKEN spin-densities (in %). Isovalue: 0.40; Right: SOMO of **3f**with the calculated hfcs. Isovalue: 0.40.

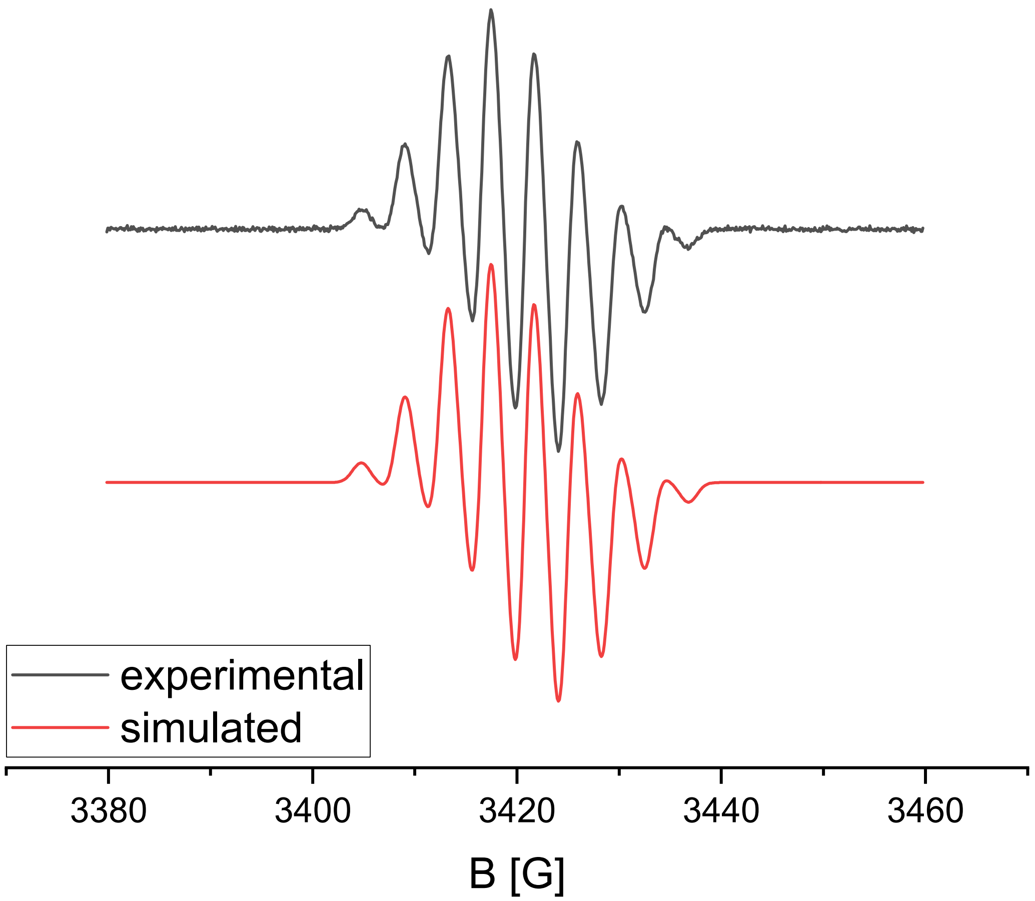

Figure S160: X-Band EPR spectrum of **3g** in thf (1 mM).
Fitting parameter: g= 2.0035; LW 0.1943
Hyperfine coupling: 1xN 11.4398; 1xN 10.7994; 1xH 4.4854; 1xH 1.8865; 1xH 2.0414; 1xH 0.5494; 3xH 12.1624.

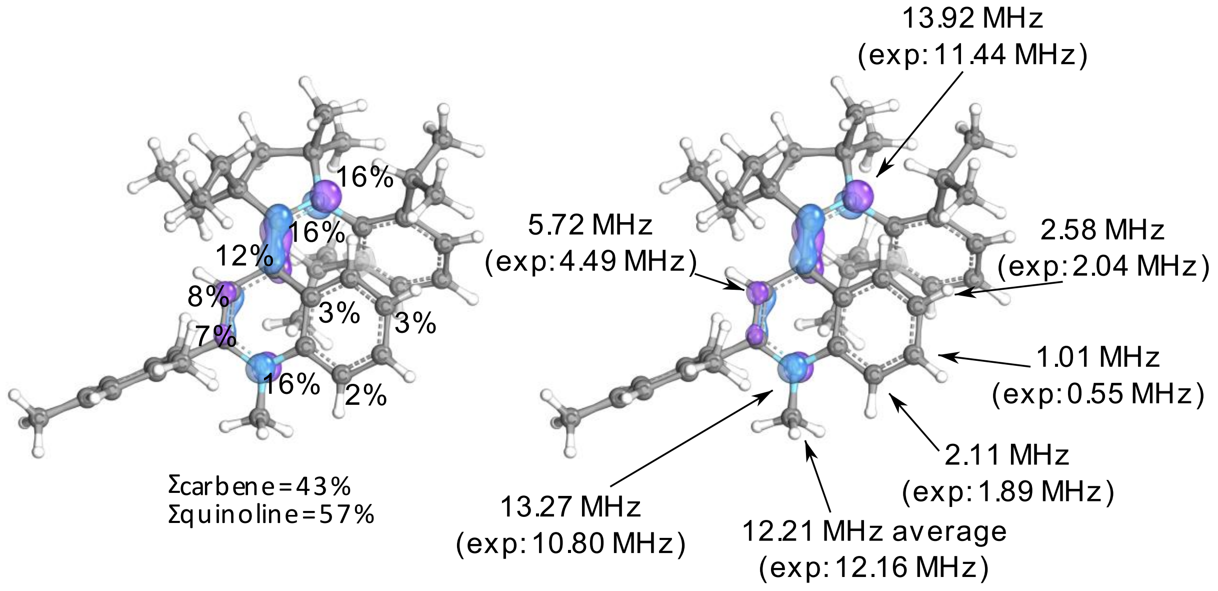

Figure S161: Left: SOMO of **3g** with the significant Mulliken spin-densities (in %). Isovalue: 0.30; Right: SOMO of **3g** with the calculated hfcs. Isovalue: 0.30.

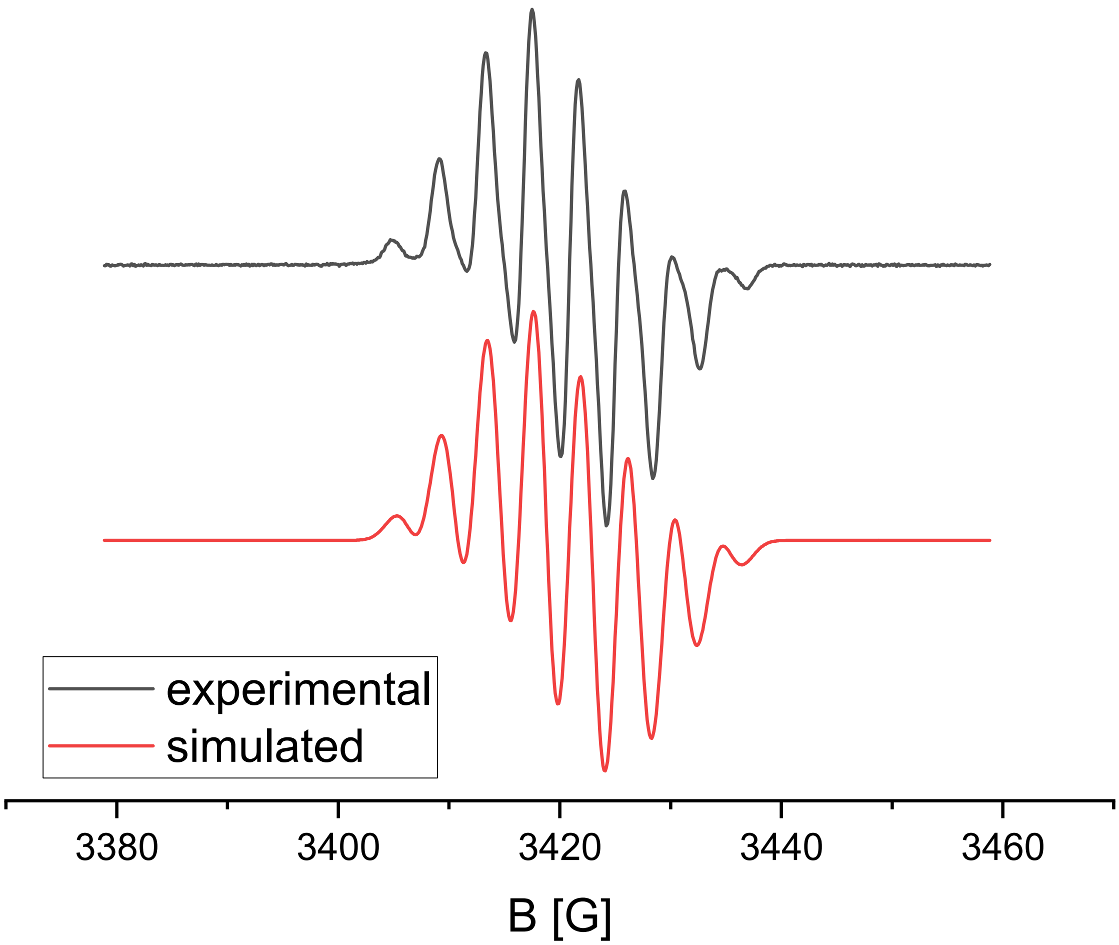

Figure S162: X-Band EPR spectrum of **3h** in thf (1 mM).
Fitting parameter: g= 2.0035; LW 0.3194
Hyperfine coupling: 1xN 13.0355; 1xN 10.4437; 3xH 11.0355.

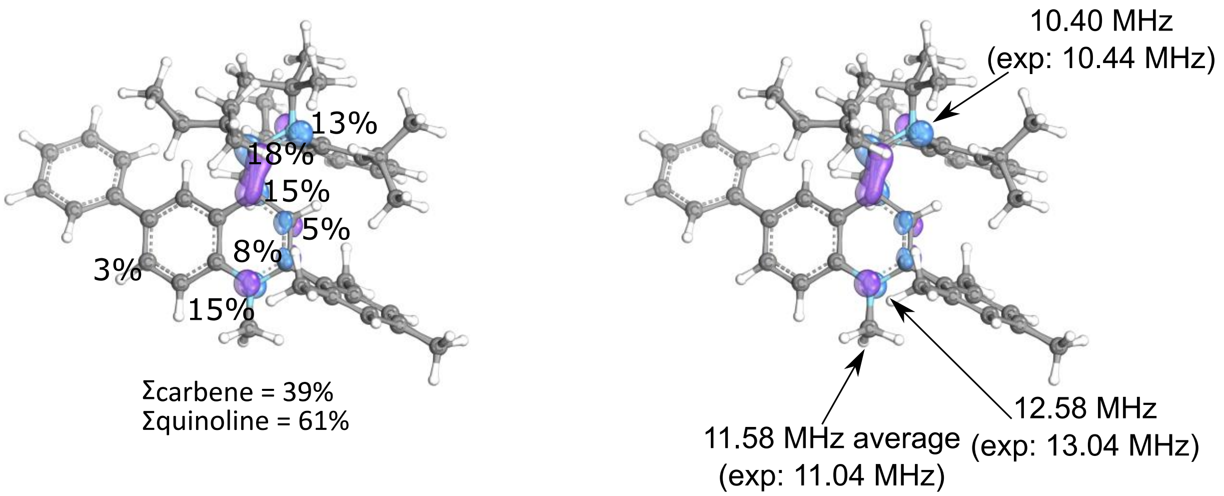

Figure S163: Left: SOMO of **3h** with the significant Mulliken spin-densities (in %). Isovalue: 0.30; Right: SOMO of **3h** with the calculated hfcs. Isovalue: 0.30.

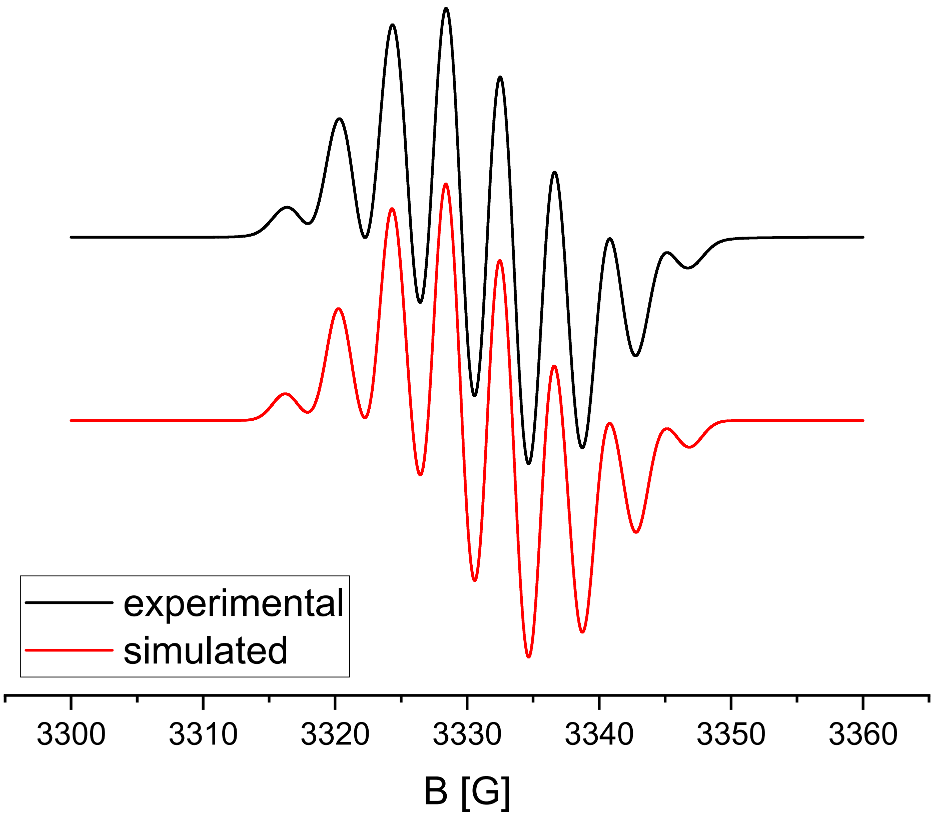

Figure S164: X-Band EPR spectrum of **3i** in thf (1 mM).
Fitting parameter: g = 2.0034; LW 0.2559
Hyperfine coupling: 1xN: 10.0799 MHz; 1xN: 11.9770 MHz 1xH: 4.3644 MHz; 3xH: 10.9798 MHz.

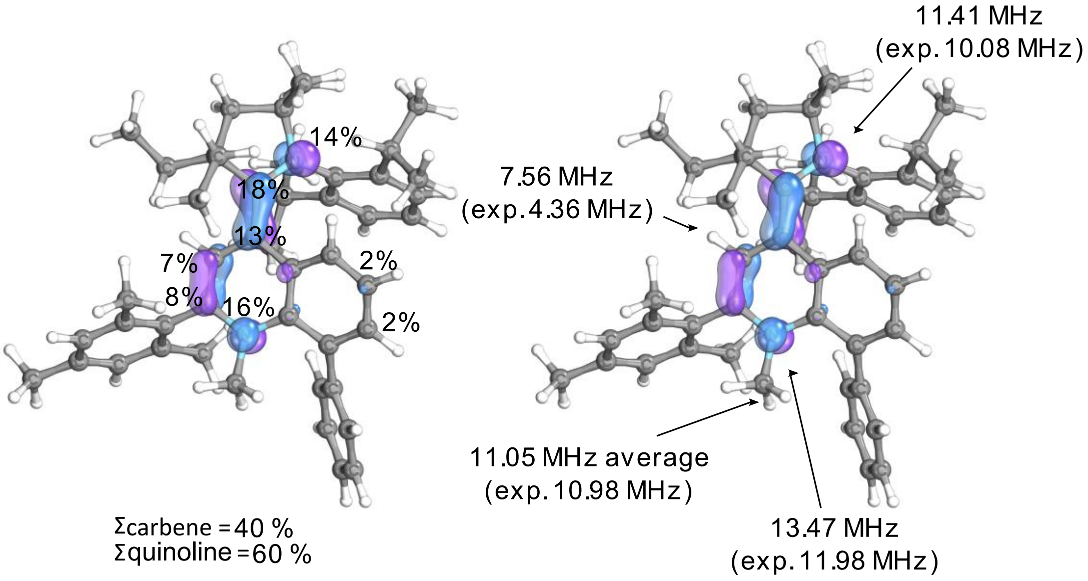

Figure S165: Left: SOMO of **3i⁺** with the significant Mulliken spin-densities (in %). Isovalue: 0.33; Right SOMO of **3i** with the calculated hfcs. Isovalue: 0.33.

X-ray Characterization

2a

A red, plate-shaped crystal was mounted on a MiTeGen micromount with perfluoroether oil. Data for mo_0852_CG_0m were collected from a shock-cooled single crystal at 100(2) K on a Bruker D8 VENTURE dual wavelength Mo/Cu four-circle diffractometer with a microfocus sealed X-ray tube using a mirror optics as monochromator and a Bruker PHOTON II detector. The diffractometer used MoK_α radiation (λ = 0.71073 Å). All data were integrated with SAINT V8.40B and a multi-scan absorption correction using SADABS 2016/2 was applied.[25,26] The structure was solved by direct methods with SHELXS and refined by full-matrix least-squares methods against F^2 using SHELXL-2019/2.[27,28] All non-hydrogen atoms were refined with anisotropic displacement parameters. All hydrogen atoms were refined isotropic on calculated positions using a riding model with their U_{iso} values constrained to 1.5 times the U_{eq} of their pivot atoms for terminal sp^3 carbon atoms and 1.2 times for all other carbon atoms. Crystallographic data for the structures reported in this paper have been deposited with the Cambridge Crystallographic Data Centre.[29] CCDC 2535132 contain the supplementary crystallographic data for this paper. These data can be obtained free of charge from The Cambridge Crystallographic Data Centre via www.ccdc.cam.ac.uk/structures. This report and the CIF file were generated using FinalCif.[30]

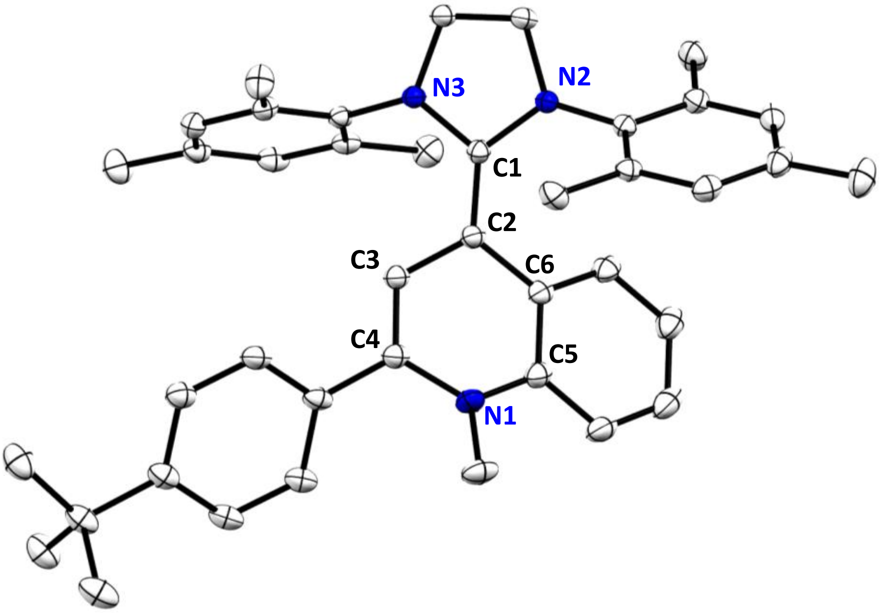

Figure S166: X-ray solid-state structure of **2a**. Hydrogen atoms were omitted for clarity. Thermal ellipsoids are shown with 50 % probability. Selected bond lengths [Å] and angles in [°]: 1.3920(13), C1–C2 1.3889(14), C2–C3 1.4509(14), C3–C4 1.3536(14), C4–N1 1.4178(14), N1–C5 1.4054(15), 1.4191(15), C6–C2 1.4654(14), N3–C1–C2–C3 −27.33(16).

4a

A colourless, plate-shaped crystal was mounted on a MiTeGen micromount with perfluoroether oil. Data for mo_0899_CG_0m_4 were collected from a shock-cooled single crystal at 100(2) K on a Bruker D8 VENTURE dual wavelength Mo/Cu four-circle diffractometer with a microfocus sealed X-ray tube using a mirror optics as monochromator and a Bruker PHOTON II detector. The diffractometer was equipped with an Oxford Cryostream 800 low temperature device and used MoK_α radiation (λ = 0.71073 Å). All data were integrated with SAINT V8.40B and a multi-scan absorption correction using TWINABS Bruker was applied.[25,26] The structure was solved by direct methods with SHELXT and refined by full-matrix least-squares methods against F^2 using SHELXL-2019/2.[27,28] All non-hydrogen atoms were refined with anisotropic displacement parameters. All hydrogen atoms were refined isotropic on calculated positions using a riding model with their U_{iso} values constrained to 1.5 times the U_{eq} of their pivot atoms for terminal sp^3 carbon atoms and 1.2 times for all other carbon atoms. Crystallographic data for the structures reported in this paper have been deposited with the Cambridge Crystallographic Data Centre.[29] CCDC 2535133 contain the supplementary crystallographic data for this paper. These data can be obtained free of charge from The Cambridge Crystallographic Data Centre via www.ccdc.cam.ac.uk/structures. This report and the CIF file were generated using FinalCif.[30]

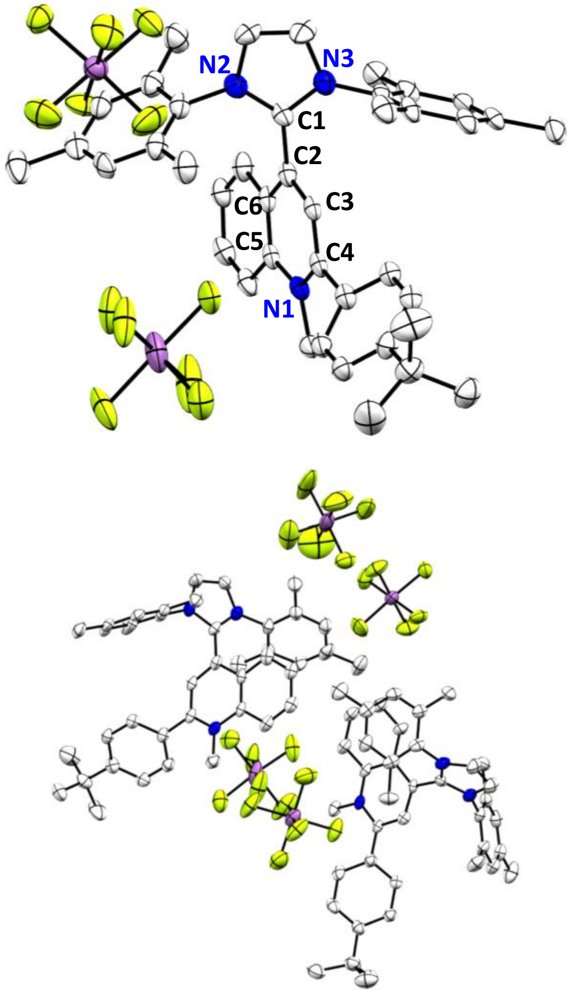

Figure S167: X-ray solid-state structure of **4a**. Hydrogen atoms were omitted for clarity as well as one thf molecule. Thermal ellipsoids are shown with 50 % probability. Selected bond lengths [Å] and angles in [°]:N2_1–C1_1 1.338(11)/ N2_2–C1_2 1.333(11), C1_1–C2_1 1.463(10)/ C1_2–C2_2 1.491(12), C2_1–C3_1 1.385(11)/ C2_2–C3_2 1.384(12), C3_1–C4_1 1.405(11)/ C3_2–C4_2 1.382(12), C4_1– N1_1 1.352(10)/ C4_2– N1_2 1.349(10) N1_1–C5_1 1.388(11) / N1_2–C5_2 1.411(11), C5_1–C6_1 1.428(11)/ C5_2–C6_2 1.395(12), C6_1–C2_1 1.423(11)/ C6_2–C2_2 1.416(11), N3_1–C1_1–C2_1–C3_1 52.0(11)/ N3_2–C1_2–C2_2–C3_2 53.1(11).

2c

A orange, plate-shaped crystal was mounted on a MiTeGen micromount with perfluoroether oil. Data for cb932e were collected from a shock-cooled single crystal at 100(2) K on a Bruker D8 VENTURE dual wavelength Mo/Cu four-circle diffractometer with a microfocus sealed X-ray tube using a mirror optics as monochromator and a Bruker PHOTON II detector. The diffractometer used MoK_{α} radiation (λ = 0.71073 Å). All data were integrated with SAINT V8.B40and a multi-scan absorption correction using SADABS 2016/2 was applied.[25,26] The structure was solved by direct methods with SHELXT and refined by full-matrix least-squares methods against F^2 using SHELXL-2019/2.[27,28] All non-hydrogen atoms were refined with anisotropic displacement parameters. All hydrogen atoms were refined isotropic on calculated positions using a riding model with their U_{iso} values constrained to 1.5 times the U_{eq} of their pivot atoms for terminal sp^3 carbon atoms and 1.2 times for all other carbon atoms. Disordered moieties were refined using bond lengths restraints and displacement parameter restraints. Crystallographic data for the structures reported in this paper have been deposited with the Cambridge Crystallographic Data Centre.[29] CCDC 2535134 contain the supplementary crystallographic data for this paper. These data can be obtained free of charge from The Cambridge Crystallographic Data Centre via www.ccdc.cam.ac.uk/structures. This report and the CIF file were generated using FinalCif.[30]

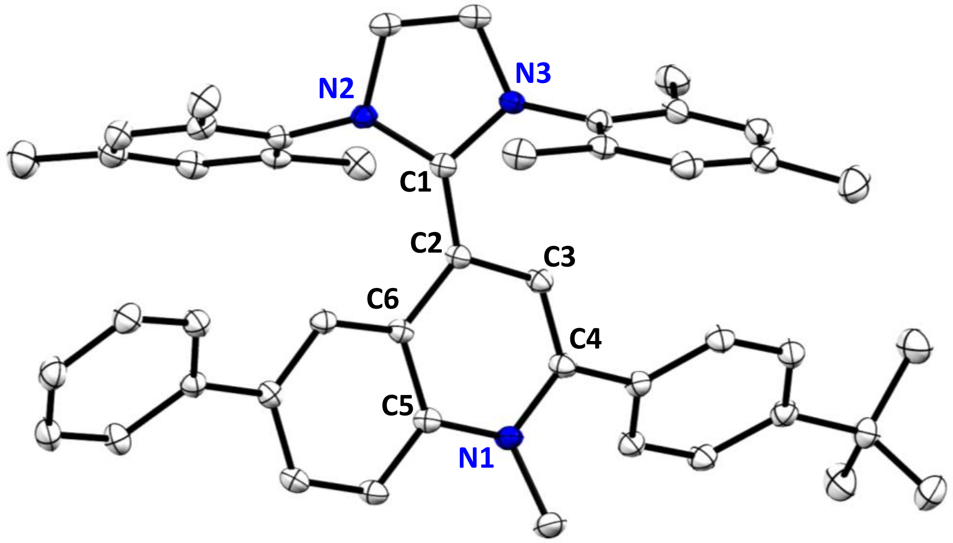

Figure S168: X-ray solid-state structure of **2c**. Hydrogen atoms were omitted for clarity as well as two Et_2O molecule. Thermal ellipsoids are shown with 50 % probability. Selected bond lengths [Å] and angles in [°]: N2–C1 1.3931(15), C1–C2 1.3912(15), C2–C3 1.4462(16), C3–C4 1.3504(15), C4– N1 1.4101(15), N1–C5 1.4065(15), C5–C6 1.4305(15), C6–C2 1.4654(16), N3–C1–C2–C3 149.31(11).

***E*-2f**

A red, block-shaped crystal was mounted on a MiTeGen micromount with perfluoroether oil. Data for cblz07b were collected from a shock-cooled single crystal at 100(2) K on a Bruker D8 VENTURE dual wavelength Mo/Cu four-circle diffractometer with a microfocus sealed X-ray tube using a mirror optics as monochromator and a Bruker PHOTON II detector. The diffractometer used MoK_α radiation (λ = 0.71073 Å). All data were integrated with SAINT V8.40B and a multi-scan absorption correction using SADABS 2016/2 was applied.[25,26] The structure was solved by direct methods with SHELXT 2018/2 and refined by full-matrix least-squares methods against F^2 using SHELXL-2019/2.[27,28] All non-hydrogen atoms were refined with anisotropic displacement parameters. All hydrogen atoms were refined isotropic on calculated positions using a riding model with their U_{iso} values constrained to 1.5 times the U_{eq} of their pivot atoms for terminal sp^3 carbon atoms and 1.2 times for all other carbon atoms. Crystallographic data for the structures reported in this paper have been deposited with the Cambridge Crystallographic Data Centre.[29] CCDC 2535135 contain the supplementary crystallographic data for this paper. These data can be obtained free of charge from The Cambridge Crystallographic Data Centre via www.ccdc.cam.ac.uk/structures. This report and the CIF file were generated using FinalCif.[30]

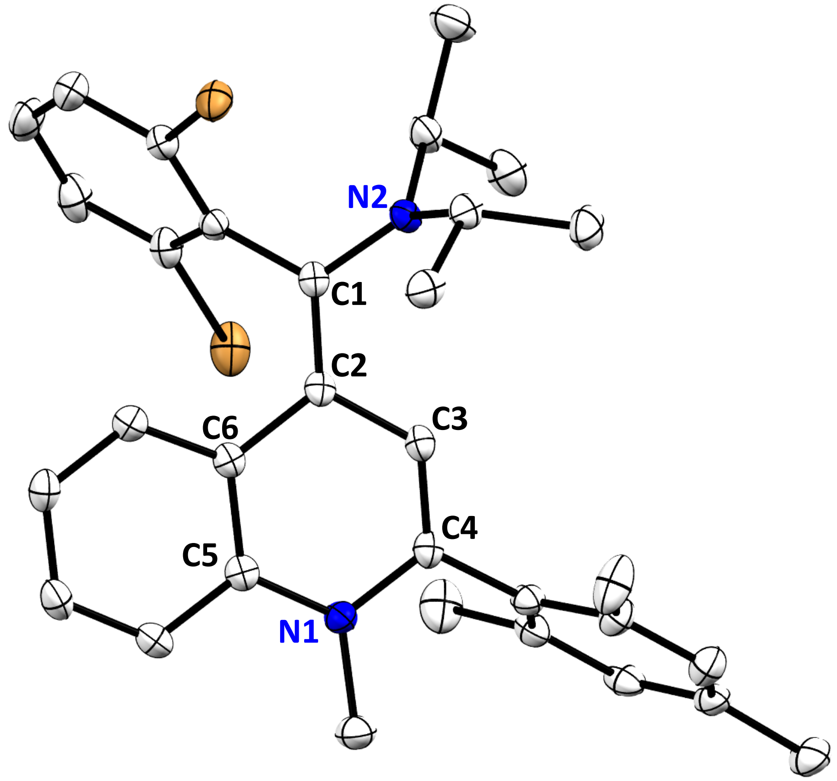

Figure S169: X-ray solid-state structure of ***E*-2f**. Hydrogen atoms were omitted for clarity. Thermal ellipsoids are shown with 50 % probability. Selected bond lengths [Å] and angles in [°]: N2–C1 1.406(3), C1–C2 1.371(3), C2–C3 1.449(3), C3–C4 1.343(3), C4– N1 1.399(3), N1–C5 1.396(3), C5–C6 1.422(3), C6–C2 1.473(3), N2–C1–C2–C3 –23.2(3).

_E-2g_

A yellow, block-shaped crystal was mounted on a MiTeGen micromount with perfluoroether oil. Data for cb86 were collected from a shock-cooled single crystal at 100(2) K on a Bruker D8 VENTURE dual wavelength Mo/Cu four-circle diffractometer with a microfocus sealed X-ray tube using a mirror optics as monochromator and a Bruker PHOTON II detector. The diffractometer used MoK_α radiation (λ = 0.71073 Å). All data were integrated with SAINT V8.40B and a multi-scan absorption correction using SADABS 2014/4 was applied.[25,26] The structure was solved by direct methods with SHELXT 2018/2 and refined by full-matrix least-squares methods against F^2 using SHELXL-2019/2.[27,28] All non-hydrogen atoms were refined with anisotropic displacement parameters. All hydrogen atoms were refined isotropic on calculated positions using a riding model with their U_{iso} values constrained to 1.5 times the U_{eq} of their pivot atoms for terminal sp^3 carbon atoms and 1.2 times for all other carbon atoms. Crystallographic data for the structures reported in this paper have been deposited with the Cambridge Crystallographic Data Centre.[29] CCDC 2535136 contain the supplementary crystallographic data for this paper. These data can be obtained free of charge from The Cambridge Crystallographic Data Centre via www.ccdc.cam.ac.uk/structures. This report and the CIF file were generated using FinalCif.[30]

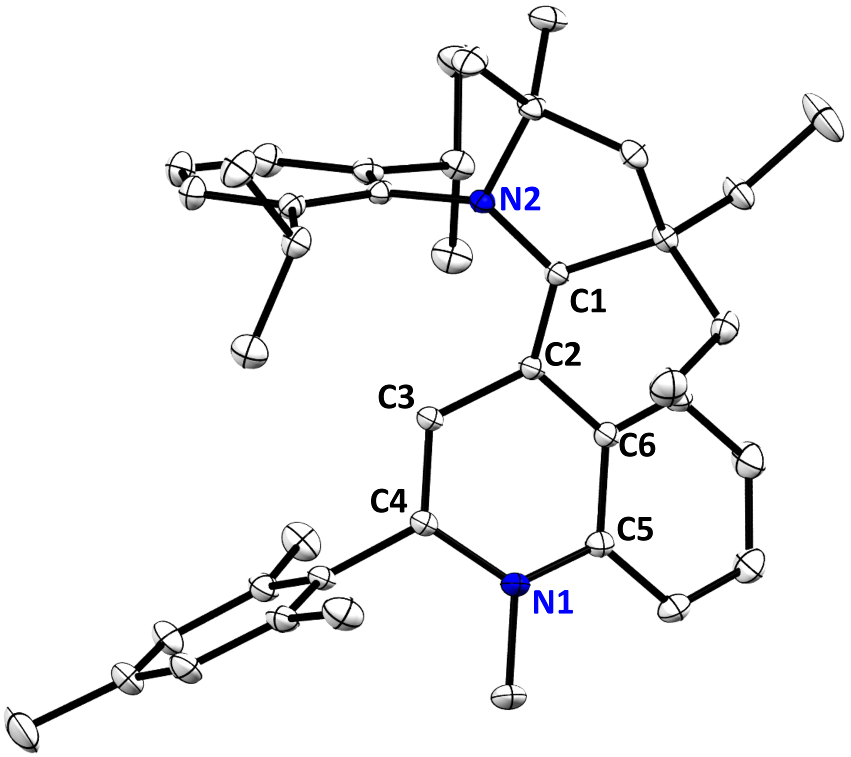

Figure S170: X-ray solid-state structure of ***E*-2g**. Hydrogen atoms were omitted for clarity. Thermal ellipsoids are shown with 50 % probability. Selected bond lengths [Å] and angles in [°]: N2–C1 1.4099(11), C1–C2 1.3735(11), C2–C3 1.4673(11), C3–C4 1.3504(12)C4– N1 1.4163(12), N1–C5 1.3987(11), C5–C6 1.4165(12), C6–C2 1.4762(12), N2–C1–C2–C3 −15.86(13).

Z-2g

A colourless needle-shaped crystal was mounted on a MiTeGen micromount with perfluoroether oil. Data for cb361_twin were collected from a shock-cooled single crystal at 100(2) K on a Bruker D8 VENTURE dual wavelength Ag/Cu four-circle diffractometer with a microfocus sealed X-ray tube using a mirror optics as monochromator and a Bruker PHOTON III detector. The diffractometer used CuK_α radiation (λ = 1.54178 Å). All data were integrated with SAINT V8.40B and a multi-scan absorption correction using SADABS 2016/2 was applied.[25,26] The structure was solved by direct methods with SHELXT 2018/2 and refined by full-matrix least-squares methods against F^2 using SHELXL-2019/2.[27,28] All non-hydrogen atoms were refined with anisotropic displacement parameters. All hydrogen atoms were refined isotropic on calculated positions using a riding model with their U_{iso} values constrained to 1.5 times the U_{eq} of their pivot atoms for terminal sp^3 carbon atoms and 1.2 times for all other carbon atoms. Disordered moieties were refined using bond lengths restraints and displacement parameter restraints. Crystallographic data for the structures reported in this paper have been deposited with the Cambridge Crystallographic Data Centre.[29] CCDC 2535137 contain the supplementary crystallographic data for this paper. These data can be obtained free of charge from The Cambridge Crystallographic Data Centre via www.ccdc.cam.ac.uk/structures. This report and the CIF file were generated using FinalCif.[30]

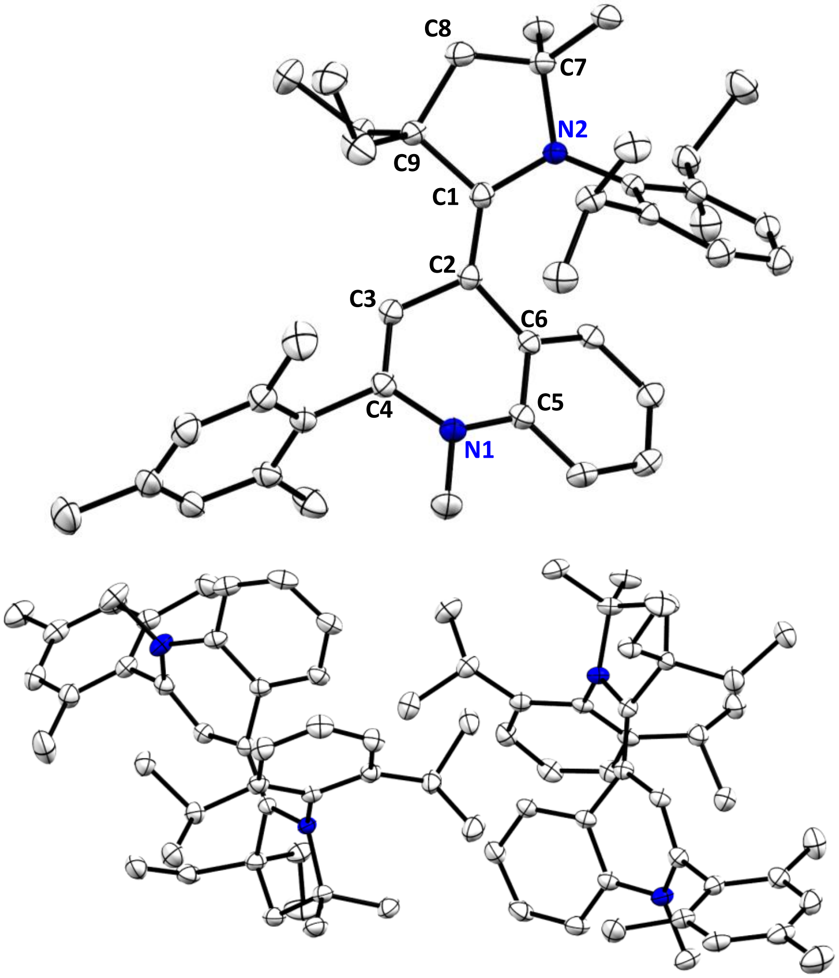

Figure S171: X-ray solid-state structure of ***Z*-2g**. Hydrogen atoms were omitted for clarity. Thermal ellipsoids are shown with 50 % probability. Selected bond lengths [Å] and angles in [°]: N2_1–C1_1 1.420(1) / N2_2–C1_2 1.421(1), C1_1–C2_1 1.3717(15)/ C1_2–C2_2 1.3709(15),C2_1–C3_1 1.4695(15)/ C2_2–C3_2 1.4702(15), C3_1–C4_1 1.3416(16)/ C3_2–C4_2 1.3403(16), C4_1– N1_1 1.4096(15) / C4_2– N1_2 1.4070(15), N1_1–C5_1 1.3977(15)/ N1_2–C5_2 1.3987(15), C5_1–C6_1 1.4186(16)/ C5_2–C6_2 1.4188(15), C6_1–C2_1 1.4832(15)/ C6_2–C2_2 1.4840(15), C9_1–C1_1–C2_1–C3_1 8.00(16)/ C9_2–C1_2–C2_2–C3_2 −6.52(17).

3g

A violet, plate-shaped crystal was mounted on a MiTeGen micromount with perfluoroether oil. Data for cb258te were collected from a shock-cooled single crystal at 100(2) K on a Bruker D8 VENTURE dual wavelength Mo/Cu four-circle diffractometer with a microfocus sealed X-ray tube using a mirror optics as monochromator and a Bruker PHOTON II detector. The diffractometer used MoK_{α} radiation (λ = 0.71073 Å). All data were integrated with SAINT V8.40B and a multi-scan absorption correction using SADABS 2016/2 was applied.[25,26] The structure was solved by direct methods with SHELXT 2018/2 and refined by full-matrix least-squares methods against F^2 using SHELXL-2019/2.[27,28] All non-hydrogen atoms were refined with anisotropic displacement parameters. All C-bound hydrogen atoms were refined isotropic on calculated positions using a riding model with their U_{iso} values constrained to 1.5 times the U_{eq} of their pivot atoms for terminal sp^3 carbon atoms and 1.2 times for all other carbon atoms. Disordered moieties were refined using bond lengths restraints and displacement parameter restraints. Crystallographic data for the structures reported in this paper have been deposited with the Cambridge Crystallographic Data Centre.[29] CCDC 2535138 contain the supplementary crystallographic data for this paper. These data can be obtained free of charge from The Cambridge Crystallographic Data Centre via www.ccdc.cam.ac.uk/structures. This report and the CIF file were generated using FinalCif.[30]

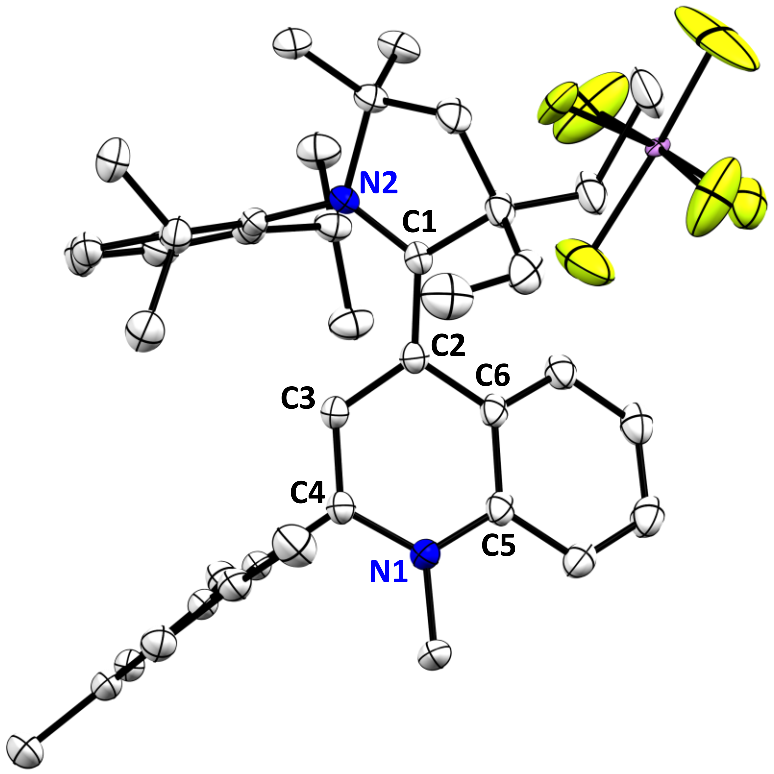

Figure S172: X-ray solid-state structure of **3g**. Hydrogen atoms were omitted for clarity as well as one thf molecule. Thermal ellipsoids are shown with 50 % probability. Selected bond lengths [Å] and angles in [°]: N2–C1 1.345(3), C1–C2 1.440(3), C2–C3 1.412(3), C3–C4 1.369(3), C4–N1 1.376(3), N1–C5 1.398(3), C5–C6 1.414(3), C6–C2 1.456(3), N2–C1–C2–C3 –36.8(3).

***E*-2h**

A red, plate-shaped crystal was mounted on a MiTeGen micromount with perfluoroether oil. The data for scs4_90ep-finalcif.cif were collected from a shock-cooled single crystal at 100(2) K on a Bruker D8 VENTURE, Cu four-circle diffractometer with a microfocus sealed X-ray tube using mirror optics as monochromator and a Bruker PHOTON III 14 detector. The diffractometer was equipped with a low temperature device and used CuK_α radiation (λ = 1.54178 Å). All data were integrated with SAINT and a multi-scan absorption correction using SADABS-2016/2was applied.[25,26] The structure was solved by direct methods with SHELXT 2018/2 and refined by full-matrix least-squares methods against F^2 using SHELXL-2019/2.[27,28] All non-hydrogen atoms were refined with anisotropic displacement parameters. All hydrogen atoms were refined isotropic on calculated positions using a riding model with their U_{iso} values constrained to 1.5 times the U_{eq} of their pivot atoms for terminal sp^3 carbon atoms and 1.2 times for all other carbon atoms. Disordered moieties were refined using bond lengths restraints and displacement parameter restraints. Crystallographic data for the structures reported in this paper have been deposited with the Cambridge Crystallographic Data Centre.[29] CCDC 2535139 contain the supplementary crystallographic data for this paper. These data can be obtained free of charge from The Cambridge Crystallographic Data Centre via www.ccdc.cam.ac.uk/ structures. This report and the CIF file were generated using FinalCif.[30]

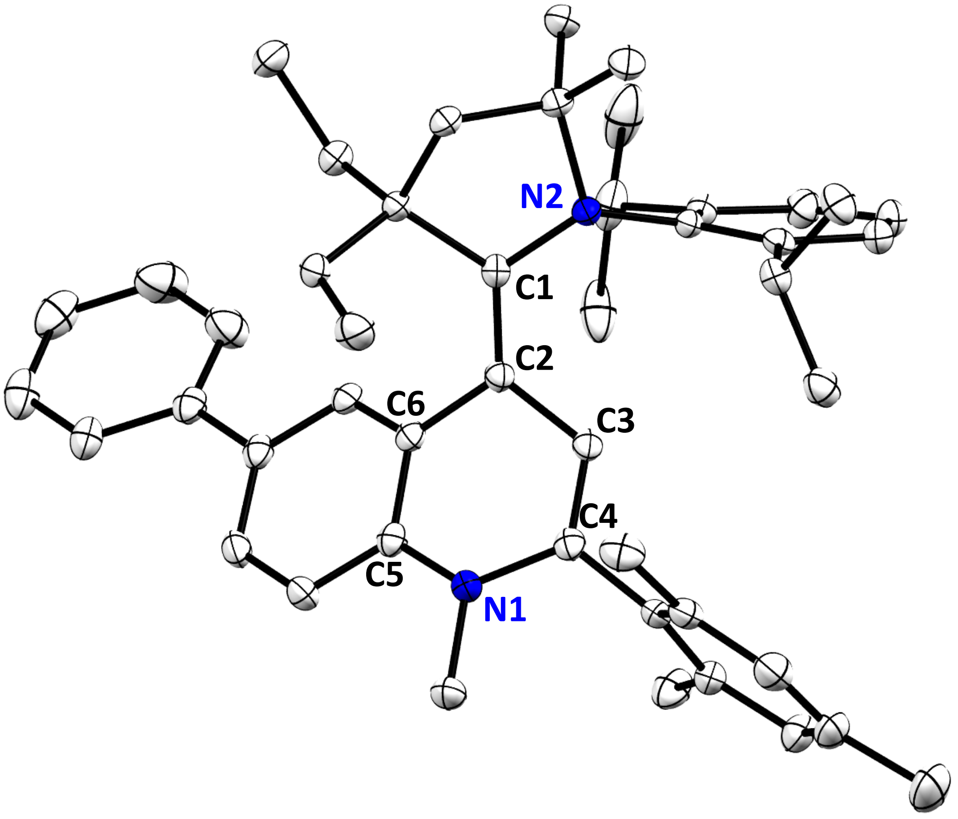

Figure S173: X-ray solid-state structure of ***E*-2h**. Hydrogen atoms were omitted for clarity. Thermal ellipsoids are shown with 50 % probability. Selected bond lengths [Å] and angles in [°]: N2–C1 1.420(2), C1–C2 1.364(2), C2–C3 1.462(2), C3–C4 1.345(2), C4– N1 1.412(2), N1–C5 1.398(2), C5–C6 1.414(2), C6–C2 1.474(2), N2–C1–C2–C3 17.5(3).

***E*-2i**

A yellow, block-shaped crystal was mounted on a MiTeGen micromount with perfluoroether oil. Data for cb326b were collected from a shock-cooled single crystal at 100(2) K on a Bruker D8 VENTURE dual wavelength Ag/Cu four-circle diffractometer with a microfocus sealed X-ray tube using a mirror optics as monochromator and a Bruker PHOTON III detector. The diffractometer used CuK_α radiation (λ = 1.54178 Å). All data were integrated with SAINT V8.40B and a multi-scan absorption correction using SADABS 2016/2 was applied.[25,26] The structure was solved by direct methods with SHELXT 2018/2 and refined by full-matrix least-squares methods against F^2 using SHELXL-2019/2.[27,28] All non-hydrogen atoms were refined with anisotropic displacement parameters. All hydrogen atoms were refined with isotropic displacement parameters. Some of their coordinates were refined freely and some on calculated positions using a riding model with their U_{iso} values constrained to 1.5 times the U_{eq} of their pivot atoms for terminal sp^3 carbon atoms and 1.2 times for all other carbon atoms. Crystallographic data for the structures reported in this paper have been deposited with the Cambridge Crystallographic Data Centre.[29] CCDC 2535140 contain the supplementary crystallographic data for this paper. These data can be obtained free of charge from The Cambridge Crystallographic Data Centre via www.ccdc.cam.ac.uk/structures. This report and the CIF file were generated using FinalCif.[30]

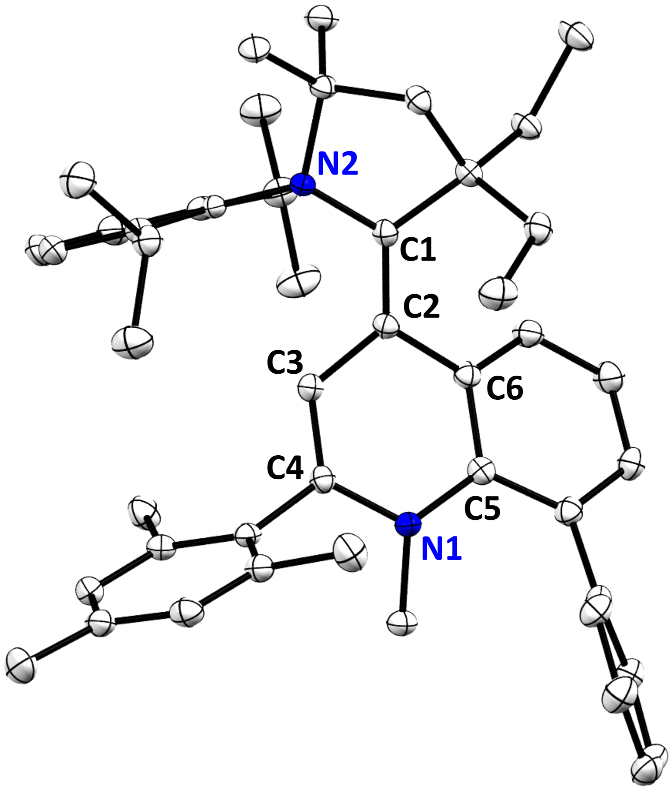

Figure S174: X-ray solid-state structure of ***E*-2i**. Hydrogen atoms were omitted for clarity. Thermal ellipsoids are shown with 50 % probability. Selected bond lengths [Å] and angles in [°]: N2–C1 1.4170(11), C1–C2 1.3743(13), C2–C3 1.4495(12), C3–C4 1.3450(13), C4– N1 1.4111(12), N1–C5 1.4093(12), C5–C6 1.4255(13), C6–C2 1.4738(12), N2–C1–C2–C3 –20.47 (13).

***E*-2j**

A orange, block-shaped crystal was mounted on a MiTeGen micromount with perfluoroether oil. Data for cb348_2 were collected from a shock-cooled single crystal at 100(2) K on a Bruker D8 VENTURE dual wavelength Ag/Cu four-circle diffractometer with a microfocus sealed X-ray tube using a mirror optics as monochromator and a Bruker PHOTON III detector. The diffractometer used AgK_{α} radiation (λ = 0.56086 Å). All data were integrated with SAINT V8.40B and a multi-scan absorption correction using SADABS 2016/2 was applied.[25,26] The structure was solved by direct methods with SHELXT 2018/2 and refined by full-matrix least-squares methods against F^2 using SHELXL-2019/2.[27,28] All non-hydrogen atoms were refined with anisotropic displacement parameters. All hydrogen atoms were refined isotropic on calculated positions using a riding model with their U_{iso} values constrained to 1.5 times the U_{eq} of their pivot atoms for terminal sp^3 carbon atoms and 1.2 times for all other carbon atoms. Crystallographic data for the structures reported in this paper have been deposited with the Cambridge Crystallographic Data Centre.[29] CCDC 2535141 contain the supplementary crystallographic data for this paper. These data can be obtained free of charge from The Cambridge Crystallographic Data Centre via www.ccdc.cam.ac.uk/structures. This report and the CIF file were generated using FinalCif.[30]

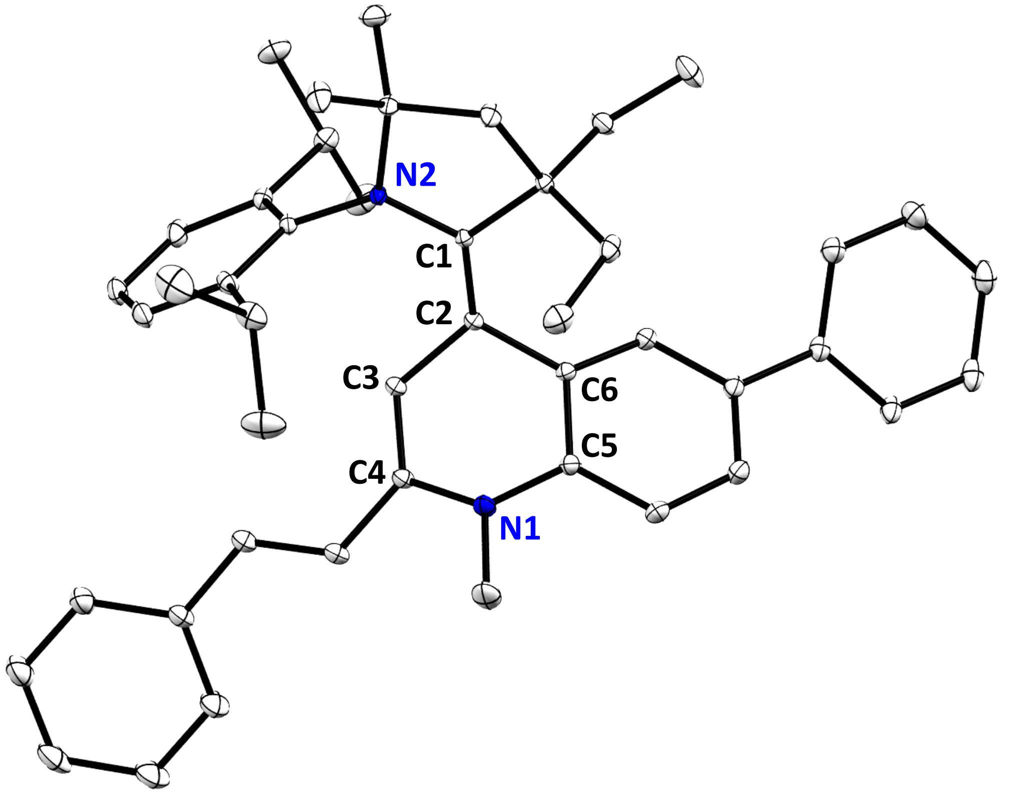

Figure S175: X-ray solid-state structure of ***E*-2j**. Hydrogen atoms were omitted for clarity. Thermal ellipsoids are shown with 50 % probability. Selected bond lengths [Å] and angles in [°]: N2–C1 1.3951(8), C1–C2 1.3742(8), C2–C3 1.4809(9), C3–C4 1.3563(9), C4– N1 1.4156(9), N1–C5 1.3928(9), C5–C6 1.4158(9), C6–C2 1.4932(9), N2–C1–C2–C3 −8.92(11).

	**2a**	**4a**	**2c**
CIF ID	mo_0852_CG_0m	mo_0899_CG_0m_4	cb932e
CCDC number	2535132	2535133	2535134
Empirical formula	$C_{41}H_{45}N_3$	$C_{45}H_{53}F_{12}N_3OSb_2$	$C_{51}H_{59}N_3O$
Formula weight	579.80	1123.40	730.01
Temperature [K]	100(2)	100(2)	100(2)
Crystal system	orthorhombic	triclinic	monoclinic
Space group (number)	$Pbca$ (61)	$P\overline{1}$ (2)	$C2/c$ (15)
a [Å]	15.427(4)	16.226(3)	33.779(16)
b [Å]	20.571(4)	17.265(3)	13.363(6)
c [Å]	21.180(7)	18.580(3)	18.746(9)
α [°]	90	111.741(4)	90
β [°]	90	100.071(5)	97.739(10)
γ [°]	90	94.618(5)	90
Volume [$Å^3$]	6722(3)	4700.0(14)	8385(7)
Z	8	4	8
ρ_{calc} [gcm^{-3}]	1.146	1.588	1.157
μ [mm^{-1}]	0.066	1.234	0.068
F(000)	2496	2248	3152
Crystal size [mm^3]	0.370×0.174×0.108	0.319×0.166×0.038	0.274×0.132×0.060
Crystal colour	red	colourless	orange
Crystal shape	plate	plate	plate
Radiation	MoK_α (λ=0.71073 Å)	MoK_α (λ=0.71073 Å)	MoK_α (λ=0.71073 Å)
2Θ range [°]	4.67 to 57.42 (0.74 Å)	4.17 to 54.35 (0.78 Å)	4.39 to 59.31 (0.72 Å)
Index ranges	-20 ≤ h ≤ 20<br>-27 ≤ k ≤ 27<br>-28 ≤ l ≤ 28	-20 ≤ h ≤ 20<br>-22 ≤ k ≤ 22<br>-23 ≤ l ≤ 23	-46 ≤ h ≤ 46<br>-18 ≤ k ≤ 18<br>-25 ≤ l ≤ 26
Reflections collected	260915	36940	213774
Independent reflections	8681<br>R_{int} = 0.0589<br>R_{sigma} = 0.0162	36940<br>R_{int} = 0.052<br>R_{sigma} = 0.0535	11804<br>R_{int} = 0.0425<br>R_{sigma} = 0.0173
Completeness	99.9 %	96.4 %	99.9 %
Data / Restraints / Parameters	8681/0/407	36940/1824/1156	11804/906/555
Goodness-of-fit on F^2	1.023	1.055	1.063
Final *R* indexes [$I \geq 2\sigma(I)$]	R_1 = 0.0415<br>wR_2 = 0.1013	R_1 = 0.0725<br>wR_2 = 0.1809	R_1 = 0.0511<br>wR_2 = 0.1482
Final *R* indexes [all data]	R_1 = 0.0532<br>wR_2 = 0.1102	R_1 = 0.0902<br>wR_2 = 0.1942	R_1 = 0.0607<br>wR_2 = 0.1571
Largest peak/hole [$eÅ^{-3}$]	0.32/-0.22	2.11/-3.13	0.74/-0.57

	***E*-2f**	***E*-2g**	***Z*-2g**
CIF ID	cblz07b	cb86	Cb361_twin
CCDC number	2535135	2535136	2535137
Empirical formula	$C_{32}H_{36}Br_2N_2$	$C_{41}H_{54}N_2$	$C_{41}H_{54}N_2$
Formula weight	608.45	574.86	574.86
Temperature [K]	100(2)	100(2)	100(2)
Crystal system	monoclinic	triclinic	monoclinic
Space group (number)	$P2_1/n$ (14)	$P\overline{1}$ (2)	$P2_1/n$ (14)
a [Å]	7.5144(3)	8.9963(4)	9.7193(2)
b [Å]	30.3300(13)	12.0588(7)	30.3393(5)
c [Å]	12.3615(6)	16.4980(9)	23.3329(4)
α [°]	90	99.424(2)	90
β [°]	92.322(2)	101.202(2)	98.3010(10)
γ [°]	90	95.423(2)	90
Volume [Å^3]	2815.0(2)	1717.17(16)	6808.2(2)
Z	4	2	8
ρ_{calc} [gcm^{-3}]	1.436	1.112	1.122
μ [mm^{-1}]	2.903	0.063	0.478
F(000)	1248	628	2512
Crystal size [mm^3]	0.500×0.203×0.138	0.500×0.300×0.200	0.100×0.050×0.050
Crystal colour	red	yellow	colourless
Crystal shape	block	block	needle
Radiation	MoK_α (λ=0.71073 Å)	MoK_α (λ=0.71073 Å)	CuK_α (λ=1.54178 Å)
2Θ range [°]	5.21 to 55.33 (0.77 Å)	5.77 to 66.28 (0.65 Å)	4.81 to 140.30 (0.82 Å)
Index ranges	-9 ≤ h ≤ 9<br>-39 ≤ k ≤ 39<br>-16 ≤ l ≤ 16	-13 ≤ h ≤ 13<br>-18 ≤ k ≤ 18<br>-25 ≤ l ≤ 25	-10 ≤ h ≤ 11<br>-36 ≤ k ≤ 37<br>-28 ≤ l ≤ 28
Reflections collected	49505	141303	143500
Independent reflections	6560<br>R_{int} = 0.0611<br>R_{sigma} = 0.0346	12598<br>R_{int} = 0.0990<br>R_{sigma} = 0.0592	12920<br>R_{int} = 0.0375<br>R_{sigma} = 0.0144
Completeness	99.9 %	96.0 %	100.0 %
Data / Restraints / Parameters	6560/0/333	12598/0/400	12920/1312/810
Goodness-of-fit on F^2	1.064	1.056	1.016
Final *R* indexes [$I \geq 2\sigma(I)$]	R_1 = 0.0338<br>wR_2 = 0.0783	R_1 = 0.0485<br>wR_2 = 0.1295	R_1 = 0.0389<br>wR_2 = 0.0962
Final *R* indexes [all data]	R_1 = 0.0412<br>wR_2 = 0.0813	R_1 = 0.0786<br>wR_2 = 0.1430	R_1 = 0.0431<br>wR_2 = 0.0995
Largest peak/hole [eÅ^{-3}]	0.52/-0.49	0.63/-0.31	0.21/-0.23

	**3g**	***E*-2h**	***E*-2i**
CIF ID	cb258te	scs4_90ep	cb326b
CCDC number	2535138	2535139	2535140
Empirical formula	$C_{45}H_{62}F_6N_2OSb$	$C_{47}H_{58}N_2$	$C_{47}H_{58}N_2$
Formula weight	882.71	650.95	650.95
Temperature [K]	100(2)	100(2)	100(2)
Crystal system	triclinic	triclinic	monoclinic
Space group (number)	$P\overline{1}$ (2)	$P\overline{1}$ (2)	$P2_1/n$ (14)
a [Å]	11.6062(9)	11.5784(7)	11.4674(3)
b [Å]	12.9842(11)	12.5446(8)	13.2369(3)
c [Å]	14.4227(12)	13.7332(8)	24.9497(6)
α [°]	81.244(3)	103.318(3)	90
β [°]	87.306(3)	92.428(4)	93.3010(10)
γ [°]	86.077(3)	95.162(3)	90
Volume [Å^3]	2141.6(3)	1929.1(2)	3780.90(16)
Z	2	2	4
ρ_{calc} [gcm^{-3}]	1.369	1.121	1.144
μ [mm^{-1}]	0.706	0.478	0.488
F(000)	918	708	1416
Crystal size [mm^3]	0.100×0.100×0.020	0.120×0.100×0.030	0.170×0.080×0.050
Crystal colour	violet	red	yellow
Crystal shape	plate	plate	block
Radiation	MoK_α (λ=0.71073 Å)	CuK_α (λ=1.54178 Å)	CuK_α (λ=1.54178 Å)
2Θ range [°]	5.72 to 56.90 (0.75 Å)	6.63 to 133.18 (0.84 Å)	7.10 to 149.04 (0.80 Å)
Index ranges	-15 ≤ h ≤ 15<br>-17 ≤ k ≤ 17<br>-19 ≤ l ≤ 19	-13 ≤ h ≤ 13<br>-14 ≤ k ≤ 12<br>-16 ≤ l ≤ 16	-14 ≤ h ≤ 14<br>-16 ≤ k ≤ 15<br>-31 ≤ l ≤ 30
Reflections collected	88294	60779	94889
Independent reflections	10702<br>R_{int} = 0.0533<br>R_{sigma} = 0.0319	6790<br>R_{int} = 0.0955<br>R_{sigma} = 0.0442	7733<br>R_{int} = 0.0246<br>R_{sigma} = 0.0113
Completeness	99.8 %	99.7 %	100.0 %
Data / Restraints / Parameters	10702/1223/629	6790/258/494	7733/0/458
Goodness-of-fit on F^2	1.140	1.121	1.038
Final *R* indexes<br>[$I \geq 2\sigma(I)$]	R_1 = 0.0412<br>wR_2 = 0.0785	R_1 = 0.0553<br>wR_2 = 0.1202	R_1 = 0.0357<br>wR_2 = 0.0876
Final *R* indexes<br>[all data]	R_1 = 0.0511<br>wR_2 = 0.0822	R_1 = 0.0634<br>wR_2 = 0.1244	R_1 = 0.0365<br>wR_2 = 0.0882
Largest peak/hole [eÅ^{-3}]	0.50/-0.83	0.21/-0.25	0.26/-0.20

	***E*-2j**
CIF ID	Cb348_2
CCDC number	2535141
Empirical formula	$C_{46}H_{54}N_2$
Formula weight	634.91
Temperature [K]	100(2)
Crystal system	triclinic
Space group (number)	$P\overline{1}$ (2)
a [Å]	8.7457(4)
b [Å]	10.5550(5)
c [Å]	20.9805(10)
α [°]	104.191(2)
β [°]	100.080(2)
γ [°]	92.908(2)
Volume [Å^3]	1839.73(15)
Z	2
ρ_{calc} [gcm^{-3}]	1.146
μ [mm^{-1}]	0.043
F(000)	688
Crystal size [mm^3]	0.250×0.200×0.100
Crystal colour	orange
Crystal shape	block
Radiation	AgK_α (λ=0.56086 Å)
2Θ range [°]	3.78 to 68.29 (0.50 Å)
Index ranges	-17 ≤ h ≤ 17<br>-21 ≤ k ≤ 21<br>-41 ≤ l ≤ 41
Reflections collected	280076
Independent reflections	30812<br>R_{int} = 0.0730<br>R_{sigma} = 0.0433
Completeness	99.8 %
Data / Restraints / Parameters	30812/0/442
Goodness-of-fit on F^2	1.154
Final *R* indexes<br>[$I \geq 2\sigma(I)$]	R_1 = 0.0760<br>wR_2 = 0.1572
Final *R* indexes<br>[all data]	R_1 = 0.0959<br>wR_2 = 0.1657
Largest peak/hole [eÅ^{-3}]	0.75/-0.36

Isomerization and Switching Experiments

Setup

As a light source one or two Kessil PR160 LED lamps with the wavelength indicated were used. To keep the sample at room temperature a fan was used.

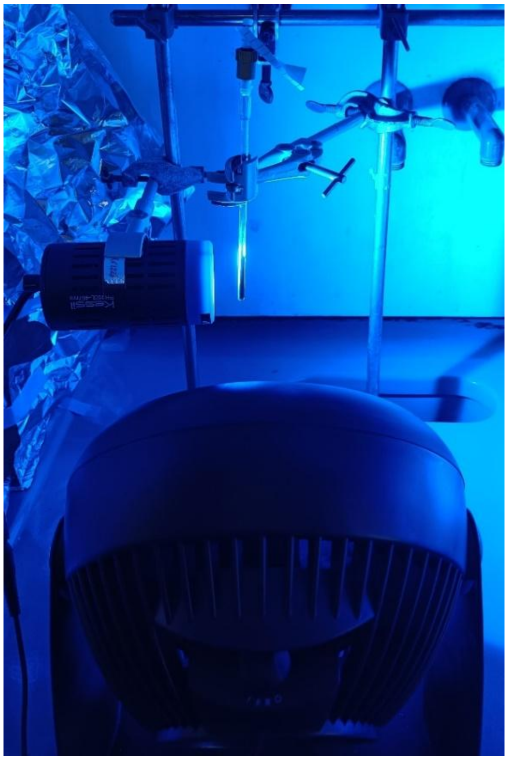

Figure S176: Irradiation setup used for the isomerization inside the J-Young NMR tube.

Wavelength dependency

Procedure: Switch (20 µmol, 1 equiv) was dissolved in C_6D_6 (0.5 mL). Then 1,3,5-trimethoxybenzene (6.6 µmol, 0.33 equiv) as an internal reference was added. The solution was then transferred into a J. Young NMR-tube. A ^{1}H-NMR spectrum was measured to determine the thermodynamic equilibrium of the *E*:*Z* ratio. Now the sample was irradiated for 1 hour as described above with the wavelength indicated utilizing Kessil led lamps. Note: 1 hour irradiation time was chosen to ensure that the PSS for every system was reached. Afterwards a ^{1}H-NMR spectrum was measured to determine the change in the *E*:*Z* ratio. To go back to the thermodynamic ratio the sample was heated at 80 °C for the time stated below. A ^{1}H-NMR spectrum was measured for verification. Now the sample was irradiated with a different wavelength repeating the described procedure.

For system **2h** 1.0 equiv of KHMDS had to be added as otherwise only broad signals were observed. Thus, the sample was not heated to switch back to the thermodynamic ratio but irradiated with 370 nm for 1 h.

Table S1: Time at 80 °C until thermodynamic ratio is reached. a) 1 equiv KHMDS added.

	**2g**	**2h**	**2i**	**2j**
t	1 h	–	1 h	30 min[a]

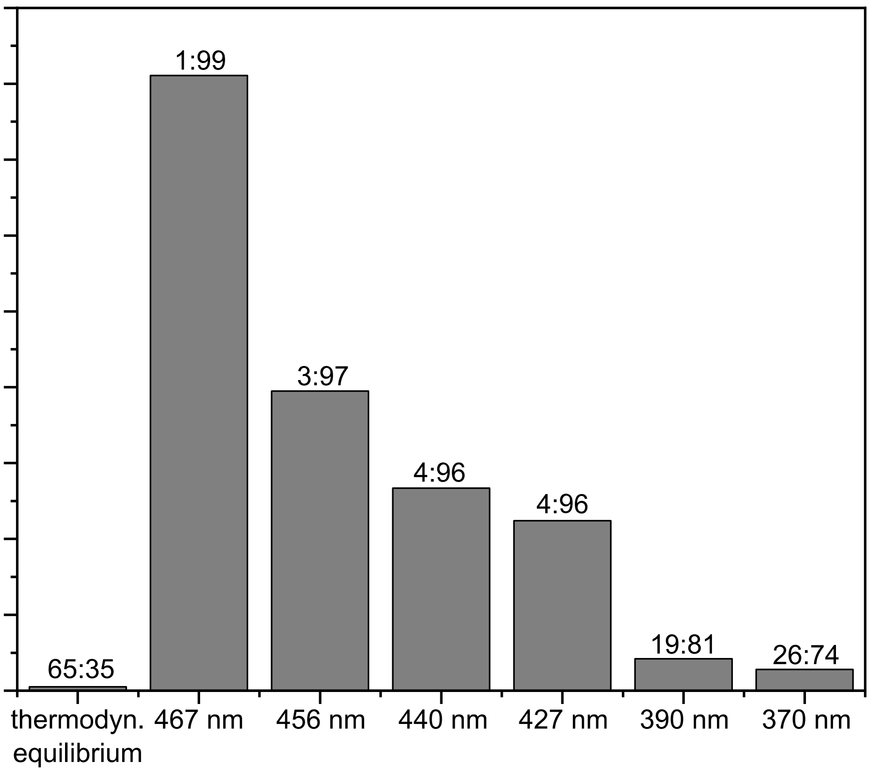

Figure S177: *E*:*Z* ratio after 1 h irradiation with the indicated wavelength for **2g**. Starting from the thermodynamic equilibrium.

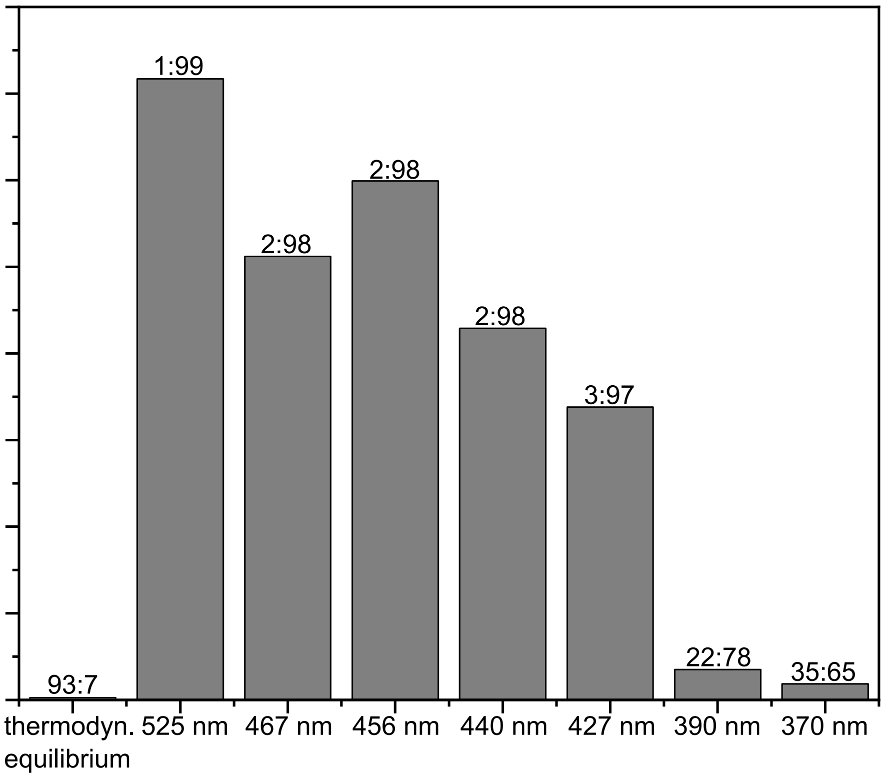

Figure S178: *E*:*Z* ratio after 1 h irradiation with the indicated wavelength for **2h**. Starting from an E:Z ratio of 36:64 generated by irradiating the sample for 1 h at 370 nm.

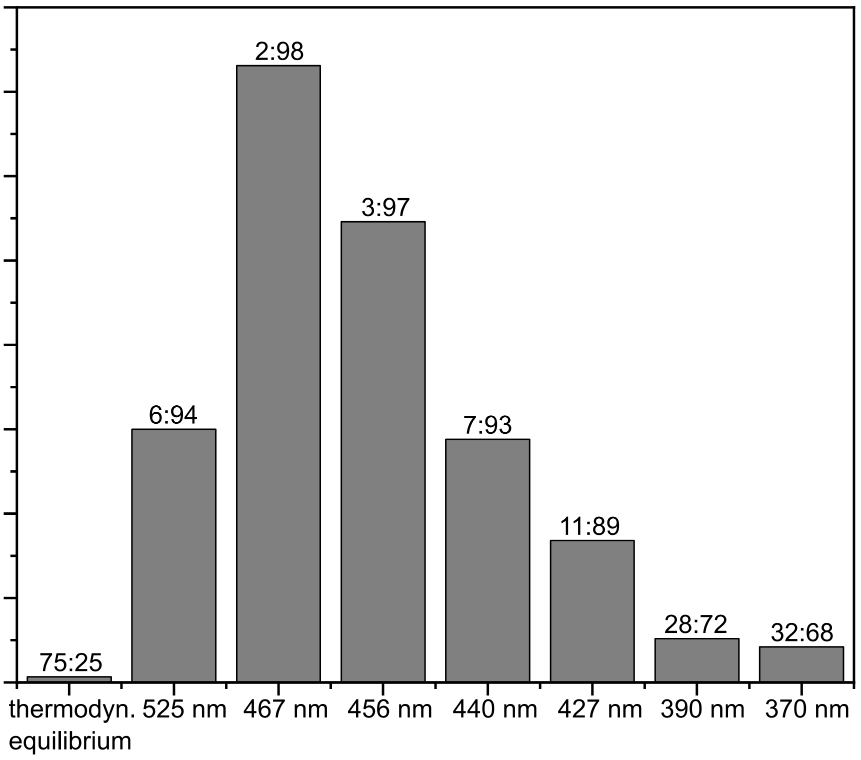

Figure S179: *E*:*Z* ratio after 1 h irradiation with the indicated wavelength for **2i**. Starting from the thermodynamic equilibrium.

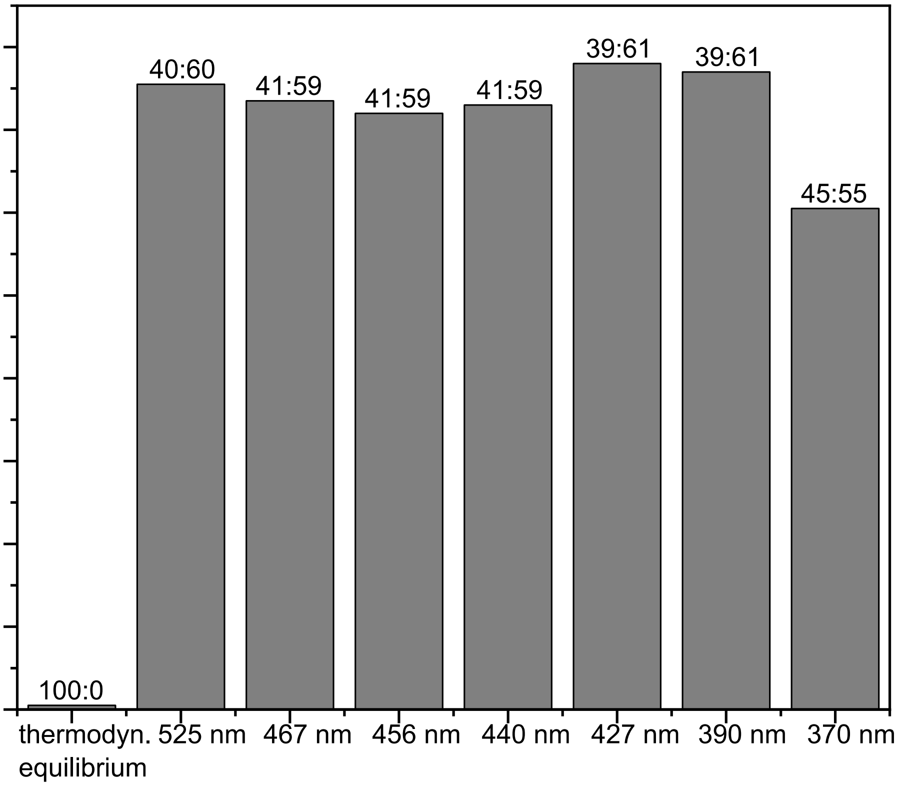

Figure S180: *E*:*Z* ratio after 1 h irradiation with the indicated wavelength for **2j**. Starting from the thermodynamic equilibrium.

To investigate how long each switch takes to reach the photo-stationary sate (PSS) ^{1}H-NMR were taken over time while irradiating the sample.

Table S2: Time until the PSS is reached at given wavelength.

	**2g**	**2h**	**2i**	**2j**
t	10 min (467 nm)	3 min (525 nm)	3 min (467 nm)	50 min (427 nm)

Control experiments

To verify that 1,3,5-trimethoxybenzene as the internal standard has no influence on the isomerization behaviour of the hybrids system **2g** was irradiated with and without the addition of 1,3,5-trimethoxybenzene. The *E*:*Z* ratio was in both cases the identical ruling out influence of the internal standard.

Half-life of the metastable *Z*-Isomer

To evaluate the half-life of the metastable *Z*-isomer at room temperature a NMR sample was prepared as described above. After irradiation at the given wavelength for 1 hour multiple ^{1}H-NMR spectra were recorded over time until the thermodynamic equilibrium was reached. In all cases the back isomerization was determined to be mono exponential.

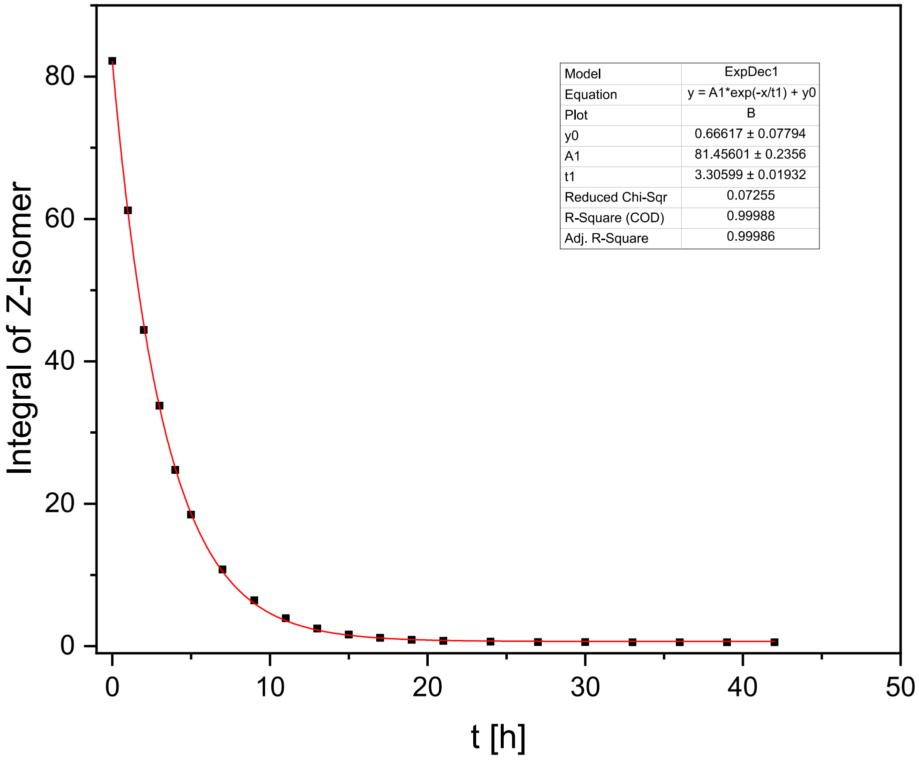

Figure S181: Back switching of the metastable *Z*-isomer of **2g** at room temperature after irradiation for 15 minutes at 467 nm. $t_{/2}$ = 2.3 h

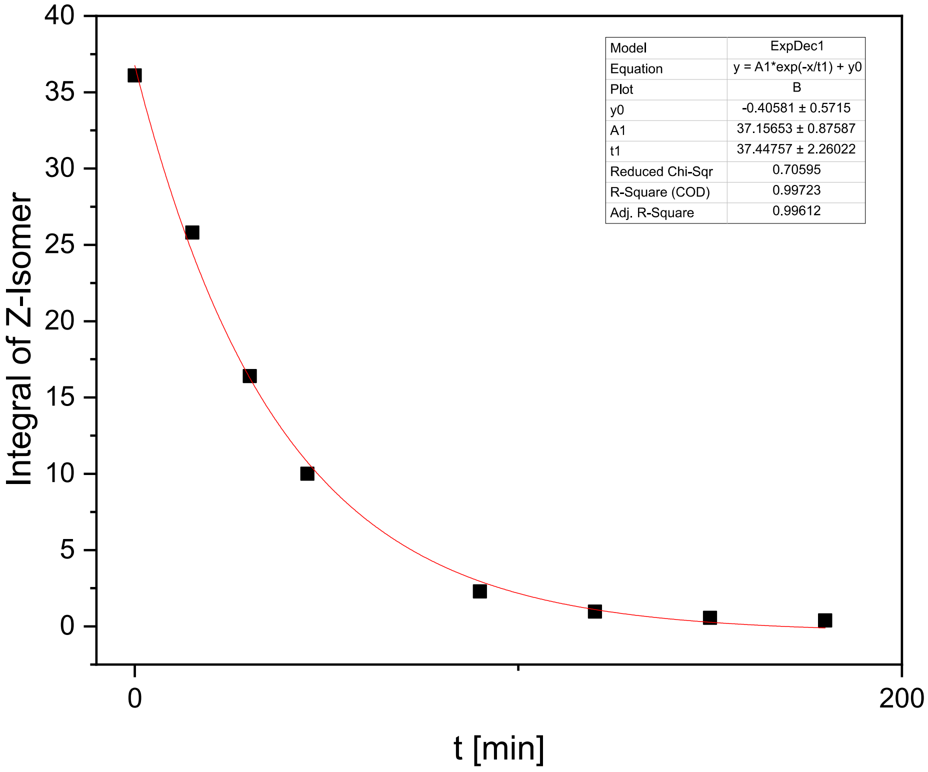

Figure S182: Back switching of the metastable *Z*-isomer of **2i** at room temperature after irradiation for 10 minutes at 467 nm. $t_{/2}$ = 26 min

Table S3: Half-life of the metastable *Z*-isomers.

	**2g**	**2h**	**2i**	**2j**
t	2.3 h	–	26 min	< 5 min

To investigate the influence of KHMDS on the half-live of the metastable state an NMR sample was prepared as described above but also 1.0 equiv. of KHMDS was added prior to irradiation. The half-life of the sample with KHMDS present is up to 57 times longer than without it.

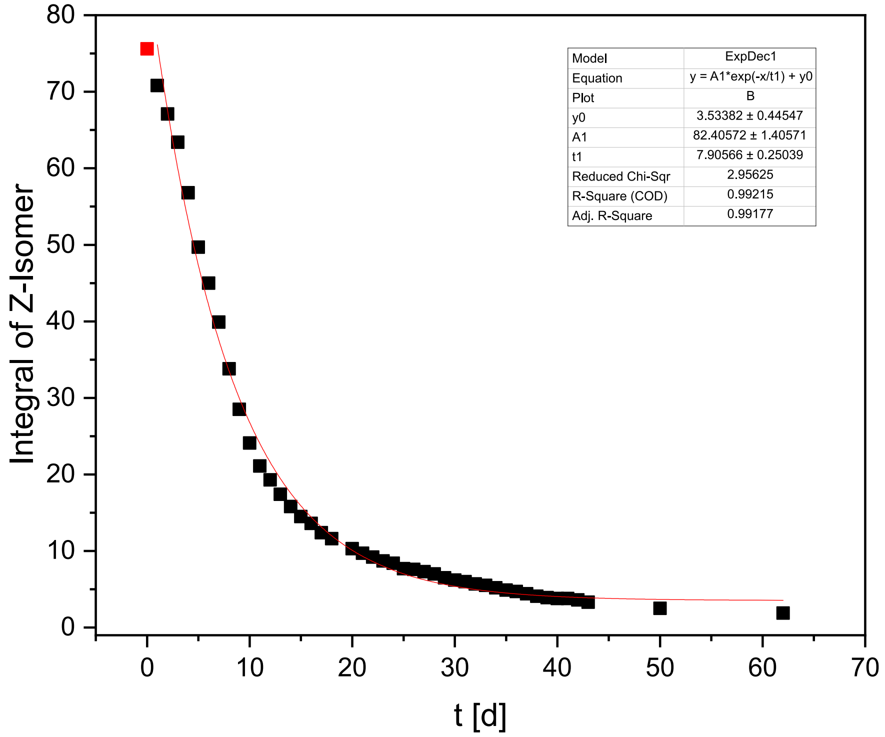

Figure S183: Back switching of the metastable *Z*-isomer of **2g** at room temperature after irradiation for 1 hour at 467 nm with 1.0 equiv. of KHMDS added. $t_{/2}$ = 5.5 d

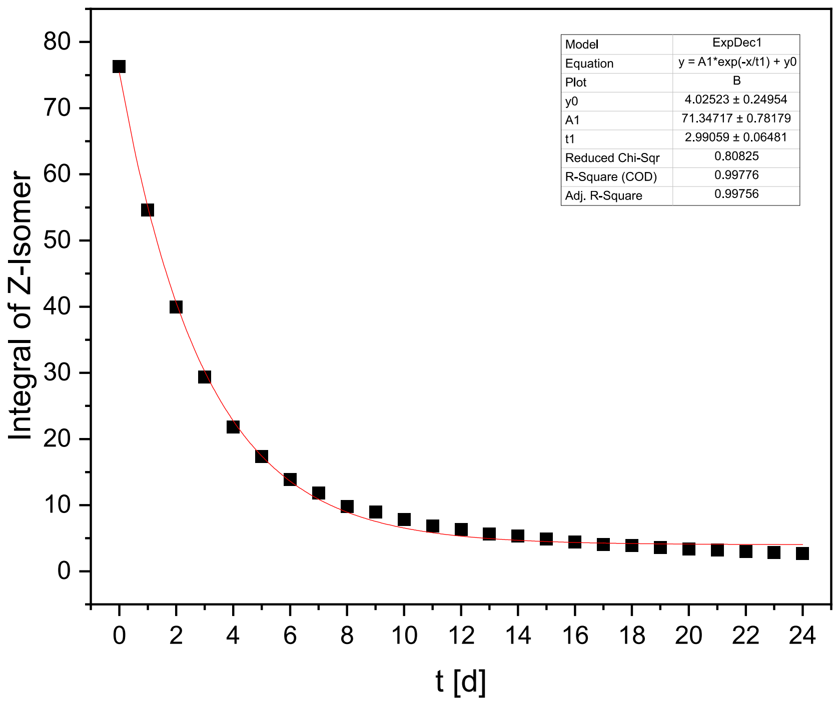

Figure S184: Back switching of the metastable *Z*-isomer of **2h** at room temperature after irradiation for 10 minutes at 525nm with 1.0 equiv of KHMDS added. $t_{/2}$ = 2.1 d

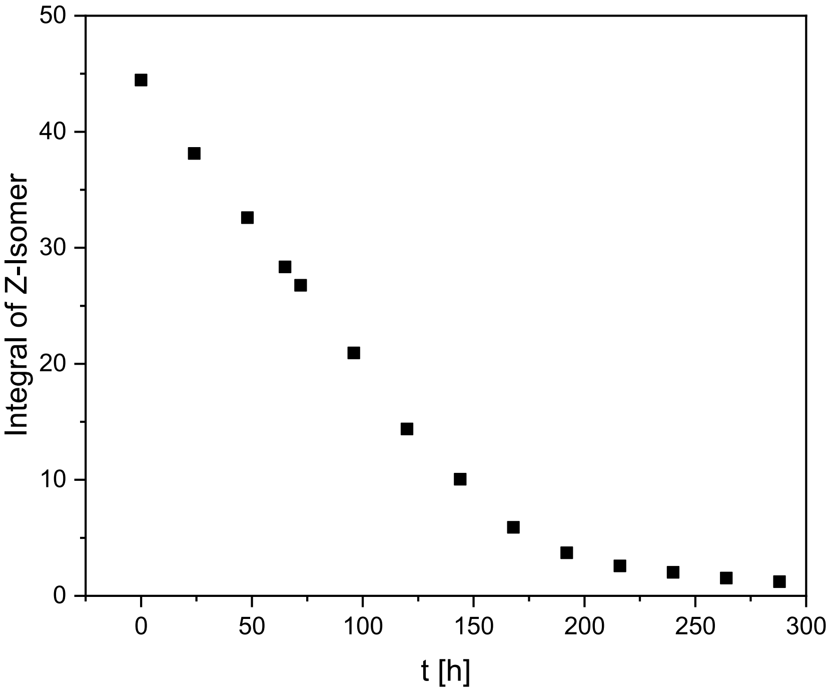

Figure S185: Back switching of the metastable *Z*-isomer of **2i** at room temperature after irradiation for 15 min at 467 nm with 1.0 equiv of KHMDS added. Note: It was not possible to find an exponential fit for the decay. $t_{1/2}$ is estimated to 2.5 d

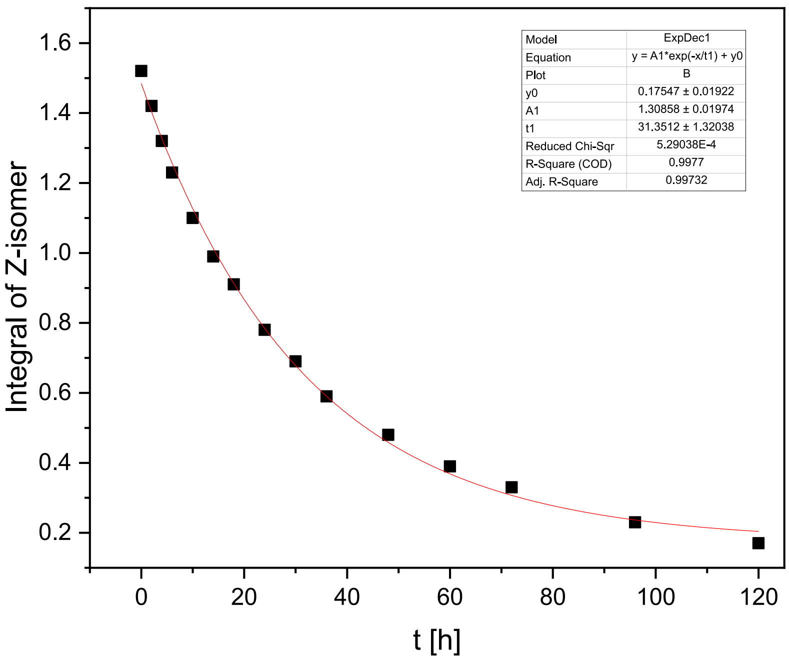

Figure S186: Back switching of the metastable *Z*-isomer of **2j** at room temperature after irradiation for 50 minutes at 427nm with 1.0 equiv of KHMDS added. $t_{/2}$ = 21.7 h.

Table S4: Half-life of the metastable *Z*-isomers with KHMDS present (1 equiv.).

	**2g**	**2h**	**2i**	**2j**
t	5.5 d (467 nm)	2.1 d (525 nm)	2.5 d (467 nm)	21.7 h (427 nm)

Light driven forth- and back-switching

To investigate the back and forth switching a sample was prepared as described above. First the isomeric mixture obtained from the synthesis was irradiated to reach the PSS. Then the sample was immediately irradiated with another wavelength to induce the back switching.

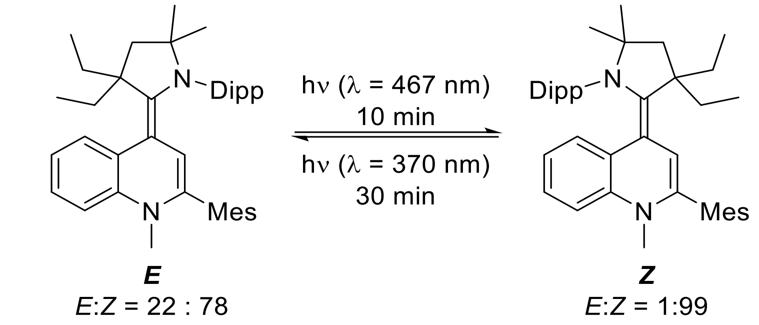

Scheme S1: Light induced back and forth switching of system **2g**.

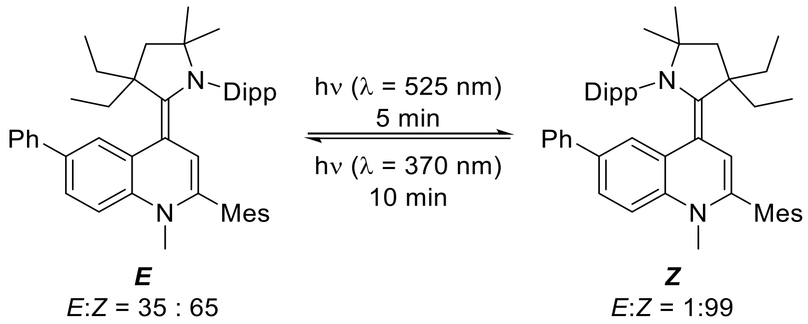

Scheme S2: Light induced back and forth switching of system **2h**.

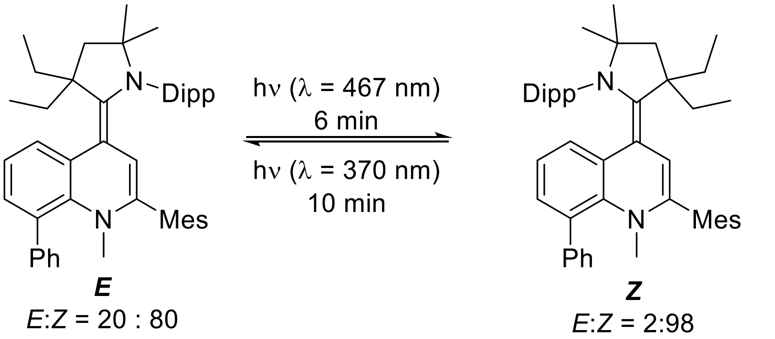

Scheme S3: Light induced back and forth switching of system **2i**.

UV-vis switching studies

Procedure: A UV-vis in benzene or thf was measured before the irradiation. Here for a 1 cm Teflon capped J. Young UV-vis cuvette was used. For the irradiation a Kessil lamp with the given wavelength was used.

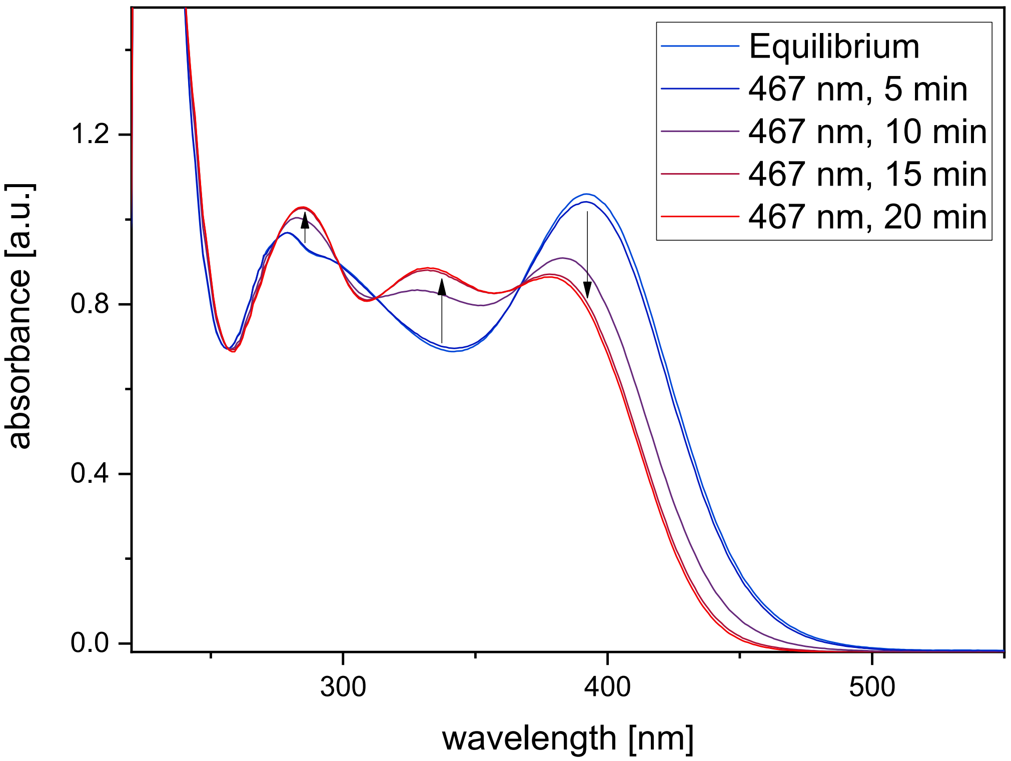

Figure S187: UV-vis spectra of **2g** in its thermodynamic equilibrium (blue; *E*:*Z* = 65:35) and after irradiation with 467 nm over time (red; after 20 min: *E*:*Z* = 1:99, PSS) in thf.

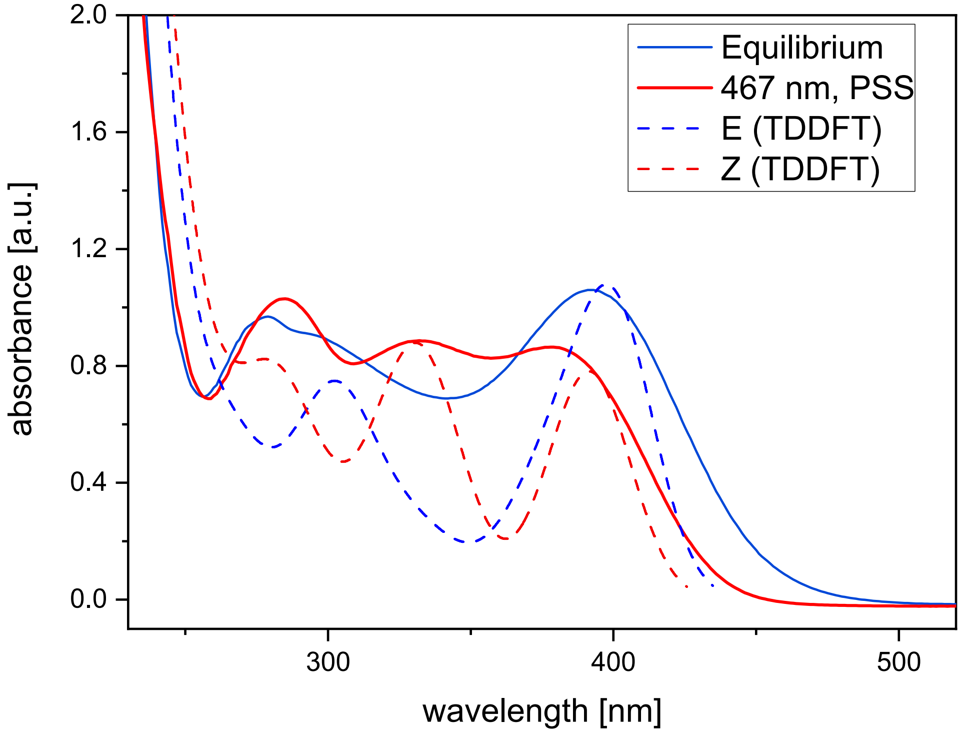

Figure S188: UV-vis spectra of **2g** in its thermodynamic equilibrium (blue; *E*:*Z* = 65:35) and after irradiation with 467 nm for 20 minutes (red; *E*:*Z* = 1:99, PSS) in thf compared with the calculated TDDFT spectra for the *E*- and *Z*-isomer in thf. TDDFT spectra are corrected by 1.12 as a scalar factor according to FEHÉR *et al.*[31]

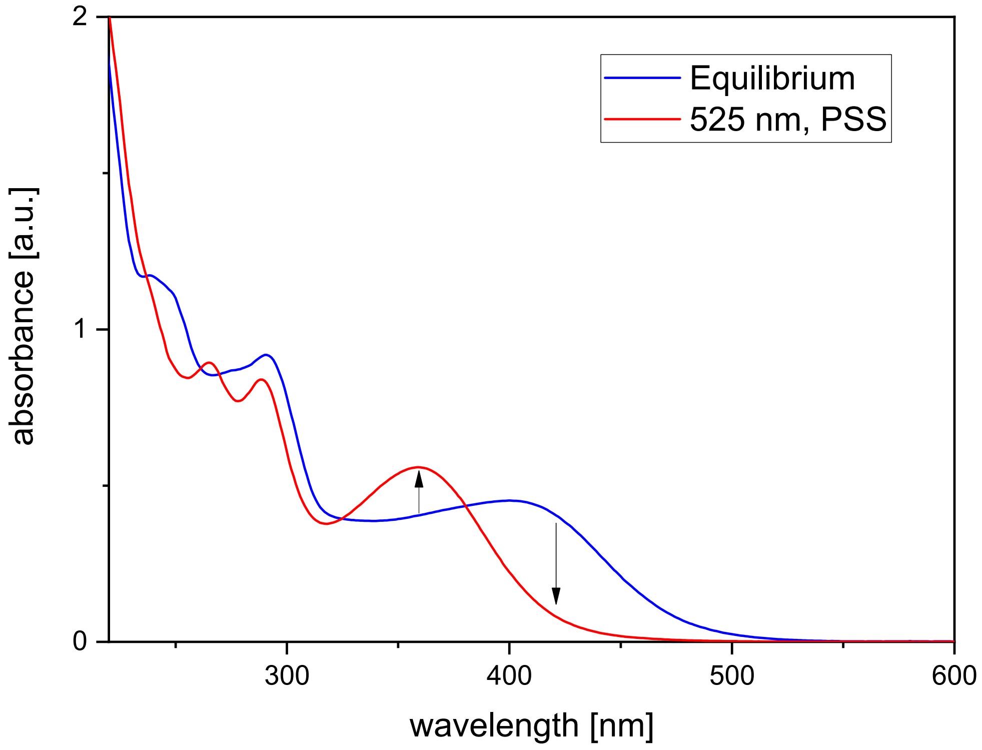

Figure S189: UV-vis spectra of **2h** (6-Ph-quino-caac) in its thermodynamic equilibrium (blue; *E*:*Z* = 93:7) and after irradiation with 525 nm for 1 minute (red; *E*:*Z* = 1:99, PSS) in thf. Note: A small amount of KHMDS (0.5 – 1 mg) was added prior to the experiment.

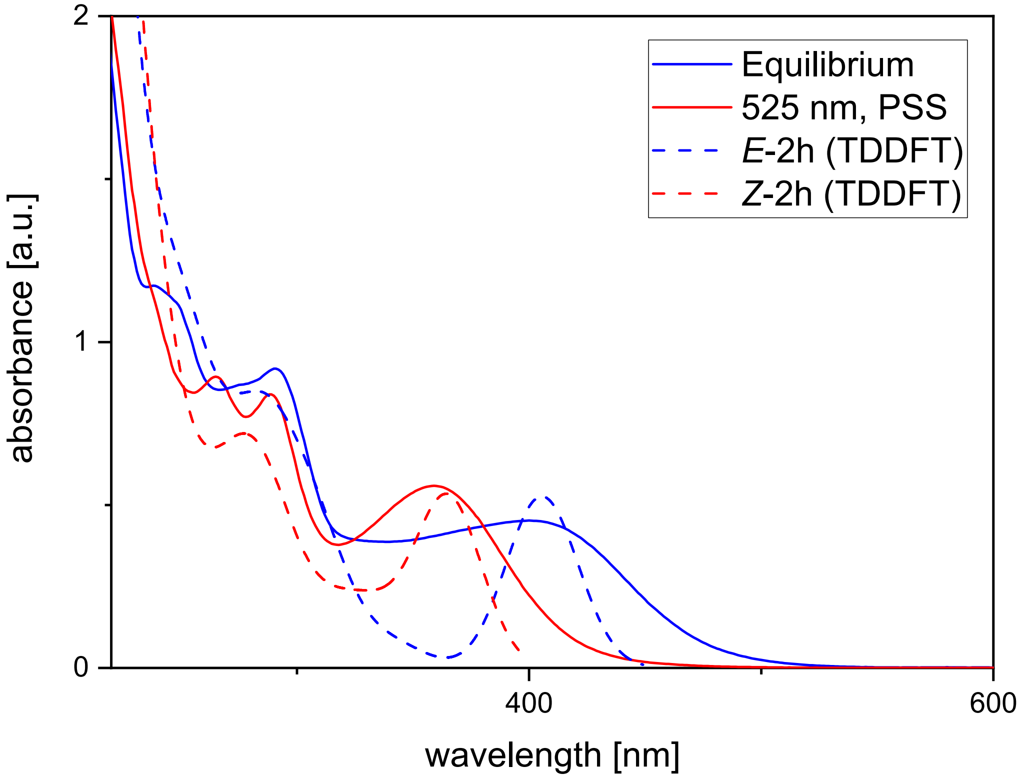

Figure S190: UV-vis spectra of **2h** in its thermodynamic equilibrium (blue; *E*:*Z* = 93:7) and after irradiation with 525 nm for 1 minute (red; *E*:*Z* = 1:99, PSS) in thf compared with the calculated TDDFT spectra for the *E*- and *Z*-isomer in thf. TDDFT spectra are corrected by 1.12 as a scalar factor according to Fehér *et al.*[31] Note: A small amount of KHMDS (0.5 – 1 mg) was added prior to the experiment.

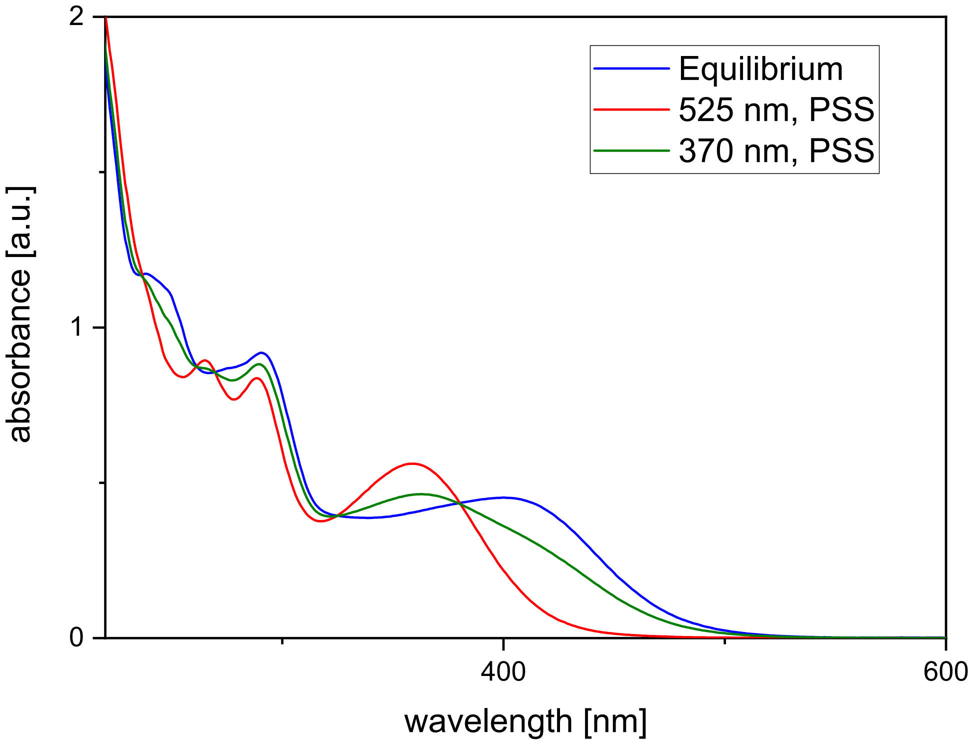

Figure S191: UV-vis spectra of **2h** in its thermodynamic equilibrium (blue; *E*:*Z* = 93:7), after irradiation with 525 nm for 1 minute (red; *E*:*Z* = 1:99, PSS) and after subsequent irradiation with 370 nm for 1 minute (green; *E*:*Z* = 35:65, PSS) in thf. Note: A small amount of KHMDS (0.5 – 1 mg) was added prior to the experiment.

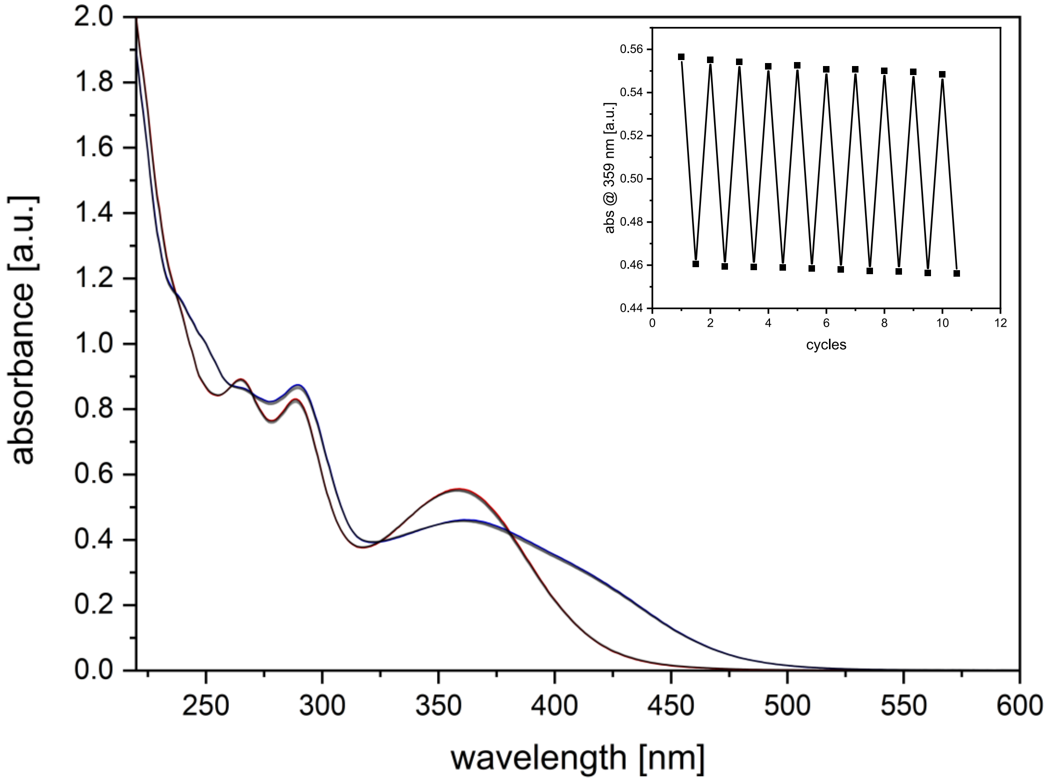

Figure S192: Forth- and back-switching of **2h**. First irradiation with 525 nm for 2 min (PSS), then irradiation with 370 nm for 2 min (PSS). This was repeated for 10 times. A minor decomposition can be observed as the absorbance decreases slightly.

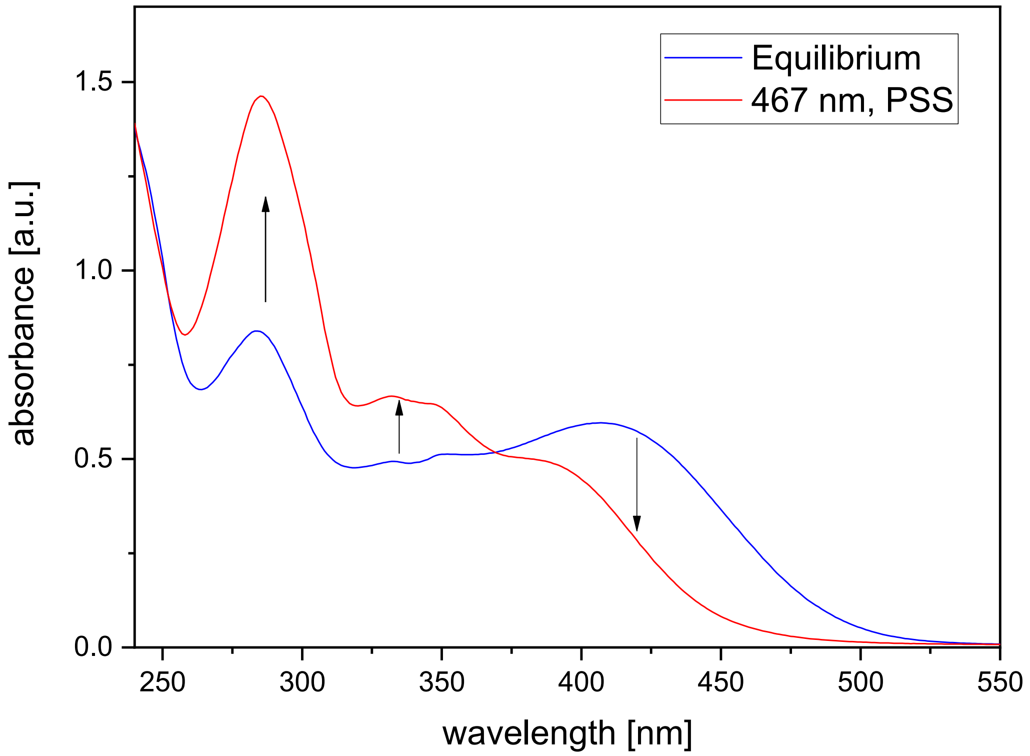

Figure S193: UV-vis spectra of **2i** in its thermodynamic equilibrium (blue; *E*:*Z* = 75:25) and after irradiation with 467 nm for 1 minute (red; *E*:*Z* = 2:98, PSS) in thf. Note: A small amount of KHMDS (0.5 – 1 mg) was added prior to the experiment.

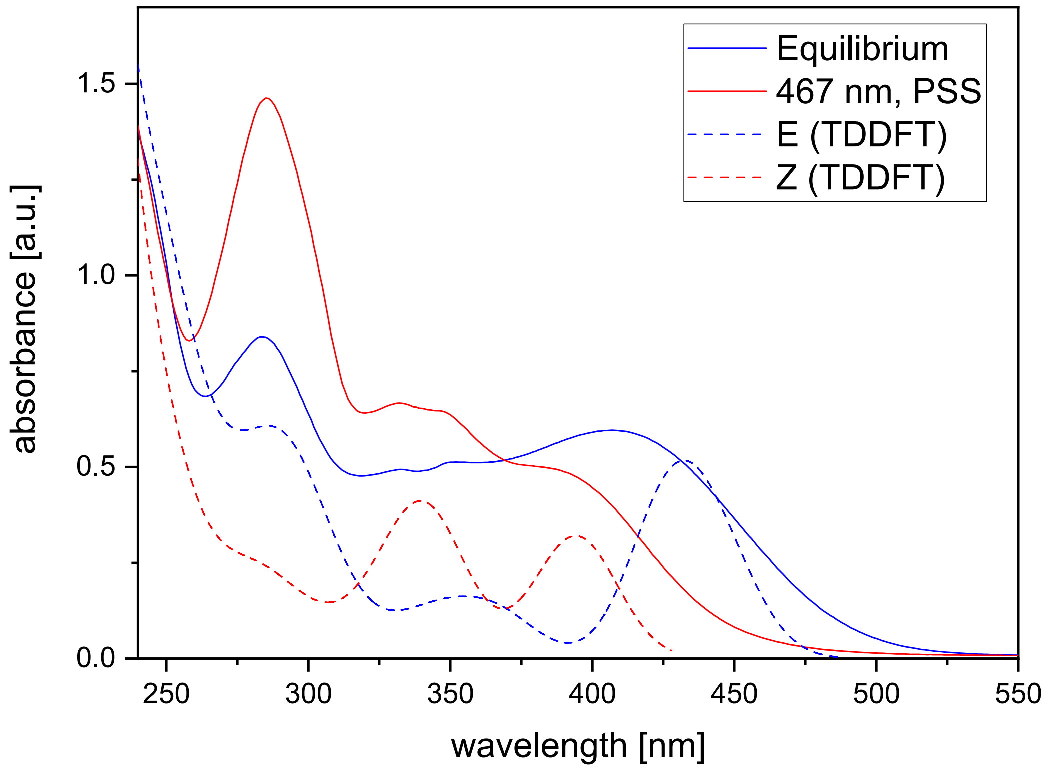

Figure S194: UV-vis spectra of **2i** in its thermodynamic equilibrium (blue; *E*:*Z* = 75:25) and after irradiation with 467 nm for 1 minute (red; *E*:*Z* = 2:98, PSS) in thf compared with the calculated TDDFT spectra for the *E*- and *Z*-isomer in thf. TDDFT spectra are corrected by 1.12 as a scalar factor according to Fehér *et al.*[31] Note: A small amount of KHMDS (0.5 – 1 mg) was added prior to the experiment.

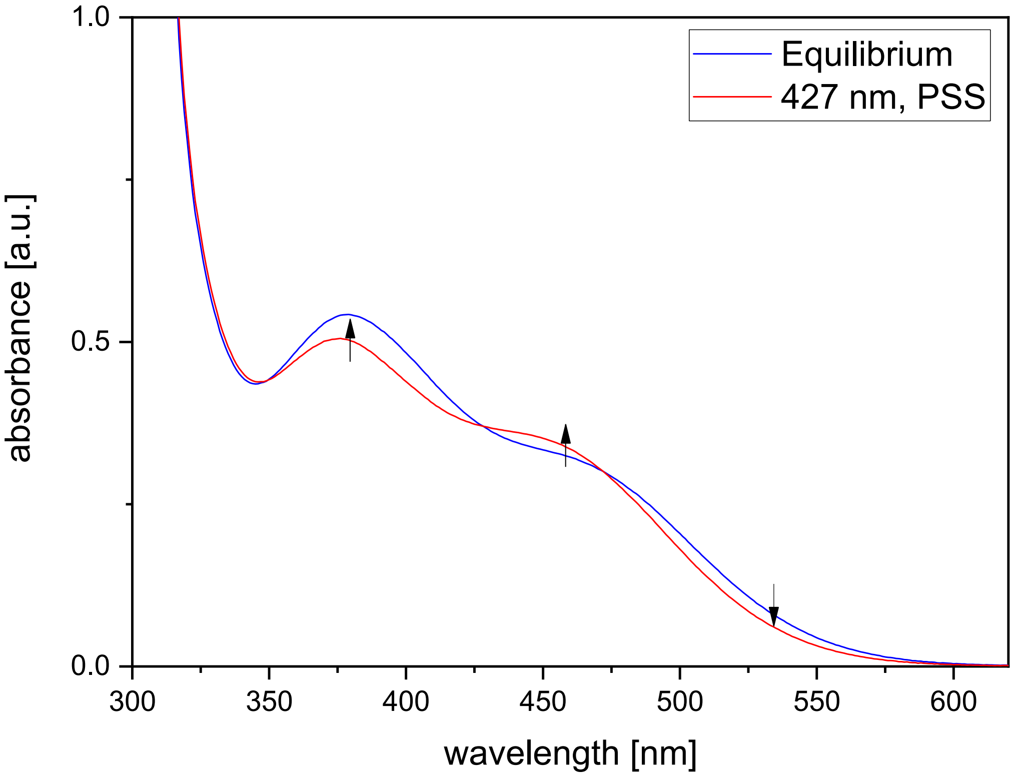

Figure S195: UV-vis spectra of **2j** in its thermodynamic equilibrium (blue; *E*:*Z* = 100:0) and after irradiation with 427 nm for 30 minutes (red; *E*:*Z* = 1:1.56, PSS) in thf. Note: A small amount of KHMDS (0.5 – 1 mg) was added prior to the experiment.

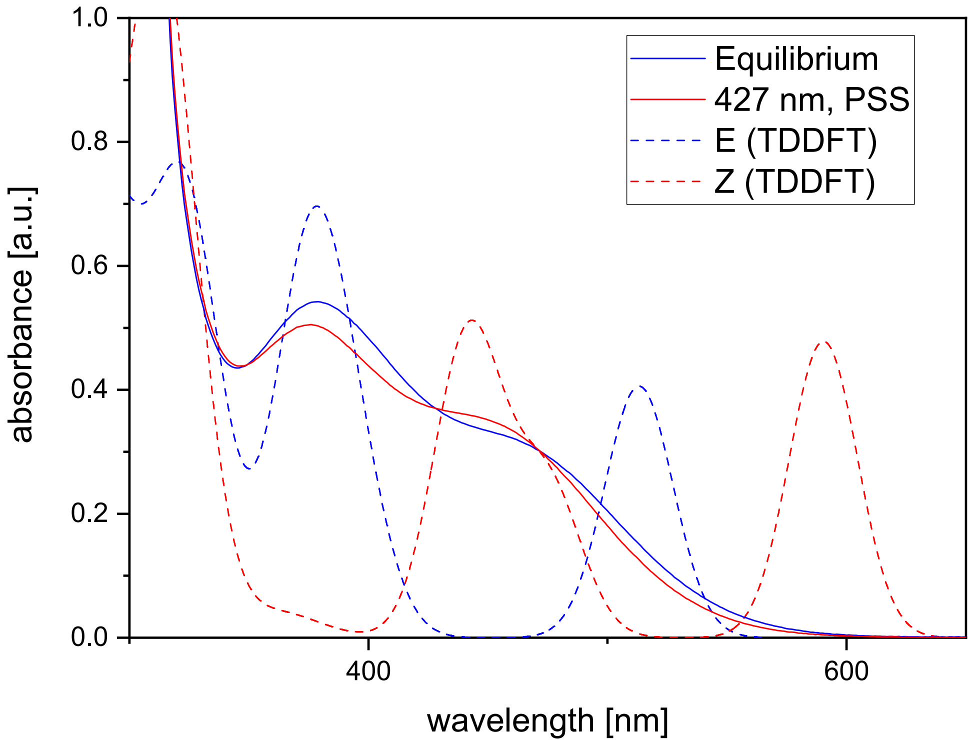

Figure S196: UV-vis spectra of **2j** in its thermodynamic equilibrium (blue; *E*:*Z* = 100:0) and after irradiation with 427 nm for 30 minutes (red; *E*:*Z* = 1:1.56, PSS) in thf compared with the calculated TDDFT spectra for the *E*- and *Z*-isomer in thf. TDDFT spectra are corrected by 1.12 as a scalar factor according to Fehér *et al.*[31] Note: A small amount of KHMDS (0.5 – 1 mg) was added prior to the experiment.

Investigation of electron hole catalysis

To investigate the switching behaviour of **2g** upon the addition of acid an NMR sample of **2g** (20 µmol, 1 equiv) in C_6D_6 was prepared. A ^{1}H-NMR and an EPR spectra was measured and the sample was irradiated with 467 nm for 30 minutes (PSS). Then a ^{1}H-NMR and an EPR spectra were measured and then LutHOTf (0.05 equiv) was added and the measurements were repeated.

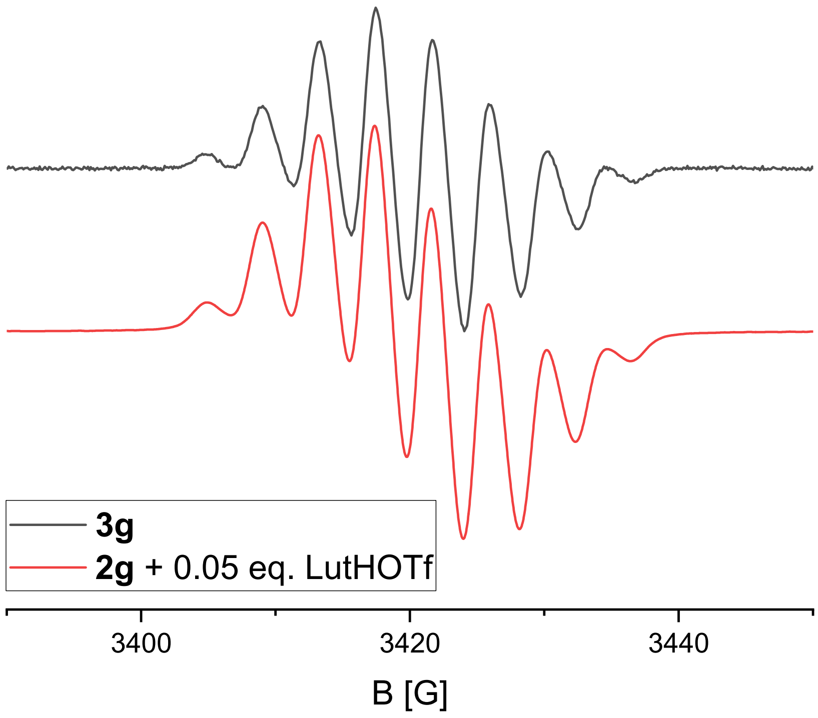

Figure S197: EPR-spectra after the addition of 0.05 equiv. lutidinium triflate to **2g** in C_6D_6 and the EPR spectra of **3g** in thf.

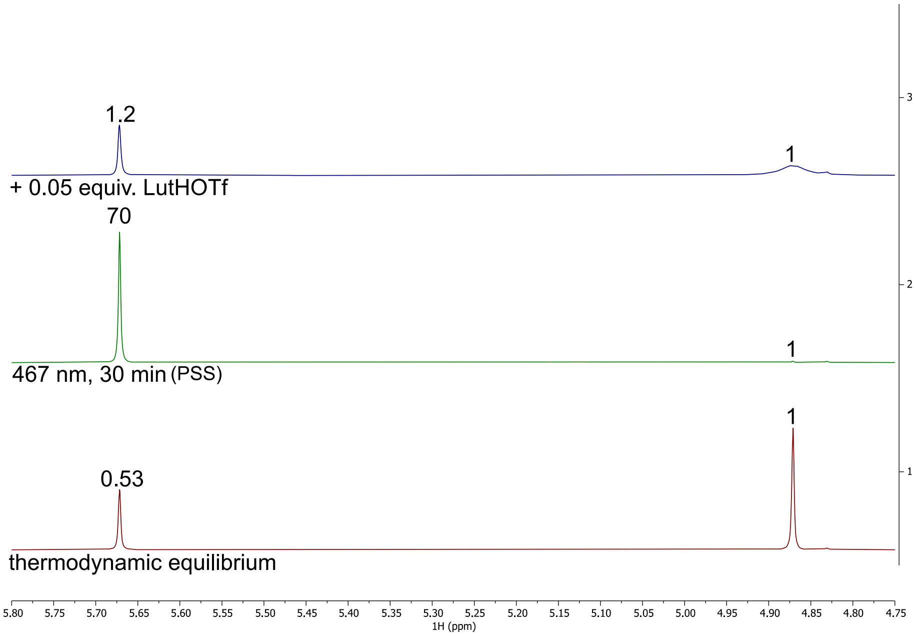

Figure S198: ^{1}H-NMR spectra in C_6D_6 of **2g** in thermodynamic equilibrium followed by irradiation with 467 nm, 30 min (PSS) followed by the addition of 0.05 equiv. LutHOTf. Magnification from 4.75 to 5.8 ppm.

Afterwards 0.1 equiv. of KHMDS was added to the sample and a ^{1}H-NMR and an EPR spectrum were measured. Then the sample was irradiated (467 nm, 30 min, PSS) and the measurements were repeated.

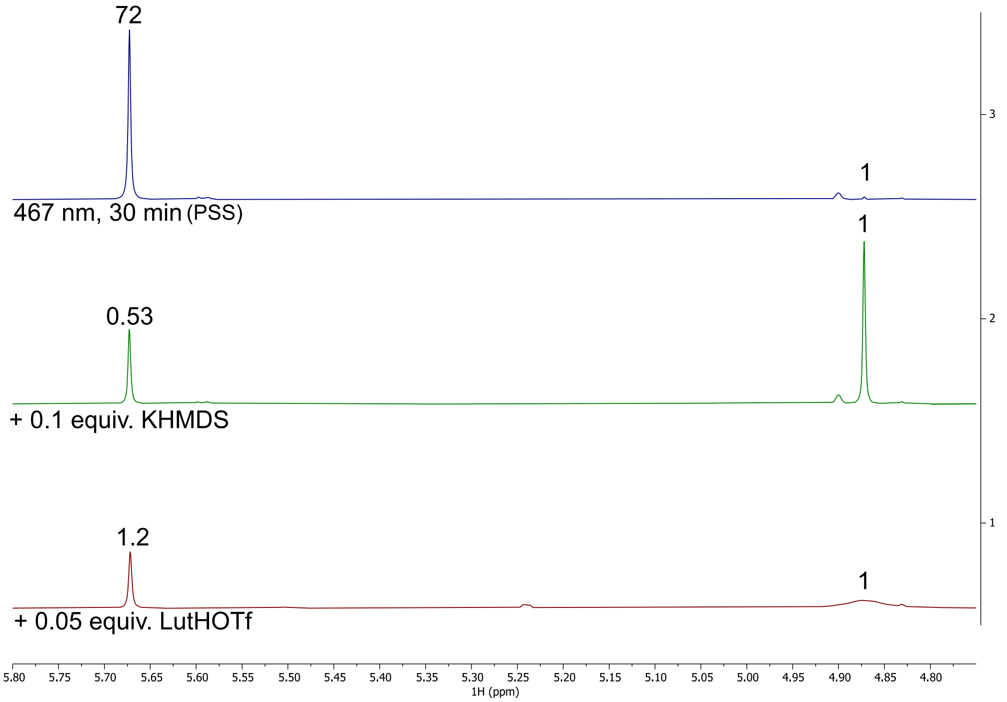

Figure S199: ^{1}H-NMR spectra of **2g** in C_6D_6 after the addition of 0.05 equiv. of LutHOTf followed by the addition of 0.1 equiv of KHMDS followed by irradiation at 467 nm for 30 minutes (PSS). Magnification from 4.75 to 5.8 ppm.

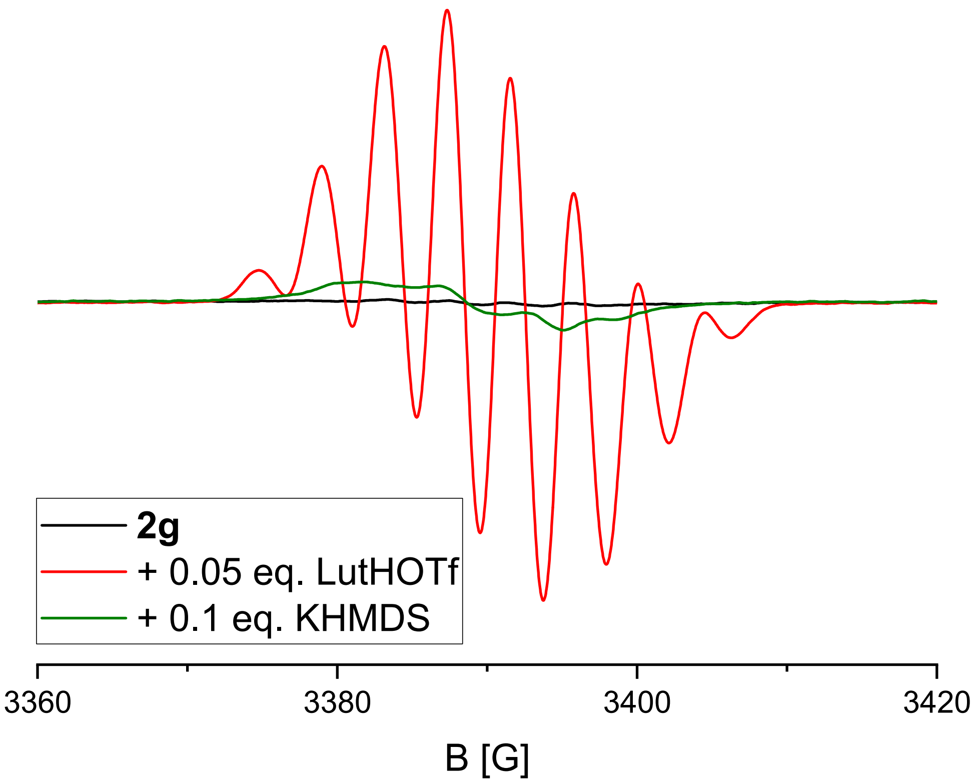

Figure S200: EPR-spectra of **2g**, after LutHOTf addition and after KHMDS addition in C_6D_6.

The experiment was also performed with isolated radical cation (0.25 mol%) as the electron hole.

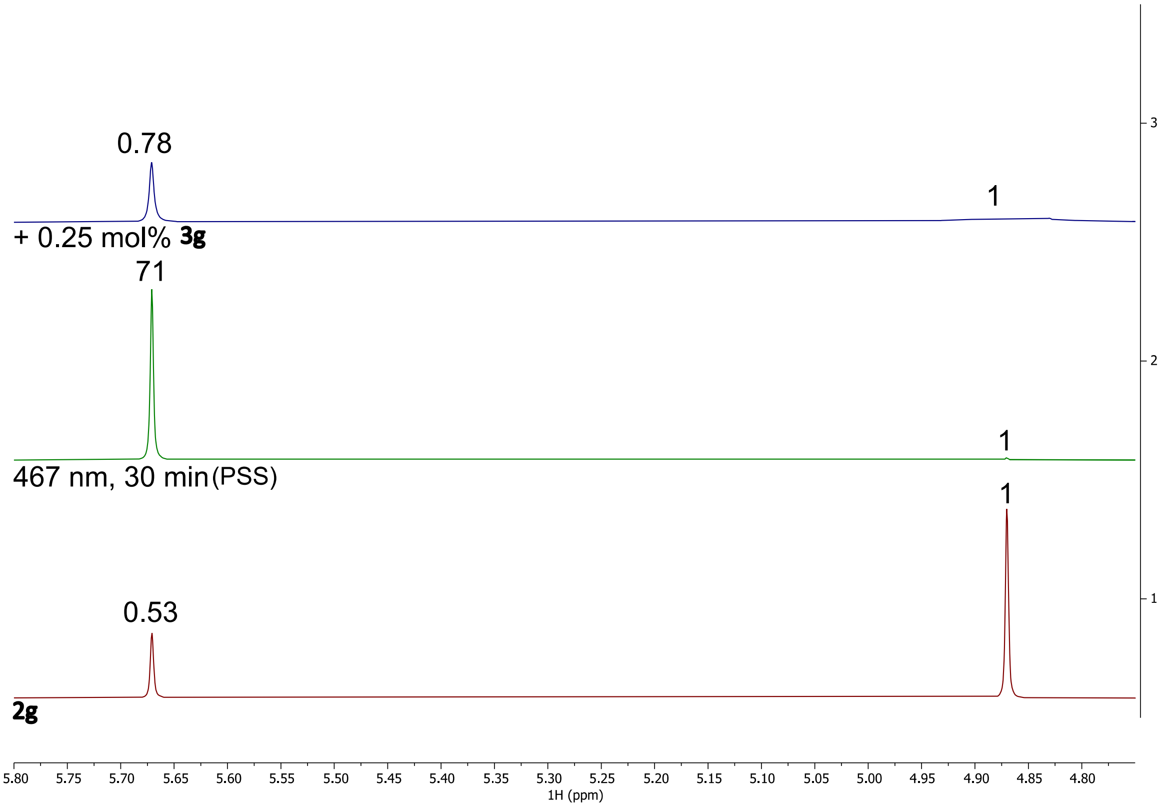

Figure S201: ^{1}H-NMR spectra of **2g** in C_6D_6 in thermodynamic equilibrium followed by irradiation with 467 nm, 30 min (PSS) followed by the addition of 0.25 mol% radical cation. Magnification from 4.75 to 5.8 ppm.

The electron hole catalysis was also observed by UV-Vis spectroscopy. Here a UV-vis spectrum was measured of the substance in its thermodynamic equilibrium and after irradiation with 467 nm for 30 minutes (PSS). Then 0.01 mol% radical cation was added and another UV-vis spectrum was measured immediately.

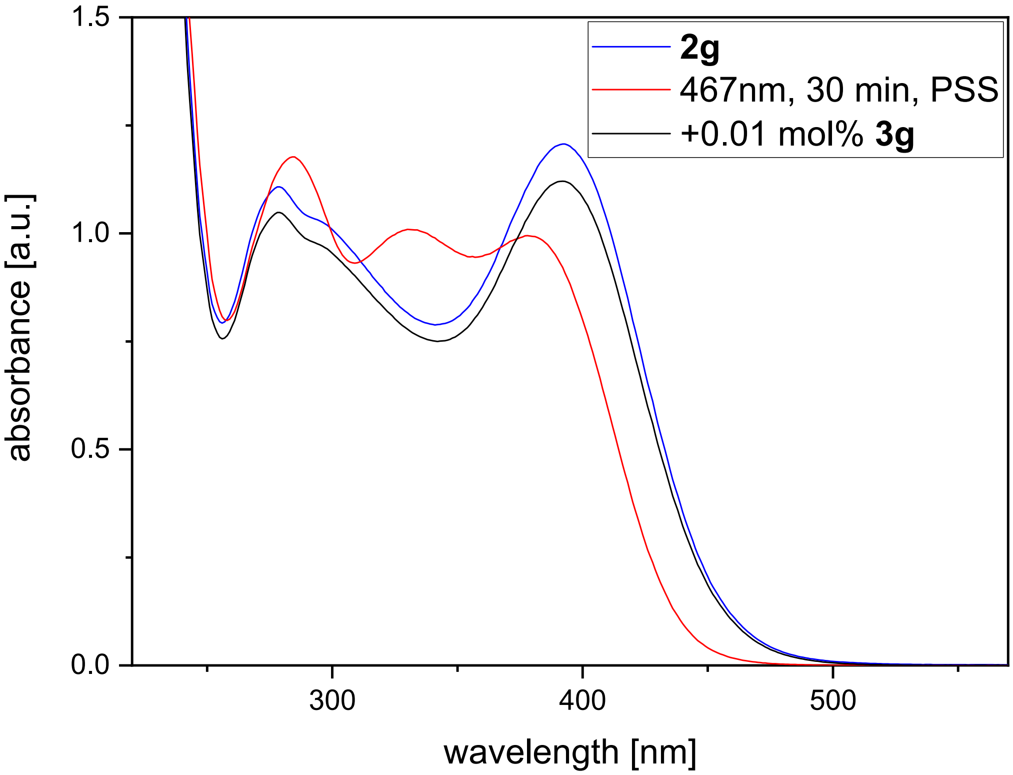

Figure S202: UV-vis spectra of hybrid **2g** in thf in its thermodynamic equilibrium, after irradiation with 467 nm for 30 minutes (PSS) and then after the addition of 0.01 mol% radical cation to the metastable *Z*-isomer.

The experiment was repeated with 0.001 mol% radical cation. Here it was observed that the *Z*->*E* isomerisation takes up to 8 minutes until the isomerisation is complete.

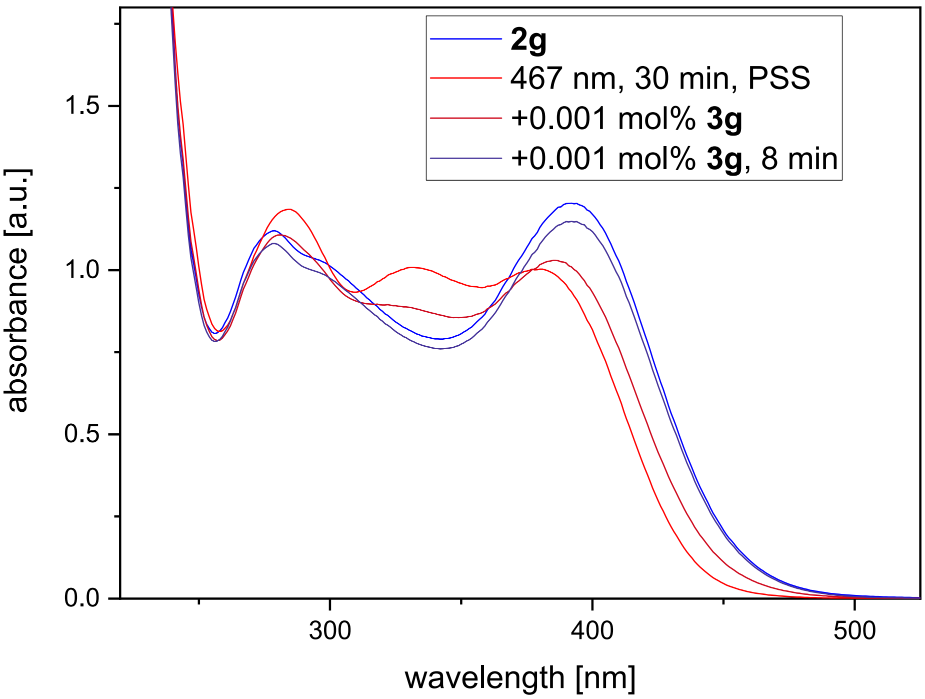

Figure S203: UV-vis spectra of hybrid **2g** in thf in its thermodynamic equilibrium, after irradiation with 467 nm for 30 minutes (PSS) and then after the addition of 0.001 mol% radical cation to the metastable *Z*-isomer.

In accordance with experiments performed by HECHT *et al.* it was investigated whether it is possible to generate small amounts of the radical cation electro-chemically and then observe the isomerisation with spectroelectro chemistry (SEC).[32] It was observed that possibly due to an interaction of the hybrid with the platinum electrode a small amount of radical cation was generated (recognisable by the broad absorption from 600 nm to 800 nm). Thus, it was not possible to induce the isomerisation by irradiation without the addition of small amounts of KHMDS (0.5 – 1 mg).

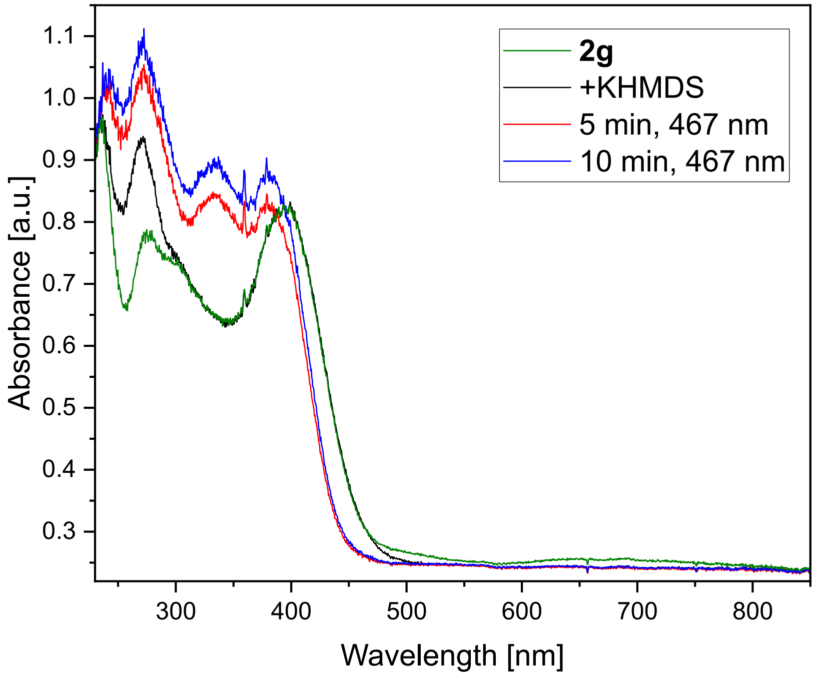

Figure S204: Left: UV-vis spectra of **2g** in thf (0.1 M *n*-Bu_4NPF_6), with KHMDS added (0.5 – 1 mg) and after irradiation at 467 nm for 5 and 10 minutes.

The increase in absorbance going from 5 to 10 minutes irradiation can be explained as the heat generated by the Kessil lamp evaporates some thf.

For the SEC experiment with added KHMDS the controlled potential coulometry (CPC) approach was chosen. It can be observed that while a potential was applied the *Z*->*E* isomerisation took place and while there was no potential applied the isomerisation stopped. This can be accorded to the KHMDS which quenches the electro chemically generated radical cation and thus inhibits the isomerisation. For details of the SEC setup see chapter *UV-vis spectroelectrochemistry*.

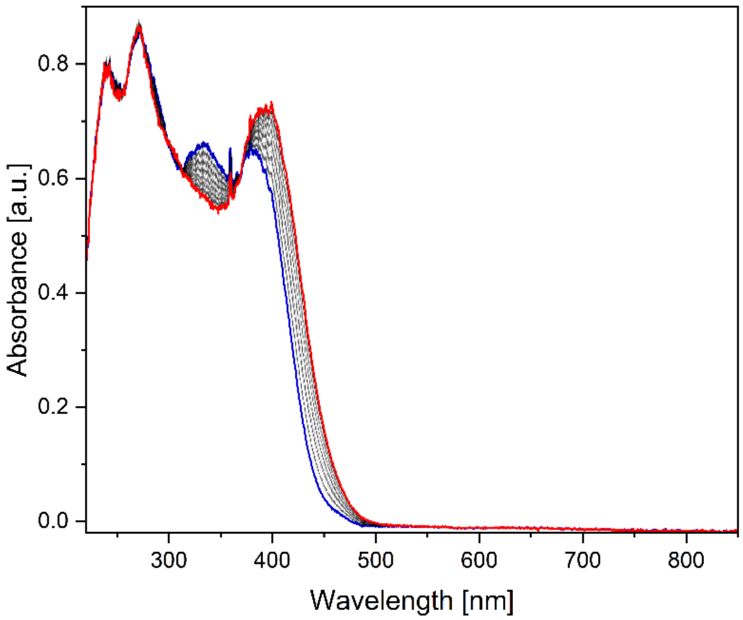

Figure S205: SEC experiment with 0.5 – 1 mg of KHMDS added. CPC at –1V. Starting from the metastable *Z*-isomer (blue) going to the hybrid in the thermodynamic equilibrium (red). Note: No radical cation can be observed during the SEC.

To verify that KHMDS does reduce the racial cation to the neutral hybrid molecule a stochiometric reduction was investigated. Here for **3g** was reacted with KHMDS (3 equiv) in thf for 45 minutes. Afterwards the solvent was evaporated and a crude ^{1}H-NMR was measured. The resulting •N(TMS)$_2$ is known but reported to be highly reactive.[33] Furthermore an EPR spectrum of the crude mixture was measured confirming the reaction of **3g** to **2g.**

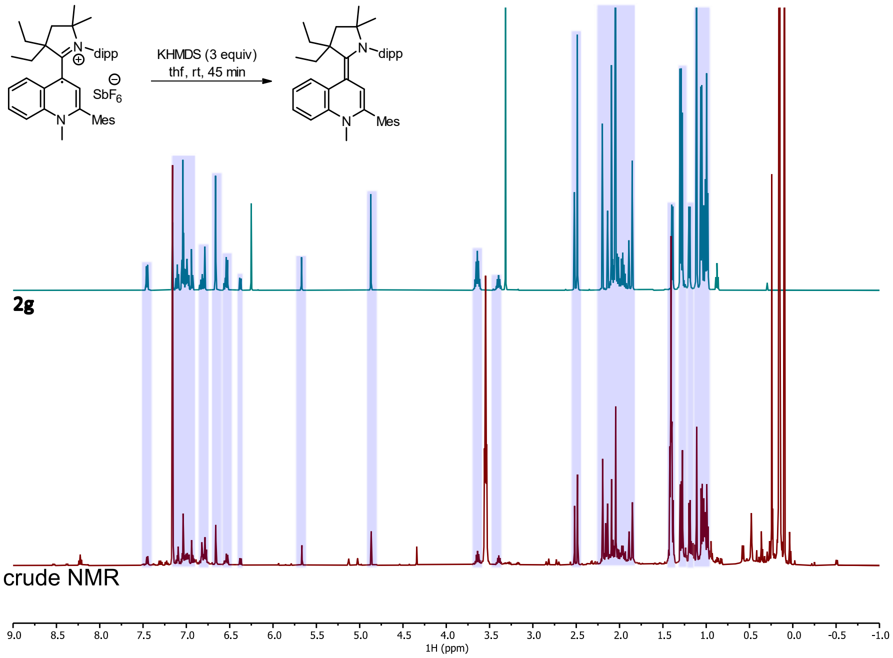

Figure S206: Crude ^{1}H-NMR of stoichiometric reduction of radical cation with KHMDS. ^{1}H of the neutral hybrid **2g** contains 1,3,5-trimethoxybenzene. ^{1}H-NMRs were measured in C_6D_6.

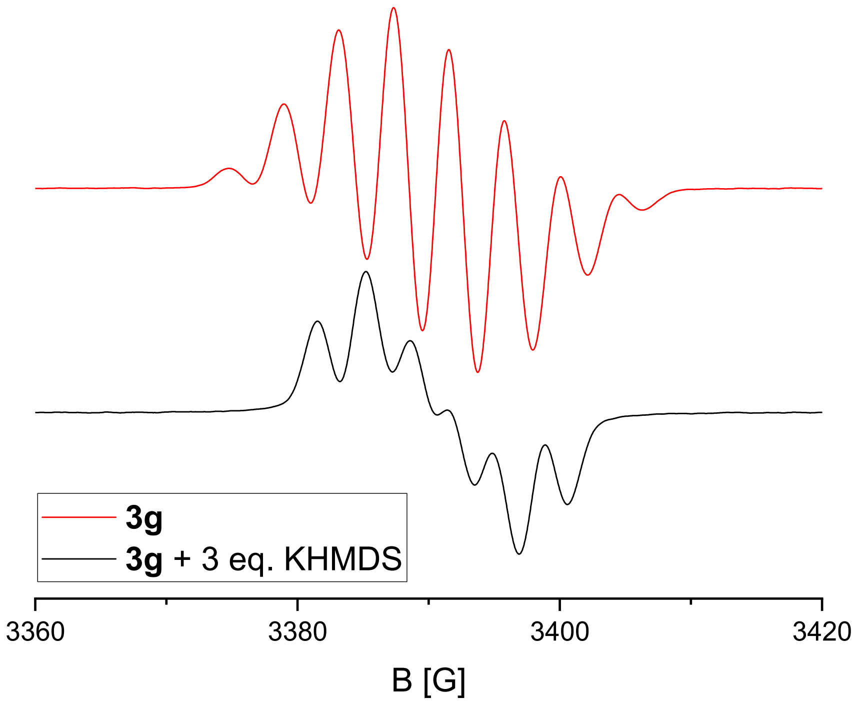

Figure S207: EPR spectrum of the stoichiometric reduction of radical cation **3g** with KHMDS in thf and an EPR spectrum of **3g**.

Stability measurements

To investigate the stability of the hybrids during the photo-switching a sample of **2g** (20 µmol, 1 equiv) in 0.5 mL C_6D_6 in a J- Young NMR-tube was prepared. Also 1,3,5-trimethoxybenzene (6.66 µmol, 0.33 equiv) was added as an internal standard. The sample was then subsequently irradiated (467 nm, 15 min) and heated (80 °C, 1 h). After every step a ^{1}H-NMR was measured and the integrals were measured against the internal standard.

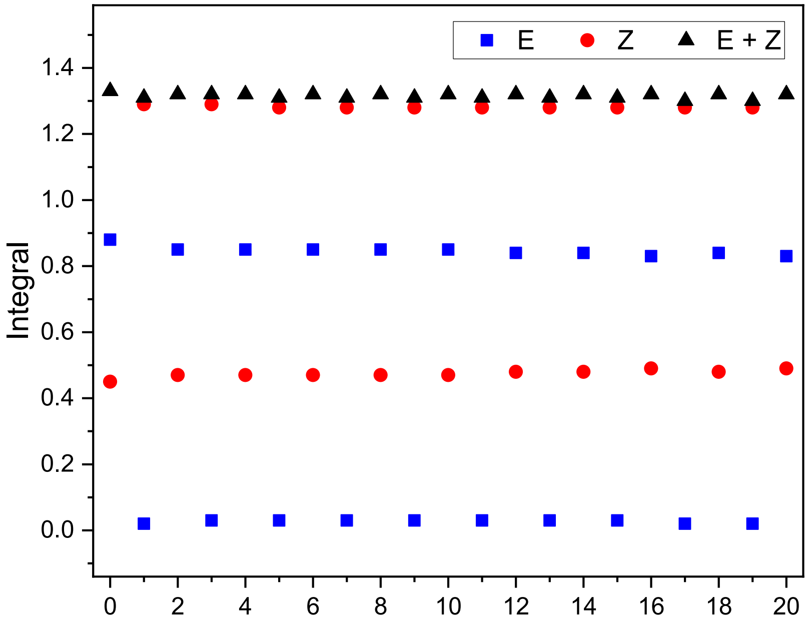

Figure S208: Measured integral of *E*- and *Z*-Isomer of **2g** against internal standard over 20 switching cycles.

To investigate the stability upon redox-switching **2g** was dissolved in thf (0.1 M n-Bu_4NPF_6) and transferred into a SEC-cuvette (for details see SEC-part below). Controlled potential coulometry (CPC) was used to switch from the neutral oxidation state to the radical cation and then to the dication. Afterwards CPC was used again to switch back to the radical cation and finally to the neutral oxidation state. The used potentials and times are listed below.

–0.9 V (30 s) -> –0.4 V (200 s) -> 1 V (200 s)

-> 1 V (30 s) -> –0.52 V (200 s) -> –0.9 V (200 s)

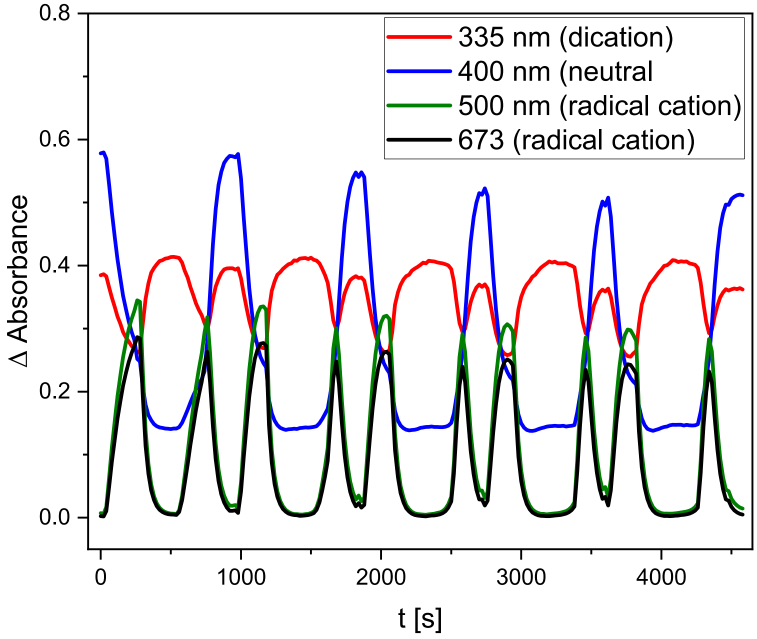

Figure S209: Changes in absorption (difference spectrum) of **2g** in thf (0.1 M n-Bu_4NPF_6) at room temperature. Controlled potential coulometry was used to switch between **2g**, **3g** and **4g**.

Electrochemical Measurements

CVs were measured with a Gamry Instruments Reference 600+ and integrated iR compensation using the positive feedback method. Samples were measured under inert atmosphere in a nitrogen glove box at rt in dry solution of the respective solvent containing tetrabutylammonium hexafluorophosphate (0.1 M). Electrochemical grade tetra-*n*-butyl-ammonium hexafluorophosphate was dried under vacuum at 85 °C for 2 days prior to use. The setup consisted of a three-neck flask with a three-electrode setup containing a glassy carbon working electrode (GC: CH Instruments, ALS Japan; A = 7.1 mm^2), a platinum wire as a counter electrode, and an Ag/$AgNO_3$ reference electrode (0.01 M $AgNO_3$ in 0.1 M *n*-Bu_4NPF_6 in CH_3CN). The reference electrode was freshly prepared by using a fritted sample holder (Vycor glass), which was activated by storing it in a CH_3CN solution for one night, followed by diluted HNO_3 (1M) for one night, followed by demineralized water for one night, followed by CH_3CN for one night dried and stored in the solvent used for the CV measurement for at least one additional night. To the fritted sample holder was added a freshly prepared 0.01 M $AgNO_3$/0.1 M *n*-Bu_4NPF_6 solution in CH_3CN and a silver wire. The working electrode was cleaned before measuring a new compound by standard methods: washed with water, polished with an Alox-slurry (0.05 µm), washed with millipore water, sonicated in HPLC grade EtOH for 3 minutes, rinsed with millipore water and HPLC grade EtOH, and dried. The 3-neck cell was filled with a specific amount of compound dissolved in 3 mL the stated solvent and then the CV was measured. The system was furthermore (doubly) referenced internally by addition of ferrocene or diacetylferrocene.

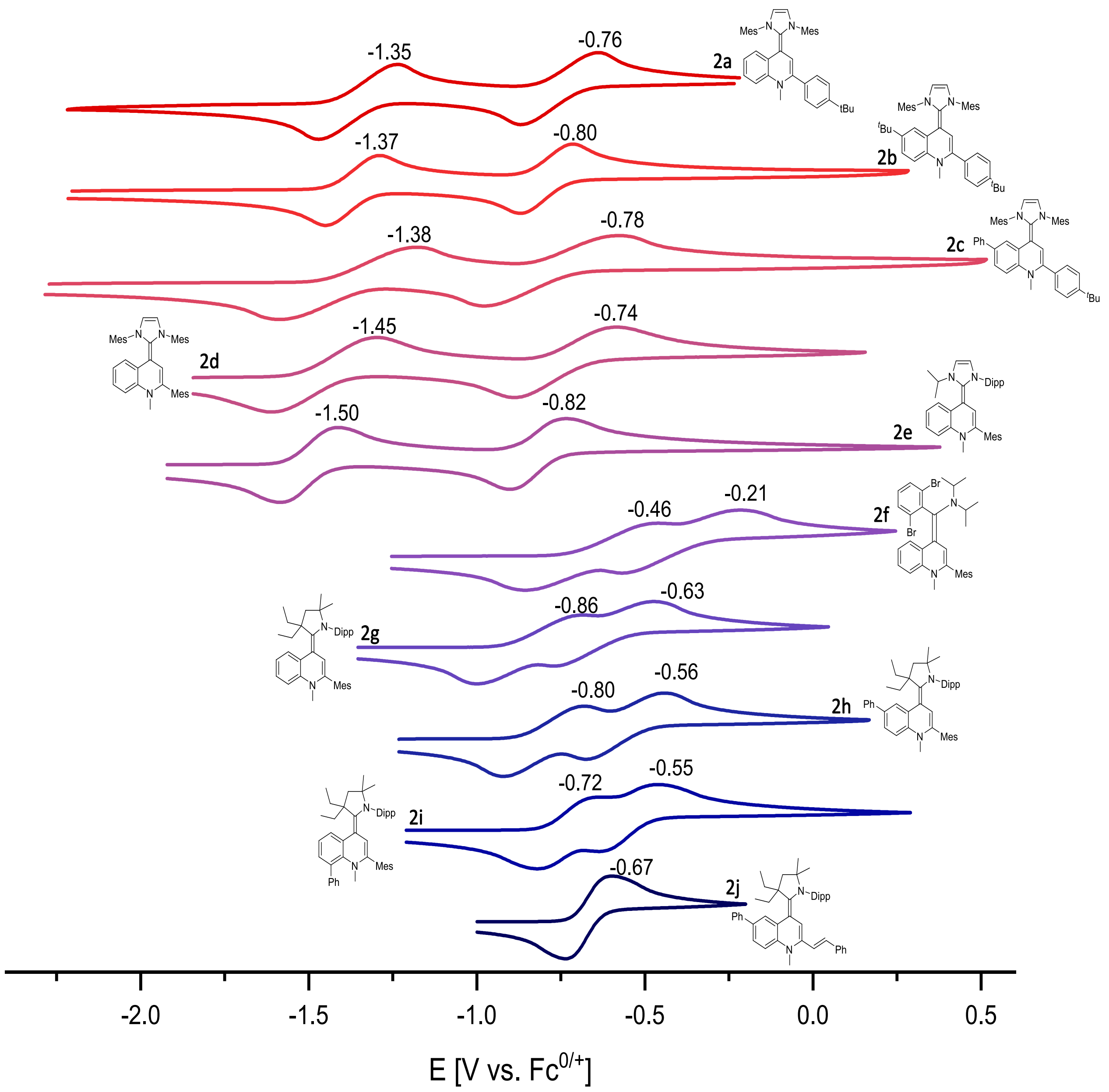

Figure S210: Comparison of normalized cyclic voltammograms in thf (0.1 M *n*-Bu_4NPF_6) at room temperature; scan rate 200 mV s^{-1}; referenced internally against ferrocene or diacetylferrocene.

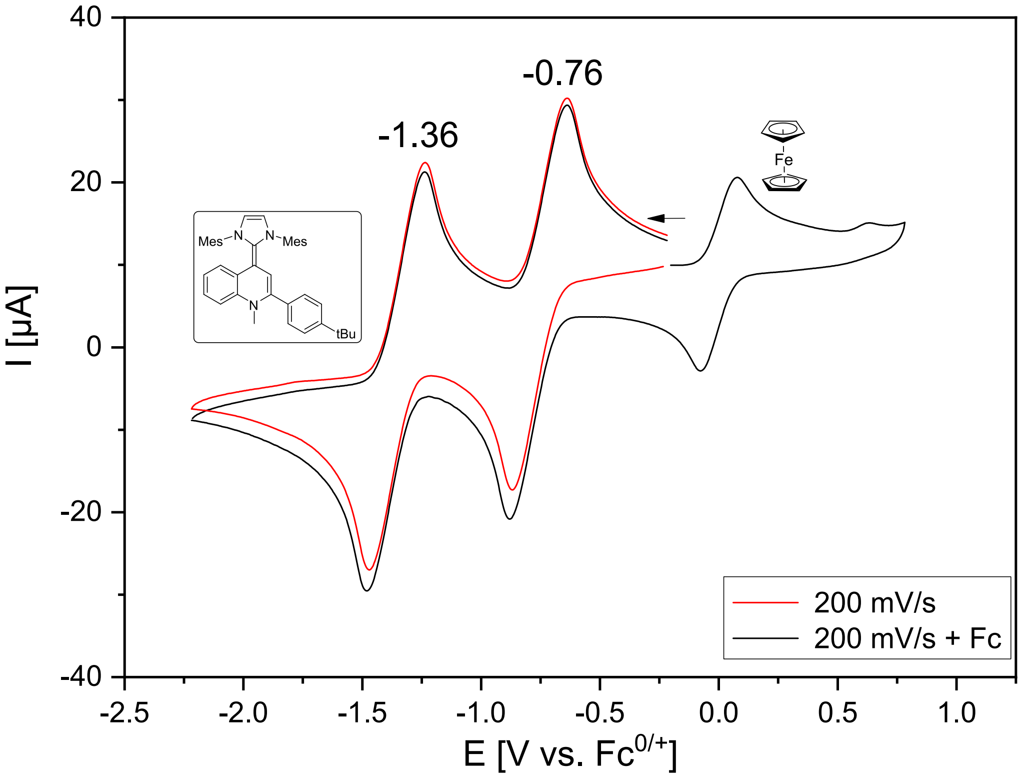

Figure S211: Cyclic voltammograms of **2a** (1.1±0.1 mg mL^{-1}) in thf (0.1 M *n*-Bu_4NPF_6) at room temperature; scan rate 200 mV s^{-1} (iR compensation = 2000 Ohm) referenced internally against ferrocene (arrows indicate scanning direction).

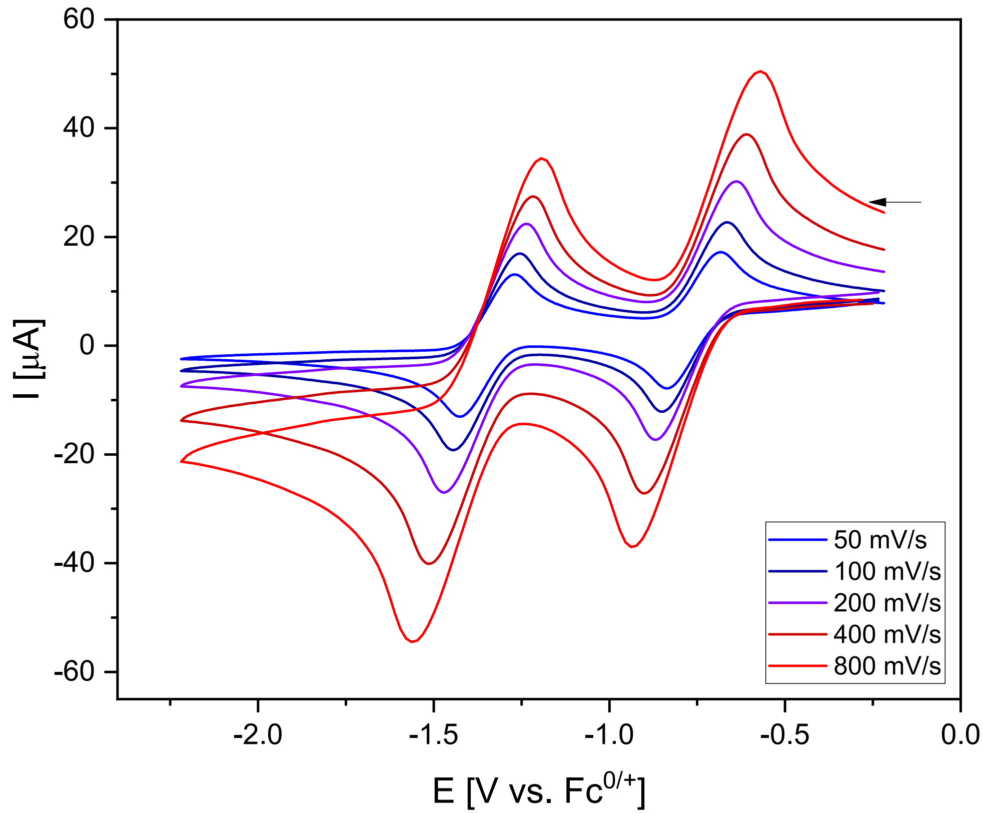

Figure S212: Cyclic voltammograms of **2a** (1.1±0.1 mg mL^{-1}) in thf (0.1 M *n*-Bu_4NPF_6) at room temperature; different scan rates (iR compensation = 2000 Ohm) referenced internally against ferrocene (arrows indicate scanning direction).

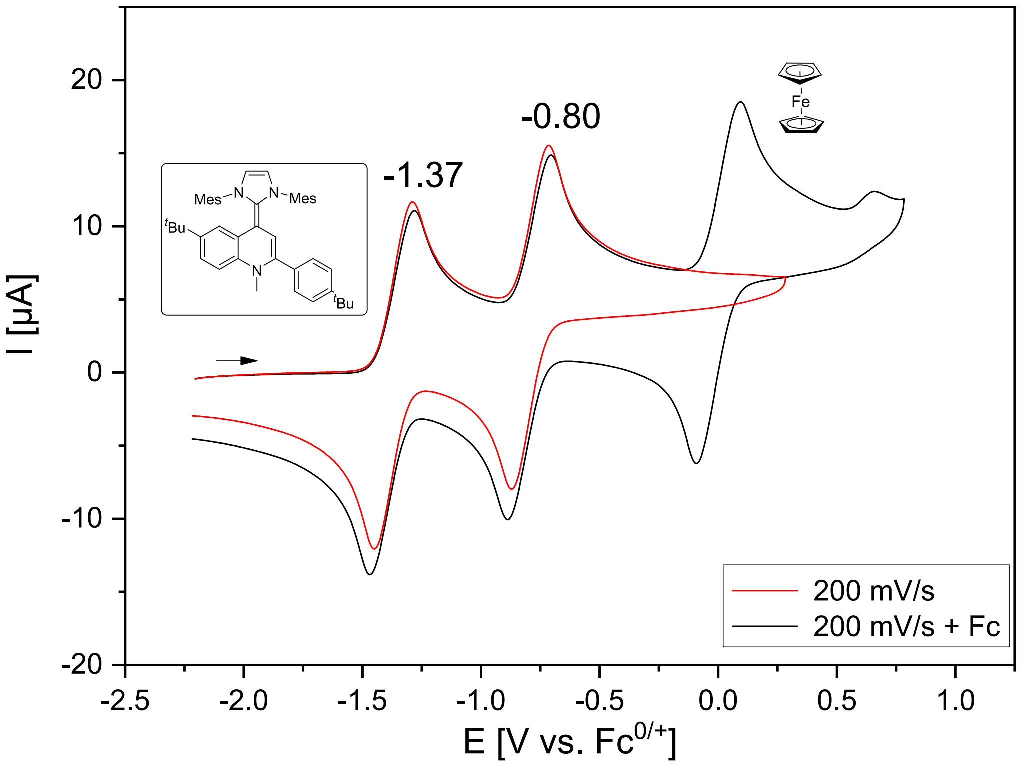

Figure S213: Cyclic voltammograms of **2b** (0.8±0.1 mg mL^{-1}) in thf (0.1 M *n*-Bu_4NPF_6) at room temperature; scan rate 200 mV s^{-1} (iR compensation = 2000 Ohm) referenced internally against ferrocene (arrows indicate scanning direction).

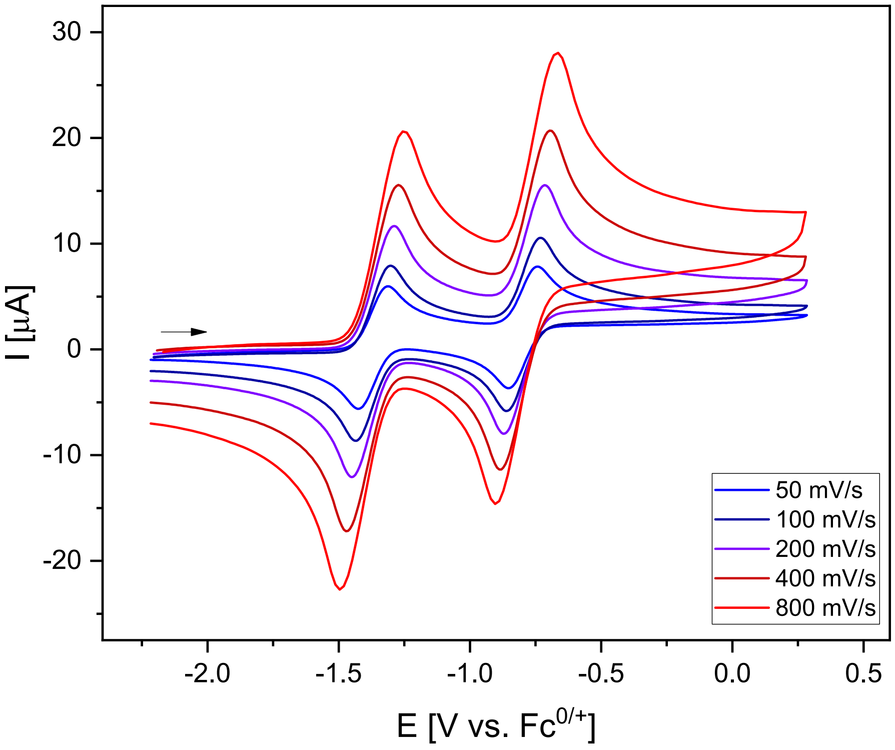

Figure S214: Cyclic voltammograms of **2b** (0.8±0.1 mg mL^{-1}) in thf (0.1 M *n*-Bu_4NPF_6) at room temperature; different scan rates (iR compensation = 2000 Ohm) referenced internally against ferrocene (arrows indicate scanning direction).

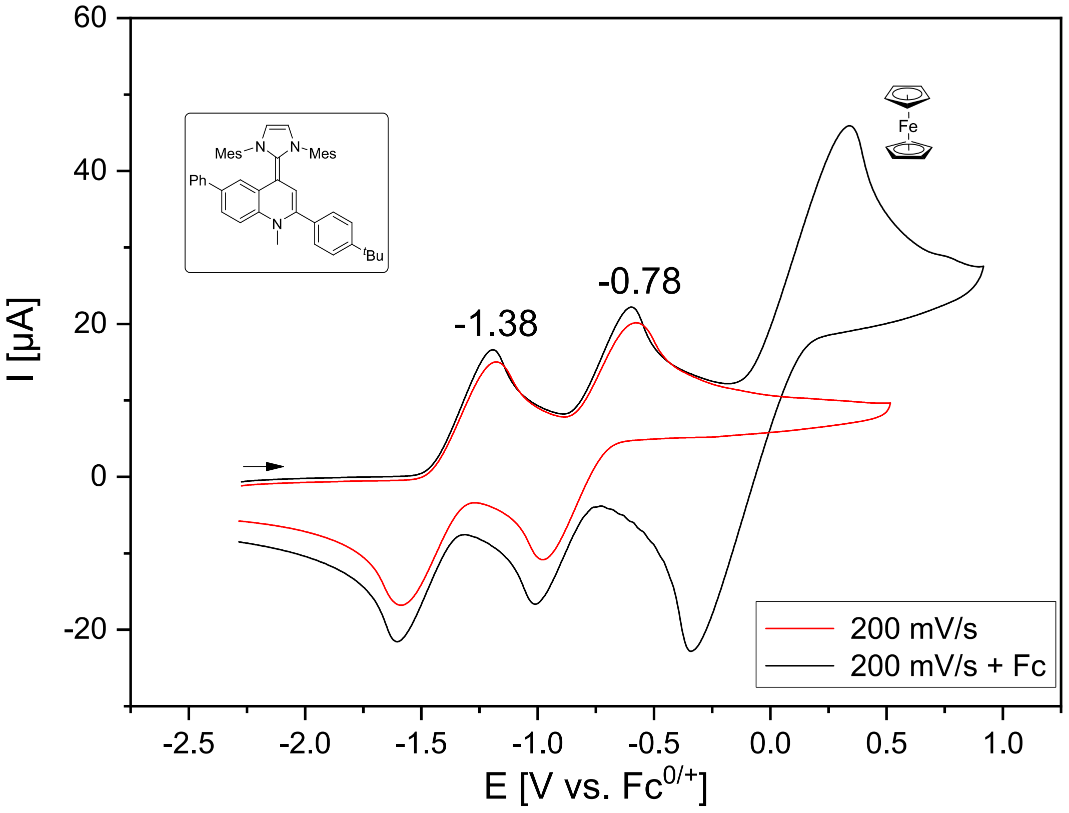

Figure S215: Cyclic voltammograms of **2c** (1.2±0.1 mg mL^{-1}) in thf (0.1 M *n*-Bu_4NPF_6) at room temperature; scan rate 200 mV s^{-1} (iR compensation = 2000 Ohm) referenced internally against ferrocene (arrows indicate scanning direction).

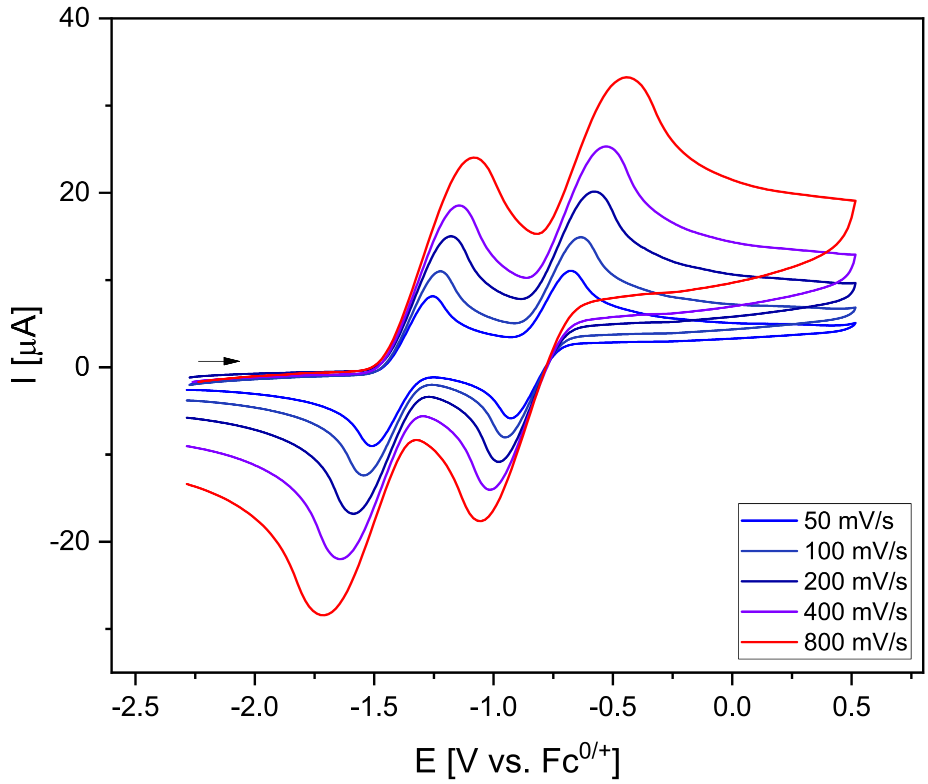

Figure S216: Cyclic voltammograms of **2c** (1.2±0.1 mg mL^{-1}) in thf (0.1 M *n*-Bu_4NPF_6) at room temperature; different scan rates (iR compensation = 2000 Ohm) referenced internally against ferrocene (arrows indicate scanning direction).

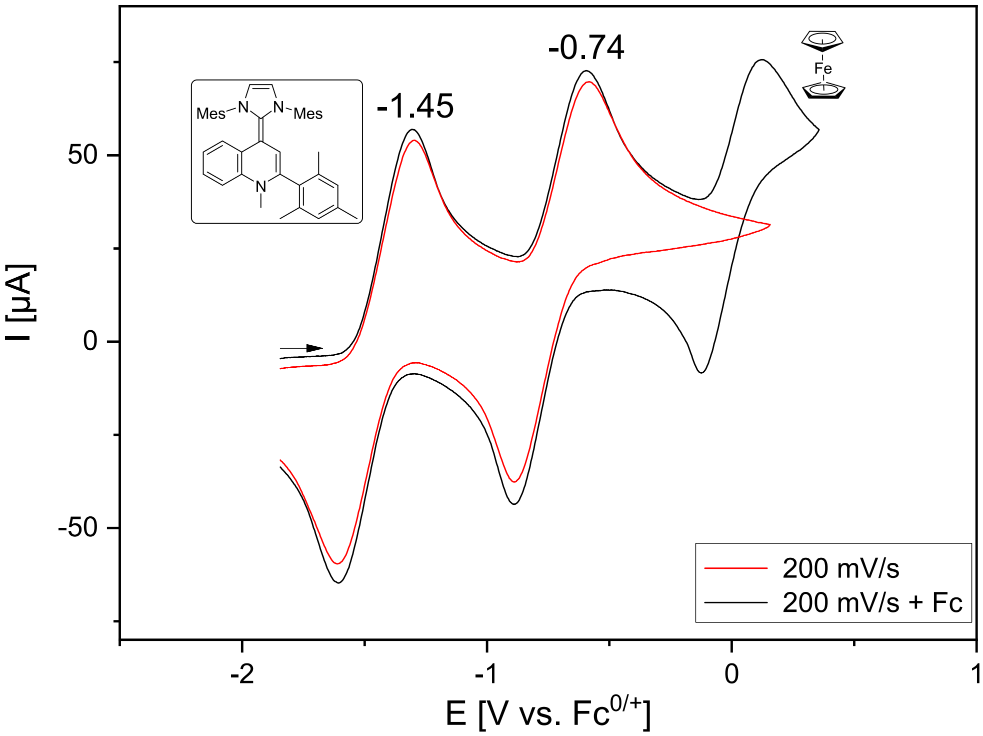

Figure S217: Cyclic voltammograms of **2d** (1.9±0.1 mg mL^{-1}) in thf (0.1 M n-Bu_4NPF_6) at room temperature; scan rate 200 mV s^{-1} (iR compensation = 2050 Ohm) referenced internally against ferrocene (arrows indicate scanning direction).

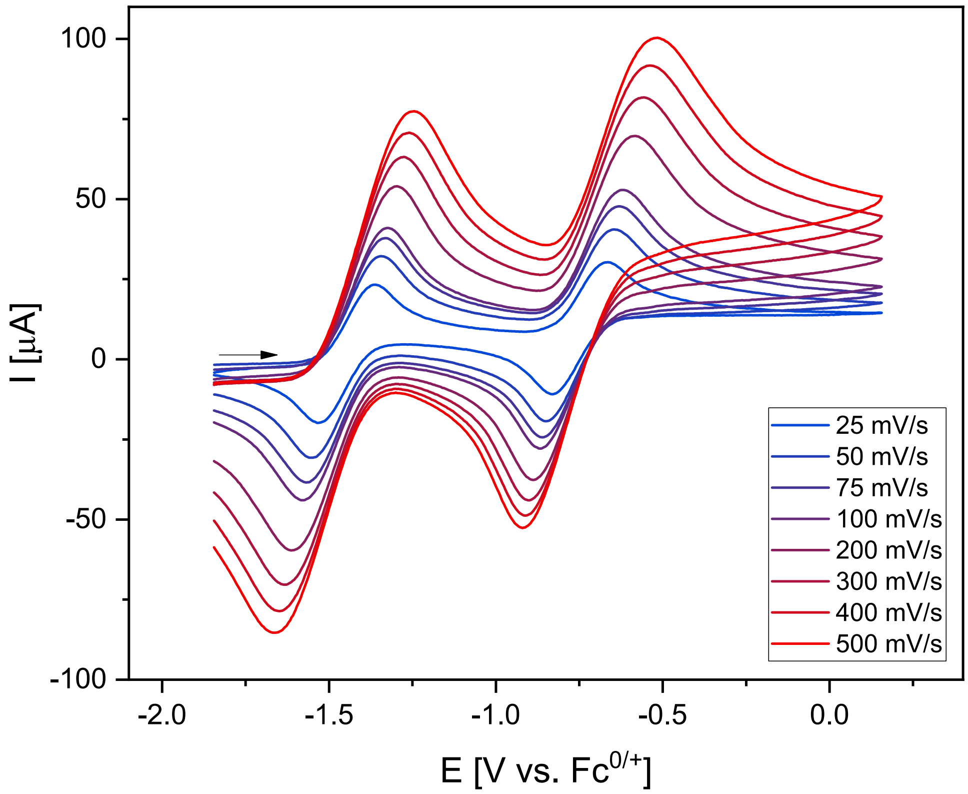

Figure S218: Cyclic voltammograms of **2d** (1.9±0.1 mg mL^{-1}) in thf (0.1 M n-Bu_4NPF_6) at room temperature; different scan rates (iR compensation = 2050 Ohm) referenced internally against ferrocene (arrows indicate scanning direction).

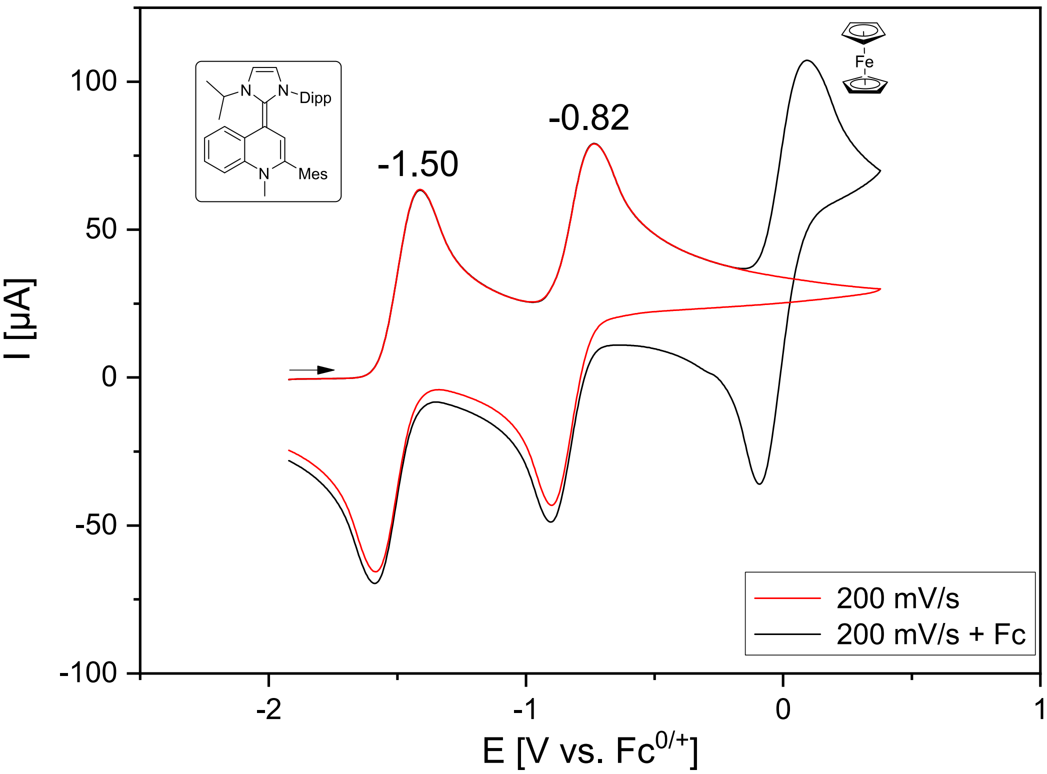

Figure S219: Cyclic voltammograms of **2e** (2.2±0.1 mg mL^{-1}) in thf (0.1 M n-Bu_4NPF_6) at room temperature; scan rate 200 mV s^{-1} (iR compensation = 2000 Ohm) referenced internally against ferrocene (arrows indicate scanning direction).

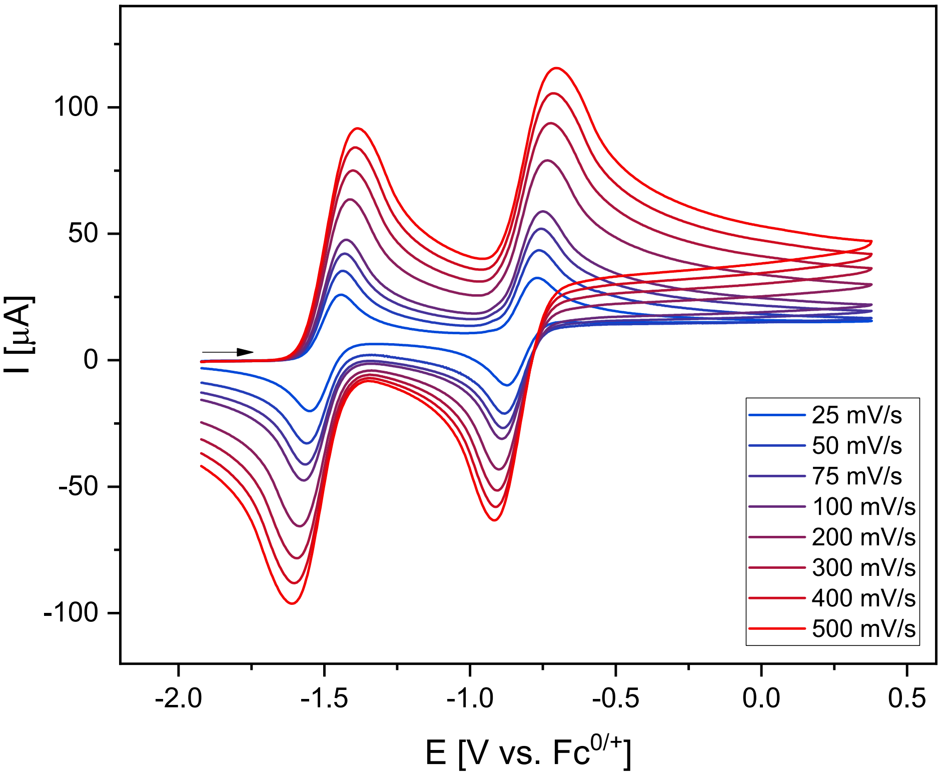

Figure S220: Cyclic voltammograms of **2e** (2.2±0.1 mg mL^{-1}) in thf (0.1 M n-Bu_4NPF_6) at room temperature; different scan rates (iR compensation = 2000 Ohm) referenced internally against ferrocene (arrows indicate scanning direction).

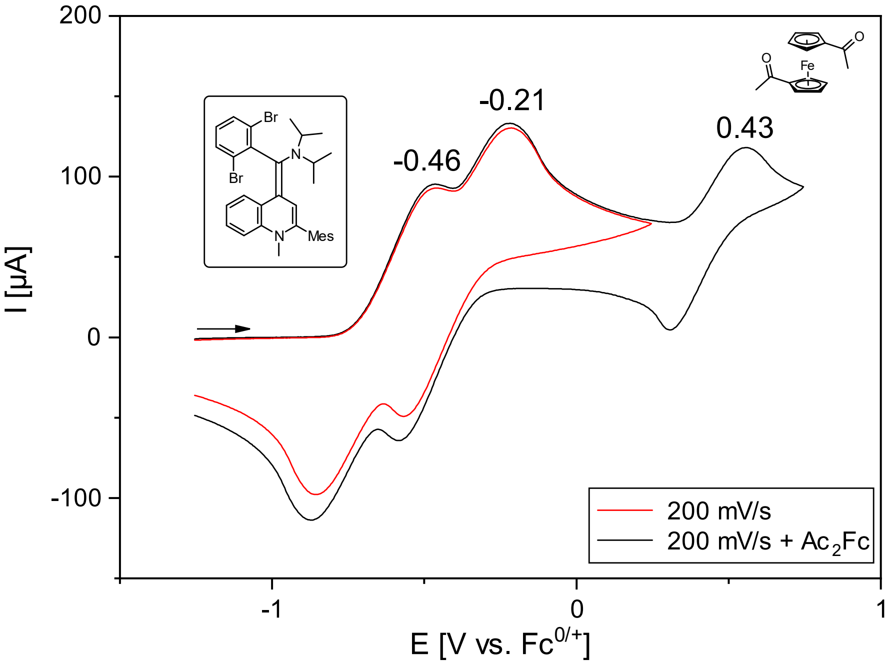

Figure S221: Cyclic voltammograms of **2f** (3 mg mL^{-1}) in thf (0.1 M n-Bu_4NPF_6) at room temperature; scan rate 200 mV s^{-1} (iR compensation = 2400 Ohm) referenced internally against diacetylferrocene (arrows indicate scanning direction). Diacetylferrocene was used as standard since ferrocene would overlap with the oxidation wave.

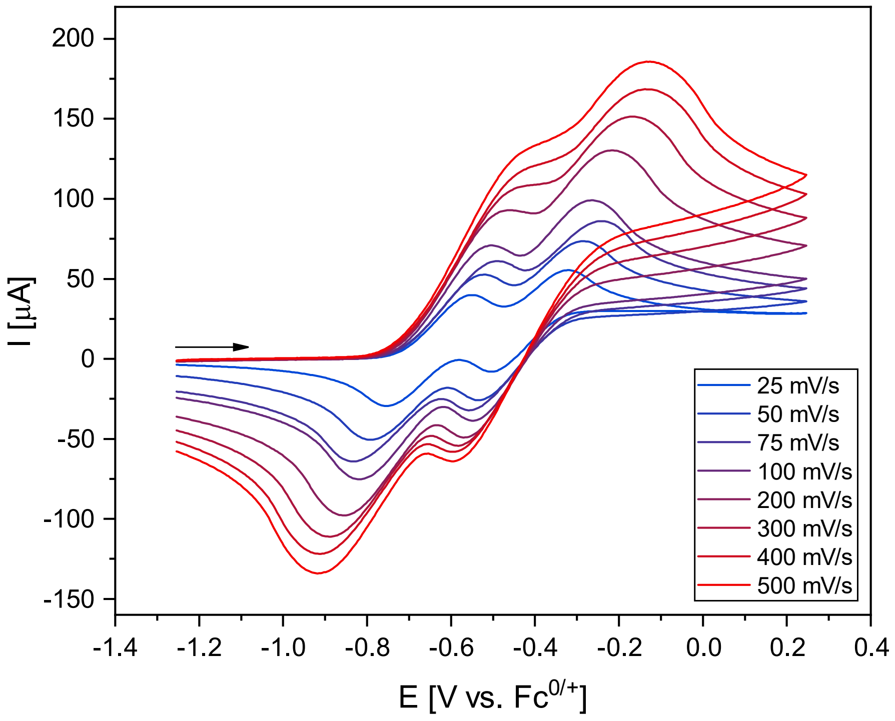

Figure S222: Cyclic voltammograms of **2f** (3 mg mL^{-1}) in thf (0.1 M n-Bu_4NPF_6) at room temperature; different scan rates (iR compensation = 2400 Ohm) referenced internally against diacetylferrocene (arrows indicate scanning direction). Diacetylferrocene was used as standard since ferrocene would overlap with the oxidation wave.

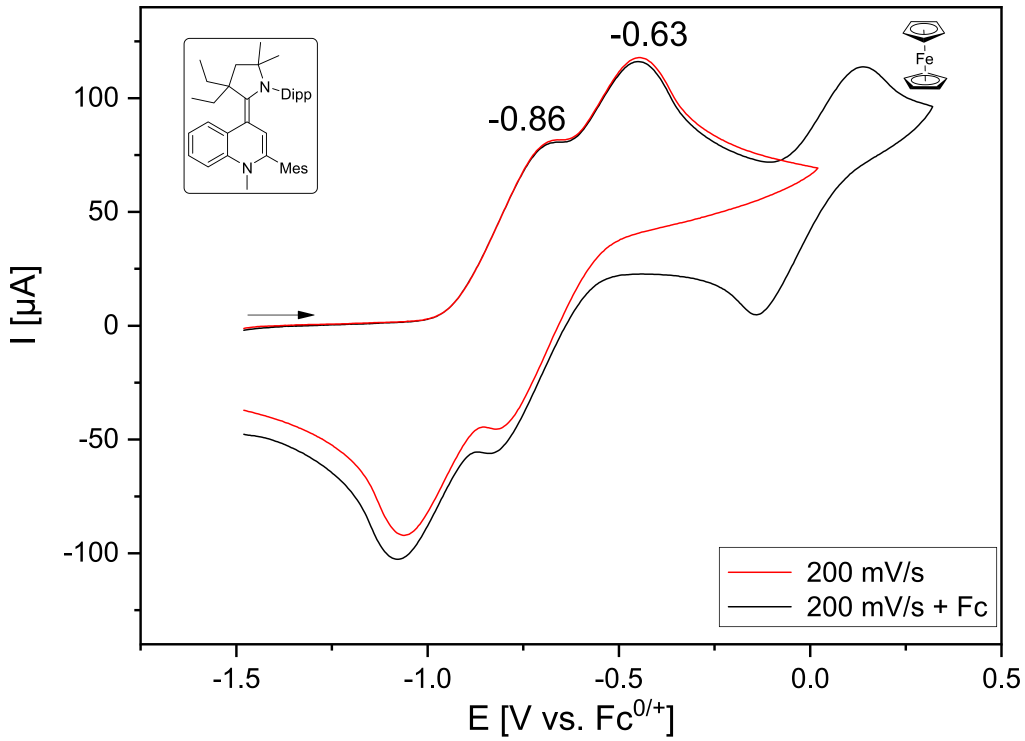

Figure S223: Cyclic voltammograms of **2g** (2.7±0.1 mg mL^{-1}) in thf (0.1 M n-Bu_4NPF_6) at room temperature; scan rate 200 mV s^{-1} (iR compensation = 1850 Ohm) referenced internally against ferrocene (arrows indicate scanning direction).

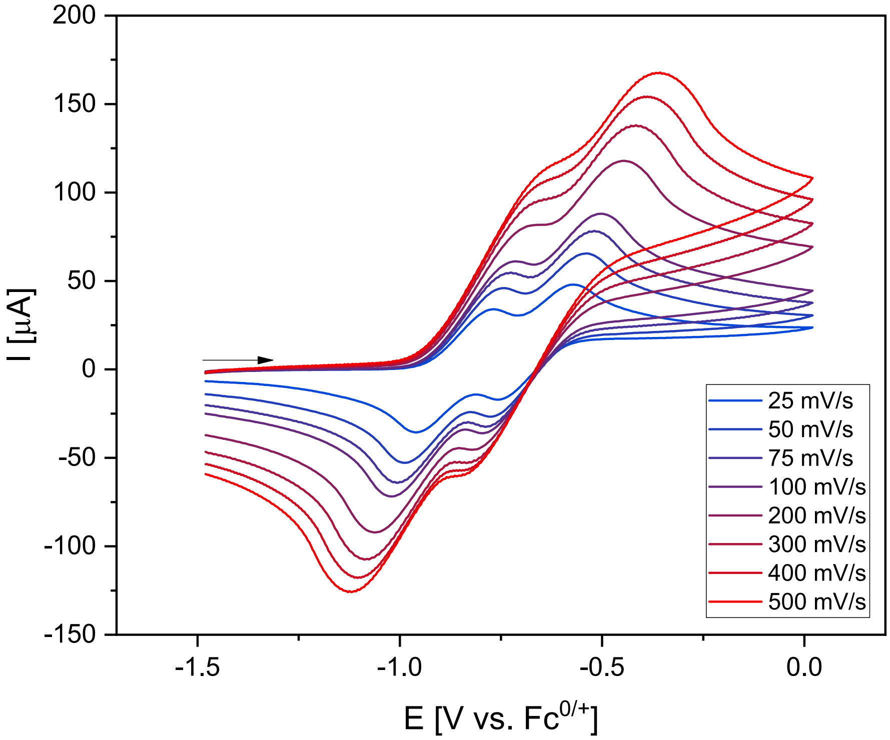

Figure S224: Cyclic voltammograms of **2g** (2.7±0.1 mg mL^{-1}) in thf (0.1 M n-Bu_4NPF_6) at room temperature; different scan rates (iR compensation = 1850 Ohm) referenced internally against ferrocene (arrows indicate scanning direction).

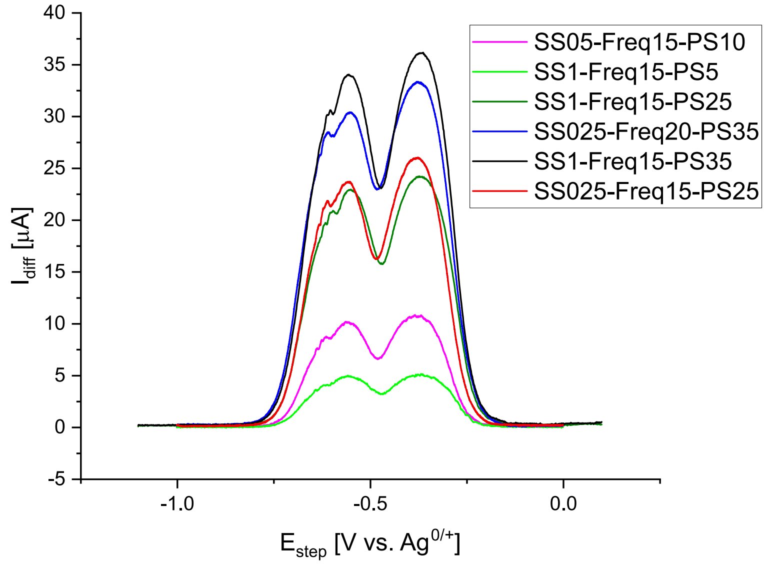

Figure S225: Square wave voltammetry of **2g** (2.7±0.1 mg mL^{-1}) in thf (0.1 M n-Bu_4NPF_6) at room temperature; Experimental parameters: iR compensation = 1850 Ohm; SS: step size [mV]; Freq: Frequency [Hz]; PS: Pulse Size [mV].

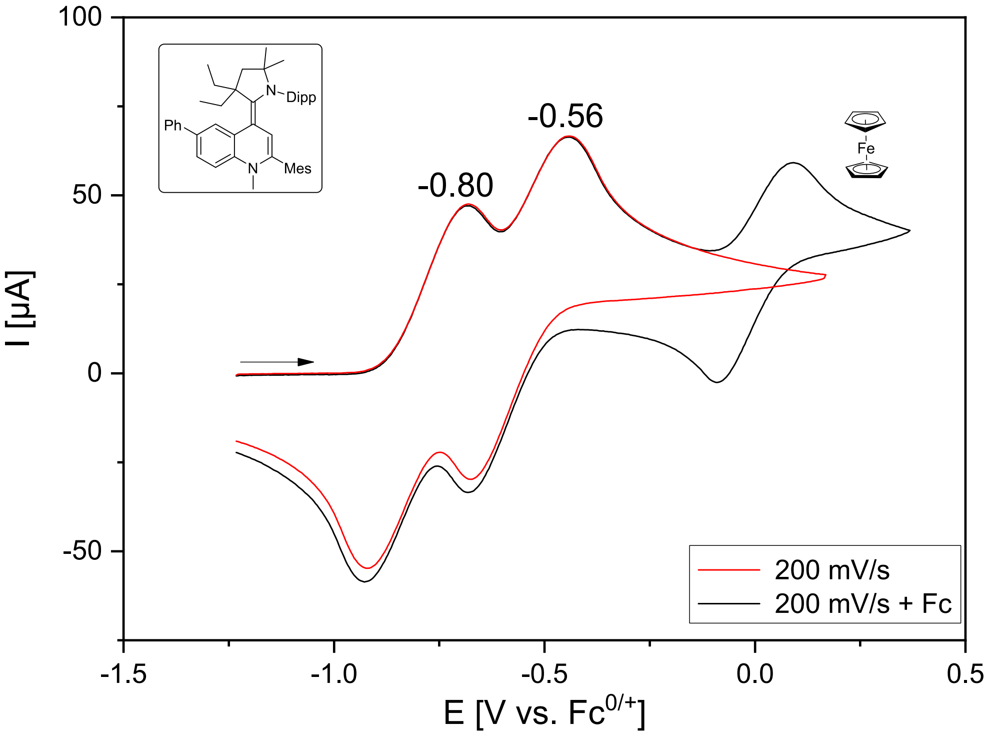

Figure S226: Cyclic voltammograms of **2h** (1.7±0.1 mg mL^{-1}) in thf (0.1 M n-Bu_4NPF_6) at room temperature; scan rate 200 mV s^{-1} (iR compensation = 2000 Ohm) referenced internally against ferrocene (arrows indicate scanning direction).

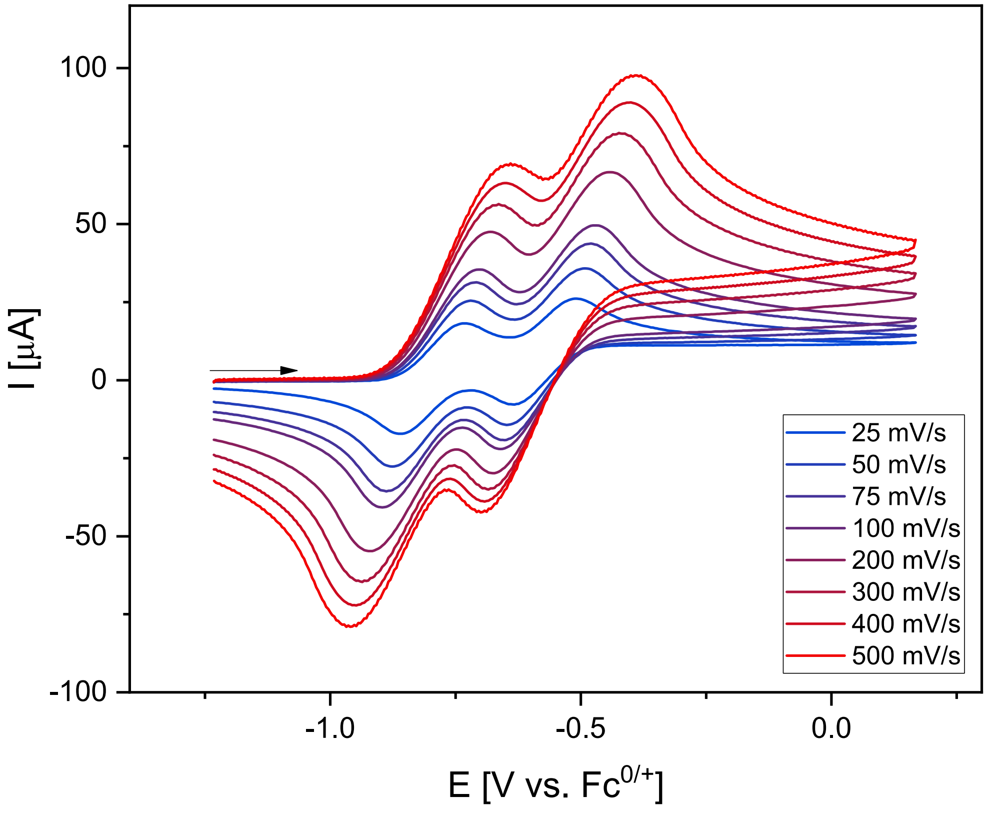

Figure S227: Cyclic voltammograms of **2h** (1.7±0.1 mg mL^{-1}) in thf (0.1 M n-Bu_4NPF_6) at room temperature; different scan rates (iR compensation = 2000 Ohm) referenced internally against ferrocene (arrows indicate scanning direction).

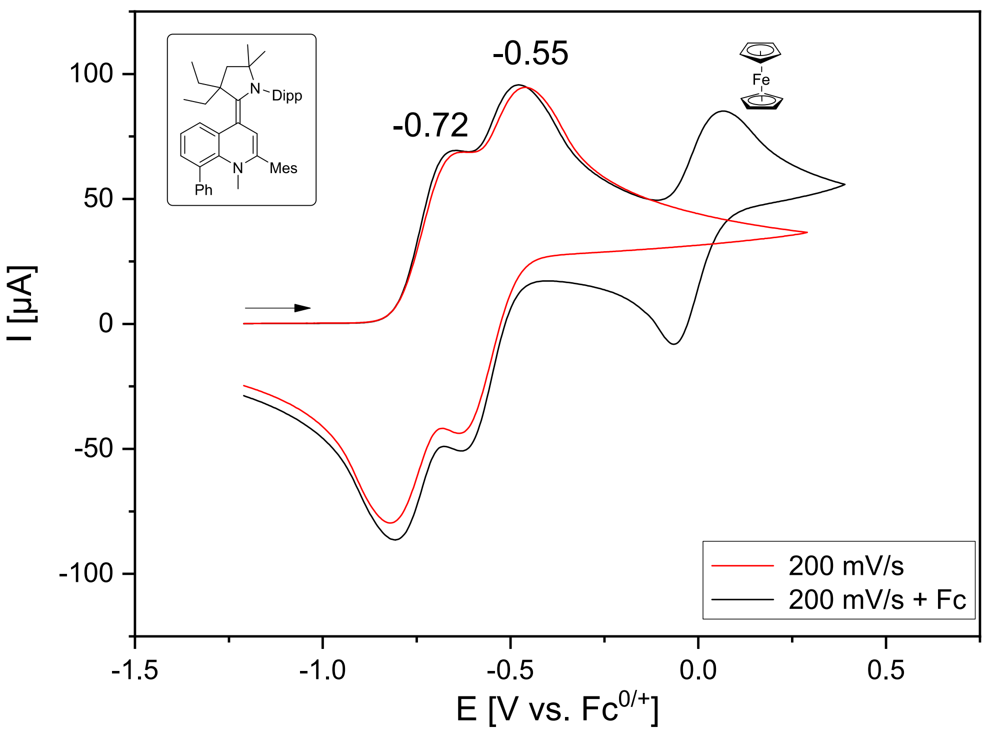

Figure S228: Cyclic voltammogram of **2i** (2.2±0.1 mg mL^{-1}) in thf (0.1 M *n*-Bu_4NPF_6) at room temperature; scan rate 200 mV s^{-1} (iR compensation = 2300 Ohm) referenced internally against ferrocene (arrows indicate scanning direction).

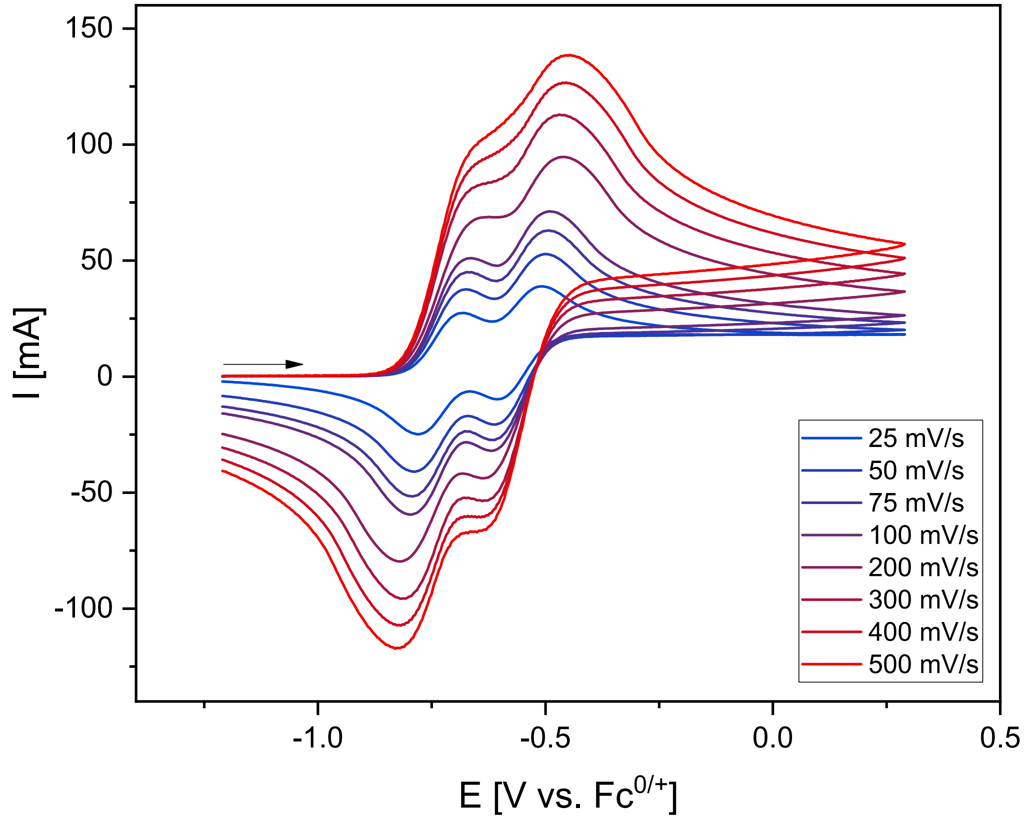

Figure S229: Cyclic voltammogram of **2i** (2.2±0.1 mg mL^{-1}) in thf (0.1 M *n*-Bu_4NPF_6) at room temperature; scan rate 200 mV s^{-1} (iR compensation = 2300 Ohm) referenced internally against ferrocene (arrows indicate scanning direction).

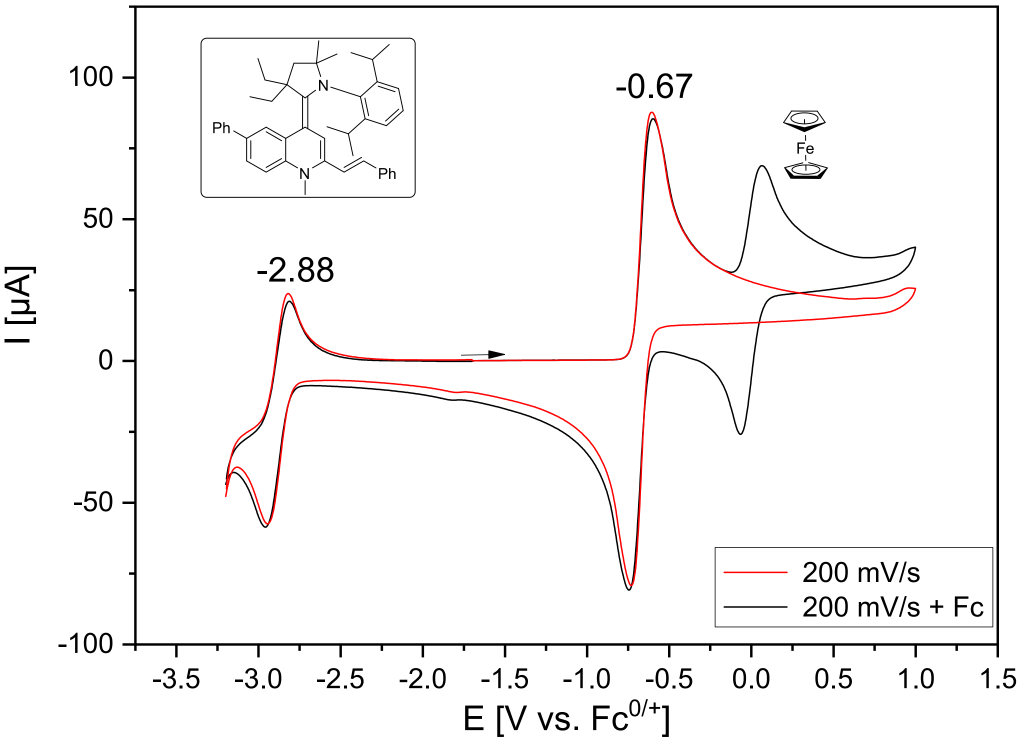

Figure S230: Cyclic voltammograms of **2j** (1.4±0.1 mg mL^{-1}) in thf (0.1 M n-Bu_4NPF_6) at room temperature; scan rate 200 mV s^{-1} (iR compensation = 2100 Ohm) referenced internally against ferrocene (arrows indicate scanning direction).

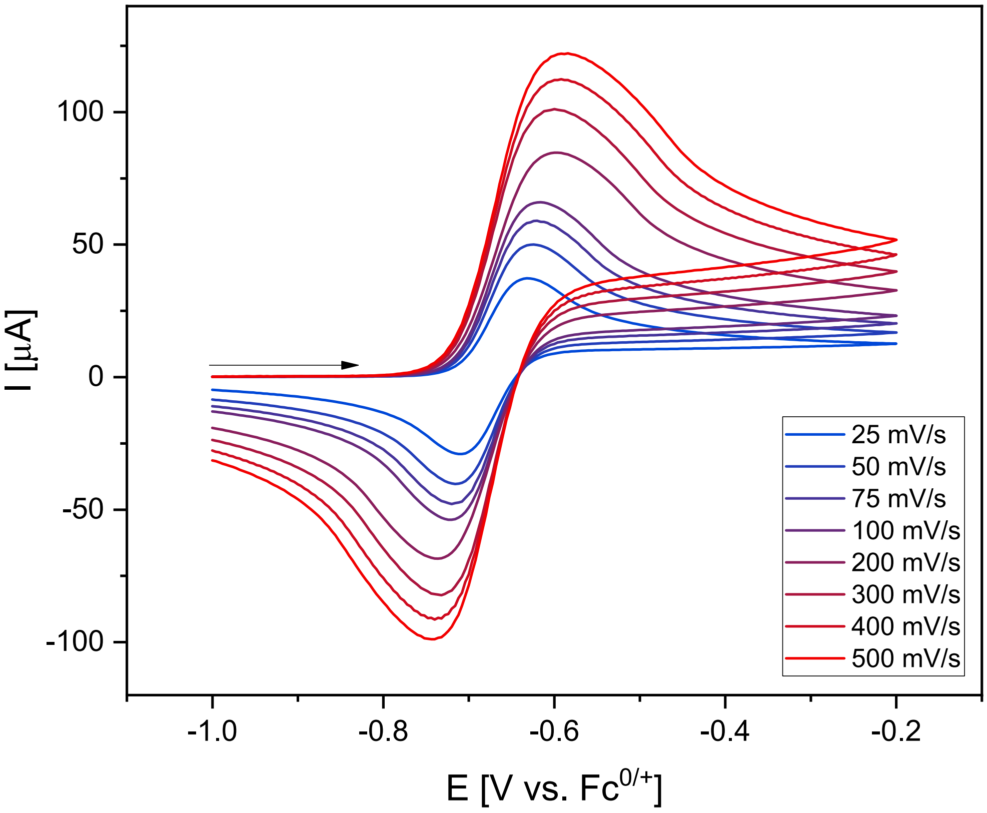

Figure S231: Cyclic voltammograms of **2j** (1.4±0.1 mg mL^{-1}) in thf (0.1 M n-Bu_4NPF_6) at room temperature; different scan rates (iR compensation = 2100 Ohm) referenced internally against ferrocene (arrows indicate scanning direction).

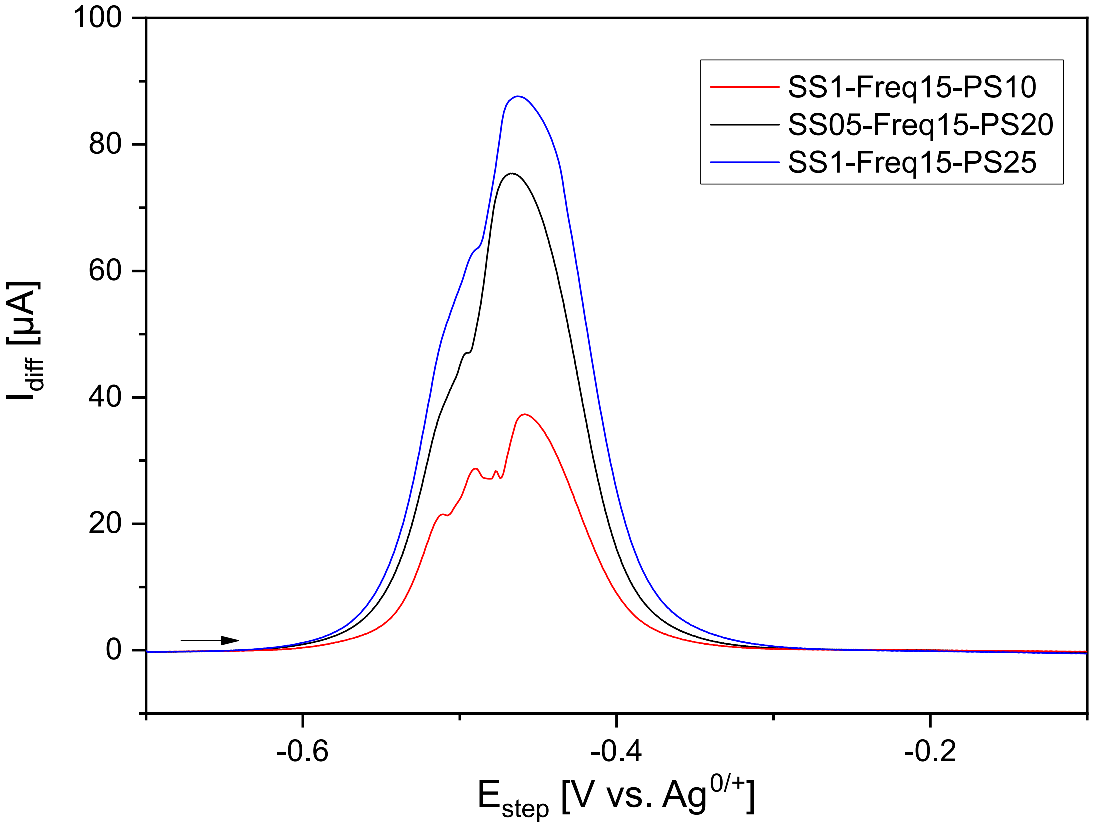

Figure S232: Square wave voltammetry of **2j** (1.4±0.1 mg mL^{-1}) in thf (0.1 M n-Bu_4NPF_6) at room temperature; Experimental parameters: iR compensation = 2100 Ohm; SS: step size [mV]; Freq: Frequency [Hz]; PS: Pulse Size [mV].

UV-vis Spectroscopy

UV-Vis spectra were recorded on an Agilent Cary60. If not stated differently 0.1 cm quartz cuvettes were used. All samples were prepared in a nitrogen filled glove-box.

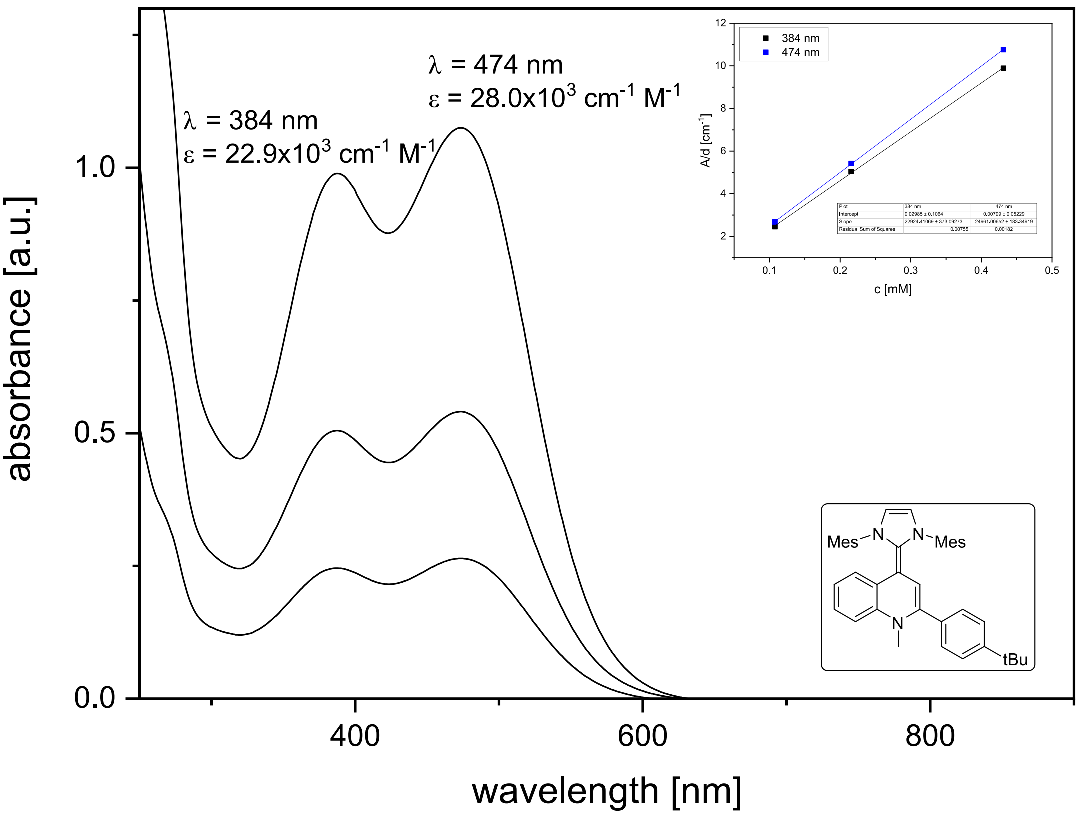

Figure S233: UV-vis spectra of **2a** in thf, measured 0.1 cm quartz cuvettes.

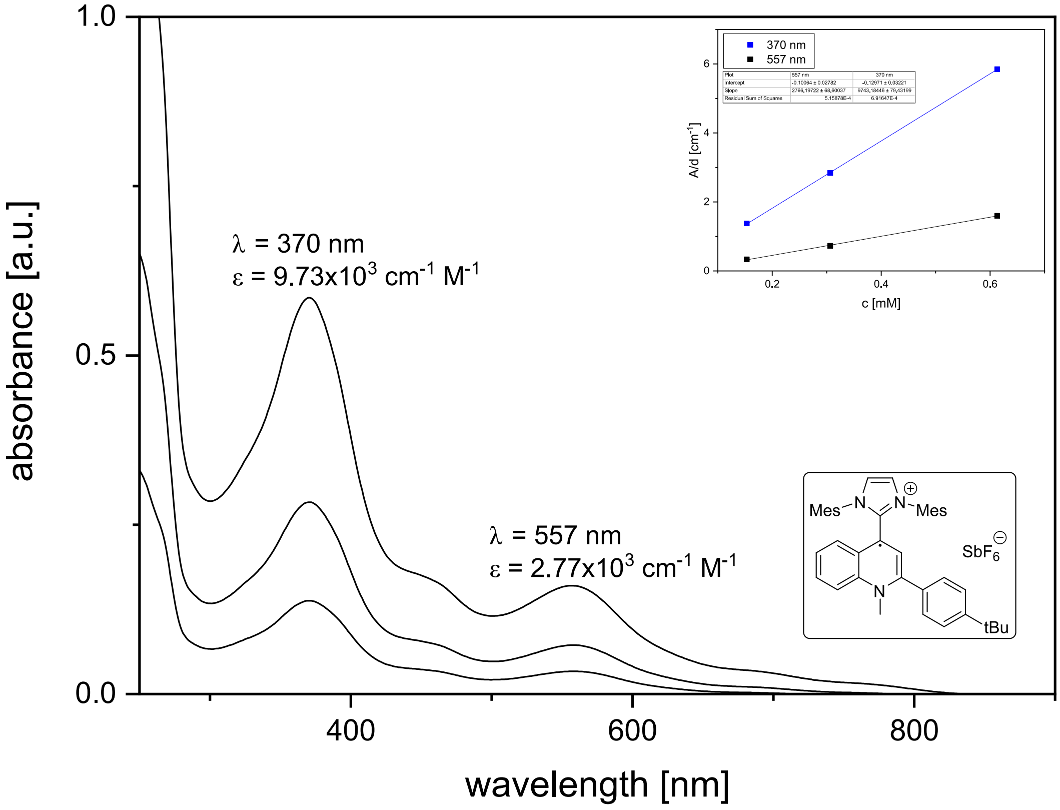

Figure S2334: UV-vis spectra of **3a** in thf, measured 0.1 cm quartz cuvettes.

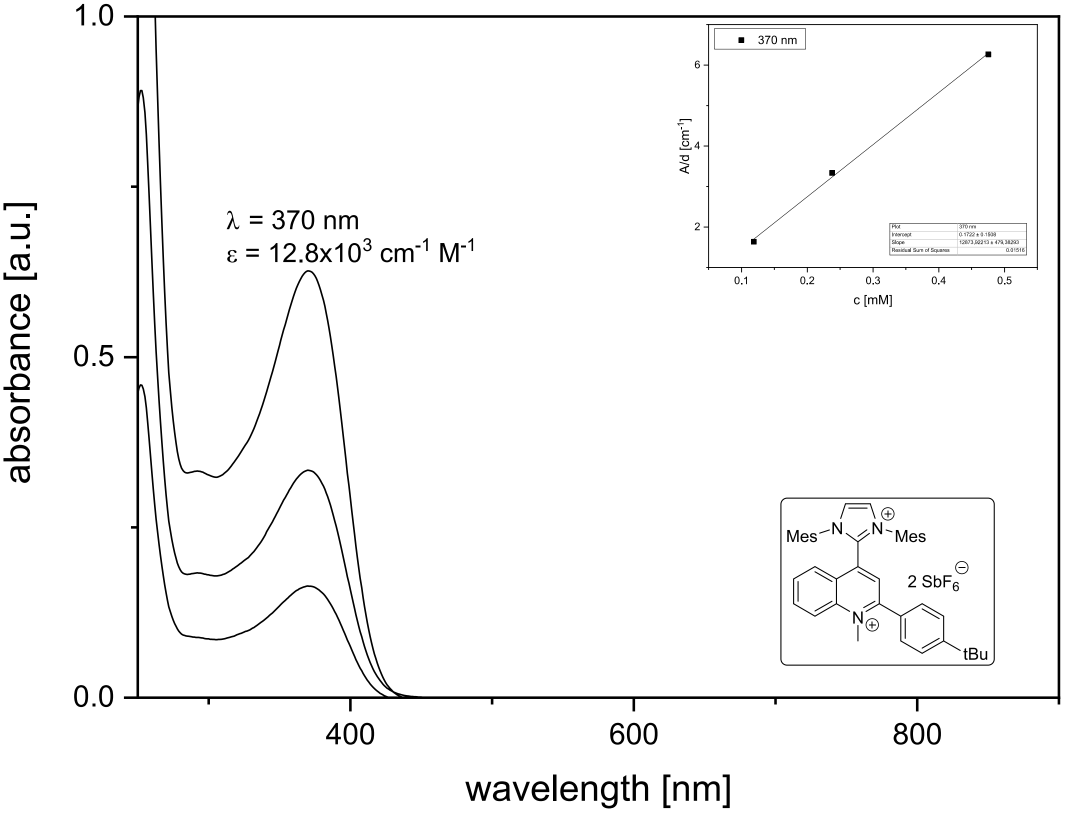

Figure S235: UV-vis spectra of **4a** in thf, measured 0.1 cm quartz cuvettes.

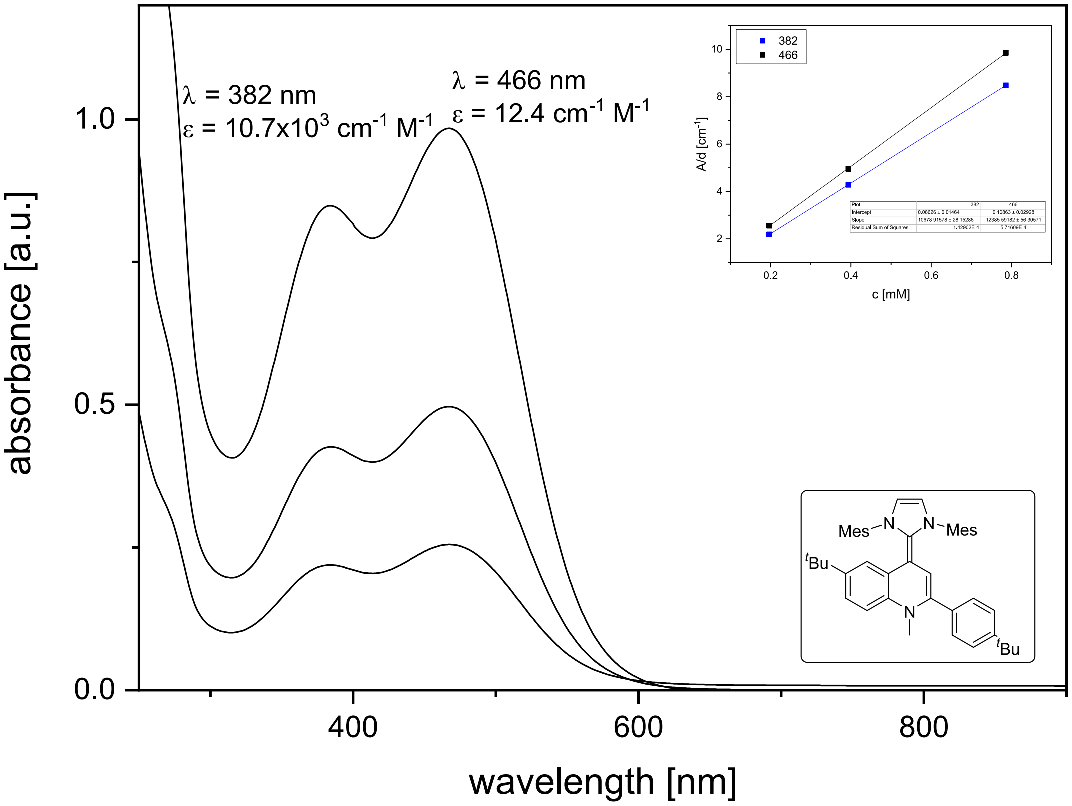

Figure S236: UV-vis spectra of **2b** in thf, measured 0.1 cm quartz cuvettes.

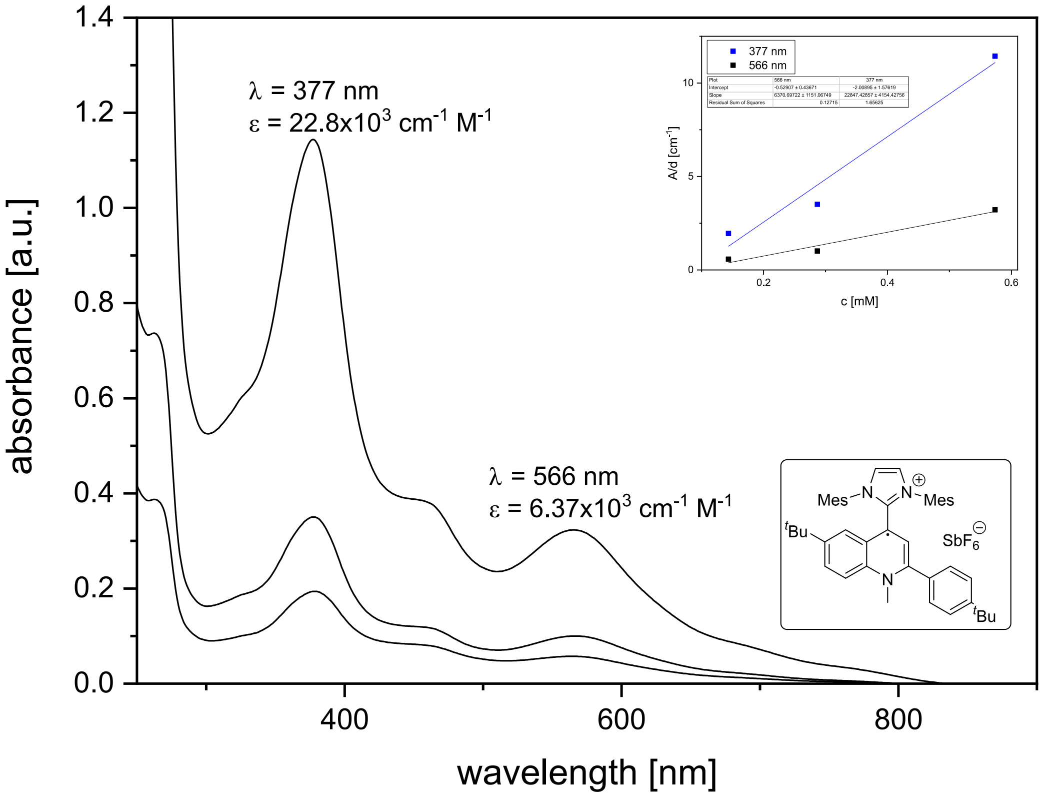

Figure S237: UV-vis spectra of **3b** in thf, measured 0.1 cm quartz cuvettes.

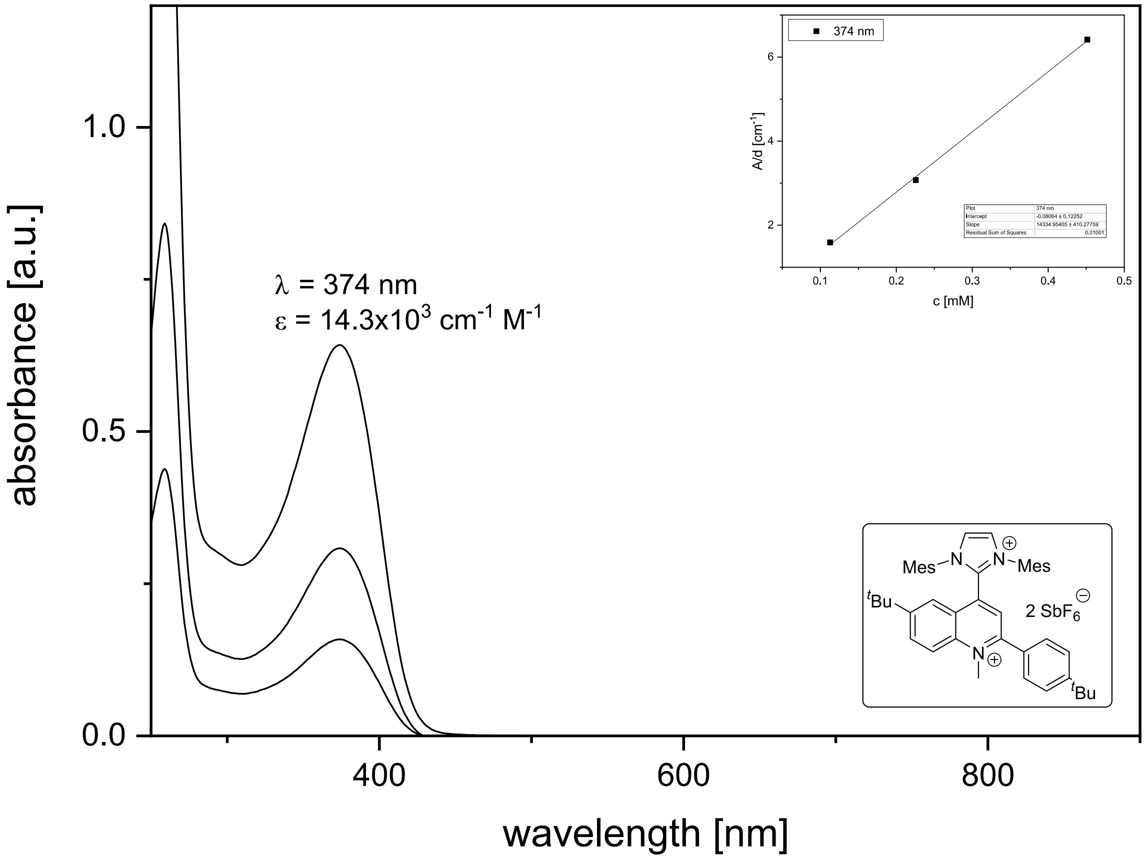

Figure S238: UV-vis spectra of **4b** in thf, measured 0.1 cm quartz cuvettes.

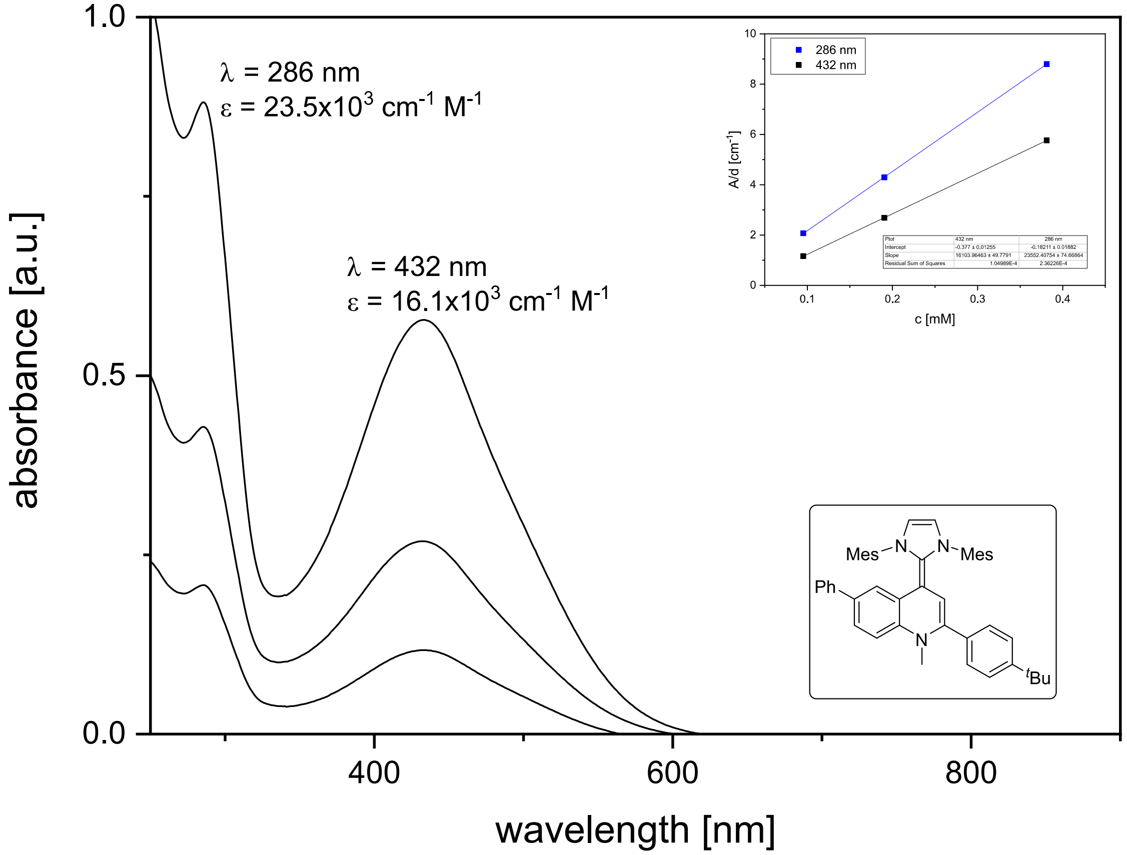

Figure S239: UV-vis spectra of **2c** in thf, measured 0.1 cm quartz cuvettes.

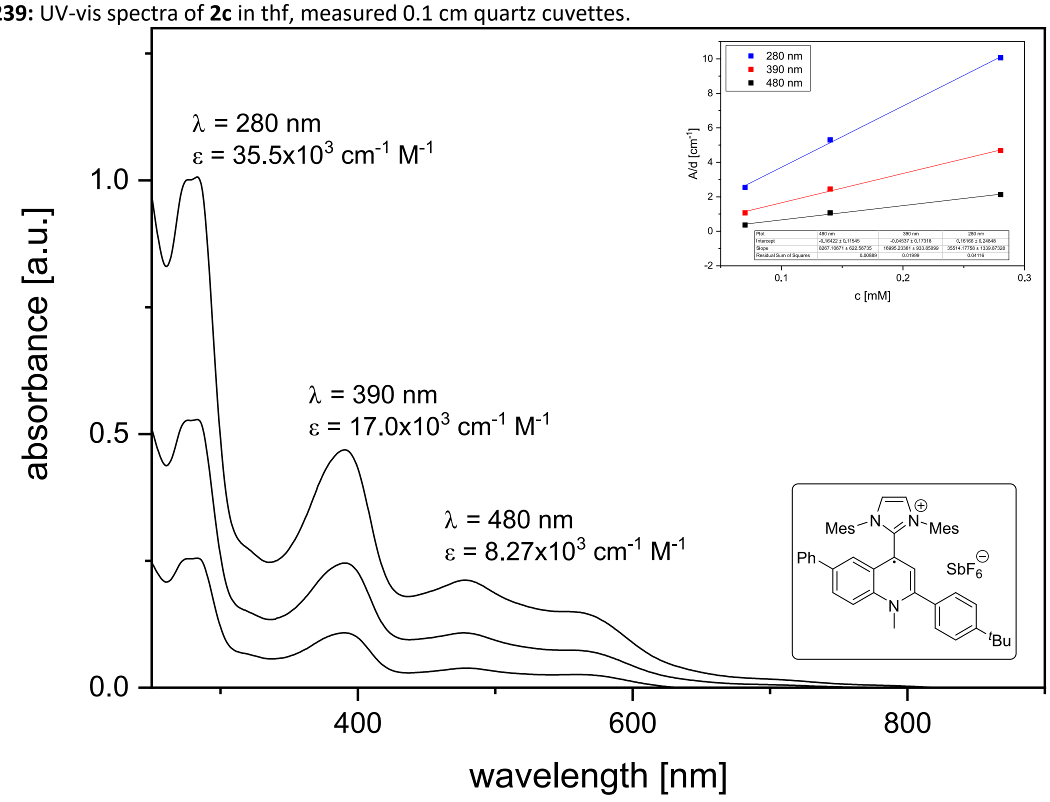

Figure S240: UV-vis spectra of **3c** in thf, measured 0.1 cm quartz cuvettes.

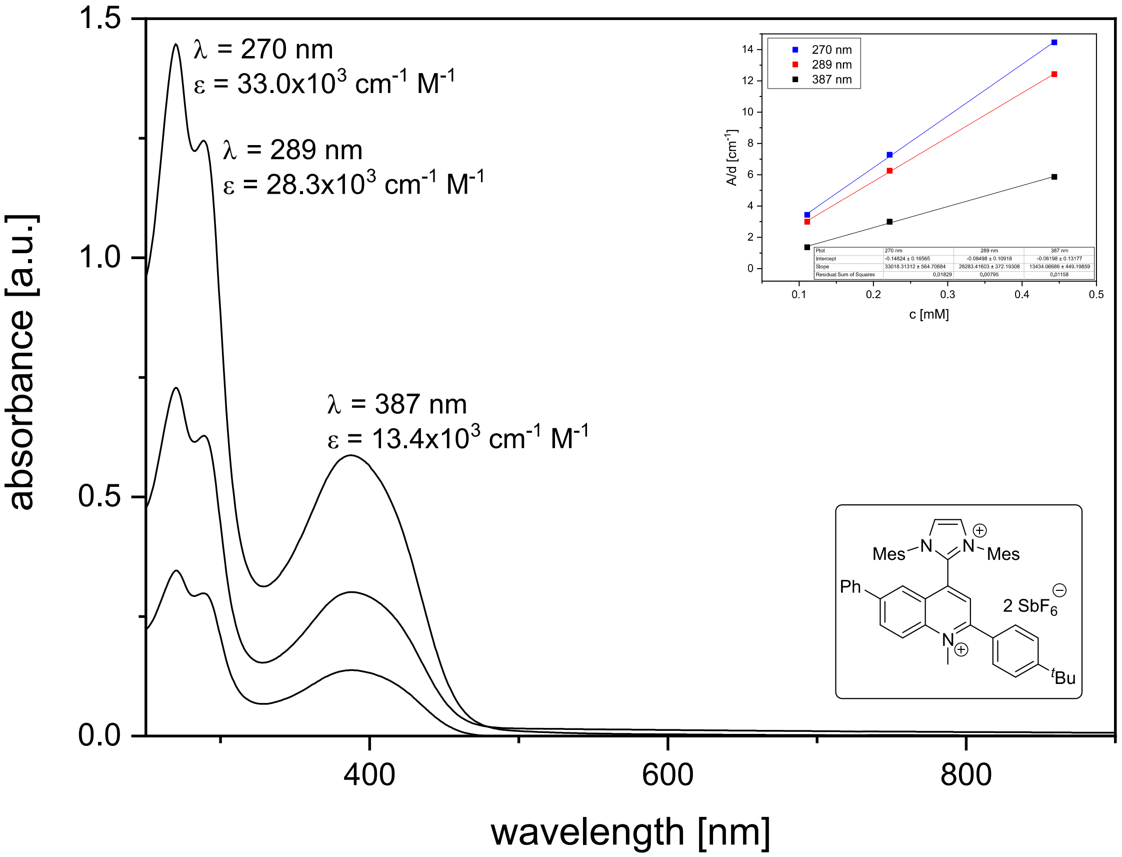

Figure S241: UV-vis spectra of **4c** in thf, measured 0.1 cm quartz cuvettes.

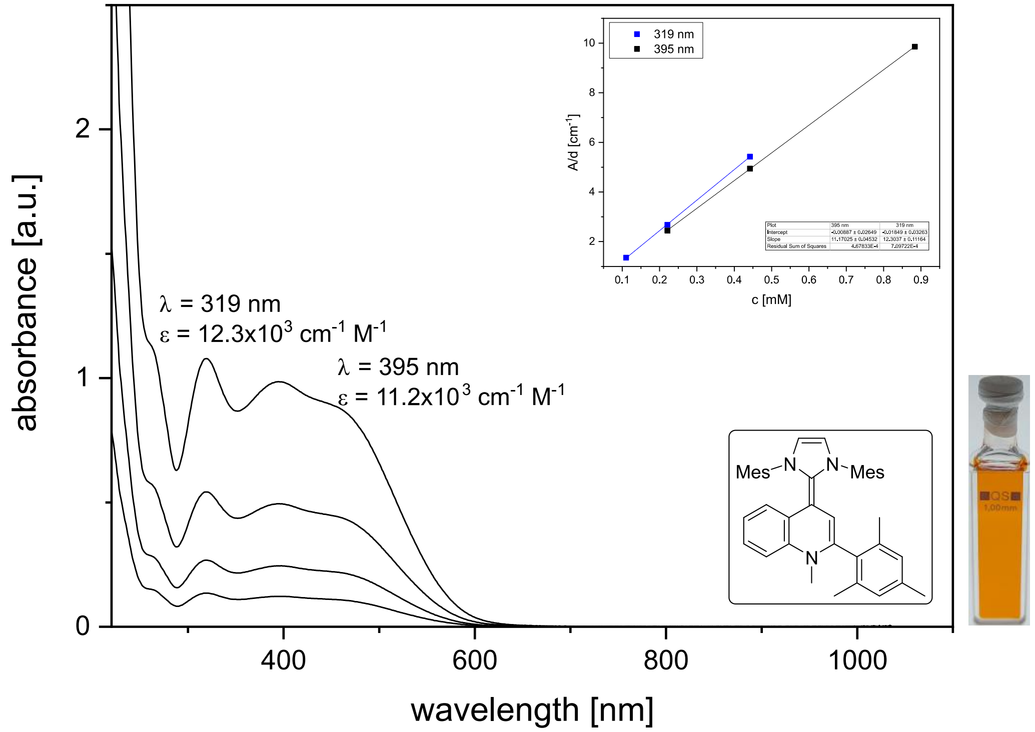

Figure S242: UV-vis spectra of **2d** in thf, measured 0.1 cm quartz cuvettes.

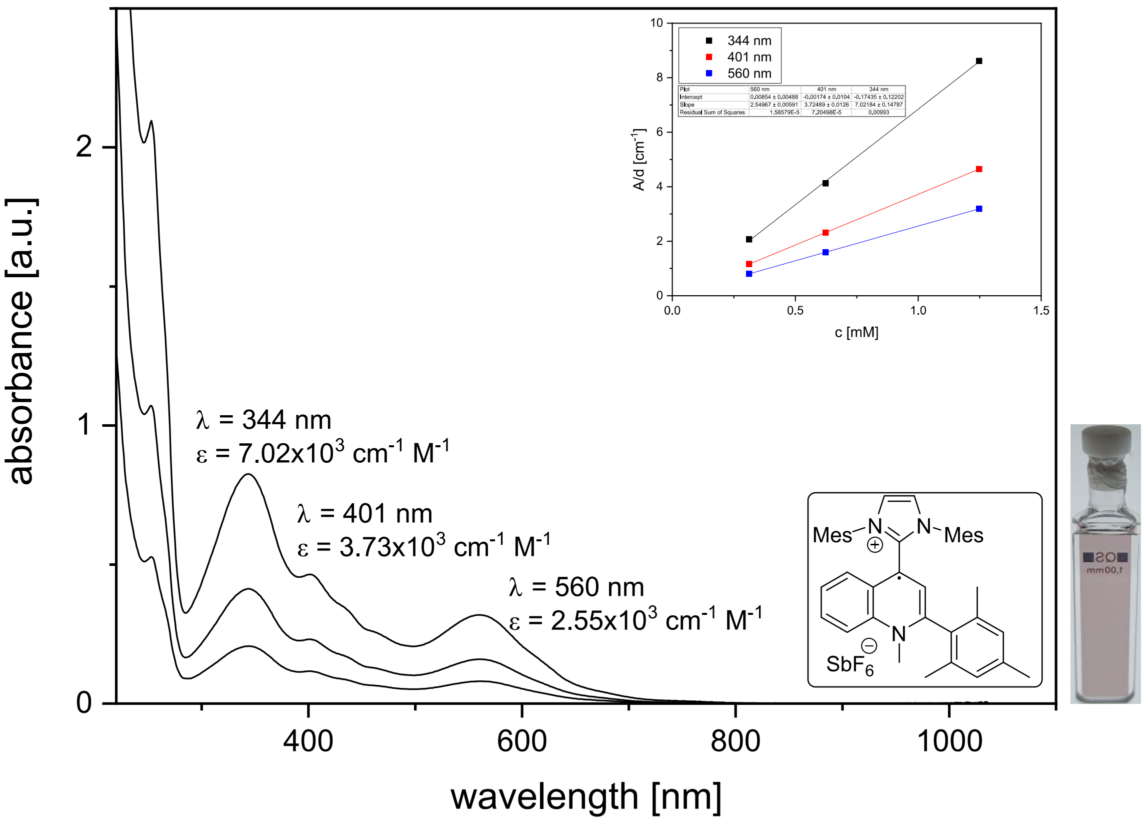

Figure S243: UV-vis spectra of **3d** in thf, measured 0.1 cm quartz cuvettes.

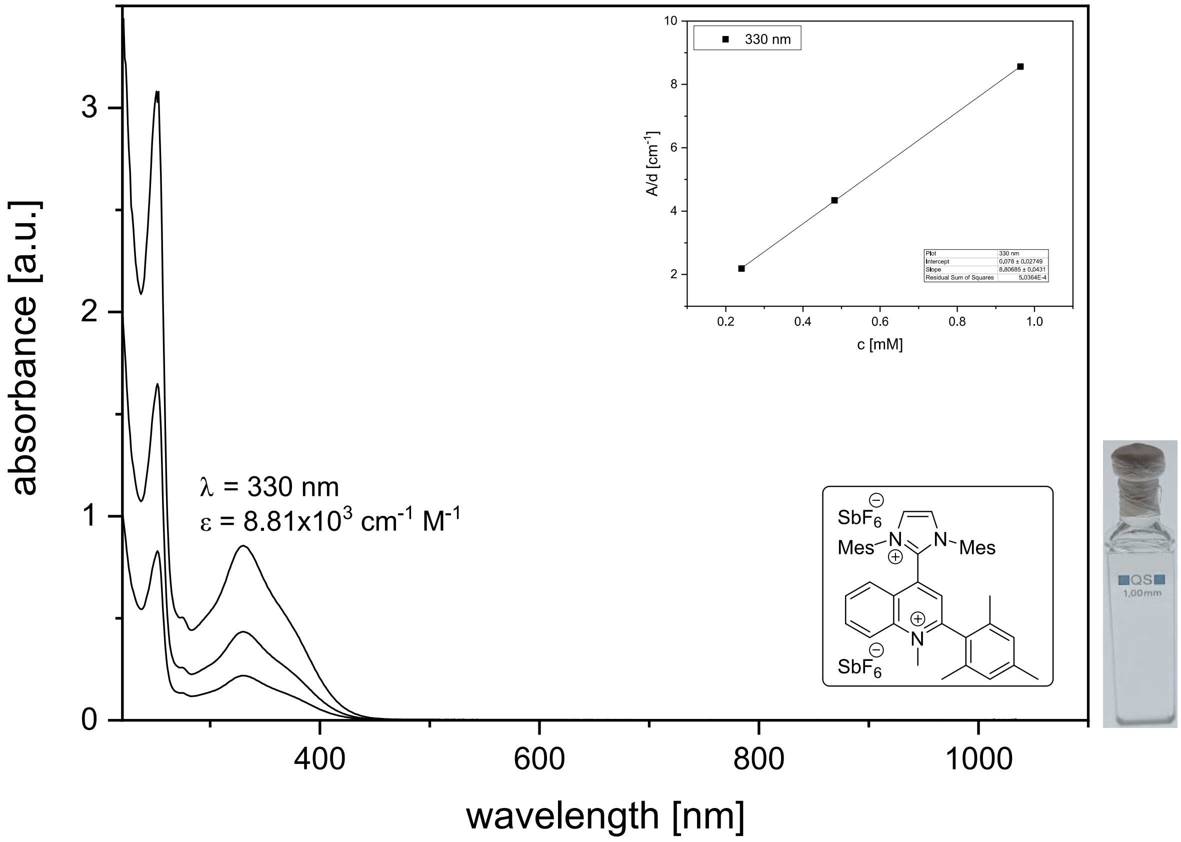

Figure S244: UV-vis spectra of **4d** in CH_3CN, measured 0.1 cm quartz cuvettes.

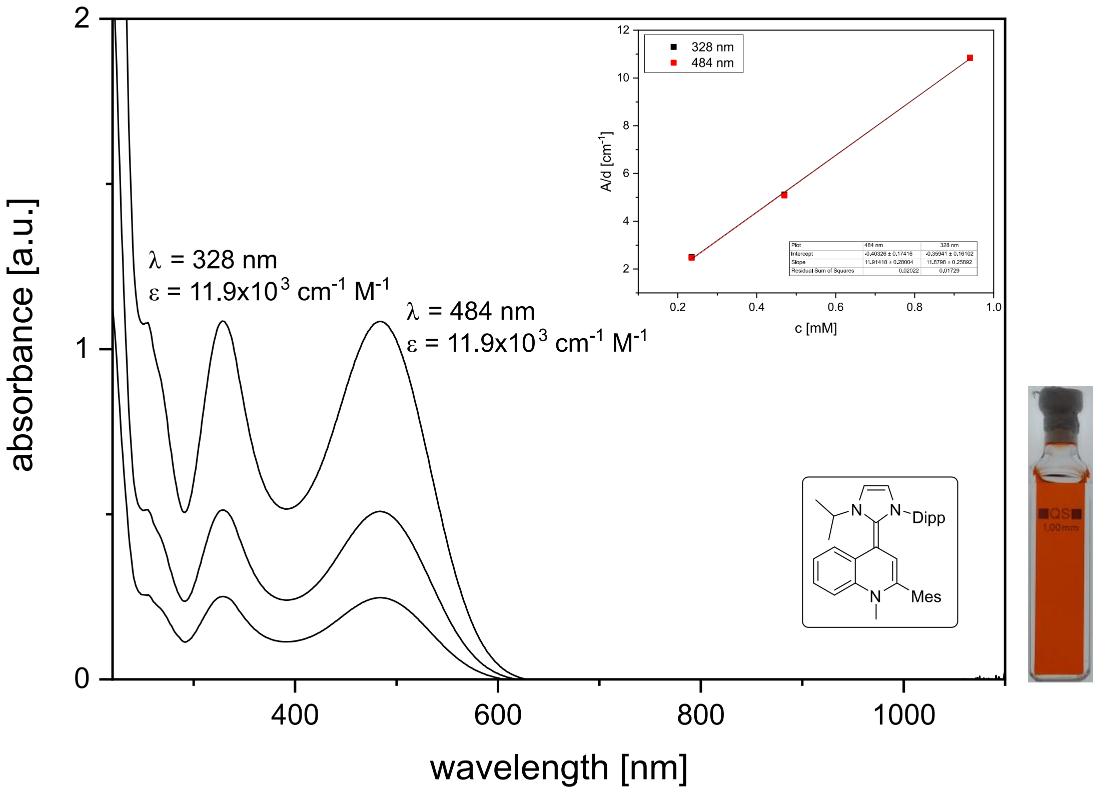

Figure S245: UV-vis spectra of **2e** in thf, measured 0.1 cm quartz cuvettes.

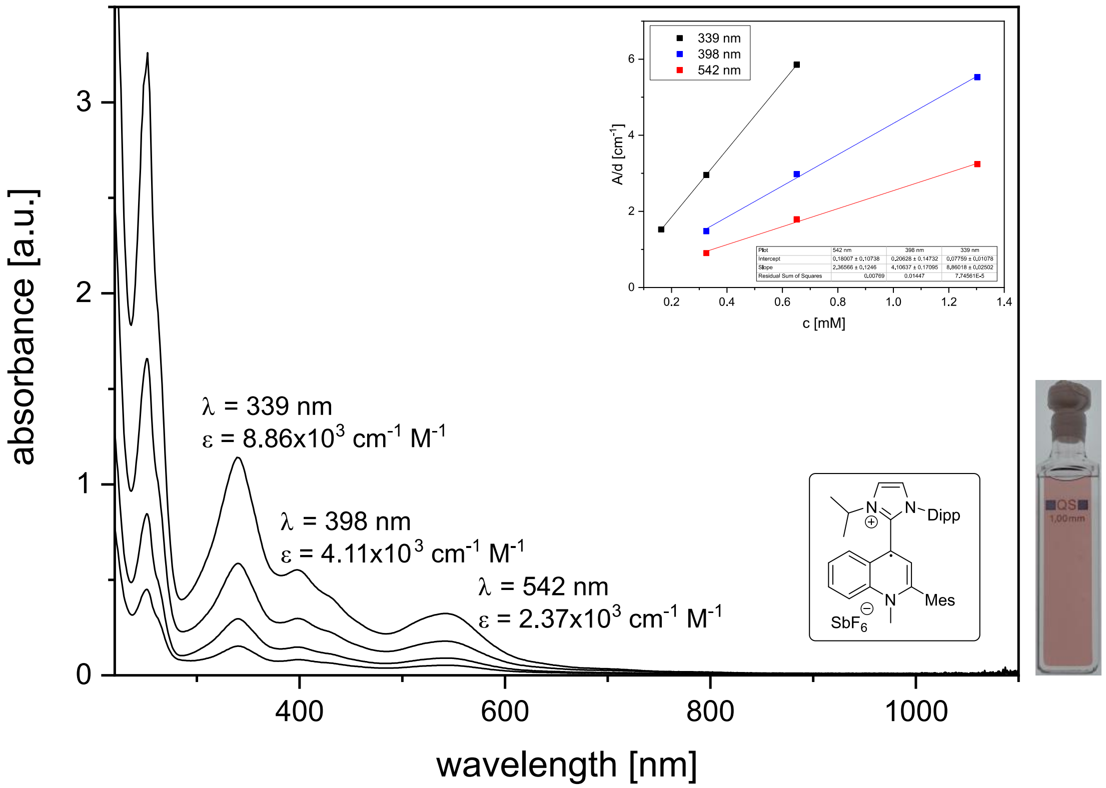

Figure S246: UV-vis spectra of **3e** in thf, measured 0.1 cm quartz cuvettes.

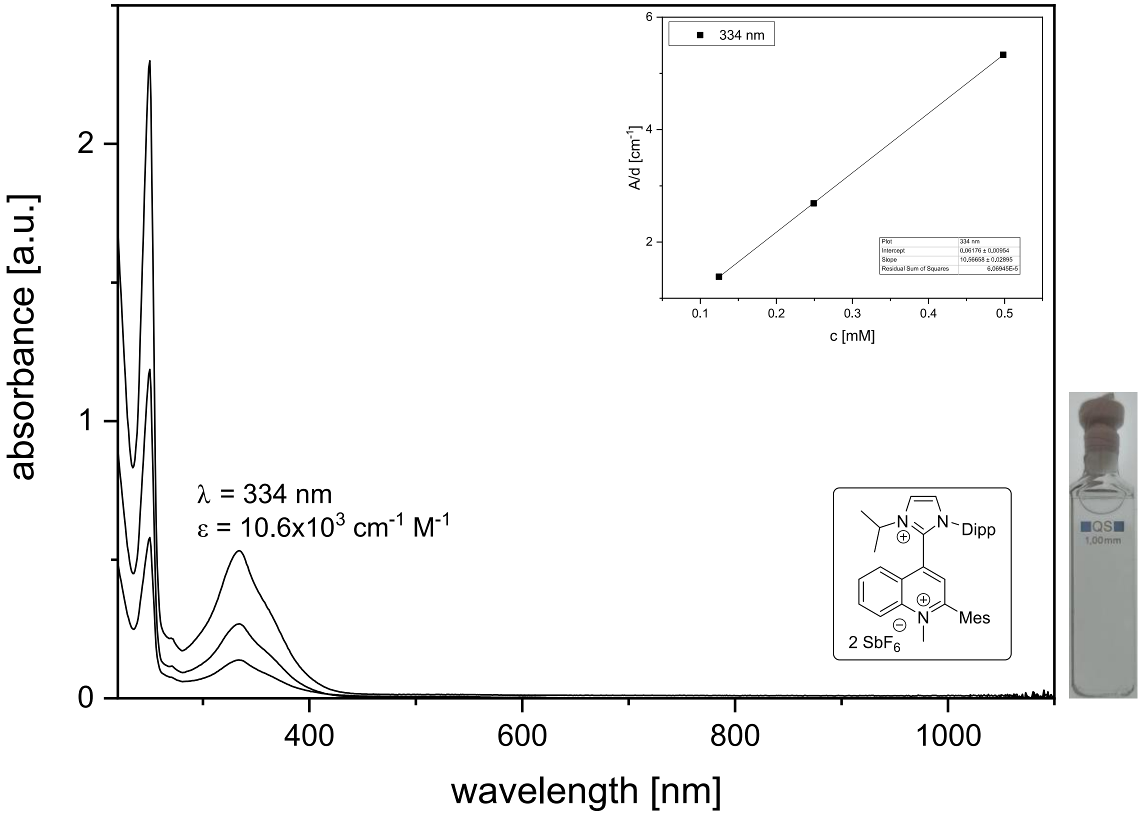

Figure S247: UV-vis spectra of **4e** in CH_3CN, measured 0.1 cm quartz cuvettes.

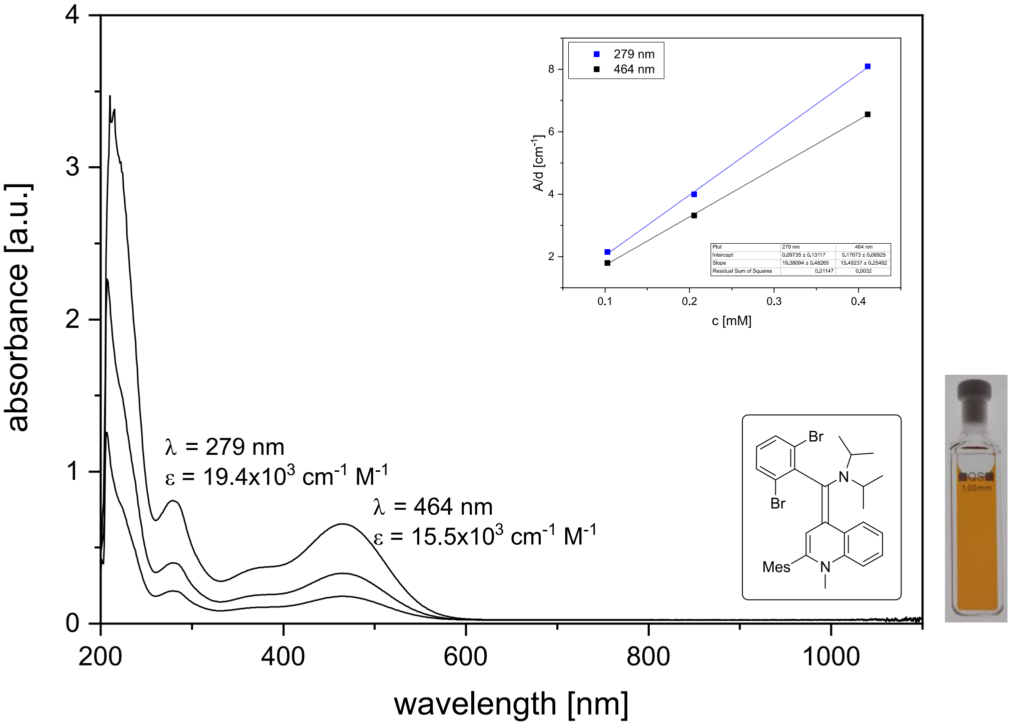

Figure S248: UV-vis spectra of **2f** in thf, measured in 0.1 cm quartz cuvettes.

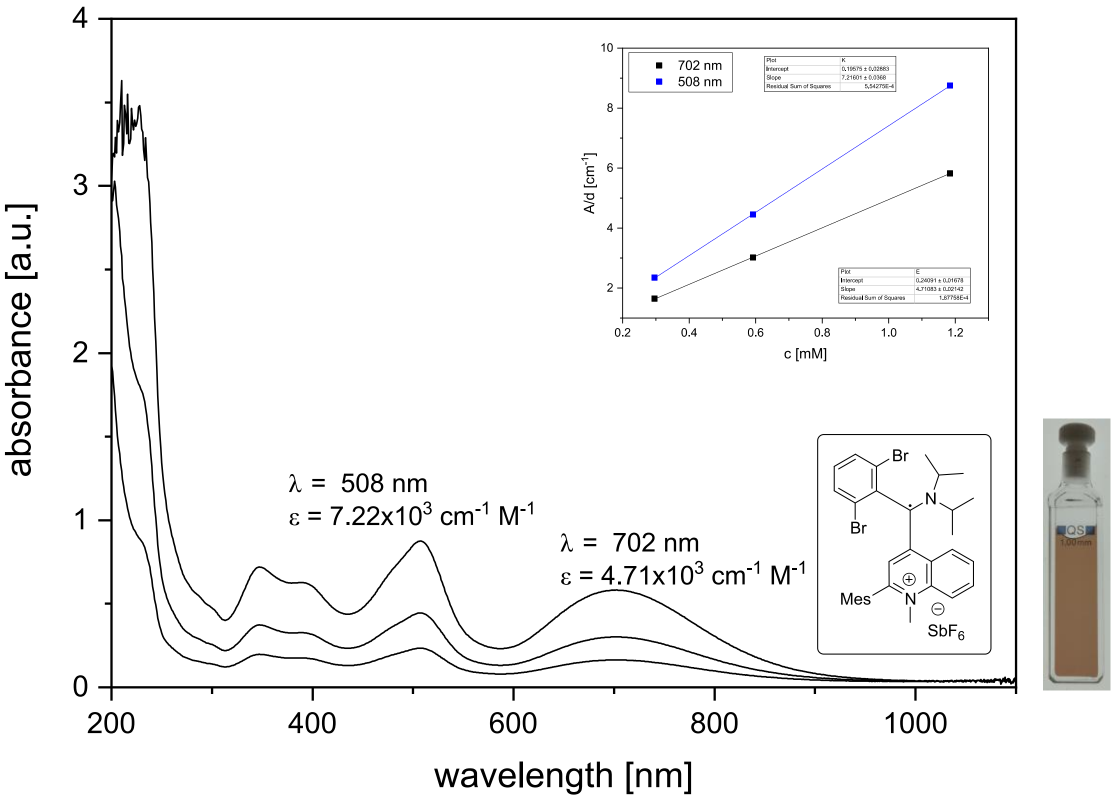

Figure S249: UV-vis spectra of **3f** in thf, measured in 0.1 cm quartz cuvettes.

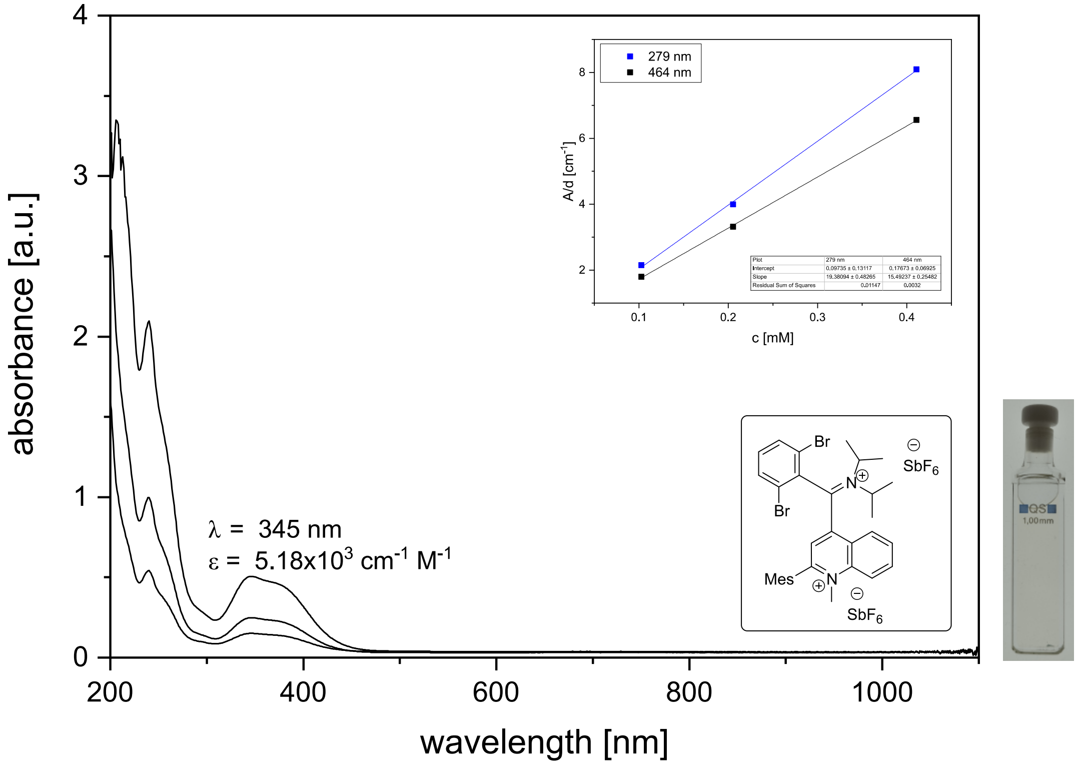

Figure S250: UV-vis spectra of **4f** in CH_3CN, measured in 0.1 cm quartz cuvettes.

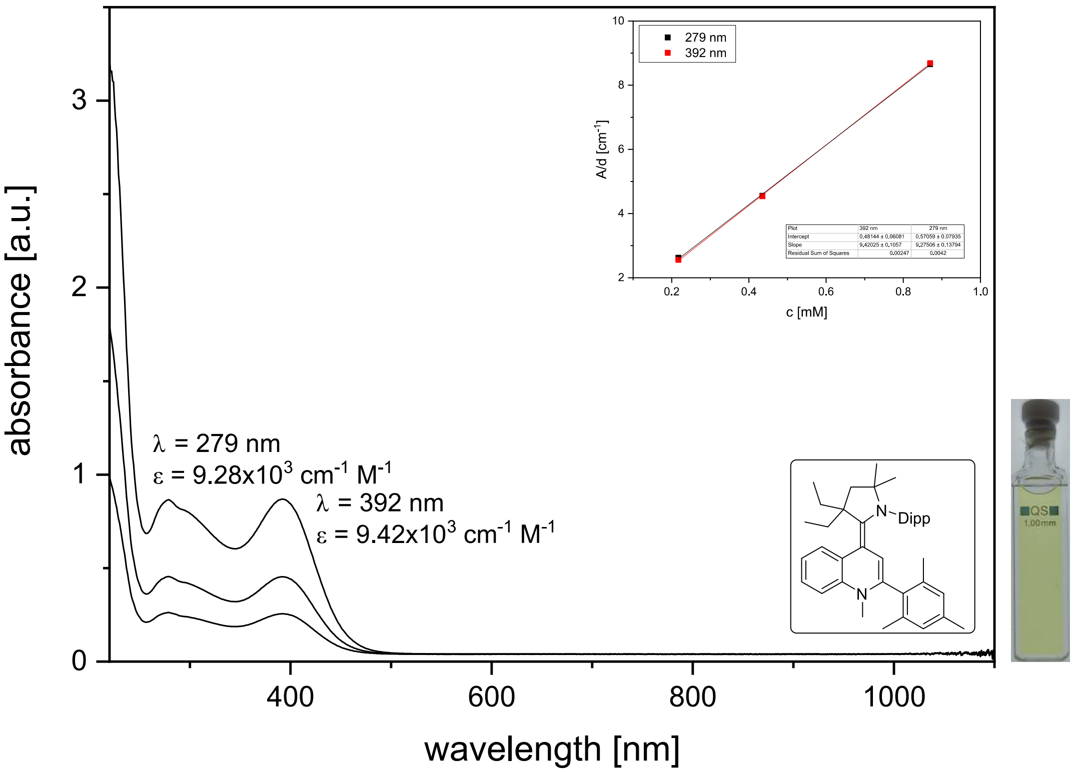

Figure S251: UV-vis spectra of **2g** in thf, measured 0.1 cm quartz cuvettes.

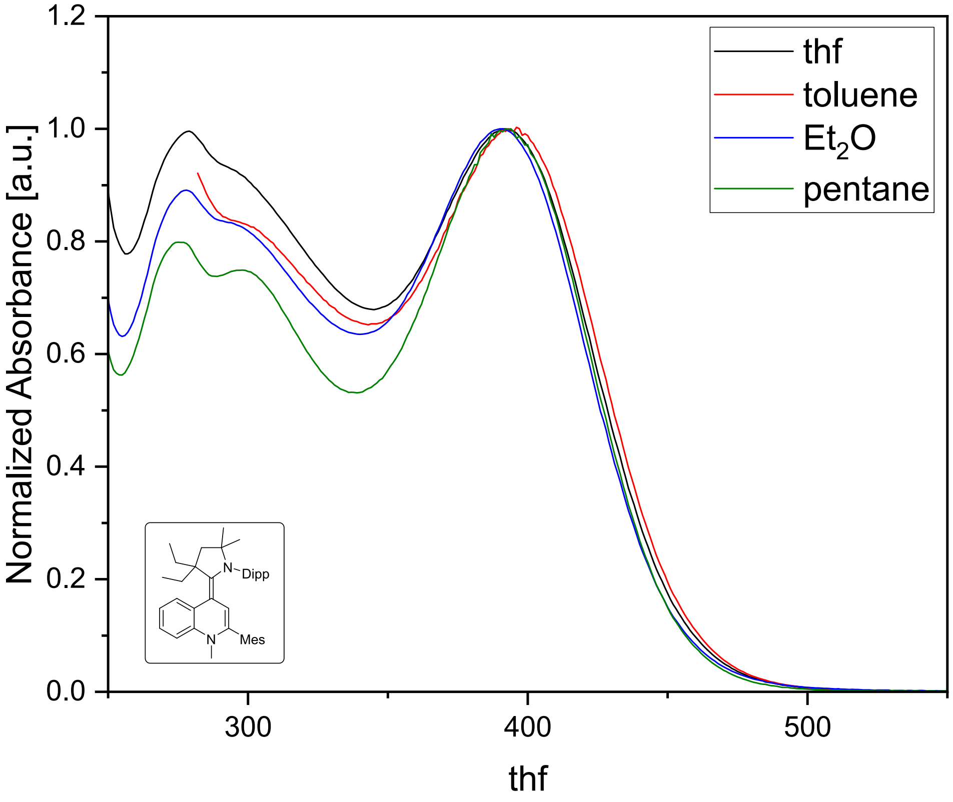

Figure S252: Normalized UV-vis spectra of **2g** in thf, toluene, Et_2O and pentane, measured 0.1 cm quartz cuvettes.

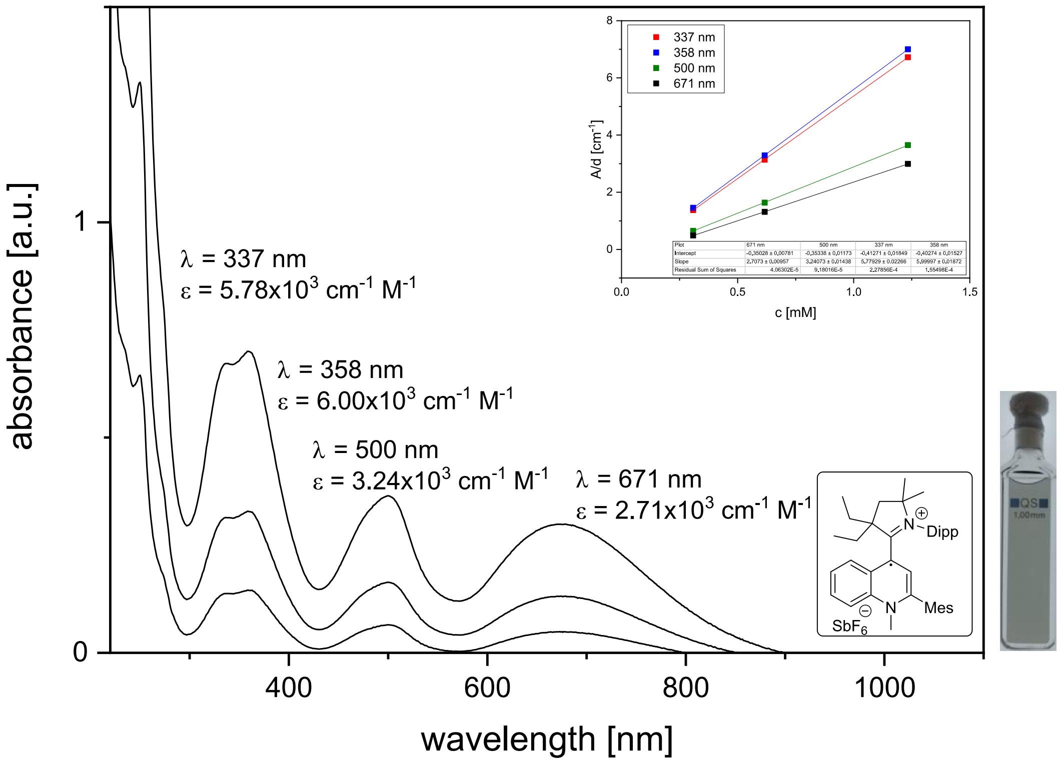

Figure S253: UV-vis spectra of **3g** in thf, measured 0.1 cm quartz cuvettes.

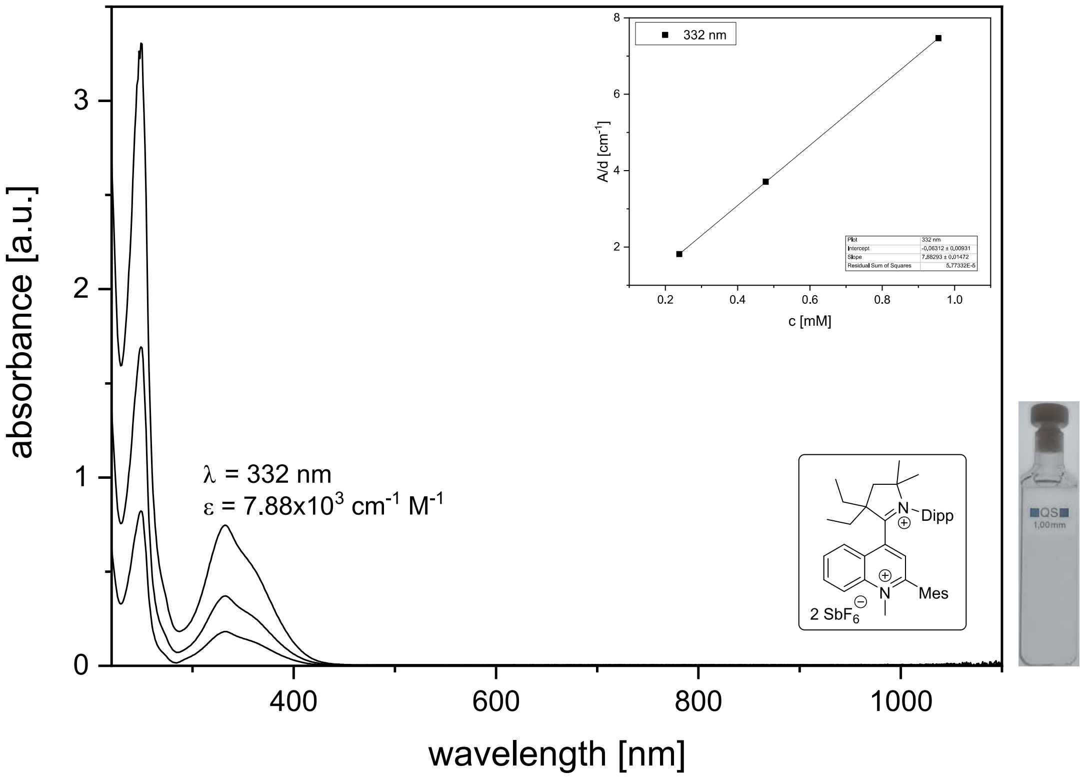

Figure S254: UV-vis spectra of **4g** in CH_3CN, measured 0.1 cm quartz cuvettes.

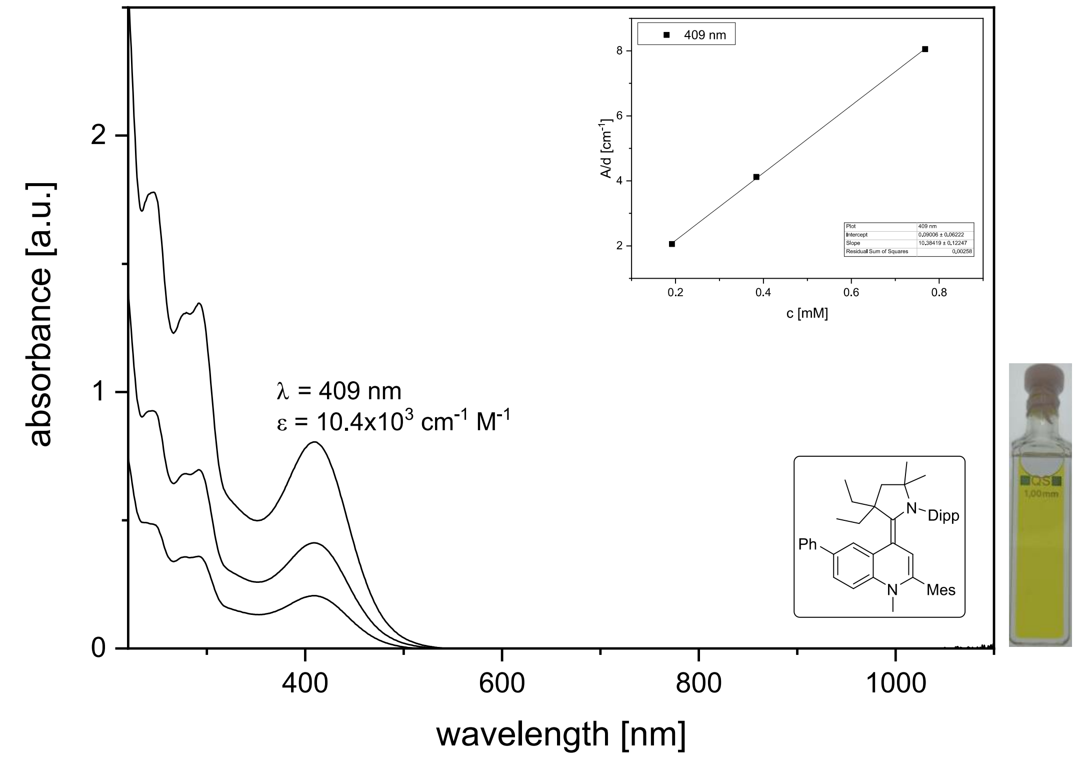

Figure S255: UV-vis spectra of **2h** in thf, measured 0.1 cm quartz cuvettes.

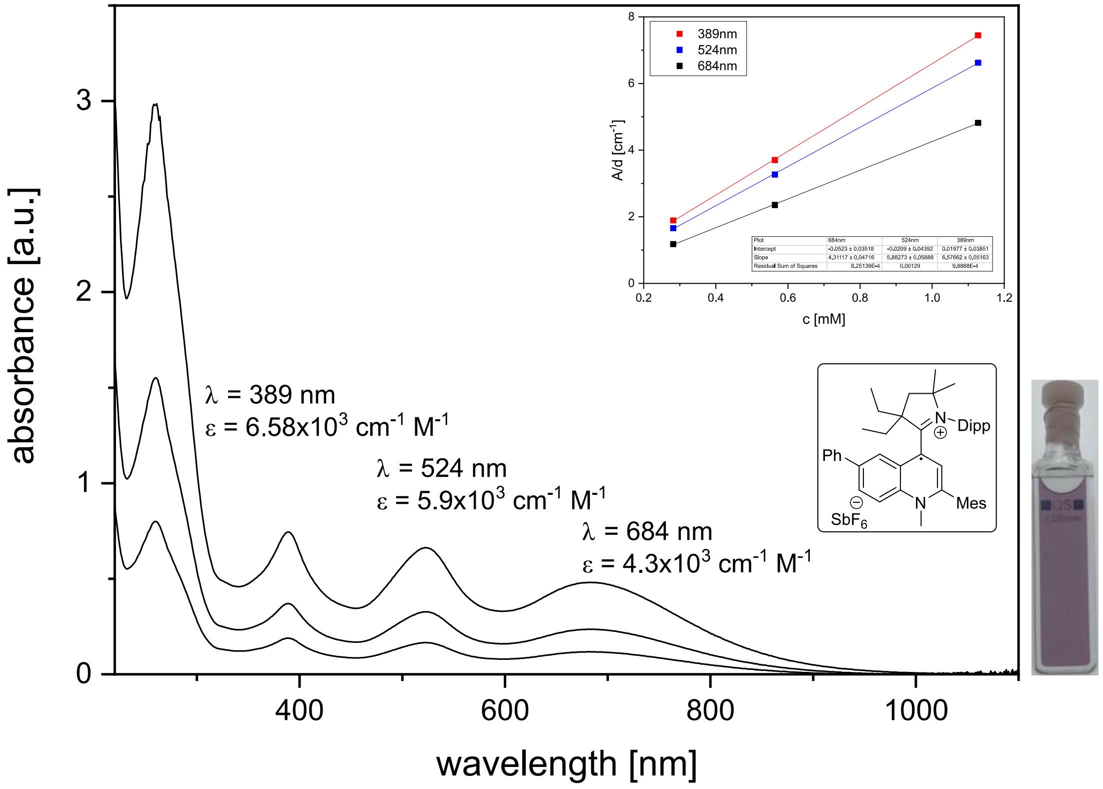

Figure S256: UV-vis spectra of **3h** in thf, measured 0.1 cm quartz cuvettes.

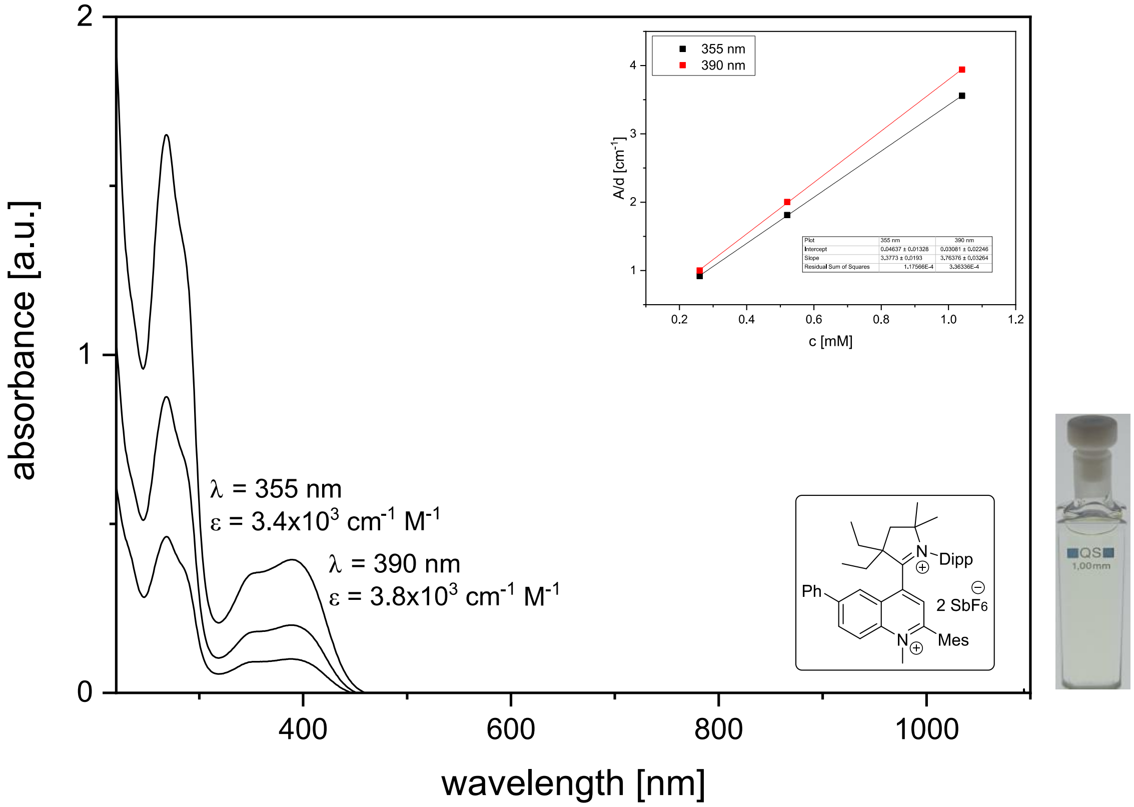

Figure S257: UV-vis spectra of **4h** in CH_3CN, measured 0.1 cm quartz cuvettes.

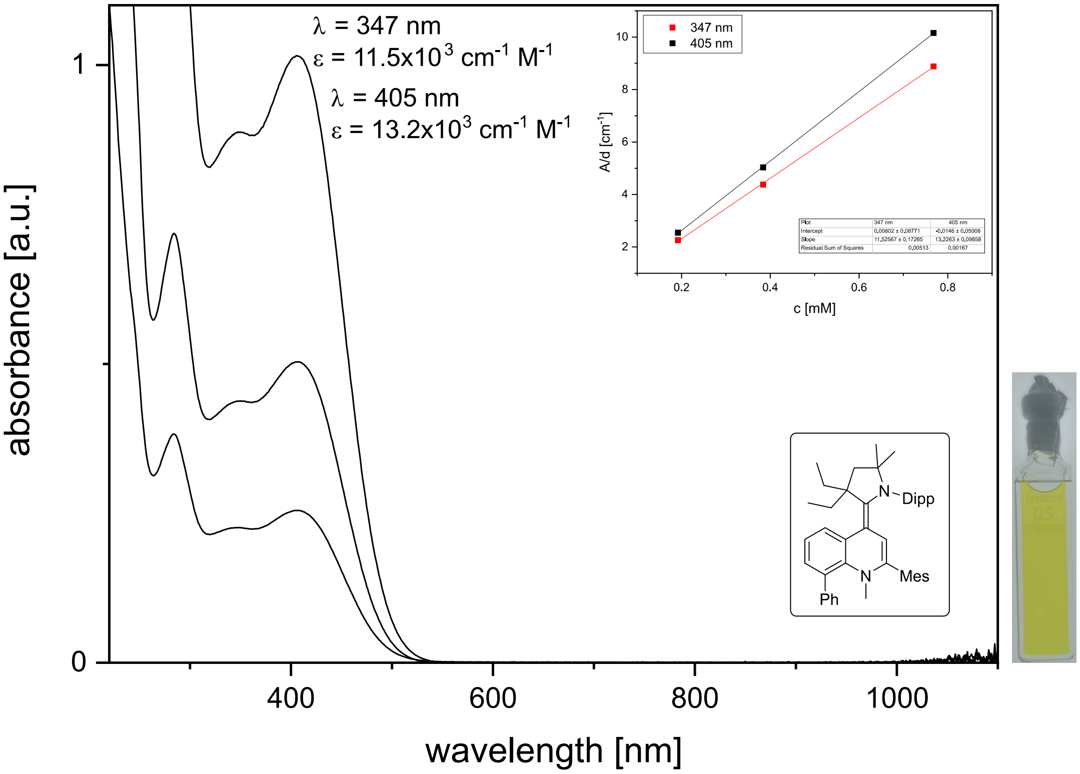

Figure S258: UV-vis spectra of **2i** in thf, measured 0.1 cm quartz cuvettes.

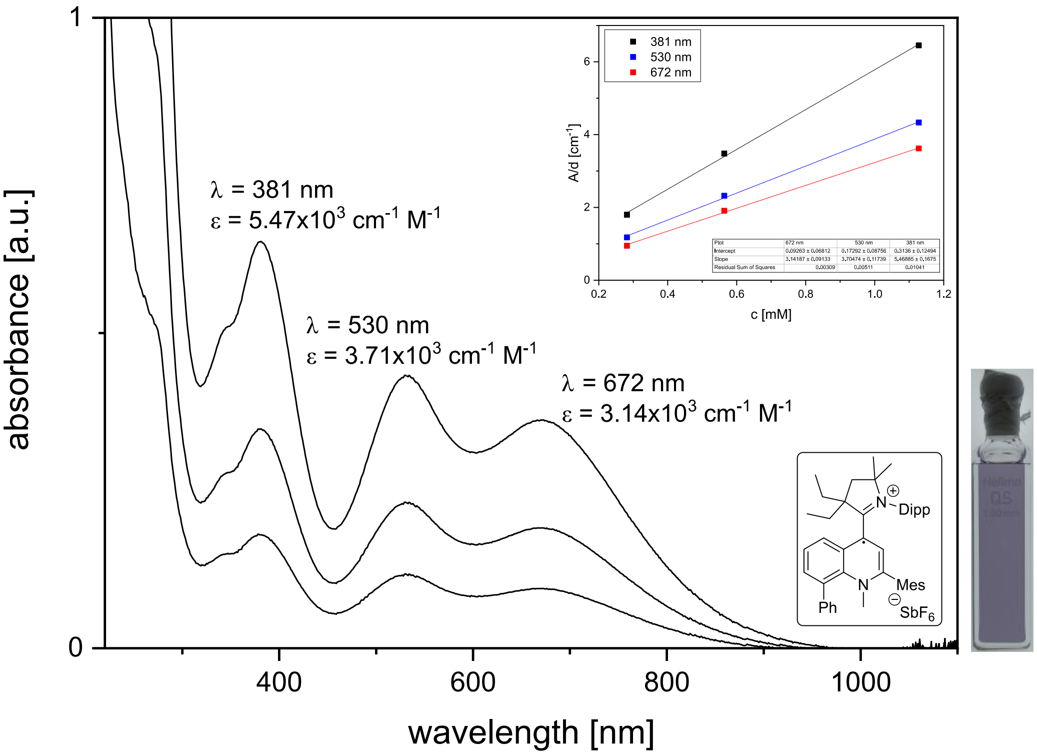

Figure S259: UV-vis spectra of **3i** in thf, measured 0.1 cm quartz cuvettes.

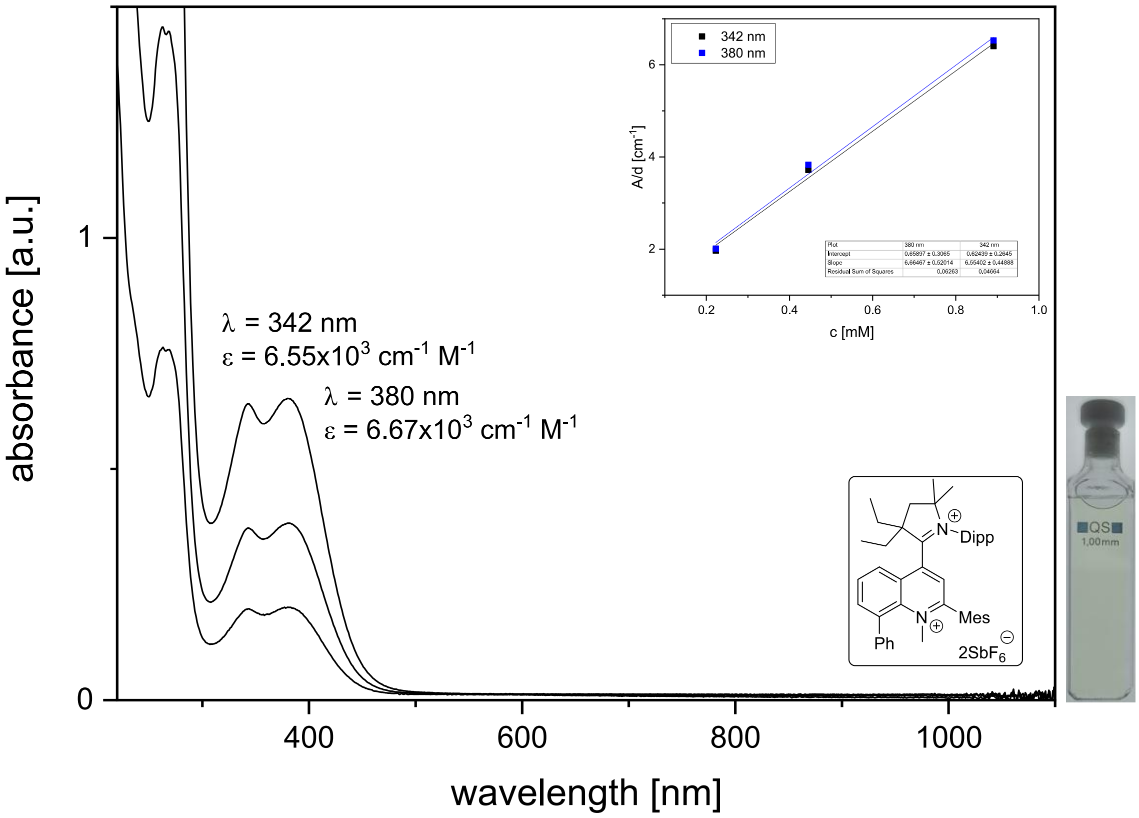

Figure S260: UV-vis spectra of **4i** in thf, measured 0.1 cm quartz cuvettes.

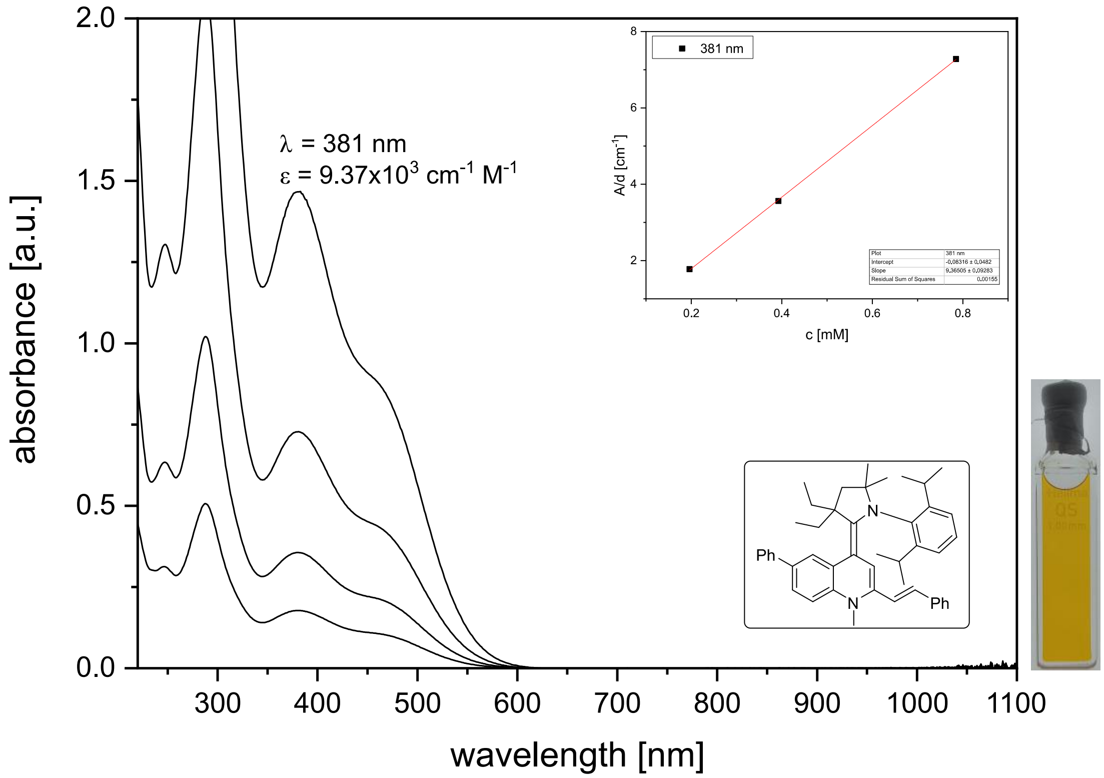

Figure S261: UV-vis spectra of **2j** in thf, measured 0.1 cm quartz cuvettes.

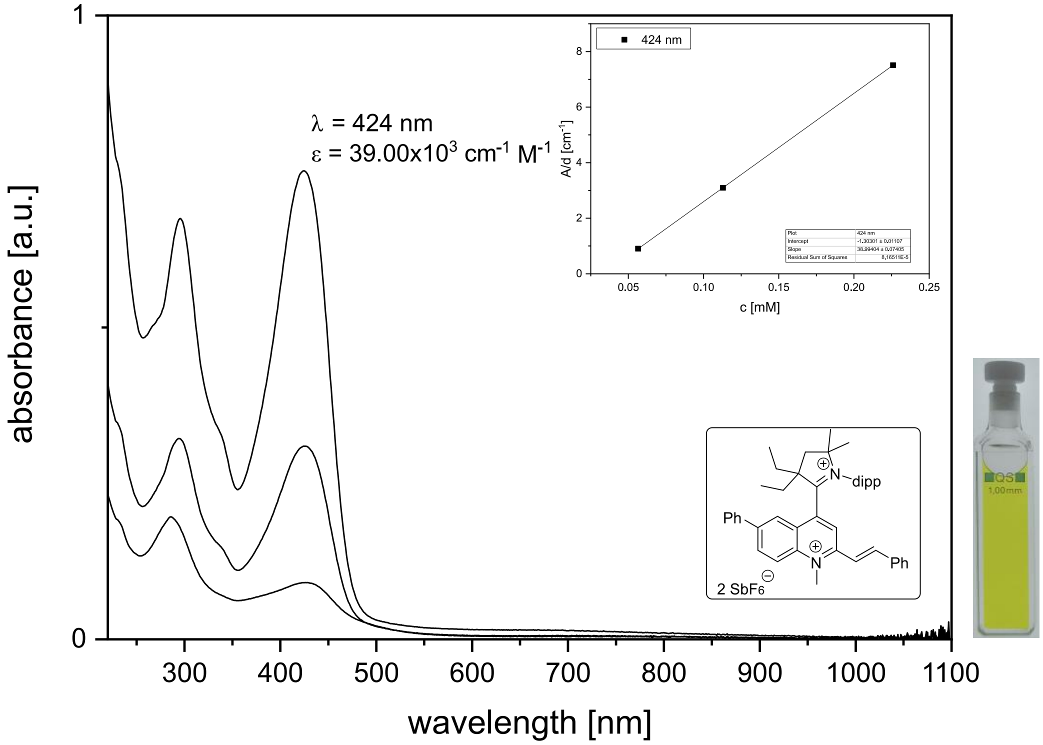

Figure S262: UV-vis spectra of **4j** in CH_3CN, measured 0.1 cm quartz cuvettes.

UV-vis Spectroelectrochemistry

Electrochemical measurements were recorded with a Gamry Instruments Reference 600+. The samples were measured starting from the neutral compounds, containing a three-electrode setup (platinum wire, platinum net and Ag/$AgNO_3$ reference electrode) in a UV-vis cell (1 mm diameter from ALS Co., Ltd; SEC-C) under nitrogen. In order to guarantee a clean oxygen free setup, the measurements were performed in a nitrogen filled glove box. The reference electrode was freshly prepared by using a fritted sample holder, which was activated as described for the sample holder preparation for cyclic voltammetry. To the fritted sample holder was added a freshly prepared 0.01 M $AgNO_3$/ 0.1 M *n*-Bu_4NPF_6 solution in CH_3CN and a silver wire. The light source was a deuterium/tungsten light source and light detected with a BWTEK ExemplarLS. For sample **2f**, **2i** and **2j** Avalight-Hal-S-Mini2/Avalight-DH-S lamps and AvaSpec-NIR256-2.5-HSC-EVO/ AvaSpec-ULS2048CL-RS-EVO-UA detectors were used. A blank spectrum and a reference spectrum with just solvent was taken in advance and was subtracted from the measured data.

Procedure A: A UV-vis spectrum was measured every 10 seconds, while the potential was scanned from negative potentials to positive potentials by a scan rate of 2.0 mV/s.

Procedure B: A UV-VIS spectrum was taken every 10 seconds, while the potential was modified using multiple step controlled potential coulometry for the given potentials and times: (The respective redox potentials were determined beforehand by cyclic voltammetry).

For the visualization of the data not every spectrum but selected are shown in order to reduce the data amount.

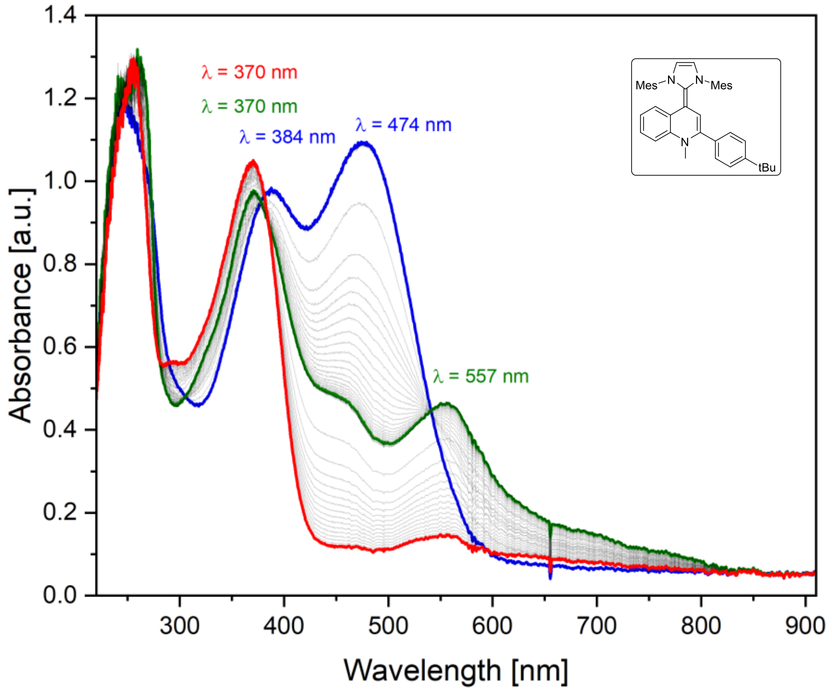

Figure S263: UV-vis-SEC of **2a** (Procedure B) in thf. Color coding: **2a** (blue), **3a** (green), **4a** (red).

Starting potential (vs. Ag^0/Ag^+): -1.7 V; End potential (vs. Ag^0/Ag^+): 0.2 V.

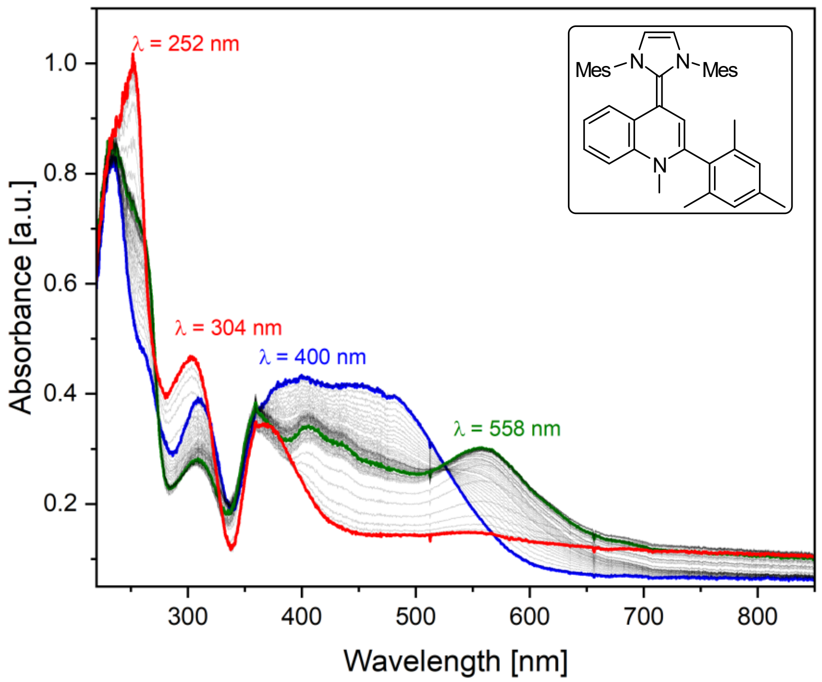

Figure S264: UV-vis-SEC of **2d** (Procedure A) in thf. Color coding: **2d** (blue), **3d** (green), **4d** (red).

Starting potential (vs. Ag^0/Ag^+): -1.4 V (20 s), first step: -0.65 V (900 s), second step 0.4 V (300 s).

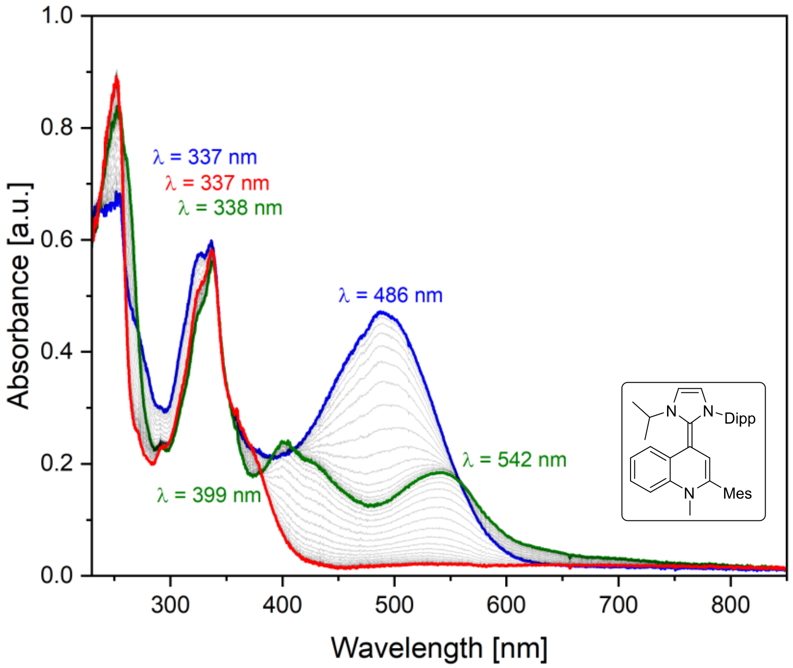

Figure S265: UV-vis-SEC of **XX** (Procedure B) in thf. Color coding: **2e** (blue), **3e** (green), **4e** (red).

Starting potential (vs. Ag^0/Ag^+): -1.7 V; End potential (vs. Ag^0/Ag^+): 0.1 V.

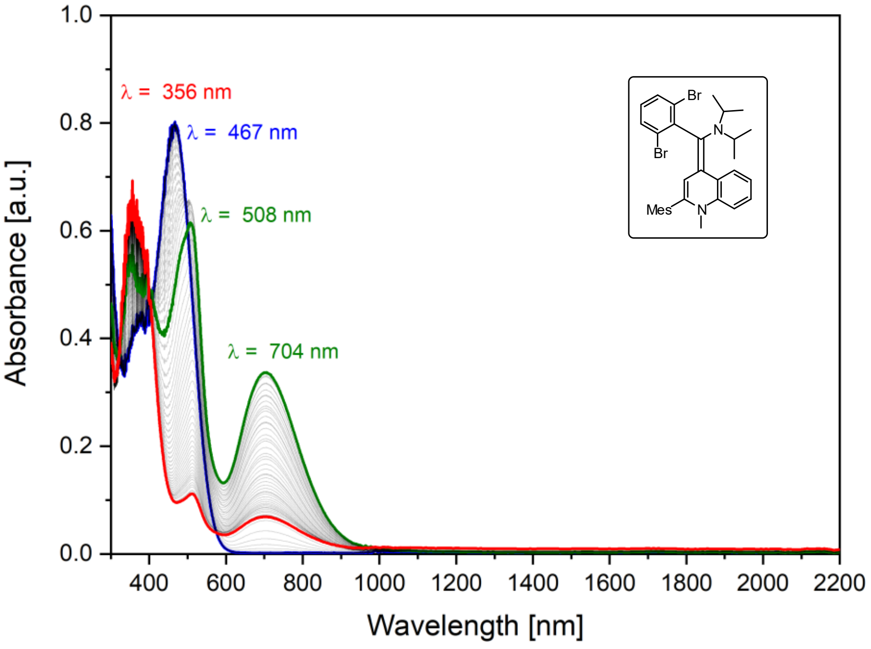

Figure S266: UV-vis-SEC of **2f** (Procedure A) in thf. Color coding: **2f** (blue), **3f** (green), **4f** (red).

Starting potential (vs. Ag0/Ag+): –0.7 V; End potential (vs. Ag0/Ag+): 1.0 V.

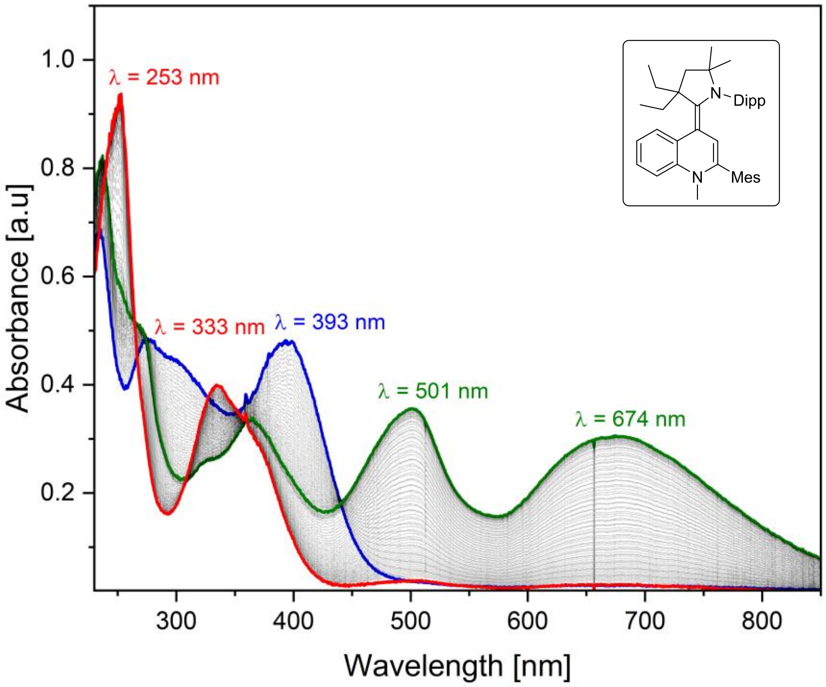

Figure S267: UV-vis-SEC of **2g** in thf (Procedure B). Color coding: **2g** (blue), **3g** (green), **4g** (red).

Starting potential (vs. Ag^0/Ag^+): -0.9 V (20 s), first step: -0.4 V (900 s), second step 1.0 V (900 s).

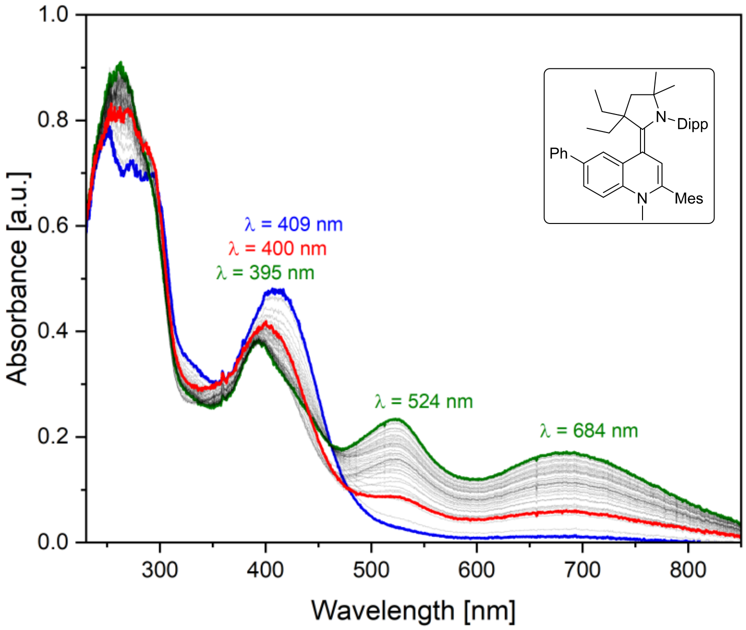

Figure S268: UV-vis-SEC of **2h** in thf (Procedure B). Color coding: **2h** (blue), **3h** (green), **4h** (red).

Starting potential (vs. Ag^0/Ag^+): -1.0 V (20 s), first step: -0.4 V (900 s), second step 1.0 V (900 s).

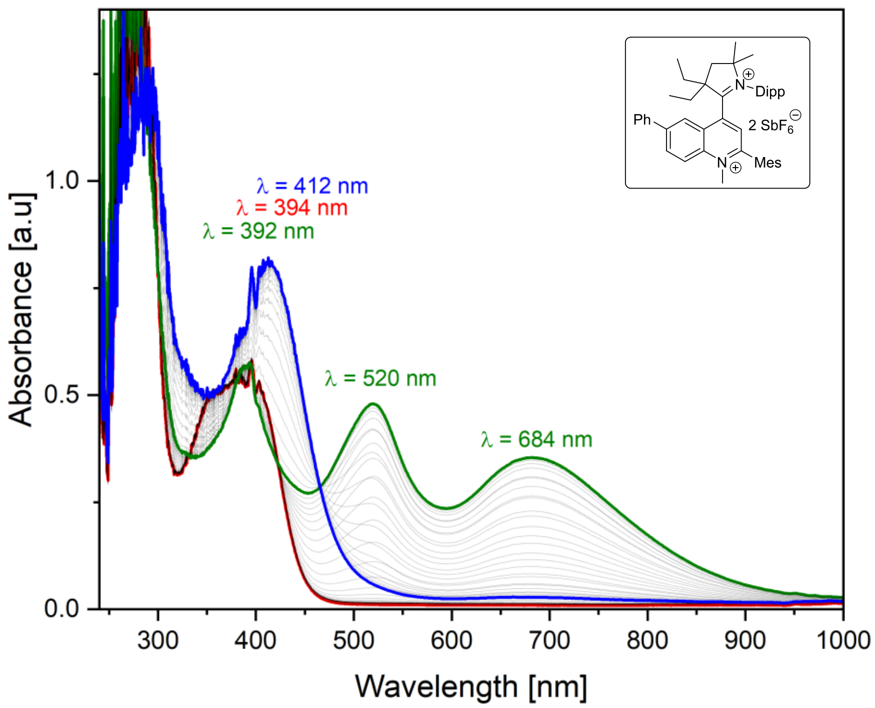

Figure S269: UV-vis-SEC of **4h** in CH_3CN (Procedure A). Color coding: **2h** (blue), **3h** (green), **4h** (red). Note: The measurement was started with **4h** followed by a reductive scan.

Starting potential (vs. Ag^0/Ag^+): 0.1 V; End potential (vs. Ag^0/Ag^+): -1.1 V.

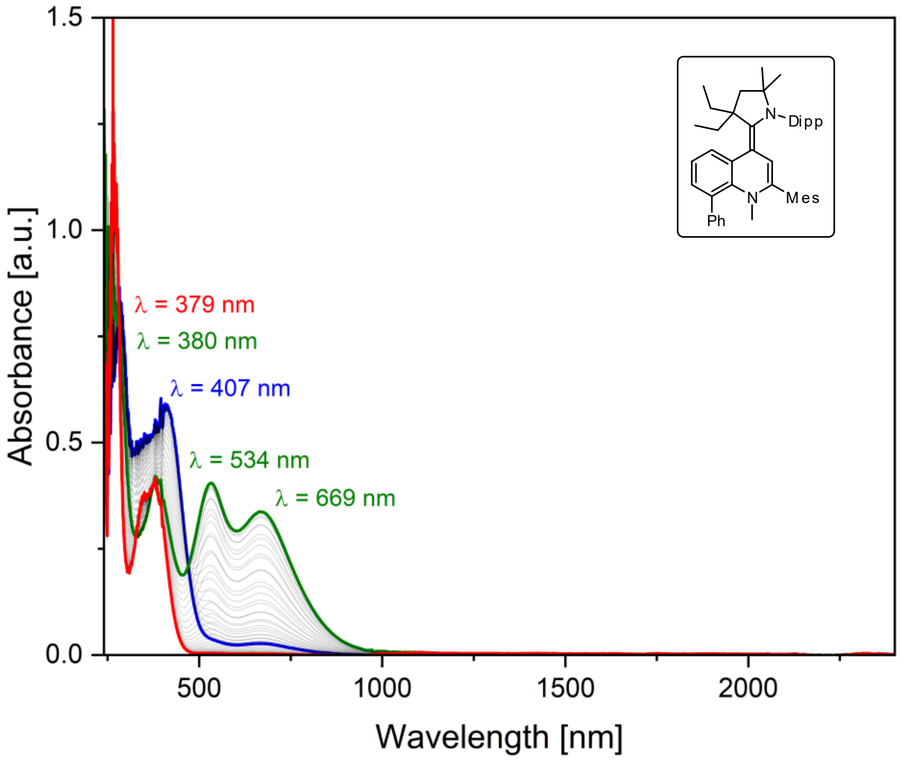

Figure S270: UV-vis-SEC of **2i** in thf (Procedure A). Colour coding: **2i** (blue), **3i** (green), **4i** (red).

Starting potential (vs. Ag^0/Ag^+): -0.7 V; End potential (vs. Ag^0/Ag^+): 0.4 V.

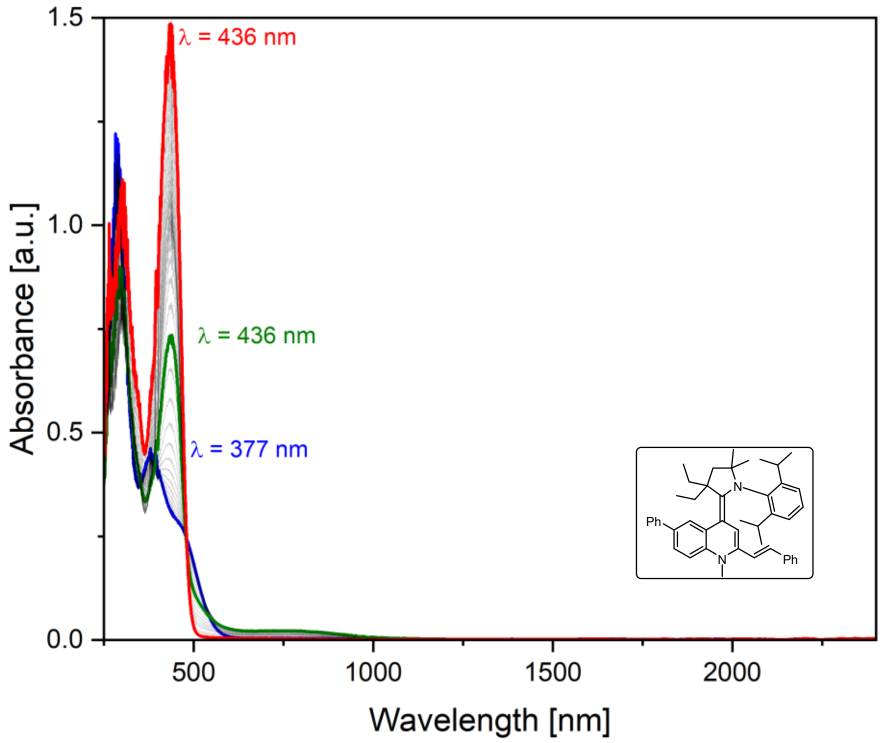

Figure S271: UV-vis-SEC of **2j** (Procedure A) in thf. Colour coding: **2j** (blue), **3j** (green), **4j** (red).

Starting potential (vs. Ag^0/Ag^+): -0.6 V; End potential(vs. Ag^0/Ag^+): 0.45 V.

Fluorescence spectroscopy

Fluorescence spectra were measured on a Carry eclipse fluorescence Spectrometer. A quartz cuvette with 1 cm path length and a J. Young adapter was used. All solvents were degassed prior to use.

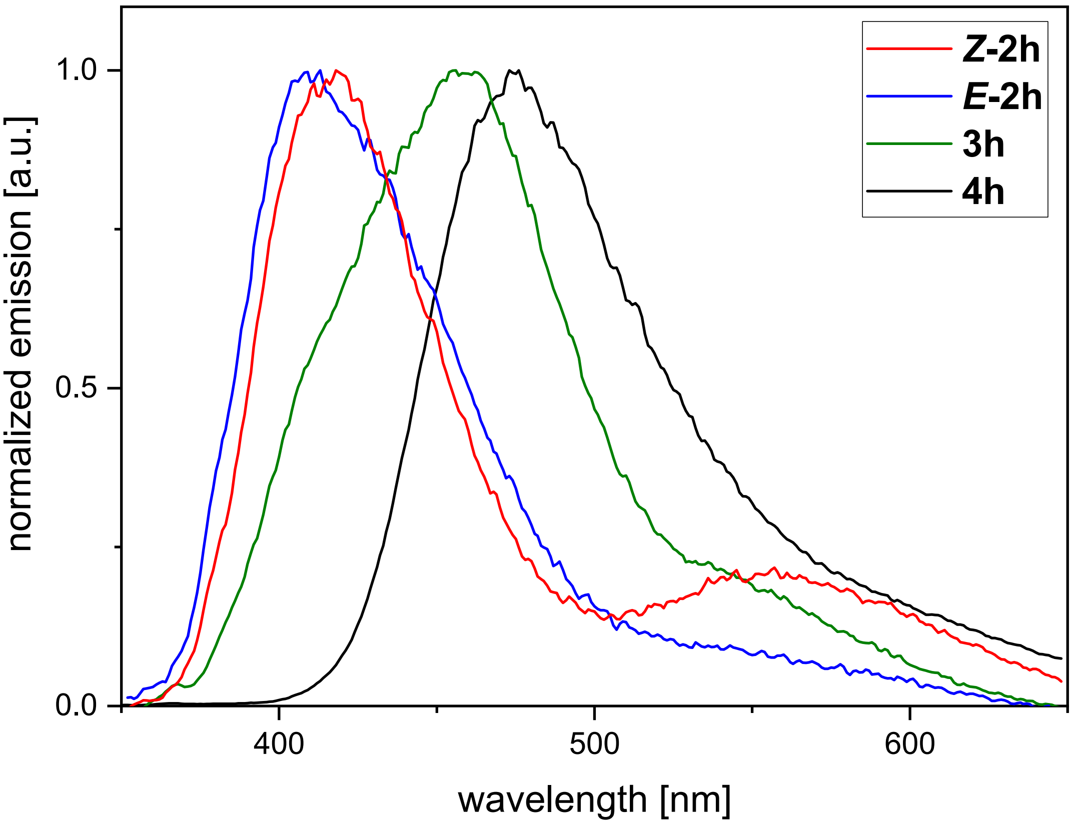

Figure S272: Normalized fluorescence spectra of *E*-**2h, *Z*-2h, 3h and 4h** in thf.

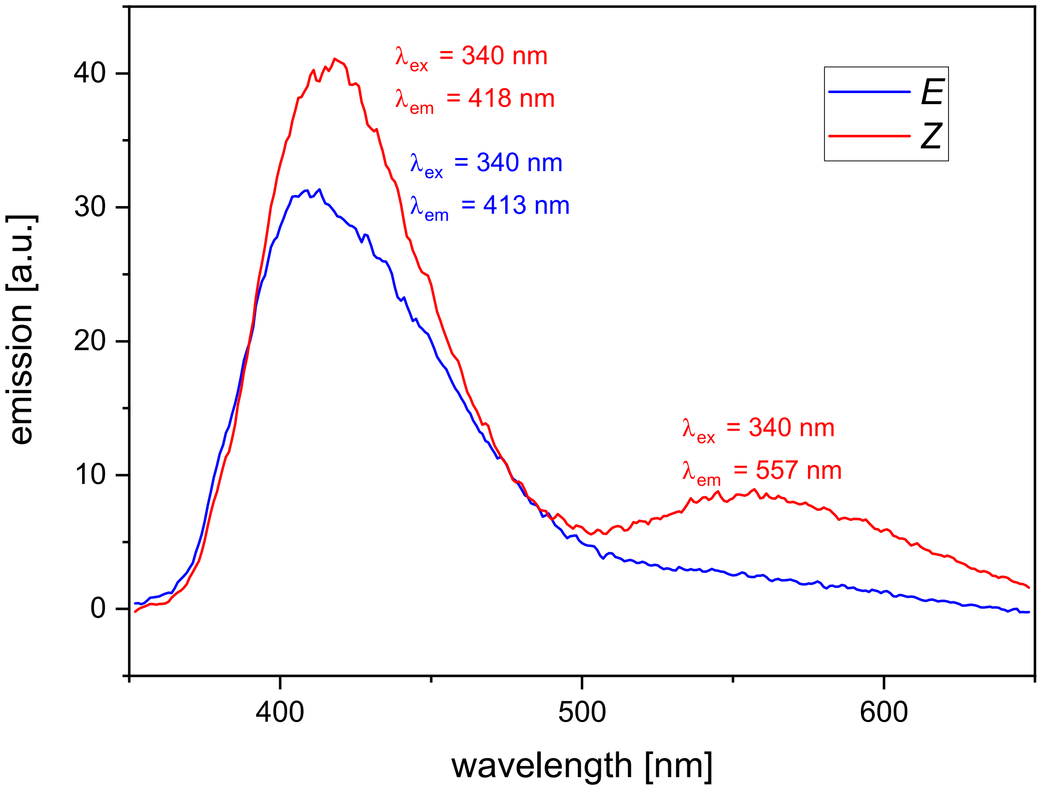

Figure S273: Fluorescence spectra of ***E*-2h** and ***Z*-2h** in thf. λ_{Ex} = 340 nm. 0.025 mg/mL.

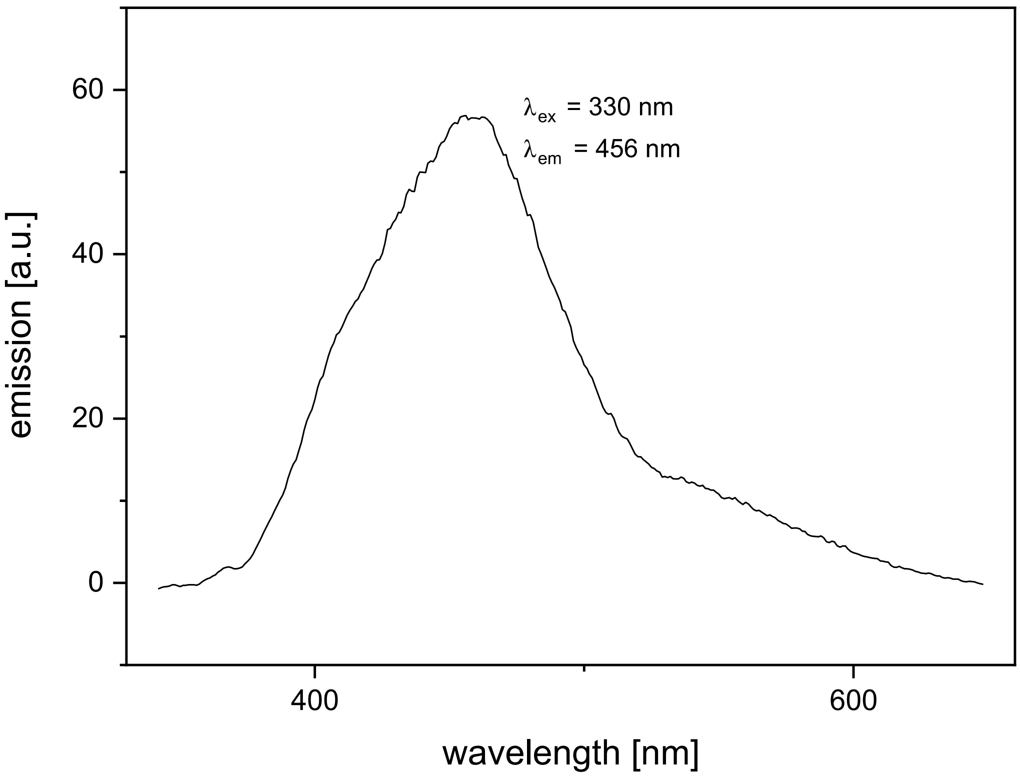

Figure S274: Fluorescence spectra of **3h** in thf. λ_{Ex} = 330 nm. 0.15 mg/mL.

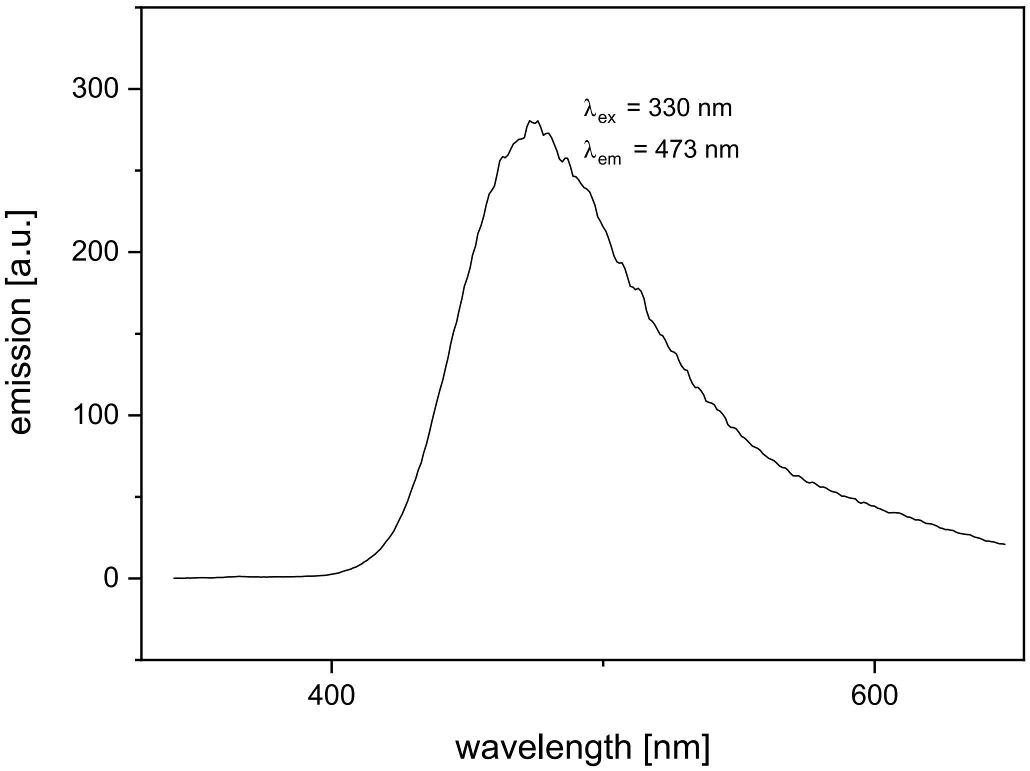

Figure S275: Fluorescence spectra of **4h** in thf. λ_{Ex} = 330 nm. 0.25 mg/mL.

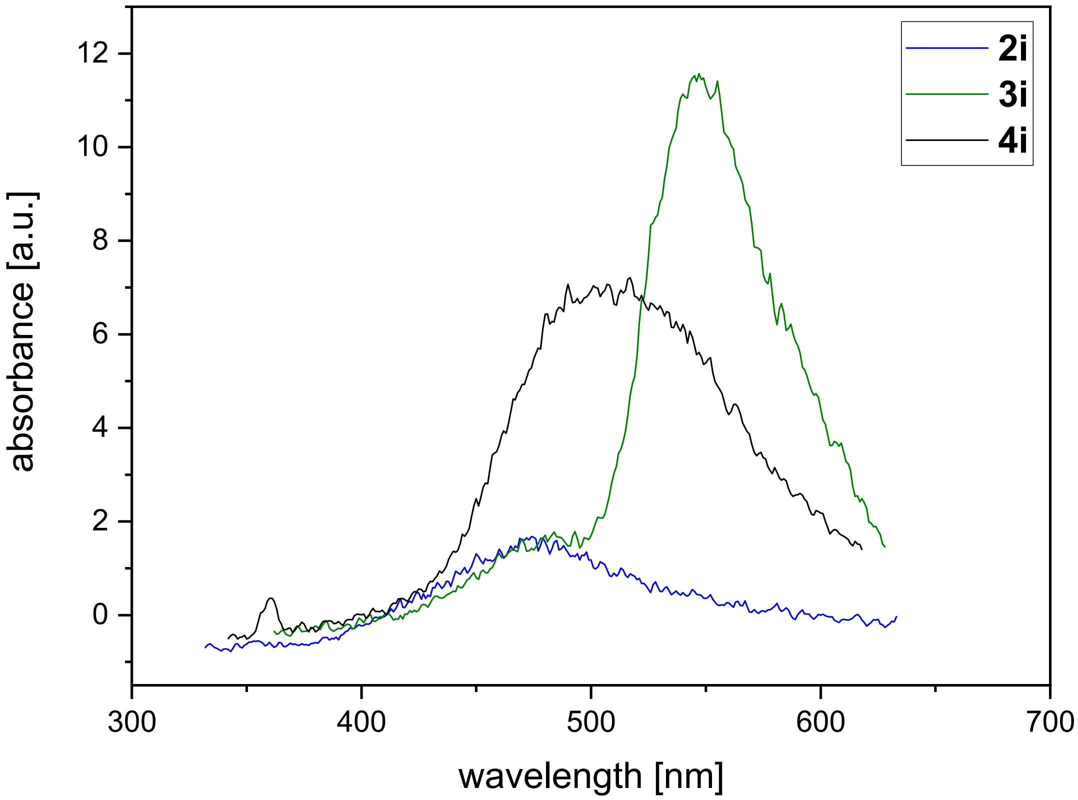

Figure S276: Comparison of fluorescence spectra of **2i, 3i** and **4i** in thf.

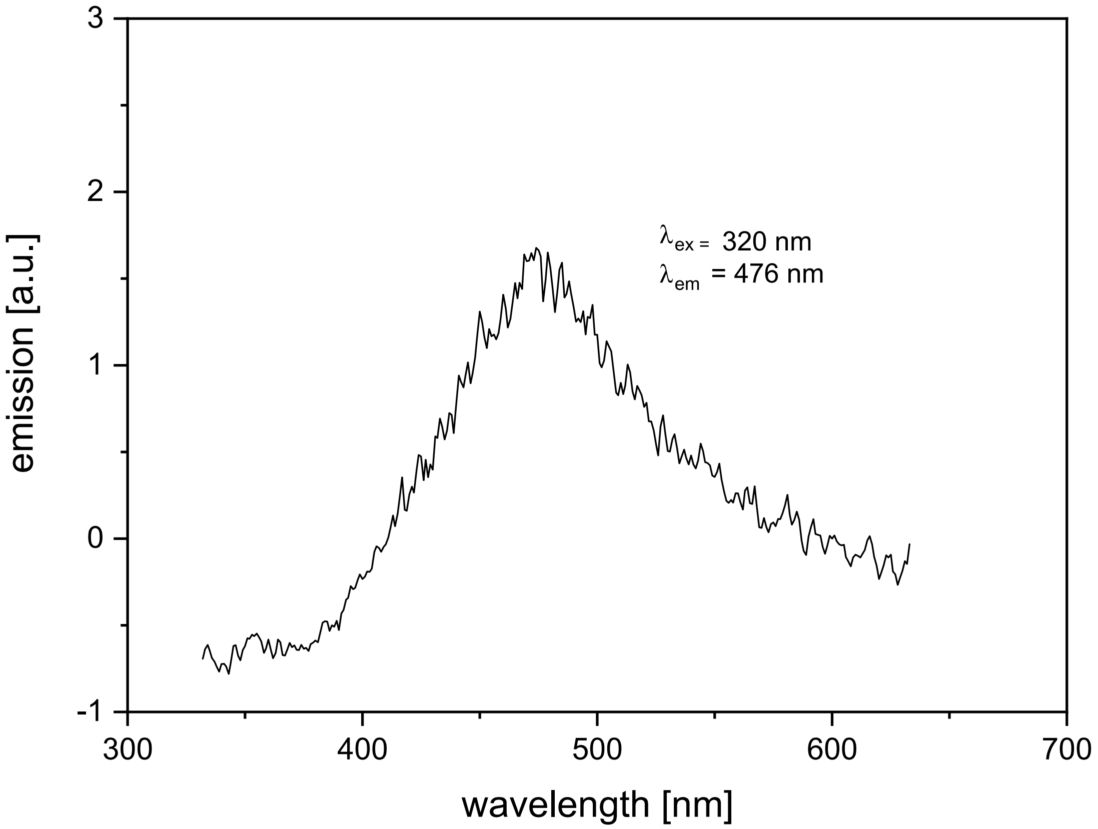

Figure S277: Fluorescence spectra of **2i** in thf. λ_{Ex} = 260 nm. 0.1 mg/mL.

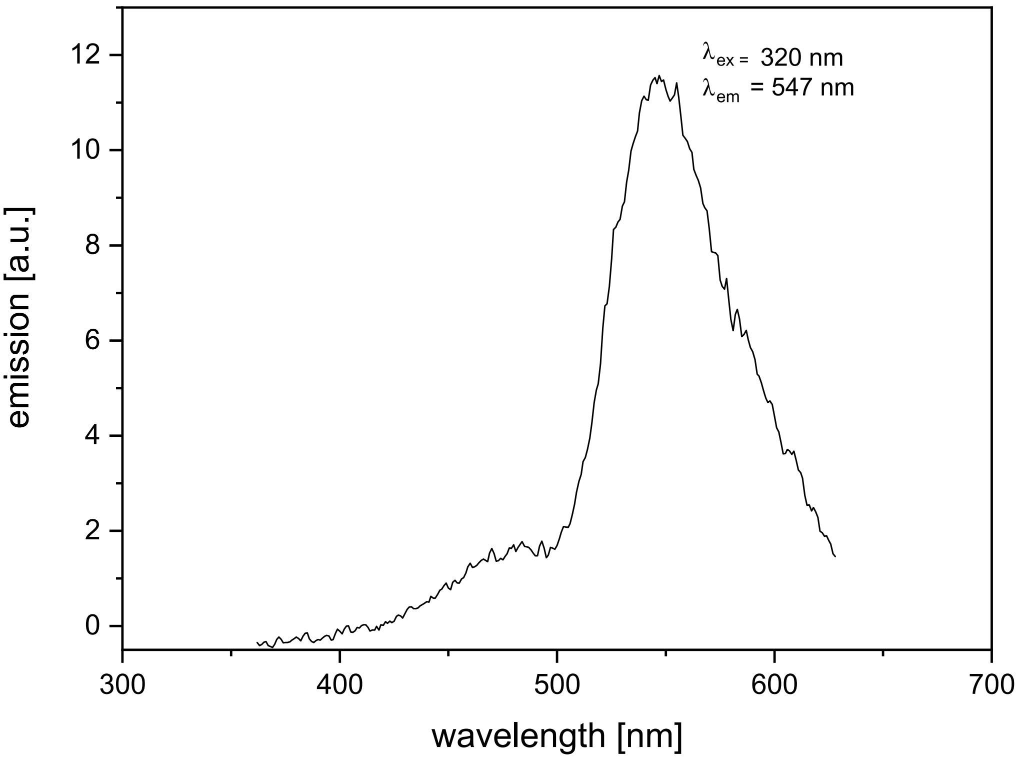

Figure S278: Fluorescence spectra of **3i** in thf. λ_{Ex} = 320 nm. 0.1 mg/mL.

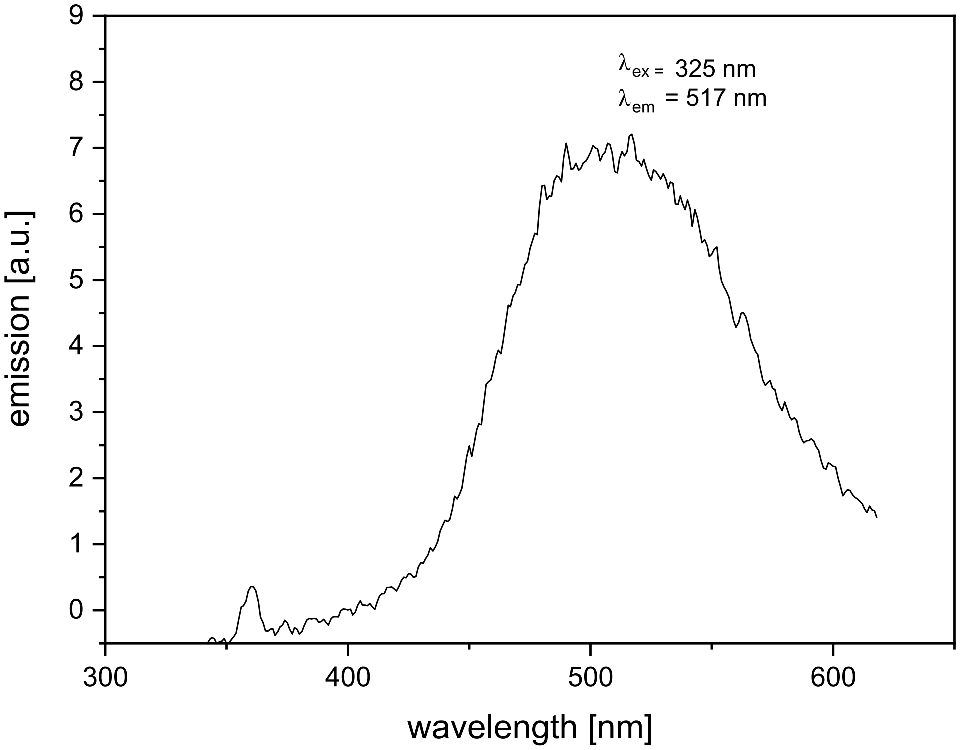

Figure S279: Fluorescence spectra of **4i** n thf. λ_{Ex} = 320 nm. 0.1 mg/mL.

Computational Data

Geometry optimization

For EPR calculation of Radical Cations

All DFT[14,15] calculations for the prediction of EPR parameters were performed with Gaussian16 program package[16] (version g16, rev.C01). All calculations were performed with the B3LYP functionals employing Ahlrich's def2-SVP basis sets.[17,18] Ground states were fully optimized without constraints at the corresponding level of theory and their identity as a minimum were confirmed by a frequency calculation (no imaginary mode). Grimme's D3 dispersion correction with Becke-Johnson damping was used in order to take dispersion effects into account.[19,20] Isotopic hyperfine constants and Mulliken spin densities were calculated at the M06-2X/cc-pVDZ level of theory. For the visualization of frontier molecular orbitals IboView[21], GaussView 6.1[22] and Chemcraft[23] were used.

3a

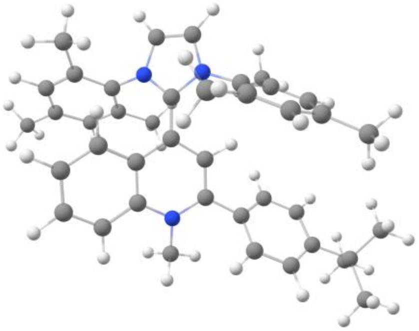

```
%nprocshared=20

%mem=50000MB

#p opt freq b3lyp def2svp
empiricaldispersion=gd3bj gfinput iop(6/7=3)
```

Charge: 1 Multiplicity: 2

Atoms	Cartesian Coordinates X	Y	Z	Atoms	Cartesian Coordinates X	Y	Z
C	3.607463	-1.786707	2.427622	H	3.511536	-3.880414	2.97677
C	3.023	-3.057847	2.450883	H	1.331253	-4.256479	1.915254
C	1.791929	-3.274027	1.837808	H	3.409547	0.240248	1.76516
C	1.134442	-2.231832	1.157671	H	-0.863726	0.656248	-0.458825
C	1.764809	-0.953699	1.053396	H	-1.515032	-3.802249	-0.180102
C	2.974865	-0.755403	1.747496	H	0.137023	-4.436434	0.040554
N	-0.139307	-2.433394	0.607614	H	-0.936574	-4.221263	1.453267
C	-0.871645	-1.355403	0.136958	H	1.73625	4.529557	-0.779597
C	-0.252528	-0.13691	-0.044521	H	4.134162	3.202561	-1.293747
C	1.099815	0.092779	0.299041	H	-2.776184	-2.465189	1.761792
C	-0.643079	-3.798085	0.479815	H	-5.197932	-2.420999	1.456194
C	-2.32471	-1.464957	-0.106561	H	-4.690765	-0.385072	-2.311639
C	1.731746	1.322012	-0.094703	H	-2.258036	-0.451748	-2.019121
N	1.121928	2.549405	-0.170192	H	4.407675	-2.287542	-2.738808
C	2.018423	3.48846	-0.659199	H	7.080176	-0.509322	0.120314
C	3.191158	2.845106	-0.894276	H	6.313308	1.247681	1.550345
N	3.01239	1.508929	-0.552123	H	5.977903	2.484272	0.329757
C	-3.186461	-2.026287	0.849937	H	4.682305	1.947012	1.40565
C	-4.570432	-1.987641	0.678499	H	2.003349	0.50371	-2.742197
C	-5.155175	-1.390855	-0.449182	H	2.551826	-0.978482	-3.55764
C	-4.283174	-0.84621	-1.410265	H	1.604564	-1.081564	-2.059379
C	-2.902465	-0.879097	-1.247868	H	6.807241	-3.372812	-0.704609
C	3.979926	0.482832	-0.834427	H	6.622975	-3.210599	-2.464834
C	3.676147	-0.505889	-1.786384	H	7.867012	-2.269769	-1.600458
C	4.62695	-1.511025	-2.001573	H	-2.181663	2.928249	2.996443
C	5.847375	-1.542176	-1.319051	H	-3.283487	3.370419	-1.138976
C	6.12437	-0.511878	-0.410125	H	-1.705479	3.065187	-2.867986
C	5.211201	0.512235	-0.150719	H	-0.199731	3.929417	-2.502911
C	5.556632	1.599373	0.835697	H	-0.217609	2.161037	-2.492982
C	2.392762	-0.511204	-2.575759	H	1.432691	3.056061	2.426689
C	6.837547	-2.654409	-1.541405	H	0.138424	2.868822	3.635433
C	-0.228243	2.837323	0.231897	H	0.716845	1.460253	2.720489
C	-0.555379	2.79689	1.594765	H	-4.923124	2.421213	0.970342
C	-1.903272	2.962638	1.940304	H	-4.481664	3.309912	2.449128
C	-2.898074	3.157545	0.975902	H	-4.799339	4.189135	0.931801
C	-2.518122	3.216247	-0.374049	H	-7.250586	-1.501023	1.458379
C	-1.190033	3.056339	-0.773421	H	-8.532949	-1.889384	0.295505
C	-0.81045	3.053234	-2.231987	H	-7.214154	-3.04605	0.565419
C	0.488164	2.536752	2.648557	H	-6.564747	-1.516066	-2.846205
C	-4.349134	3.279197	1.358814	H	-8.141711	-1.929496	-2.141187
C	-6.672833	-1.299647	-0.653411	H	-6.76738	-3.051212	-1.969037
C	-7.452579	-1.974365	0.485194	H	-8.164671	0.286148	-0.851916
C	-7.05379	-1.988197	-1.981193	H	-6.812102	0.702114	0.231454
C	-7.077214	0.189905	-0.707231	H	-6.581759	0.719504	-1.534887
H	4.549675	-1.600887	2.946134				

Point Group	C1	ε + U_{corr}	-1751.730867 Eh
Electronic Energy (ε)	-1752.532799 Eh	ε + H_{corr}	-1751.730867 Eh
ε + *ZPE*	-1751.774405 Eh	ε + G_{corr}	-1751.853372 Eh

3b

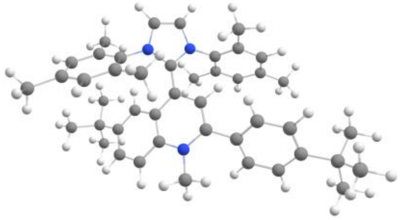

```
%nprocshared=20

%mem=50000MB

#p opt freq b3lyp def2svp
empiricaldispersion=gd3bj gfinput
iop(6/7=3)
```

Charge: 1 Multiplicity: 2

Atoms	X	Y	Z	Atoms	X	Y	Z
	Cartesian Coordinates				Cartesian Coordinates		
C	3.401419	-1.731841	1.546781	H	-1.486969	0.804569	-0.480468
C	2.867929	-3.024593	1.378779	H	-1.847681	-3.672184	-0.938025
C	1.61156	-3.232158	0.830033	H	-0.147393	-4.208289	-0.985453
C	0.831106	-2.14739	0.394002	H	-1.077408	-4.311295	0.537068
C	1.386009	-0.834001	0.445542	H	0.695744	4.823742	-0.711872
C	2.650096	-0.665035	1.053925	H	3.059893	3.789855	-1.753187
N	-0.479174	-2.353226	-0.059421	H	-2.973131	-2.777269	1.326464
C	-1.319955	-1.270864	-0.253158	H	-5.411816	-2.877067	1.25505
C	-0.790373	0.000288	-0.276967	H	-5.426961	-0.210589	-2.134287
C	0.582314	0.26791	-0.055165	H	-2.977082	-0.12988	-2.080095
C	-0.912796	-3.711606	-0.371947	H	3.754002	-1.617665	-3.503281
C	-2.781938	-1.447617	-0.373257	H	6.599054	0.516661	-1.091409
C	1.07459	1.582641	-0.361204	H	5.914879	2.21108	0.395164
N	0.364398	2.748727	-0.206055	H	5.045832	3.34759	-0.639214
C	1.078508	3.808844	-0.744913	H	4.195258	2.556378	0.69643
C	2.239294	3.30654	-1.235634	H	1.006387	0.807423	-2.996409
N	2.240496	1.935557	-0.99721	H	1.665143	-0.476458	-4.035964
C	-3.502826	-2.226942	0.546042	H	0.990808	-0.874784	-2.443835
C	-4.896769	-2.271637	0.510382	H	6.104669	-2.207921	-3.632786
C	-5.631492	-1.545169	-0.439366	H	7.33099	-1.145605	-2.891612
C	-4.899872	-0.780329	-1.36684	H	6.539509	-2.400379	-1.920845
C	-3.510381	-0.728774	-1.338648	H	-2.282545	2.533996	3.540893
C	3.270286	1.053769	-1.474594	H	-4.175672	3.402286	-0.223292
C	2.954496	0.042484	-2.398696	H	-2.954835	3.345652	-2.245266
C	3.984668	-0.822329	-2.789883	H	-1.490874	4.328186	-2.05481
C	5.293496	-0.687627	-2.318244	H	-1.346876	2.579179	-2.270297
C	5.575756	0.374616	-1.447931	H	1.147613	2.930411	2.325591
C	4.586763	1.258537	-1.011646	H	0.11481	2.5397	3.722679
C	4.948002	2.3971	-0.089934	H	0.551731	1.27585	2.552621
C	1.581939	-0.128345	-2.996491	H	-5.376025	2.179656	1.981369
C	6.372283	-1.657555	-2.71977	H	-4.657569	2.81004	3.484549
C	-0.896754	2.883982	0.470423	H	-5.253611	3.929768	2.233614
C	-0.954279	2.660864	1.854043	H	-7.508112	-2.126277	1.605416
C	-2.214729	2.709164	2.464375	H	-8.876135	-2.424683	0.515702
C	-3.382501	2.965094	1.737591	H	-7.465757	-3.500872	0.467691
C	-3.272419	3.202466	0.358887	H	-7.265837	-1.400969	-2.685782
C	-2.042101	3.166135	-0.299729	H	-8.734532	-2.043794	-1.920755
C	-1.954974	3.363912	-1.791539	H	-7.281899	-3.067079	-2.060587
C	0.279665	2.339555	2.655264	H	-8.768582	-0.073669	-0.278243
C	-4.734453	2.97253	2.400588	H	-7.336472	0.275706	0.724361
C	-7.164877	-1.544633	-0.486953	H	-7.299461	0.588198	-1.028669
C	-7.778755	-2.452526	0.589408	H	4.529162	-3.369821	3.521182
C	-7.634448	-2.041459	-1.870755	H	5.654467	-2.156434	4.15829
C	-7.667914	-0.102928	-0.255715	H	3.896636	-1.880316	4.261437
C	4.747623	-1.565862	2.264368	H	5.196761	0.461607	1.581204
C	4.698932	-2.28868	3.627388	H	6.038592	-0.016192	3.064176
C	5.085468	-0.092169	2.519948	H	4.313605	0.40401	3.128802
C	5.857846	-2.184452	1.388474	H	6.836533	-2.089644	1.884997
H	3.430401	-3.894104	1.720798	H	5.6791	-3.254035	1.201321
H	1.22437	-4.247493	0.777358	H	5.911262	-1.673841	0.415952
H	3.023322	0.350188	1.161483				

Point Group	C1	ε + U_{corr}	-1908.782648 Eh
Electronic Energy (ε)	-1909.701255 Eh	ε + H_{corr}	-1908.781704 Eh
ε + *ZPE*	-1908.830571 Eh	ε + G_{corr}	-1908.914519 Eh

3c

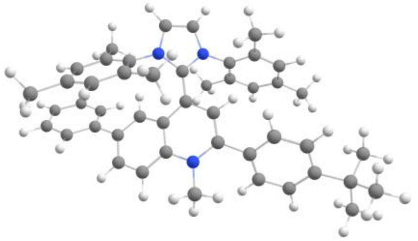

```
%nprocshared=20

%mem=50000MB

#p opt freq b3lyp def2svp
empiricaldispersion=gd3bj gfinput
iop(6/7=3)
```

Charge: 1 Multiplicity: 2

	Cartesian Coordinates				Cartesian Coordinates		
Atoms	*X*	*Y*	*Z*	Atoms	*X*	*Y*	*Z*
C	-3.311874	-1.641196	-1.168986	H	-3.464204	-3.800994	-1.257638
C	-2.825069	-2.952444	-1.008723	H	-1.163033	-4.217329	-0.559265
C	-1.517806	-3.189851	-0.602435	H	-2.792147	0.431503	-1.003574
C	-0.657414	-2.124268	-0.278637	H	1.790308	0.771365	0.457937
C	-1.158848	-0.788481	-0.341827	H	2.067915	-3.711444	0.871186
C	-2.447967	-0.587957	-0.862442	H	0.361716	-4.190768	1.067377
N	0.673217	-2.36033	0.085006	H	1.157595	-4.34363	-0.525715
C	1.55094	-1.297939	0.230964	H	-0.415525	4.860966	0.75961
C	1.06073	-0.010446	0.285549	H	-2.827293	3.840926	1.715184
C	-0.310326	0.297039	0.117128	H	3.068086	-2.858245	-1.432999
C	1.088896	-3.727858	0.38464	H	5.503425	-3.004156	-1.520063
C	3.012933	-1.50999	0.262346	H	5.790222	-0.305919	1.831937
C	-0.787197	1.616264	0.432306	H	3.345249	-0.180224	1.93878
N	-0.06868	2.779819	0.294347	H	-3.558886	-1.521491	3.554003
C	-0.802572	3.847507	0.789792	H	-6.267129	0.387951	0.814135
C	-1.983273	3.352366	1.240741	H	-5.503567	2.018169	-0.697834
N	-1.974173	1.979099	1.025193	H	-4.797945	3.272774	0.340061
C	3.65754	-2.3115	-0.693994	H	-3.781471	2.43536	-0.84016
C	5.049838	-2.382261	-0.749457	H	-0.861378	0.981122	3.164747
C	5.858772	-1.660101	0.141374	H	-1.505249	-0.349241	4.154418
C	5.203751	-0.873469	1.107244	H	-0.736725	-0.69056	2.591372
C	3.816793	-0.796701	1.170558	H	-5.720282	-2.536322	3.12085
C	-3.019895	1.104192	1.483211	H	-6.853517	-1.175587	3.277226
C	-2.736242	0.134413	2.458034	H	-6.675401	-1.990542	1.713937
C	-3.763348	-0.753847	2.803177	H	2.76714	2.538737	-3.308599
C	-5.033899	-0.685946	2.223545	H	4.480636	3.282128	0.567194
C	-5.28377	0.325298	1.285576	H	3.14744	3.285493	2.516357
C	-4.298867	1.235828	0.903005	H	1.681603	4.252858	2.26557
C	-4.607776	2.292243	-0.126924	H	1.551103	2.498176	2.442849
C	-1.390108	0.018063	3.12483	H	-0.705262	3.058627	-2.279731
C	-6.122308	-1.653522	2.603725	H	0.3831	2.620571	-3.620011
C	1.228345	2.899739	-0.31337	H	-0.171386	1.378647	-2.476555
C	1.353197	2.69628	-1.695186	H	5.721767	1.981293	-1.626461
C	2.646299	2.702602	-2.234975	H	5.1446	2.759915	-3.120449
C	3.781123	2.899073	-1.440312	H	5.744488	3.748414	-1.764271
C	3.603951	3.125048	-0.0664	H	7.584801	-2.31662	-2.011815
C	2.338386	3.127782	0.523016	H	9.017276	-2.616064	-1.008987
C	2.172629	3.299872	2.011028	H	7.595423	-3.665291	-0.842726
C	0.152086	2.42956	-2.563292	H	7.649129	-1.490965	2.267754
C	5.167479	2.847355	-2.025387	H	9.048906	-2.178558	1.417919
C	7.391632	-1.684061	0.085145	H	7.592662	-3.171124	1.68381
C	7.917123	-2.625708	-1.008862	H	8.998238	-0.241697	-0.260558
C	7.948262	-2.15693	1.444773	H	7.511702	0.101985	-1.181581
C	7.898041	-0.255951	-0.213978	H	7.586948	0.459725	0.561978
C	-4.70095	-1.377708	-1.609452	H	-4.198959	0.339598	-2.826287
C	-5.00595	-0.288201	-2.442696	H	-6.541103	0.834163	-3.461913
C	-6.325136	-0.011873	-2.805206	H	-8.398957	-0.605402	-2.625203
C	-7.366581	-0.821363	-2.341804	H	-7.882095	-2.557325	-1.15865
C	-7.07547	-1.917077	-1.523323	H	-5.54291	-3.033966	-0.500146
C	-5.756038	-2.193394	-1.16351				

Point Group	C1	ε + U_{corr}	-1982.572915 Eh
Electronic Energy (ε)	-1983.459910 Eh	ε + H_{corr}	-1982.571971 Eh
ε + *ZPE*	-1982.619906 Eh	ε + G_{corr}	-1982.702460 Eh

3d

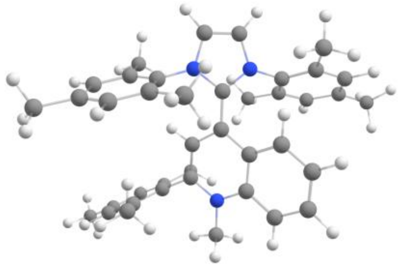

```
%nprocshared=20

%mem=50000MB

#p opt freq b3lyp def2svp
empiricaldispersion=gd3bj gfinput
iop(6/7=3)
```

Charge: 1 Multiplicity: 2

	Cartesian Coordinates				Cartesian Coordinates		
Atoms	X	Y	Z	Atoms	X	Y	Z
C	2.94736	-0.93972	3.07884	H	3.84827	-0.57239	3.57459
C	2.36884	-2.15198	3.46767	H	2.82052	-2.75263	4.26
C	1.19219	-2.58877	2.86398	H	0.74612	-3.51922	3.20765
C	0.57702	-1.8368	1.84317	H	2.80365	0.75716	1.78308
C	1.19818	-0.62826	1.38839	H	-1.28601	0.26376	-0.82931
C	2.36317	-0.19779	2.05977	H	-5.97279	-1.45539	0.67219
N	-0.64752	-2.25232	1.29645	H	-4.20385	-3.55424	-2.63782
C	-1.31814	-1.45394	0.38019	H	-2.15277	-3.70713	1.20839
C	-0.72892	-0.29759	-0.08296	H	-0.52717	-4.34415	1.57788
C	0.56356	0.13081	0.32443	H	-1.45538	-3.4726	2.8325
C	-2.6763	-1.85528	-0.1047	H	1.02729	4.27557	-1.65349
C	-3.82112	-1.42808	0.6054	H	3.47512	2.98071	-1.89763
C	-5.08725	-1.78528	0.12157	H	-7.41683	-2.4067	-0.99892
C	-5.25113	-2.54955	-1.03991	H	-6.73281	-2.73757	-2.61095
C	-4.09876	-2.95671	-1.7274	H	-6.79443	-4.0239	-1.3952
C	-2.81347	-2.62446	-1.28439	H	-1.89075	-3.78676	-2.86103
C	-1.22636	-3.51287	1.75566	H	-1.08665	-2.23535	-2.54334
C	1.15626	1.27997	-0.32371	H	-0.86233	-3.58644	-1.4249
N	0.49191	2.43972	-0.64327	H	-3.07914	-1.0924	2.62574
C	1.34922	3.29266	-1.32296	H	-3.22584	0.37794	1.65487
C	2.54853	2.6677	-1.42677	H	-4.68532	-0.40539	2.30599
N	2.43225	1.42546	-0.81589	H	4.21695	-2.48784	-2.4698
C	-6.62058	-2.94334	-1.53452	H	6.6437	-0.13835	0.18891
C	-1.60435	-3.08386	-2.06607	H	5.64525	1.69122	1.42154
C	-3.69864	-0.59619	1.86088	H	5.42673	2.69018	-0.02069
C	3.29709	-0.69038	-1.7391	H	4.04014	2.34703	1.02178
C	3.47911	0.43109	-0.90187	H	1.22929	-1.3225	-1.95875
C	4.3427	-1.61607	-1.82157	H	1.67822	0.0485	-2.99033
C	5.5478	-1.45096	-1.12407	H	2.24478	-1.58678	-3.39081
C	5.70194	-0.30056	-0.34258	H	6.74581	-2.86888	-2.24288
C	4.69021	0.66109	-0.21914	H	7.61098	-2.08393	-0.89603
C	4.95253	1.90668	0.59536	H	6.41054	-3.35253	-0.57101
C	2.04763	-0.89539	-2.55972	H	-2.55588	3.89035	2.48969
C	6.63926	-2.48669	-1.21603	H	-3.91571	3.39018	-1.56134
C	-1.06824	3.19338	1.10348	H	-2.53227	2.40799	-3.20541
C	-0.83868	2.83635	-0.23928	H	-1.04463	3.36958	-3.18827
C	-2.36038	3.60829	1.45154	H	-0.98013	1.651	-2.77611
C	-3.39867	3.68771	0.51344	H	0.25341	2.10844	2.44252
C	-3.11908	3.33513	-0.81434	H	0.95974	3.59365	1.7762
C	-1.84796	2.91171	-1.22023	H	-0.27753	3.69022	3.04809
C	-1.58748	2.56314	-2.66656	H	-4.96315	4.01624	1.98117
C	0.02337	3.14427	2.14346	H	-4.8687	5.25699	0.71592
C	-4.76928	4.17503	0.91033	H	-5.56006	3.66927	0.33615

Point Group	C1	ε + U_{corr}	-1712.258751 Eh
Electronic Energy (ε)	-1713.027861 Eh	ε + H_{corr}	-1712.257807 Eh
ε + *ZPE*	-1712.301741 Eh	ε + G_{corr}	-1712.383535 Eh

3e

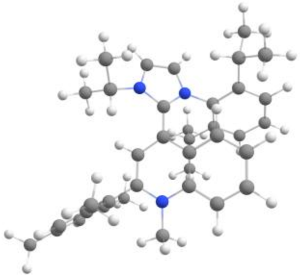

```
%nprocshared=20

%mem=50000MB

#p opt freq b3lyp def2svp
empiricaldispersion=gd3bj gfinput
iop(6/7=3)
```

Charge: 1 Multiplicity: 2

Atoms	Cartesian Coordinates X	Y	Z	Atoms	Cartesian Coordinates X	Y	Z
C	-2.07388	-2.16197	-2.48929	H	-2.40745	-0.15673	-1.80804
C	-1.16214	-3.22053	-2.55934	H	1.63655	1.34215	0.43026
C	0.11219	-3.08515	-2.01348	H	2.32929	-3.74038	-0.48038
C	0.48943	-1.90183	-1.35277	H	2.92206	-3.10016	-2.04135
C	-0.4651	-0.84831	-1.20062	H	3.67975	-2.57219	-0.515
C	-1.71614	-0.99422	-1.83045	H	5.94572	-0.2419	2.46062
N	1.79563	-1.73882	-0.87003	H	6.45957	0.66928	-1.71024
C	2.19808	-0.53469	-0.31854	H	4.65757	0.63242	-3.21544
C	1.27351	0.45716	-0.08707	H	3.56229	-0.73298	-2.91671
C	-0.09467	0.33222	-0.45055	H	3.03295	0.90688	-2.53558
C	3.64413	-0.2951	-0.04835	H	2.82757	-1.82619	2.19595
C	2.73288	-2.85039	-0.98575	H	3.89071	-0.95568	3.32611
C	-1.02382	1.34621	-0.01993	H	2.48984	-0.13986	2.59229
C	4.17359	-0.41719	1.25086	H	8.09953	0.45987	1.69939
C	5.53259	-0.14329	1.45344	H	8.46574	-0.18641	0.08208
C	6.37301	0.24613	0.40523	H	8.10483	1.53602	0.27957
C	5.81969	0.36201	-0.87834	H	-3.6445	2.49229	1.57373
C	4.47035	0.09952	-1.12638	H	-1.77529	4.41335	0.86425
C	3.90656	0.23561	-2.51897	H	0.88412	2.61668	-1.21357
C	3.3051	-0.85553	2.40202	H	0.49819	4.75326	-2.37913
C	7.8346	0.52868	0.63501	H	-0.96995	3.74463	-2.43202
N	-2.24649	1.17711	0.58788	H	-0.87332	5.1383	-1.31849
C	-2.70421	2.40519	1.04103	H	2.05692	4.61358	-0.2941
C	-1.7805	3.34078	0.70057	H	1.66603	3.46984	1.01437
N	-0.75621	2.68797	0.03433	H	0.69346	4.94604	0.79444
C	-2.96977	-0.05446	0.7988	H	-2.80702	-2.96205	2.53878
C	0.31385	3.40167	-0.70334	H	-4.90985	-3.35553	1.29954
C	-0.30168	4.3115	-1.76704	H	-5.80771	-1.62055	-0.21928
C	1.23213	4.14606	0.26294	H	-3.99997	1.56446	-1.04296
C	-2.45489	-1.00798	1.69662	H	-5.57643	1.05054	-2.83973
C	-3.17543	-2.19979	1.85082	H	-4.4096	-0.28365	-2.70385
C	-4.36393	-2.42001	1.16012	H	-6.05964	-0.46976	-2.07052
C	-4.86695	-1.4428	0.30374	H	-6.34265	2.33843	-0.79078
C	-4.18308	-0.2396	0.09815	H	-5.6639	1.97789	0.81687
C	-4.77665	0.81317	-0.83192	H	-6.77517	0.83063	0.05149
C	-5.22564	0.23882	-2.18449	H	-0.70122	0.12657	2.17233
C	-5.95212	1.53444	-0.14794	H	-0.76635	-0.2173	4.62089
C	-1.22338	-0.76053	2.55492	H	-2.33057	0.41284	4.04084
C	-1.6467	-0.44906	4.00097	H	-2.16495	-1.30743	4.4564
C	-0.23083	-1.92798	2.50068	H	0.64966	-1.70365	3.11846
H	-3.05796	-2.24888	-2.953	H	0.10753	-2.11749	1.47169
H	-1.43224	-4.14923	-3.06544	H	-0.67257	-2.85761	2.88985
H	0.81678	-3.90673	-2.11792				

Point Group	C1	ε + U_{corr}	-1599.414165 Eh
Electronic Energy (ε)	-1600.190555 Eh	ε + H_{corr}	-1599.413221 Eh
ε + *ZPE*	-1599.454405 Eh	ε + G_{corr}	-1599.528579 Eh

3f

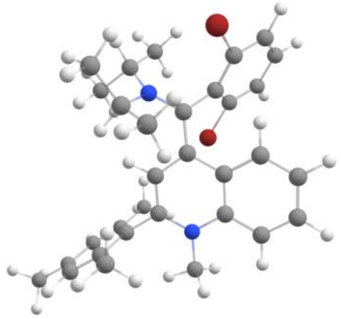

```
%nprocshared=20

%mem=50000MB

#p opt freq b3lyp def2svp
empiricaldispersion=gd3bj gfinput
iop(6/7=3)
```

Charge: 1 Multiplicity: 2

	Cartesian Coordinates				Cartesian Coordinates		
**Atoms**	*X*	*Y*	*Z*	**Atoms**	*X*	*Y*	*Z*
C	1.51794	3.27097	-2.23399	H	2.47287	3.63935	-2.61268
C	0.35864	4.05476	-2.35607	H	0.41135	5.04505	-2.81276
C	-0.86625	3.56846	-1.92734	H	-1.75253	4.18274	-2.06463
C	-0.96074	2.28875	-1.3389	H	2.33814	1.41076	-1.57549
C	0.22166	1.51112	-1.14472	H	-1.33469	-1.23651	0.19662
C	1.44303	2.02307	-1.64253	H	-3.27246	3.54645	-0.55536
N	-2.20453	1.7737	-0.96752	H	-3.55609	2.85734	-2.18386
C	-2.31564	0.50051	-0.48067	H	-4.26468	2.07279	-0.74449
C	-1.18657	-0.26703	-0.27114	H	5.8059	0.52756	0.19301
C	0.11707	0.21787	-0.50077	H	5.42611	2.31541	1.89149
C	-3.39442	2.61408	-1.12387	H	3.1241	2.71487	2.7735
C	1.23613	-0.54262	-0.01549	H	2.04808	-3.61898	0.8108
C	2.40089	0.18734	0.55	H	-0.50224	-2.79775	-0.95634
C	3.72749	-0.03036	0.10566	H	0.30751	-2.5538	-3.30409
C	4.80378	0.72352	0.57499	H	0.10133	-0.97482	-2.54621
C	4.58645	1.72313	1.52228	H	1.73282	-1.65646	-2.72843
C	3.30123	1.95664	2.01058	H	0.77421	-4.50079	-2.24822
C	2.23765	1.19356	1.53281	H	0.97978	-4.72316	-0.51077
Br	0.54292	1.49731	2.36369	H	2.28526	-3.97272	-1.47404
Br	4.1451	-1.34704	-1.21074	H	2.52762	-2.87102	3.03905
N	1.19734	-1.90546	-0.02506	H	3.39036	-1.84218	1.88687
C	1.51192	-2.73768	1.17907	H	1.93096	-1.23471	2.72372
C	0.56916	-2.6499	-1.17436	H	0.37623	-3.88475	2.63435
C	0.68515	-1.89958	-2.50572	H	-0.47876	-3.69116	1.09255
C	1.19532	-4.04161	-1.34313	H	-0.3522	-2.31384	2.22251
C	2.39451	-2.11198	2.25395	H	-5.74381	-0.47041	2.51268
C	0.18234	-3.1839	1.809	H	-6.24094	-1.7405	-1.56749
C	-3.65366	-0.05427	-0.13844	H	-4.57629	-1.35335	-3.19826
C	-4.12244	0.02645	1.18727	H	-3.77604	0.21996	-3.00787
C	-5.36949	-0.53504	1.48784	H	-2.90296	-1.261	-2.59634
C	-6.14665	-1.17377	0.51434	H	-3.01973	1.73007	1.96601
C	-5.64767	-1.24357	-0.79504	H	-3.85536	0.76393	3.20382
C	-4.41046	-0.69587	-1.14258	H	-2.36185	0.16321	2.44578
C	-3.89353	-0.7796	-2.55724	H	-7.45512	-2.87814	0.73505
C	-3.30264	0.70524	2.25648	H	-7.77693	-1.56276	1.88973
C	-7.48165	-1.78237	0.85429	H	-8.27149	-1.40584	0.18506

Point Group	C1	ε + U_{corr}	-6496.706973 Eh
Electronic Energy (ε)	-6497.346467 Eh	ε + H_{corr}	-6496.706029 Eh
ε + *ZPE*	-6496.743555 Eh	ε + G_{corr}	-6496.816425 Eh

3g

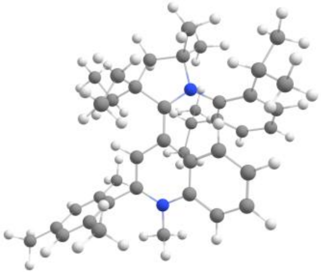

```
%nprocshared=20

%mem=50000MB

#p opt freq b3lyp def2svp
empiricaldispersion=gd3bj gfinput
iop(6/7=3)
```

Charge: 1 Multiplicity: 2

Atoms	Cartesian Coordinates X	Y	Z	Atoms	Cartesian Coordinates X	Y	Z
C	-1.67543	-3.24168	-1.43908	H	3.23841	-3.73628	-0.13183
C	-0.79009	-4.18938	-0.91016	H	3.84399	-2.4981	0.99942
C	0.41414	-3.78135	-0.35837	H	6.20377	1.0951	1.94625
C	0.7376	-2.41086	-0.29169	H	6.6342	-0.54793	-2.00467
C	-0.21981	-1.42748	-0.69514	H	-1.91273	4.2394	-0.50837
C	-1.38512	-1.8905	-1.33257	H	-1.53261	3.43142	1.01827
N	2.01502	-2.01763	0.10831	H	-0.20734	3.97664	-2.26682
C	2.41293	-0.72132	-0.0925	H	-1.30849	2.67609	-2.66762
C	1.47977	0.24676	-0.38997	H	0.75093	2.52408	-4.00524
C	0.08573	-0.0074	-0.51887	H	0.61213	1.08439	-2.9797
C	-0.84516	1.07752	-0.44446	H	1.75907	2.37735	-2.56053
C	2.94628	-3.00966	0.64146	H	0.87208	2.61206	1.06967
C	3.86049	-0.3776	-0.03471	H	1.68841	2.913	-0.46619
C	4.41024	0.26924	1.08918	H	1.7361	4.95275	0.74577
C	5.77121	0.6003	1.07293	H	0.62179	5.19898	-0.60785
C	6.58952	0.31327	-0.0251	H	-0.00855	4.96695	1.03816
C	6.01248	-0.32394	-1.13347	H	-4.28892	3.46571	0.96877
C	4.66123	-0.67589	-1.16139	H	-4.66167	1.72814	0.98017
N	-2.17988	0.9668	-0.15861	H	-3.31423	2.35247	1.95415
C	-2.89825	2.30921	-0.18776	H	-3.15643	2.18163	-2.37969
C	-1.71293	3.26222	-0.05291	H	-4.68112	2.13724	-1.46921
C	-0.4765	2.57355	-0.65779	H	-3.79452	3.65336	-1.62127
C	-0.34806	2.88718	-2.17987	H	-5.18394	-2.54793	0.31121
C	0.7545	2.1748	-2.96168	H	-4.1861	-3.23277	2.46978
C	0.77418	3.12618	0.09987	H	-2.34995	-1.89601	3.45266
C	0.76596	4.64148	0.33021	H	-3.98452	0.25025	-1.80171
C	-3.84533	2.45944	1.00123	H	-5.31302	-1.37093	-3.1583
C	-3.67629	2.56766	-1.49453	H	-3.72081	-1.99853	-2.72489
C	-2.7648	-0.15565	0.54814	H	-5.17039	-2.57583	-1.86869
C	-3.85603	-0.883	0.0022	H	-6.44066	0.40346	-1.92719
C	-4.34862	-1.97815	0.72117	H	-5.9734	0.81051	-0.25718
C	-3.7944	-2.3644	1.93597	H	-6.62591	-0.79174	-0.6259
C	-2.75489	-1.61751	2.47832	H	-0.86058	1.1529	1.93478
C	-2.23461	-0.49567	1.82052	H	-1.03406	1.58786	4.33887
C	-4.56464	-0.53317	-1.3044	H	-2.67953	1.51544	3.65984
C	-4.68859	-1.69079	-2.31038	H	-2.04044	0.14432	4.58194
C	-5.97644	0.01035	-1.00932	H	0.80984	0.1208	3.41632
C	-1.18412	0.31919	2.57044	H	0.52502	-0.9448	2.02111
C	-1.77197	0.92746	3.85671	H	-0.18638	-1.33447	3.59933
C	0.06177	-0.50974	2.91392	H	4.7981	-1.41113	-3.19134
C	4.06539	-1.34401	-2.37595	H	3.72094	-2.36759	-2.15538
C	3.56454	0.57928	2.29839	H	3.18888	-0.79	-2.74833
C	8.05062	0.6784	-0.03072	H	3.10581	-0.33179	2.71376
H	-2.59223	-3.56763	-1.92894	H	4.16386	1.04645	3.09154
H	-1.02415	-5.25411	-0.96737	H	2.73673	1.26237	2.05178
H	1.12112	-4.53222	-0.01381	H	8.35655	1.14664	0.91523
H	-2.0512	-1.15555	-1.77137	H	8.68016	-0.21186	-0.19029
H	1.87737	1.24293	-0.4832	H	8.27779	1.38232	-0.84796
H	2.47581	-3.54622	1.47675	H	3.23841	-3.73628	-0.13183

Point Group	C1	ε + U_{corr}	-1702.324311 Eh
Electronic Energy (ε)	-1703.225458 Eh	ε + H_{corr}	-1702.323367 Eh
ε + *ZPE*	-1702.368391 Eh	ε + G_{corr}	-1702.443701 Eh

3h

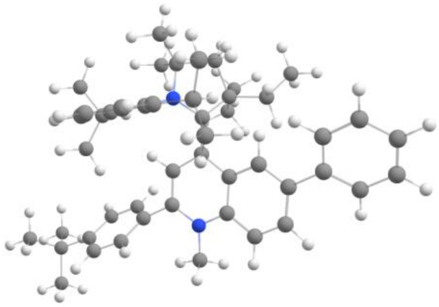

```
%nprocshared=20

%mem=50000MB

#p opt freq b3lyp def2svp
empiricaldispersion=gd3bj gfinput
iop(6/7=3)
```

Charge: 1 Multiplicity: 2

	Cartesian Coordinates				Cartesian Coordinates		
Atoms	*X*	*Y*	*Z*	Atoms	*X*	*Y*	*Z*
C	3.54455	-2.5615	0.26433	H	-1.2361	-4.66409	0.72072
C	2.87488	-3.80397	0.29894	H	-2.33828	-4.03231	-0.53163
C	1.49894	-3.88637	0.15777	H	-3.11251	-0.95587	-2.22162
C	0.72129	-2.73199	-0.06441	H	-5.56801	-0.91662	-2.17786
C	1.37481	-1.4646	-0.16102	H	-5.51436	-2.48974	1.8379
C	2.76154	-1.42527	0.06675	H	-3.06122	-2.5445	1.78652
N	-0.6679	-2.81949	-0.16711	H	2.23458	3.41994	-2.77429
C	-1.4284	-1.67614	-0.22727	H	2.96299	3.11112	-1.19191
C	-0.82077	-0.43839	-0.30887	H	1.42212	-0.17115	-3.39778
C	0.58175	-0.27038	-0.39177	H	0.07949	0.86458	-2.98432
C	1.07175	1.03059	-0.76325	H	0.76435	1.16321	-5.34318
C	-1.28908	-4.1419	-0.2468	H	0.90899	2.68776	-4.45911
C	-2.90882	-1.75796	-0.22767	H	2.36191	1.75533	-4.85761
C	-3.64057	-1.29998	-1.32939	H	3.86601	0.98309	-1.02728
C	-5.0368	-1.27676	-1.29796	H	3.30369	-0.34602	-2.04533
C	-5.74971	-1.69443	-0.16519	H	5.33079	0.82964	-2.91645
C	-5.00079	-2.15901	0.93364	H	4.01132	0.84216	-4.0918
C	-3.6107	-2.1969	0.90831	H	4.39278	2.31353	-3.16497
N	0.47751	2.15127	-0.28795	H	1.42462	5.43024	-0.82185
C	0.82542	3.38389	-1.09745	H	0.20603	4.89265	0.35847
C	2.0861	2.89821	-1.82271	H	1.89681	4.37075	0.52758
C	1.9654	1.36854	-1.97893	H	-0.6212	2.86148	-2.67086
C	1.1519	0.88498	-3.23453	H	-1.14464	4.19619	-1.62128
C	1.31663	1.66987	-4.53664	H	0.11981	4.46986	-2.82192
C	3.38403	0.74689	-1.98942	H	1.01211	2.25645	4.28694
C	4.31848	1.21714	-3.10616	H	-1.33935	2.92027	4.67831
C	1.10179	4.58429	-0.19734	H	-2.89889	3.09667	2.76411
C	-0.27988	3.7371	-2.10582	H	2.50737	1.47816	0.98119
C	-0.07157	2.26018	1.05082	H	4.29702	2.39902	2.39968
C	0.8202	2.10881	2.1476	H	3.14711	3.68813	1.9717
C	0.33404	2.35529	3.43783	H	3.11316	2.96919	3.59369
C	-0.98686	2.72513	3.66335	H	3.50858	-0.02747	2.66262
C	-1.85637	2.82974	2.58509	H	1.78229	-0.42152	2.4851
C	-1.42982	2.59941	1.26966	H	2.34942	0.50625	3.88983
C	2.276	1.66969	2.03776	H	-2.02156	2.41809	-0.77497
C	3.25891	2.745	2.52594	H	-4.3525	1.77086	-0.43712
C	2.48817	0.35525	2.80871	H	-3.3637	0.74193	0.62047
C	-2.48388	2.71598	0.17618	H	-4.26351	2.10614	1.30202
C	-3.67684	1.77651	0.43155	H	-3.65024	4.25949	-0.84281
C	-2.99478	4.16127	0.03664	H	-2.17734	4.8897	-0.05948
C	-7.918	-1.13976	-1.38206	H	-3.58148	4.45149	0.92152
C	-7.81927	-3.06548	0.2071	H	-9.01303	-1.12717	-1.2774
C	-7.28078	-1.64777	-0.08	H	-7.59866	-0.11404	-1.62297
C	5.01535	-2.46167	0.42746	H	-7.6753	-1.78845	-2.23779
C	-7.68508	-0.69604	1.06684	H	-8.91831	-3.04777	0.27198
C	5.62131	-1.28101	0.898	H	-7.43727	-3.46757	1.15724
C	7.00593	-1.18719	1.0347	H	-7.53616	-3.7643	-0.59533
C	7.82129	-2.27153	0.69882	H	-7.3036	0.32122	0.88614
C	7.23696	-3.44863	0.22424	H	-8.78198	-0.64074	1.1466
C	5.85145	-3.54223	0.09049	H	-7.29642	-1.03545	2.03858

H	3.43503	-4.71985	0.48967	H	5.00514	-0.42794	1.18452
H	1.02582	-4.86158	0.24525	H	7.45084	-0.26404	1.41297
H	3.23559	-0.45462	0.09649	H	8.90571	-2.19912	0.80562
H	-1.47355	0.4204	-0.40714	H	7.86439	-4.29929	-0.0508
H	-0.77435	-4.74544	-1.00679	H	5.41973	-4.46209	-0.30751
Point Group			C1	ε + U_{corr}			-1972.414054 Eh
Electronic Energy (ε)			-1973.430896 Eh	ε + H_{corr}			-1972.413110 Eh
ε + *ZPE*			-1972.463223 Eh	ε + G_{corr}			-1972.545545 Eh

3i

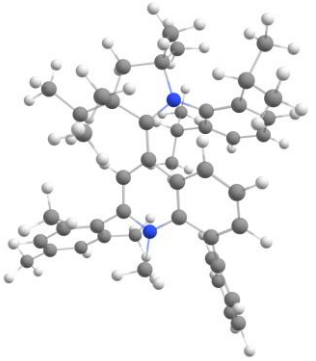

```
%nprocshared=20

%mem=50000MB

#p opt freq b3lyp def2svp
empiricaldispersion=gd3bj gfinput
iop(6/7=3)
```

Charge: 1 Multiplicity: 2

Atoms	Cartesian Coordinates *X*	*Y*	*Z*	Atoms	Cartesian Coordinates *X*	*Y*	*Z*
C	1.206416	2.70973	-2.040701	H	-4.02135	0.194561	-1.628398
C	0.035497	3.410645	-1.737516	H	-4.02639	1.627403	-0.563053
C	-1.069065	2.76832	-1.177258	H	3.697024	-3.959012	-0.017485
C	-0.994317	1.36126	-0.968903	H	2.727554	-3.404218	1.357608
C	0.274636	0.699719	-1.030065	H	2.742796	-3.906427	-2.216976
C	1.336539	1.387726	-1.648118	H	2.916765	-2.164744	-2.321755
N	-2.155646	0.600917	-0.772134	H	0.449824	-3.68687	0.879276
C	-2.060742	-0.696832	-0.335154	H	-0.136846	-3.684109	-0.785481
C	-0.823049	-1.286752	-0.175712	H	1.379416	-2.860815	-4.060793
C	0.402668	-0.656577	-0.512342	H	0.425494	-1.96365	-2.863018
C	-3.446482	1.068627	-1.305676	H	0.342859	-3.738566	-2.92642
C	1.63776	-1.348557	-0.254603	H	0.317114	-5.975517	-0.062372
N	2.801255	-0.788949	0.184655	H	1.440016	-5.559849	-1.36789
C	3.892845	-1.829681	0.445066	H	2.004875	-5.597926	0.317581
C	3.085807	-3.128217	0.353632	H	5.236461	-2.420068	2.01603
C	1.864597	-2.848329	-0.535604	H	4.985481	-0.66082	1.939669
C	2.20378	-2.958497	-2.060888	H	3.720813	-1.715227	2.616821
C	0.750429	-3.855921	-0.16767	H	4.692191	-1.801847	-1.61937
C	1.01902	-2.876496	-3.021177	H	5.769542	-1.057145	-0.412831
C	1.160418	-5.321737	-0.330674	H	5.575542	-2.805497	-0.460094
C	4.487488	-1.63434	1.838139	H	4.298659	3.605651	0.398028
C	5.040928	-1.852499	-0.581754	H	2.798462	4.127195	2.296054
C	2.862822	0.534574	0.780055	H	1.422668	2.331112	3.299154
C	3.70512	1.543162	0.241059	H	4.551578	0.321831	-1.290397
C	3.66042	2.822046	0.808998	H	5.539197	2.142546	-2.697252
C	2.825323	3.118319	1.879068	H	3.774235	2.171954	-2.65802
C	2.046045	2.108891	2.431681	H	4.706833	3.352995	-1.708069
C	2.055156	0.806057	1.9166	H	1.275125	-1.185312	2.12878
C	4.728554	1.31471	-0.866674	H	1.192704	-1.264357	4.585285
C	4.670824	2.305005	-2.040834	H	2.823611	-0.742331	4.091931
C	1.221795	-0.228831	2.665498	H	1.661084	0.445262	4.702471
C	1.761345	-0.462531	4.088367	H	-0.878658	-0.654397	3.085379
C	-0.256313	0.184975	2.741087	H	-0.631962	0.514142	1.766065
C	6.144508	1.348139	-0.2567	H	-0.400116	1.01432	3.450847
C	-3.305675	-1.454281	-0.034544	H	6.895176	1.015129	-0.99043
C	-3.648157	-2.56369	-0.84684	H	6.230068	0.715724	0.638229
C	-4.806372	-3.285702	-0.548998	H	6.407331	2.373212	0.04709
C	-5.632413	-2.952588	0.533197	H	-5.078552	-4.131542	-1.186284
C	-5.27773	-1.848267	1.313547	H	-5.911004	-1.568061	2.159707
C	-4.135457	-1.083437	1.047439	H	-4.142437	-0.078689	2.958271
C	-3.847158	0.115233	1.916945	H	-2.787864	0.392775	1.911274
C	-2.814038	-2.966847	-2.039443	H	-4.420467	0.995801	1.581278
C	-6.855114	-3.773225	0.847326	H	-1.957431	-3.592328	-1.741166
C	-2.212299	3.591183	-0.702884	H	-3.410994	-3.556188	-2.749277
C	-2.640993	3.474879	0.630817	H	-2.401304	-2.09983	-2.575424
C	-3.686346	4.263578	1.113017	H	-6.572882	-4.723049	1.332649
C	-4.315176	5.185899	0.270702	H	-7.537035	-3.242709	1.526911

C	-3.884774	5.321003	-1.052513	H	-7.409915	-4.031461	-0.067711
C	-2.838191	4.5326	-1.534985	H	-2.141038	2.765707	1.293694
H	2.034356	3.226387	-2.524225	H	-4.005433	4.165274	2.15322
H	-0.017932	4.487226	-1.909502	H	-5.134229	5.802786	0.646567
H	2.262731	0.850249	-1.828537	H	-4.36952	6.042128	-1.714324
H	-0.83205	-2.25874	0.294192	H	-2.515607	4.631018	-2.574244
H	-3.261087	1.720468	-2.164981				

Point Group	C1	ε + U_{corr}	-1933.151568 Eh
Electronic Energy (ε)	-1934.138361 Eh	ε + H_{corr}	-1933.150624 Eh
ε + *ZPE*	-1933.200250 Eh	ε + G_{corr}	-1933.281906 Eh

For TD-DFT calculations of Photoswitches

A preliminary conformational search for the *E*- and *Z*-isomers was performed using the GOAT module implemented in ORCA 6.0.1[34,35] at the GFN2-xTB[36,37] level of theory. The lowest-energy conformers obtained from this search were subsequently reoptimized at the r²SCAN-3c[38] level of theory utilizing standard settings, with solvent effects (thf) accounted for implicitly using the SMD[39] solvation model. Harmonic vibrational frequency calculations confirmed the optimized structures as minima (no imaginary frequencies).

***E*-2g**

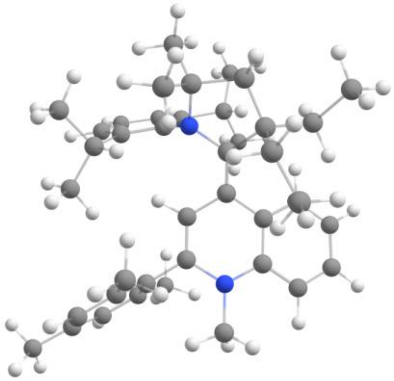

```
%maxcore 2000
%pal nprocs 20
end

!TIGHTOPT FREQ R2SCAN-3c  CPCM
!LargePrint printMOs printbasis

%cpcm
smd true
SMDsolvent "thf"
end
```

Charge: 0 Multiplicity: 1

	Cartesian Coordinates				Cartesian Coordinates		
**Atoms**	*X*	*Y*	*Z*	**Atoms**	*X*	*Y*	*Z*
C	1.83931063	1.14113788	4.41373192	H	-2.61773	-5.26494903	-1.05442486
C	1.42828678	0.03929617	5.15491748	H	2.87253682	-4.68082943	0.91084828
C	0.71636737	-0.98823549	4.54087278	H	2.6079229	-3.01063766	0.37532173
C	0.36148634	-0.89472472	3.18745184	H	2.09953779	-3.49934089	1.9864334
C	0.65831832	0.28587382	2.45323501	H	-0.06782416	-7.93595546	-1.03106627
C	1.47140463	1.2387453	3.07371525	H	-1.75523647	-7.5347545	-1.4185748
N	-0.25871348	-1.95319501	2.52464014	H	-0.45633837	-7.08460426	-2.52751625
C	-0.09660893	-2.02838697	1.13033054	H	-2.5200826	-1.87505099	-0.48302405
C	0.18416824	-0.92016125	0.40984193	H	-3.59928079	-3.27564776	-0.32142827
C	0.20279132	0.3894346	1.05622895	H	-2.88274369	-2.51480883	1.11404705
C	-0.26334979	-3.35722963	0.48392852	H	0.17942161	-3.69404587	3.68439405
C	0.83203128	-4.23210668	0.39399724	H	-1.30256722	-2.80949594	4.12905054
C	0.66178801	-5.46737575	-0.23345222	H	-1.26313327	-3.78245884	2.65161924
C	-0.56994775	-5.85365324	-0.76519498	H	-0.25731106	4.31730333	-0.33663056
C	-1.64827773	-4.97434136	-0.65324225	H	-2.02349128	4.26264017	-0.21482829
C	-1.51464227	-3.73014983	-0.03513214	H	-2.83108234	1.70432118	0.7783331
C	2.17409799	-3.84013994	0.94732173	H	-2.96190008	3.23509853	1.6157018
C	-0.72295235	-7.17491495	-1.46654862	H	-3.42895327	1.38144836	3.13647941
C	-2.69465325	-2.80409354	0.07256873	H	-1.93437968	2.14758636	3.6809172
C	-0.67405888	-3.12285255	3.28883031	H	-1.87617615	0.6152994	2.79240257
C	-0.207089	1.53778345	0.41028227	H	-0.08779158	3.02092433	2.98268359
C	-0.94760135	2.76871783	0.99824186	H	0.78563975	3.84864133	1.70421215
C	-1.12214815	3.64471007	-0.258135	H	-0.33961326	5.45595255	3.18626474
C	-1.12647459	2.72295288	-1.48343672	H	-1.07387706	5.5713528	1.58498865
N	-0.27493311	1.59038388	-0.99664287	H	-1.91708984	4.72354998	2.8923881
C	-2.34605349	2.33014804	1.53299857	H	-1.14729556	4.29941877	-2.93768979
C	-2.38661053	1.58010221	2.86027242	H	0.48843296	3.81175305	-2.45943354
C	-0.21979615	3.60790589	2.07317194	H	-0.45611839	2.7957739	-3.56540337
C	-0.93441505	4.9089253	2.44585524	H	-3.21558142	3.1516335	-1.86888457
C	-0.51499406	3.44093805	-2.68382009	H	-2.58251621	1.83229302	-2.8528601
C	-2.55270197	2.27876133	-1.8570988	H	-2.96131904	1.55214185	-1.15160549
C	0.75595452	1.05619364	-1.83956056	H	4.09727213	1.33877095	-2.45519606
C	2.09832121	1.48783038	-1.68724362	H	3.52094869	-0.22268268	-4.27695597
C	3.06947164	1.0035266	-2.5670957	H	1.18894594	-0.99573194	-4.53110432
C	2.75090306	0.12736067	-3.59443644	H	1.69499281	2.78152857	-0.03777917
C	1.44031472	-0.29902158	-3.73587257	H	3.82839147	2.46278474	1.14995905
C	0.4336718	0.13454785	-2.86823084	H	3.14175892	0.87132989	0.76017901
C	2.56285281	2.46629519	-0.6227475	H	4.48174225	1.52267166	-0.19908489
C	3.55844303	1.78769131	0.32945529	H	4.12811912	3.49602187	-1.75767168
C	3.19880568	3.72766186	-1.22491893	H	3.44416616	4.43818109	-0.4264688
C	-0.95699952	-0.4401965	-3.07743031	H	2.52586273	4.23001248	-1.92704522
C	-1.56121995	0.01630713	-4.41505867	H	-1.59004828	-0.08817488	-2.257021
C	-0.95085994	-1.97771198	-3.04232622	H	-2.60608534	-0.30690532	-4.49499697
H	2.44882821	1.91792069	4.86714068	H	-1.52811377	1.10198321	-4.54251764
H	1.69356979	-0.05086375	6.20494351	H	-1.01032065	-0.42962295	-5.25182003
H	0.45861461	-1.86910997	5.11721431	H	-0.4280483	-2.39234958	-3.91149514
H	1.81748922	2.07842966	2.48515342	H	-1.97924459	-2.35728377	-3.06405611

H	0.30027426	-1.03267143	-0.65968132	H	-0.46747427	-2.3682336	-2.14269765
H	1.51061377	-6.14529726	-0.30598032				
Point Group			C1	ε + ZPE + U_{corr}			-1703.09375602 Eh
Electronic Energy (ε)			-1703.98540470 Eh	$U + H_{corr}$			-1703.09281181 Eh
ε + *ZPE*			-1703.138226 Eh	$U + G_{corr}$			-1703.20533269 Eh

Z-2g

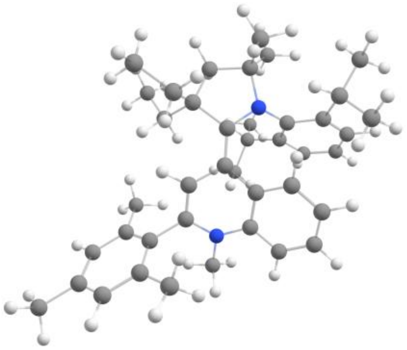

```
%maxcore 2000
%pal nprocs 20
end

!TIGHTOPT FREQ R2SCAN-3c  CPCM
!LargePrint printMOs printbasis

%cpcm
smd true
SMDsolvent "thf"
end
```

Charge: 0 Multiplicity: 1

	Cartesian Coordinates				Cartesian Coordinates		
**Atoms**	*X*	*Y*	*Z*	**Atoms**	*X*	*Y*	*Z*
C	2.24430487	1.19103632	3.19295895	H	-2.21298421	-5.83786984	3.44940844
C	3.00684585	0.04552431	3.3904486	H	-0.62662012	-4.9867525	-2.11022568
C	2.65617386	-1.14178704	2.75711717	H	-1.43123655	-3.43186011	-1.86775075
C	1.55823791	-1.18104161	1.88566631	H	0.25675588	-3.61602193	-1.40050543
C	0.84702668	0.01194627	1.57642177	H	-2.9210074	-8.14262651	0.54595987
C	1.17466984	1.15884159	2.3014005	H	-2.34912974	-8.24513886	2.22238021
N	1.1212985	-2.38648081	1.3434202	H	-3.89211869	-7.47212376	1.86728807
C	-0.22457509	-2.46862476	0.95702139	H	-1.41093268	-2.40892815	3.45783537
C	-0.89103863	-1.35206553	0.59866116	H	-1.4601641	-3.87625823	4.46010266
C	-0.23912858	-0.03910121	0.57470389	H	0.06704635	-3.31184188	3.76310021
C	-0.89243242	-3.79269482	1.07261505	H	1.52213106	-4.37305372	0.84811825
C	-1.13752455	-4.58076197	-0.06168863	H	2.95677838	-3.3808645	1.15339887
C	-1.77693476	-5.8154802	0.09307137	H	1.96859964	-3.96085649	2.51820391
C	-2.17279171	-6.28254834	1.34415175	H	-2.62300228	2.40821177	-2.54673812
C	-1.90981096	-5.48736191	2.46390446	H	-1.10591565	1.67351118	-3.06091711
C	-1.27162187	-4.25433094	2.35006343	H	-2.52247806	-1.24994587	-1.50740625
C	-0.70852283	-4.13211446	-1.43188624	H	-0.88691317	-0.96581683	-2.05174828
C	-2.86813416	-7.60697348	1.49800148	H	-2.57922841	-1.47003923	-3.86032912
C	-1.00784699	-3.42205546	3.57429787	H	-1.68416386	0.01471427	-4.16100413
C	1.93255368	-3.58698689	1.48368607	H	-3.34822375	0.09330486	-3.56768633
C	-0.62625495	0.94434701	-0.30863598	H	-3.13027138	0.18277971	0.4061672
C	-1.86270103	0.80846647	-1.23348533	H	-3.1219557	1.91068476	0.14789422
C	-1.67625741	1.99757563	-2.18576693	H	-5.31022177	0.99899668	-0.43860626
C	-0.83612023	3.05373272	-1.46139036	H	-4.56465514	1.5844997	-1.92748318
N	-0.02570423	2.20407214	-0.5177609	H	-4.61044943	-0.15260155	-1.57744179
C	-1.89327019	-0.53175569	-2.04111209	H	-2.16703498	3.67683517	0.17625827
C	-2.4049309	-0.45485568	-3.48453367	H	-1.17056494	4.98591182	-0.47626002
C	-3.15113024	0.94993087	-0.37641879	H	-2.54268497	4.38653033	-1.40214588
C	-4.47560093	0.8378553	-1.13049093	H	0.62873861	3.08420658	-3.06966855
C	-1.72584439	4.08233852	-0.7377913	H	-0.61724683	4.3416516	-3.15984439
C	0.03654783	3.78623054	-2.4775692	H	0.71656977	4.50021195	-2.00862793
C	1.3868983	2.41777721	-0.3888927	H	4.35646489	1.08105068	-1.39641257
C	2.28979816	1.57592337	-1.08581183	H	5.22857025	2.86834891	0.068097
C	3.66159503	1.7384763	-0.87972333	H	3.66276892	4.4361829	1.150344
C	4.1568782	2.74990187	-0.06901975	H	0.76974611	0.60687269	-2.22618549
C	3.27155461	3.62587035	0.5408734	H	2.02052996	0.23757182	-4.26911872
C	1.88883836	3.47618825	0.41371368	H	2.28653955	1.9255089	-3.79326741
C	1.85654402	0.55568615	-2.1274418	H	3.54577164	0.71583215	-3.51052308
C	2.46293278	0.88483492	-3.50238327	H	3.31474929	-0.9920454	-1.64383317
C	2.22800327	-0.88085335	-1.74122309	H	1.89306763	-1.57959725	-2.51738654
C	1.00639912	4.48397283	1.14144814	H	1.76995453	-1.17418623	-0.79417469
C	1.31939926	4.636889	2.63904528	H	-0.02796967	4.14118533	1.06007276
C	1.12723632	5.87797361	0.49950886	H	2.35787198	4.9391357	2.81145808
H	2.49290773	2.10698404	3.72009	H	0.67774219	5.42004174	3.05959273
H	3.8594996	0.06074212	4.06403317	H	1.13031425	3.72521864	3.20840628
H	3.22145328	-2.04304264	2.96490352	H	0.41368658	6.57144116	0.96012228
H	0.56867357	2.04215038	2.1432105	H	0.94256332	5.86845535	-0.57724194

H	-1.94283261	-1.46803996	0.37537268	H	2.13438435	6.28152595	0.65943835
H	-1.95943064	-6.42830372	-0.78790075				

Point Group	C1	ε + ZPE + U_{corr}	-1703.09277620 Eh
Electronic Energy (ε)	-1703.98515178 Eh	U + H_{corr}	-1703.09183199 Eh
ε + *ZPE*	-1703.13687807 Eh	U + G_{corr}	-1703.20339580 Eh

E-2h

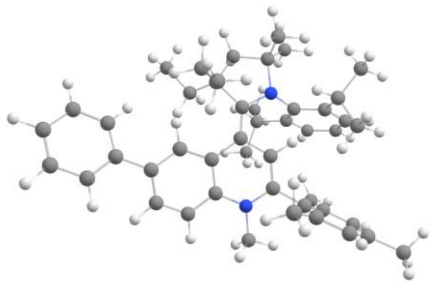

```
%maxcore 2000
%pal nprocs 20
end

!OPT FREQ R2SCAN-3c  CPCM
!LargePrint printMOs printbasis

%geom
       Calc_Hess true
       Recalc_Hess  10
end

%cpcm
smd true
SMDsolvent "thf"
end
```

Charge: 0 Multiplicity: 1

	Cartesian Coordinates				Cartesian Coordinates		
Atoms	X	Y	Z	Atoms	X	Y	Z
C	-3.17186441	-0.21385546	1.85259595	H	2.57599066	-4.91114255	-4.18044083
C	-3.67702296	-1.44130977	1.41888916	H	5.20065032	-5.83689515	-2.63561987
C	-2.98340877	-2.19392219	0.48426184	H	4.32072437	-7.09028937	-1.75794363
C	-1.78031423	-1.73519973	-0.07766713	H	3.98476742	-6.80107487	-3.47894197
C	-1.31162832	-0.42100081	0.23673812	H	-0.815847	-4.45190555	-1.79723582
C	-1.99706787	0.25657099	1.2501332	H	-1.63365157	-4.50899816	-0.22548001
N	-1.03234279	-2.57880302	-0.89717215	H	-2.49023067	-3.89375528	-1.66412258
C	0.32362939	-2.26966297	-1.08153709	H	0.59941741	4.69367425	-0.5544054
C	0.75364089	-1.00835025	-0.88829513	H	-0.1771293	3.9695205	-1.96912998
C	-0.11578916	0.0843352	-0.47903609	H	-0.87058536	3.00943304	1.88869823
C	1.27309959	-3.35363455	-1.45435181	H	0.56272077	3.8688651	1.41598895
C	2.00365385	-4.01016933	-0.44915119	H	-1.99847191	2.77468546	-1.54334178
C	2.94358036	-4.97569893	-0.81508836	H	-2.4125079	2.13016187	0.02333479
C	3.1718106	-5.30580569	-2.151769	H	1.82300279	1.58899049	1.45077402
C	2.42110869	-4.65698565	-3.13330289	H	0.38111173	0.90265402	2.17344915
C	1.4712393	-3.68848648	-2.80529862	H	1.22770929	2.21664603	2.99310971
C	4.21967599	-6.31704921	-2.52603099	H	-1.8689125	5.08637331	-0.60931896
C	-1.52169021	-3.92933775	-1.15336137	H	-3.42449536	4.33403285	-0.24265508
C	0.25228634	1.38055445	-0.77045333	H	-2.19671997	4.47115244	1.01661802
N	1.36824773	1.66033226	-1.59833646	H	1.36272327	3.5233825	-3.66534968
C	1.74222199	3.11889009	-1.55175206	H	2.30526187	4.75484483	-2.81109116
C	0.4319398	3.75088957	-1.08272994	H	3.072817	3.23308972	-3.28825102
C	-0.2988804	2.70445199	-0.21109714	H	3.03209524	4.48959788	-0.49904619
C	1.68535525	0.93186978	-2.80165503	H	2.6627794	3.04027293	0.44088654
C	0.05769249	2.90126416	1.31455506	H	3.82208662	2.96093229	-0.89295522
C	-1.81875877	2.90142609	-0.46902992	H	1.04396919	-3.27130012	-4.87400895
C	0.91799947	1.8407192	2.01047248	H	-0.39246277	-3.35979696	-3.84180067
C	-2.34715444	4.27453788	-0.05045476	H	0.6491528	-1.94403656	-3.76187216
C	2.14368083	3.67620063	-2.91603635	H	0.73797385	-3.59246015	1.25568358
C	2.88121783	3.40563598	-0.56301849	H	2.25557924	-4.43009743	1.64536458
C	0.65489547	-3.03080943	-3.88068463	H	2.25668598	-2.70383535	1.24926453
C	1.8009862	-3.67095535	1.00235508	H	4.32217863	-0.5934713	-4.33250998
C	2.97755853	0.3791709	-2.96830912	H	2.72035738	-0.64697185	-6.21207653
C	3.32586569	-0.17941042	-4.20163845	H	0.43621763	0.23615299	-5.89628686
C	2.42927287	-0.2148226	-5.25803887	H	-0.7543644	1.92187494	-2.82469057
C	1.14888242	0.28707522	-5.07706016	H	-2.07744829	2.70826385	-4.69754071
C	0.75309163	0.85935126	-3.86609105	H	-0.38361162	3.12681381	-5.01038298
C	-0.67354562	1.35662933	-3.75309216	H	-1.13650163	1.76456804	-5.8564529
C	-1.08313585	2.29158037	-4.89735904	H	-2.66676895	0.52265881	-3.47568128
C	-1.64496098	0.16987858	-3.66216216	H	-1.36448384	-0.51118309	-2.85221181
C	4.01983349	0.34269464	-1.86270331	H	-1.65024382	-0.3991007	-4.60015391
C	4.47182172	-1.09435808	-1.55051606	H	3.56158838	0.75749018	-0.95813833
C	5.25462959	1.18325475	-2.22466782	H	5.14634462	-1.09381402	-0.68611549
C	-3.8456255	0.5687648	2.90792102	H	3.63125653	-1.75753887	-1.32214019
C	-3.82019483	1.97282433	2.90259257	H	5.01710303	-1.52982769	-2.39556544
C	-4.44776378	2.70534899	3.90422886	H	4.99009185	2.19838997	-2.53545099
C	-5.1222127	2.05561841	4.93567149	H	5.80567254	0.71839861	-3.050715

C	-5.15929507	0.66250031	4.9532817	H	5.9349697	1.25237253	-1.36760466
C	-4.52802292	-0.07154649	3.95527762	H	-3.3234029	2.50009067	2.09336972
H	-4.61396789	-1.82513395	1.81297953	H	-4.41787778	3.79155538	3.87191593
H	-3.38408964	-3.15880031	0.20041545	H	-5.61301398	2.62815995	5.71782428
H	-1.59633953	1.19051536	1.60967281	H	-5.67395212	0.14216061	5.75690465
H	1.81044948	-0.81557641	-1.0001544	H	-4.54303054	-1.15715509	4.00167504
H	3.5112767	-5.48217513	-0.03625787				
Point Group			C1	ε + ZPE + U_{corr}			-1934.01851930 Eh
Electronic Energy (ε)			-1934.99408200 Eh	$U + H_{corr}$			-1934.01757509 Eh
ε + *ZPE*			-1934.06612664 Eh	$U + G_{corr}$			-1934.13624141 Eh

Z-2h

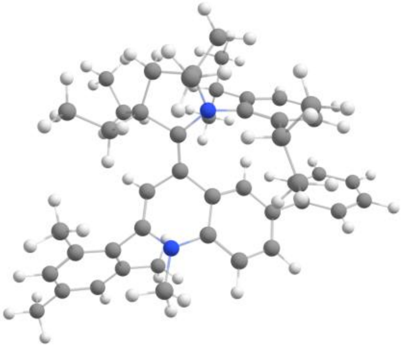

```
%maxcore 2000
%pal nprocs 20
end

!TIGHTOPT FREQ R2SCAN-3c  CPCM
!LargePrint printMOs printbasis

%cpcm
smd true
SMDsolvent "thf"
end
```

Charge: 0 Multiplicity: 1

	Cartesian Coordinates				Cartesian Coordinates		
Atoms	*X*	*Y*	*Z*	Atoms	*X*	*Y*	*Z*
C	-2.99654541	-0.91733214	-0.87262388	H	5.326152	-5.31283632	0.02538855
C	-3.02107973	-2.16424243	-1.50399	H	5.376409	-7.12266674	1.56470937
C	-1.83761015	-2.8266716	-1.79803514	H	4.52084932	-6.724383	3.06419247
C	-0.60137361	-2.2330426	-1.50766128	H	3.81471287	-7.87835431	1.93536729
C	-0.54744298	-0.88966468	-1.03971474	H	1.62724634	-4.61389753	-2.30089651
C	-1.75492896	-0.30598898	-0.65517293	H	0.0568608	-5.03564459	-1.58080059
N	0.58567674	-2.9579105	-1.57739804	H	0.13303403	-4.23705261	-3.17224771
C	1.57958184	-2.60277431	-0.64117897	H	3.69980613	3.00890322	-2.24122406
C	1.69571148	-1.30984675	-0.27802349	H	2.67308145	3.52334714	-0.90449552
C	0.8076651	-0.29440444	-0.87413062	H	3.39181174	0.51211924	-3.01802431
C	2.32956391	-3.71013574	0.01231388	H	3.46086674	-0.61056748	-1.67754818
C	1.67212563	-4.46513707	1.00793701	H	2.34094615	1.36099613	1.02286587
C	2.35576604	-5.50296195	1.63626931	H	3.72783539	0.38731198	0.57857358
C	3.6748836	-5.82185831	1.2995471	H	5.46990825	1.6769328	-2.1302715
C	4.30324614	-5.0718161	0.30924323	H	5.71900199	-0.02719746	-2.51086284
C	3.65059959	-4.01959739	-0.34297439	H	5.54893016	0.47767076	-0.82847302
C	4.38790262	-6.94621539	1.99830597	H	3.65690776	3.42391345	0.91912248
C	0.59358206	-4.2844416	-2.18031179	H	4.58338313	2.22025001	1.80927621
C	1.28050337	0.93554514	-1.26569718	H	5.01254664	2.60137329	0.13763784
N	0.59568206	1.95909049	-1.9289709	H	0.82113264	4.71432907	-1.62719123
C	1.54444222	2.90147692	-2.60515695	H	1.80159611	4.97258593	-3.08037621
C	2.77592827	2.78148433	-1.70130365	H	0.1225016	4.43316051	-3.23743669
C	2.77248679	1.35457657	-1.12024556	H	1.98832398	1.39312834	-4.14495526
C	-0.76008874	2.35837054	-1.71327134	H	1.034106	2.76290851	-4.72742527
C	3.67813639	0.42144844	-1.9650054	H	2.75688416	2.97059206	-4.40217189
C	3.22732663	1.33957961	0.37698669	H	5.18265637	-3.85779333	-1.84156358
C	5.1825704	0.65847653	-1.84457716	H	3.70377049	-2.94554908	-2.21980366
C	4.17074392	2.46217874	0.82285232	H	4.83277919	-2.3407448	-1.00814111
C	1.02963033	4.33721241	-2.6317537	H	-0.44611633	-4.31656012	0.58504636
C	1.84467279	2.47275646	-4.05361625	H	-0.0564557	-4.7582303	2.25519631
C	4.37730642	-3.25292857	-1.41366631	H	0.15881477	-3.08797756	1.68732324
C	0.25852882	-4.14366639	1.40739924	H	-3.78361552	2.47239482	-3.26852817
C	-1.72451073	2.17227399	-2.7238667	H	-4.40595827	3.56870705	-1.14757
C	-3.02954193	2.62045102	-2.49940395	H	-2.7129362	3.89300319	0.61793938
C	-3.3834222	3.23611816	-1.3080904	H	0.88703806	3.01787653	0.23783026
C	-2.42735876	3.4158928	-0.31629082	H	-0.03805549	5.38957583	0.34283439
C	-1.10799875	2.99830054	-0.4986015	H	-1.05344993	4.87893791	1.70090787
C	-0.11403443	3.21672402	0.62886932	H	0.70595493	4.81235097	1.84559757
C	-0.13021171	4.6589405	1.15347775	H	0.36018811	2.41618246	2.59500374
C	-0.3624123	2.24032446	1.78886085	H	-0.2663608	1.19714849	1.47074712
C	-1.41191219	1.48390882	-4.03869313	H	-1.36831982	2.38108465	2.20348684
C	-2.24147484	0.20808555	-4.23293847	H	-0.36054683	1.18571036	-4.01369147
C	-1.62688034	2.43527753	-5.22501081	H	-1.98558827	-0.26200788	-5.19016588
C	-4.24087169	-0.24672694	-0.447678	H	-2.0476337	-0.51451012	-3.43593046
C	-4.30123117	0.43738591	0.77548172	H	-3.31623139	0.4248821	-4.24326632
C	-5.46554748	1.083385	1.1756738	H	-2.69254835	2.65661817	-5.35881209
C	-6.59718054	1.06131399	0.36234518	H	-1.26274623	1.97875245	-6.15313849

C	-6.55224297	0.38159414	-0.85327387	H	-1.10590304	3.38821887	-5.08427238
C	-5.38781292	-0.26626019	-1.25415637	H	-3.42823647	0.45080872	1.42350104
H	-3.96859204	-2.6581904	-1.7034812	H	-5.49103529	1.60296163	2.13019114
H	-1.8816235	-3.83330352	-2.1984979	H	-7.50649012	1.56807602	0.67388636
H	-1.73961028	0.67374469	-0.19910652	H	-7.42673498	0.36292361	-1.49861091
H	2.41000249	-1.06624336	0.49838907	H	-5.35895283	-0.7740369	-2.21508847
H	1.8500069	-6.0776468	2.41079				

Point Group	C1	ε + ZPE + U_{corr}	-1934.01767502 Eh
Electronic Energy (ε)	-1934.99486546 Eh	$U + H_{corr}$	-1934.01673081 Eh
ε + *ZPE*	-1934.06641579 Eh	$U + G_{corr}$	-1934.13749483 Eh

E-2i

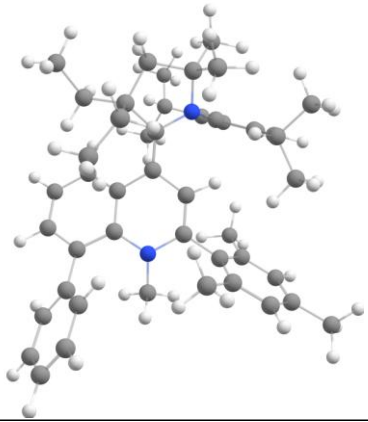

```
%maxcore 2000
%pal nprocs 20
end

!TIGHTOPT FREQ R2SCAN-3c  CPCM
!LargePrint printMOs printbasis

%cpcm
smd true
SMDsolvent "thf"
end
```

Charge: 0 Multiplicity: 1

	Cartesian Coordinates				Cartesian Coordinates		
Atoms	*X*	*Y*	*Z*	Atoms	*X*	*Y*	*Z*
C	3.55594685	-1.33384647	-1.1549011	H	-1.45446199	-4.04381574	-0.83148259
C	3.16092979	-2.27163333	-2.09470883	H	-1.14081087	-3.99911294	-2.5802361
C	1.80119827	-2.52765648	-2.33099336	H	1.02535146	4.83331398	-0.97301792
C	0.832744	-1.83022402	-1.5629119	H	1.69140522	4.08973488	0.49034746
C	1.22989013	-0.66176514	-0.84405941	H	-0.30594834	5.72275849	1.40919617
C	2.58990622	-0.52614515	-0.55822912	H	-1.44286857	4.58080583	2.14256655
N	-0.47939251	-2.29494556	-1.45251836	H	0.30709471	4.33566662	2.32746039
C	-1.3893692	-1.55916041	-0.67581581	H	-1.68504659	3.79718528	-1.4775478
C	-1.06783251	-0.36085828	-0.13986729	H	-2.47835537	4.54892406	-0.0868768
C	0.20770189	0.28145151	-0.36666851	H	-1.21622231	5.42048177	-0.95303331
C	-0.73851602	-3.73517517	-1.5957887	H	0.93401037	3.65130831	-2.93076221
C	0.36965331	1.64984643	-0.20707123	H	-0.42417828	2.65507278	-2.44660329
N	-0.54195487	2.39342813	0.56835679	H	3.126452	2.34760464	-0.04273446
C	-0.43668375	3.87213815	0.33451683	H	3.01999157	1.5301706	-1.59474481
C	0.94784637	3.96843173	-0.30884377	H	0.62988514	1.68615328	-4.40882197
C	1.23208687	2.63988323	-1.03593203	H	0.85940226	0.57922697	-3.05205245
C	-1.07688969	1.90174584	1.80696522	H	2.20339985	1.58504305	-3.61514674
C	-0.47511546	4.66456564	1.63834817	H	4.58786722	3.38917176	-1.75531138
C	-1.52420503	4.42883763	-0.60218997	H	3.20072914	3.74961238	-2.78494831
C	0.6683137	2.67967778	-2.49384175	H	3.37743122	4.55613671	-1.21842884
C	2.76424365	2.45283052	-1.07368202	H	-4.04387651	1.37386109	3.40467826
C	1.1230725	1.56871342	-3.43676528	H	-2.51735701	0.683441	5.21905074
C	3.51357112	3.6060272	-1.74516283	H	-0.07714759	0.7614858	4.85935184
C	-2.47918594	1.84250584	2.01022342	H	1.58465394	1.95781489	1.77173424
C	-2.96933894	1.41226936	3.2461751	H	3.03713187	2.48243798	3.65554606
C	-2.11698536	1.02070329	4.26648705	H	1.54590243	3.42303021	3.83795957
C	-0.74608026	1.06293587	4.05766826	H	1.81241813	1.97956138	4.82787183
C	-0.20169849	1.50513814	2.84927285	H	2.96537559	0.11762187	2.64947472
C	1.31331765	1.53504741	2.74330631	H	1.42106746	-0.54515035	2.07433786
C	1.95717762	2.40849165	3.82998422	H	1.70442595	-0.32409286	3.81065146
C	1.88235078	0.11034601	2.81971009	H	-2.95721516	2.43357374	0.02305425
C	-3.50011748	2.21309965	0.94645737	H	-5.15975501	1.3695817	-0.16365414
C	-4.47867806	1.06783482	0.64115603	H	-3.96374003	0.1590402	0.31704859
C	-4.30532842	3.4535325	1.36688392	H	-5.09098282	0.81588006	1.51429275
C	-2.77454354	-2.07777994	-0.55221506	H	-4.93748058	3.79988854	0.54060592
C	-3.26407678	-2.49923647	0.69722029	H	-3.66243268	4.28211794	1.67889622
C	-4.59377869	-2.91615137	0.79894631	H	-4.96286698	3.21213207	2.21055997
C	-5.44733919	-2.92351157	-0.30311256	H	-4.96929549	-3.24727865	1.76568222
C	-4.94115792	-2.50403767	-1.53566811	H	-5.59639522	-2.49347757	-2.4053632
C	-3.61975604	-2.08368103	-1.67989319	H	-3.96676006	-1.39558718	-3.68680512
C	-3.12680233	-1.61278496	-3.02083128	H	-2.51513104	-0.70859255	-2.92362553
C	-2.39052276	-2.50899827	1.92272448	H	-2.50327808	-2.37138664	-3.51050688
C	-6.88588283	-3.33913417	-0.16643121	H	-2.29288522	-1.5036105	2.35160866
C	1.44250478	-3.40560961	-3.46808497	H	-2.81649029	-3.15859588	2.69349241
C	0.46669943	-2.99834403	-4.38966979	H	-1.37620817	-2.85231366	1.69311147
C	0.16736596	-3.77512595	-5.50313985	H	-7.53810096	-2.46088314	-0.07603708

C	0.83818679	-4.97681342	-5.72315925	H	-7.2236479	-3.90296031	-1.04214159
C	1.81332477	-5.39046793	-4.81750196	H	-7.03940897	-3.95579212	0.72415702
C	2.11372145	-4.61381392	-3.70313354	H	-0.0546089	-2.05728614	-4.23101943
H	4.60754352	-1.19153155	-0.92245158	H	-0.58901033	-3.43642383	-6.20657004
H	3.90179436	-2.81517774	-2.67502612	H	0.60391847	-5.58507355	-6.59243498
H	2.88653896	0.23511704	0.15070868	H	2.33984623	-6.3281702	-4.97551465
H	-1.85842936	0.15764459	0.37958983	H	2.86415081	-4.95552675	-2.99454556
H	0.18959717	-4.29185335	-1.44302425				
Point Group			C1	ε + ZPE + U_{corr}			-1934.00678632 Eh
Electronic Energy (ε)			-1934.98388614 Eh	$U + H_{corr}$			-1934.00584211 Eh
ε + *ZPE*			-1934.05552299 Eh	$U + G_{corr}$			-1934.12633017 Eh

Z-2i

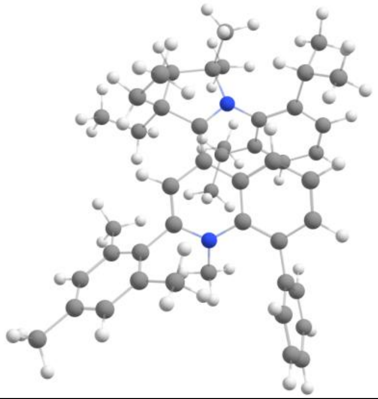

```
%maxcore 2000
%pal nprocs 20
end

!TIGHTOPT FREQ R2SCAN-3c  CPCM
!LargePrint printMOs printbasis

%cpcm
smd true
SMDsolvent "thf"
end
```

Charge: 0 Multiplicity: 1

	Cartesian Coordinates				Cartesian Coordinates		
Atoms	*X*	*Y*	*Z*	Atoms	*X*	*Y*	*Z*
C	-3.59510806	0.02556225	-0.55627645	H	1.21972428	-3.69514775	0.27681265
C	-3.67151716	-1.33304877	-0.2893804	H	-0.32564797	-3.56148293	1.12426406
C	-2.52485873	-2.13010587	-0.22861155	H	2.09352236	4.59711257	-0.34237445
C	-1.25700398	-1.52408453	-0.38888729	H	2.62271745	3.76348167	1.1101536
C	-1.16932251	-0.10478616	-0.52030949	H	1.09479768	5.20327574	2.59912472
C	-2.34842646	0.62562601	-0.67281513	H	-0.37470697	4.28413803	2.95472144
N	-0.07114627	-2.25628235	-0.50146422	H	1.21025535	3.47458652	2.95783999
C	0.98737019	-1.68037511	-1.22073231	H	-1.08545711	5.50946698	0.86225526
C	1.10074949	-0.33903803	-1.30515199	H	0.50534653	6.00281939	0.30819479
C	0.15961785	0.54035988	-0.60422599	H	-0.49451089	4.97146436	-0.72601476
C	0.14491747	-3.55246929	0.13812738	H	2.37361742	1.75122111	-2.46772016
C	0.5283479	1.77935398	-0.12244232	H	2.98677257	3.32851428	-2.04891638
N	-0.22157986	2.63873121	0.70567987	H	2.96883873	1.55002537	1.21871685
C	0.47179173	3.95720075	0.96302881	H	2.96354021	0.53139971	-0.20425039
C	1.86080977	3.76193529	0.32389666	H	1.15839964	3.41277885	-3.75559877
C	1.89258451	2.42131051	-0.43850034	H	0.74818205	4.3464082	-2.31718082
C	-1.18790951	2.11624598	1.64164593	H	0.01673226	2.75144745	-2.57524352
C	0.60146785	4.2344329	2.46160936	H	4.58429212	3.13797203	0.00560083
C	-0.2092211	5.17201136	0.31003036	H	5.21475681	1.52148114	0.33507593
C	2.10696362	2.68019212	-1.95463616	H	4.68478267	1.9397698	-1.29532603
C	3.06190251	1.56667578	0.12644585	H	-4.48039154	2.30439269	2.51054981
C	0.93893916	3.33335077	-2.68484657	H	-3.82700727	0.42299459	3.96452281
C	4.45820387	2.07488172	-0.23279122	H	-1.45924731	-0.28293112	4.05218235
C	-2.53357044	2.56774296	1.64522591	H	1.28010961	1.34943118	2.18062743
C	-3.45046697	1.95836147	2.50584377	H	2.12490659	0.92387039	4.40778966
C	-3.08804927	0.90573475	3.33021385	H	0.74853774	2.02946966	4.59294448
C	-1.76162863	0.50160817	3.36292872	H	0.56249172	0.31065594	4.96690304
C	-0.79303239	1.11466183	2.5658038	H	2.01140321	-0.9576854	2.69727086
C	0.65294178	0.72933818	2.821697	H	0.75199663	-0.96035126	1.44821374
C	1.0397646	1.02084141	4.28205358	H	0.35291176	-1.40996922	3.11898361
C	0.95579781	-0.73715503	2.49706119	H	-2.26546831	3.99740652	0.09558069
C	-3.04493071	3.73118342	0.81009627	H	-4.65594219	4.38259525	-0.46692216
C	-4.31574494	3.44864795	-0.0038653	H	-4.14484096	2.73349187	-0.81104934
C	-3.32474426	4.9286813	1.73787201	H	-5.13601138	3.07682139	0.61875908
C	1.88669009	-2.6127996	-1.95482104	H	-3.50953292	5.83801444	1.15352679
C	1.37281067	-3.31535053	-3.06749574	H	-2.49975292	5.12256541	2.4306085
C	2.20646988	-4.17282361	-3.77966371	H	-4.21941331	4.73256163	2.34131835
C	3.54112469	-4.37110248	-3.41227832	H	1.80843034	-4.69815532	-4.64667103
C	4.02625643	-3.68612017	-2.30266072	H	5.05790238	-3.83975915	-1.9902285
C	3.22155847	-2.80985674	-1.56458815	H	4.32404446	-1.19369591	-0.65084929
C	3.81580713	-2.12330886	-0.36463565	H	3.05309175	-1.86233801	0.3742236
C	-0.05665781	-3.13733017	-3.4932338	H	4.56364585	-2.76560479	0.11177399
C	4.42013147	-5.29856048	-4.20470915	H	-0.34361255	-2.07933904	-3.5022405
C	-2.76807176	-3.59350325	-0.06184413	H	-0.2226599	-3.55408542	-4.49073057
C	-2.90458459	-4.15552723	1.21141706	H	-0.73664403	-3.64434611	-2.79895799
C	-3.18480077	-5.51048478	1.36186332	H	5.40026858	-5.41804082	-3.73425451
C	-3.34469428	-6.32139224	0.23974971	H	3.96342925	-6.29003833	-4.30319575

C	-3.2414068	-5.76417807	-1.03225071	H	4.57764293	-4.91710635	-5.22104786
C	-2.96261047	-4.40739164	-1.18116005	H	-2.77792515	-3.52469258	2.08829941
H	-4.50582259	0.60729298	-0.6535331	H	-3.28004956	-5.93425208	2.35822238
H	-4.6397846	-1.8131488	-0.17133086	H	-3.56067325	-7.37991739	0.35702385
H	-2.2629914	1.68749975	-0.87518542	H	-3.38325746	-6.38515064	-1.91292372
H	1.88649191	0.0438167	-1.94166216	H	-2.89944857	-3.97162838	-2.17457428
H	-0.24470695	-4.39897371	-0.44211072				
Point Group			C1	ε + ZPE + U_{corr}			-1934.00380626 Eh
Electronic Energy (ε)			-1934.98127958 Eh	$U + H_{corr}$			-1934.00286205 Eh
ε + *ZPE*			-1934.05263214 Eh	$U + G_{corr}$			-1934.12374762 Eh

***E*-2j**

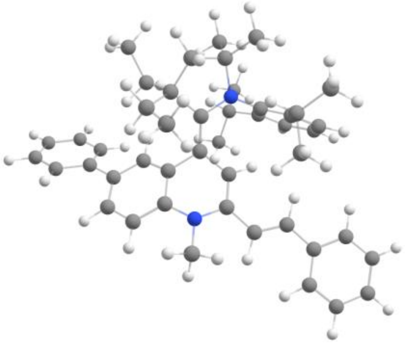

```
%maxcore 2000
%pal nprocs 20
end

!TIGHTOPT FREQ R2SCAN-3c  CPCM
!LargePrint printMOs printbasis

%cpcm
smd true
SMDsolvent "thf"
end
```

Charge: 0 | Multiplicity: 1

	Cartesian Coordinates				Cartesian Coordinates		
**Atoms**	*X*	*Y*	*Z*	**Atoms**	*X*	*Y*	*Z*
C	2.81876683	-1.96994397	-2.52708353	H	-0.83685873	-0.7030755	1.57848361
C	2.00542165	-2.92120702	-3.15448888	H	-2.02466931	-3.30225984	-2.9637828
C	0.68302847	-3.10330321	-2.76354754	H	-1.75946743	-4.51017882	-1.68265294
C	0.11694114	-2.28054175	-1.78063981	H	-3.05910932	-3.29735411	-1.53391918
C	0.87045977	-1.19803566	-1.26261976	H	-2.48043753	-3.88611383	0.52678733
C	2.228421	-1.14275327	-1.55973189	H	-0.74676153	3.36308031	-1.6959449
N	-1.14666196	-2.50158506	-1.24670042	H	0.83060037	4.14856806	-1.70928817
C	-1.34729054	-2.09816567	0.0963885	H	1.61679604	4.97185738	0.60262837
C	-0.68631089	-0.99103861	0.5470966	H	1.32440406	3.92300576	1.99507493
C	0.2012473	-0.26204893	-0.34042684	H	2.31697801	3.3459256	0.64232116
C	-2.03669844	-3.4633816	-1.88144783	H	-1.13499696	4.36023565	1.72760453
C	-2.2223801	-2.90720625	0.92232111	H	-0.6705882	5.39554981	0.37468788
C	0.36864864	1.11147019	-0.38682965	H	-1.90948734	4.16106435	0.13367451
N	-0.11744713	1.99044337	0.5714103	H	0.87844244	0.49715873	-3.20585152
C	0.15199825	3.42720834	0.23561263	H	0.6424646	2.16190627	-3.70556787
C	0.26495949	3.32031911	-1.2798493	H	2.90113247	1.86450196	-0.70966033
C	0.88369057	1.93875796	-1.58904435	H	2.7675027	1.16797148	-2.3066084
C	-0.47026872	1.60516684	1.9033058	H	-1.48599141	0.42715477	-2.31559774
C	1.43714394	3.934845	0.90608877	H	-1.39083332	0.76547501	-4.04904441
C	-0.97000026	4.37727876	0.6454879	H	-1.75232845	2.07042773	-2.92189703
C	0.34811089	1.42046895	-2.94920795	H	2.93867672	4.15780046	-1.67842666
C	2.44480392	2.01518993	-1.69496091	H	4.09394574	3.12702414	-2.522542
C	-1.15351439	1.16325139	-3.05568007	H	2.54962276	3.51735121	-3.28480389
C	3.0264231	3.28045911	-2.32669514	H	-3.21116018	1.27881681	3.89749104
C	-1.83307119	1.62929532	2.28677515	H	-1.47847643	0.56349712	5.50539203
C	-2.16802918	1.26425648	3.5924433	H	0.88093953	0.43740173	4.79893046
C	-1.19804249	0.85155235	4.4954605	H	2.12349761	1.35694933	1.41293565
C	0.12961536	0.78735721	4.09593972	H	2.63648671	2.83244463	3.42291147
C	0.52174288	1.15570781	2.8058668	H	2.90919706	1.36481217	4.37500312
C	1.98449783	0.98720907	2.43109066	H	3.94816201	1.72098295	2.98978396
C	2.91754977	1.77678888	3.35950936	H	1.74050609	-1.08950326	1.77992032
C	2.38650653	-0.49638684	2.43419788	H	2.32945787	-0.91905995	3.44438767
C	-2.96387939	1.94150696	1.31891877	H	3.41996521	-0.60630094	2.08375486
C	-3.79338212	0.68624747	1.01181241	H	-2.51638671	2.26431085	0.37565564
C	-3.88929079	3.05045013	1.83823995	H	-4.2660204	0.28918137	1.91796186
C	-2.73201465	-2.54793038	2.124384	H	-4.58900688	0.93259035	0.29828833
C	-3.5750382	-3.3647117	2.98203802	H	-3.17299588	-0.10012905	0.57233767
C	-4.08910886	-2.78844834	4.16005085	H	-4.59012469	3.35298654	1.05144173
C	-4.910841	-3.5089457	5.01783681	H	-3.33577436	3.9369447	2.16106882
C	-5.24285632	-4.83031188	4.72586398	H	-4.48435923	2.69888915	2.68898624
C	-4.7372	-5.4201807	3.56541157	H	-2.51299786	-1.55581443	2.51715176
C	-3.91468568	-4.70481336	2.70708625	H	-3.83440831	-1.75671738	4.39375193
C	4.24815432	-1.82721691	-2.86809737	H	-5.29378774	-3.0368506	5.91889658
C	5.18784069	-1.44039559	-1.89870635	H	-5.88425847	-5.39734878	5.39454597
C	6.53248288	-1.29613854	-2.22272361	H	-4.9843458	-6.45267228	3.33174704
C	6.97483897	-1.53777705	-3.52181249	H	-3.5284246	-5.1955178	1.81819742
C	6.05411632	-1.92331323	-4.49408735	H	4.86594956	-1.27274159	-0.87423189

C	4.70805443	-2.06280489	-4.17356617	H	7.24091131	-1.00281259	-1.45227724
H	2.42440957	-3.58781501	-3.90324953	H	8.02573735	-1.42598014	-3.77395793
H	0.11161251	-3.91577034	-3.19866069	H	6.38396508	-2.10513427	-5.51375111
H	2.83096516	-0.40899003	-1.04002033	H	4.00046034	-2.33484855	-4.95210679
Point Group			C1	ε + ZPE + U_{corr}			-1893.54924824 Eh
Electronic Energy (ε)			-1894.47553397 Eh	$U + H_{corr}$			-1893.54830404 Eh
ε + *ZPE*			-1893.59528873 Eh	$U + G_{corr}$			-1893.66466231 Eh

Z-2j

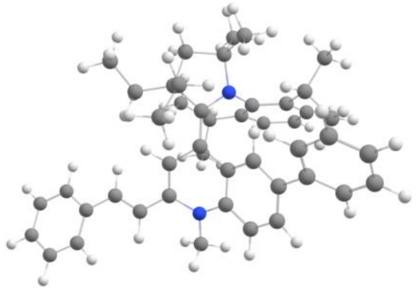

```
%maxcore 2000
%pal nprocs 20
end

!TIGHTOPT FREQ R2SCAN-3c  CPCM
!LargePrint printMOs printbasis

%cpcm
smd true
SMDsolvent "thf"
end
```

Charge: 0 Multiplicity: 1

Atoms	Cartesian Coordinates X	Y	Z	Atoms	Cartesian Coordinates X	Y	Z
C	-3.26090344	-1.59931034	-0.68841782	H	2.37771955	-0.7189639	-0.95199927
C	-3.13690009	-2.91282296	-0.22576414	H	0.49898996	-5.23643394	0.39239365
C	-1.91201052	-3.40053566	0.21189779	H	1.61405912	-4.48142192	1.55886659
C	-0.77428073	-2.58354967	0.20657754	H	-0.12114199	-4.50453059	1.89346176
C	-0.89898727	-1.21079963	-0.1363715	H	2.69479425	-4.31998894	-0.40371539
C	-2.13708251	-0.76437727	-0.59971151	H	1.02354251	4.04370762	-1.52815749
N	0.49027786	-3.09415846	0.47794655	H	1.94650558	3.37902946	-0.17372664
C	1.59840677	-2.4765685	-0.14561928	H	0.26464359	5.19786539	1.02318805
C	1.49070255	-1.17011302	-0.53616595	H	-0.82756925	4.2147018	2.00754415
C	0.31592088	-0.39092883	-0.29015272	H	0.90195736	3.82817421	1.94792312
C	0.6251289	-4.40163808	1.09958323	H	-2.19827097	4.19672141	-0.00420753
C	2.81265396	-3.24243619	-0.3046086	H	-1.01583871	4.85245809	-1.12304843
C	0.36390991	1.00721378	-0.32895535	H	-1.75149668	3.28155034	-1.44879619
N	-0.36825499	1.84804802	0.49318797	H	0.32337949	2.67003636	-3.28125202
C	-0.19406328	3.30981688	0.1240075	H	-0.89537776	1.82672353	-2.34620288
C	1.04380975	3.24747148	-0.78034872	H	3.15932035	1.43754828	-0.94257044
C	1.07401822	1.84859324	-1.41219631	H	2.55855409	0.54124874	-2.33339954
C	-0.832991	1.48001909	1.81767267	H	-0.49465079	0.51552189	-4.30554356
C	0.05149693	4.17623704	1.35705047	H	0.19732274	-0.38976128	-2.95295726
C	-1.37087627	3.92725344	-0.65604219	H	1.25396725	0.49354976	-4.06958098
C	0.14621932	1.7727632	-2.67411124	H	4.14275588	2.22944755	-3.06686249
C	2.52279169	1.51033651	-1.83138688	H	2.55146923	2.66181639	-3.69758593
C	0.29396698	0.52722237	-3.54437487	H	3.22345519	3.53211037	-2.30924421
C	3.13473201	2.5468287	-2.77795078	H	-3.70243625	1.55132765	3.68374277
C	-2.21102882	1.64063613	2.14338073	H	-2.13311378	0.86663653	5.46521123
C	-2.65147993	1.41246969	3.44960449	H	0.24697466	0.58464281	4.92225113
C	-1.78142393	1.027388	4.44965892	H	1.92035338	1.20507347	3.80795905
C	-0.44643157	0.8674899	4.13393944	H	2.88680979	-0.94899799	2.97803158
C	0.07268896	1.07826022	2.84725396	H	1.33667029	-1.15899642	3.81417504
C	1.58694697	0.79245242	2.84593312	H	1.40522207	-1.22898396	2.04738082
C	1.81344052	-0.72871898	2.9276747	H	3.55137563	1.20215982	2.11655614
C	2.51777872	1.40266182	1.80912991	H	2.40623957	2.48539381	1.7315564
C	-3.29998953	2.08151829	1.17867611	H	2.3782182	0.96004008	0.82556144
C	-4.47523936	1.09141906	1.15684852	H	-2.87919129	2.12800701	0.1718922
C	-3.86102595	3.46214422	1.57205501	H	-5.15328678	1.33534998	0.33115054
C	4.06772465	-2.72318851	-0.32275274	H	-4.13526797	0.06288645	1.02937932
C	5.29657421	-3.4579797	-0.5592655	H	-5.05619344	1.14581055	2.08372225
C	6.52846348	-2.7940016	-0.38746475	H	-4.50945549	3.85338141	0.7789176
C	7.73696821	-3.45268032	-0.57371372	H	-3.08139739	4.20038895	1.77815217
C	7.75637052	-4.79778335	-0.93965182	H	-4.46804798	3.37270946	2.48022303
C	6.54598848	-5.46999234	-1.12323208	H	4.19553896	-1.65959452	-0.11955536
C	5.33602185	-4.81492254	-0.9442134	H	6.52417214	-1.74546829	-0.09738639
C	-4.51838768	-1.11799901	-1.29224116	H	8.67019246	-2.91349196	-0.43201773
C	-4.50092467	-0.16977215	-2.32689299	H	8.69999927	-5.31556075	-1.08620892
C	-5.68421095	0.30528633	-2.88232611	H	6.54803125	-6.51632207	-1.41852065
C	-6.91459203	-0.15953379	-2.42288476	H	4.41168001	-5.36043366	-1.11220902
C	-6.94670729	-1.10791297	-1.40174539	H	-3.54952143	0.18639325	-2.71345734

C	-5.7649238	-1.57969772	-0.84199505	H	-5.64416351	1.0361504	-3.68597889
H	-3.98286395	-3.5925829	-0.27531718	H	-7.83894508	0.21173692	-2.85674134
H	-1.83284065	-4.44544411	0.49057347	H	-7.9000586	-1.47338938	-1.02880001
H	-2.21207297	0.26952325	-0.92417718	H	-5.81100081	-2.29156469	-0.02211754
Point Group			C1	ε + ZPE + U_{corr}			-1893.51681028 Eh
Electronic Energy (ε)			-1894.44323194 Eh	$U + H_{corr}$			-1893.51586607 Eh
ε + *ZPE*			-1893.56266483Eh	$U + G_{corr}$			-1893.63143939 Eh

Transition state and E/Z isomer optimization

The ground state energy minimization and the transition state optimization were doine using ORCA 5.0.3.[34] The level of theory is density functional theory (DFT) using the CAM-B3LYP functional[40] and the 6-31G* basis set.[41] Atom-pairwise dispersion correction was applied to the DFT energy with Becke-Johnson damping. The transition state was confirmed by the normal mode analysis and the presence of only one imaginary frequency.

TDDFT Calculations

Time-dependent density functional theory (TD-DFT) calculations were performed at the CAM-B3LYP/def2-TZVPP level of theory[18,40] employing the def2/J auxiliary basis set in the resolution of identity approximation.[42,43] Solvent effects (thf) were included using the CPCM continuum solvation model.[44] The resulting absorption wavelengths shown in chapter *UV-vis switching studies* were linearly scaled by a factor of 1.12 following the procedure reported by Fehér and Stirling.[31]

***E*-2g**

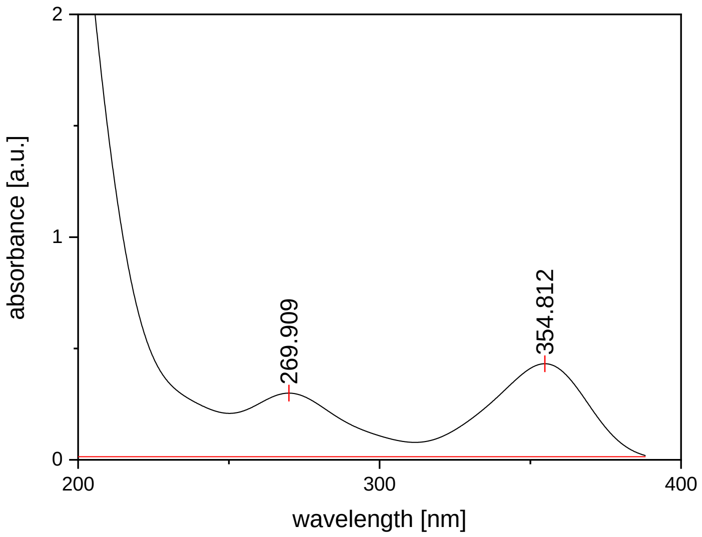

<u>357 nm: Band 1</u>

STATE 1: E= 0.127691 au 3.475 eV 28024.9 cm**-1 <S**2> = 0.000000 Mult 1

156a -> 157a : 0.889547 (c= 0.94315800)

156a -> 159a : 0.012097 (c= 0.10998543)

156a -> 161a : 0.018476 (c= -0.13592701)

156a -> 162a : 0.021451 (c= -0.14646122)

fosc: 0.398641691

<u>334 nm: Band 2</u>

STATE 2: E= 0.136318 au 3.709 eV 29918.3 cm**-1 <S**2> = 0.000000 Mult 1

156a -> 157a : 0.055861 (c= -0.23634871)

156a -> 159a : 0.281480 (c= 0.53054700)

156a -> 160a : 0.061266 (c= 0.24751916)

156a -> 161a : 0.281207 (c= -0.53028982)

156a -> 162a : 0.262548 (c= -0.51239446)

fosc: 0.139635971

HOMO (156a)

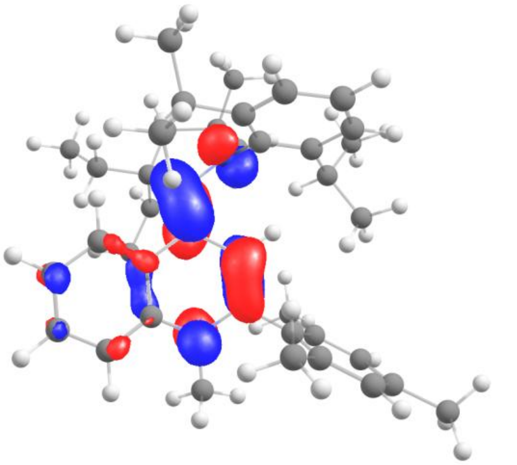

LUMO (157a)

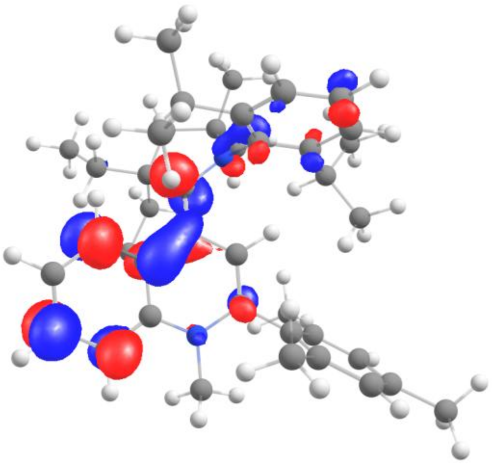

HOMO-1 (155a)

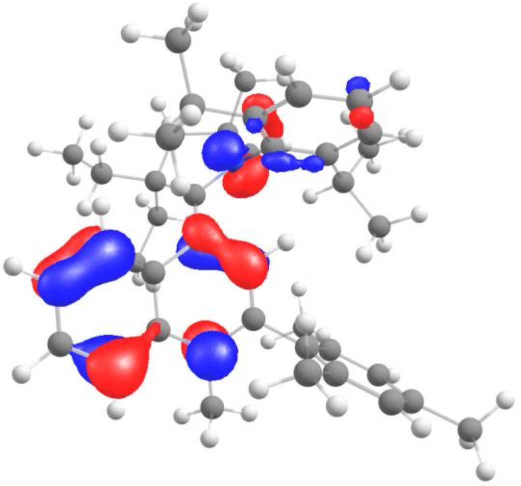

LUMO+1 (158a)

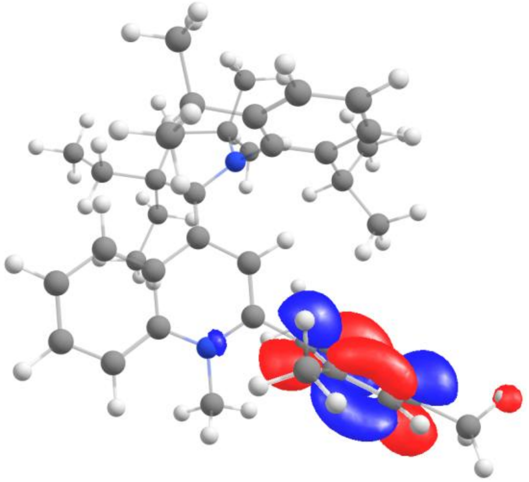

***Z*-2g**

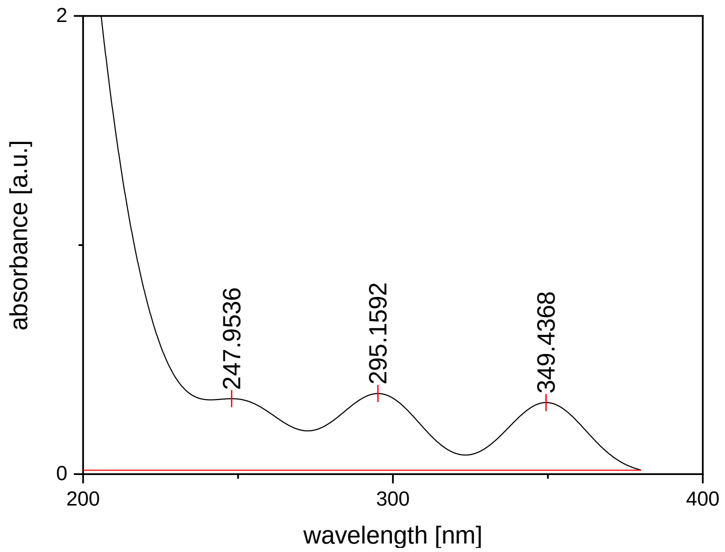

<u>350 nm: Band 1</u>

STATE 1: E= 0.130324 au 3.546 eV 28602.7 cm**-1 <S**2> = 0.000000 Mult 1

156a -> 157a : 0.919580 (c= -0.95894745)

156a -> 158a : 0.021356 (c= 0.14613696)

fosc: 0.310731970

<u>298 nm: Band 3</u>

STATE 3: E= 0.152942 au 4.162 eV 33566.9 cm**-1 <S**2> = 0.000000 Mult 1

156a -> 157a : 0.013349 (c= 0.11553615)

156a -> 158a : 0.734624 (c= 0.85710238)

156a -> 159a : 0.034703 (c= 0.18628640)

156a -> 160a : 0.039302 (c= 0.19824830)

156a -> 161a : 0.097347 (c= -0.31200444)

156a -> 162a : 0.023782 (c= 0.15421523)

fosc: 0.270827228

HOMO (156a)

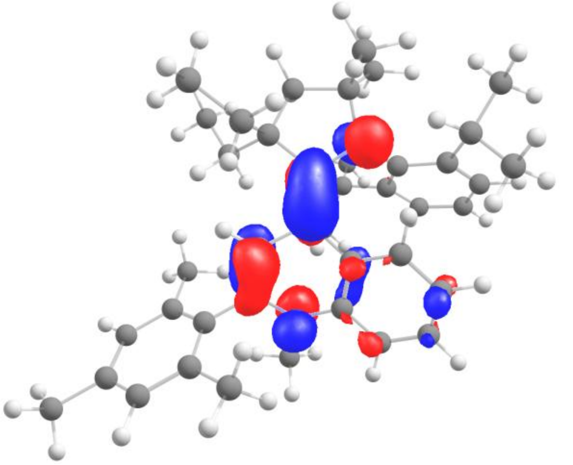

LUMO (157a)

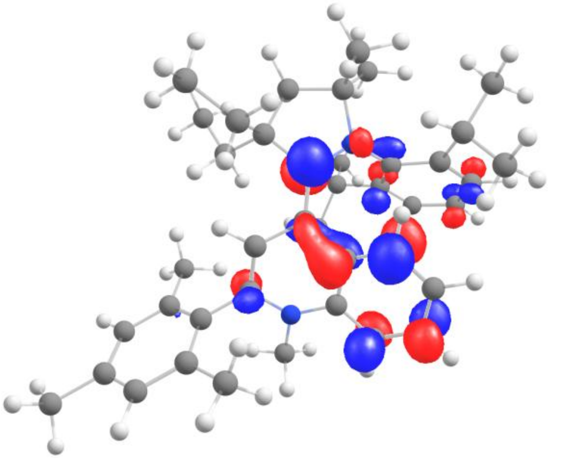

HOMO-1 (155a)

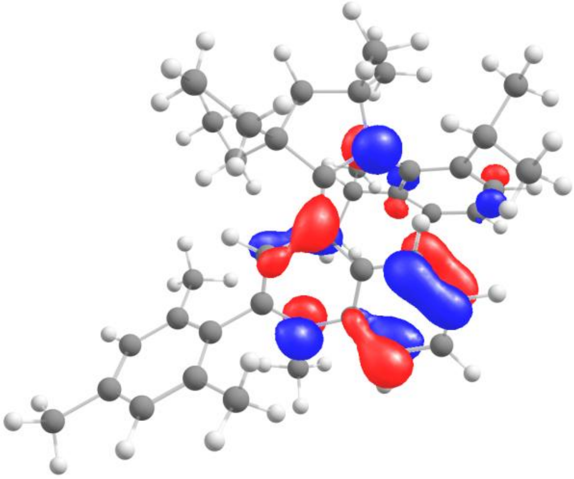

LUMO+1 (158a)

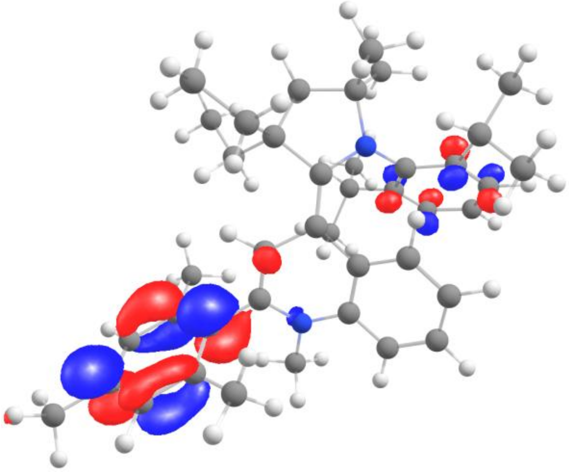

***E*-2h**

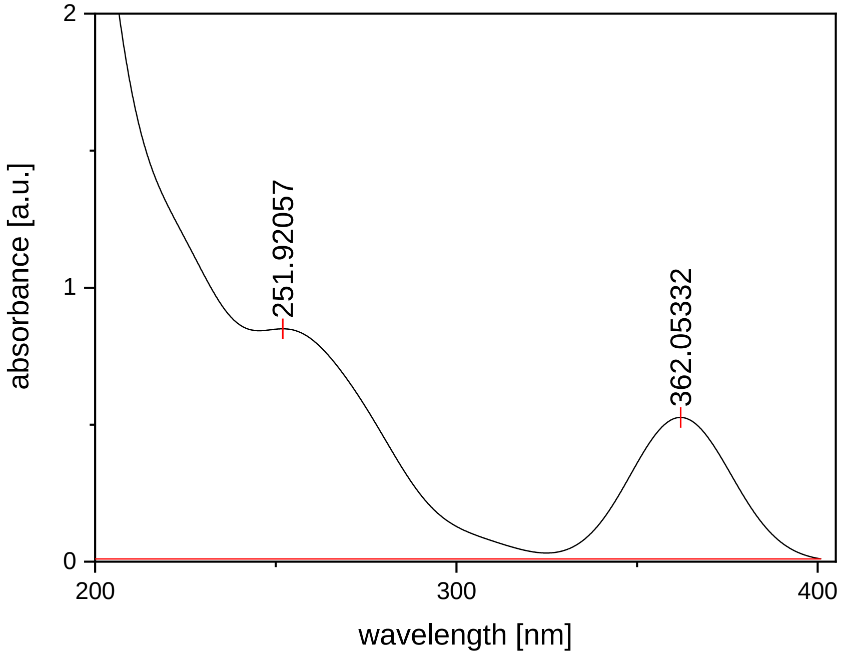

<u>369 nm: Band 1</u>

STATE 1: E= 0.123602 au 3.363 eV 27127.5 cm**-1 <S**2> = 0.000000 Mult 1

176a -> 177a : 0.819351 (c= -0.90518029)

176a -> 184a : 0.100136 (c= -0.31644314)

176a -> 186a : 0.022024 (c= -0.14840605)

fosc: 0.221134194

<u>358 nm: Band 2</u>

STATE 2: E= 0.127205 au 3.461 eV 27918.2 cm**-1 <S**2> = 0.000000 Mult 1

176a -> 177a : 0.013078 (c= 0.11435905)

176a -> 178a : 0.871942 (c= -0.93377826)

176a -> 180a : 0.019441 (c= -0.13943035)

176a -> 181a : 0.017065 (c= 0.13063455)

176a -> 184a : 0.027459 (c= -0.16570872)

fosc: 0.348264393

HOMO (176a)

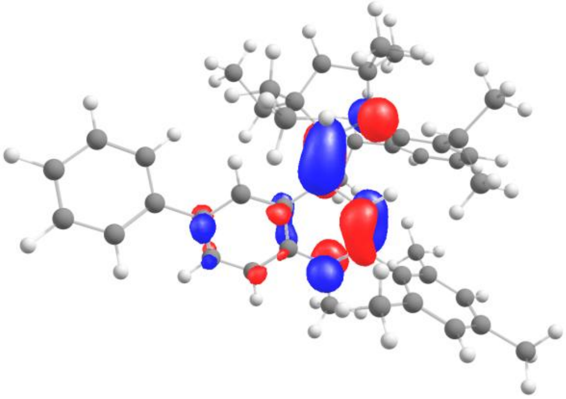

LUMO (177a)

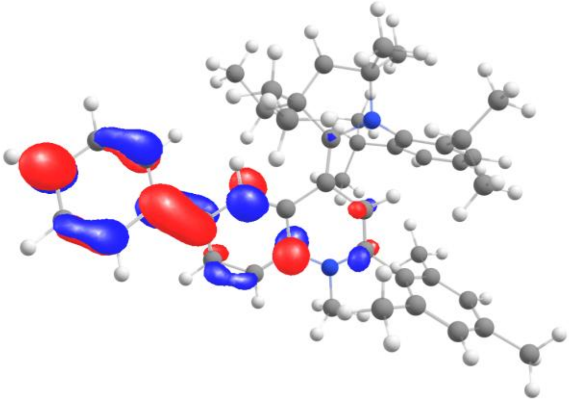

HOMO-1 (175a)

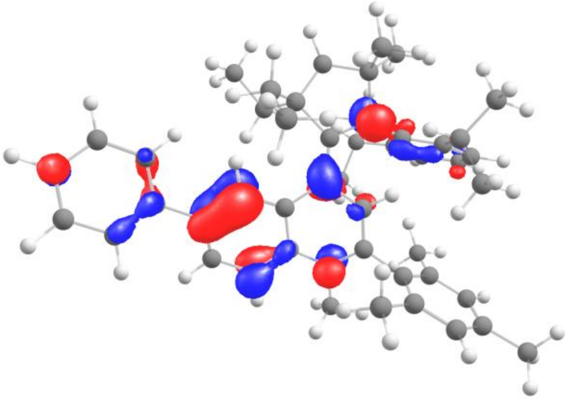

LUMO+1 (178a)

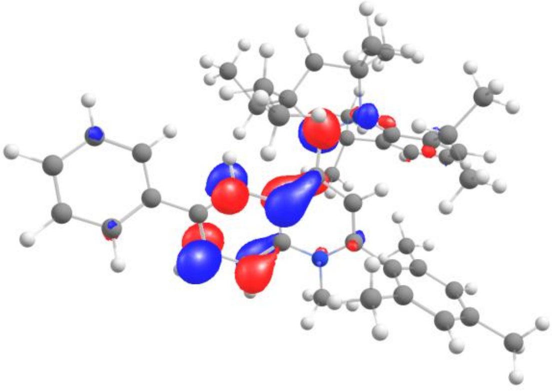

***Z*-2h**

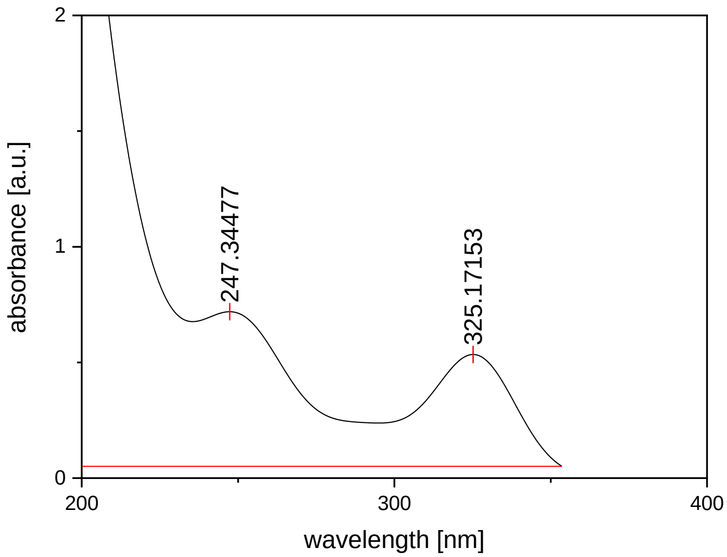

<u>328 nm: Band 1</u>

STATE 1: E= 0.139111 au 3.785 eV 30531.3 cm**-1 <S**2> = 0.000000 Mult 1

176a -> 177a : 0.307553 (c= -0.55457458)

176a -> 178a : 0.562595 (c= -0.75006348)

176a -> 180a : 0.028837 (c= -0.16981594)

176a -> 184a : 0.013363 (c= 0.11559818)

176a -> 186a : 0.020012 (c= 0.14146541)

fosc: 0.284275549

<u>324 nm: Band 2</u>

STATE 2: E= 0.140708 au 3.829 eV 30881.8 cm**-1 <S**2> = 0.000000 Mult 1

176a -> 177a : 0.499055 (c= 0.70643828)

176a -> 178a : 0.282664 (c= -0.53166128)

176a -> 179a : 0.016060 (c= -0.12672983)

176a -> 180a : 0.056682 (c= -0.23808033)

176a -> 184a : 0.057996 (c= -0.24082260)

176a -> 185a : 0.014031 (c= 0.11845114)

176a -> 186a : 0.025309 (c= -0.15908769)

0.245820915

HOMO (176a)

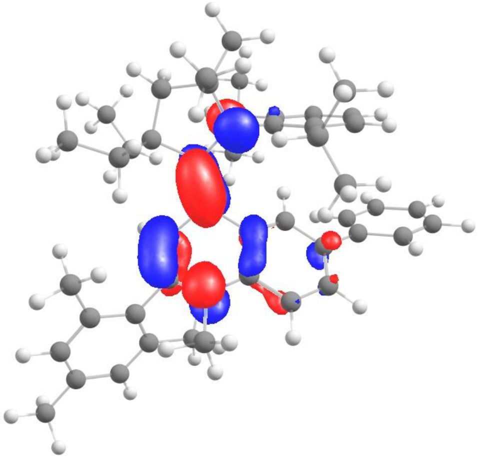

LUMO (177a)

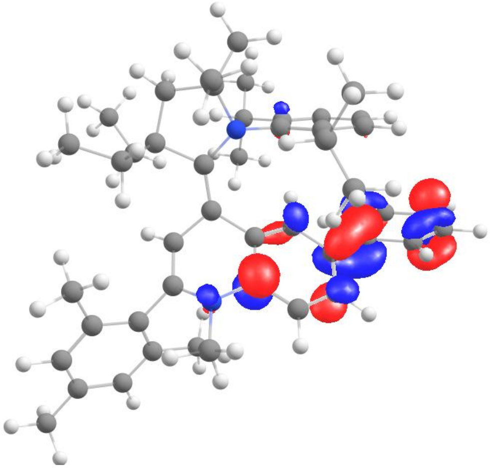

HOMO-1 (175a)

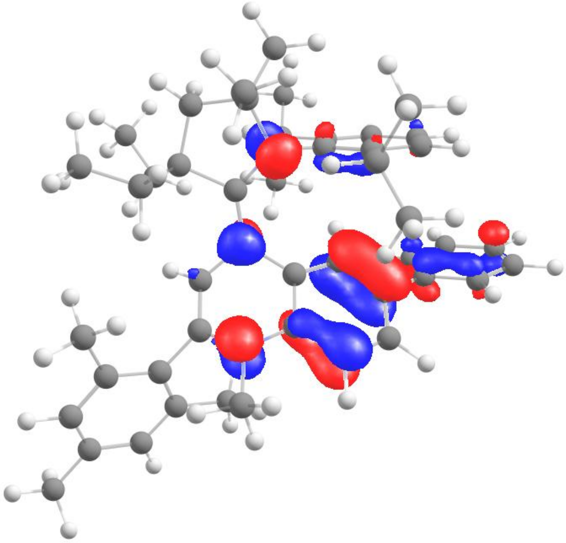

LUMO+1 (178a)

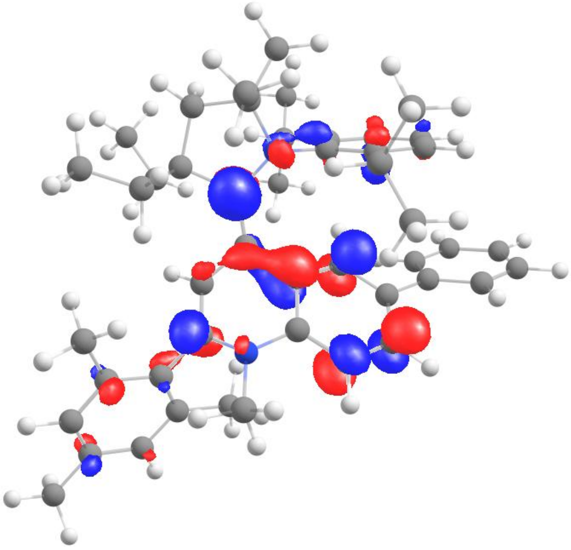

***E*-2i**

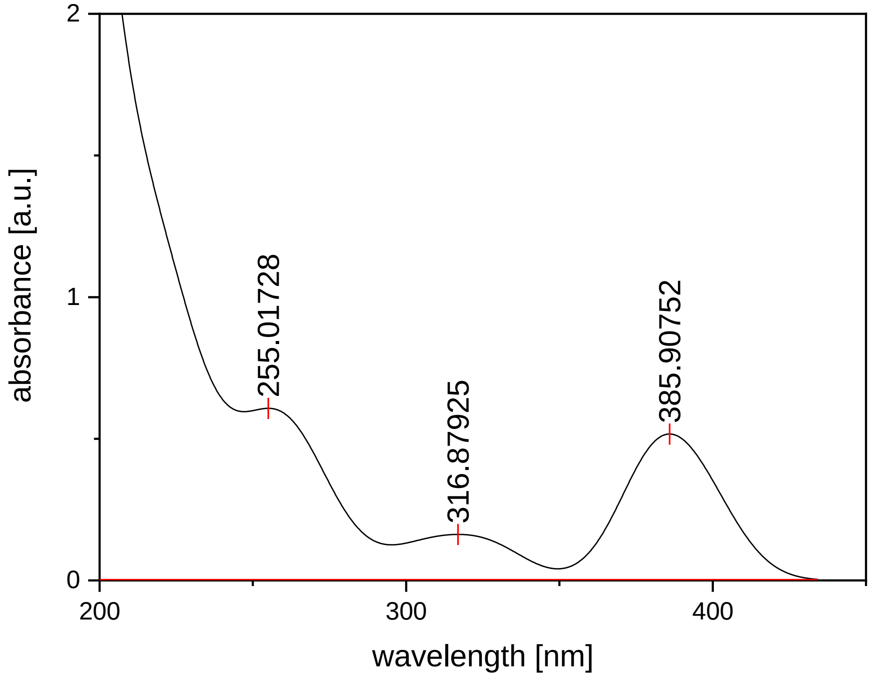

398 nm: Band 150

STATE 1: E= 0.114550 au 3.117 eV 25140.8 cm**-1 <S**2> = 0.000000 Mult 1

176a -> 177a : 0.540455 (c= 0.73515646)

176a -> 178a : 0.152691 (c= -0.39075690)

176a -> 179a : 0.170486 (c= -0.41289963)

176a -> 180a : 0.019407 (c= 0.13930919)

176a -> 183a : 0.027927 (c= -0.16711409)

176a -> 184a : 0.033923 (c= 0.18418181)

fosc: 0.216722343

382 nm: Band 149

STATE 2: E= 0.119429 au 3.250 eV 26211.6 cm**-1 <S**2> = 0.000000 Mult 1

176a -> 177a : 0.367789 (c= -0.60645609)

176a -> 178a : 0.378311 (c= -0.61506989)

176a -> 179a : 0.178486 (c= -0.42247553)

176a -> 186a : 0.010420 (c= 0.10208066)

fosc: 0.399877170

327 nm: Band 148

STATE 3: E= 0.139427 au 3.794 eV 30600.6 cm**-1 <S**2> = 0.000000 Mult 1

175a -> 179a : 0.010525 (c= -0.10259244)

176a -> 178a : 0.363889 (c= -0.60323192)

176a -> 179a : 0.442268 (c= 0.66503226)

176a -> 180a : 0.010129 (c= -0.10064408)

176a -> 181a : 0.019966 (c= -0.14129942)

176a -> 182a : 0.023066 (c= -0.15187457)

176a -> 183a : 0.029674 (c= 0.17226042)

176a -> 186a : 0.030870 (c= -0.17569724)

fosc: 0.114397704

HOMO (176a)

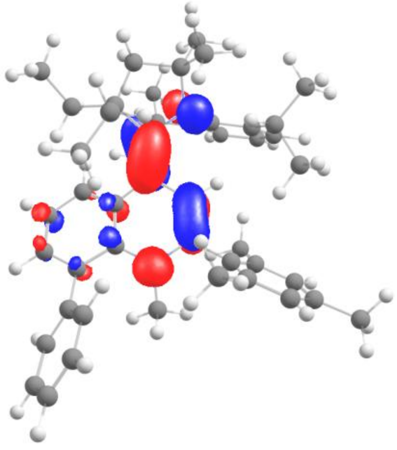

LUMO (177a)

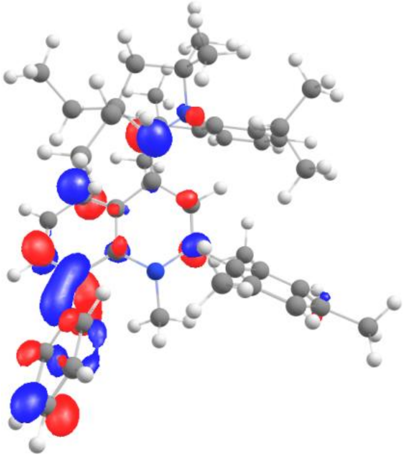

HOMO-1 (175a)

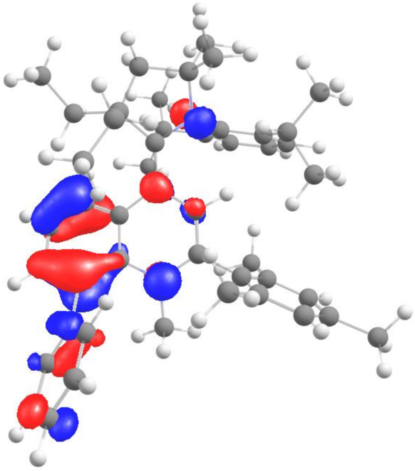

LUMO+1 (178a)

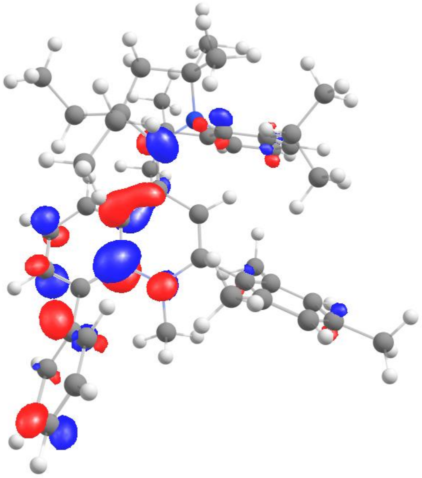

Z-2i

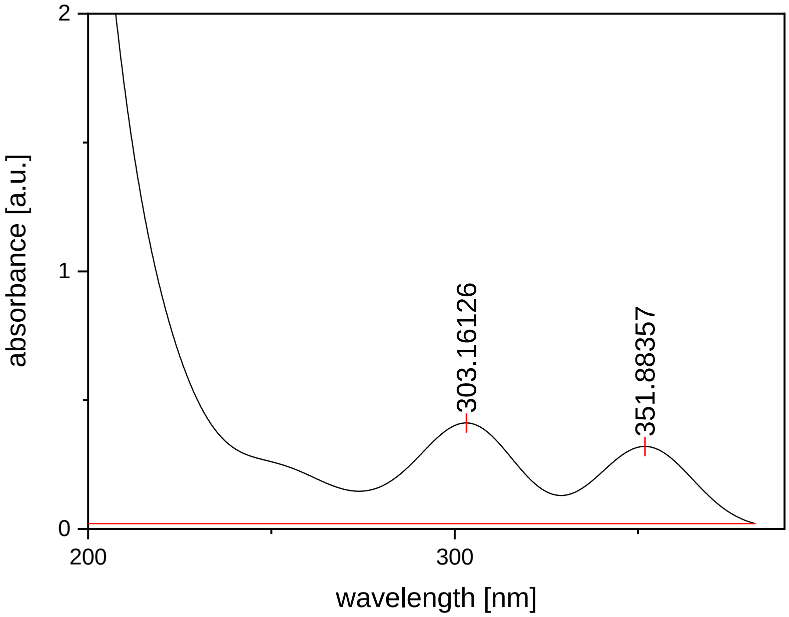

352 nm: Band 150

STATE 1: E= 0.129333 au 3.519 eV 28385.4 cm**-1 <S**2> = 0.000000 Mult 1

176a -> 177a : 0.791985 (c= 0.88993533)

176a -> 178a : 0.087358 (c= -0.29556354)

176a -> 179a : 0.026476 (c= -0.16271301)

176a -> 180a : 0.025739 (c= 0.16043431)

176a -> 183a : 0.015827 (c= 0.12580352)

fosc: 0.312365332

305 nm: Band 148

STATE 3: E= 0.149574 au 4.070 eV 32827.6 cm**-1 <S**2> = 0.000000 Mult 1

176a -> 177a : 0.068621 (c= 0.26195546)

176a -> 178a : 0.022425 (c= 0.14974817)

176a -> 179a : 0.471352 (c= 0.68655044)

176a -> 180a : 0.335514 (c= -0.57923581)

176a -> 183a : 0.022055 (c= 0.14850876)

176a -> 184a : 0.016569 (c= 0.12872035)

fosc: 0.369180146

HOMO (176a)

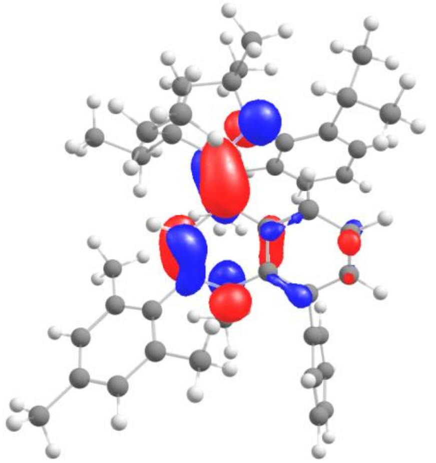

LUMO (177a)

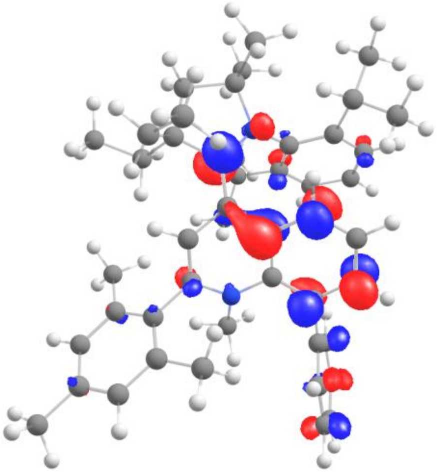

HOMO-1 (175a)

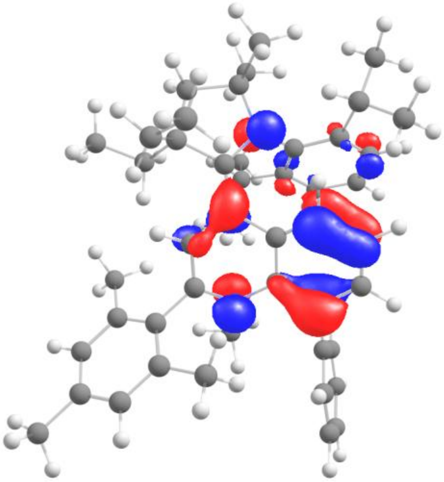

LUMO+1 (178a)

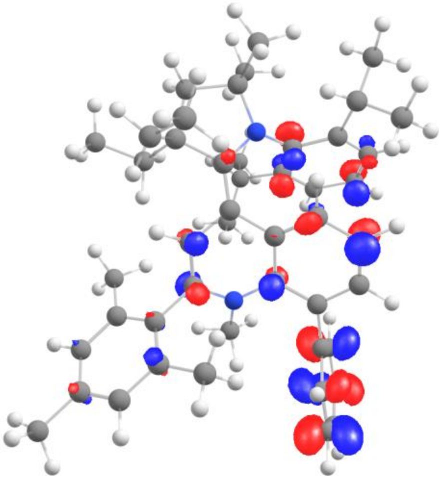

***E*-2j**

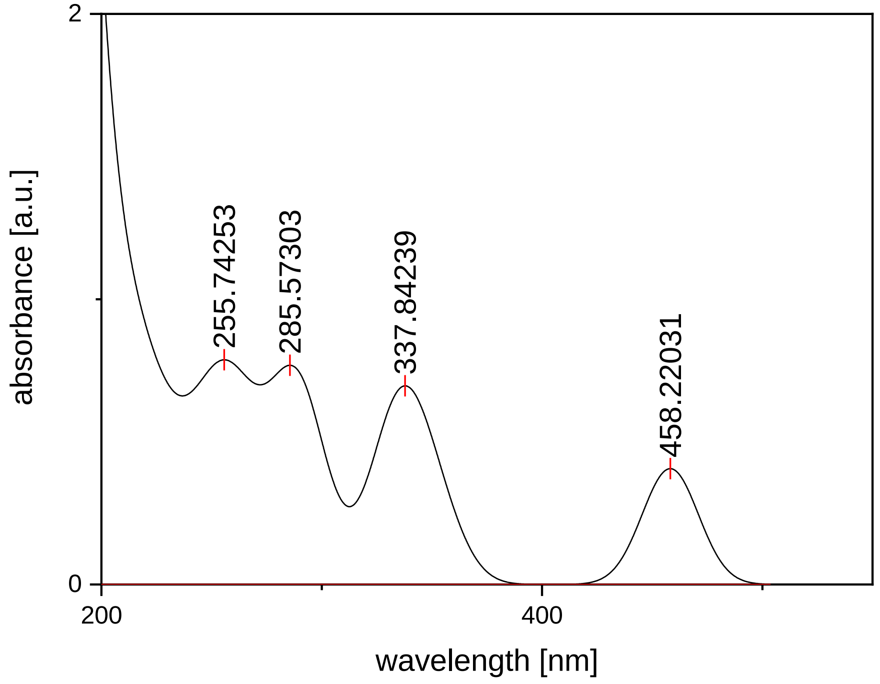

<u>458 nm: Band 150</u>

STATE 1: E= 0.099449 au 2.706 eV 21826.5 cm**-1 <S**2> = 0.000000 Mult 1

171a -> 172a : 0.918180 (c= 0.95821721)

171a -> 173a : 0.012909 (c= -0.11361753)

171a -> 179a : 0.023860 (c= 0.15446598)

fosc: 0.405988655

<u>351 nm: Band 149</u>

STATE 2: E= 0.129823 au 3.533 eV 28492.9 cm**-1 <S**2> = 0.000000 Mult 1

171a -> 172a : 0.014285 (c= -0.11951885)

171a -> 173a : 0.463534 (c= -0.68083312)

171a -> 174a : 0.354760 (c= -0.59561700)

171a -> 176a : 0.026259 (c= 0.16204646)

171a -> 178a : 0.017647 (c= -0.13284370)

171a -> 179a : 0.026909 (c= 0.16403978)

171a -> 181a : 0.017559 (c= -0.13251074)

fosc: 0.239723460

335 nm: Band 148

STATE 3: E=   0.136134 au   3.704 eV   29877.9 cm**-1   <S**2> =   0.000000 Mult 1

170a -> 172a :   0.018866 (c= 0.13735217)

171a -> 173a :   0.375648 (c= -0.61290128)

171a -> 174a :   0.447536 (c= 0.66898145)

171a -> 177a :   0.014764 (c= -0.12150820)

171a -> 178a :   0.030936 (c= 0.17588574)

171a -> 181a :   0.023549 (c= -0.15345840)

fosc: 0.569019814

HOMO (171a)

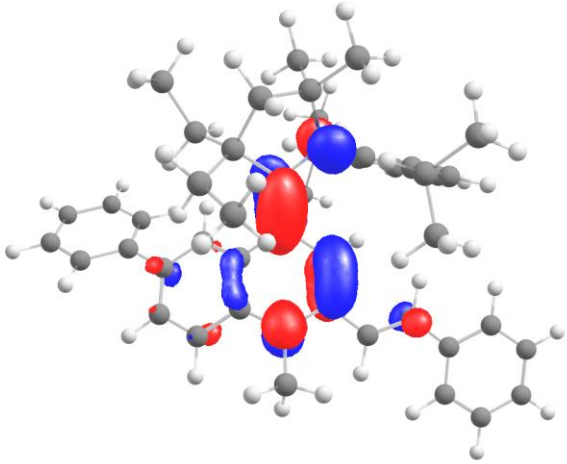

LUMO (172a)

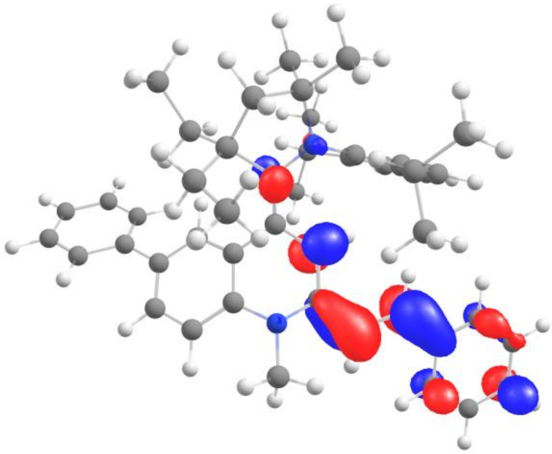

HOMO-1 (170a)

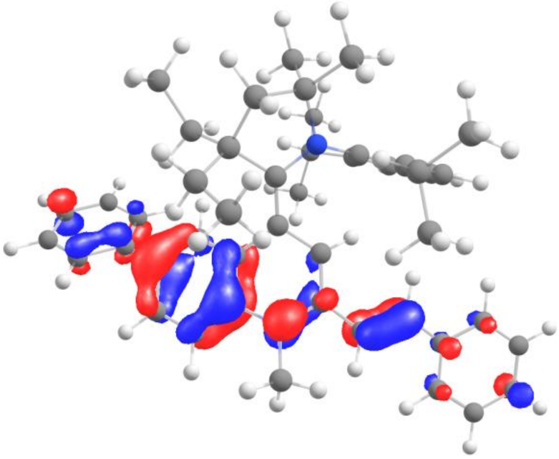

LUMO+1 (173a)

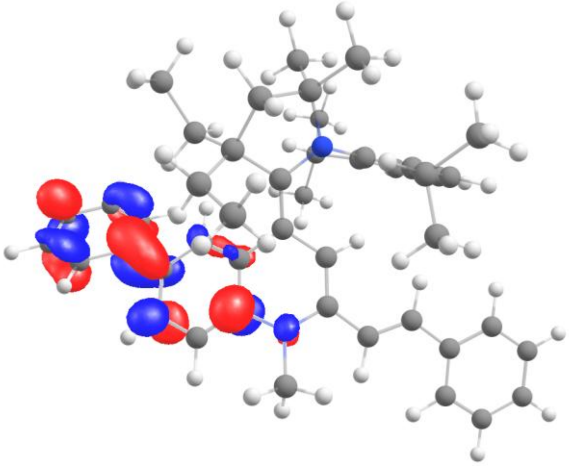

***Z*-2j**

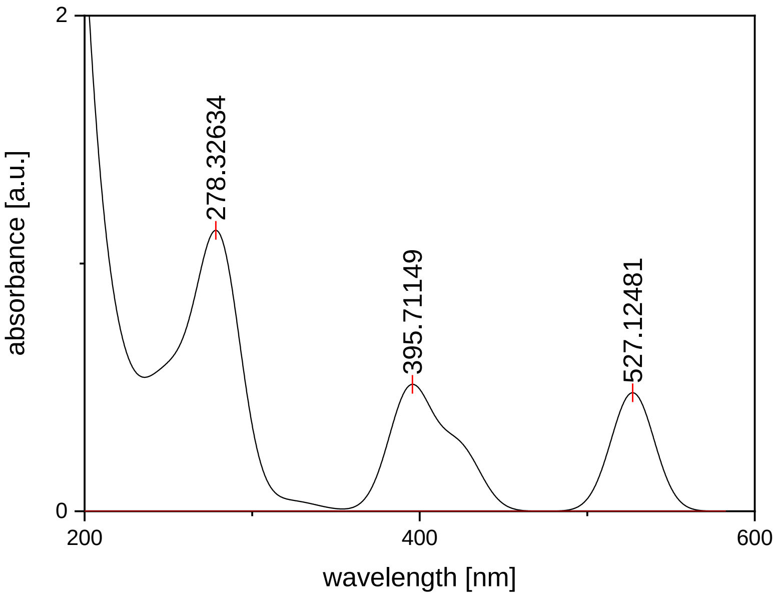

<u>527 nm: Band 150</u>

STATE 1: E= 0.086445 au 2.352 eV 18972.4 cm**-1 <S**2> = 0.000000 Mult 1

171a -> 172a : 0.879757 (c= -0.93795365)

171a -> 173a : 0.043220 (c= 0.20789525)

171a -> 179a : 0.023742 (c= -0.15408580)

fosc: 0.478310938

<u>424 nm: Band 149</u>

STATE 2: E= 0.107538 au 2.926 eV 23601.9 cm**-1 <S**2> = 0.000000 Mult 1

171a -> 172a : 0.030175 (c= 0.17371042)

171a -> 173a : 0.145022 (c= 0.38081773)

171a -> 174a : 0.719093 (c= -0.84799347)

171a -> 179a : 0.031718 (c= -0.17809416)

fosc: 0.249498400

395 nm: Band 148

STATE 3: E=   0.115510 au   3.143 eV   25351.6 cm**-1   <S**2> =   0.000000 Mult 1

171a -> 172a :   0.032237 (c= -0.17954576)

171a -> 173a :   0.680174 (c= -0.82472684)

171a -> 174a :   0.188055 (c= -0.43365332)

171a -> 180a :   0.027606 (c= 0.16615011)

171a -> 181a :   0.020622 (c= -0.14360406)

fosc: 0.492138527

HOMO (171a)

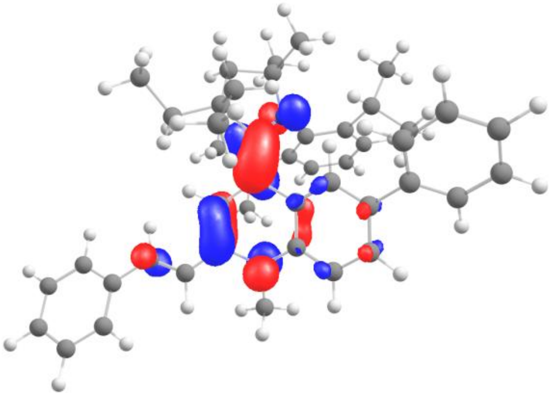

LUMO (172a)

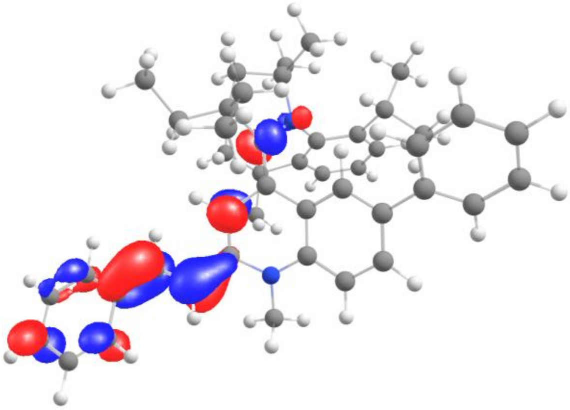

HOMO-1 (170a)

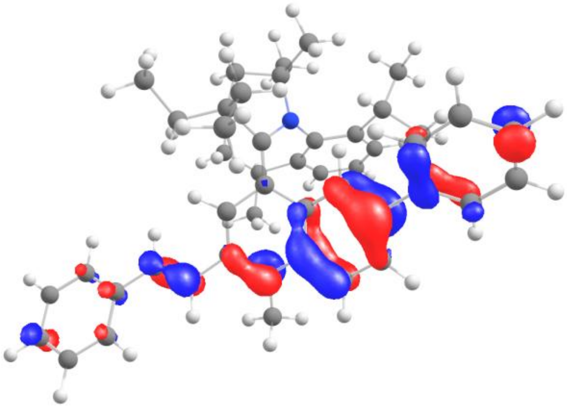

LUMO+1 (173a)

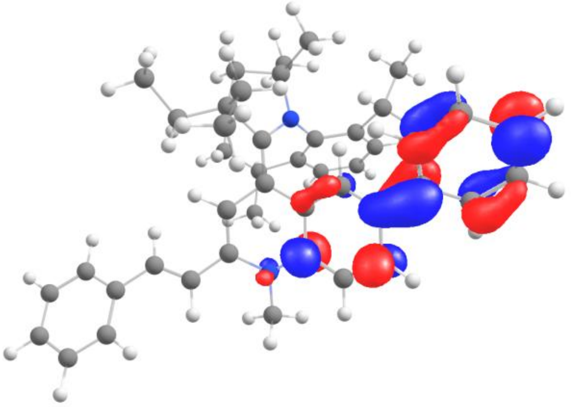

Electron density difference

The difference between the electron density in the excited state (S_1) and the ground state was calculated at CAM-B3LYP/6-31G* level of theory using ORCA 5.0.3. Negative and positive values are colored in red and green, respectively.

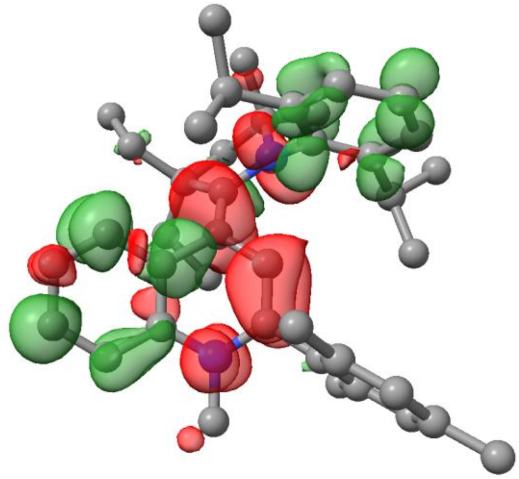

Figure S280: Electron density difference ($S_1 - S_0$) of ***E*-2g**. Hydrogen atoms are omitted for clarity.

Nonadiabatic molecular dynamics simulations

The photoisomerization of the 2g compound was studied by nonadiabatic molecular dynamics simulations. The quantum chemical method for this was hole–hole Tamm–Dancoff-approximated density functional theory (hh-TDA-DFT) with 6-31G*basis set as implemented in TeraChem.[45] The time step was chosen to the 1.0 fs. To account for transition from the excited to the ground state we used Tully's surface hopping algorithm.[46,47]

In total 60 trajectories have been calculated which showed 32 cases of successful isomerization from *E* to *Z* isomer (Figure S279). In all the cases the rotation of the central double bond was anti clockwise, in the same direction. This is shown in the figure below, where the evolution of the dihedral in each individual trajectory is represented by one line.

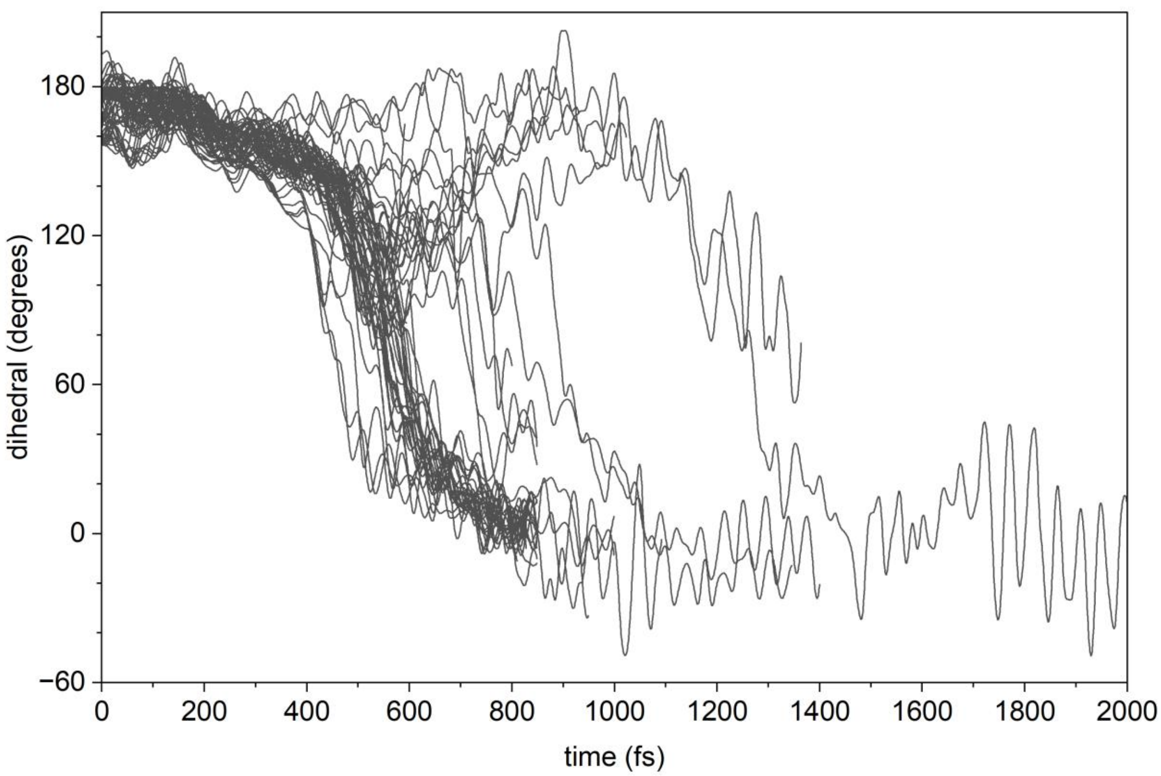

Figure S281: Plot of the trajectory calculations.

References

[1] V. Lavallo, Y. Canac, C. Präsang, B. Donnadieu, G. Bertrand, *Angew. Chem. Int. Ed.* **2005**, *44*, 5705.

[2] L.-Y. Xi, R.-Y. Zhang, L. Zhang, S.-Y. Chen, X.-Q. Yu, *Org. Biomol. Chem.* **2015**, *13*, 3924.

[3] C. Win-Long, C. Yung-Chi, US2019181356A1, **2019**.

[4] J. Preindl, S. Chakrabarty, J. Waser, *Chem. Sci.* **2017**, *8*, 7112.

[5] A. Banik, P. Datta, S. K. Mandal, *Org. Lett.* **2023**, *25*, 1305.

[6] A. Flahaut, S. Roland, P. Mangeney, *J. Organomet. Chem.* **2007**, *692*, 5754.

[7] D. Magis, J. J. Cabrera-Trujillo, J. Vignolle, J.-M. Sotiropoulos, D. Taton, K. Miqueu, Y. Landais, *J. Am. Chem. Soc.* **2024**.

[8] P. W. Antoni, A. Behnke, C. Golz, M. M. Hansmann, *J. Org. Chem.* **2025**, *90*, 9108.

[9] F. Hu, X. Lei, *Tetrahedron* **2014**, *70*, 3854.

[10] S. E. Wengryniuk, A. Weickgenannt, C. Reiher, N. A. Strotman, K. Chen, M. D. Eastgate, P. S. Baran, *Org. Lett.* **2013**, *15*, 792.

[11] Y. Yan, K. Xu, Y. Fang, Z. Wang, *J. Org. Chem.* **2011**, *76*, 6849.

[12] E. Talsi, *J. Mol. Catal. A Chem.* **1999**, *139*, 131.

[13] S. Stoll, A. Schweiger, *J. Magn. Reson.* **2006**, *178*, 42.

[14] P. Hohenberg, W. Kohn, *Phys. Rev.* **1964**, *136*, B864-B871.

[15] W. Kohn, L. J. Sham, *Phys. Rev.* **1965**, *140*, A1133-A1138.

[16] Gaussian 16, Revision C.01, M. J. Frisch, G. W. Trucks, H. B. Schlegel, G. E. Scuseria, M. A. Robb, J. R. Cheeseman, G. Scalmani, V. Barone, G. A. Petersson, H. Nakatsuji, X. Li, M. Caricato, A. V. Marenich, J. Bloino, B. G. Janesko, R. Gomperts, B. Mennucci, H. P. Hratchian, J. V. Ortiz, A. F. Izmaylov, J. L. Sonnenberg, D. Williams-Young, F. Ding, F. Lipparini, F. Egidi, J. Goings, B. Peng, A. Petrone, T. Henderson, D. Ranasinghe, V. G. Zakrzewski, J. Gao, N. Rega, G. Zheng, W. Liang, M. Hada, M. Ehara, K. Toyota, R. Fukuda, J. Hasegawa, M. Ishida, T. Nakajima, Y. Honda, O. Kitao, H. Nakai, T. Vreven, K. Throssell, J. A. Montgomery, Jr., J. E. Peralta, F. Ogliaro, M. J. Bearpark, J. J. Heyd, E. N. Brothers, K. N. Kudin, V. N. Staroverov, T. A. Keith, R. Kobayashi, J. Normand, K. Raghavachari, A. P. Rendell, J. C. Burant, S. S. Iyengar, J. Tomasi, M. Cossi, J. M. Millam, M. Klene, C. Adamo, R. Cammi, J. W. Ochterski, R. L. Martin, K. Morokuma, O. Farkas, J. B. Foresman, D. J. Fox, Gaussian, Inc., Wallingford CT., **2016**.

[17] F. Weigend, *Phys. Chem. Chem. Phys.* **2006**, *8*, 1057.

[18] F. Weigend, R. Ahlrichs, *Phys. Chem. Chem. Phys.* **2005**, *7*, 3297.

[19] S. Grimme, J. Antony, S. Ehrlich, H. Krieg, *J. Chem. Phys.* **2010**, *132*, 154104.

[20] S. Grimme, S. Ehrlich, L. Goerigk, *J. Comput. Chem.* **2011**, *32*, 1456.

[21] G. Knizia, *J. Chem. Theory Comput.* **2013**, *9*, 4834.

[22] GaussView, Version 6.1, Roy Dennington, Todd A. Keith, and John M. Millam, Semichem Inc., Shawnee Mission, KS., **2016**.

[23] Chemcraft - graphical software for visualization of quantum chemistry computations. Version 1.8, build 682. https://www.chemcraftprog.com.

[24] https://zenodo.org/records/18281325, last visited: 04.02.2026.

[25] Bruker, SAINT, V8.40A, Bruker AXS Inc., Madison, Wisconsin, USA.

[26] L. Krause, R. Herbst-Irmer, G. M. Sheldrick, D. Stalke, *J. Appl. Crystallogr.* **2015**, *48*, 3.

[27] G. M. Sheldrick, *Acta Crystallogr. C Struct. Chem.* **2015**, *71*, 3.

[28] G. M. Sheldrick, *Acta Crystallogr. A Found Adv.* **2015**, *71*, 3.

[29] C. R. Groom, I. J. Bruno, M. P. Lightfoot, S. C. Ward, *Acta Crystallogr. B Struct. Sci. Cryst. Eng. Mater.* **2016**, *72*, 171.
[30] D. Kratzert, FinalCif, V152, https://dkratzert.de/finalcif.html.
[31] P. P. Fehér, Á. Madarász, A. Stirling, *Chemistry Methods* **2023**, *3*.
[32] A. Goulet-Hanssens, C. Rietze, E. Titov, L. Abdullahu, L. Grubert, P. Saalfrank, S. Hecht, *Chem* **2018**, *4*, 1740.
[33] B. P. Roberts, J. N. Winter, *J. Chem. Soc., Chem. Commun.* **1978**, 545.
[34] F. Neese, *WIREs Comput. Mol. Sci.* **2022**, *12*.
[35] F. Neese, *WIREs Comput. Mol. Sci.* **2025**, *15*.
[36] C. Bannwarth, E. Caldeweyher, S. Ehlert, A. Hansen, P. Pracht, J. Seibert, S. Spicher, S. Grimme, *WIREs Comput. Mol. Sci.* **2021**, *11*.
[37] C. Bannwarth, S. Ehlert, S. Grimme, *J. Chem. Theory Comput.* **2019**, *15*, 1652.
[38] S. Grimme, A. Hansen, S. Ehlert, J.-M. Mewes, *J. Chem. Phys.* **2021**, *154*, 64103.
[39] A. V. Marenich, C. J. Cramer, D. G. Truhlar, *J. Phys. Chem. B* **2009**, *113*, 6378.
[40] T. Yanai, D. P. Tew, N. C. Handy, *Chem. Phys. Lett.* **2004**, *393*, 51.
[41] V. A. Rassolov, J. A. Pople, M. A. Ratner, T. L. Windus, *J. Chem. Phys.* **1998**, *109*, 1223.
[42] B. Helmich-Paris, B. de Souza, F. Neese, R. Izsák, *J. Chem. Phys.* **2021**, *155*, 104109.
[43] F. Neese, G. Olbrich, *Chem. Phys. Lett.* **2002**, *362*, 170.
[44] M. Garcia-Ratés, F. Neese, *J. Comput. Chem.* **2020**, *41*, 922.
[45] S. Seritan, C. Bannwarth, B. S. Fales, E. G. Hohenstein, C. M. Isborn, S. I. L. Kokkila-Schumacher, X. Li, F. Liu, N. Luehr, J. W. Snyder et al., *WIREs Comput. Mol. Sci.* **2021**, *11*.
[46] J. C. Tully, *J. Chem. Phys.* **1990**, *93*, 1061.
[47] S. Hammes-Schiffer, J. C. Tully, *J. Chem. Phys.* **1994**, *101*, 4657.
